# Dispersion‐Enhanced Nitrogen‐Centered Photocatalysis of the Direct Hydrogen Atom Transfer

**DOI:** 10.1002/anie.202522022

**Published:** 2025-12-18

**Authors:** Jiasong Zhang, Kaitong Zhuang, Ramon Trevino, Babu Raj Dhungana, Huiying Sun, Shuyu Yin, Yuting Li, Jacob A. Sanchez, Xiaoyu Xia, Ramy Elerian, Yao Sun, Seth O. Fremin, Chao Huang, Min He, Maosheng Cheng, Oleg V. Larionov, Shengfei Jin

**Affiliations:** ^1^ Wuya College of Innovation Shenyang Pharmaceutical University Shenyang 110016 P.R. China; ^2^ Department of Chemistry The University of Texas at San Antonio One UTSA Circle San Antonio TX 78249 USA; ^3^ Key Laboratory of Structure‐Based Drug Design and Discovery, Ministry of Education, School of Pharmaceutical Engineering Shenyang Pharmaceutical University Shenyang 110016 P.R. China

**Keywords:** Acridine, Hydrogen atom transfer, Noncovalent interactions, Photocatalysis, Radical reactions

## Abstract

The development of structurally distinct and modular photocatalysts for direct hydrogen atom transfer (HAT) has emerged as a key frontier in radical‐mediated C–H functionalization of strong carbon–hydrogen bonds. Despite the largely untapped potential across the fields of organic synthesis, medicinal chemistry, and materials science, few classes of direct HAT photocatalysts are currently known, and the approaches to enhancing their photocatalytic activity remain underdeveloped. We report herein the development of nitrogen‐centered direct HAT photocatalysts based on the acridine framework, which leverage a C9‐*ortho*‐biaryl substituent for enhancing the photocatalytic activity through stabilizing dispersion interactions with the substrate as the key steering effect. The acridine direct HAT photocatalysis enables an array of carbon–carbon and carbon–heteroatom bond‐forming reactions in diverse structural and experimental settings, including cryogenic conditions. It also provides a blueprint for enhancing the photocatalytic activity of direct HAT catalysts by leveraging photocatalyst‐substrate dispersion interactions.

## Graphical abstract

Nitrogen‐centered acridine photocatalysts for direct hydrogen atom transfer have been developed, leveraging dispersion interactions to enhance photocatalytic activity. This approach enables diverse functionalization reactions of strong C─H bonds even under cryogenic conditions and provides a new strategy for boosting the efficiency of direct HAT photocatalysis.

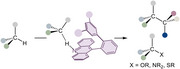

## Introduction

**Figure 1:**
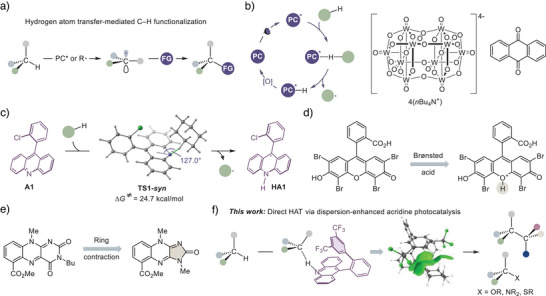
Direct hydrogen atom transfer by dispersion‐enhanced acridine photocatalysis. a). Hydrogen atom transfer‐mediated C–H functionalization. b). The generalized catalytic cycle for direct HAT photocatalysis and examples of well‐established direct HAT photocatalysts. c). Computational studies of the direct HAT process mediated by acridine **A1** with cyclohexane as a substrate. d). Enhancement of direct HAT photocatalytic activity by Brønsted acid. e). Enhancement of direct HAT photocatalytic activity by ring contraction. f). Development of dispersion‐enhanced acridine photocatalysis for direct HAT C–H functionalization.

Hydrogen atom transfer (HAT) has emerged as a major strategy for the functionalization of carbon–hydrogen bonds with applications in medicinal chemistry, materials science, and organic synthesis.^[^ ^1^, ^2^, ^3^, ^4^, ^5^, ^6^, ^7^, ^8^, ^9^ ^]^ This strategy enables efficient introduction of diverse functionalities and obviates ancillary multistep synthetic manipulations. Despite the recent progress, the generation of reactive species that can mediate the cleavage of strong C─H bonds remains challenging and can require harsh conditions and unselective HAT mediators.^[^ ^1^, ^9^ ^]^ To that end, photocatalysis can provide the thermodynamic driving force for the HAT process by leveraging the energy of visible light (Figure 1a). This approach can also open the door for the systematic manipulation of HAT efficiency and chemoselectivity through rational catalyst design.^[^ ^3^, ^9^, ^10^, ^11^, ^12^, ^13^, ^14^, ^15^, ^16^, ^17^, ^18^, ^19^, ^20^, ^21^, ^22^, ^23^, ^24^, ^25^, ^26^, ^27^ ^]^ In this context, direct HAT photocatalysis, wherein the excited state of the photocatalyst abstracts a hydrogen atom from the substrate, can provide a chemoselective and tunable approach for C–H functionalization in complex structural settings.^[^ ^9^ ^]^ Despite the potential advantages, the approach remains underdeveloped because of the dearth of structurally and mechanistically distinct direct HAT photocatalysts (Figure 1b). In this regard, oxygen‐centered photocatalysts that promote direct HAT via the formation of an O─H bond, such as metal oxides^[^ ^28^, ^29^ ^]^ and aromatic ketones,^[^ ^30^, ^31^, ^32^, ^33^ ^]^ are well‐established and occupy a privileged position. In contrast, other types of direct HAT photocatalysts, such as N‐centered photocatalysts, have remained underexplored, due to the lower thermodynamic driving force (BDE = 108 kcal mol^−1^ for N–H versus 119 kcal mol^−1^ for O–H)^[^ ^34^ ^]^ and a lack of well‐explored chemotypes with suitable photochemical reactivity. Despite these limitations, recent studies by Yanai, García Mancheño, Ohmatsu, and Ooi on zwitterionic onium amidate photocatalysts^[^ ^35^ ^]^ and by Lambert on the C–H functionalization of ethers in the presence of a trisaminocyclopropenium electrophotocatalyst^[^ ^36^ ^]^ validated the feasibility of this approach. Additionally, flavin derivatives were recently introduced by Storch as direct HAT photocatalysts.^[^ ^37^, ^38^ ^]^ On the other hand, previous studies by our groups^[^ ^39^, ^40^, ^41^, ^42^, ^43^, ^44^, ^45^, ^46^, ^47^ ^]^ as well as others^[^ ^48^, ^49^, ^50^, ^51^, ^52^, ^53^, ^54^, ^55^, ^56^, ^57^, ^58^, ^59^, ^60^, ^61^ ^]^ have demonstrated the efficiency and versatility of visible light photocatalysis of direct decarboxylative functionalization by C9‐aryl‐substituted acridines. Mechanistic studies pointed to the proton‐coupled electron transfer (PCET) in the singlet excited state of the acridine‐carboxylic acid hydrogen bond complex as the primary pathway for direct decarboxylation.^[^ ^39−42^ ^]^

We posited that an appropriately designed photocatalyst based on the acridine framework could enable efficient photocatalysis of direct HAT in the context of photocatalytic C–H functionalization (Figure 1c). Given that acridines cannot form hydrogen bond complexes with nonpolar C─H bonds, direct HAT photocatalysis was expected to be mediated by the longer‐lived triplet excited state of acridine. However, the lower energy of the triplet state and the lack of precomplexation with the substrate could lead to low efficiency of the HAT process and require elevated temperatures to compensate for higher kinetic barriers, which may affect the chemoselectivity and synthetic utility of the method.

Indeed, our preliminary computational studies indicated that the direct HAT process mediated by the most commonly used acridine catalyst **A1** proceeds with a barrier of 24.7 kcal mol^−1^. This result suggested that the reaction efficiency would be limited by the competition of the slow HAT step with unproductive excited state deactivation processes under ambient conditions. Although such challenges are commonly encountered for direct HAT photocatalysts, few strategies are available for enhancing photocatalytic activity. To that end, Hong and Wu reported that the direct HAT photocatalytic activity of eosin Y can be enhanced in the presence of Brønsted acids (Figure 1d).^[^ ^33^ ^]^ Significant enhancement of photocatalytic activity was also achieved for flavin photocatalysts by Storch through a carbonyl group excision‐induced ring contraction (Figure 1e).^[^ ^38^ ^]^

Notably, an inspection of the geometric features of transition state structure **TS1‐*syn*** revealed an obtuse N···H···C9 angle (127.0°), which is consistent with the *π*→π* character of the lowest triplet excited state of the acridine. We therefore postulated that the bent geometry of the HAT transition state could be leveraged for improving the efficiency of direct HAT acridine photocatalysis via stabilizing dispersion interactions with the substrate. While significant progress in the systematic elucidation of key roles of dispersion has been achieved in asymmetric catalytic reactions,^[^ ^62^, ^63^, ^64^, ^65^, ^66^, ^67^, ^68^, ^69^, ^70^ ^]^ strategies for the enhancement of photocatalytic activity by leveraging dispersion interactions as a key reactivity control element in direct HAT photocatalysis have remained unexplored. To that end, we posited that the introduction of an appropriately substituted aryl group in the *ortho*‐position of the C9‐phenyl ring would provide additional stabilization to the transition state through increased dispersion interactions of the catalyst with the substrate, resulting in enhanced photocatalytic activity.

We report herein the development of a nitrogen‐centered photocatalytic system for direct HAT C–H functionalization, which is based on the acridine framework (Figure 1f). The approach leverages dispersion interactions as a guiding design principle for improved photocatalytic activity. Furthermore, the method enabled efficient functionalization of strong C─H bonds in reactions with a wide array of radical coupling partners and under a range of reaction conditions, including cryogenic temperatures. Notably, this work complements the recent studies by Guo and Xia, and Barham on related acridine‐based catalytic systems, which appeared while this work was prepared for publication.^[^ ^71^, ^72^ ^]^ Furthermore, it charts a new path for enhancing direct HAT photocatalytic activity by leveraging photocatalyst‐substrate dispersion interactions in a departure from the previously established Brønsted acid and ring size adjustment approaches.^[^ ^33^, ^38^ ^]^

## Results and discussion

Optimization studies revealed that an efficient light‐mediated C–H functionalization reaction can take place with cyclohexane (**1**) as the substrate and TEMPO (**2**) as the radical trap in the presence of catalytic quantities of acridine photocatalyst **A2** (Table 1, entry 1). The photocatalytic character of the reaction was confirmed in the control experiments conducted without light or the acridine catalyst (entry 2). Acetonitrile proved to be the solvent of choice as lower conversions were observed in other aprotic solvents (entries 3–6).

**Table 1:** Development of acridine d‐HAT photocatalysis. ^a)^

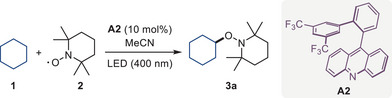		
Entry	Variation from standard conditions	Yield, %^b)^
1	None	99 (94)^c)^
2	No **A2** or light	0
3	In dichloromethane	54
4	In acetone	65
5	In trifluorotoluene	56
6	In fluorobenzene	62
7	Blue LED (*λ*_max_ = 450 nm)	0
8	Bobbitt's salt instead of TEMPO with and without **A2**	0

^a)^ Reaction conditions: cyclohexane (0.5 mmol), 2,2,6,6‐tetramethylpiperidine 1‐oxyl, (TEMPO, **2**) (0.2 mmol), acridine **A2** (10 mol %), acetonitrile (2 mL), rt, LED (*λ*_max_ = 400 nm), 16 h.

^b)^ Yields were determined by ^1^H NMR with 1,3,5‐trimethoxybenzene as an internal standard.

^c)^ Isolated yield. Bobbitt's salt, 4‐(acetylamino)‐2,2,6,6‐tetramethyl‐1‐oxo‐piperidinium tetrafluoroborate.

The use of LED light with longer wavelengths was likewise detrimental to the reaction performance (entry 7). Furthermore, replacing TEMPO with Bobbitt's salt did not produce any product, indicating that the C–H functionalization is not mediated by an oxoammonium intermediate formed by oxidation of TEMPO (entry 8).

Notably, 9‐arylacridines lacking an *ortho*‐aryl substituent in the C9‐aromatic ring and featuring a halogen, methyl, or trifluoromethyl group (**A1** and **A3‐A6**) showed lower catalytic activity. Furthermore, acridine catalyst **A7** lacking the *meta*‐trifluoromethyl substituents in the C9‐biaryl fragment also demonstrated lower catalytic activity. Replacing the two *meta*‐trifluoromethyl substituents with a single *para*‐trifluoromethyl group in photocatalyst **A8** similarly resulted in a lower yield of product **3a**. A decrease in the photocatalytic activity was likewise observed with acridines **A9** and **A10**, featuring other electron‐withdrawing or fluoro substituents and other substitution patterns in the *ortho*‐aryl ring. Notably, lower catalytic activity was also observed when the trifluoromethyl groups were replaced with *tert*‐butyl groups (**A11**). Given the presence of electron rich C─H bonds in **A11**, the lower catalytic performance may be due to degradation of the catalyst under the reaction conditions. Indeed, products of the reaction of **A11** with one and two molecules of TEMPO were observed by high resolution mass spectrometry analysis of the reaction mixture (Figure S2), suggesting that electron rich substituents prone to hydrogen atom abstraction are less suitable than the optimal trifluoromethyl groups. In contrast, acridine **A12** featuring phenyl groups in the *meta* positions demonstrated improved catalytic performance over **A1**, although the reaction was less efficient than with catalyst **A2**.

**Figure 2:**
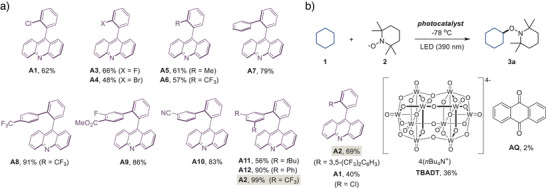
Photocatalytic activity of acridines in the C–H functionalization reaction of cyclohexane **1** with TEMPO (**2**). a) Substituent effects on the HAT photocatalytic activity of acridines. See Table 1 for reaction conditions. b) Benchmarking of the photocatalytic activity of acridine **A2** and other direct HAT photocatalysts under cryogenic conditions. Yields of product **3a** were determined by ^1^H NMR with 1,3,5‐trimethoxybenzene as an internal standard.

Photocatalysis under cryogenic conditions can provide opportunities for the development of new and selective reactions.^[^ ^73^, ^74^, ^75^, ^76^ ^]^ However, the development of direct HAT photocatalysts that can efficiently operate at cryogenic temperatures remains challenging. To that end, the performance of acridine **A2** was benchmarked against the commonly used direct HAT photocatalysts TBADT and aromatic ketone **AQ** (Figure 2b). Notably, product **3a** was produced in 69% yield with acridine **A2** at –78 °C, while significantly lower yields (36% and 2%) were observed for TBADT and ketone **AQ**, suggesting that the acridine photocatalyst can be used when previously established direct HAT photocatalysts do not afford sufficient photocatalytic activity. Significantly, catalyst **A1** afforded product **3a** in 40% yield, indicating that the beneficial effect of the *ortho*‐aryl substituent is retained under the cryogenic conditions.

**Figure 3:**
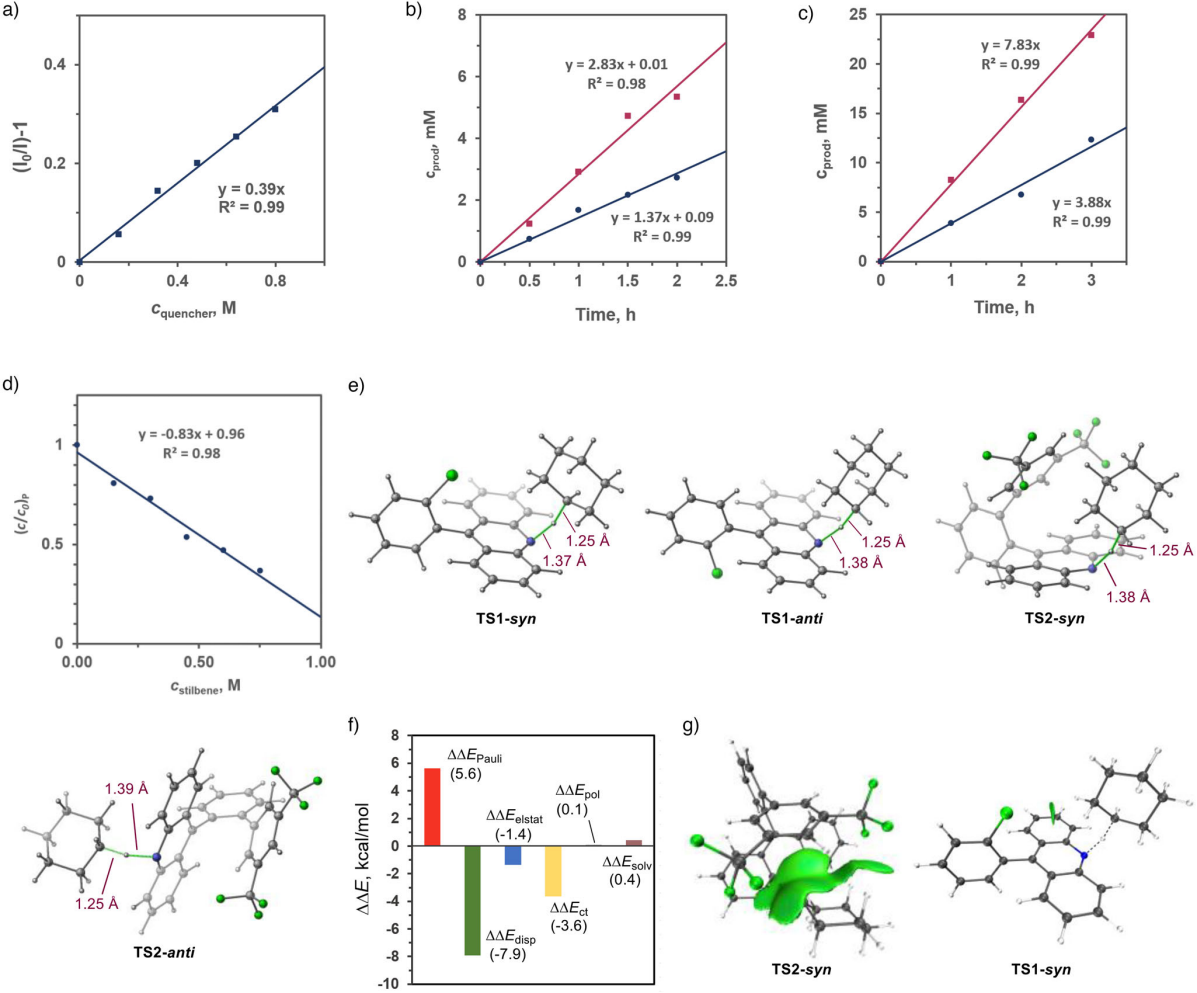
Mechanistic studies of the acridine‐catalyzed direct HAT C–H functionalization. a) Stern–Volmer luminescence quenching study. b) Kinetic profile of the C–H functionalization of cyclohexane with TEMPO catalyzed by acridines **A1** (▬●▬) and **A2** (▬■▬). c) Kinetic isotope effect study for the **A2**‐catalyzed C–H functionalization of cyclohexane (▬■▬) and *d*_12_‐dyclohexane (▬●▬) with 2‐(4‐fluorobenzylidene)malononitrile. d) Dependence of the **A2**‐catalyzed C–H functionalization efficiency (ratio of the product concentrations in the presence (*c*) and in the absence (*c*_0_) of the quencher) on the initial concentration of trans‐stilbene. e) Transition state structures for the **A1**‐ and **A2**‐mediated HAT processes. f) Energy decomposition analysis of transition state structures **TS2‐*syn*** and **TS2‐*anti***, ΔΔ*E^‡^* = Δ*E^‡^* **_TS2‐_ *_syn_*** – Δ*E^‡^* **_TS2‐_ *_anti_***, kcal mol^−1^. g) IGMH analysis of **TS2‐*syn*** and **TS1‐*syn***.

We next sought to elucidate the mechanistic underpinnings of direct HAT photocatalysis by acridines. Stern–Volmer plot studies showed that the acridine luminescence can be quenched by a C–H substrate (Figure 3a). These results indicate that the acridine excited state is involved in the direct HAT process.

Furthermore, kinetic profiles of the C–H functionalization reactions with acridines **A1** and **A2** revealed a substantially higher reaction rate for **A2** (Figure 3b). These results are consistent with the higher yield of product **3a** observed for **A1** and **A2** in the optimization studies (Figure 2), and they suggest that the HAT process is more kinetically facile with catalyst **A2**. Additionally, kinetic isotope effect studies yielded a *k*_H_/*k*_D_ ratio of 1.9, suggesting that the photoinduced HAT by acridine is a rate‐limiting step (Figure 3c).

To gain insight into the nature of the acridine excited state, which mediates the HAT process, a triplet quenching study was carried out with *trans*‐stilbene as a triplet quencher (Figure 3d). A concentration‐dependent decrease in the direct HAT photocatalytic activity of acridine **A2** was observed in the presence of *trans*‐stilbene, which was accompanied by the isomerization to *cis*‐stilbene (*E*/*Z* ratio = 2: 1). These observations are consistent with the direct HAT process mediated by the triplet excited state of acridine.

To gain further insight into the catalyst‐substrate interactions underlying the improved photocatalytic activity of C9‐biaryl‐substituted acridine **A2**, a computational analysis of the photoinduced HAT process was performed for acridines **A2** and **A1**. Electron‐hole analysis of the three lowest singlet and triplet excited states for acridines **A1** and **A2** revealed that the different *ortho*‐substituents in the C9‐aryl group do not have any significant effect on the energies and orbital characteristics of the excited states, reflected by substantially similar electron/hole distribution and separation parameters (Figures S4–S7 and Tables S3–S6). No significant differences were also observed in the spin density distribution for triplet **A1** and **A2** and acridinyl radicals **HA1** and **HA2**. Taken together, these results indicate that the structural changes in the C9‐aryl *ortho*‐substituent do not have a significant effect on the nature and properties of the acridine excited states and acridinyl intermediates, which can be involved in the photocatalytic reaction. Transition state structures for the HAT process were examined next (Figure 3e). In line with the preliminary studies, the HAT transition state structures adopted bent geometries, which correspond to the approach of the C–H substrate either at the side of the *ortho*‐substituent in the acridine catalyst (**TS1‐*syn*** and **TS2‐*syn***) or at the unobstructed side (**TS1‐*anti*** and **TS2‐*anti***). Notably, a substantially lower activation energy was observed for *syn*‐transition state structure **TS2‐*syn*** (Δ*G^‡^* = 21.7 kcal mol^−1^), than for **TS2‐*anti*** (Δ*G^‡^* = 25.4 kcal mol^−1^) with **A2** as a catalyst. In contrast, no significant difference in the energies of the *syn*‐ and *anti*‐transition state structures (**TS1‐*syn*** and **TS1‐*anti***, Δ*G^‡^* = 24.7 and 24.5 kcal/mol) was revealed for catalyst **A1**, which has an *ortho*‐chloro substituent. Importantly, the barrier for the HAT process, which traverses the *syn*‐transition state structure **TS2‐*syn*** is also significantly lower than both the *syn*‐ and *anti*‐pathways mediated by catalyst **A1**. These results suggest that the direct HAT process catalyzed by **A2** benefits from stabilizing noncovalent interactions (NCIs) imparted by the C9‐*ortho*‐biaryl substituent.

Energy decomposition analysis (EDA)^[^ ^77^ ^]^ was performed next to elucidate the nature of the NCIs, which stabilize the *syn*‐transition state structure (Figure 3f). The studies revealed that **TS2‐*syn*** suffered from higher Pauli (steric) repulsion than **TS2‐*anti***, which is consistent with the proximity of the C–H substrate to the C9‐*ortho*‐biaryl substituent. However, the repulsive interactions were counterbalanced by substantially stronger attractive interactions, with dispersion proving the major contribution to the stabilization of **TS2‐*syn***.

Additionally, **TS2‐*syn*** also benefited from stronger charge transfer (orbital) *n*_(CF3)_→σ*(C–H) interactions between the substrate and the catalyst, which were revealed by complementary occupied‐virtual orbitals pairs (COVP) analysis (Figures S11–S13). Further analysis via the independent gradient model based on the Hirshfeld partition (IGMH, Figure 3g)^[^ ^78^ ^]^ indicated that the dispersion stabilization was due to the interactions between the substrate and both the *ortho*‐phenyl ring in the biaryl fragment and the trifluoromethyl substituents. By contrast, the dispersion interaction was substantially smaller between the chloro substituent and the C–H substrate in **TS1‐*syn***, underscoring the stabilizing role of the *ortho*‐aryl substituent.

**Figure 4:**
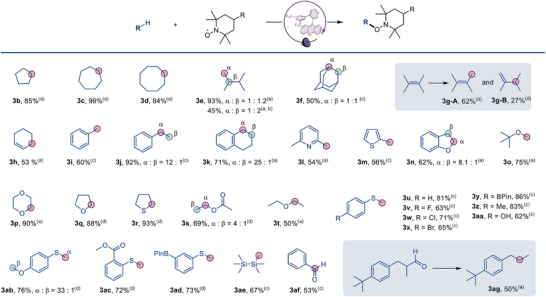
Scope of C–H substrates in the acridine catalyzed C–H aminoxylation reactions. See Table 1 and the SI for the experimental details; isolated yields. ^a)^ 4‐Hydroxy‐TEMPO was used. ^b)^ The reaction was carried out at –78 °C. ^c)^ TEMPO was used. ^d)^ 4‐Oxo‐TEMPO was used. Red and green circles denote the position of the alkoxyamine substituent in the product.

With the optimal conditions established, we investigated the scope of C(*sp* ^3^)─H bond‐containing hydrocarbons (Figure 4). Cycloalkanes such as cyclopentane (**3b**), cycloheptane (**3c**), and cyclooctane (**3d**) yielded corresponding C(*sp* ^3^)−H aminoxylation products in 84%–99% yield. Notably, linear alkanes (**3e**) and adamantane (**3f**) exhibited preferential activation of primary and secondary C─H bonds over tertiary C─H bonds, contrary to conventional selectivity trends. This inversion likely stems from steric constraints imposed by the acridine catalyst. Allylic substrates (**3g**–**3h**) were efficiently functionalized, while (hetero)aromatic substrates (**3i**–**3m**) with benzylic C─H bonds demonstrated selective reactivity. The protocol further achieved α‐oxy C(*sp* ^3^)−H aminoxylation in cyclic/acyclic ethers (**3n**–**3q**, **3t**) and esters (**3s**). Analogously, tetrahydrothiophene (**3r**) underwent exclusive α‐sulfur functionalization. Aryl thioethers (**3u**–**3ad**) proved highly compatible, including those bearing halides (**3v**–**3x**), alkyl (**3z**), hydroxy (**3aa**), methoxy (**3ab**), esters (**3ac**), and boronic esters (**3y**, **3ad**). Organosilanes (**3ae**) and aliphatic/aromatic aldehydes (**3af**, **3ag**) also participated effectively. Additionally, lily aldehyde underwent decarbonylative oxidation under standard conditions (**3ag**).

To investigate the effect of cryogenic conditions on the HAT regioselectivity, the reactions were conducted with substrate **3e**. Improved selectivity was observed (*α*: *β* = 1: 2, Figure 4), indicating that the cryogenic catalytic system can be used to modulate the selectivity of the C–H functionalization.

**Figure 5:**
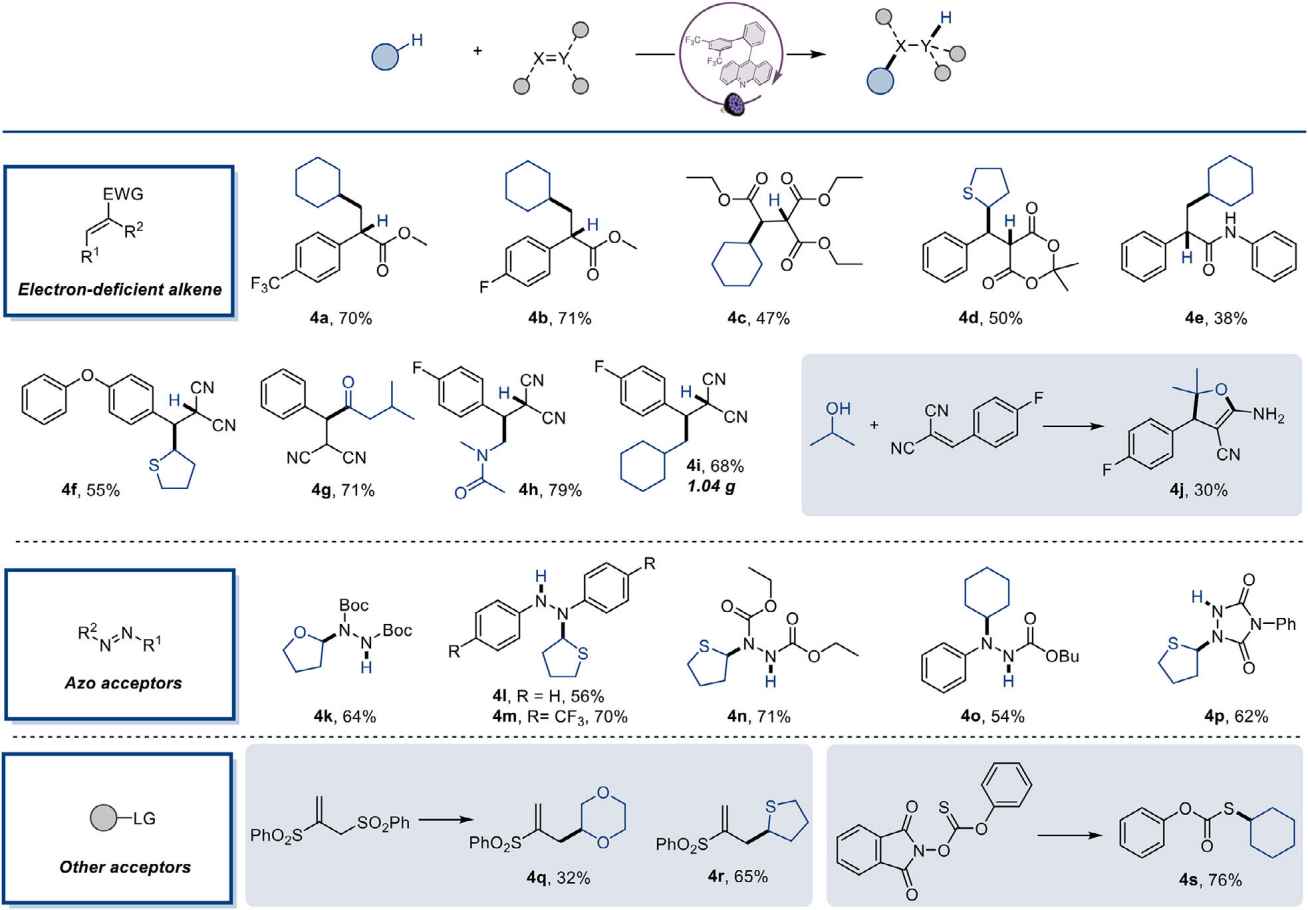
Scope of radical acceptors in the acridine‐catalyzed C–H functionalization reactions. See Table 1 and Supporting Information for the experimental details, isolated yields.

The scope of acridine‐mediated C(*sp* ^3^)−H bond activation was subsequently expanded to radical addition reactions with diverse acceptors (Figure 5). Electron‐deficient alkenes, particularly α‐arylacrylates (**4a**, **4b**), served as effective electrophiles in the C–H alkylation protocol. Similarly, various Michael acceptors (**4c**–**4e**) demonstrated excellent compatibility under mild conditions. Arylidenemalononitriles (**4f**–**4i**) exhibited moderate to good reactivity. Moreover, the successful gram‐scale synthesis of **4i** highlights the potential of this methodology for practical applications. Notably, employing isopropanol as the C − H substrate afforded a tandem radical addition/cyclization product (**4j**). As anticipated, diverse azo‐type acceptors coupled efficiently with radical precursors to furnish C–N coupling products in moderate to good yields (**4k**–**4p**). The protocol also tolerated allyl sulfones (**4q**, **4r**) and thionocarbonate (**4s**).

**Figure 6:**
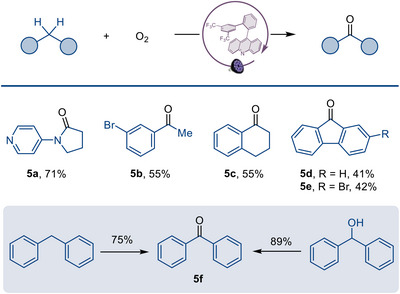
Acridine‐catalyzed direct aerobic oxidation of aliphatic C─H bonds. Reaction conditions: C–H substrate (0.5 mmol), acridine **A2** (4 mol %), acetonitrile (0.5 mL), oxygen (1 atm), LED (*λ*_max_ = 400 nm), rt, 12 h; isolated yields.

Direct aerobic oxidation of aliphatic C─H bonds is a potentially attractive and sustainable synthetic strategy as it utilizes molecular oxygen as the terminal oxidant while minimizing waste generation. Pleasingly, our catalytic system demonstrated excellent selectivity for both cyclic and acyclic substrates (Figure 6). For instance, 4‐pyrrolidinylpyridine was smoothly oxidized to corresponding amide **5a**, while alkylarenes (**5b**–**5f**) underwent selective benzylic oxidation to furnish acetophenones and biaryl ketones in good yields.

**Figure 7:**
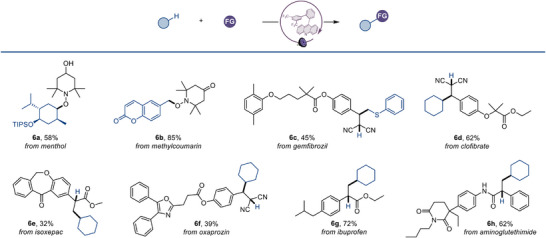
Acridine‐catalyzed C–H‐functionalization of medicinal compounds. See Table 1 and the Supporting Information for the experimental details; isolated yields.

To further evaluate the scope of the catalytic system, we examined substrates with increased structural complexity (Figure 7). Notably, protected menthol derivatives and coumarin scaffolds (**6a**, **6b**) were selectively functionalized at the most sterically accessible secondary C─H bonds and benzylic positions with excellent regiocontrol. The methodology proved particularly valuable for late‐stage functionalization of pharmaceutical compounds, including gemfibrozil (**6c**), clofibrate (**6d**), isoxepac (**6e**), oxaprozin (**6f**), ibuprofen (**6g**), and aminoglutethimide (**6h**). These medicinally relevant substrates were smoothly converted to their corresponding functionalized products, highlighting the performance of the photocatalytic system in complex structural settings.

## Conclusion

In summary, we have developed a nitrogen‐centered photocatalytic system for radical C–H functionalization via direct hydrogen atom transfer (HAT), based on the acridine framework. This photocatalytic system enables efficient formation of carbon–carbon and carbon–heteroatom bonds, including C–H functionalization under challenging cryogenic conditions. The photocatalytic activity of acridines is enhanced by stabilizing dispersion interactions involving the C9‐*ortho*‐biaryl substituent, providing a blueprint for improving the efficiency of direct HAT photocatalysis through substrate‐photocatalyst dispersion interactions.

## Conflict of Interests

The authors declare no conflict of interest.

## Supporting information



Supporting Information

## Data Availability

The data supporting the findings of this study are available in the Supporting Information for this article.
