# Supplementary material for: Dispersion‐Enhanced Nitrogen‐Centered Photocatalysis of the Direct Hydrogen Atom Transfer
Source: Angew Chem Int Ed Engl. 2025 Dec 18;65(6):e22022. doi: 10.1002/anie.202522022 (PMC12865266; doi:10.1002/anie.202522022)
Supplement: Supplementary file 1 — Supporting Information [file ANIE-65-e22022-s001.pdf]

Supporting Information for

Dispersion-Enhanced Nitrogen-Centered Photocatalysis of the Direct Hydrogen Atom Transfer

Jiasong Zhang[a¶], Kaitong Zhuang[a¶], Ramon Trevino[b¶], Babu Raj Dhungana[b¶], Huiying Sun[a], Shuyu Yin[a], Yuting Li[a], Jacob A. Sanchez[b], Xiaoyu Xia[a], Ramy Elerian[b], Yao Sun[a], Seth O. Fremin[b], Chao Huang[a], Min He[a], Maosheng Cheng*[c], Oleg V. Larionov*[b], Shengfei Jin*[a]

[a] Wuya College of Innovation, Shenyang Pharmaceutical University, Shenyang 110016, P. R. China
[b] Department of Chemistry, The University of Texas at San Antonio, One UTSA Circle, San Antonio, TX, 78249, USA
[c] Key Laboratory of Structure-Based Drug Design and Discovery, Ministry of Education, School of Pharmaceutical Engineering, Shenyang Pharmaceutical University, Shenyang 110016, P. R. China
oleg.larionov@utsa.edu, mscheng@syphu.edu.cn, jinshengfei@syphu.edu.cn

Table of contents

Materials and experimental details S1
General procedures S2
Mechanistic studies S3
Synthesis of the photocatalysts and substrates S5
Product characterization S12
Computational studies S36
NMR spectroscopic data S57
References S237

Materials and experimental details

Materials: Acetonitrile were thoroughly degassed under the atmosphere of argon and dried with 3 Å molecular sieves before use. Free radical acceptor **S1**,[1] **S2**,[1] **S3**,[1] **S4**,[2] **S5**,[1] **S6**,[3] **S7**,[3] **S8**,[3] **S9**,[4] **S10**,[5] **S11**,[6] **S12**,[7] **S13**,[3] **S14**,[3] **S15**,[1] **S16**,[3] **S17**,[1] **S18**,[1] and acridine photocatalysts **A1**[8] and **A5**[8] were prepared according to the literature procedures. All other chemicals were used as commercially available.

Experimental equipment: An 8 mL reaction tube was used for a 0.1 mmol scale reaction. The reaction tube was arranged to maintain a 2–3 cm distance from a 36 W LED light (λ_{max} = 400 nm, 2.3 mW/cm^2) while ensuring efficient stirring. Experiments at –78 °C were carried out in an EYELA PSL-1810 cryostat in an ethanol bath, using Heyseek HSL40-390 LED light (λ_{max} = 390 nm, 10.5 mW/cm^2) positioned 8 cm from the reaction vessel.

Purification: Flash column chromatography was performed on silica gel (particle size 200–300 mesh, purchased from Shanxi, China). Thin layer chromatography was carried out on silica gel-coated glass plates (PuKe GF254). Plates were visualized under ultraviolet light (254 nm) and using a potassium permanganate stain.

Characterization: ^{1}H, ^{13}C and ^{19}F NMR spectra were recorded at 400 MHz (^{1}H), 100 MHz (^{13}C) and 375 MHz (^{19}F) on

¶ These authors contributed equally

Bruker Ascend 400 instruments and in $CDCl_3$ or other specified deuterated solvents with and without tetramethylsilane (TMS) as an internal standard at 25 °C, unless specified otherwise. ^{1}H and ^{13}C NMR spectra were recorded at 600 MHz (^{1}H) and 150 MHz (^{13}C) on Bruker AVANCE 600 instruments and in $CDCl_3$ or other specified deuterated solvents with and without tetramethylsilane (TMS) as an internal standard at 25 °C, unless specified otherwise. Chemical shifts (δ) are reported in parts per million (ppm) from tetramethylsilane (^{1}H and ^{13}C), CF_3COOH (^{19}F). Coupling constants (*J*) are in Hz. Proton multiplicity is assigned using the following abbreviations: singlet (s), doublet (d), triplet (t), quartet (q), quintet (quint.), septet (sept.), multiplet (m), broad (br).

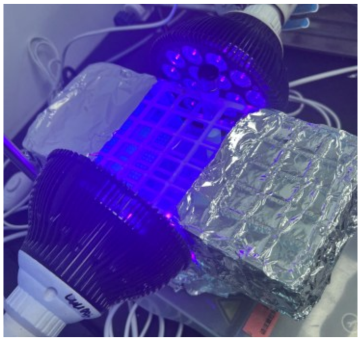

Room temperature reaction setup

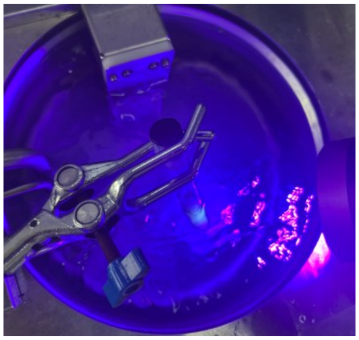

Low temperature reaction setup

High-resolution mass spectra (HRMS) were recorded on a Bruker SolariX 7.0T instrument. Infrared measurements were carried out on a Bruker IFS 55 spectrometer. UV–Vis absorption spectra were recorded on a Shimadzu UV-2600 instrument in quartz cuvettes. Samples for the UV–Vis measurements were prepared in anhydrous degassed acetonitrile.

General procedures

General procedure for the acridine-catalyzed direct HAT reactions with TEMPO (GP1)

An oven-dried 8 mL reaction tube fitted with a PTFE-coated stir bar was charged with TEMPO or its derivatives (0.2 mmol) and acridine **A2** (10 mol%，0.01 mmol). The reaction tube was sealed with a screw-cap containing a PTFE-lined silicone septum. The reaction tube was degassed with argon by three evacuation-backfill cycles. Then acetonitrile (2 mL) was added, followed by C–H substrates (0.4–1 mmol). The tube was capped with a screw cap, wrapped with parafilm and irradiated with LED light (λ_{max} = 400 nm) at room temperature for 16–24 hours. The reaction mixture was then concentrated under reduced pressure and purified by flash chromatography on silica gel with a mixture of petroleum ether and ethyl acetate as an eluent to give the corresponding product.

General procedure for the acridine-catalyzed direct HAT reactions with other radical acceptors (GP2)

An oven-dried 8 mL reaction tube fitted with a PTFE-coated stir bar was charged with radical acceptor (0.2 mmol) and acridine **A2** (10 mol%，0.02 mmol). The reaction tube was sealed with a screw-cap containing a PTFE-lined silicone septum. The reaction tube was degassed with argon by three evacuation-backfill cycles. Then acetonitrile (2 ml) was added, followed by C–H substrate (1–2 mmol). The tube was capped with a screw cap, wrapped with parafilm and irradiated with LED light (λ_{max} = 400 nm) at room temperature for 24 hours. The reaction mixture was then concentrated under reduced pressure and purified by flash chromatography on silica gel with a mixture of petroleum ether and ethyl acetate as an eluent to give the corresponding product.

General procedure for the acridine-catalyzed C–H oxidation reaction (GP3)

An oven-dried 8 mL reaction tube fitted with a PTFE-coated stir bar was charged with C–H substrates (0.5 mmol) and acridine **A2** (4 mol%, 0.02 mmol). The reaction tube was sealed with a screw-cap containing a PTFE-lined silicone septum. The reaction tube was degassed with oxygen by three evacuation-backfill cycles. Then acetonitrile (0.5 mL) was added. The tube wrapped with parafilm, attached with an oxygen-filled balloon via needle and irradiated with LED light (λ_{max} = 400 nm) at room temperature for 24 hours. The reaction mixture was then concentrated under reduced pressure and purified by flash chromatography on silica gel with a mixture of petroleum ether and ethyl

acetate as an eluent to give the corresponding product.

Mechanistic studies

Stern-Volmer quenching studies

The Stern-Volmer quenching experiments were carried out with 0.02mM solutions of acridine photocatalysts in anhydrous and degassed acetonitrile with a quencher (methyl *tert*-butyl ether) that is present in varied concentrations were irradiated at 360 nm, and the emission was measured (Figure S1).

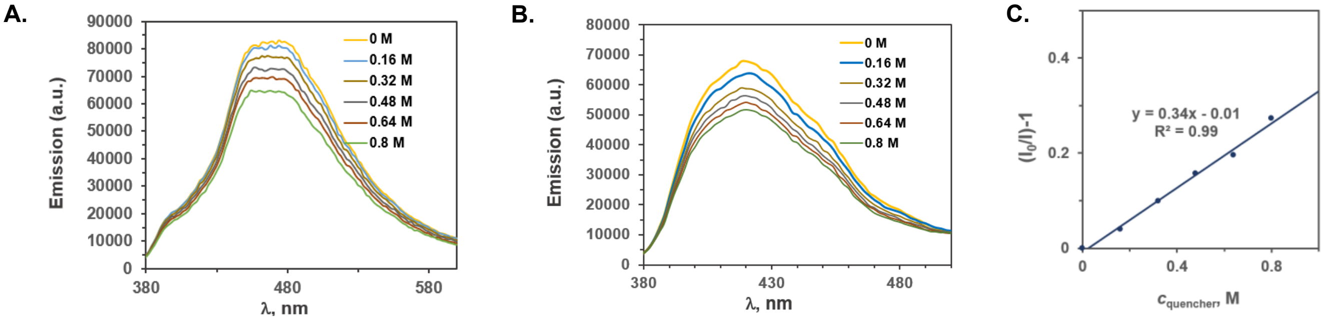

Figure S1. Emission spectra of acridine **A1** (**A**) and **A2** (**B**) (*c* = 0.02 mM in acetonitrile) in the presence of varied concentrations of methyl *tert*-butyl ether and a Stern–Volmer luminescence quenching plot for acridine **A1**.

Stern–Volmer luminescence quenching studies show that acridines **A1** and **A2** have similar quenching rates (k_Q = 0.39 for **A2** and 0.34 for **A1**). This is consistent with the computational studies of the lifetimes and spin–orbit coupling constants (page S44) and hole–electron analysis of the excited states (Tables S3–S7), which indicate that the 9-aryl substituent does not have a significant effect on the photophysical properties of the acridine catalysts. Because most collisions with the quencher result in unproductive excited state quenching, due to the activation barrier for the HAT process, only a small difference in the quenching rates is expected for the catalysts, as observed experimentally.

Triplet quenching study of the HAT reaction

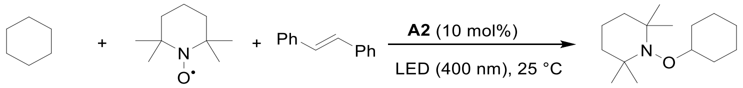

Radical trapping studies were carried out with cyclohexane (42 mg, 0.5 mmol), acridine **A2** (4.7 mg, 0.01 mmol, 10 mol%) and acetonitrile (2 mL) with TEMPO (31.2 mg, 0.2 mmol) in presence of varied quantities of *trans*-stilbene (0, 0.3, 0.6, 0.9, 1.2, 1.5 mmol). The reaction mixture was irradiated with LED light (λ = 400 nm) while stirring at rt for 4 h. Yields and ratios of *trans*-stilbene to *cis*-stilbene were determined by ^{1}H NMR with 1,3,5-trimethoxybenezene as an internal standard.

KIE study of the acridine-catalyzed HAT reaction

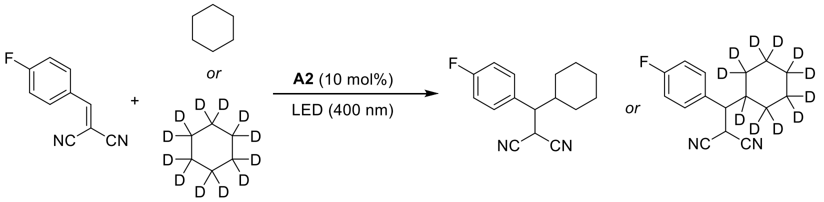

The study was carried out using 2-(4-fluorobenzylidene)malononitrile (17.2 mg, 0.1 mmol), acridine **A2** (4.7 mg, 0.01 mmol, 10 mol%), cyclohexane (42 mg, 0.5 mmol) or *d*12- cyclohexane (48 mg, 0.5 mmol), and acetonitrile (2 mL) according to GP1. The reaction mixture was irradiated with LED light (λ = 400 nm) while stirring at rt. Aliquots of the reaction mixture were taken up at 1, 2, and 3 h, and the product yields were determined by ^{1}H NMR with 1,3,5-

trimethoxybenezene as an internal standard.

Low temperature experiments with different photocatalysts

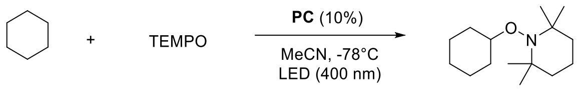

The study was carried out using TEMPO (31.3 mg, 0.2 mmol), cyclohexane (0.12 ml, 1.0 mmol) and acetonitrile (2 mL) and acridine **A2** (4.7 mg, 10 mol%, 0.01 mmol). The tube was capped with a screw cap and wrapped with parafilm then irradiated with LED light (λ_{max} = 400 nm) at –78 °C for 36 hours. Reaction yields were determined by ^{1}H NMR with 1,3,5-trimethoxybenezene as an internal standard. Two parallel experiments were conducted with TBADT (25.9 mg, 10 mol%, 0.01 mmol), anthraquinone (2.1 mg, 10 mol%, 0.01 mmol), and **A1** (2.9 mg, 10 mol%, 0.01 mmol) as the photocatalysts.

Study of the regioselectivity of the HAT reaction at low temperature

The study was carried out using TEMPO (31.3 mg, 0.2 mmol), C–H substrate **3e** (1 mmol) and acetonitrile (2 mL) and acridine **A2** (4.7 mg, 10 mol%, 0.01 mmol). The tube was capped with a screw cap and wrapped with parafilm then irradiated with LED light (λ_{max} = 400 nm) at –78 °C for 36 hours. The reaction yield (45%) and regioselectivity (α/β ratio 1 : 2) were determined by ^{1}H NMR with 1,3,5-trimethoxybenezene as an internal standard.

Kinetic studies of HAT reactions with A1 or A2 as photocatalyst

General procedure GP1 was followed with **A1** as the photocatalyst, aliquots of the reaction mixture were taken up at 0.5, 1, 1.5 and 2 hours. A parallel reaction was conducted with **A2** as the photocatalyst, and aliquots of the reaction mixture were taken up at 0.5, 1, 1.5 and 2 hours. The product yields were determined by ^{1}H NMR with 1,3,5-trimethoxybenezene as an internal standard.

Mass spectrometric detection of the acridine A11-TEMPO adducts

The general procedure **GP1** was followed with TEMPO (31.3 mg, 0.2 mmol), acridine **A11** (4.4 mg, 10 mol%, 0.01 mmol), cyclohexane (0.06 ml, 0.5 mmol) and acetonitrile (2 mL). The tube was capped with a screw cap and wrapped with parafilm then irradiated with LED light (λ_{max} = 400 nm) at room temperature for 24 hours and then reaction mixture was subjected to mass spectrometric analysis. Mono and di-TEMPO-acridine adducts were detected by high resolution mass spectrometry (Figure S2).

A.

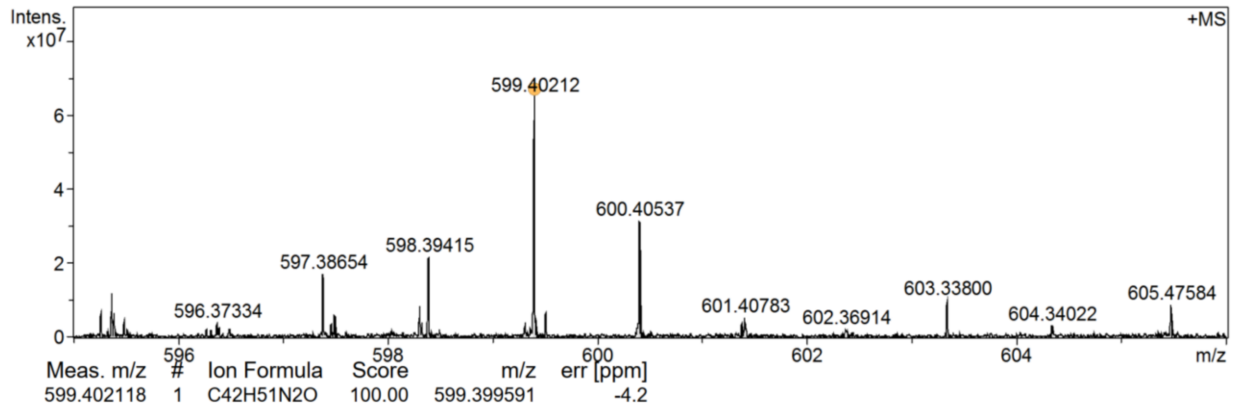

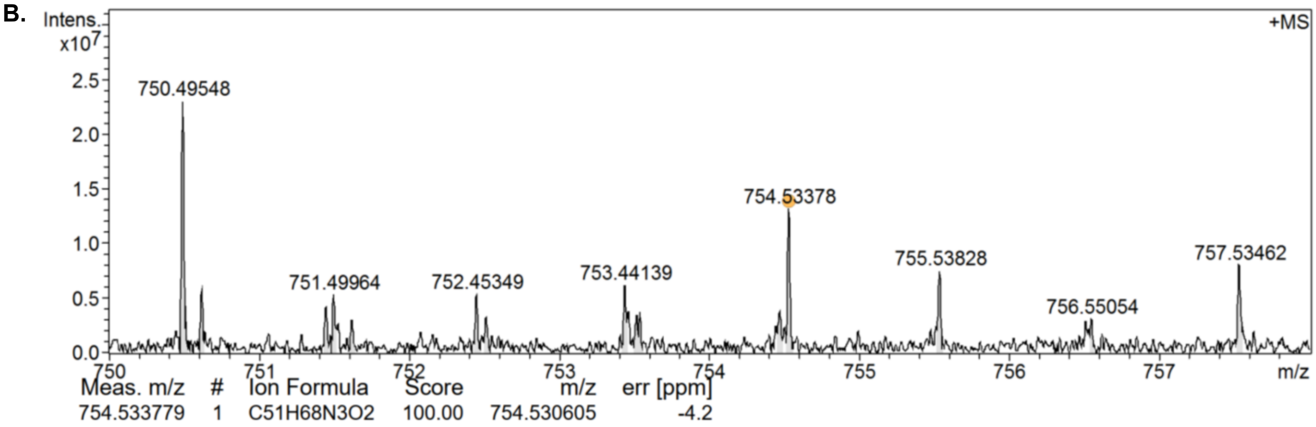

Figure S2. Mass spectrometric detection of the acridine **A11**–TEMPO adducts with one (**A**) and two (**B**) molecules of TEMPO produced in the photocatalytic C–H functionalization of cyclohexane with TEMPO.

Synthesis of the photocatalysts and substrates

9-(3',5'-Bis(trifluoromethyl)-[1,1'-biphenyl]-2-yl)acridine (A2)

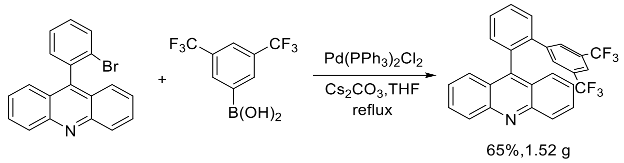

A sealed tube was charged with 9-(2-bromophenyl)acridine (**A4**) (1.67 g, 5 mmol), bis(triphenylphosphine)-palladium(II) chloride (0.175 g, 0.25 mmol), 3,5-bis(trifluoromethyl)benzeneboronic acid (1.42 g, 5.5 mmol), cesium carbonate (1.79 g, 5.5 mmol) and THF (50 mL) under an atmosphere of argon. The mixture was heated to reflux for 24 hours. The reaction was monitored by thin layer chromatography. When the reaction was completed, the solid was filtered and washed with dichloromethane. The organic layer was concentrated under reduced pressure. The crude product was purified by flash chromatography on silica gel with EtOAc/hexane, 1 : 50 v/v as an eluent to give corresponding product (1.52 g, 65%) as a yellow solid.

^{1}H NMR (400 MHz, $CDCl_3$): δ 8.22 (d, *J* = 8.8 Hz, 2H), 7.76 – 7.62 (m, 5H), 7.58 (d, *J* = 8.7 Hz, 2H), 7.51 (dd, *J* = 7.3, 1.5 Hz, 1H), 7.43 (dd, *J* = 8.7, 6.5 Hz, 2H), 7.36 (s, 3H) ppm. – ^{13}C NMR (100 MHz, $CDCl_3$): δ 148.3, 142.3, 139.8, 134.9, 131.5, 131.4, 131.0, 130.7, 130.4, 130.3, 129.9, 129.8, 129.5, 128.9, 128.6, 128.5, 126.4, 126.1, 125.4, 124.2, 121.5, 120.8, 120.8, 120.7, 120.7, 120.6, 118.8 ppm. – ^{19}F NMR (376 MHz, $CDCl_3$) δ -63.5 ppm. – HRMS: calcd for $C_{27}H_{16}F_6N$ 468.1109, found 468.1185 [M+H$^+$].

9-(2-Fluorophenyl)acridine (A3)

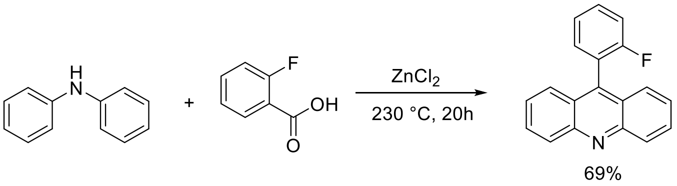

A sealed tube was charged with diphenylamine (0.8461 g, 5 mmol), 2-fluorobenzoic acid (2.1017 g, 15 mmol) and zinc chloride (3.4075g, 25 mmol). The mixture was slowly heated to 230 ℃ with sand bath for 20 hours. When the reaction was completed, ethyl acetate (50 mL) was added. The diluted mixture was washed with ammonia and brine. The organic layer was dried with sodium sulfate and concentrated under reduced pressure. The crude product was purified by pulping with dichloromethane and *n*-hexane and obtained the product 9-(2-fluorophenyl)acridine (0.94

g, 69%) as a yellow solid.

^{1}H NMR (400 MHz, $CDCl_3$): δ 8.31 (d, J = 8.8 Hz, 2H), 7.78 (m, 2H), 7.68 – 7.63 (m, 2H), 7.61 – 7.55 (m, 1H), 7.45 (m, 2H), 7.40 – 7.31 (m, 3H) ppm. – ^{13}C NMR (100 MHz, $CDCl_3$): δ 161.5, 159.0, 148.9, 140.9, 132.6, 132.6, 130.9, 130.9, 130.1, 129.9, 126.4, 126.1, 125.4, 124.4, 124.4, 123.6, 123.4, 116.3, 116.1 ppm. – ^{19}F NMR (376 MHz, $CDCl_3$) δ -112.8 ppm. – HRMS: calcd for $C_{19}H_{13}FN$ 274.1027, found 274.1016 [M+H$^+$].

9-(2-Bromophenyl)acridine (A4)[8]

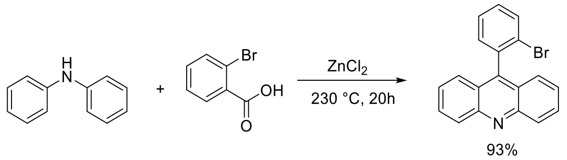

A sealed tube was charged with diphenylamine (1.69 g, 10 mmol), 2-bromobenzoic acid (6.03 g, 30 mmol) and zinc chloride (7.09 g, 52 mmol). The mixture was slowly heated to 230 °C with sand bath for 20 hours. When the reaction was completed, ethyl acetate (50 mL) was added. The diluted mixture was washed with ammonia and brine. The organic layer was dried with sodium sulfate and concentrated under reduced pressure. The crude product was purified by pulping with dichloromethane and *n*-hexane and obtained the product 9-(2-bromophenyl)acridine (**A4**) (3.11 g, 93%) as a yellow solid.

9-(2-(Trifluoromethyl)phenyl)acridine (A6)

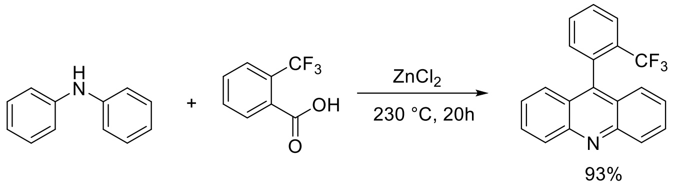

A sealed tube was charged with diphenylamine (0.8461 g, 5 mmol), 2-(trifluoromethyl)benzoic acid (2.8518 g, 15 mmol) and zinc chloride (3.4075g, 25 mmol). The mixture was slowly heated to 230 ℃ with sand bath for 20 hours. When the reaction was completed, ethyl acetate (50 mL) was added. The diluted mixture was washed with ammonia and brine. The organic layer was dried with sodium sulfate and concentrated under reduced pressure. The crude product was purified by pulping with dichloromethane and n-hexane and obtained the product 9-(2-(trifluoromethyl)phenyl)acridine (97 mg, 6%) as a yellow solid.

^{1}H NMR (400 MHz, CDCl3): δ 8.29 (d, *J* = 8.8 Hz, 2H), 7.96 (dd, *J* = 7.0, 2.3 Hz, 1H), 7.74 (m, 4H), 7.45 – 7.32 (m, 5H) ppm. – ^{13}C NMR (100 MHz, CDCl3): δ 148.7, 144.1, 135.0, 135.0, 134.5, 132.3, 131.9, 130.4, 130.1, 130.1, 129.8, 129.7, 129.2, 129.0, 126.7, 126.7, 126.6, 126.6, 125.9, 125.6, 125.1, 123.9, 122.4 ppm. – ^{19}F NMR (376 MHz, CDCl3) δ -59.7 ppm. – HRMS: calcd for $C_{20}H_{13}F_3N$ 324.0995, found 324.0984 [M+H$^+$]

9-([1,1'-Biphenyl]-2-yl)acridine (A7)

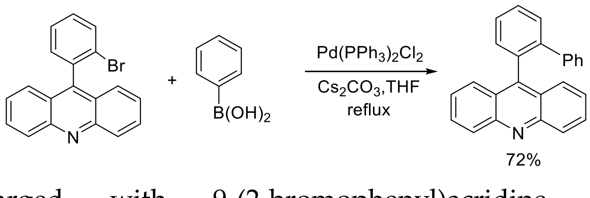

A sealed tube was charged with 9-(2-bromophenyl)acridine (**A4**) (1.67 g, 5 mmol), bis(triphenylphosphine)palladium(II) chloride (175.4 mg, 0.25 mmol), phenylboronic acid (0.67 g, 5.5 mmol), cesium carbonate (1.79 g, 5.5 mmol) and THF (50 mL) under an atmosphere of argon. The mixture was heated to reflux for 24 hours. The reaction was monitored by thin layer chromatography. When the reaction was completed, the solid was filtered and washed with dichloromethane. The organic layer was concentrated under reduced pressure. The

Go to table of contents

crude product was purified by flash chromatography on silica gel with EtOAc/hexane, 1 : 50 v/v as an eluent to give corresponding product (1.19 g, 72%) as a yellow solid.

^{1}H NMR (400 MHz, $CDCl_3$): δ 8.23 – 8.17 (m, 2H), 7.72 – 7.61 (m, 6H), 7.55 (ddd, *J* = 7.6, 5.9, 2.9 Hz, 1H), 7.36 (ddd, *J* = 8.3, 6.7, 1.2 Hz, 3H), 6.98 – 6.91 (m, 2H), 6.91 – 6.80 (m, 3H) ppm. – ^{13}C NMR (100 MHz, $CDCl_3$): δ 148.3, 146.7, 142.4, 140.2, 134.1, 131.3, 130.1, 129.6, 129.3, 128.7, 128.1, 127.4, 127.0, 126.7, 126.6, 125.4, 125.2 ppm.– HRMS: calcd for $C_{25}H_{18}N$ 332.1361, found 332.1433 [M+H$^+$].

9-(4'-(Trifluoromethyl)-[1,1'-biphenyl]-2-yl)acridine (A8)

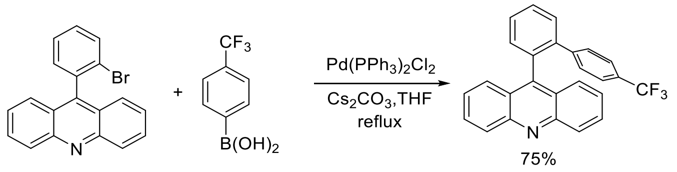

A sealed tube was charged with 9-(2-bromophenyl)acridine (**A4**) (1.67 g, 5 mmol), bis(triphenylphosphine)palladium(II) chloride (175.4 mg, 0.25 mmol), 4-trifluoromethylphenylboronic acid (1.04 g, 5.5 mmol), cesium carbonate (1.79 g, 5.5 mmol) and THF (50 mL) under an atmosphere of argon. The mixture was heated to reflux for 24 hours. The reaction was monitored by thin layer chromatography. When the reaction was completed, the solid was filtered and washed with dichloromethane. The organic layer was concentrated under reduced pressure. The crude product was purified by flash chromatography on silica gel with EtOAc/hexane, 1 : 50 v/v as an eluent to give corresponding product (1.50 g, 75%) as a yellow solid.

^{1}H NMR (400 MHz, $CDCl_3$): δ 8.21 (d, J = 8.8 Hz, 2H), 7.73 – 7.55 (m, 7H), 7.42 – 7.35 (m, 3H), 7.14 – 7.01 (m, 4H) ppm. – ^{13}C NMR (100 MHz, $CDCl_3$): δ 148.3, 145.8, 143.9, 141.0, 134.2, 131.4, 130.0, 129.7, 129.5, 128.9, 128.9, 128.5, 128.4, 127.8, 126.3, 125.7, 125.2, 125.0, 124.5, 124.5, 124.4, 124.4, 122.3 ppm. – ^{19}F NMR (376 MHz, $CDCl_3$) δ -62.7 ppm. – HRMS: calcd for $C_{26}H_{17}F_3N$ 399.1235, found 400.1307 [M+H$^+$].

Methyl 2'-(acridin-9-yl)-3-fluoro-[1,1'-biphenyl]-4-carboxylate (A9)

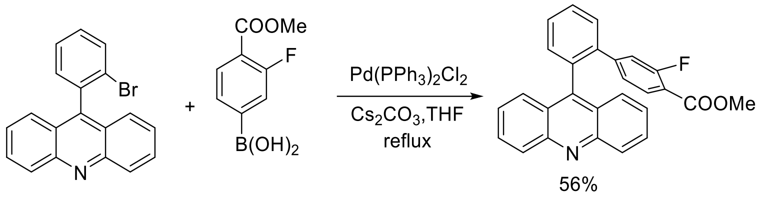

A sealed tube was charged with 9-(2-bromophenyl)acridine (**A4**) (1.67 g, 5 mmol), bis(triphenylphosphine)palladium(II) chloride (175.4 mg, 0.25 mmol), [3-fluoro-4-(methoxycarbonyl)phenyl]boronic acid (1.09 g, 5.5 mmol), cesium carbonate (1.79 g, 5.5 mmol) and THF (50 mL) under an atmosphere of argon. The mixture was heated to reflux for 24 hours. The reaction was monitored by thin layer chromatography. When the reaction was completed, the solid was filtered and washed with dichloromethane. The organic layer was concentrated under reduced pressure. The crude product was purified by flash chromatography on silica gel with EtOAc/hexane, 1 : 50 v/v as an eluent to give corresponding product (1.14 g, 56%) as a yellow solid.

^{1}H NMR (400 MHz, $CDCl_3$): δ 8.20 (d, J = 8.8 Hz, 2H), 7.74 – 7.62 (m, 3H), 7.62 – 7.55 (m, 4H), 7.43 – 7.30 (m, 4H), 6.80 (dd, J = 11.7, 1.7 Hz, 1H), 6.67 (dd, J = 8.2, 1.7 Hz, 1H), 3.72 (s, 3H) ppm. – ^{13}C NMR (100 MHz, $CDCl_3$): δ 164.1, 164.0, 162.1, 159.5, 148.2, 147.0, 147.0, 145.3, 140.0, 140.0, 134.1, 131.5, 131.2, 131.2, 129.8, 129.6, 129.0, 128.2, 126.0, 125.9, 125.1, 123.8, 123.7, 116.8, 116.6, 116.6, 116.5, 51.9 ppm. – ^{19}F NMR (376 MHz, $CDCl_3$) δ -109.7 ppm. – HRMS: calcd for $C_{27}H_{19}FNO_2$ 407.1322, found 408.1403 [M+H$^+$].

2'-(Acridin-9-yl)-[1,1'-biphenyl]-3-carbonitrile (A10)

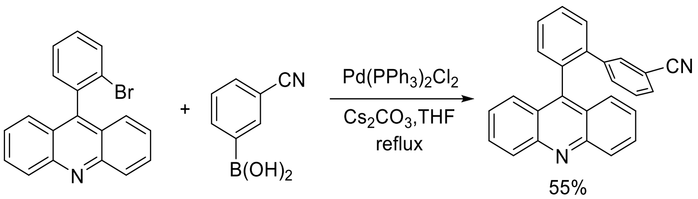

A sealed tube was charged with 9-(2-bromophenyl)acridine (**A4**) (1.67 g, 5 mmol), bis(triphenylphosphine)palladium(II) chloride (175.4 mg, 0.25 mmol), 3-cyanophenylboronic acid (0.81 g, 5.5 mmol), cesium carbonate (1.79 g, 5.5 mmol) and THF (50 mL) under an atmosphere of argon. The mixture was heated to reflux for 24 hours. The reaction was monitored by thin layer chromatography. When the reaction was completed, the solid was filtered and washed with dichloromethane. The organic layer was concentrated under reduced pressure. The crude product was purified by flash chromatography on silica gel with EtOAc/hexane, 1 : 50 v/v as an eluent to give corresponding product (0.98 g, 55%) as a yellow solid.

^{1}H NMR (400 MHz, $CDCl_3$): δ 8.20 (d, J = 8.7 Hz, 2H), 7.75 – 7.66 (m, 3H), 7.66 – 7.55 (m, 4H), 7.46 – 7.37 (m, 4H), 7.17 (dt, J = 7.7, 1.4 Hz, 1H), 6.97 (dt, J = 8.0, 1.5 Hz, 1H), 6.82 (t, J = 7.8 Hz, 1H) ppm. – ^{13}C NMR (100 MHz, $CDCl_3$): δ 148.2, 145.3, 141.5, 140.1, 134.2, 132.3, 131.7, 131.5, 130.4, 129.9, 129.9, 129.5, 129.1, 128.2, 126.0, 126.0, 125.1, 118.1, 111.8 ppm. – HRMS: calcd for $C_{26}H_{17}N_2$ 356.1313, found 357.1384 [M+H$^+$].

9-(3',5'-Di-*tert*-butyl-[1,1'-biphenyl]-2-yl)acridine (A11)

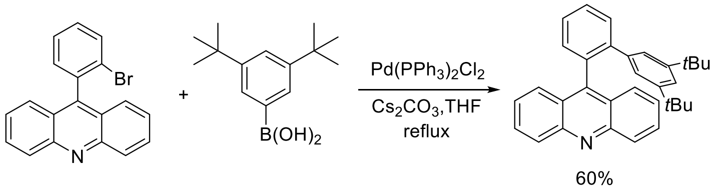

A sealed tube was charged with 9-(2-bromophenyl)acridine (0.5 g, 1.5 mmol), bis(triphenylphosphine)palladium(II) chloride (52.6 mg, 0.075 mmol), (3,5-Di-*tert*-butylphenyl)boronic acid (0.39 g, 1.65 mmol), cesium carbonate (0.54 g, 1.65 mmol) and THF (15 mL) under an atmosphere of argon. The mixture was heated to reflux for 24 hours. The reaction was monitored by TLC. When the reaction was completed, filter out the solid and wash the solid with dichloromethane. The organic layer was concentrated under reduced pressure. The crude product was purified by flash chromatography on silica gel with EtOAc/hexane, 1: 50 v/v as an eluent to give corresponding product **A11** (0.4 g, 60%) as a yellow solid.

^{1}H NMR (400 MHz, $CDCl_3$): δ 8.15 (d, J = 8.8 Hz, 2H), 7.67 (m, 6H), 7.60 – 7.54 (m, 1H), 7.47 – 7.42 (m, 1H), 7.35 (ddd, J = 8.5, 6.7, 1.2 Hz, 2H), 6.85 (t, J = 1.8 Hz, 1H), 6.74 (d, J = 1.8 Hz, 2H), 0.84 (s, 18H) ppm. – ^{13}C NMR (100 MHz, $CDCl_3$): δ 149.6, 148.6, 147.5, 144.1, 139.5, 135.0, 131.1, 129.9, 129.8, 129.5, 128.9, 127.2, 127.1, 125.6, 125.5, 123.2, 120.2, 34.4, 31.0 ppm. – HRMS: calcd for $C_{33}H_{34}N$ 444.2686,, found 444.2684 [M+H$^+$].

9-(5'-Phenyl-[1,1':3',1''-terphenyl]-2-yl)acridine (A12)

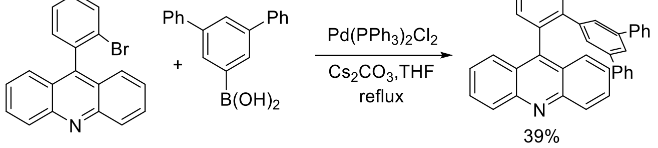

A sealed tube was charged with 9-(2-bromophenyl)acridine (0.5 g, 1.5 mmol), bis(triphenylphosphine)palladium(II) chloride (52.6 mg, 0.075 mmol), (3,5-diphenylphenyl)boronic acid (0.45 g, 1.65 mmol), cesium carbonate (0.54 g, 1.65 mmol) and THF (15 mL) under an atmosphere of argon. The mixture was heated to reflux for 24 hours. The reaction

was monitored by TLC. When the reaction was completed, filter out the solid and wash the solid with dichloromethane. The organic layer was concentrated under reduced pressure. The crude product was purified by flash chromatography on silica gel with EtOAc/hexane, 1: 50 v/v as an eluent to give corresponding product **A12** (0.28 g, 39%) as a yellow solid.

^{1}H NMR (400 MHz, $CDCl_3$): δ 8.34 – 8.27 (m, 2H), 7.78 (dd, *J* = 8.5, 6.7 Hz, 6H), 7.64 (td, *J* = 7.5, 1.4 Hz, 1H), 7.52 (dd, *J* = 7.6, 1.4 Hz, 1H), 7.46 (td, *J* = 7.2, 6.7, 1.2 Hz, 2H), 7.36 (t, *J* = 1.7 Hz, 1H), 7.27 (q, *J* = 4.8, 4.3 Hz, 6H), 7.23 (d, *J* = 1.7 Hz, 2H), 6.99 (dd, *J* = 7.6, 2.1 Hz, 4H) ppm. – ^{13}C NMR (100 MHz, $CDCl_3$): δ 148.7, 147.1, 142.6, 141.2, 141.0, 140.6, 134.8, 131.2, 130.2, 130.0, 129.8, 129.1, 128.6, 127.6, 127.3, 127.0, 127.0, 126.4, 126.0, 125.8, 124.6 ppm. – HRMS: calcd for $C_{37}H_{26}N$ 484.2060, found 484.2064 [M+H$^+$].

4-(2,2-Dicyanovinyl)phenyl 5-(2,5-dimethylphenoxy)-2,2-dimethylpentanoate (S13)

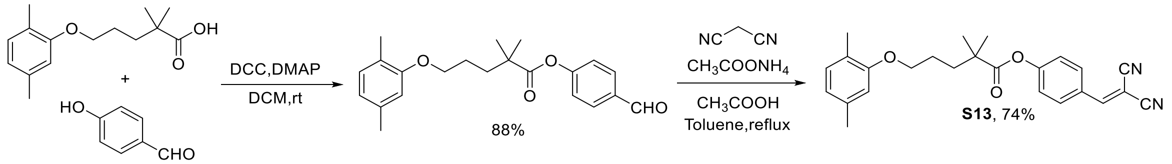

A round bottom flask was charged with gemfibrozil (0.75 mg, 3 mmol), 4-hydroxybenzaldehyde (0.244 g, 2 mmol), 4-dimethylaminopyridine (24.4 mg, 0.2 mmol) and dicyclohexylcarbodiimide (0.619 g, 3 mmol) and dichloromethane (4 mL). The mixture was stirred overnight at room temperature. The reaction was monitored by thin layer chromatography. When the reaction was completed, the solid was filtered and washed with dichloromethane. The organic layer was dried with sodium sulfate and concentrated under reduced pressure. The crude product did not require purification and can be directly sent to the next step. Malononitrile (0.106 g, 1.6 mmol) and 2 ml of toluene were added to a 15 ml sealed tube with magnetic stirring. After all dissolved, ammonium acetate (37 mg, 0.48 mmol), gemfibrozil aldehyde derivatives (0.624 g, 1.76 mmol) and acetic acid (0.05 mL) were added in turn, then heated to reflux for two hours. After cooling to room temperature, the reaction mixture was washed with a solution of saturated solution of sodium chloride (3 × 10 mL). The organic layer was dried over anhydrous Na_2SO_4, filtered and concentrated under reduced pressure. The resulting solid was recrystallized in EtOH for two times and obtained the product **S13** (0.477 g, 74%) as a yellow solid.

^{1}H NMR (400 MHz, $CDCl_3$): δ 7.98 – 7.89 (m, 2H), 7.73 (s, 1H), 7.25 – 7.18 (m, 2H), 7.01 (d, *J* = 7.4 Hz, 1H), 6.69 (d, *J* = 7.5 Hz, 1H), 6.64 (d, *J* = 1.7 Hz, 1H), 4.01 (t, *J* = 5.6 Hz, 2H), 2.32 (s, 3H), 2.18 (s, 3H), 1.90 (m, 4H), 1.41 (s, 6H) ppm. – ^{13}C NMR (100 MHz, $CDCl_3$): δ 175.6, 158.6, 156.8, 155.8, 136.6, 132.4, 130.5, 128.4, 123.6, 123.0, 121.0, 113.8, 112.6, 112.1, 82.5, 67.6, 42.8, 37.1, 25.3, 25.1, 21.5, 15.9 ppm. – HRMS: calcd for $C_{25}H_{27}N_2O_3$ 403.1943, found 403.1836 [M+H$^+$].

Ethyl 2-(4-(2,2-dicyanovinyl)phenoxy)-2-methylpropanoate (S14)

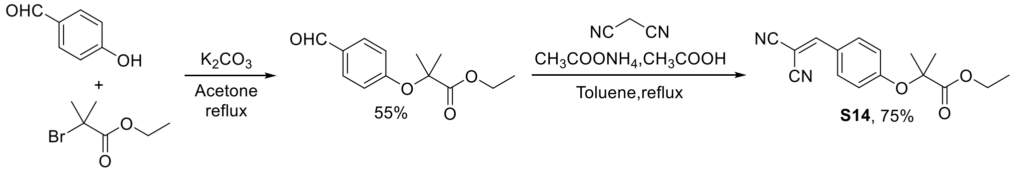

A sealed tube was charged with 4-hydroxybenzaldehyde (122.1 mg, 1 mmol), ethyl-2-bromoisobutyrate (204.8 mg, 1.05 mmol), potassium carbonate (276.4 mg, 2 mmol) and acetone (2 mL). The mixture was heated to reflux for 8 hours. The reaction was monitored by thin layer chromatography. When the reaction was completed, ethyl acetate (20 mL) was added. The diluted mixture was extracted with water and brine. The organic layer was dried with sodium sulfate and concentrated under reduced pressure. The crude product did not require purification and can be directly sent to the next step. Malononitrile (33.0 mg, 0.5 mmol) and 0.5 ml of toluene were added to a 15 ml sealed tube with magnetic

stirring. After all dissolved, ammonium acetate (11.6 mg, 0.15 mmol), corresponding aldehyde (129.9 mg, 0.55 mmol) and acetic acid (0.02 mL) were added in turn, then heated to reflux for two hours. After cooling to room temperature, the reaction mixture was washed with a solution of saturated solution of sodium chloride (3 × 5 mL). The organic layer was dried over anhydrous Na_2SO_4, filtered and concentrated under reduced pressure. The resulting solid was recrystallized in EtOH for two times and obtained the product **S14** (106.6 mg, 75%) as a yellow solid.

^{1}H NMR (400 MHz, $CDCl_3$): δ 7.88 – 7.80 (m, 2H), 7.64 (s, 1H), 6.92 – 6.83 (m, 2H), 4.23 (q, *J* = 7.1 Hz, 2H), 1.67 (s, 6H), 1.22 (t, *J* = 7.1 Hz, 3H) ppm. – ^{13}C NMR (100 MHz, $CDCl_3$): δ 173.2, 161.4, 158.9, 133.1, 124.4, 118.3, 114.4, 80.0, 79.1, 62.0, 25.5, 14.1 ppm. – HRMS: calcd for $C_{16}H_{17}N_2O_3$ 285.1161, found 285.1055 [M+H$^+$].

4-(2,2-Dicyanovinyl)phenyl 3-(4,5-diphenyl-4,5-dihydrooxazol-2-yl)propanoate (S15)

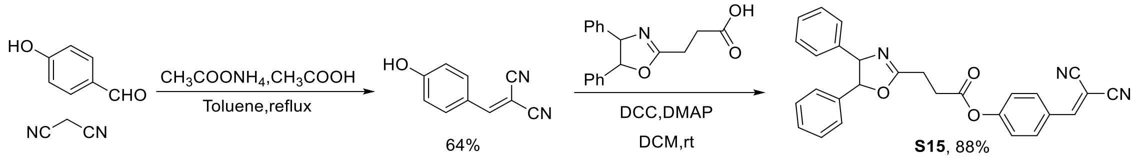

Malononitrile (330.3 mg, 5 mmol) and 5 ml of toluene were added to a 15 ml sealed tube with magnetic stirring. After all dissolved, ammonium acetate (115.6 mg, 1.5 mmol), 4-hydroxybenzaldehyde (671.7 mg, 5.5 mmol) and acetic acid (0.15 mL) were added in turn, then heated to reflux for two hours. After cooling to room temperature, the reaction mixture was washed with a solution of saturated solution of sodium chloride (3 × 15 mL). The organic layer was dried over anhydrous Na_2SO_4, filtered and concentrated under reduced pressure. The resulting solid was recrystallized in EtOH for two times and obtained the product 2-(4-hydroxybenzylidene)malononitrile (544.5 mg, 64%) as a white solid. This product will be used as the substrate for the next step. A round bottom flask was charged with oxaprozin (586.6 mg, 2 mmol), 2-(4-hydroxybenzylidene)malononitrile (340.3 mg, 2 mmol), 4-dimethylaminopyridine (24.4 mg, 0.2 mmol) and dicyclohexylcarbodiimide (618.9 mg, 3 mmol) and dichloromethane (10 mL). The mixture was stirred overnight at room temperature. The reaction was monitored by thin layer chromatography. When the reaction was completed, the solid was filtered and washed with dichloromethane. The organic layer was dried with sodium sulfate and concentrated under reduced pressure. The crude product was purified by flash chromatography on silica gel with EtOAc/hexane, 1 : 40 v/v as an eluent to give corresponding gemfibrozil aldehyde derivative **S15** (840.4 mg, 88%) as a yellow oil.

^{1}H NMR (400 MHz, $CDCl_3$): δ 7.92 (d, *J* = 8.8 Hz, 2H), 7.73 (s, 1H), 7.66 – 7.60 (m, 2H), 7.59 – 7.55 (m, 2H), 7.35 (m, 8H), 3.32 (t, *J* = 6.8 Hz, 2H), 3.21 (t, *J* = 6.8 Hz, 2H) ppm. – ^{13}C NMR (100 MHz, $CDCl_3$): δ 169.9, 163.2, 161.3, 159.1, 158.6, 155.2, 145.9, 135.1, 133.8, 132.3, 132.1, 128.8, 128.7, 128.7, 128.5, 128.4, 128.0, 126.5, 123.3, 122.9, 116.9, 114.7, 113.6, 113.6, 112.5, 82.6, 31.2, 23.4 ppm. – HRMS: calcd for $C_{28}H_{22}N_3O_3$ 448.1583, found 448.1385 [M+H$^+$].

Methyl 2-(11-oxo-6,11-dihydrodibenzo[*b*,*e*]oxepin-2-yl)acrylate (S16)

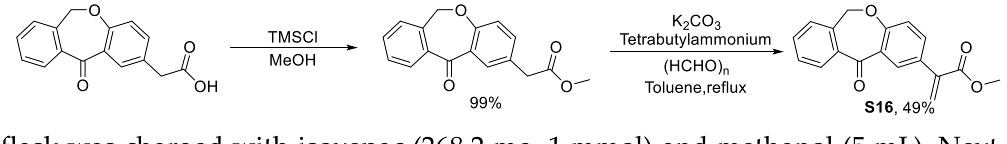

A round bottom flask was charged with isoxepac (268.2 mg, 1 mmol) and methanol (5 mL). Next, TMSCl (0.38 ml,3 mmol) was slowly added, and the reaction mixture was stirred overnight at room temperature. The reaction was monitored by thin layer chromatography. When the reaction was completed, the mixture was concentrated in vacuo to get corresponding ester without further purification. A sealed tube was charged with crude ester (282.3 mg, 1 mmol), tetrabutylammonium hydrogen sulfate (34.0 mg, 0.1 mmol), K_2CO_3 (207.3 mg, 1.5 mmol), paraformaldehyde (780.2 mg, 6 mmol) and toluene (8 mL). The mixture was heated to reflux overnight. The reaction was monitored by thin layer chromatography. When the reaction was completed, the reaction mixture was filtered, the organic layer was

concentrated under reduced pressure. The crude product was purified by flash chromatography on silica gel with EtOAc/hexane, 1 : 20 v/v as an eluent to give corresponding product **S16** (144.2 mg, 49%) as a faint yellow oil.

^{1}H NMR (400 MHz, $CDCl_3$): δ 8.18 (d, *J* = 2.4 Hz, 1H), 7.78 (dd, *J* = 7.6, 1.4 Hz, 1H), 7.50 – 7.40 (m, 2H), 7.36 (td, *J* = 7.6, 1.3 Hz, 1H), 7.29 – 7.21 (m, 1H), 6.93 (d, *J* = 8.5 Hz, 1H), 6.28 (d, *J* = 1.1 Hz, 1H), 5.85 (d, *J* = 1.1 Hz, 1H), 5.09 (s, 2H), 3.73 (s, 3H) ppm. – ^{13}C NMR (100 MHz, $CDCl_3$): δ 190.7, 167.0, 161.2, 140.5, 139.8, 135.4, 135.3, 132.8, 131.7, 130.7, 129.5, 129.3, 127.9, 126.9, 124.9, 120.5, 73.6, 52.3 ppm. – HRMS: calcd for $C_{18}H_{15}O_4$ 295.0892, found 295.0971 [M+H$^+$].

Ethyl 2-(4-isobutylphenyl)acrylate (S17)

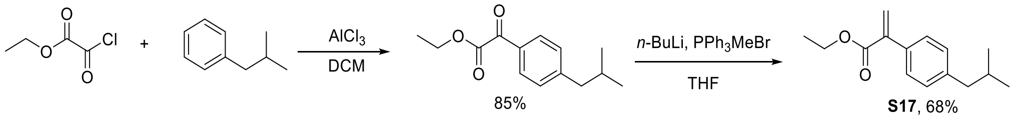

To a solution of aluminium chloride (1.60 g, 12 mmol) in dichloromethane (15 ml), ethyl chlorooxoacetate (1.12 ml, 10 mmol) was added at 0 °C. Isobutylbenzene (0.8 ml, 5 mmol) was added and the reaction was allowed to warm up to room temperature slowly. The reaction was monitored by thin layer chromatography. When the reaction was completed, the mixture was added crushed ice to quench and added hydrochloric acid for acidification. The mixture was extracted with dichloromethane for three times (3 × 30 mL). The organic layer was washed with saturated sodium bicarbonate and saturated saline solution. The organic layer was dried with sodium sulfate and concentrated under reduced pressure. The crude product did not require purification and can be directly sent to the next step. To a suspension of methyltriphenylphosphonium bromide (1.1271 g, 3.2 mmol) in tetrahydrofuran (12 ml) was added *n*-BuLi (1.28 ml, 3.2 mmol, 2.5 mol/L in hexane,) at 0 °C under argon. Ethyl 2-(4-isobutylphenyl)-2-oxoacetate was added after 15 minutes and the reaction mixture was warmed to room temperature. The reaction was monitored by thin layer chromatography. After the reaction was completed, the reaction mixture was quenched with a saturated ammonium chloride solution and extracted with ethyl acetate for 2 times. The combined organic layers were dried over sodium sulfate and concentrated in vacuo. The crude product was purified by flash chromatography on silica gel with EtOAc/hexane, 1 : 50 v/v as an eluent to give corresponding product **S17** (315.9 mg, 68%) as a faint yellow oil.

^{1}H NMR (400 MHz, $CDCl_3$): δ 7.39 (d, J = 8.1 Hz, 2H), 7.17 (d, J = 8.1 Hz, 2H), 6.32 (d, J = 1.4 Hz, 1H), 5.89 (d, J = 1.4 Hz, 1H), 4.32 (q, J = 7.2 Hz, 2H), 2.52 (d, J = 7.2 Hz, 2H), 1.93 (dh, J = 13.5, 6.7 Hz, 1H), 1.36 (t, J = 7.1 Hz, 3H), 0.96 (d, J = 6.7 Hz, 6H) ppm. – ^{13}C NMR (100 MHz, $CDCl_3$): δ 166.9, 141.7, 141.5, 134.1, 128.8, 128.0, 125.5, 61.0, 45.2, 30.2, 22.4, 14.2 ppm. – HRMS: calcd for $C_{15}H_{21}O_2$ 233.1463, found 233.1358 [M+H$^+$].

***N*-(4-(1-Butyl-3-ethyl-2,6-dioxopiperidin-3-yl)phenyl)-2-phenylacrylamide (S18)**

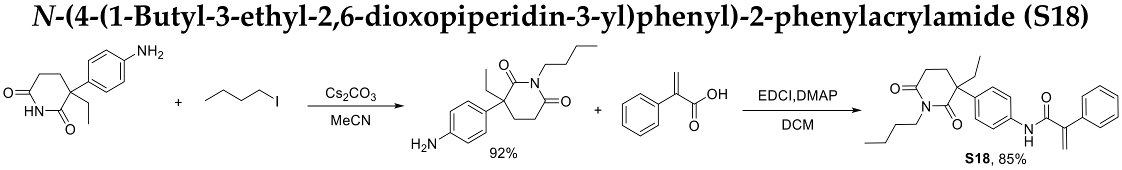

To a solution of aminoglutethimide (0.2323 mg, 1 mmol) and cesium carbonate (977.4 mg, 3 mmol) in acetonitrile (5 ml), 1-iodobutane (0.24 ml, 2 mmol) was added at 0 °C. The reaction was rapidly warmed to 40 °C with oil bath. The reaction was monitored by thin layer chromatography. When the reaction was completed, the solid was filtered and washed with dichloromethane. The organic layer was dried with sodium sulfate and concentrated under reduced pressure. The crude product did not require purification and can be directly sent to the next step. A round bottom flask was charged with Butylated derivatives of aminoglutethimide (288.4 mg, 1 mmol), atropic acid (162.9 mg, 1.1 mmol), 4-dimethylaminopyridine (12.2 mg, 0.1 mmol) and EDCI (287.6 mg, 1.5 mmol) and dichloromethane (5 mL). The mixture was stirred overnight at room temperature. The reaction was monitored by thin layer chromatography. When the reaction was completed, concentrated under reduced pressure. The crude product was purified by flash

chromatography on silica gel with EtOAc/hexane, 1 : 3 v/v as an eluent to give corresponding product **S18** (355.8 mg, 85%) as a yellow solid.

^{1}H NMR (400 MHz, $CDCl_3$): δ 7.50 (d, J = 8.5 Hz, 2H), 7.41 (m, 5H), 7.16 (d, J = 8.5 Hz, 2H), 6.29 (s, 1H), 5.73 (d, J = 1.2 Hz, 1H), 3.88 – 3.72 (m, 2H), 2.63 (m, 1H), 2.43 (m, 1H), 2.28 (m, 1H), 2.15 (m, 1H), 2.02 (m, 1H), 1.88 (m, 1H), 1.47 (m, 2H), 1.38 – 1.27 (m, 2H), 0.92 (t, J = 7.3 Hz, 3H), 0.84 (t, J = 7.4 Hz, 3H) ppm. – ^{13}C NMR (100 MHz, $CDCl_3$): δ 175.0, 172.0, 165.3, 144.9, 136.9, 136.5, 135.7, 129.0, 128.9, 128.3, 126.9, 123.6, 120.2, 51.1, 40.1, 33.8, 30.0, 29.8, 29.7, 25.9, 20.2, 13.8, 9.1 ppm. – HRMS: calcd for $C_{26}H_{31}N_2O_3$ 419.2256, found 419.2334 [M+H$^+$].

Gram scale reaction

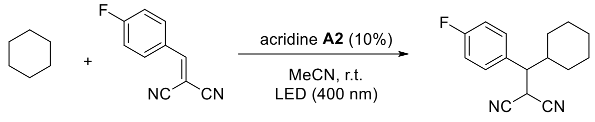

4i, 68%,1.044 g

An oven-dried 250 mL round bottom flask equipped with a stir bar was charged with 2-(4-fluorobenzylidene)malononitrile (1.03 g, 6 mmol), acridine **A2** (283.2 mg, 10 mol%, 0.6 mmol), cyclohexane (3.6 ml, 30 mmol) and acetonitrile (60 mL). The reaction tube was degassed with argon and capped and the irradiated with LED light (λ_{max} = 400 nm) at room temperature for 24 hours. The reaction mixture was concentrated under reduced pressure and purified by flash chromatography on silica gel with EtOAc/petroleum ether (1 : 50 v/v) as an eluent to give the corresponding product **4i** (1.04 g, 68%) as a yellow oil.

^{1}H NMR (400 MHz, $CDCl_3$): δ 7.36 – 7.27 (m, 2H), 7.10 (t, *J* = 8.6 Hz, 2H), 4.18 (d, *J* = 5.4 Hz, 1H), 2.87 (dd, *J* = 9.9, 5.4 Hz, 1H), 2.04 – 1.78 (m, 3H), 1.75 – 1.61 (m, 2H), 1.50 – 1.30 (m, 2H), 1.28 – 1.12 (m, 2H), 1.12 – 0.98 (m, 1H), 0.86 – 0.73 (m, 1H) ppm. – ^{13}C NMR (100 MHz, $CDCl_3$): δ 164.1, 161.6, 132.4, 132.4, 130.1, 130.0, 116.3, 116.0, 112.1, 111.7, 51.6, 39.2, 31.1, 30.6, 27.2, 27.2, 25.8, 25.7, 25.7 ppm. – ^{19}F NMR (376 MHz, $CDCl_3$) δ -112.9 ppm. – HRMS: calcd for $C_{16}H_{18}FN_2$ 257.1376, found 257.1271 [M+H$^+$].

Product characterization

1-(Cyclohexyloxy)-2,2,6,6-tetramethylpiperidine (3a)

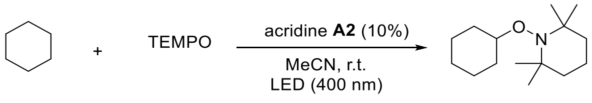

3a, 94%

The general procedure **GP1** was followed with TEMPO (31.3 mg, 0.2 mmol), acridine **A2** (4.7 mg, 10 mol%, 0.01 mmol), cyclohexane (0.06 ml, 0.5 mmol) and acetonitrile (2 mL). The tube was capped with a screw cap and wrapped with parafilm then irradiated with LED light (λ_{max} = 400 nm) at room temperature for 24 hours. The reaction mixture was concentrated under reduced pressure and purified by flash chromatography on silica gel with EtOAc/petroleum ether (1 : 100 v/v) as an eluent to give the corresponding product **3a** (22.4 mg, 94%) as a yellow oil.

^{1}H NMR (400 MHz, $CDCl_3$): δ 3.52 (tt, *J* = 9.2, 4.1 Hz, 1H), 1.98 (m, 2H), 1.66 (m, 2H), 1.48 – 1.33 (m, 6H), 1.10 (m, 18H) ppm. – ^{13}C NMR (100 MHz, $CDCl_3$): δ 81.7, 59.6, 40.3, 32.9, 26.0, 25.1, 17.3 ppm. – HRMS: calcd for $C_{15}H_{30}NO$: 240.2328, found 240.2322 [M+H$^+$].

1-(Cyclopentyloxy)-2,2,6,6-tetramethylpiperidin-4-ol (3b)

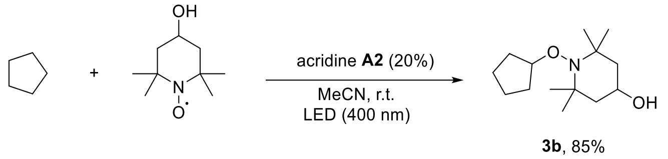

The general procedure **GP1** was followed with 4-OH-TEMPO (34.4 mg, 0.2 mmol), acridine **A2** (9.4 mg, 20 mol%, 0.02 mmol), cyclopentane (0.06 ml, 0.5 mmol) and acetonitrile (2 mL). The tube was capped with a screw cap and wrapped with parafilm then irradiated with LED light (λ_{max} = 400 nm) at room temperature for 24 hours. The reaction mixture was concentrated under reduced pressure and purified by flash chromatography on silica gel with EtOAc/petroleum ether (1 : 10 v/v) as an eluent to give the corresponding product **3b** (20.5 mg, 85%) as a yellow solid.

^{1}H NMR (400 MHz, $CDCl_3$): δ 4.23 (m, 1H), 3.93 (tt, *J* = 11.3, 4.3 Hz, 1H), 1.91 (m, 2H), 1.79 (m, 2H), 1.62 (m, 4H), 1.50 – 1.37 (m, 4H), 1.21 (s, 6H), 1.11 (s, 6H) ppm. – ^{13}C NMR (100 MHz, $CDCl_3$): δ 88.4, 63.4, 59.8, 48.7, 34.2, 32.7, 23.4, 21.2 ppm. – HRMS: calcd for $C_{14}H_{28}NO_2$ 242.2042, found 242.2112 [M+H$^+$].

1-(Cycloheptyloxy)-2,2,6,6-tetramethylpiperidin-4-ol (3c)

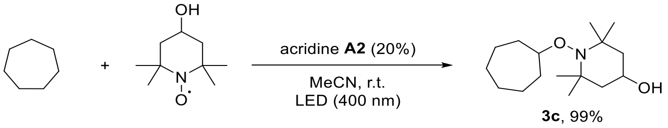

The general procedure **GP1** was followed with 4-OH-TEMPO (34.4 mg, 0.2 mmol), acridine **A2** (9.4 mg, 20 mol%, 0.02 mmol), cycloheptane (0.07 ml, 0.5 mmol) and acetonitrile (2 mL). The tube was capped with a screw cap and wrapped with parafilm then irradiated with LED light (λ_{max} = 400 nm) at room temperature for 24 hours. The reaction mixture was concentrated under reduced pressure and purified by flash chromatography on silica gel with EtOAc/petroleum ether (1 : 10 v/v) as an eluent to give the corresponding product **3c** (26.7 mg, 99%) as a white solid.

^{1}H NMR (400 MHz, $CDCl_3$): δ 3.94 (tt, *J* = 11.1, 4.2 Hz, 1H), 3.82 (tt, *J* = 8.5, 4.4 Hz, 1H), 2.07 – 1.94 (m, 2H), 1.85 – 1.72 (m, 2H), 1.69 – 1.26 (m, 12H), 1.15 (d, *J* = 10.7 Hz, 12H) ppm. – ^{13}C NMR (100 MHz, $CDCl_3$): δ 84.0, 63.4, 60.1, 48.9, 34.4, 33.4, 28.6, 23.4, 21.3 ppm. – HRMS: calcd for $C_{16}H_{32}NO_2$ 270.2355, found 270.2426 [M+H$^+$].

1-(Cyclooctyloxy)-2,2,6,6-tetramethylpiperidin-4-ol (3d)

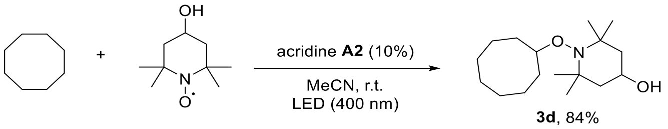

The general procedure **GP1** was followed with 4-OH-TEMPO (34.4 mg, 0.2 mmol), acridine **A2** (9.4 mg, 20 mol%, 0.02 mmol), cyclooctane (0.07 ml, 0.5 mmol) and acetonitrile (2 mL). The tube was capped with a screw cap and wrapped with parafilm then irradiated with LED light (λ_{max} = 400 nm) at room temperature for 24 hours. The reaction mixture was concentrated under reduced pressure and purified by flash chromatography on silica gel with EtOAc/petroleum ether (1 : 10 v/v) as an eluent to give the corresponding product **3d** (23.8 mg, 84%) as a yellow solid.

^{1}H NMR (400 MHz, $CDCl_3$): δ 3.94 (tt, *J* = 11.1, 4.2 Hz, 1H), 3.83 (tt, *J* = 8.2, 3.7 Hz, 1H), 1.97 (m, , 2H), 1.84 – 1.74 (m, 2H), 1.73 – 1.34 (m, 14H), 1.15 (d, *J* = 11.0 Hz, 12H) ppm. – ^{13}C NMR (100 MHz, $CDCl_3$): δ 83.4, 63.4, 60.1, 48.9, 34.5, 31.1, 27.5, 25.7, 23.6, 21.3 ppm. – HRMS: calcd for $C_{17}H_{34}NO_2$ 284.2511, found284.2582 [M+H$^+$].

Go to table of contents

1-(2,3-Dimethylbutoxy)-2,2,6,6-tetramethylpiperidin-4-ol (3e-α) and 1-((2,3-Dimethylbutan-2-yl)oxy)-2,2,6,6-tetramethylpiperidin-4-ol (3e-β)

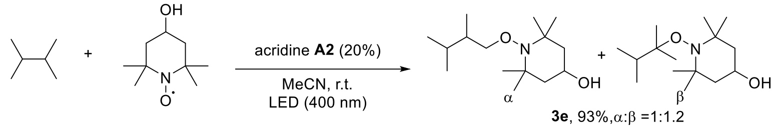

The general procedure **GP1** was followed with 4-OH-TEMPO (34.4 mg, 0.2 mmol), acridine **A2** (9.4 mg, 20 mol%, 0.02 mmol), 2,3-dimethylbutane (0.28 mL, 2 mmol) and acetonitrile (2 mL). The tube was capped with a screw cap and wrapped with parafilm then irradiated with LED light (λ_{max} = 400 nm) at room temperature for 24 hours. The reaction mixture was concentrated under reduced pressure and purified by flash chromatography on silica gel with EtOAc/petroleum ether (1 : 10 v/v) as an eluent to give the corresponding product **3e** (23.9 mg, 93%) as yellow oil.

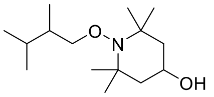

^{1}H NMR (400 MHz, CDCl$_3$): δ 3.94 (tt, *J* = 11.1, 4.2 Hz, 1H), 3.70 (dd, *J* = 8.5, 5.4 Hz, 1H), 3.53 (t, *J* = 7.9 Hz, 1H), 1.87 – 1.74 (m, 2H), 1.72 – 1.52 (m, 3H), 1.45 (t, *J* = 11.8 Hz, 2H), 1.17 (dd, *J* = 17.6, 2.5 Hz, 12H), 0.86 (dd, *J* = 15.6, 6.7 Hz, 8H) ppm. – ^{13}C NMR (100 MHz, CDCl$_3$): δ 80.3, 63.4, 48.4, 38.8, 33.2, 29.8, 21.1, 20.4, 18.6, 13.7 ppm. – HRMS: calcd for $C_{15}H_{32}NO_2$ 258.2355, found 258.2430 [M+H$^+$].

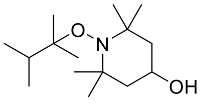

^{1}H NMR (400 MHz, CDCl$_3$): δ 3.94 (tt, *J* = 11.1, 4.2 Hz, 1H), 2.03 (hept, *J* = 6.9 Hz, 1H), 1.82 (ddt, *J* = 12.8, 3.9, 2.0 Hz, 2H), 1.45 (t, *J* = 11.8 Hz, 2H), 1.22 – 1.10 (m, 18H), 0.91 (d, *J* = 6.8 Hz, 6H) ppm. – ^{13}C NMR (100 MHz, CDCl$_3$): δ 81.7, 63.3, 59.8, 49.5, 38.5, 35.0, 22.7, 21.7, 18.3 ppm. – HRMS: calcd for $C_{15}H_{32}NO_2$ 258.2355, found 258.2427 [M+H$^+$].

1-(((3*S*,5*S*,7*S*)-Adamantan-1-yl)oxy)-2,2,6,6-tetramethylpiperidin-4-ol (3f-α) and 1-(((1*R*,3*R*,5*R*,7*R*)-Adamantan-2-yl)oxy)-2,2,6,6-tetramethylpiperidin-4-ol (3f-β)

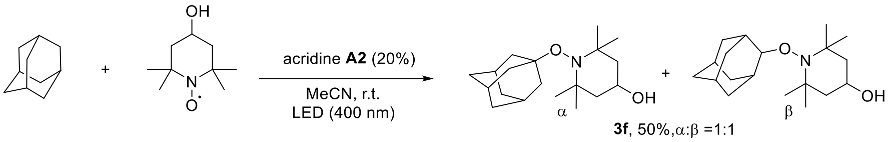

The general procedure **GP1** was followed with 4-OH-TEMPO (34.4 mg, 0.2 mmol), acridine **A2** (9.4 mg, 20 mol%, 0.02 mmol), adamantane (0.136 mg, 1 mmol) and acetonitrile (2 mL). The tube was capped with a screw cap and wrapped with parafilm then irradiated with LED light (λ_{max} = 400 nm) at room temperature for 24 hours. The reaction mixture was concentrated under reduced pressure and purified by flash chromatography on silica gel with EtOAc/petroleum ether (1 : 5 v/v) as an eluent to give the corresponding product **3f** (15.7 mg, 50%) as a white solid.

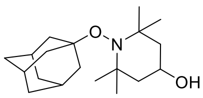

^{1}H NMR (400 MHz, CDCl$_3$): δ 4.00 – 3.85 (m, 1H), 2.18 – 2.06 (m, 3H), 1.89 (d, *J* = 3.1 Hz, 6H), 1.83 (ddt, *J* = 12.9, 3.9, 2.0 Hz, 2H), 1.61 – 1.56 (m, 6H), 1.43 (t, *J* = 11.7 Hz, 2H), 1.22 (s, 6H), 1.14 (s, 6H) ppm. – ^{13}C NMR (100 MHz, CDCl$_3$): δ 63.3, 59.7, 49.5, 42.5, 36.5, 35.4, 31.4, 21.5 ppm. – HRMS: calcd for $C_{19}H_{34}NO_2$ 308.2511, found 308.2585 [M+H$^+$].

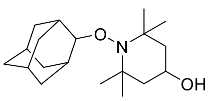

^{1}H NMR (400 MHz, CDCl$_3$): δ 3.96 (m, 1H), 3.73 (t, *J* = 3.4 Hz, 1H), 2.26 – 2.15 (m, 2H), 2.10 – 2.00 (m, 2H), 1.86 – 1.41 (m, 14H), 1.19 (d, *J* = 5.7 Hz, 12H) ppm. – ^{13}C NMR (100 MHz, CDCl$_3$): δ 86.3, 60.2, 48.9, 37.4, 37.2, 32.5, 32.1, 27.4, 27.1 ppm. – HRMS: calcd for $C_{19}H_{34}NO_2$ 308.2511, found 308.2583 [M+H$^+$].

1-((2,3-Dimethylbut-2-en-1-yl)oxy)-2,2,6,6-tetramethylpiperidin-4-ol (3g-A) and 1-((2,3-dimethylbut-3-en-2-yl)oxy)-2,2,6,6-tetramethylpiperidin-4-ol (3g-B)

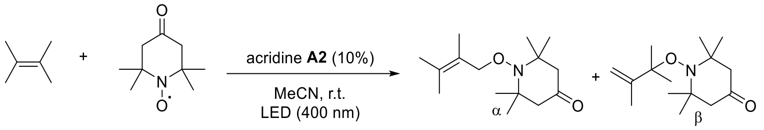

3g, 89%, α : β = 2.3 : 1

The general procedure **GP1** was followed with 4-Oxo-TEMPO (34.0 mg, 0.2 mmol), acridine **A2** (4.7 mg, 10 mol%, 0.01 mmol), 2,3-dimethylbut-2-ene (0.12 ml, 1 mmol) and acetonitrile (2 mL). The tube was capped with a screw cap and wrapped with parafilm then irradiated with LED light (λ_{max} = 400 nm) at room temperature for 24 hours. The reaction mixture was concentrated under reduced pressure and purified by flash chromatography on silica gel with EtOAc/petroleum ether (1 : 30 v/v) as an eluent to give the corresponding product **3g** (22.6 mg, 89%) as a yellow oil.

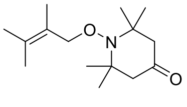

^{1}H NMR (400 MHz, $CDCl_3$): δ 4.35 (s, 2H), 2.58 (d, *J* = 12.8 Hz, 2H), 2.20 (d, *J* = 12.8 Hz, 2H), 1.75 (dd, *J* = 10.2, 1.8 Hz, 6H), 1.69 (s, 3H), 1.35 (s, 6H), 1.16 (s, 6H) ppm. – ^{13}C NMR (100 MHz, $CDCl_3$): δ 208.5, 129.9, 124.7, 78.0, 63.2, 53.6, 32.8, 22.6, 20.9, 20.8, 17.3 ppm. – HRMS: calcd for $C_{15}H_{28}NO_2$ 254.2042, found 254.1942 [M+H$^+$].

^{1}H NMR (400 MHz, $CDCl_3$): δ 4.84 (s, 1H), 4.72 (t, *J* = 1.5 Hz, 1H), 2.58 (d, *J* = 13.0 Hz, 2H), 2.22 – 2.16 (m, 2H), 1.86 (d, *J* = 1.4 Hz, 3H), 1.44 (s, 6H), 1.21 (s, 6H), 1.14 (s, 6H) ppm. – ^{13}C NMR (100 MHz, $CDCl_3$): δ 81.7, 63.3, 59.8, 49.5, 38.5, 35.0, 22.7, 21.7, 18.3 ppm. – HRMS: calcd for $C_{15}H_{28}NO_2$ 254.2042, found 254.1943 [M+H$^+$].

1-(Cyclohex-2-en-1-yloxy)-2,2,6,6-tetramethylpiperidin-4-ol (3h)

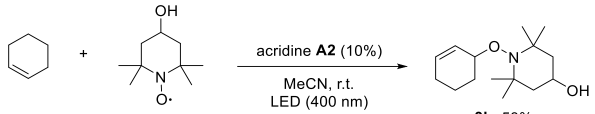

3h, 53%

The general procedure **GP1** was followed with 4-OH-TEMPO (34.4 mg, 0.2 mmol), acridine **A2** (4.7 mg, 10 mol%, 0.01 mmol), cyclohexene (0.1 ml, 0.4 mmol) and acetonitrile (2 mL). The tube was capped with a screw cap and wrapped with parafilm then irradiated with LED light (λ_{max} = 400 nm) at room temperature for 24 hours. The reaction mixture was concentrated under reduced pressure and purified by flash chromatography on silica gel with EtOAc/petroleum ether (1 : 100 v/v) as an eluent to give the corresponding product **3h** (13.4 mg, 53%) as a brown solid.

^{1}H NMR (400 MHz, $CDCl_3$): δ 5.97 – 5.89 (m, 1H), 5.85 – 5.77 (m, 1H), 4.23 (s, 1H), 3.96 (dd, *J* = 14.0, 9.0 Hz, 1H), 2.12 – 1.08 (m, 23H) ppm. – ^{13}C NMR (100 MHz, $CDCl_3$): δ 130.3, 128.9, 77.6, 63.5, 60.5, 59.9, 49.0, 34.8, 34.6, 29.6, 25.5, 21.4, 21.3, 19.8 ppm. – HRMS: calcd for $C_{15}H_{28}NO_2$ 254.2042, found 254.2118 [M+H$^+$].

1-(Benzyloxy)-2,2,6,6-tetramethylpiperidin-4-ol (3i)

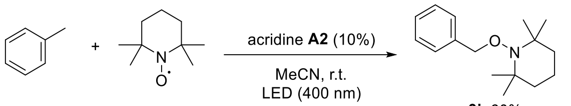

3i, 60%

The general procedure **GP1** was followed with TEMPO (31.3 mg, 0.2 mmol), acridine **A2** (4.7 mg, 10 mol%, 0.01 mmol), toluene (0.043 ml, 0.4 mmol) and acetonitrile (2 mL). The tube was capped with a screw cap and wrapped with parafilm then irradiated with LED light (λ_{max} = 400 nm) at room temperature for 24 hours. The reaction mixture was concentrated under reduced pressure and purified by flash chromatography on silica gel with EtOAc/petroleum ether (1 : 100 v/v) as an eluent to give the corresponding product **3i** (14.8 mg, 60%) as a yellow solid.

^{1}H NMR (400 MHz, $CDCl_3$): δ 7.42 – 7.31 (m, 4H), 7.29 (dd, *J* = 5.3, 3.1 Hz, 1H), 4.83 (s, 2H), 1.63 – 1.44 (m, 5H), 1.40 – 1.31 (m, 1H), 1.26 (s, 6H), 1.16 (s, 6H) ppm. – ^{13}C NMR (100 MHz, $CDCl_3$): δ 138.5, 128.4, 127.6, 127.4, 78.9, 60.2, 39.9, 33.2, 20.5, 17.3 ppm. – HRMS: calcd for $C_{16}H_{26}NO$ 248.1936, found 248.2001 [M+H$^+$].

2,2,6,6-Tetramethyl-1-(1-phenylethoxy)piperidine (3j-α) and 2,2,6,6-Tetramethyl-1-phenethoxypiperidine (3j-β)

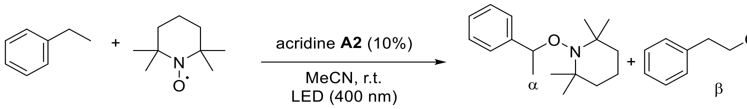

3j, 92%, α : β = 12 : 1

The general procedure **GP1** was followed with TEMPO (31.3 mg, 0.2 mmol), acridine **A2** (4.7 mg, 10 mol%, 0.01 mmol), ethylbenzene (0.053 ml, 0.5 mmol) and acetonitrile (2 mL). The tube was capped with a screw cap and wrapped with parafilm then irradiated with LED light (λ_{max} = 400 nm) at room temperature for 24 hours. The reaction mixture was concentrated under reduced pressure and purified by flash chromatography on silica gel with EtOAc/petroleum ether (1 : 100 v/v) as an eluent to give the corresponding product **3j** (24.0 mg, 92%) as a brown solid.

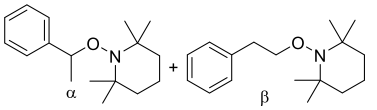

^{1}H NMR (400 MHz, $CDCl_3$): δ 7.30 – 7.08 (m, 5.43H), 4.70 (q, *J* = 6.7 Hz, 1H), 3.87(t, *J* = 6.7 Hz, 0.17H), 2.75(t, *J* = 6.7 Hz, 0.17H), 1.52 – 0.87 (m, 19.53H), 0.58 (s, 3H). – ^{13}C NMR (100 MHz, $CDCl_3$): δ 146.0, 129.2, 128.2, 128.1, 126.9, 126.7, 126.0, 83.2, 59.8, 59.8, 40.5, 39.7, 35.5, 34.6, 34.2, 33.1, 23.7, 20.5, 17.4, 17.3 ppm. – HRMS: calcd for $C_{17}H_{28}NO$ 262.2093, found 262.2166 [M+H$^+$].

2,2,6,6-Tetramethyl-1-((1,2,3,4-tetrahydronaphthalen-1-yl)oxy)piperidin-4-ol (3k-α) and 2,2,6,6-Tetramethyl-1-((1,2,3,4-tetrahydronaphthalen-2-yl)oxy)piperidin-4-ol (3k-β)

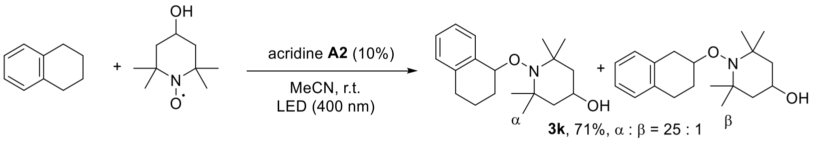

3k, 71%, α : β = 25 : 1

The general procedure **GP1** was followed with 4-OH-TEMPO (34.4 mg, 0.2 mmol), acridine **A2** (4.7 mg, 10 mol%, 0.01 mmol), 1,2,3,4-tetrahydronaphthalene (0.053 mg, 0.4 mmol) and acetonitrile (2 mL). The tube was capped with a screw cap and wrapped with parafilm then irradiated with LED light (λ_{max} = 400 nm) at room temperature for 24 hours. The reaction mixture was concentrated under reduced pressure and purified by flash chromatography on silica gel with EtOAc/petroleum ether (1 : 10 v/v) as an eluent to give the corresponding product **3k** (21.5 mg, 71%) as a white solid.

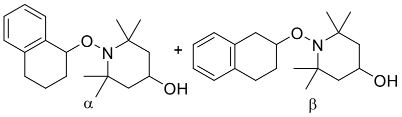

^{1}H NMR (400 MHz, $CDCl_3$): δ 7.58 (dd, *J* = 7.5, 1.7 Hz, 1H), 7.22 – 7.12 (m, 2H), 7.08 (d, *J* = 7.4 Hz, 1H), 4.88 (t, *J* = 4.8 Hz, 1H), 3.97 (tt, *J* = 11.4, 4.4 Hz, 1H), f3.55(d, *J* = 5.9 Hz, 0.04H), 2.85 (dt, *J* = 17.0, 5.9 Hz, 1H), 2.73 (dt, *J* = 17.0, 5.9 Hz, 1H), 2.14 – 1.96 (m, 3H), 1.85 (dt, *J* = 12.4, 4.0 Hz, 1H), 1.73 (m, 2H), 1.53 (t, *J* = 12.0 Hz, 1H), 1.40 (t, *J* = 12.0 Hz, 1H), 1.29 (m, 4H), 1.20 (d, *J* = 4.0 Hz, 6H), 0.68 (s, 3H). – ^{13}C NMR (100 MHz, $CDCl_3$): δ 137.9, 137.5, 130.3, 128.7, 127.5, 124.9, 78.3, 63.6, 60.6, 60.6, 49.1, 34.9, 33.4, 29.1, 29.1, 21.9, 21.6, 19.1 ppm. – HRMS: calcd for $C_{19}H_{30}NO_2$ 304.2198, found 304.2269 [M+H$^+$].

2,2,6,6-Tetramethyl-1-((6-methylpyridin-2-yl)methoxy)piperidin-4-ol (3l)

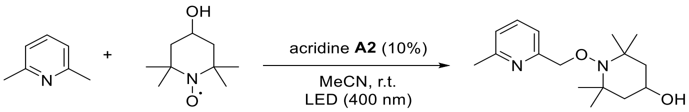

3l, 54%

The general procedure **GP1** was followed with 4-OH-TEMPO (34.4 mg, 0.2 mmol), acridine **A2** (4.7 mg, 10 mol%, 0.01 mmol), 2,6-dimethylpyridine (107.2 mg, 1 mmol) and acetonitrile (2 mL). The tube was capped with a screw cap and wrapped with parafilm then irradiated with LED light (λ_{max} = 400 nm) at room temperature for 24 hours. The reaction mixture was concentrated under reduced pressure and purified by flash chromatography on silica gel with EtOAc/petroleum ether (1 : 10 v/v) as an eluent to give the corresponding product **3l** (14.8 mg, 54%) as a brown solid. ^{1}H NMR (400 MHz, $CDCl_3$): δ 7.59 (t, *J* = 7.7 Hz, 1H), 7.32 (d, *J* = 7.7 Hz, 1H), 7.03 (d, *J* = 7.7 Hz, 1H), 4.97 (s, 2H), 3.99 (tt, *J* = 11.3, 4.2 Hz, 1H), 2.53 (s, 3H), 1.84 (dd, *J* = 12.6, 4.1 Hz, 2H), 1.53 (t, *J* = 11.8 Hz, 2H), 1.24 (d, *J* = 5.2 Hz, 12H)

ppm. – ^{13}C NMR (100 MHz, $CDCl_3$): δ 157.9, 157.6, 136.9, 121.7, 117.8, 79.8, 63.3, 60.4, 58.5, 48.4, 33.1, 24.4, 21.3, 18.5 ppm. – HRMS: calcd for $C_{16}H_{27}N_2O_2$ 279.1994, found 279.2066 [M+H$^+$].

2,2,6,6-Tetramethyl-1-(thiophen-2-ylmethoxy)piperidine (3m)

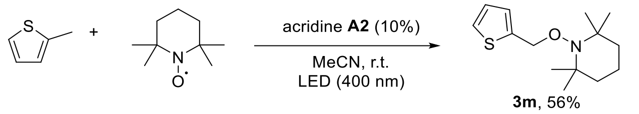

The general procedure **GP1** was followed with TEMPO (31.3 mg, 0.2 mmol), acridine **A2** (4.7 mg, 10 mol%, 0.01 mmol), 2-methylthiophene (39.3mg, 0.4 mmol) and acetonitrile (2 mL). The tube was capped with a screw cap and wrapped with parafilm then irradiated with LED light (λ_{max} = 400 nm) at room temperature for 24 hours. The reaction mixture was concentrated under reduced pressure and purified by flash chromatography on silica gel with EtOAc/petroleum ether (1 : 10 v/v) as an eluent to give the corresponding product **3m** (14.2 mg, 56%) as a yellow solid.

^1H NMR (400 MHz, $CDCl_3$): δ 7.27 (s, 1H), 6.97 (d, *J* = 3.4 Hz, 2H), 4.94 (s, 2H), 1.61 – 1.45 (m, 5H), 1.34 (dt, *J* = 13.4, 3.6 Hz, 1H), 1.26 (s, 6H), 1.13 (s, 6H) ppm. – ^{13}C NMR (100 MHz, $CDCl_3$): δ 140.5, 126.4, 125.4, 125.1, 73.7, 60.0, 39.7, 33.1, 20.2, 17.1 ppm. – HRMS: calcd for $C_{14}H_{24}NOS$ 254.1500, found 254.1575 [M+H$^+$].

1-((1,3-Dihydroisobenzofuran-1-yl)oxy)-2,2,6,6-tetramethylpiperidin-4-ol (3n-α) and 1-((2,3-dihydrobenzofuran-2-yl)oxy)-2,2,6,6-tetramethylpiperidin-4-ol (3n-β)

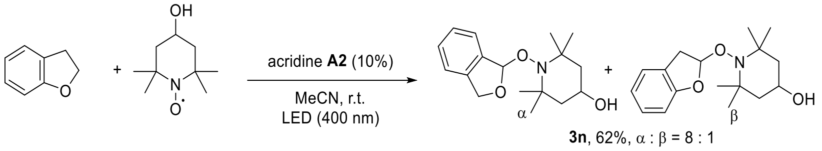

The general procedure **GP1** was followed with 4-OH-TEMPO (34.4 mg, 0.2 mmol), acridine **A2** (4.7 mg, 10 mol%, 0.01 mmol), 2,3-dihydrobenzofuran (0.048 mg, 0.4 mmol) and acetonitrile (2 mL). The tube was capped with a screw cap and wrapped with parafilm then irradiated with LED light (λ_{max} = 400 nm) at room temperature for 24 hours. The reaction mixture was concentrated under reduced pressure and purified by flash chromatography on silica gel with EtOAc/petroleum ether (1 : 10 v/v) as an eluent to give the corresponding product **3n** (18.1 mg, 62%) as a brown solid.

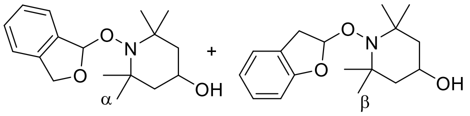

^1H NMR (400 MHz, $CDCl_3$): δ 7.52 (dd, *J* = 7.5, 1.4 Hz, 1H), 7.28 – 7.21 (m, 1H),7.19 (d, *J* = 7.5, 1.4 Hz, 0.12H), 7.13 (t, *J* = 7.5, 1.4 Hz, 0.12H), 6.90 (t, *J* = 7.4 Hz, 1.12H), 6.85 (d, *J* = 8.1 Hz, 1H), 6.80 (d, *J* = 7.5, 1.4 Hz, 0.12H), 5.98 (dd, *J* = 7.5, 1.4 Hz, 0.12H), 5.52 (dd, *J* = 6.5, 2.7 Hz, 1H), 4.78 (dd, *J* = 10.5, 2.8 Hz, 1H), 4.40 (dd, *J* = 10.6, 6.5 Hz, 1H), 3.96 (m, 1.12H), 3.41 (dd, *J* = 7.5, 1.4 Hz, 0.12H), 3.11 (dd, *J* = 7.5, 1.4 Hz, 0.12H) 1.80 (m, 2.24H), 1.58 – 0.83 (m, 15.68H). – ^{13}C NMR (100 MHz, $CDCl_3$): δ 161.1, 158.1, 130.5, 128.0, 127.6, 126.5, 125.4, 124.6, 120.6, 120.1, 111.2, 110.3, 109.6, 82.9, 76.4, 63.2, 60.9, 60.5, 60.3, 48.8, 48.7, 48.2, 35.6, 34.3, 34.0, 33.5, 33.3, 29.7, 21.4, 21.4, 21.2 ppm. – HRMS: calcd for $C_{17}H_{26}NO_3$ 292.1834, found 292.1911 [M+H$^+$].

1-(*tert*-Butoxymethoxy)-2,2,6,6-tetramethylpiperidin-4-ol (3o)

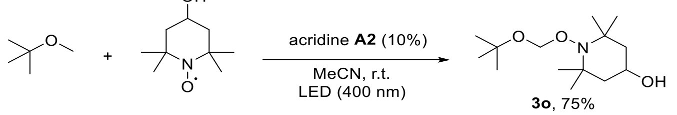

The general procedure **GP1** was followed with 4-OH-TEMPO (34.4 mg, 0.2 mmol), acridine **A2** (4.7 mg, 10 mol%, 0.01 mmol), 2-methoxy-2-methylpropane (0.048 mL, 0.4 mmol) and acetonitrile (2 mL). The tube was capped with a screw cap and wrapped with parafilm then irradiated with LED light (λ_{max} = 400 nm) at room temperature for 24 hours. The reaction mixture was concentrated under reduced pressure and purified by flash chromatography on silica gel with

EtOAc/petroleum ether (1 : 10 v/v) as an eluent to give the corresponding product **3o** (19.5 mg, 75%) as a yellow solid. ^{1}H NMR (400 MHz, $CDCl_3$): δ 4.92 (s, 2H), 3.95 (tt, *J* = 10.9, 3.5 Hz, 1H), 1.82 (dd, *J* = 12.1, 4.0 Hz, 2H), 1.49 – 1.36 (m, 3H), 1.24 (s, 9H), 1.19 (s, 6H), 1.12 (s, 6H) ppm. – ^{13}C NMR (100 MHz, $CDCl_3$): δ 96.6, 74.4, 63.5, 59.6, 48.3, 33.5, 28.8, 21.2 ppm. – HRMS: calcd for $C_{14}H_{30}NO_3$ 260.2147, found 260.2220 [M+H$^+$].

1-((1,4-Dioxan-2-yl)oxy)-2,2,6,6-tetramethylpiperidin-4-ol (3p)

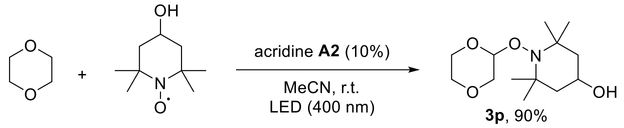

The general procedure **GP1** was followed with 4-*OH*-TEMPO (34.4 mg, 0.2 mmol), acridine **A2** (4.7 mg, 10 mol%, 0.01 mmol), 1,4-dioxane (0.034 mL, 0.4 mmol) and acetonitrile (2 mL). The tube was capped with a screw cap and wrapped with parafilm then irradiated with LED light (λ_{max} = 400 nm) at room temperature for 24 hours. The reaction mixture was concentrated under reduced pressure and purified by flash chromatography on silica gel with EtOAc/petroleum ether (1 : 10 v/v) as an eluent to give the corresponding product **3p** (23.4 mg, 90%) as a brown solid.
^{1}H NMR (400 MHz, $CDCl_3$): δ 4.83 (d, *J* = 4.9 Hz, 1H), 4.09 – 3.93 (m, 2H), 3.85 (dd, *J* = 11.4, 2.6 Hz, 1H), 3.65 (m, 3H), 3.55 (dd, *J* = 11.5, 5.7 Hz, 1H), 1.92 – 1.78 (m, 2H), 1.61 – 1.41 (m, 3H), 1.30 (s, 3H), 1.19 (s, 6H), 1.16 (s, 3H) ppm. – ^{13}C NMR (100 MHz, $CDCl_3$): δ 101.5, 68.0, 65.9, 63.3, 63.1, 61.0, 59.9, 48.9, 48.4, 33.9, 33.4, 21.6, 21.0 ppm. – HRMS: calcd for $C_{13}H_{26}NO_4$ 260.1784, found 260.1858 [M+H$^+$].

2,2,6,6-Tetramethyl-1-((tetrahydrofuran-2-yl)oxy)piperidin-4-one (3q)

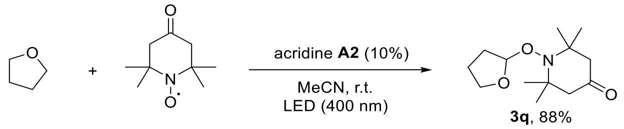

The general procedure **GP1** was followed with 4-Oxo-TEMPO (34.0 mg, 0.2 mmol), acridine **A2** (4.7 mg, 10 mol%, 0.01 mmol), tetrahydrofuran (0.033ml, 0.4 mmol) and acetonitrile (2 mL). The tube was capped with a screw cap and wrapped with parafilm then irradiated with LED light (λ_{max} = 400 nm) at room temperature for 24 hours. The reaction mixture was concentrated under reduced pressure and purified by flash chromatography on silica gel with EtOAc/petroleum ether (1 : 30 v/v) as an eluent to give the corresponding product **3q** (21.2 mg, 88%) as a brown solid.
^{1}H NMR (400 MHz, $CDCl_3$): δ 5.45 (dd, *J* = 5.4, 1.8 Hz, 1H), 3.88 (m, 2H), 2.61 (dd, *J* = 12.9, 4.2 Hz, 2H), 2.20 (m, 2H), 2.09 – 1.90 (m, 3H), 1.82 (m, 1H), 1.35 (s, 3H), 1.25 (s, 3H), 1.11 (d, J = 9.0 Hz, 6H) ppm. – ^{13}C NMR (100 MHz, $CDCl_3$): δ 208.4, 109.8, 67.1, 53.7, 53.2, 33.6, 32.9, 31.3, 23.9, 22.8, 22.1 ppm. – HRMS: calcd for $C_{13}H_{24}NO_3$ 242.1678, found 242.1753 [M+H$^+$].

2,2,6,6-Tetramethyl-1-((tetrahydrothiophen-2-yl)oxy)piperidin-4-one (3r)

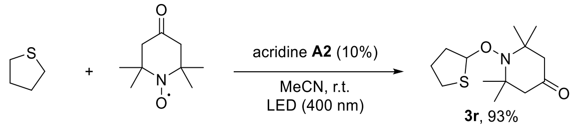

The general procedure **GP1** was followed with 4-Oxo-TEMPO (34.0 mg, 0.2 mmol), acridine **A2** (4.7 mg, 10 mol%, 0.01 mmol), tetrahydrothiophene (0.035 mL, 0.4 mmol) and acetonitrile (2 mL). The tube was capped with a screw cap and wrapped with parafilm then irradiated with LED light (λ_{max} = 400 nm) at room temperature for 24 hours. The reaction mixture was concentrated under reduced pressure and purified by flash chromatography on silica gel with EtOAc/petroleum ether (1 : 30 v/v) as an eluent to give the corresponding product **3r** (23.9 mg, 93%) as a brown solid.
^{1}H NMR (400 MHz, $CDCl_3$): δ 5.67 (t, *J* = 4.3 Hz, 1H), 2.96 (dt, *J* = 10.1, 5.1 Hz, 1H), 2.66 (dt, *J* = 10.0, 7.0 Hz, 1H), 2.51 (d, *J* = 13.4 Hz, 2H), 2.28 – 1.90 (m, 6H), 1.36 (s, 3H), 1.27 (s, 3H), 1.08 (s, 6H) ppm. – ^{13}C NMR (100 MHz, $CDCl_3$): δ

208.1, 97.9, 62.9, 53.8, 53.6, 38.3, 34.6, 33.9, 32.4, 29.0, 22.7, 22.5 ppm. – HRMS: calcd for $C_{13}H_{24}NO_2S$ 258.1449, found 258.1345 [M+H$^+$].

1-((2,2,6,6-Tetramethyl-4-oxopiperidin-1-yl)oxy)ethyl acetate (3s-α) and 2-((2,2,6,6-Tetramethyl-4-oxopiperidin-1-yl)oxy)ethyl acetate (3s-β)

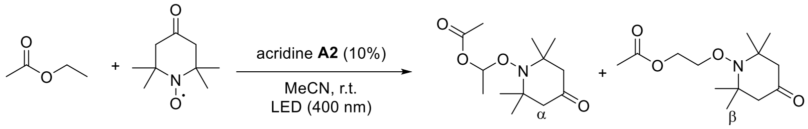

3s, 69%, α : β = 4 : 1

The general procedure **GP1** was followed with 4-Oxo-TEMPO (34.0 mg, 0.2 mmol), acridine **A2** (4.7 mg, 10 mol%, 0.01 mmol) and ethyl acetate (2 mL). The tube was capped with a screw cap and wrapped with parafilm then irradiated with LED light (λ_{max} = 400 nm) at room temperature for 24 hours. The reaction mixture was concentrated under reduced pressure and purified by flash chromatography on silica gel with EtOAc/petroleum ether (1 : 30 v/v) as an eluent to give the corresponding product **3s** (17.8 mg, 69%) as a brown oil.

^{1}H NMR (400 MHz, $CDCl_3$): δ 6.25 (q, *J* = 5.5 Hz, 1H), 4.26 – 4.20 (m, 0.52H), 4.08 – 4.02 (m, 0.52H), 2.65 – 2.51 (m, 2.52H), 2.19 (m, 2.52H), 2.08 (s, 0.78H), 2.05 (s, 3H), 1.41 (d, *J* = 5.5 Hz, 3H), 1.30 (d, *J* = 4.1 Hz, 7.56H), 1.19 – 1.10 (m, 7.56H) ppm. – ^{13}C NMR (100 MHz, $CDCl_{3f}$): δ 207.8, 170.1, 100.0, 74.9, 63.8, 62.7, 62.5, 53.8, 53.4, 53.4, 33.6, 33.0, 32.5, 29.8, 22.7, 22.5, 22.1, 21.6, 21.0, 19.2 ppm. – HRMS: calcd for $C_{13}H_{24}NO_4$ 258.1627, found 258.1524 [M+H$^+$].

1-(1-Ethoxyethoxy)-2,2,6,6-tetramethylpiperidin-4-ol (3t)

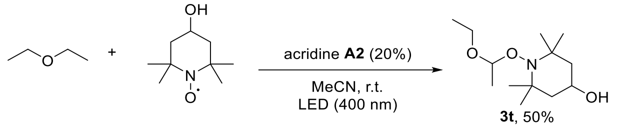

3t, 50%

The general procedure **GP1** was followed with 4-OH-TEMPO (34.4 mg, 0.2 mmol), acridine **A2** (9.4 mg, 20 mol%, 0.02 mmol), ethoxyethane (0.22 mL, 2 mmol) and acetonitrile (2 mL). The tube was capped with a screw cap and wrapped with parafilm then irradiated with LED light (λ_{max} = 400 nm) at room temperature for 24 hours. The reaction mixture was concentrated under reduced pressure and purified by flash chromatography on silica gel with EtOAc/petroleum ether (1 : 10 v/v) as an eluent to give the corresponding product **3t** (12.3 mg, 50%) as a yellow oil.

^{1}H NMR (400 MHz, $CDCl_3$): δ 4.80 (q, J = 5.5 Hz, 1H), 3.89 (tt, J = 11.5, 4.3 Hz, 1H), 3.69 (dq, J = 9.1, 7.1 Hz, 1H), 3.50 (dq, J = 9.2, 7.0 Hz, 1H), 1.76 (ddt, J = 21.6, 12.3, 3.8 Hz, 2H), 1.38 (td, J = 11.9, 5.3 Hz, 2H), 1.27 – 1.19 (m, 6H), 1.12 – 1.05 (m, 12H) ppm. – ^{13}C NMR (100 MHz, $CDCl_3$): δ 105.0, 63.3, 63.0, 60.8, 59.6, 49.0, 48.5, 33.8, 33.7, 21.4, 20.9, 19.3, 15.3 ppm. – HRMS: calcd for $C_{17}H_{29}N$ 247.2220, found 247.2216 [M+H$^+$].

2,2,6,6-Tetramethyl-1-((phenylthio)methoxy)piperidin-4-one (3u)

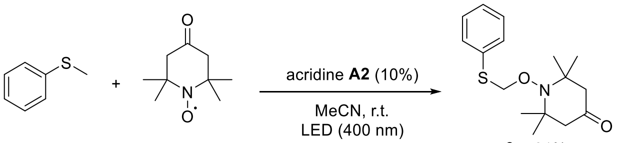

3u, 81%

The general procedure **GP1** was followed with 4-Oxo-TEMPO (34.0 mg, 0.2 mmol), acridine **A2** (4.7 mg, 10 mol%, 0.01 mmol), methyl(phenyl)sulfane (49.7 mg, 0.4 mmol) and acetonitrile (2 mL). The tube was capped with a screw cap and wrapped with parafilm then irradiated with LED light (λ_{max} = 400 nm) at room temperature for 24 hours. The reaction mixture was concentrated under reduced pressure and purified by flash chromatography on silica gel with EtOAc/petroleum ether (1 : 50 v/v) as an eluent to give the corresponding product **3u** (23.8 mg, 81%) as a yellow oil.

^{1}H NMR (400 MHz, $CDCl_3$): δ 7.59 – 7.51 (m, 2H), 7.37 – 7.25 (m, 3H), 5.15 (s, 2H), 2.58 (d, *J* = 12.8 Hz, 2H), 2.22 (d, *J* =

12.8 Hz, 2H), 1.27 (s, 6H), 1.18 (s, 6H) ppm. – ^{13}C NMR (100 MHz, $CDCl_3$): δ 207.8, 135.5, 131.5, 129.0, 127.4, 81.0, 63.2, 53.4, 32.8, 22.5 ppm. – HRMS: calcd for $C_{16}H_{24}NO_2S$ 294.1449, found 294.1346 [M+H$^+$].

1-(((4-Fluorophenyl)thio)methoxy)-2,2,6,6-tetramethylpiperidin-4-one (3v)

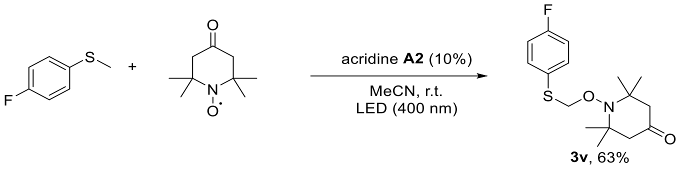

The general procedure **GP1** was followed with 4-Oxo-TEMPO (34.0 mg, 0.2 mmol), acridine **A2** (4.7 mg, 10 mol%, 0.01 mmol), (4-fluorophenyl)(methyl)sulfane (56.4 mg, 0.4 mmol) and acetonitrile (2 mL). The tube was capped with a screw cap and wrapped with parafilm then irradiated with LED light (λ_{max} = 400 nm) at room temperature for 24 hours. The reaction mixture was concentrated under reduced pressure and purified by flash chromatography on silica gel with EtOAc/petroleum ether (1 : 50 v/v) as an eluent to give the corresponding product **3v** (19.6 mg, 63%) as a yellow oil.

^{1}H NMR (400 MHz, $CDCl_3$): δ 7.52 – 7.41 (m, 2H), 6.95 (m, 2H), 4.98 (s, 2H), 2.48 (d, *J* = 12.9 Hz, 2H), 2.12 (d, *J* = 12.9 Hz, 2H), 1.14 (s, 6H), 1.08 (s, 6H) ppm. – ^{13}C NMR (100 MHz, $CDCl_3$): δ 207.7, 164.0, 161.5, 134.6, 134.6, 130.3, 130.3, 116.2, 116.0, 81.4, 63.3, 53.4, 32.7, 22.5 ppm. – ^{19}F NMR (376 MHz, $CDCl_3$) δ -114.0 ppm. – HRMS: calcd for $C_{16}H_{23}FNO_2S$ 312.1335, found 312.1255 [M+H$^+$].

1-(((4-Chlorophenyl)thio)methoxy)-2,2,6,6-tetramethylpiperidin-4-one (3w)

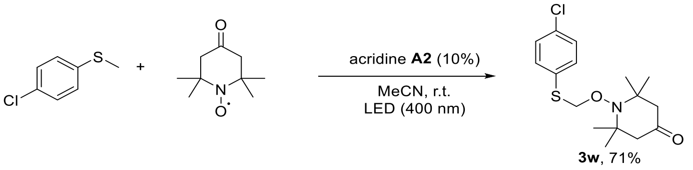

The general procedure **GP1** was followed with 4-*Oxo*-TEMPO (34.0 mg, 0.2 mmol), acridine **A2** (4.7 mg, 10 mol%, 0.01 mmol), (4-chlorophenyl)(methyl)sulfane (63.5 mg, 0.4 mmol) and acetonitrile (2 mL). The tube was capped with a screw cap and wrapped with parafilm then irradiated with LED light (λ_{max} = 400 nm) at room temperature for 24 hours. The reaction mixture was concentrated under reduced pressure and purified by flash chromatography on silica gel with EtOAc/petroleum ether (1 : 50 v/v) as an eluent to give the corresponding product **3w** (23.3 mg, 71%) as a yellow oil.

^{1}H NMR (400 MHz, $CDCl_3$): δ 7.46 (d, *J* = 8.6 Hz, 2H), 7.28 (d, *J* = 8.6 Hz, 2H), 5.10 (s, 2H), 2.55 (d, *J* = 12.9 Hz, 2H), 2.19 (d, *J* = 12.9 Hz, 2H), 1.25 (s, 6H), 1.15 (s, 6H) ppm. – ^{13}C NMR (100 MHz, $CDCl_3$): δ 207.6, 134.0, 133.6, 132.9, 129.2, 81.0, 63.3, 53.4, 32.9, 22.5 ppm. – HRMS: calcd for $C_{16}H_{23}ClNO_2S$ 328.1060, found 328.0957 [M+H$^+$].

1-(((4-Bromophenyl)thio)methoxy)-2,2,6,6-tetramethylpiperidin-4-one (3x)

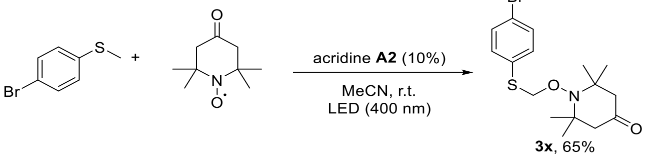

The general procedure **GP1** was followed with 4-*Oxo*-TEMPO (34.0 mg, 0.2 mmol), acridine **A2** (4.7 mg, 10 mol%, 0.01 mmol), (4-bromophenyl)(methyl)sulfane (81.3mg, 0.4 mmol) and acetonitrile (2 mL). The tube was capped with a screw cap and wrapped with parafilm then irradiated with LED light (λ_{max} = 400 nm) at room temperature for 24 hours. The reaction mixture was concentrated under reduced pressure and purified by flash chromatography on silica gel with EtOAc/petroleum ether (1 : 50 v/v) as an eluent to give the corresponding product **3x** (24.2 mg, 65%) as a

brown solid.
^{1}H NMR (400 MHz, $CDCl_3$): δ 7.42 (m, 4H), 5.12 (s, 2H), 2.57 (d, *J* = 12.9 Hz, 2H), 2.20 (d, *J* = 12.9 Hz, 2H), 1.26 (s, 6H), 1.16 (s, 6H) ppm. – ^{13}C NMR (100 MHz, $CDCl_3$): δ 207.5, 134.7, 133.0, 132.1, 121.6, 80.9, 63.4, 58.6, 53.4, 32.8, 22.5 ppm. – HRMS: calcd for $C_{16}H_{23}BrNO_2S$ 372.0555, found 372.0458 [M+H$^+$].

2,2,6,6-Tetramethyl-1-(((4-(4,4,5,5-tetramethyl-1,3,2-dioxaborolan-2-yl)phenyl)thio)methoxy)piperidin-4-one (3y)

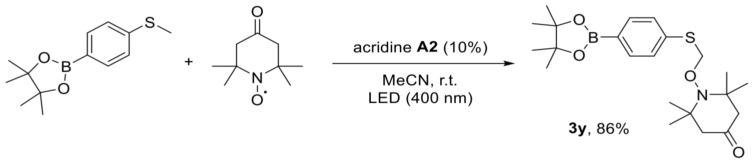

The general procedure **GP1** was followed with 4-Oxo-TEMPO (34.0 mg, 0.2 mmol), acridine **A2** (4.7 mg, 10 mol%, 0.01 mmol), 4,4,5,5-tetramethyl-2-(4-(methylthio)phenyl)-1,3,2-dioxaborolane (100 mg, 0.4 mmol) and acetonitrile (2 mL). The tube was capped with a screw cap and wrapped with parafilm then irradiated with LED light (λ_{max} = 400 nm) at room temperature for 24 hours. The reaction mixture was concentrated under reduced pressure and purified by flash chromatography on silica gel with EtOAc/petroleum ether (1 : 50 v/v) as an eluent to give the corresponding product **3y** (36.1 mg, 86%) as a brown oil.
^{1}H NMR (400 MHz, $CDCl_3$): δ 7.74 (d, *J* = 8.2 Hz, 2H), 7.49 (d, *J* = 8.2 Hz, 2H), 5.19 (s, 2H), 2.58 (d, *J* = 12.9 Hz, 2H), 2.21 (d, *J* = 12.9 Hz, 2H), 1.34 (s, 12H), 1.30 (s, 6H), 1.17 (s, 6H) ppm. – ^{13}C NMR (100 MHz, $CDCl_3$): δ 207.7, 139.7, 135.3, 129.3, 84.0, 80.4, 63.3, 53.4, 32.9, 25.0, 22.5 ppm. – HRMS: calcd for $C_{22}H_{35}BNO_4S$ 420.2302, found 420.2197 [M+H$^+$].

2,2,6,6-Tetramethyl-1-((p-tolylthio)methoxy)piperidin-4-one (3z)

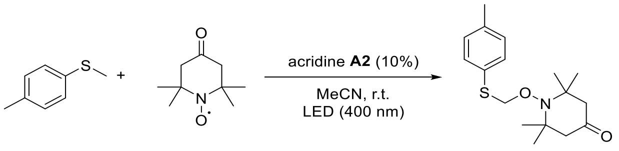

The general procedure **GP1** was followed with 4-Oxo-TEMPO (34.0 mg, 0.2 mmol), acridine **A2** (4.7 mg, 10 mol%, 0.01 mmol), methyl(p-tolyl)sulfane (55.3mg, 0.4 mmol) and acetonitrile (2 mL). The tube was capped with a screw cap and wrapped with parafilm then irradiated with LED light (λ_{max} = 400 nm) at room temperature for 24 hours. The reaction mixture was concentrated under reduced pressure and purified by flash chromatography on silica gel with EtOAc/petroleum ether (1 : 50 v/v) as an eluent to give the corresponding product **3z** (25.5 mg, 83%) as a yellow solid.
^{1}H NMR (400 MHz, $CDCl_3$): δ7.36 (d, *J* = 8.0 Hz, 2H), 7.05 (d, *J* = 8 Hz, 2H), 5.01 (s, 2H), 2.48 (d, *J* = 12.9 Hz, 2H), 2.26 (s, 3H), 2.12 (d, *J* = 12.9 Hz, 2H), 1.16 (s, 6H), 1.09 (s, 6H) ppm. – ^{13}C NMR (100 MHz, $CDCl_3$): δ 207.9, 137.6, 132.1, 131.8, 129.8, 81.4, 63.3, 53.4, 32.8, 22.6, 21.2 ppm. – HRMS: calcd for $C_{17}H_{26}NO_2S$ 308.1606, found 308.1501 [M+H$^+$].

1-(((4-Hydroxyphenyl)thio)methoxy)-2,2,6,6-tetramethylpiperidin-4-one (3aa)

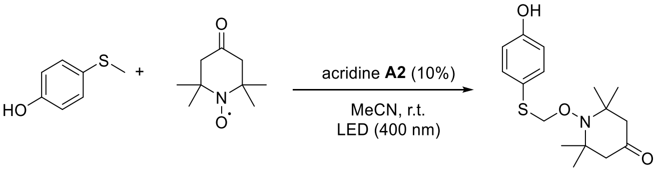

The general procedure **GP1** was followed with 4-Oxo-TEMPO (34.0 mg, 0.2 mmol), acridine **A2** (4.7 mg, 10 mol%, 0.01 mmol), 4-(methylthio)phenol (56.0 mg, 0.4 mmol) and acetonitrile (2 mL). The tube was capped with a screw cap and

wrapped with parafilm then irradiated with LED light (λ_{max} = 400 nm) at room temperature for 24 hours. The reaction mixture was concentrated under reduced pressure and purified by flash chromatography on silica gel with EtOAc/petroleum ether (1 : 15 v/v) as an eluent to give the corresponding product **3aa** (19.2 mg, 62%) as a white solid. ^{1}H NMR (400 MHz, $CDCl_3$): δ7.53 – 7.41 (m, 2H), 6.80 (d, *J* = 7.9 Hz, 2H), 4.99 (s, 2H), 2.55 (d, *J* = 12.9 Hz, 2H), 2.20 (d, *J* = 12.9 Hz, 2H), 1.17 (s, 6H), 1.14 (s, 6H) ppm. – ^{13}C NMR (100 MHz, $CDCl_3$): δ 208.8, 156.0, 135.2, 125.2, 116.2, 81.7, 63.3, 53.2, 32.5, 22.5 ppm. – HRMS: calcd for $C_{16}H_{24}NO_3S$ 310.1399, found 310.1293 [M+H$^+$].

1-(((4-Methoxyphenyl)thio)methoxy)-2,2,6,6-tetramethylpiperidin-4-one (3ab-α) and 2,2,6,6-tetramethyl-1-((4-(methylthio)phenoxy)methoxy)piperidin-4-one (3ab-β)

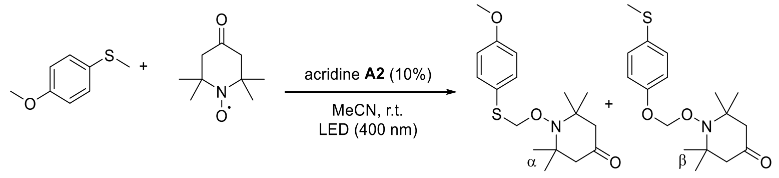

3ab, 76%, ,α:β = 33:1

The general procedure **GP1** was followed with 4-Oxo-TEMPO (34.0 mg, 0.2 mmol), acridine **A2** (4.7 mg, 10 mol%, 0.01 mmol), (4-methoxyphenyl)(methyl)sulfane (61.7 mg, 0.4 mmol) and acetonitrile (2 mL). The tube was capped with a screw cap and wrapped with parafilm then irradiated with LED light (λ_{max} = 400 nm) at room temperature for 24 hours. The reaction mixture was concentrated under reduced pressure and purified by flash chromatography on silica gel with EtOAc/petroleum ether (1 : 20 v/v) as an eluent to give the corresponding product **3ab** (24.6 mg, 76%) as a yellow oil.

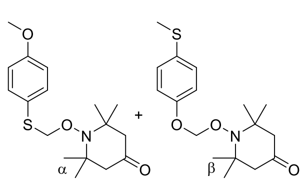

^{1}H NMR (400 MHz, $CDCl_3$): δ 7.50 (d, *J* = 8.8 Hz, 2H), 7.24 (s, 0.06H), 6.96 (d, *J* = 8.8 Hz, 0.06H), 6.85 (d, *J* = 8.8 Hz, 2H), 5.44 (s, 0.06H), 5.00 (s, 2H), 3.80 (s, 3H), 2.63 (d, *J* = 12.9 Hz, 0.06H), 2.53 (d, *J* = 12.9 Hz, 2H), 2.45 (s, 0.06H), 2.24 (s, 0.06H), 2.17 (d, *J* = 12.8 Hz, 2H), 1.29 (d, *J* = 12.8 Hz, 0.36H), 1.17 (s, 6H) , 1.14 (s, 6H) ppm. – ^{13}C NMR (100 MHz, $CDCl_3$): δ 207.9, 159.8, 134.9, 129.8, 125.5, 116.9, 114.5, 81.9, 63.3, 55.5, 53.4, 32.6, 22.6 ppm. – HRMS: calcd for $C_{17}H_{26}NO_3S$ 324.1555, found 324.1450 [M+H$^+$].

Methyl 2-((((2,2,6,6-tetramethyl-4-oxopiperidin-1-yl)oxy)methyl)thio)benzoate (3ac)

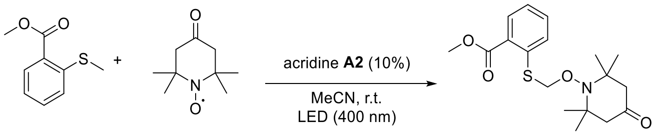

3ac, 72%

The general procedure **GP1** was followed with 4-Oxo-TEMPO (34.0 mg, 0.2 mmol), acridine **A2** (4.7 mg, 10 mol%, 0.01 mmol), methyl 2-(methylthio)benzoate (72.8 mg, 0.4 mmol) and acetonitrile (2 mL). The tube was capped with a screw cap and wrapped with parafilm then irradiated with LED light (λ_{max} = 400 nm) at room temperature for 24 hours. The reaction mixture was concentrated under reduced pressure and purified by flash chromatography on silica gel with EtOAc/petroleum ether (1 : 15 v/v) as an eluent to give the corresponding product **3ac** (25.3 mg, 72%) as a brown solid. ^{1}H NMR (400 MHz, $CDCl_3$): δ 7.86 (dd, *J* = 7.8, 1.6 Hz, 1H), 7.65 (dd, *J* = 8.2, 1.1 Hz, 1H), 7.41 (td, *J* = 7.7, 1.6 Hz, 1H), 7.19 – 7.13 (m, 1H), 5.19 (s, 2H), 3.85 (s, 3H), 2.54 (d, *J* = 12.9 Hz, 2H), 2.16 (d, *J* = 12.9 Hz, 2H), 1.33 (s, 6H), 1.12 (s, 6H) ppm. – ^{13}C NMR (100 MHz, $CDCl_3$): δ 207.6, 167.1, 140.8, 132.7, 131.0, 128.7, 128.4, 125.3, 79.8, 63.2, 53.4, 52.3, 33.1, 22.4 ppm. – HRMS: calcd for $C_{18}H_{26}NO_4S$ 352.1504, found 352.1402 [M+H$^+$].

Go to table of contents

2,2,6,6-Tetramethyl-1-(((3-(4,4,5,5-tetramethyl-1,3,2-dioxaborolan-2-yl)phenyl)thio)methoxy)piperidin-4-one (3ad)

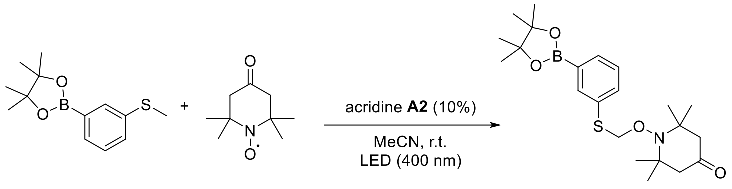

3ad, 73%

The general procedure **GP1** was followed with 4-Oxo-TEMPO (34.0 mg, 0.2 mmol), acridine **A2** (4.7 mg, 10 mol%, 0.01 mmol), 4,4,5,5-tetramethyl-2-(3-(methylthio)phenyl)-1,3,2-dioxaborolane (100 mg, 0.4 mmol) and acetonitrile (2 mL). The tube was capped with a screw cap and wrapped with parafilm then irradiated with LED light (λ_{max} = 400 nm) at room temperature for 24 hours. The reaction mixture was concentrated under reduced pressure and purified by flash chromatography on silica gel with EtOAc/petroleum ether (1 : 15 v/v) as an eluent to give the corresponding product **3ad** (30.6 mg, 73%) as a yellow solid.

^{1}H NMR (400 MHz, $CDCl_3$): δ 8.07 – 8.01 (m, 1H), 7.67 (dt, *J* = 7.4, 1.2 Hz, 1H), 7.58 (m, 1H), 7.30 (t, *J* = 7.6 Hz, 1H), 5.12 (s, 2H), 2.56 (d, *J* = 12.9 Hz, 2H), 2.21 (d, *J* = 12.9 Hz, 2H), 1.32 (s, 12H), 1.27 (s, 6H), 1.19 (s, 6H) ppm. – ^{13}C NMR (100 MHz, $CDCl_3$): δ 208.0, 137.5, 135.3, 133.7, 133.4, 128.4, 84.1, 80.6, 63.3, 53.4, 32.8, 25.0, 22.7 ppm. – HRMS: calcd for $C_{22}H_{35}BNO_4S$ 420.2302, found 420.2191 [M+H$^+$].

2,2,6,6-Tetramethyl-1-((1,1,2,2,2-pentamethyldisilaneyl)methoxy)piperidine (3ae)

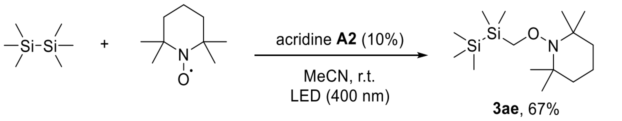

3ae, 67%

The general procedure **GP1** was followed with TEMPO (31.3 mg, 0.2 mmol), acridine **A2** (4.7 mg, 10 mol%, 0.01 mmol), 1,1,1,2,2,2-hexamethyldisilane (73.2 mg, 0.5 mmol) and acetonitrile (2 mL). The tube was capped with a screw cap and wrapped with parafilm then irradiated with LED light (λ_{max} = 400 nm) at room temperature for 24 hours. The reaction mixture was concentrated under reduced pressure and purified by flash chromatography on silica gel with EtOAc/petroleum ether (1 : 100 v/v) as an eluent to give the corresponding product **3ae** (20.2 mg, 67%) as a yellow solid.

^{1}H NMR (400 MHz, $CDCl_3$): δ 3.67 (s, 2H), 1.52 – 1.24 (m, 6H), 1.17 (s, 6H), 1.08 (s, 6H), 0.10 (s, 15H) ppm. – ^{13}C NMR (100 MHz, $CDCl_3$): δ 70.9, 59.8, 39.6, 32.9, 19.9, 16.9, -2.3, -5.7 ppm. – HRMS: calcd for $C_{15}H_{36}NOSi$ 302.2257, found 302.2332 [M+H$^+$].

2,2,6,6-Tetramethylpiperidin-1-yl benzoate (3af)

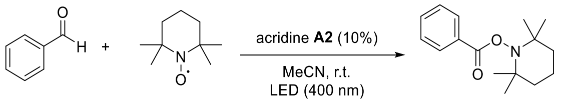

3af, 53%

The general procedure **GP1** was followed with TEMPO (31.3 mg, 0.2 mmol), acridine **A2** (4.7 mg, 10 mol%, 0.01 mmol), benzaldehyde (42.5 mg, 0.4 mmol) and acetonitrile (2 mL). The tube was capped with a screw cap and wrapped with parafilm then irradiated with LED light (λ_{max} = 400 nm) at room temperature for 24 hours. The reaction mixture was concentrated under reduced pressure and purified by flash chromatography on silica gel with EtOAc/petroleum ether (1 : 100 v/v) as an eluent to give the corresponding product **3af** (13.9 mg, 53%) as a yellow solid.

^{1}H NMR (400 MHz, $CDCl_3$): δ 8.12 – 8.03 (m, 2H), 7.61 – 7.54 (m, 1H), 7.46 (t, *J* = 7.7 Hz, 2H), 1.79 (dd, *J* = 14.6, 11.2 Hz, 2H), 1.74 – 1.65 (m, 1H), 1.59 (m, 2H), 1.46 (m , 1H), 1.28 (s, 6H), 1.12 (s, 6H) ppm. – ^{13}C NMR (100 MHz, $CDCl_3$): δ 166.5, 133.0, 129.9, 129.7, 128.6, 60.6, 39.2, 32.1, 21.0, 17.2 ppm. – HRMS: calcd for $C_{16}H_{24}NO_2$ 262.1729, found 262.1625

[M+H+].

1-((1-(4-(*tert*-Butyl)phenyl)propan-2-yl)oxy)-2,2,6,6-tetramethylpiperidin-4-ol (3ag)

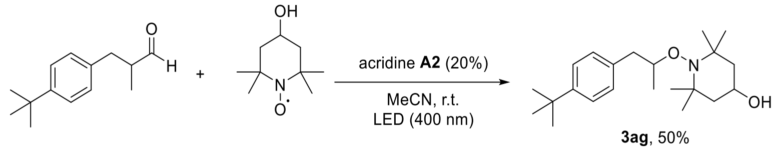

The general procedure **GP1** was followed with 4-OH- TEMPO (34.4 mg, 0.2 mmol), acridine **A2** (9.4 mg, 20 mol%, 0.02 mmol), Lily aldehyde (0.22 ml, 1 mmol) and acetonitrile (2 mL). The tube was capped with a screw cap and wrapped with parafilm then irradiated with LED light (λ_{max} = 400 nm) at room temperature for 24 hours. The reaction mixture was concentrated under reduced pressure and purified by flash chromatography on silica gel with EtOAc/petroleum ether (1 : 10 v/v) as an eluent to give the corresponding product **3ag** (17.4 mg, 50%) as a brown solid.
^{1}H NMR (400 MHz, $CDCl_3$): δ 7.31 – 7.27 (m, 2H), 7.14 – 7.09 (m, 2H), 4.15 – 4.03 (m, 1H), 3.96 (s, 1H), 3.11 (dd, *J* = 13.1, 5.1 Hz, 1H), 2.49 (dd, *J* = 13.1, 8.2 Hz, 1H), 1.79 (m, 2H), 1.47 (t, *J* = 11.8 Hz, 2H), 1.31 (s, 12H), 1.21 – 1.08 (m, 12H) ppm. – ^{13}C NMR (100 MHz, $CDCl_3$): δ 148.7, 136.5, 129.2, 125.0, 79.8, 63.5, 60.4, 59.9, 48.9, 42.3, 34.7, 34.5, 34.3, 31.4, 21.4, 21.3, 19.4 ppm. – HRMS: calcd for $C_{22}H_{38}NO_2$ 348.2824, found 348.2892 [M+H+].

Methyl-3-cyclohexyl-2-(4-(trifluoromethyl)phenyl)propanoate (4a)

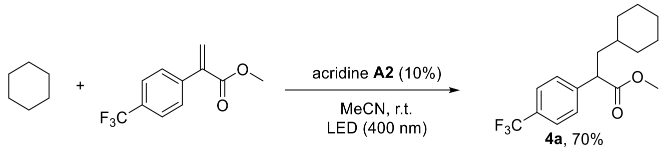

The general procedure **GP2** was followed with methyl-2-(4-(trifluoromethyl)phenyl)acrylate (46.0 mg, 0.2 mmol), acridine **A2** (9.4 mg, 10 mol%, 0.02 mmol), cyclohexane (0.11 ml, 1 mmol) and acetonitrile (2 mL). The tube was capped with a screw cap and wrapped with parafilm then irradiated with LED light (λ_{max} = 400 nm) at room temperature for 24 hours. The reaction mixture was concentrated under reduced pressure and purified by flash chromatography on silica gel with EtOAc/petroleum ether (1 : 50 v/v) as an eluent to give the corresponding product **4a** (44.0 mg, 70%) as a yellow oil.
^{1}H NMR (400 MHz, $CDCl_3$): δ 7.57 (d, *J* = 8.0 Hz, 2H), 7.43 (d, *J* = 8.1 Hz, 2H), 3.76 (t, *J* = 7.8 Hz, 1H), 3.66 (s, 3H), 1.99 (dt, *J* = 13.7, 7.5 Hz, 1H), 1.78 – 1.59 (m, 6H), 1.22 – 1.04 (m, 4H), 0.91 (m, 2H) ppm. – ^{13}C NMR (100 MHz, $CDCl_3$): δ 174.2, 143.6, 130.1, 129.8, 129.4, 129.1, 128.5, 125.7, 125.7, 125.7, 125.6, 122.9, 52.3, 48.8, 41.2, 35.4, 33.4, 33.0, 26.6, 26.2, 26.2 ppm. – ^{19}F NMR (376 MHz, $CDCl_3$) δ -62.5 ppm. – HRMS: calcd for $C_{17}H_{22}F_3O_2$ 315.1494, found 315.1391 [M+H+].

Methyl-3-cyclohexyl-2-(4-fluorophenyl)propanoate (4b)

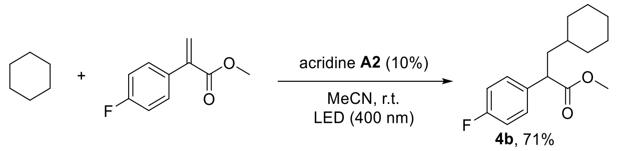

The general procedure **GP2** was followed with methyl-2-(4-fluorophenyl)acrylate (36.0 mg, 0.2 mmol), acridine **A2** (9.4 mg, 10 mol%, 0.02 mmol), cyclohexane (0.11 ml, 1 mmol) and acetonitrile (2 mL). The tube was capped with a screw cap and wrapped with parafilm then irradiated with LED light (λ_{max} = 400 nm) at room temperature for 24 hours. The reaction mixture was concentrated under reduced pressure and purified by flash chromatography on silica gel with EtOAc/petroleum ether (1 : 50 v/v) as an eluent to give the corresponding product **4b** (37.6 mg, 71%) as a yellow oil.
^{1}H NMR (400 MHz, $CDCl_3$): δ 7.26 (m, 2H), 6.99 (t, *J* = 8.6 Hz, 2H), 3.66 (d, *J* = 12.1 Hz, 4H), 2.00 – 1.88 (m, 1H), 1.76 –

1.53 (m, 6H), 1.13 (m, 4H), 0.97 – 0.81 (m, 2H) ppm. – ^{13}C NMR (100 MHz, $CDCl_3$): δ 174.8, 163.3, 160.9, 135.3, 135.2, 129.6, 129.5, 115.6, 115.4, 52.1, 48.1, 41.3, 35.3, 33.4, 33.0, 26.6, 26.2, 26.2 ppm. – ^{19}F NMR (376 MHz, $CDCl_3$) δ -115.7 ppm. – HRMS: calcd for $C_{16}H_{22}FO_2$ 265.1526, found 265.1705 [M+H$^+$].

Triethyl-2-cyclohexylethane-1,1,2-tricarboxylate (4c)

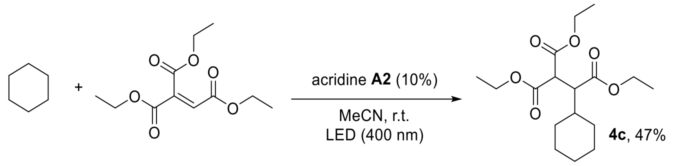

The general procedure **GP2** was followed with triethyl ethene-1,1,2-tricarboxylate (48.4 mg, 0.2 mmol), acridine **A2** (9.4 mg, 10 mol%, 0.02 mmol), cyclohexane (0.086 ml, 0.8 mmol) and acetonitrile (2 mL). The tube was capped with a screw cap and wrapped with parafilm then irradiated with LED light (λ_{max} = 400 nm) at room temperature for 24 hours. The reaction mixture was concentrated under reduced pressure and purified by flash chromatography on silica gel with EtOAc/petroleum ether (1 : 50 v/v) as an eluent to give the corresponding product **4c** (31.0 mg, 47%) as a brown oil.

^1H NMR (400 MHz, $CDCl_3$): δ 4.31 – 4.09 (m, 6H), 3.87 (d, J = 11.3 Hz, 1H), 3.06 (dd, J = 11.3, 3.8 Hz, 1H), 1.81 – 1.67 (m, 4H), 1.67 – 1.58 (m, 1H), 1.45 (m, 1H), 1.34 – 1.16 (m, 11H), 1.14 – 1.01 (m, 2H), 0.91 (m, 1H) ppm. – ^{13}C NMR (100 MHz, $CDCl_3$): δ 172.5, 168.5, 168.4, 61.8, 61.7, 60.6, 52.5, 50.1, 38.6, 32.1, 28.6, 26.9, 26.6, 26.3, 14.4, 14.2, 14.1 ppm. – HRMS: calcd for $C_{17}H_{29}O_6$ 329.1886, found 329.1783 [M+H$^+$].

2,2-Dimethyl-5-(phenyl(tetrahydrothiophen-2-yl)methyl)-1,3-dioxane-4,6-dione (4d)

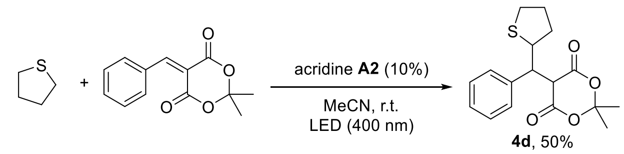

The general procedure **GP2** was followed with 5-benzylidene-2,2-dimethyl-1,3-dioxane-4,6-dione (46.4 mg, 0.2 mmol), acridine **A2** (9.4 mg, 10 mol%, 0.02 mmol), tetrahydrothiophene (0.2 ml, 2 mmol) and acetonitrile (2 mL). The tube was capped with a screw cap and wrapped with parafilm then irradiated with LED light (λ_{max} = 400 nm) at room temperature for 24 hours. The reaction mixture was concentrated under reduced pressure and purified by flash chromatography on silica gel with EtOAc/petroleum ether (1 : 50 v/v) as an eluent to give the corresponding product **4d** (46.4 mg, 50%) as a yellow solid.

^1H NMR (400 MHz, $CDCl_3$): δ 7.30 – 7.19 (m, 5H), 3.89 (d, J = 3.2 Hz, 1H), 3.38 (dd, J = 11.3, 3.2 Hz, 1H), 2.47 – 2.34 (m, 1H), 2.05 (dt, J = 12.1, 3.3 Hz, 1H), 1.84 (m, 1H), 1.72 – 1.63 (m, 1H), 1.60 (s, 3H), 1.47 – 1.37 (m, 2H), 1.03 (s, 3H), 0.79 – 0.67 (m, 1H) ppm. – ^{13}C NMR (100 MHz, $CDCl_3$): δ 166.6, 164.9, 139.8, 129.3, 128.8, 127.6, 105.5, 53.2, 47.9, 37.9, 32.0, 31.2, 28.6, 28.0, 26.3, 26.1, 26.0 ppm. – HRMS: calcd for $C_{17}H_{21}O_4S$ 321.1082, found 321.1159 [M+H$^+$].

3-Cyclohexyl-*N*,2-diphenylpropanamide (4e)

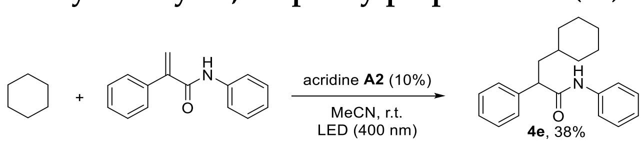

The general procedure **GP2** was followed with *N*,2-diphenylacrylamide (44.7 mg, 0.2 mmol), acridine **A2** (9.4 mg, 10 mol%, 0.02 mmol), cyclohexane (0.24 ml, 2 mmol) and acetonitrile (2 mL). The tube was capped with a screw cap and wrapped with parafilm then irradiated with LED light (λ_{max} = 400 nm) at room temperature for 24 hours. The reaction

mixture was concentrated under reduced pressure and purified by flash chromatography on silica gel with EtOAc/petroleum ether (1 : 50 v/v) as an eluent to give the corresponding product **4e** (23.4 mg, 38%) as a yellow solid. ^{1}H NMR (400 MHz, $CDCl_3$): δ 7.43 (d, *J* = 7.6 Hz, 2H), 7.36 (d, *J* = 4.4 Hz, 4H), 7.32 – 7.22 (m, 2H), 7.13 (s, 1H), 7.06 (t, *J* = 7.4 Hz, 1H), 3.64 (t, *J* = 7.6 Hz, 1H), 2.13 (m, 1H), 1.82 – 1.56 (m, 6H), 1.31 – 1.08 (m, 4H), 0.94 (m, 2H) ppm. – ^{13}C NMR (100 MHz, $CDCl_3$): δ 172.1, 140.1, 138.1, 129.2, 129.0, 128.1, 127.6, 124.3, 119.8, 51.6, 40.9, 35.3, 33.8, 32.9, 26.6, 26.3, 26.2 ppm. – HRMS: calcd for $C_{21}H_{26}NO$ 308.1936, found 308.1839 [M+H^{+}].

2-((4-Phenoxyphenyl)(tetrahydrothiophen-2-yl)methyl)malononitrile (4f)

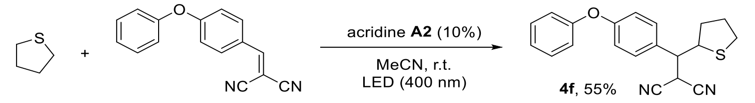

The general procedure **GP1** was followed with 2-(4-phenoxybenzylidene)malononitrile (49.2 mg, 0.2 mmol), acridine **A2** (9.4 mg, 10 mol%, 0.02 mmol), tetrahydrothiophene (0.18 ml, 2 mmol) and acetonitrile (2 mL). The tube was capped with a screw cap and wrapped with parafilm then irradiated with LED light (λ_{max} = 400 nm) at room temperature for 24 hours. The reaction mixture was concentrated under reduced pressure and purified by flash chromatography on silica gel with EtOAc/petroleum ether (1 : 20 v/v) as an eluent to give the corresponding product **4f** (36.8 mg, 55%) as a yellow oil.

a:^{1}H NMR (400 MHz, $CDCl_3$): δ 7.42 – 7.31 (m, 4H), 7.19 – 7.12 (m, 1H), 7.10 – 7.00 (m, 4H), 4.60 (d, *J* = 4.1 Hz, 1H), 3.92 (m, 1H), 3.06 – 2.94 (m, 3H), 2.11 – 1.87 (m, 3H), 1.68 – 1.59 (m, 1H) ppm. – ^{13}C NMR (100 MHz, $CDCl_3$): δ 158.6, 156.3, 130.1, 129.9, 129.8, 124.2, 119.8, 118.9, 112.2, 111.5, 53.4, 50.1, 35.9, 33.3, 29.9, 29.8 ppm. – HRMS: calcd for $C_{20}H_{18}N_2OS$ 334.1140, found 334.1032 [M+H^{+}]. b:^{1}H NMR (400 MHz, $CDCl_3$): δ 7.42 – 7.31 (m, 4H), 7.20 – 7.12 (m, 1H), 7.10 – 6.98 (m, 4H), 4.15 (d, *J* = 6.0 Hz, 1H), 4.08 (m, 1H), 3.32 (dd, *J* = 9.1, 6.1 Hz, 1H), 2.88 – 2.73 (m, 2H), 2.39 – 2.31 (m, 1H), 2.12 – 1.90 (m, 2H), 1.66 (m, 1H) ppm. – ^{13}C NMR (100 MHz, $CDCl_3$): δ 158.4, 156.2, 130.5, 129.9, 129.8, 124.0, 119.7, 118.6, 111.6, 111.5, 51.4, 50.4, 35.5, 32.5, 31.2, 28.8 ppm. – HRMS: calcd for $C_{20}H_{19}N_2OS$ 335.1140, found 335.1039 [M+H^{+}].

2-(4-Methyl-2-oxo-1-phenylpentyl)malononitrile (4g)

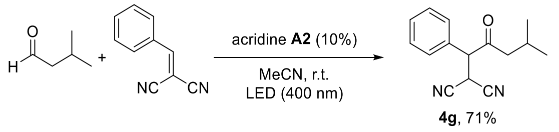

The general procedure **GP2** was followed with 2-benzylidenemalononitrile (30.6 mg, 0.2 mmol), acridine **A2** (9.4 mg, 10 mol%, 0.02 mmol), 3-methylbutanal (0.24 ml, 2 mmol) and acetonitrile (2 mL). The tube was capped with a screw cap and wrapped with parafilm then irradiated with LED light (λ_{max} = 400 nm) at room temperature for 24 hours. The reaction mixture was concentrated under reduced pressure and purified by flash chromatography on silica gel with EtOAc/petroleum ether (1 : 75 v/v) as an eluent to give the corresponding product **4g** (34.2 mg, 71%) as a brown oil.

^{1}H NMR (400 MHz, $CDCl_3$): δ 7.49 – 7.43 (m, 3H), 7.25 (m, 2H), 4.39 (d, *J* = 8.9 Hz, 1H), 4.23 (d, *J* = 8.9 Hz, 1H), 2.38 – 2.11 (m, 3H), 0.90 (d, *J* = 6.6 Hz, 3H), 0.79 (d, *J* = 6.6 Hz, 3H) ppm. – ^{13}C NMR (100 MHz, $CDCl_3$): δ 203.4, 131.0, 130.1, 130.0, 128.7, 112.0, 111.4, 58.7, 49.8, 25.5, 24.6, 22.5, 22.0 ppm. – HRMS: calcd for $C_{15}H_{17}N_2O$ 241.1263, found 241.1158 [M+H^{+}].

***N*-(3,3-Dicyano-2-(4-fluorophenyl)propyl)-N-methylacetamide (4h)**

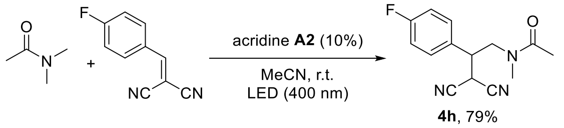

The general procedure **GP2** was followed with 2-(4-fluorobenzylidene)malononitrile (34.4 mg, 0.2 mmol), acridine **A2**

Go to table of contents

(9.4 mg, 10 mol%, 0.02 mmol), *N*,*N*-dimethylacetamide (0.093 ml, 1 mmol) and acetonitrile (2 mL). The tube was capped with a screw cap and wrapped with parafilm then irradiated with LED light (λ_{max} = 400 nm) at room temperature for 24 hours. The reaction mixture was concentrated under reduced pressure and purified by flash chromatography on silica gel with EtOAc/petroleum ether (1 : 25 v/v) as an eluent to give the corresponding product **4h** (41.0 mg, 79%) as a yellow oil.

^{1}H NMR (400 MHz, $CDCl_3$): δ 7.39 – 7.32 (m, 2H), 7.12 (t, *J* = 8.5 Hz, 2H), 4.26 (d, *J* = 6.3 Hz, 1H), 4.19 (dd, *J* = 14.0, 8.8 Hz, 1H), 3.66 (m, 1H), 3.45 (dd, *J* = 14.0, 5.9 Hz, 1H), 2.95 (s, 3H), 2.08 (s, 3H) ppm. – ^{13}C NMR (100 MHz, $CDCl_3$): δ 172.4, 164.3, 161.8, 131.0, 131.0, 129.9, 129.8, 116.6, 116.4, 112.2, 111.6, 51.1, 44.1, 37.7, 27.6, 21.8 ppm. – ^{19}F NMR (376 MHz, $CDCl_3$) δ -110.6 ppm. – HRMS: calcd for $C_{14}H_{15}FN_3O$ 260.1121, found 260.1209 [M+H$^+$].

2-Amino-4-(4-fluorophenyl)-5,5-dimethyl-4,5-dihydrofuran-3-carbonitrile (4j)

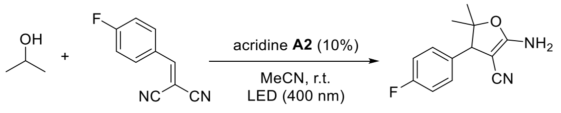

4j, 30%

The general procedure **GP2** was followed with 2-(4-fluorobenzylidene)malononitrile (34.4 mg, 0.2 mmol), acridine **A2** (9.4 mg, 10 mol%, 0.02 mmol), isopropyl alcohol (0.15 ml, 2 mmol) and acetonitrile (2 mL). The tube was capped with a screw cap and wrapped with parafilm then irradiated with LED light (λ_{max} = 400 nm) at room temperature for 24 hours. The reaction mixture was concentrated under reduced pressure and purified by flash chromatography on silica gel with EtOAc/petroleum ether (1 : 35 v/v) as an eluent to give the corresponding product **4j** (14.0 mg, 30%) as a brown solid.

^{1}H NMR (400 MHz, $CDCl_3$): δ 7.15 (dd, *J* = 8.6, 5.4 Hz, 2H), 7.03 (t, *J* = 8.6 Hz, 2H), 4.79 (s, 2H), 4.02 (s, 1H), 1.54 (s, 3H), 0.87 (s, 3H) ppm. – ^{13}C NMR (100 MHz, $CDCl_3$): δ 166.7, 163.5, 161.1, 134.4, 134.4, 129.8, 129.7, 119.3, 115.5, 115.3, 91.4, 91.4, 56.8, 56.1, 29.1, 24.3 ppm. – ^{19}F NMR (376 MHz, $CDCl_3$) δ -115.2 ppm. – HRMS: calcd for $C_{13}H_{14}FN_2O$ 233.1012, found 233.0907 [M+H$^+$].

Di-*tert*-butyl 1-(tetrahydrofuran-2-yl)hydrazine-1,2-dicarboxylate (4k)

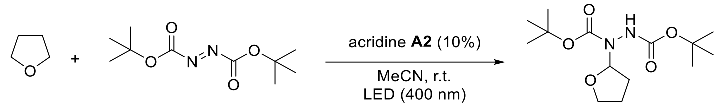

4k, 64%

The general procedure **GP2** was followed with di-*tert*-butyl (*E*)-diazene-1,2-dicarboxylate (46.1 mg, 0.2 mmol), acridine **A2** (9.4 mg, 10 mol%, 0.02 mmol), tetrahydrofuran (0.0334 ml, 0.4 mmol) and acetonitrile (2 mL). The tube was capped with a screw cap and wrapped with parafilm then irradiated with LED light (λ_{max} = 400 nm) at room temperature for 24 hours. The reaction mixture was concentrated under reduced pressure and purified by flash chromatography on silica gel with EtOAc/petroleum ether (1 : 75 v/v) as an eluent to give the corresponding product **4k** (38.8 mg, 64%) as a yellow solid.

^{1}H NMR (400 MHz, $CDCl_3$): δ 6.05 (m, 2H), 3.97 (m, 1H), 3.81 – 3.69 (m, 1H), 2.01 (s, 3H), 1.91 – 1.82 (m, 1H), 1.47 (s, 18H) ppm. – ^{13}C NMR (100 MHz, $CDCl_3$): δ 155.9, 155.8, 154.5, 87.6, 87.1, 82.0, 81.2, 77.5, 68.6, 29.5, 28.3, 25.4 ppm. – HRMS: calcd for $C_{14}H_{27}N_2O_5$ 303.1842, found 303.1741 [M+H$^+$].

1,2-Diphenyl-1-(tetrahydrothiophen-2-yl)hydrazine (4l)

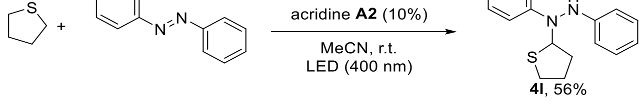

4l, 56%

The general procedure **GP2** was followed with 1,2-diphenyldiazene (36.5 mg, 0.2 mmol), acridine **A2** (9.4 mg, 10 mol%,

0.02 mmol), tetrahydrothiophene (0.035 ml, 0.4 mmol) and acetonitrile (2 mL). The tube was capped with a screw cap and wrapped with parafilm then irradiated with LED light (λ_{max} = 400 nm) at room temperature for 24 hours. The reaction mixture was concentrated under reduced pressure and purified by flash chromatography on silica gel with EtOAc/petroleum ether (1 : 100 v/v) as an eluent to give the corresponding product **4l** (30.2 mg, 56%) as a yellow oil.
^{1}H NMR (400 MHz, $CDCl_3$): δ 7.28 – 7.19 (m, 4H), 7.07 – 6.99 (m, 2H), 6.95 – 6.85 (m, 3H), 6.80 (d, *J* = 1.3 Hz, 1H), 6.01 (t, *J* = 6.2 Hz, 1H), 5.87 (s, 1H), 3.01 (m, 1H), 2.86 (m, 1H), 2.23 – 2.09 (m, 3H), 1.96 – 1.82 (m, 1H) ppm. – ^{13}C NMR (100 MHz, $CDCl_3$): δ 150.4, 149.0, 129.6, 129.4, 121.5, 119.3, 116.2, 111.8, 73.6, 34.2, 33.0, 30.5 ppm. – HRMS: calcd for $C_{16}H_{19}N_2S$ 271.1191, found 271.0883 [M+H$^+$].

1-(Tetrahydrothiophen-2-yl)-1,2-bis(4-(trifluoromethyl)phenyl)hydrazine (4m)

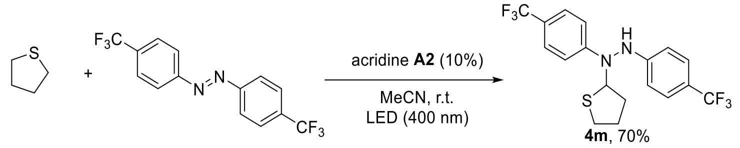

The general procedure **GP2** was followed with 1,2-bis(4-(trifluoromethyl)phenyl)diazene (63.6 mg, 0.2 mmol), acridine **A2** (9.4 mg, 10 mol%, 0.02 mmol), tetrahydrothiophene (0.035 ml, 0.4 mmol) and acetonitrile (2 mL). The tube was capped with a screw cap and wrapped with parafilm then irradiated with LED light (λ_{max} = 400 nm) at room temperature for 24 hours. The reaction mixture was concentrated under reduced pressure and purified by flash chromatography on silica gel with EtOAc/petroleum ether (1 : 50 v/v) as an eluent to give the corresponding product **4m** (56.8 mg, 70%) as a yellow oil.
^{1}H NMR (400 MHz, $CDCl_3$): δ 7.49 (t, *J* = 8.8 Hz, 4H), 7.07 (d, *J* = 8.5 Hz, 2H), 6.89 (d, *J* = 8.4 Hz, 2H), 6.23 (s, 1H), 6.03 (t, *J* = 6.3 Hz, 1H), 3.04 (m, 1H), 2.90 (m, 1H), 2.25 – 1.88 (m, 4H) ppm. – ^{13}C NMR (100 MHz, $CDCl_3$): δ 152.5, 151.0, 127.3, 127.2, 126.9, 126.8, 126.8, 126.8, 126.1, 125.8, 123.9, 123.5, 123.4, 123.1, 121.9, 121.6, 115.7, 111.2, 72.7, 34.2, 33.1, 30.4 ppm. – ^{19}F NMR (376 MHz, $CDCl_3$) δ -61.3, -61.7 ppm. – HRMS: calcd for $C_{18}H_{17}F_6N_2S$ 407.0938, found 407.0869 [M+H$^+$].

Diethyl 1-(tetrahydrothiophen-2-yl)hydrazine-1,2-dicarboxylate (4n)

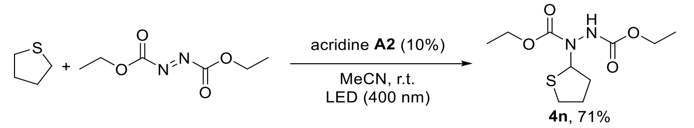

The general procedure **GP2** was followed with diethyl (*E*)-diazene-1,2-dicarboxylate (34.9 mg, 0.2 mmol), acridine **A2** (9.4 mg, 10 mol%, 0.02 mmol), tetrahydrothiophene (0.035 ml, 0.4 mmol) and acetonitrile (2 mL). The tube was capped with a screw cap and wrapped with parafilm then irradiated with LED light (λ_{max} = 400 nm) at room temperature for 24 hours. The reaction mixture was concentrated under reduced pressure and purified by flash chromatography on silica gel with EtOAc/petroleum ether (1 : 100 v/v) as an eluent to give the corresponding product **4n** (37.2 mg, 71%) as a brown oil.
^{1}H NMR (400 MHz, $CDCl_3$): δ 6.55 (s, 1H), 6.11 (s, 1H), 4.29 – 4.08 (m, 4H), 3.01 (dt, *J* = 10.5, 5.5 Hz, 1H), 2.76 (dt, *J* = 10.5, 5.5 Hz, 1H), 2.01 (m, 4H), 1.26 (m, 6H) ppm. – ^{13}C NMR (100 MHz, $CDCl_3$): δ 156.6, 155.4, 67.4, 63.0, 62.2, 34.4, 33.3, 30.2, 14.5, 14.5 ppm. – HRMS: calcd for $C_{10}H_{19}N_2O_4S$ 263.0987, found 263.0886 [M+H$^+$].

Butyl 2-cyclohexyl-2-phenylhydrazine-1-carboxylate (4o)

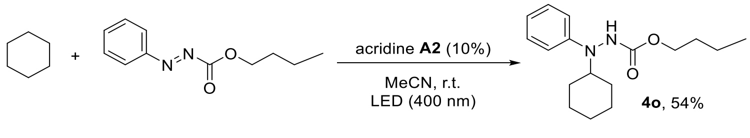

The general procedure **GP2** was followed with diethyl butyl (*E*)-2-phenyldiazene-1-carboxylate (34.9 mg, 0.2 mmol), acridine **A2** (9.4 mg, 10 mol%, 0.02 mmol), cyclohexane (0.06 ml, 0.4 mmol) and acetonitrile (2 mL). The tube was capped with a screw cap and wrapped with parafilm then irradiated with LED light (λ_{max} = 400 nm) at room temperature for 24 hours. The reaction mixture was concentrated under reduced pressure and purified by flash chromatography on silica gel with EtOAc/petroleum ether (1 : 25 v/v) as an eluent to give the corresponding product **4o** (31.4 mg, 54%) as a brown solid.

^{1}H NMR (400 MHz, $CDCl_3$): δ 7.12 (t, *J* = 7.9 Hz, 2H), 6.76 (t, *J* = 7.3 Hz, 1H), 6.70 (d, *J* = 8.0 Hz, 2H), 5.60 (s, 1H), 4.06 – 3.89 (m, 3H), 1.72 (m, 4H), 1.57 – 0.92 (m, 10H), 0.74 (s, 3H) ppm. – ^{13}C NMR (100 MHz, $CDCl_3$): δ 157.3, 149.2, 129.0, 120.4, 113.0, 65.9, 57.9, 31.0, 30.2, 25.7, 25.4, 19.0, 13.7 ppm. – HRMS: calcd for $C_{17}H_{27}N_2O_2$ 291.1994, found 291.2072 [M+H$^+$].

4-Phenyl-1-(tetrahydrothiophen-2-yl)-1,2,4-triazolidine-3,5-dione (4p)

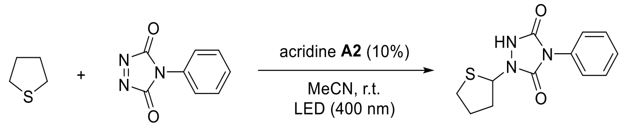

The general procedure **GP2** was followed with 4-phenyl-3*H*-1,2,4-triazole-3,5(4*H*)-dione (35.2 mg, 0.2 mmol), acridine **A2** (9.4 mg, 10 mol%, 0.02 mmol), tetrahydrothiophene (0.18 ml, 2 mmol) and acetonitrile (2 mL). The tube was capped with a screw cap and wrapped with parafilm then irradiated with LED light (λ_{max} = 400 nm) at room temperature for 24 hours. The reaction mixture was concentrated under reduced pressure and purified by flash chromatography on silica gel with EtOAc/petroleum ether (1 : 5 v/v) as an eluent to give the corresponding product **4p** (32.6 mg, 62%) as a white solid.

^{1}H NMR (400 MHz, $CDCl_3$): δ 7.53 – 7.43 (m, 4H), 7.43 – 7.34 (m, 1H), 5.98 (m, 1H), 3.13 – 3.05 (m, 1H), 2.88 – 2.79 (m, 1H), 2.28 – 2.14 (m, 3H), 2.12 – 2.00 (m, 1H) ppm. – ^{13}C NMR (100 MHz, $CDCl_3$): δ 154.0, 151.9, 131.1, 129.3, 128.5, 125.7, 65.3, 35.3, 33.5, 30.1 ppm. – HRMS: calcd for $C_{12}H_{14}N_3O_2S$ 264.0728, found 264.0625 [M+H$^+$].

2-(2-(Phenylsulfonyl)allyl)-1,4-dioxane (4q)

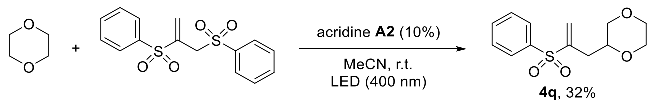

The general procedure **GP2** was followed with (prop-2-ene-1,2-diyldisulfonyl)dibenzene (64.4 mg, 0.2 mmol), acridine **A2** (9.4 mg, 10 mol%, 0.02 mmol), 1,4-dioxane (0.20 ml, 2 mmol) and acetonitrile (2 mL). The tube was capped with a screw cap and wrapped with parafilm then irradiated with LED light (λ_{max} = 400 nm) at room temperature for 24 hours. The reaction mixture was concentrated under reduced pressure and purified by flash chromatography on silica gel with EtOAc/petroleum ether (1 : 15 v/v) as an eluent to give the corresponding product **4q** (17.2 mg, 32%) as a yellow oil.

^{1}H NMR (400 MHz, $CDCl_3$): δ 7.87 (dd, *J* = 8.4, 1.3 Hz, 2H), 7.63 (d, *J* = 7.5 Hz, 1H), 7.55 (dd, *J* = 8.3, 6.9 Hz, 2H), 6.46 (s, 1H), 5.94 (d, *J* = 1.5 Hz, 1H), 3.64 (m, 4H), 3.59 – 3.45 (m, 2H), 3.18 (dd, *J* = 11.8, 10.1 Hz, 1H), 2.31 (dd, *J* = 5.9, 1.3 Hz, 2H) ppm. – ^{13}C NMR (100 MHz, $CDCl_3$): δ 146.1, 138.9, 133.8, 129.4, 128.4, 126.5, 72.9, 70.6, 66.7, 66.4, 31.8 ppm. – HRMS: calcd for $C_{13}H_{17}O_4S$ 269.0769, found269.0668 [M+H$^+$].

2-(2-(Phenylsulfonyl)allyl)tetrahydrothiophene (4r)

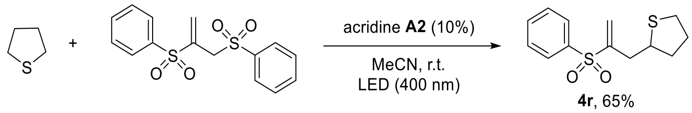

The general procedure **GP2** was followed with (prop-2-ene-1,2-diyldisulfonyl)dibenzene (64.4 mg, 0.2 mmol), acridine **A2** (9.4 mg, 10 mol%, 0.02 mmol), tetrahydrothiophene (0.036 ml, 0.4 mmol) and acetonitrile (2 mL). The tube was capped with a screw cap and wrapped with parafilm then irradiated with LED light (λ_{max} = 400 nm) at room temperature for 24 hours. The reaction mixture was concentrated under reduced pressure and purified by flash chromatography on silica gel with EtOAc/petroleum ether (1 : 15 v/v) as an eluent to give the corresponding product **4r** (34.9 mg, 65%) as a brown oil.

^{1}H NMR (400 MHz, $CDCl_3$): δ 7.91 – 7.83 (m, 2H), 7.66 – 7.59 (m, 1H), 7.59 – 7.51 (m, 2H), 6.41 (s, 1H), 5.86 (d, *J* = 1.7 Hz, 1H), 3.51 (m, 1H), 2.86 – 2.75 (m, 2H), 2.62 (m, 1H), 2.39 (m, 1H), 2.08 – 1.82 (m, 3H), 1.58 – 1.46 (m, 1H) ppm. – ^{13}C NMR (100 MHz, $CDCl_3$): δ 149.0, 138.8, 133.7, 129.4, 128.4, 125.1, 46.2, 37.6, 36.9, 32.5, 30.0 ppm. – HRMS: calcd for $C_{13}H_{17}O_2S_2$ 269.0592, found 269.0488 [M+H$^+$].

S-Cyclohexyl *O*-phenyl carbonothioate (4s)

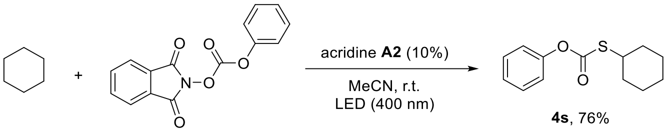

The general procedure **GP2** was followed with 1,3-dioxoisoindolin-2-yl phenyl carbonate (59.8 mg, 0.2 mmol), acridine **A2** (9.4 mg, 10 mol%, 0.02 mmol), cyclohexane (0.24 ml, 2 mmol) and acetonitrile (2 mL). The tube was capped with a screw cap and wrapped with parafilm then irradiated with LED light (λ_{max} = 400 nm) at room temperature for 24 hours. The reaction mixture was concentrated under reduced pressure and purified by flash chromatography on silica gel with EtOAc/petroleum ether (1 : 50 v/v) as an eluent to give the corresponding product **4s** (35.9 mg, 76%) as a yellow solid.

^{1}H NMR (400 MHz, $CDCl_3$): δ 7.30 (t, *J* = 7.9 Hz, 2H), 7.19 – 7.13 (m, 1H), 7.07 (dd, *J* = 7.5, 1.7 Hz, 2H), 3.36 (tt, *J* = 10.4, 3.8 Hz, 1H), 2.00 (dt, *J* = 13.8, 4.1 Hz, 2H), 1.75 – 1.62 (m, 2H), 1.61 – 1.30 (m, 6H), 1.23 (m, 2H) ppm. – ^{13}C NMR (100 MHz, $CDCl_3$): δ 170.0, 151.3, 129.6, 121.5, 45.1, 33.2, 26.0, 25.6 ppm. – HRMS: calcd for $C_{13}H_{17}O_2S$ 237.0871, found 237.0766 [M+H$^+$].

1-(Pyridin-4-yl)pyrrolidin-2-one (5a)

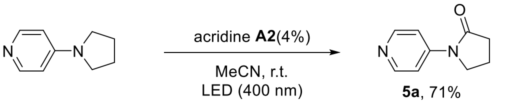

The general procedure **GP3** was followed with 4-(pyrrolidin-1-yl)pyridine (74.1 mg, 0.5 mmol), acridine **A2** (9.4 mg, 4 mol%, 0.02 mmol) and acetonitrile (0.5 mL). The tube was capped with a screw cap and wrapped with parafilm then irradiated with LED light (λ_{max} = 400 nm) at room temperature for 24 hours. The reaction mixture was concentrated under reduced pressure and purified by flash chromatography on silica gel with EtOAc/petroleum ether (1 : 100 v/v) as an eluent to give the corresponding product **5a** (57.6 mg, 71%) as a brown solid.

^{1}H NMR (400 MHz, $CDCl_3$): δ 8.55 – 8.43 (m, 2H), 7.60 – 7.50 (m, 2H), 3.80 (t, *J* = 7.1 Hz, 2H), 2.59 (t, *J* = 8.2 Hz, 2H), 2.15 (m, 2H) ppm. – ^{13}C NMR (100 MHz, $CDCl_3$): δ 175.2, 150.6, 145.9, 112.8, 47.5, 32.9, 17.7 ppm. – HRMS: calcd for $C_9H_{11}N_2O$ 163.0793, found 163.0867 [M+H$^+$].

1-(3-Bromophenyl)ethan-1-one (5b)

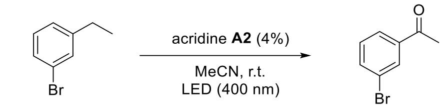

5b, 55%

The general procedure **GP3** was followed with 1-bromo-3-ethylbenzene (92.5 mg, 0.5 mmol), acridine **A2** (9.4 mg, 4 mol%, 0.02 mmol) and acetonitrile (0.5 mL). The tube was capped with a screw cap and wrapped with parafilm then irradiated with LED light (λ_{max} = 400 nm) at room temperature for 24 hours. The reaction mixture was concentrated under reduced pressure and purified by flash chromatography on silica gel with EtOAc/petroleum ether (1 : 200 v/v) as an eluent to give the corresponding product **5b** (54.7 mg, 55%) as a brown oil.

^{1}H NMR (400 MHz, $CDCl_3$): δ 8.07 (t, *J* = 1.8 Hz, 1H), 7.86 (m, 1H), 7.67 (m, 1H), 7.33 (t, *J* = 7.9 Hz, 1H), 2.58 (s, 3H) ppm. – ^{13}C NMR (100 MHz, $CDCl_3$): δ 196.7, 138.9, 136.0, 131.5, 130.3, 126.9, 123.0, 26.7 ppm. – HRMS: calcd for C_8H_8BrO 198.9680, found 198.9579 [M+H$^+$].

3,4-Dihydronaphthalen-1(2*H*)-one (5c)

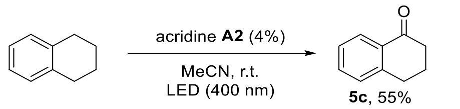

5c, 55%

The general procedure **GP3** was followed with 1,2,3,4-tetrahydronaphthalene (66.1 mg, 0.5 mmol), acridine **A2** (9.4 mg, 4 mol%, 0.02 mmol) and acetonitrile (0.5 mL). The tube was capped with a screw cap and wrapped with parafilm then irradiated with LED light (λ_{max} = 400 nm) at room temperature for 24 hours. The reaction mixture was concentrated under reduced pressure and purified by flash chromatography on silica gel with EtOAc/petroleum ether (1 : 100 v/v) as an eluent to give the corresponding product **5c** (40.2 mg, 55%) as a brown oil.

^{1}H NMR (400 MHz, $CDCl_3$): δ 8.03 (dd, *J* = 7.8, 1.5 Hz, 1H), 7.47 (td, *J* = 7.5, 1.5 Hz, 1H), 7.34 – 7.22 (m, 2H), 2.97 (t, *J* = 6.1 Hz, 2H), 2.66 (dd, *J* = 7.3, 5.8 Hz, 2H), 2.15 (m, 2H) ppm. – ^{13}C NMR (100 MHz, $CDCl_3$): δ 198.5, 144.6, 133.5, 132.7, 128.9, 127.3, 126.7, 39.3, 29.8, 23.4 ppm. – HRMS: calcd for $C_{10}H_{11}O$ 147.0732, found 147.0807 [M+H$^+$].

9*H*-Fluoren-9-one (5d)

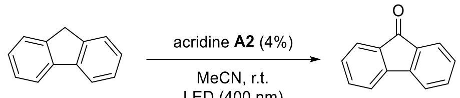

5d, 41%

The general procedure **GP3** was followed with 9*H*-fluorene (83.1 mg, 0.5 mmol), acridine **A2** (9.4 mg, 4 mol%, 0.02 mmol) and acetonitrile (0.5 mL). The tube was capped with a screw cap and wrapped with parafilm then irradiated with LED light (λ_{max} = 400 nm) at room temperature for 24 hours. The reaction mixture was concentrated under reduced pressure and purified by flash chromatography on silica gel with EtOAc/petroleum ether (1 : 100 v/v) as an eluent to give the corresponding product **5d** (36.9 mg, 41%) as a yellow solid.

^{1}H NMR (400 MHz, $CDCl_3$): δ 7.59 (d, *J* = 7.4 Hz, 2H), 7.47 – 7.37 (m, 4H), 7.22 (m, 2H) ppm. – ^{13}C NMR (100 MHz, $CDCl_3$): δ 194.0, 144.5, 134.8, 134.2, 129.2, 124.4, 120.4 ppm. – HRMS: calcd for $C_{13}H_9O$ 181.0575, found 181.0465 [M+H$^+$].

2-Bromo-9*H*-fluoren-9-one (5e)

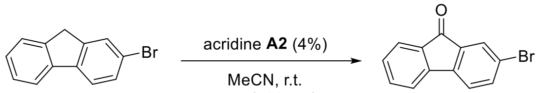

5e, 42%

The general procedure **GP3** was followed with 2-bromo-9*H*-fluorene (122.6 mg, 0.5 mmol), acridine **A2** (9.4 mg, 4 mol%, 0.02 mmol) and acetonitrile (0.5 mL). The tube was capped with a screw cap and wrapped with parafilm then

irradiated with LED light (λ_{max} = 400 nm) at room temperature for 24 hours. The reaction mixture was concentrated under reduced pressure and purified by flash chromatography on silica gel with EtOAc/petroleum ether (1 : 100 v/v) as an eluent to give the corresponding product **5e** (54.4 mg, 42%) as a yellow solid.

^{1}H NMR (400 MHz, $CDCl_3$): δ 7.66 (d, *J* = 1.9 Hz, 1H), 7.57 (d, *J* = 7.4 Hz, 1H), 7.52 (dd, *J* = 7.9, 1.9 Hz, 1H), 7.47 – 7.39 (m, 2H), 7.33 – 7.20 (m, 2H) ppm. – ^{13}C NMR (100 MHz, $CDCl_3$): δ 192.4, 143.7, 143.1, 137.2, 135.8, 135.1, 133.8, 129.5, 127.6, 124.7, 123.0, 121.8, 120.5 ppm. – HRMS: calcd for $C_{13}H_8BrO$ 258.9680, found 258.9578 [M+H$^+$].

Benzophenone (5f)

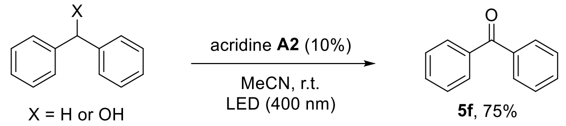

From diphenylmethane. The general procedure **GP3** was followed with diphenylmethane (33.6 mg, 0.2 mmol), acridine **A2** (9.4 mg, 10 mol%, 0.02 mmol) and acetonitrile (0.2 mL). The tube was capped with a screw cap and wrapped with parafilm then irradiated with LED light (λ_{max} = 400 nm) at room temperature for 24 hours. The reaction mixture was concentrated under reduced pressure and purified by flash chromatography on silica gel with EtOAc/petroleum ether (1 : 200 v/v) as an eluent to give the corresponding product **5f** (27.3 mg, 75%) as a white solid.

From diphenylmethanol. The general procedure **GP3** was followed with diphenylmethanol (36.8 mg, 0.2 mmol), acridine **A2** (9.4 mg, 10 mol%, 0.02 mmol) and acetonitrile (0.2 mL). The tube was capped with a screw cap and wrapped with parafilm then irradiated with LED light (λ_{max} = 400 nm) at room temperature for 24 hours. The reaction mixture was concentrated under reduced pressure and purified by flash chromatography on silica gel with EtOAc/petroleum ether (1 : 200 v/v) as an eluent to give the corresponding product **5f** (32.4 mg, 89%) as a white solid.

^{1}H NMR (400 MHz, $CDCl_3$): δ 7.81 (d, *J* = 7.8 Hz, 4H), 7.58 (d, *J* = 7.6 Hz, 2H), 7.48 (t, *J* = 7.8 Hz, 4H) ppm. – ^{13}C NMR (100 MHz, $CDCl_3$): δ 196.8, 137.6, 132.4, 130.1, 128.3 ppm. – HRMS: calcd for $C_{13}H_{11}O$ 183.0732, found 183.0632 [M+H$^+$].

1-(((2*S*,4*R*,5*S*)-5-Isopropyl-2-methyl-4-((triisopropylsilyl)oxy)cyclohexyl)oxy)-2,2,6,6-tetramethylpiperidin-4-ol (6a)

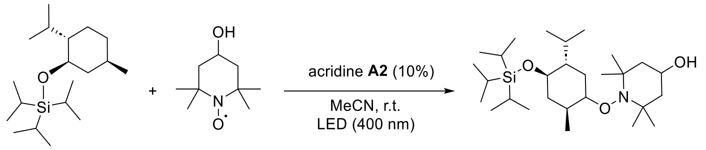

The general procedure **GP1** was followed with 4-OH-TEMPO (34.4 mg, 0.2 mmol), acridine **A2** (4.7 mg, 10 mol%, 0.02 mmol), triisopropyl(((1*R*,2*S*,5*R*)-2-isopropyl-5-methylcyclohexyl)oxy)silane (125.0 mg, 0.4 mmol) and acetonitrile (2 mL). The tube was capped with a screw cap and wrapped with parafilm then irradiated with LED light (λ_{max} = 400 nm) at room temperature for 24 hours. The reaction mixture was concentrated under reduced pressure and purified by flash chromatography on silica gel with EtOAc/petroleum ether (1 : 10 v/v) as an eluent to give the corresponding product **6a** (28.1 mg, 58%) as a yellow oil.

^{1}H NMR (400 MHz, $CDCl_3$): δ 4.00 – 3.89 (m, 2H), 3.76 (m, 1H), 3.52 (td, *J* = 10.2, 4.2 Hz, 1H), 2.30 (m, 1H), 1.98 – 1.88 (m, 1H), 1.79 (dd, *J* = 12.5, 4.1 Hz, 2H), 1.61 (m, 2H), 1.51 – 1.42 (m, 2H), 1.28 – 1.12 (m, 18H), 1.12 – 0.98 (m, 18H), 0.91 – 0.83 (m, 6H), 0.74 (d, J = 6.9 Hz, 3H) ppm. – ^{13}C NMR (100 MHz, $CDCl_3$): δ 78.6, 73.1, 73.1, 63.6, 60.4, 60.0, 50.9, 50.8, 48.6, 48.4, 45.9, 45.9, 34.7, 33.6, 33.4, 31.9, 25.1, 22.8, 22.5, 21.6, 21.6, 21.3, 21.3, 21.1, 21.1, 18.4, 18.4, 18.4, 18.3, 18.3, 16.1, 16.0, 13.5, 13.3, 13.3, 13.2, 13.1, 13.1 ppm. – HRMS: calcd for $C_{28}H_{58}NO_3Si$ 484.4108, found 484.4186 [M+H$^+$].

6-(((4-Hydroxy-2,2,6,6-tetramethylpiperidin-1-yl)oxy)methyl)-2*H*-chromen-2-one (6b)

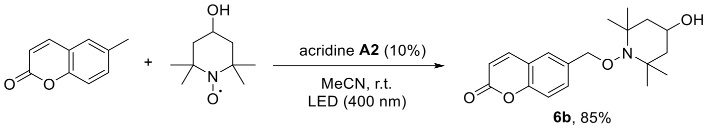

The general procedure **GP5** was followed with 4-OH-TEMPO (34.4 mg, 0.2 mmol), acridine **A2** (4.7 mg, 10 mol%, 0.02 mmol), 6-Methylcoumarin (125.0 mg, 0.4 mmol) and acetonitrile (2 mL). The tube was capped with a screw cap and wrapped with parafilm then irradiated with LED light (λ_{max} = 400 nm) at room temperature for 24 hours. The reaction mixture was concentrated under reduced pressure and purified by flash chromatography on silica gel with EtOAc/petroleum ether (1 : 2 v/v) as an eluent to give the corresponding product **6b** (28.2 mg, 85%) as a brown solid.
^{1}H NMR (400 MHz, $CDCl_3$): δ 7.72 (d, *J* = 9.5 Hz, 1H), 7.50 (dd, *J* = 8.5, 2.0 Hz, 1H), 7.45 (d, *J* = 1.9 Hz, 1H), 7.31 (d, *J* = 8.4 Hz, 1H), 6.43 (d, *J* = 9.5 Hz, 1H), 4.84 (s, 2H), 3.99 (tt, *J* = 11.4, 4.2 Hz, 1H), 1.85 (m, 2H), 1.52 (t, J = 11.8 Hz, 2H), 1.28 (s, 6H), 1.20 (s, 6H) ppm. – ^{13}C NMR (100 MHz, $CDCl_3$): δ 160.9, 153.4, 143.5, 134.5, 131.2, 126.6, 118.7, 116.9, 116.8, 77.9, 63.2, 60.4, 48.3, 33.3, 29.7, 21.3 ppm. – HRMS: calcd for $C_{19}H_{26}NO_4$ 332.1784, found 332.1680 [M+H$^+$].

4-(3-(*tert*-Butoxy)-1,1-dicyanopropan-2-yl)phenyl 5-(2,5-dimethylphenoxy)-2,2-dimethylpentanoate (6c)

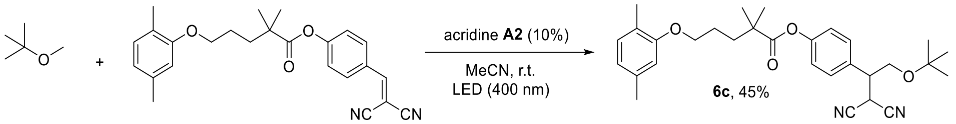

The general procedure **GP5** was followed with 4-(2,2-dicyanovinyl)phenyl 5-(2,5-dimethylphenoxy)-2,2-dimethylpentanoate (80.5 mg, 0.2 mmol), acridine **A2** (9.4 mg, 10 mol%, 0.02 mmol), 2-methoxy-2-methylpropane (0.24 ml, 2 mmol) and acetonitrile (2 mL). The tube was capped with a screw cap and wrapped with parafilm then irradiated with LED light (λ_{max} = 400 nm) at room temperature for 24 hours. The reaction mixture was concentrated under reduced pressure and purified by flash chromatography on silica gel with EtOAc/petroleum ether (1 : 40 v/v) as an eluent to give the corresponding product **6c** (44.2 mg, 45%) as a yellow oil.
^{1}H NMR (400 MHz, $CDCl_3$): δ 7.44 – 7.39 (m, 2H), 7.12 – 7.07 (m, 2H), 7.01 (d, *J* = 7.4 Hz, 1H), 6.71 – 6.61 (m, 2H), 4.45 (d, *J* = 5.8 Hz, 1H), 4.00 (d, *J* = 5.3 Hz, 2H), 3.80 – 3.71 (m, 2H), 3.43 – 3.36 (m, 1H), 2.31 (s, 3H), 2.18 (s, 3H), 1.94 – 1.82 (m, 4H), 1.38 (s, 6H), 1.25 (s, 9H) ppm. – ^{13}C NMR (100 MHz, $CDCl_3$): δ 176.2, 157.0, 151.7, 136.6, 132.4, 130.5, 129.5, 123.7, 122.4, 120.9, 112.4, 112.1, 111.8, 74.4, 67.8, 61.2, 46.4, 42.6, 37.3, 27.5, 26.4, 25.4, 25.2, 21.5, 15.9 ppm. – HRMS: calcd for $C_{30}H_{39}N_2O_4$ 491.2832, found 491.2728 [M+H$^+$].

Ethyl 2-(4-(2,2-dicyano-1-cyclohexylethyl)phenoxy)-2-methylpropanoate (6d)

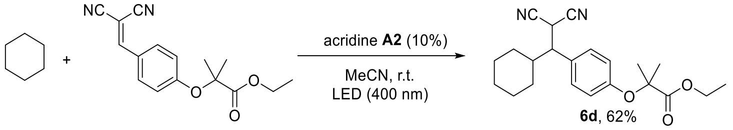

The general procedure **GP2** was followed with ethyl 2-(4-(2,2-dicyanovinyl)phenoxy)-2-methylpropanoate (56.9 mg, 0.2 mmol), acridine **A2** (9.4 mg, 10 mol%, 0.02 mmol), cyclohexane (0.24 ml, 2 mmol) and acetonitrile (2 mL). The tube was capped with a screw cap and wrapped with parafilm then irradiated with LED light (λ_{max} = 400 nm) at room temperature for 24 hours. The reaction mixture was concentrated under reduced pressure and purified by flash chromatography on silica gel with EtOAc/petroleum ether (1 : 50 v/v) as an eluent to give the corresponding product **6d** (45.7 mg, 62%) as a yellow oil.
^{1}H NMR (400 MHz, $CDCl_3$): δ 7.21 – 7.13 (m, 2H), 6.87 – 6.81 (m, 2H), 4.22 (q, *J* = 7.2 Hz, 2H), 4.15 (d, *J* = 5.4 Hz, 1H), 2.81 (dd, *J* = 9.8, 5.4 Hz, 1H), 2.01 – 1.75 (m, 3H), 1.61 (s, 8H), 1.50 – 1.28 (m, 2H), 1.21 (t, *J* = 7.2 Hz, 3H), 1.18 – 0.96 (m, 3H), 0.84 – 0.71 (m, 1H) ppm. – ^{13}C NMR (100 MHz, $CDCl_3$): δ 174.2, 155.9, 130.0, 129.2, 119.2, 112.4, 112.1, 79.3, 61.6,

51.8, 39.4, 31.3, 30.7, 27.4, 26.0, 25.9, 25.8, 25.6, 25.5, 14.2 ppm. – HRMS: calcd for $C_{22}H_{29}N_2O_3$ 369.2100, found 369.1995 [M+H+].

Methyl 3-cyclohexyl-2-(11-oxo-6,11-dihydrodibenzo[*b*,*e*]oxepin-2-yl)propanoate (6e)

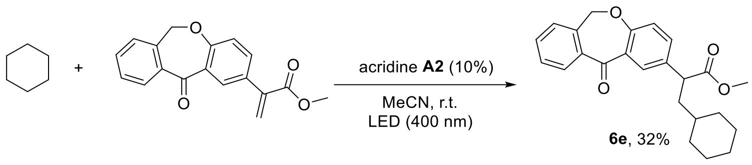

The general procedure **GP2** was followed with methyl 2-(11-oxo-6,11-dihydrodibenzo[*b*,*e*]oxepin-2-yl)acrylate (58.3 mg, 0.2 mmol), acridine **A2** (9.4 mg, 10 mol%, 0.02 mmol), cyclohexane (0.24 ml, 2 mmol) and acetonitrile (2 mL). The tube was capped with a screw cap and wrapped with parafilm then irradiated with LED light (λ_{max} = 400 nm) at room temperature for 24 hours. The reaction mixture was concentrated under reduced pressure and purified by flash chromatography on silica gel with EtOAc/petroleum ether (1 : 100 v/v) as an eluent to give the corresponding product **6e** (24.2 mg, 32%) as a yellow oil.

^{1}H NMR (400 MHz, $CDCl_3$): δ 8.12 (d, *J* = 2.4 Hz, 1H), 7.90 (dd, *J* = 7.7, 1.4 Hz, 1H), 7.56 (td, *J* = 7.4, 1.5 Hz, 1H), 7.50 – 7.45 (m, 2H), 7.36 (dd, *J* = 7.7, 1.4 Hz, 1H), 7.02 (d, *J* = 8.5 Hz, 1H), 5.18 (s, 2H), 3.75 (t, *J* = 7.8 Hz, 1H), 3.65 (s, 3H), 1.99 (m, 1H), 1.77 – 1.59 (m, 6H), 1.20 – 1.08 (m, 4H), 0.91 (m, 2H) ppm. – ^{13}C NMR (100 MHz, $CDCl_3$): δ 191.1, 174.8, 160.6, 140.7, 135.7, 134.8, 133.4, 132.9, 131.5, 129.6, 129.4, 127.9, 125.3, 121.3, 73.7, 52.2, 48.0, 41.0, 35.4, 33.4, 33.1, 26.6, 26.3, 26.2 ppm. – HRMS: calcd for $C_{24}H_{27}O_4$ 379.1831, found 379.1732 [M+H+].

4-(2,2-Dicyano-1-cyclohexylethyl)phenyl 3-(4,5-diphenyloxazol-2-yl)propanoate (6f)

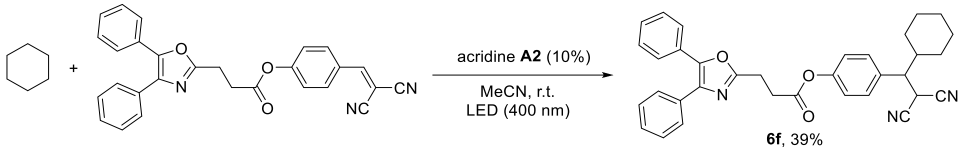

The general procedure **GP2** was followed with 4-(2,2-dicyanovinyl)phenyl 3-(4,5-diphenyloxazol-2-yl)propanoate (82.8 mg, 0.2 mmol), acridine **A2** (9.4 mg, 10 mol%, 0.02 mmol), cyclohexane (0.24 ml, 2 mmol) and acetonitrile (2 mL). The tube was capped with a screw cap and wrapped with parafilm then irradiated with LED light (λ_{max} = 400 nm) at room temperature for 24 hours. The reaction mixture was concentrated under reduced pressure and purified by flash chromatography on silica gel with EtOAc/petroleum ether (1 : 10 v/v) as an eluent to give the corresponding product **6f** (41.3 mg, 39%) as a yellow oil.

^{1}H NMR (400 MHz, $CDCl_3$): δ 7.70 – 7.63 (m, 2H), 7.62 – 7.56 (m, 2H), 7.36 (m, 8H), 7.21 – 7.14 (m, 2H), 4.17 (d, *J* = 5.2 Hz, 1H), 3.31 (t, *J* = 7.0 Hz, 2H), 3.18 (t, *J* = 7.0 Hz, 2H), 2.88 (dd, *J* = 9.8, 5.2 Hz, 1H), 2.06 – 1.78 (m, 3H), 1.72 – 1.61 (m, 2H), 1.42 (m, 2H), 1.22 – 1.00 (m, 3H), 0.91 – 0.74 (m, 1H) ppm. – ^{13}C NMR (100 MHz, $CDCl_3$): δ 170.5, 161.5, 150.9, 145.7, 135.3, 134.4, 132.5, 129.4, 129.0, 128.8, 128.7, 128.7, 128.2, 128.0, 126.7, 122.3, 112.2, 111.9, 51.8, 39.4, 31.4, 31.2, 30.7, 27.2, 25.9, 25.8, 23.6 ppm. – HRMS: calcd for $C_{34}H_{32}N_3O_3$ 530.2365, found 530.2435 [M+H+].

Ethyl 3-cyclohexyl-2-(4-isobutylphenyl)propanoate (6g)

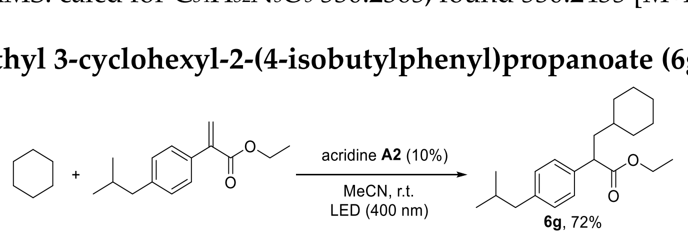

The general procedure **GP2** was followed with ethyl 2-(4-isobutylphenyl)acrylate (46.4 mg, 0.2 mmol), acridine **A2** (9.4 mg, 10 mol%, 0.02 mmol), cyclohexane (0.24 ml, 2 mmol) and acetonitrile (2 mL). The tube was capped with a screw cap and wrapped with parafilm then irradiated with LED light (λ_{max} = 400 nm) at room temperature for 24

hours. The reaction mixture was concentrated under reduced pressure and purified by flash chromatography on silica gel with EtOAc/petroleum ether (1 : 200 v/v) as an eluent to give the corresponding product **6g** (45.6 mg, 72%) as a brown oil.

^{1}H NMR (400 MHz, $CDCl_3$): δ 7.24 – 7.18 (m, 2H), 7.10 – 7.04 (m, 2H), 4.19 – 4.02 (m, 2H), 3.64 (dd, *J* = 8.7, 6.9 Hz, 1H), 2.44 (d, *J* = 7.2 Hz, 2H), 2.02 – 1.92 (m, 1H), 1.91 – 1.78 (m, 1H), 1.77 – 1.69 (m, 2H), 1.67 – 1.57 (m, 4H), 1.35 – 1.10 (m, 9H), 0.89 (d, *J* = 6.6 Hz, 6H) ppm. – ^{13}C NMR (100 MHz, $CDCl_3$): δ 174.7, 140.5, 137.0, 129.4, 127.7, 60.7, 48.7, 45.2, 41.4, 35.6, 33.3, 33.3, 30.3, 26.7, 26.3, 22.5, 14.3 ppm. – HRMS: calcd for $C_{21}H_{33}O_2$ 317.2402, found 317.2197 [M+H$^+$].

N-(4-(1-Butyl-3-ethyl-2,6-dioxopiperidin-3-yl)phenyl)-3-cyclohexyl-2-phenylpropanamide (6h)

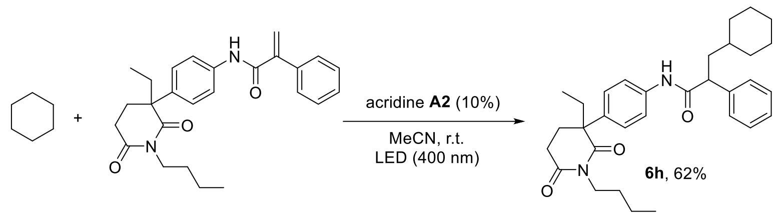

The general procedure **GP2** was followed with *N*-(4-(1-Butyl-3-ethyl-2,6-dioxopiperidin-3-yl)phenyl)-2-phenylacrylamide (83.7 mg, 0.2 mmol), acridine **A2** (9.4 mg, 10 mol%, 0.02 mmol), cyclohexane (0.24 ml, 2 mmol) and acetonitrile (2 mL). The tube was capped with a screw cap and wrapped with parafilm then irradiated with LED light (λ_{max} = 400 nm) at room temperature for 24 hours. The reaction mixture was concentrated under reduced pressure and purified by flash chromatography on silica gel with EtOAc/petroleum ether (1 : 2 v/v) as an eluent to give the corresponding product **6h** (62.3 mg, 62%) as a yellow oil.

^{1}H NMR (400 MHz, $CDCl_3$): δ 7.42 (d, *J* = 8.5 Hz, 2H), 7.34 (d, *J* = 5.7 Hz, 4H), 7.29 (dd, *J* = 6.0, 2.6 Hz, 1H), 7.19 – 7.13 (m, 1H), 7.12 – 7.07 (m, 2H), 3.77 (m, 2H), 3.62 (t, *J* = 7.6 Hz, 1H), 2.64 – 2.56 (m, 1H), 2.40 (m, 1H), 2.25 (m, 1H), 2.12 (m, 2H), 2.06 – 1.94 (m, 1H), 1.90 – 1.80 (m, 1H), 1.79 – 1.57 (m, 5H), 1.54 – 1.41 (m, 2H), 1.37 – 1.22 (m, 3H), 1.13 (d, *J* = 7.3 Hz, 4H), 0.91 (t, *J* = 7.3 Hz, 5H), 0.85 – 0.77 (m, 3H) ppm. – ^{13}C NMR (100 MHz, $CDCl_3$): δ 175.2, 172.1, 172.1, 139.9, 137.3, 129.2, 128.1, 127.6, 126.9, 120.1, 51.5, 51.1, 40.8, 40.2, 35.3, 33.9, 33.7, 33.0, 30.1, 30.0, 26.6, 26.3, 26.2, 26.0, 20.4, 13.9, 9.2 ppm. – HRMS: calcd for $C_{32}H_{43}N_2O_3$ 503.3195, found 503.3097 [M+H$^+$].

Computational studies

Calculations were performed using computational resources at the Texas Advanced Computing Centers (TACC) hosted by The University of Texas at Austin and the Advanced Cyberinfrastructure Coordination Ecosystem: Services and Support (ACCESS). Density Functional Theory (DFT) optimization, vibrational analysis, and Intrinsic Reaction Coordinate (IRC) calculations were conducted with Gaussian 16 (rC.01).[9] Visualizations and monitoring of calculations were performed using Chemcraft.[10] Images were rendered using CYLview 2.0[11] and VMD 1.9.3.[12]

Details of Computational Methods

Ground state minima and transition states were optimized without constraints using the ωB97X-D density functional approximation (DFA) and def2-SVP basis set. All structures were optimized in acetonitrile using the SMD solvent model. Optimizations were performed with a convergence criterion of "tight" and an ultrafine grid. Frequency calculations were performed at the same level of theory to classify each stationary point. Geometries with no imaginary frequencies were deemed minima whereas those with one imaginary frequency along the reaction coordinate were deemed transition states. An intrinsic reaction coordinate (IRC) calculation was performed for each transition state to further corroborate the transition state connected reactants to products. A cut-off frequency of 50 cm^{-1} was selected for all structures to correct for potential errors associated with low magnitude vibrational frequencies via GoodVibes.[13] Single point calculations were performed at the ωB97X-D / 6-311++G** / SMD (MeCN) level of theory. Benchmarking studies for the Gibbs free activation energies for key HAT processes mediated by acridines **A1** and **A2**. with a range of DFA and basis set combinations revealed that the differences between the barriers to the HAT processes are consistent across a variety of methods (Table S1).

Table S1. Benchmarking studies for the Gibbs free activation energies of **A1**- and **A2**-mediated HAT processes.[a]

Entry	Optimization	Single point	$\Delta G^{\neq}$, kcal/mol			
			**TS2**-*anti*	**TS2**-*syn*	**TS1**-*anti*	**TS1**-*syn*
1	ωB97X-D/def2-SVP	ωB97X-D/6-311+G**	25.4	21.8	24.6	24.7
2	ωB97X-D/def2-SVP	ωB97X-D/def2-TZVP	26.4	23.2	25.6	25.8
3	ωB97X-D/def2-SVP	M06-D3/6-311+G*	27.4	22.1	26.2	26.0
4	ωB97X-D/def2-SVP	ωB97X-D/cc-pvTZ	26.1	22.5	25.2	25.6
5	ωB97X-D/def2-SVP	M06-2X-D3/6-311+G**	25.4	22.7	24.5	24.5
6	ωB97X-D/6-31++G(2d,p)	ωB97X-D/6-311+G**	23.4	20.3	23.0	22.8
7	ωB97X-D/Aug-cc-pVDZ	ωB97X-D/6-311+G**	23.5	20.5	22.9	22.8
8	Cam-B3LYP-D3BJ/Aug-cc-pVDZ	ωB97X-D/6-311+G**	23.0	19.9	22.7	22.3
9	M06-2X-D3/Aug-cc-pVDZ	ωB97X-D/6-311+G**	22.8	19.8	22.6	22.0
10	Cam-B3LYP-D3BJ/6-31+G**	ωB97X-D/6-311+G**	23.5	19.7	22.8	22.7
11	M06-2X-D3/6-31++G*	ωB97X-D/6-311+G**	22.8	19.6	22.5	22.3

[a] Single-point calculations were conducted with acetonitrile as the solvent using the SMD solvent model.

Investigation of the acridine-catalyzed direct HAT C–H functionalization

Computational studies were carried out to elucidate the details of the acridine-catalyzed direct HAT C–H functionalization (Figure S3). Following the photoexcitation, acridine **A2** can undergo intersystem crossing to the triplet ^{3}**A2**, which can mediate hydrogen atom abstraction from C–H substrate **1**. The most kinetically favorable pathway traverses transition state structure **TS2-*syn***, producing acridinyl radical **HA2** and alkyl radical **S19** This pathway has a substantially lower barrier that the one traversing transition state structure **TS2-*anti***. Subsequent kinetically facile ($\Delta G^{\neq}$ = 8.1 kcal/mol) addition of alkyl radical **S19** to Michael acceptor **S20** affords α-carbonyl radical **S21**, which can engage acridinyl radical **HA2** in an exergonic and nearly barrierless single electron transfer (SET) process. The subsequent proton transfer between the resulting acridinium cation **HA2**$^+$ and enolate **S21**$^-$ leads to the regeneration of catalyst **A2** and formation of product **S22**. These results are consistent with the experimentally observed facile acridine **A2**-catalyzed C–H functionalization of C–H substrates.

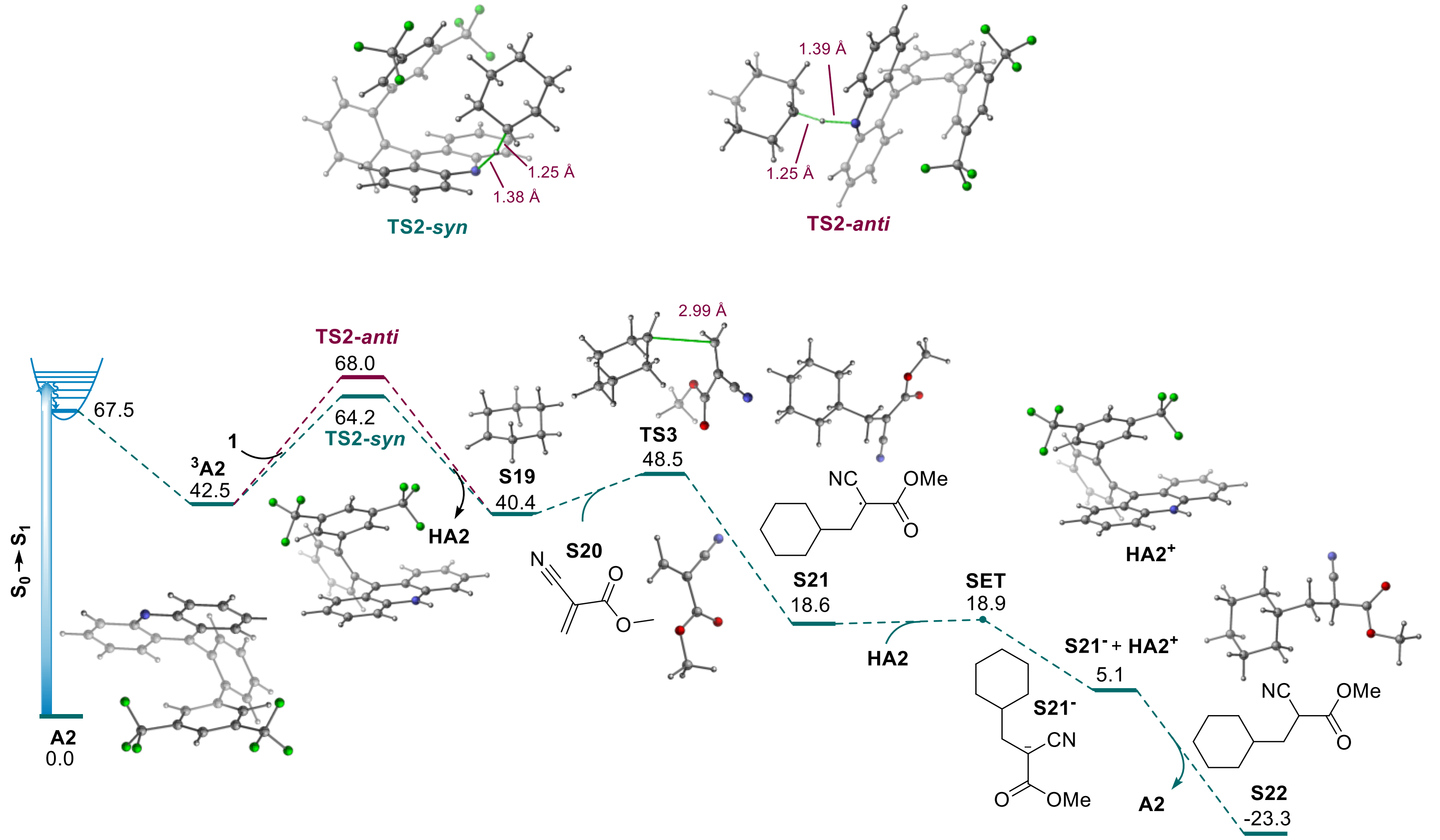

Figure S3. Computed Gibbs free energy profile for the acridine **A2**-catalyzed C–H functionalization, kcal/mol.

Investigation of the influence of quantum tunneling on the acridine-mediated HAT process

The influence of quantum tunneling on the acridine-mediated HAT process was probed using the Wigner Transmission Coefficient (WTC) approximated to the second order[14,15] with the imaginary frequencies (in Hz) of computationally derived transition state structures **TS1-*syn***, **TS1-*anti***, and **TS2-*syn***, **TS2-*anti*** applied to calculate the transmission coefficient κ:

$$\kappa = 1 + \frac{1}{24}\left(\frac{\hbar \mathrm{Im}(v^{\neq})}{k_B T}\right)^2$$

The calculated tunneling-corrected transmission coefficient (κ = 1.1 for all four transition state structures) indicated

that tunneling was not a significant contributor to the HAT process. This result is consistent with the experimentally observed kinetic isotope effect (KIE, k_H/k_D = 1.9), which is in the normal KIE range of ~2–7.[16]

Investigation of the influence of trifluoromethyl groups on the direct HAT process

Computational studies were conducted to determine the effect of the trifluoromethyl groups on the direct HAT process by comparing the barriers for the HAT step with acridines **A2** and **A7**. The studies indicate that the trifluoromethyl groups have a strong effect on the barrier for the *syn*-TS, while no stabilizing effect is observed for the *anti*-TS (Table S2). These results are consistent with the key role of noncovalent interactions resulting from the changes in the properties of the *ortho*-aryl substituents upon the introduction of the trifluoromethyl groups in the HAT process.

Table S2. Gibbs free activation energies for the HAT process with **A2** and **A7**.

$\Delta G^{\neq}$, kcal/mol			
**A2**		**A7**	
**TS2-*anti***	**TS2-*syn***	**TS3-*anti***	**TS3-*syn***
25.5	21.7	25.3	24.0

TD-DFT and electron–hole analysis

Time-dependent density functional theory (TD-DFT) calculations for acridine structures **A1** and **A2** were performed at the ωB97X-D / 6-311+G** / SMD (MeCN) level of theory as previously described.[17] An optimization and frequency calculation was performed for both the singlet and triplet states using "roots=1" and "roots=1,Triplets" keywords respectively in the TD options list. The first 10 excited states were collected for each calculation, "Nstates=10", with the added keyword "IOP(9/40=4)" to ensure all configuration coefficients with magnitudes larger than 0.0001 were collected. An electron-hole analysis was performed with the formatted checkpoint and output files via Multiwfn.

Table S3. Results of the electron–hole analysis for the three lowest singlet excited states of **A1**.

Excited state	D, Å	S_r, a.u.	Δσ	HCT	H, Å	t, Å	Δ*E*, eV
**S1**	0.20	0.87	–0.10	1.53	2.91	–1.33	3.00
**S2**	0.15	0.93	0.11	1.39	2.90	1.24	3.88
**S3**	1.16	0.49	0.92	1.23	2.26	0.06	3.98

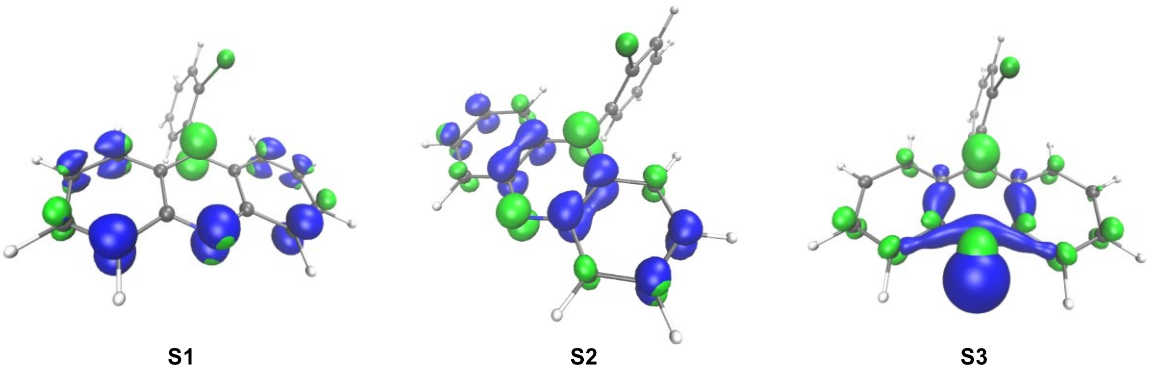

Figure S4. Hole-electron analysis for the three lowest singlet excited states of **A1**.

Table S4. Results of the electron–hole analysis for the three lowest triplet excited states of **A1**.

**Excited state**	**D, Å**	**S_r, a.u.**	**Δσ**	**HCT**	**H, Å**	**t, Å**	**Δ*E*, eV**
**T1**	0.18	0.88	0.02	1.58	2.83	–1.40	1.70
**T2**	0.11	0.88	0.08	1.66	3.07	–1.55	3.23
**T3**	0.16	0.69	0.05	1.49	2.84	–1.32	3.39

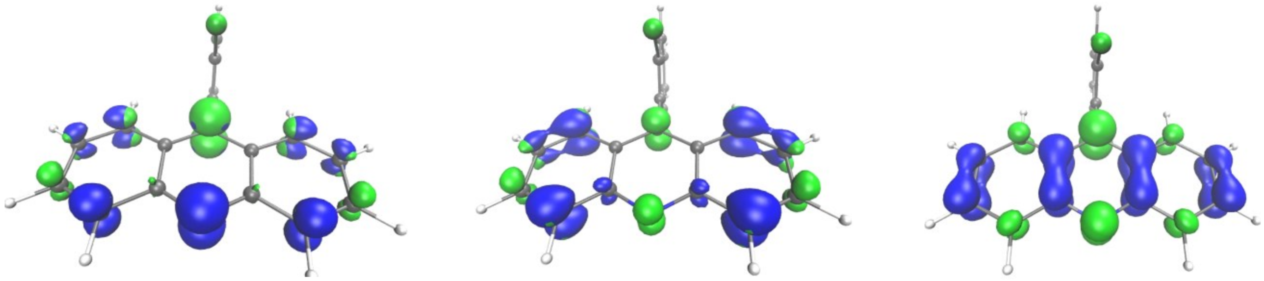

T1 T2 T3

Figure S5. Hole-electron analysis for the three lowest triplet excited states of **A1**.

Table S5. Results of the electron–hole analysis for the three lowest singlet excited states of **A2**.

**Excited state**	**D, Å**	**S_r, a.u.**	**Δσ**	**HCT**	**H, Å**	**t, Å**	**Δ*E*, eV**
**S1**	0.19	0.87	–0.07	1.59	2.98	–1.40	2.94
**S2**	0.20	0.92	0.17	1.53	2.96	–1.34	3.85
**S3**	1.18	0.49	0.94	1.29	2.33	–0.11	3.97

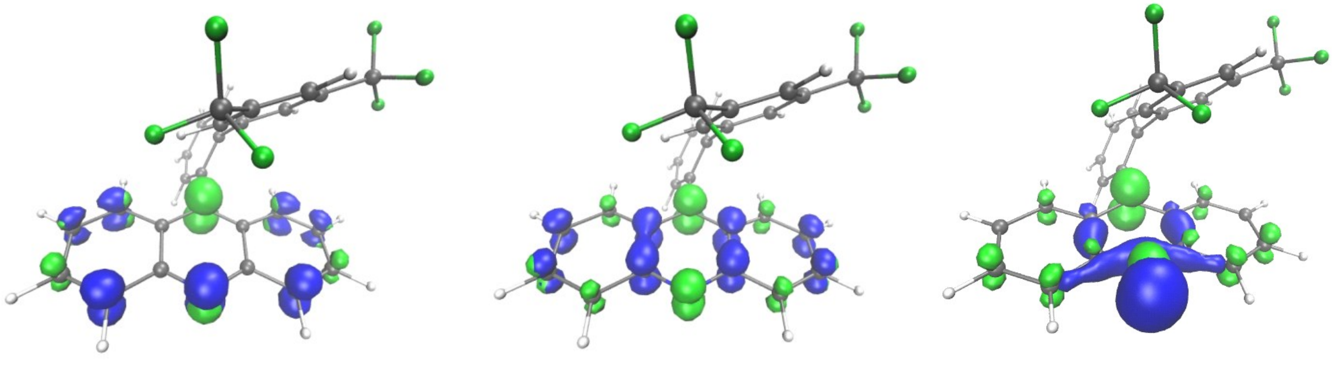

S1 S2 S3

Figure S6. Hole-electron analysis for the three lowest singlet excited states of **A2**.

Table S6. Results of the electron–hole analysis for the three lowest triplet excited states of **A2**.

Excited state	D, Å	S_r, a.u.	Δσ	HCT	H, Å	t, Å	Δ*E*, eV
**T1**	0.19	0.87	0.05	1.70	2.90	–1.51	1.68
**T2**	0.16	0.66	0.13	1.85	3.12	–1.68	3.23
**T3**	0.22	0.69	0.01	1.69	2.89	–1.47	3.39

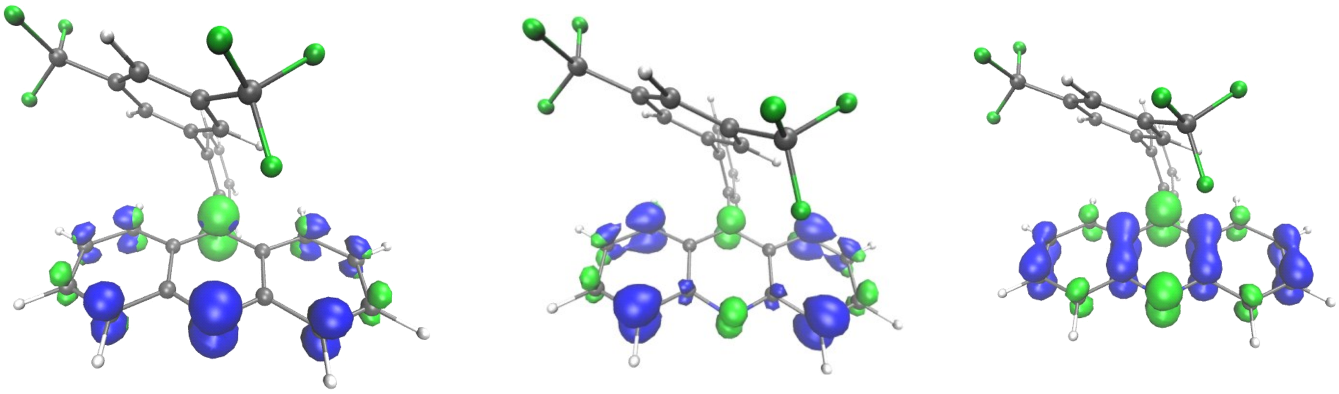

T1 T2 T3

Figure S7. Hole-electron analysis for the three lowest triplet excited states of **A2**.

Spin density calculations

Spin density was collected from structures **A1**, **A2**, **HA1**, and **HA2**. Spin density images were rendered in VMD using cube files that were collected from the formatted check files of the optimized structures. An isovalue of ± 0.005 was utilized for all structures to show the probable location of unpaired electron(s) in the acridine structures. The Mulliken spin density term was used for all structures and is shown for each carbon and nitrogen atom of the acridine in Figures S8 and S9. The blue surface indicates the positive phase, while green indicates the negative phase of the spin density.

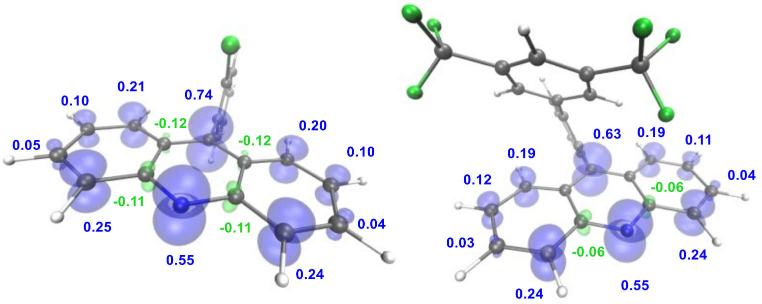

A1 A2

Figure S8. Spin densities for structures **A1** and **A2**.

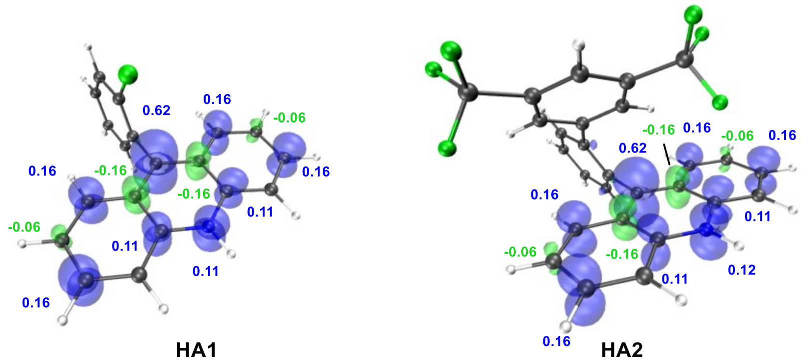

Figure S9. Spin densities for structures **HA1** and **HA2**.

Calculation of redox potentials

The standard potential of the R/O couple **HA1/HA1⁺** (E_{red} = –0.58 V vs SCE) and **HA2/HA2⁺** (E_{red} = –0.60 V vs SCE) was calculated by taking the absolute difference in free energies of the two optimized structures after the single point calculations at the ωB97X-D / 6- 311++G** / SMD (MeCN) level of theory, converting the free energy value to units of eV, and subtracting the value of the absolute potential of the saturated calomel electrode (SCE) in acetonitrile.[18]

$$E^{\ominus,SCE}_{0/R,MeCN} = E^{\ominus,abs}_{0/R,MeCN} - 4.43\,V$$

Energy Decomposition Analysis

The second generation Absolutely Localized Molecular Orbital Energy Decomposition Analysis (ALMO-EDA2) method of Head-Gordon and co-workers[19] was employed to gain quantitative insight into the intermolecular forces governing the interaction energies of for the transition states **TS2-*syn***, **TS2-*anti***, **TS1-*syn***, and **TS1-*anti***. ALMO-EDA2 calculations[20] were performed at the ωB97X-D / def2-SVP / SMD (MeCN) level of theory in Q-Chem[21] using the previously optimized geometries. Fragment definition for structures was defined as a singlet cyclohexane and the remaining acridine moiety calculated as a triplet.

Table S7. Energy decomposition analysis for **TS2**, kcal/mol.

Structure	Prep	ΔE_{Solv}	ΔE_{CT}	ΔE_{Elec}	ΔE_{Disp}	ΔE_{Pol}	ΔE_{Pauli}	Total $\Delta E^{\neq}_{int}$
**TS2-*syn***	0	2.5	–1.1	–41.1	–26.3	–7.8	110.2	–3.6
**TS2-*anti***	0	2.0	–37.5	–39.7	–18.4	–7.9	104.6	–3.1

Table S8. Tabulated energy decomposition analysis for **TS1**, kcal/mol.

Structure	Prep	ΔE_{Solv}	ΔE_{CT}	ΔE_{Elec}	ΔE_{Disp}	ΔE_{Pol}	ΔE_{Pauli}	Total $\Delta E^{\neq}_{int}$
**TS1-*syn***	0	2.2	–39.9	–40.5	–19.3	–7.9	107.1	1.7
**TS1-*anti***	0	2.0	–38.4	–39.9	–18.5	–7.8	105.3	2.7

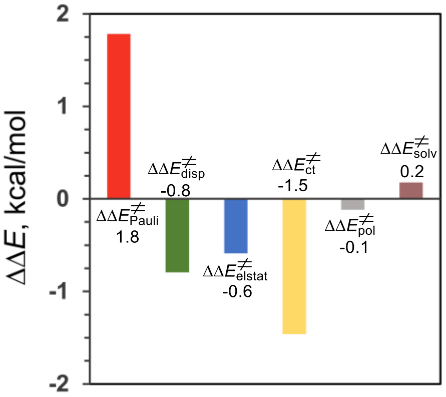

Figure S10. Energy decomposition analysis for **TS1**. $\mathbf{\Delta\Delta E^{\neq} = \Delta E^{\neq}_{TS1\text{-}\mathit{syn}} - \Delta E^{\neq}_{TS1\text{-}\mathit{anti}}}$, kcal/mol.

Complementary occupied–virtual orbital pairs (COVP) analysis

To gain insight into the dominant donor/acceptor orbital interactions giving rise to ΔE_{CT}, complementary occupied–virtual orbitals pairs (COVP) analysis was performed in tandem with ALMO-EDA2 calculation in Q-Chem. The COVPs were calculated for both the **TS2-*syn*** and **TS2-*anti***. The COVPS that contribute most to charge transfer were rendered in VMD and shown in Figure S11. Additionally, COVPs were identified between the *ortho* substituents and the cyclohexane moieties for **TS2-*syn***, Figure S13. These charge transfer interactions are not observed in **TS2-*anti***.

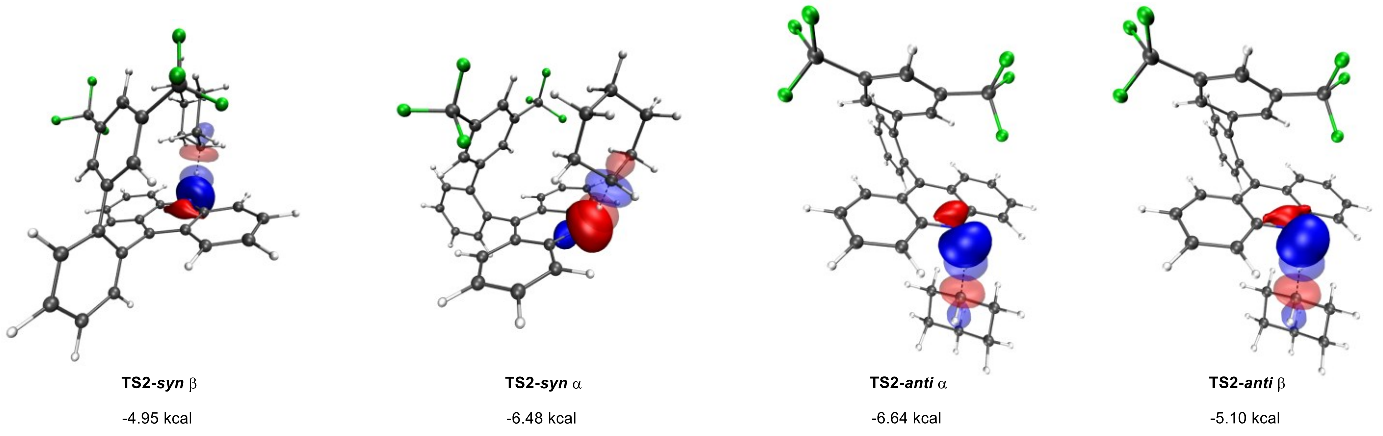

Figure S11. COVPs for charge transfer interactions in the alpha and beta space for **TS2-*syn*** and **TS2-*anti***. Donor COVPs are shown as an opaque surface while acceptor COVPs are represented as a transparent surface. COVPs indicate charge transfer between the lone pair on the acridine nitrogen atom to the C–H σ* in the cyclohexane fragment.

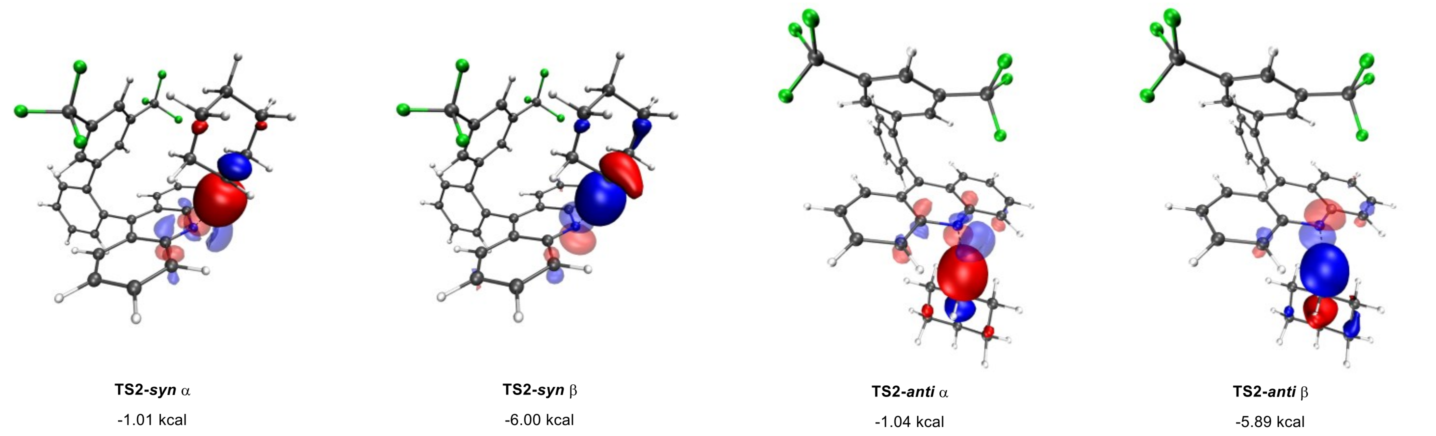

Figure S12. COVPs for additional charge transfer interactions in the alpha and beta space for **TS2-*syn*** and **TS2-*anti***. Donor COVPs are shown as an opaque surface while acceptor COVPs are represented as a transparent surface. COVPs indicate charge transfer between the C–H σ bond of cyclohexane moiety to π* on acridine.

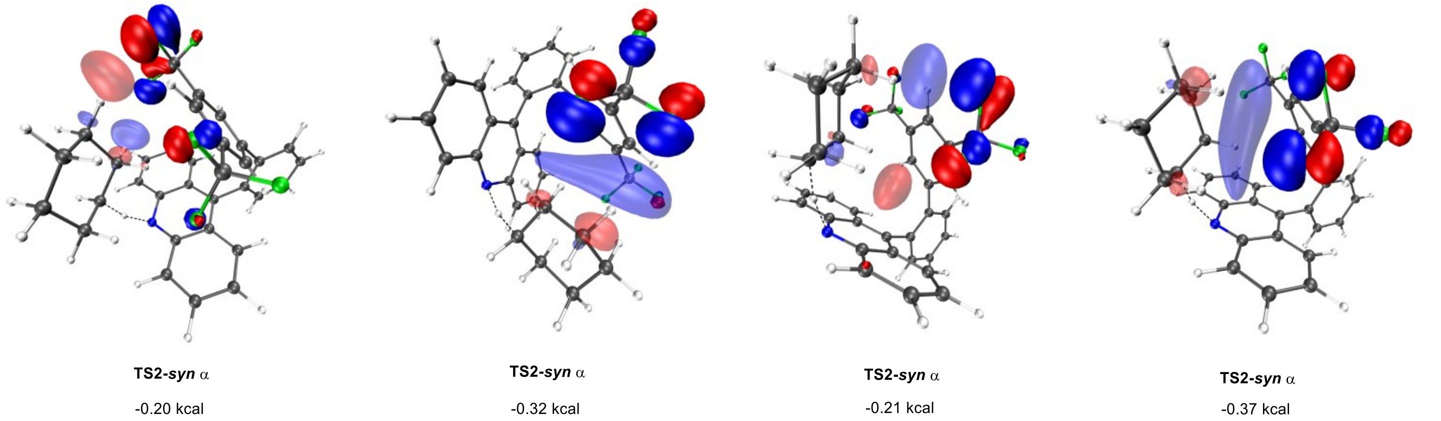

Figure S13. Additional COVPs that are specific to **TS2-*syn*** and not observed in **TS2-*anti***. Donor COVP is shown as an opaque surface while acceptor COVP is represented as a transparent surface. COVPs indicate charge transfer from the lone pairs on fluorine in the trifluoromethyl substituents to C–H σ* on cyclohexane moiety.

The COVPs for the **TS2-*syn*** and **TS2-*anti*** transition states indicate that **TS2-*syn*** has more favorable contribution to charge transfer. Interestingly, the COVPs that contribute most to charge transfer shows the **TS2-*anti*** transition state to be favored by 0.2 kcal/mol. However, **TS2-*syn*** includes additional interactions from the trifluoromethyl substituents. These interactions are not observed in **TS2-*anti***.

Noncovalent interaction analysis

To identify noncovalent interactions between the substituents in the acridine catalyst, an independent gradient model based on Hirshfeld partition (IGMH) for **TS1-*syn*** and **TS2-*syn*** transition state structures was implemented. The cube files were generated from the previously ωB97X-D / def2-SVP / SMD (MeCN) optimized geometries using Multiwfn[22] and exported and rendered in VMD. An isovalue of 0.002 was used to display the isosurface. The color of the isosurface signifies a specific interaction between the two fragments for each transition state. A green region represents Van der Waals (vdw) interaction, blue represents a strong attractive interaction, and red represents a strong repulsive interaction. Fragment definition for each transition was defined as the ortho substituent and the cyclohexane

moiety. Transition states for **TS1-*anti*** and **TS2-*anti*** were omitted from this analysis since the ortho substituent was distal from the cyclohexane moiety.

The results of the IGMH seems to agree with dispersion term for EDA. The phenyl group of the **A2** expands the interactions between the ortho substituent to the cyclohexane moiety compared to the **TS1-*syn*** transition state. The additional benzene ring allows for π interactions to occur which are absent from the chlorine substituent.

Marcus theory single electron transfer calculations The single electron transfer (SET) was approximated using Marcus theory as previously described.[23] The SET barrier was calculated for **HA2** and tabulated in Table S9. This calculation was performed for the reaction between **S6** and **HA2** as demonstrated below.

Table S9. Results of the Marcus theory calculations for SET between **S6** and **HA2**.

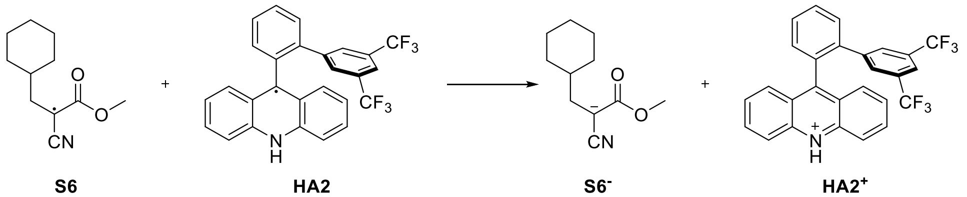

a_1, Å	a_2, Å	ε_{op}	ε	λ_0	ΔG_r	$\Delta G^{\ddagger}_{SET}$
5.58	4.34	1.81	35.69	18.1	–13.5	0.3

Excited state lifetime calculations

For structures **A1** and **A2**, the τ for singlet excited states were computed using the definition provided by Jaidane.[24] The oscillator strength and energy gap between ground state and each excited states (in cm^{-1}) were collected from the TD-DFT optimized geometries at the wB97X-D / 6-311+G** / SMD (MeCN) level of theory. The lifetime for singlet excited states for **A1** and **A2** were found to be in the nanoseconds range and demonstrate a slightly longer life span in excited **A2**. (Tables S10 and S11).

Table S10. Calculated lifetime for the singlet excited states of **A1**.

Transition	ΔE (cm^{-1})	Oscillator strength	$f\Delta E^2$ (x10^8)	τ (ns)
**S1**	24197	0.29	1.70	8.83
**S2**	31294	0.36	3.53	4.25

Table S11. Calculated lifetime for the singlet excited states of **A2**.

Transition	ΔE (cm^{-1})	Oscillator strength	$f\Delta E^2$ (x10^8)	τ (ns)
**S1**	23713	0.30	1.69	8.89
**S2**	31052	0.31	2.99	5.01

Spin-orbit coupling calculations

The spin-orbit-coupling constants (SOCC) for structures **A1** and **A2** were calculated in Q-Chem. The calculations were performed at the ωB97X-D/def2-SVP/SMD (MeCN) level of theory using the previously optimized structures. The resulting SOCC values for the lowest energy triplet states (T_1/S_0) of **A1** and **A2** were 0.87 and 0.82 cm^{-1}, which is consistent with the results of the hole–electron analysis, indicating that C9-substituents are not involved in the excitation transitions (Figures S4–S7).

Coordinates of the Representative Optimized Geometries

1

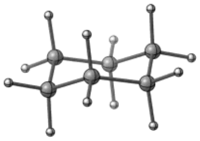

E(RωB97XD) = -235.652954481

Charge = 0 Multiplicity = 1

C	-5.1303829984	-1.1784158846	-2.4402623784
C	-4.9114353172	0.2069259744	-3.0521118320
C	-4.1001065481	-2.1867940639	-2.9531674845
H	-6.1437295061	-1.5327298626	-2.7031973954
C	-4.8979662251	0.1483174512	-4.5810495162
H	-3.9447227181	0.6089099731	-2.6979570798
H	-5.6885856551	0.9061972813	-2.7029522495
C	-4.0866411312	-2.2454065389	-4.4821049871
H	-3.0971217818	-1.8916853699	-2.5946883178
H	-4.3014247192	-3.1860703902	-2.5339283396
C	-3.8676952433	-0.8600652744	-5.0939607673
H	-4.6966476672	1.1475941691	-5.0002882567
H	-5.9009523355	-0.1467901004	-4.9395240444
H	-3.3094957783	-2.9446811349	-4.8312692082
H	-5.0533587390	-2.6473847139	-4.8362519866
H	-3.9040379919	-0.9178045269	-6.1940554254
H	-2.8543478682	-0.5057527536	-4.8310319096
H	-5.0940560231	-1.1206806085	-1.3401663884

^{3}A1

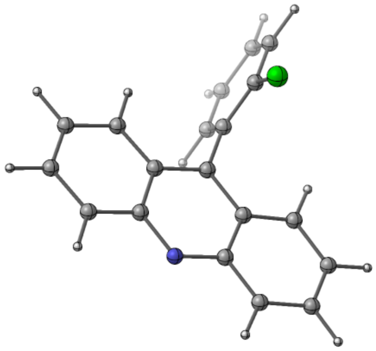

E(UωB97XD) = -1245.19409231

Charge = 0 Multiplicity = 3

C	-3.3473412204	0.1688406519	1.5222166420
C	-2.1111824683	0.1460717951	0.8685959634
C	-1.7725267116	1.2193187949	-0.0280222327
C	-2.6877993087	2.2535095482	-0.2070627818
C	-3.9327020420	2.2557571898	0.4663637184
C	-4.2615317054	1.2237037686	1.3216862611
H	-3.5842317830	-0.6567985313	2.1967530963
H	-2.4429973540	3.0792703685	-0.8787884211
H	-4.6276093428	3.0819522403	0.3016823252
H	-5.2198232834	1.2175041719	1.8449326808
C	-0.4913297152	1.1757800025	-0.6903111444
C	-0.0564965513	-0.9485542358	0.4989043999
C	0.3899450086	0.0648380302	-0.4189778431
C	0.7818587876	-2.0331434611	0.7770518032
C	2.0520664442	-2.1478418032	0.1766699979
H	2.6875325539	-3.0041362910	0.4115050393
C	2.4791473621	-1.1741984555	-0.7040487298
H	3.4600548012	-1.2477717095	-1.1784261045
C	1.6472087387	-0.0690731540	-1.0018678327
H	2.0004809893	0.6907470050	-1.7026870395
N	-1.2747141717	-0.9065136663	1.1195320894
C	-0.0941163158	2.2437733570	-1.6393310743
C	0.6902220638	3.3345453222	-1.2393853869
C	-0.4985850790	2.1905134807	-2.9803520838
C	1.0661656228	4.3367135473	-2.1317777786
C	-0.1315526009	3.1843548802	-3.8848296276
H	-1.1118631943	1.3481964364	-3.3092286879
C	0.6516707387	4.2570188090	-3.4595106861
H	1.6773853800	5.1718475389	-1.7854451984
H	-0.4580541079	3.1200823389	-4.9248523691
H	0.9441381233	5.0406398459	-4.1617200412
H	0.4222590135	-2.7912740278	1.4761463468
Cl	1.2113403276	3.4500282120	0.4263166998

TS1-*anti*

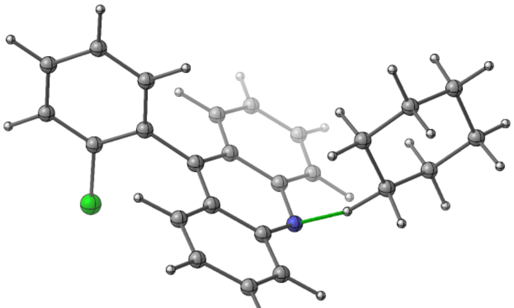

E(UωB97XD) = -1480.82795829

Charge = 0 Multiplicity = 3

C	-3.4938711339	0.1432012234	1.4020402236
C	-2.1452844060	0.1209277253	1.0407944659
C	-1.6890107117	0.9251069830	-0.0459980975
C	-2.6258463847	1.7358932700	-0.7143627452
C	-3.9726649377	1.7367583384	-0.3507162798
C	-4.4092345597	0.9388184690	0.7046191849
H	-3.8188371759	-0.4684199463	2.2461368592
H	-2.2868495334	2.3739397360	-1.5335942313
H	-4.6790889959	2.3704681448	-0.8914537702
H	-5.4603566347	0.9404403247	1.0014811061
C	-0.2996239893	0.8679021500	-0.4126667755
C	0.0720926025	-0.7583565838	1.4022674660

C	0.5970477342	0.0268569939	0.3328046514
C	0.9123201970	-1.6249907371	2.1042694961
C	2.2693766471	-1.7244362465	1.7802758922
H	2.9104336761	-2.4078257425	2.3416336403
C	2.7993533423	-0.9404310838	0.7572475501
H	3.8615549621	-1.0012148622	0.5101888667
C	1.9727527578	-0.0695619866	0.0477477710
H	2.3939683941	0.5490870959	-0.7478519388
N	-1.2658499549	-0.6724557734	1.7664593203
C	0.1940246531	1.6251957825	-1.5905437840
C	0.6293022625	2.9537240212	-1.4916748586
C	0.2392345200	1.0210390385	-2.8546257236
C	1.0908862391	3.6626591486	-2.5995445159
C	0.6978705775	1.7145629144	-3.9722095284
H	-0.0952188701	-0.0148354707	-2.9507891212
C	1.1235028264	3.0365132667	-3.8435838018
H	1.4216810699	4.6962448375	-2.4844018757
H	0.7225660583	1.2212440343	-4.9461372861
H	1.4843957286	3.5884289213	-4.7142104858
H	0.4894339276	-2.2220995118	2.9151811052
Cl	0.5962157590	3.7528732533	0.0643798465
C	-2.0536685563	-3.1166390297	1.3536665362
C	-1.6396302551	-3.2137049033	-0.0979985352
C	-2.0534481697	-4.5734702803	-0.6821854981
C	-3.5442442011	-4.8398401180	-0.4707536095
C	-3.9328516133	-4.7240422203	1.0033126488
C	-3.5295958507	-3.3553137929	1.5787802024
H	-1.7598273863	-1.9637159827	1.7506193985
H	-1.4160331963	-3.7234474657	2.0192444291
H	-0.5537352639	-3.0655198979	-0.2049122968
H	-2.1375221314	-2.4100885734	-0.6705625365
H	-1.8011694631	-4.6099984848	-1.7539851358
H	-1.4675135913	-5.3693424303	-0.1896769004
H	-3.8086864694	-5.8381645832	-0.8551045999
H	-4.1298875374	-4.1096276235	-1.0575120758
H	-5.0168583780	-4.8735935693	1.1313986139
H	-3.4301542711	-5.5211265342	1.5787005130
H	-3.7798717743	-3.2960019269	2.6492258154
H	-4.1112605387	-2.5735223113	1.0604104047

TS1-*syn*

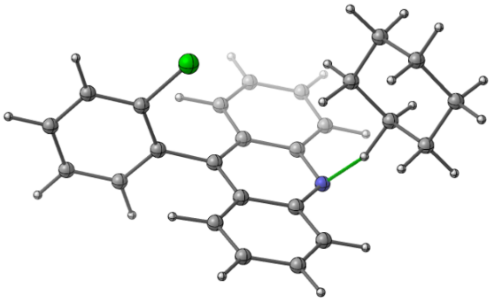

E(UωB97XD) = -1480.82911996

Charge = 0 Multiplicity = 3

C	-3.4029259305	0.2643813317	1.8588543824
C	-2.1284872419	0.2061122125	1.2913714941
C	-1.7986982895	1.0633533001	0.2002393188
C	-2.7745456518	1.9711110898	-0.2537666155
C	-4.0447939858	2.0148706615	0.3202666300
C	-4.3625081540	1.1584321639	1.3727592468
H	-3.6365698579	-0.4032485313	2.6911249774
H	-2.5273412651	2.6483135545	-1.0744257206
H	-4.7855099619	2.7236412420	-0.0565545717
H	-5.3543281825	1.1881119637	1.8293703874
C	-0.5012050792	0.9414862932	-0.4073013658
C	0.0462747882	-0.8344754348	1.2114216134
C	0.4383490672	-0.0157533422	0.1105544784
C	0.9318032935	-1.7881409916	1.7167319690
C	2.1969362561	-1.9649375319	1.1458556749
H	2.8737984744	-2.7189223797	1.5537921842
C	2.5913123281	-1.1694111448	0.0723668751
H	3.5807309709	-1.2953482867	-0.3728201574
C	1.7239282712	-0.2004582321	-0.4328089901
H	2.0430113411	0.4295995773	-1.2660377176
N	-1.2018798556	-0.6932723141	1.8060971908
C	-0.1524632619	1.7642740869	-1.5926125955
C	-0.4670438442	1.3532121250	-2.8954916143
C	0.5091265429	2.9907625868	-1.4426522201
C	-0.1429315826	2.1240787238	-4.0103988156
C	0.8413531769	3.7735809181	-2.5462609761
H	0.7633365702	3.3275467415	-0.4347515423
C	0.5143071502	3.3393250117	-3.8304475878
H	-0.4043234054	1.7720345150	-5.0097926578
H	1.3565775085	4.7255897610	-2.4021701197
H	0.7703562531	3.9462841417	-4.7015741637
H	0.6177961225	-2.3924335534	2.5702820379
Cl	-1.2900094893	-0.1719429755	-3.1420215053
C	-2.4034940926	-2.9429166962	1.3174761191
C	-1.6295992946	-4.1821537581	1.7052904023
C	-2.2791550082	-5.4249348557	1.0717128370
C	-2.4062029088	-5.2704077140	-0.4440245173
C	-3.1721503679	-4.0009351885	-0.8177259277
C	-2.5374455510	-2.7567736621	-0.1774707321
H	-1.8088095611	-1.9296278864	1.7664352353
H	-3.3677512522	-2.8554578227	1.8468849224
H	-1.5763700610	-4.2880776371	2.7997891836
H	-0.5945166348	-4.1002618226	1.3309539996
H	-1.6863011855	-6.3192467289	1.3218888862
H	-3.2814838112	-5.5730328954	1.5105055709
H	-2.9041395757	-6.1540865653	-0.8747166131
H	-1.3958956189	-5.2280314987	-0.8889480988

H	-3.2084855696	-3.8773466730	-1.9119221393
H	-4.2171085320	-4.0901375555	-0.4721264062
H	-3.1335518706	-1.8603792046	-0.4085961408
H	-1.5342538140	-2.6012486804	-0.6136784880

[3]A2

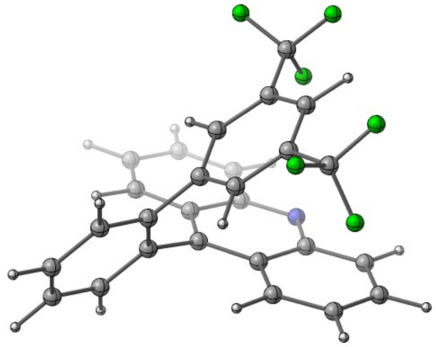

E(UωB97XD) = -1689.96798870

Charge = 0 Multiplicity = 3

C	-2.9544151454	0.2707188051	2.0229541863
C	-1.7733128939	0.1654623561	1.2783806187
C	-1.5677572031	1.0417615274	0.1577683729
C	-2.5798819997	1.9374673293	-0.1750645207
C	-3.7647065371	2.0299078863	0.5916763310
C	-3.9485655402	1.2093533355	1.6870579948
H	-3.0801062768	-0.3987843668	2.8765792624
H	-2.4582083799	2.5920204177	-1.0404029616
H	-4.5265642923	2.7598081794	0.3104394932
H	-4.8574223758	1.2771279006	2.2883434079
C	-0.3106598374	0.9619276939	-0.5581624244
C	0.3036079728	-0.8988594573	0.9574870479
C	0.6375215772	-0.0581496449	-0.1608720000
C	1.2167019903	-1.8707270170	1.3811832527
C	2.4579692763	-2.0380537012	0.7381273397
H	3.1527113628	-2.8042739661	1.0879841831
C	2.7863410813	-1.2197841017	-0.3246315978
H	3.7484140501	-1.3266553564	-0.8302166896
C	1.8805432937	-0.2278726877	-0.7666285440
H	2.1698328617	0.4171538077	-1.5981575487
N	-0.8651855046	-0.7815148525	1.6578372242
C	0.0240140369	1.9514182538	-1.6105343131
C	0.1929769907	3.3182874970	-1.2906428264
C	0.1498979777	1.5491302344	-2.9477135206
C	0.4543939688	4.2396969341	-2.3101757623
C	0.4231084913	2.4741852782	-3.9529181719
H	0.0139487027	0.4946687786	-3.1997338900
C	0.5727251524	3.8234477921	-3.6345176129
H	0.5810652438	5.2949141703	-2.0554770787
H	0.5116712275	2.1397186101	-4.9889861025
H	0.7846318629	4.5543838729	-4.4179667893
H	0.9401678824	-2.4910215861	2.2364381113
C	0.0550216509	3.7690821388	0.1206455732
C	-0.8865868439	4.7419482038	0.4617767209
C	0.7947212504	3.1611723587	1.1391138420
C	-1.1100087044	5.0695475206	1.7991571508
C	0.5616220671	3.4899378883	2.4726952653
C	-0.3972592491	4.4402402057	2.8158425328
H	-1.4785100703	5.2168938118	-0.3240881113
H	1.5496818657	2.4135682035	0.8877155182
H	-0.5870065072	4.6844680580	3.8608822315
C	1.3325473168	2.7541309040	3.5365490375
F	2.6499197570	2.7702444796	3.2968886231
F	1.1447537592	3.2687809349	4.7540844546
F	0.9710193914	1.4628580893	3.5959910308
C	-2.1889059075	6.0695576305	2.1207520433
F	-3.4021933992	5.6007201957	1.7907825890
F	-2.2292771935	6.3821828468	3.4179938193
F	-2.0244532006	7.2125586077	1.4420362076

TS2-*anti*

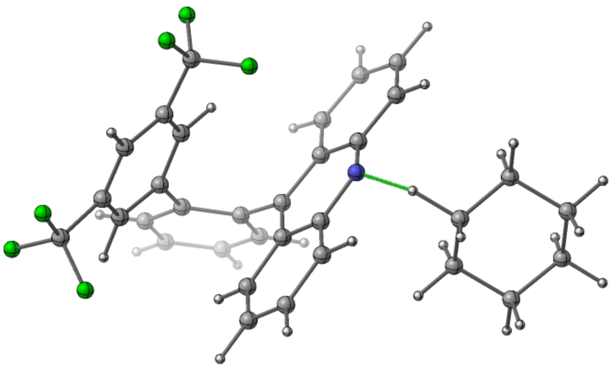

E(UωB97XD) = -1925.60119989

Charge = 0 Multiplicity = 3

C	-3.0495169339	0.2198346863	1.9518216117
C	-1.8332495278	0.1609602840	1.2661037585
C	-1.5895967926	1.0409170949	0.1718505035
C	-2.6053373379	1.9490805637	-0.1839787745
C	-3.8156687162	1.9961315812	0.5061127395
C	-4.0395905090	1.1315894382	1.5757777519
H	-3.2096667450	-0.4595022877	2.7917529357
H	-2.4399361648	2.6316007131	-1.0196092351
H	-4.5806726957	2.7162475512	0.2079690495
H	-4.9814918083	1.1658818645	2.1277722811
C	-0.3225091813	0.9665919182	-0.5143133030
C	0.3126027169	-0.8985964003	0.9752179653
C	0.6335139751	-0.0354166682	-0.1146910817
C	1.2258207312	-1.8729219869	1.3852480394
C	2.4562197522	-2.0198106103	0.7380525467

H	3.1555503470	-2.7890936975	1.0728811264
C	2.7940246810	-1.1643369466	-0.3083739011
H	3.7633763652	-1.2544341442	-0.8035267548
C	1.8991424027	-0.1755092698	-0.7167924767
H	2.1851445291	0.5073628649	-1.5188905798
N	-0.8745593221	-0.7537287928	1.6792025782
C	0.0057843600	1.9423541917	-1.5857950924
C	0.1966096863	3.3087270008	-1.2794219038
C	0.1021223281	1.5302169420	-2.9216646340
C	0.4514896166	4.2208355226	-2.3092563209
C	0.3710424260	2.4444441632	-3.9382675944
H	-0.0497964834	0.4753181791	-3.1631659960
C	0.5431363308	3.7940253998	-3.6323775393
H	0.5929961792	5.2765205819	-2.0642121132
H	0.4386246654	2.1017290544	-4.9732985586
H	0.7508865840	4.5167542569	-4.4245789325
H	0.9657357252	-2.5071425176	2.2349048858
C	-2.1588506351	-3.0074145681	1.4153795052
C	-1.3110812220	-4.2390869805	1.6405311256
C	-2.0733696880	-5.4916230071	1.1747978492
C	-2.5232441439	-5.3554434652	-0.2799702639
C	-3.3571735932	-4.0920193503	-0.4954081778
C	-2.6076488191	-2.8376559886	-0.0192608791
H	-1.4973064867	-1.9906131139	1.7287108183
H	-2.9932877970	-2.9283737390	2.1332348077
H	-1.0239368564	-4.3321534014	2.6993089677
H	-0.3804093203	-4.1575783635	1.0527196271
H	-1.4351876238	-6.3807452011	1.3014488126
H	-2.9572844038	-5.6381816337	1.8200486776
H	-3.0984474946	-6.2449665550	-0.5836704390
H	-1.6320911884	-5.3173429346	-0.9317786181
H	-3.6278348733	-3.9825452542	-1.5577250306
H	-4.3034971548	-4.1795002177	0.0668798676
H	-3.2466755001	-1.9474308630	-0.1260123349
H	-1.7210192447	-2.6826839005	-0.6604673526
C	0.0780131432	3.7694270976	0.1307541538
C	-0.8626512452	4.7405342011	0.4792102850
C	0.8348873523	3.1716293718	1.1428328425
C	-1.0672763198	5.0775011822	1.8175794516
C	0.6198516617	3.5084982620	2.4771360687
C	-0.3374225800	4.4581252527	2.8278328807
H	-1.4678970701	5.2075640576	-0.3012869264
H	1.5879683548	2.4242228690	0.8853643426
H	-0.5129543928	4.7087694710	3.8738350983
C	1.4063131927	2.7799757174	3.5342285474
F	2.7209429592	2.8003374265	3.2794020055
F	1.2309402347	3.2982582624	4.7523247678
F	1.0508348880	1.4874284289	3.6028027755
C	-2.1439627580	6.0770168599	2.1469222383
F	-3.3585066776	5.6121354879	1.8163504974
F	-2.1815587315	6.3826769922	3.4460582466
F	-1.9792071506	7.2240270665	1.4744727808

TS2 -*syn*

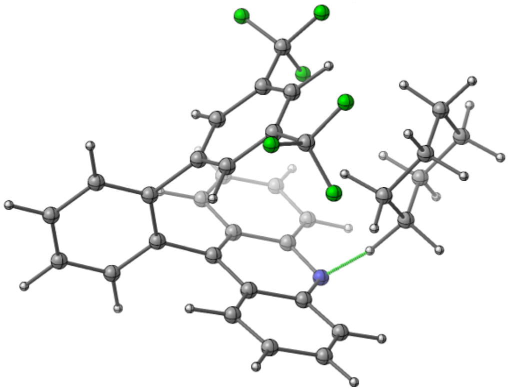

E(UωB97XD) = -1925.60890547

Charge = 0 Multiplicity = 3

C	-2.6212574593	0.3569891127	1.8690525849
C	-1.3419773065	0.3535456212	1.3085330868
C	-1.0431892797	1.2255602234	0.2183814606
C	-2.0588535968	2.0854649782	-0.2429566474
C	-3.3366895846	2.0637973802	0.3146006977
C	-3.6211655163	1.1961614117	1.3672578607
H	-2.8259065410	-0.3085545776	2.7096395770
H	-1.8359225836	2.7759807711	-1.0592748869
H	-4.1081831802	2.7332000353	-0.0722732294
H	-4.6172706076	1.1774559238	1.8149208362
C	0.2607772961	1.1634234006	-0.3817246520
C	0.8846570118	-0.5717606753	1.2562817050
C	1.2410830881	0.2551171394	0.1496330053
C	1.8065597784	-1.4909499174	1.7624554210
C	3.0762337629	-1.6199272173	1.1912211009
H	3.7806084369	-2.3490443076	1.5977537637
C	3.4394621423	-0.8117055523	0.1155663366
H	4.4328181657	-0.9004348535	-0.3298112210
C	2.5350573211	0.1212408668	-0.3907474352
H	2.8294169282	0.7619124364	-1.2247055434
N	-0.3699769363	-0.4808777815	1.8418953846
C	0.5743254866	1.9586476914	-1.5990992893
C	0.3472365818	1.4108943597	-2.8757429919
C	1.1247535948	3.2417530915	-1.4963471462
C	0.6829004001	2.1456195019	-4.0171859307
C	1.4499699320	3.9719310213	-2.6386532004
H	1.3027038434	3.6663998641	-0.5052294813
C	1.2319479959	3.4218886662	-3.9012056276
H	0.5071532760	1.7115800438	-5.0046213284
H	1.8783524146	4.9721486501	-2.5411429019
H	1.4882134106	3.9875305713	-4.7999276445

H	1.5113300392	-2.1174294342	2.6071290438
C	-1.2492279174	-2.9302357112	1.5858305572
C	-0.7453149109	-3.2085223347	0.1876685977
C	-1.2356677320	-4.5817043199	-0.2937350317
C	-2.7558903979	-4.6937869776	-0.1913883986
C	-3.2459572940	-4.3916823770	1.2245015327
C	-2.7547246555	-3.0145459232	1.7033834766
H	-0.8830073075	-1.7737756365	1.8949430524
H	-0.7259341094	-3.5220638614	2.3565060206
H	0.3522969413	-3.1592327212	0.1419587670
H	-1.1332834844	-2.4297781380	-0.4920670072
H	-0.9020458531	-4.7533628764	-1.3282677912
H	-0.7669340488	-5.3679133373	0.3240711721
H	-3.0847797585	-5.6991414015	-0.5005320808
H	-3.2179293015	-3.9798796530	-0.8945025203
H	-4.3463036050	-4.4255447048	1.2693947385
H	-2.8737311053	-5.1688328077	1.9149145542
H	-3.0820970986	-2.8276579915	2.7377682767
H	-3.2140414718	-2.2389579550	1.0674245970
C	-0.2532941032	0.0491709417	-2.9990926315
C	0.5521366468	-1.0721185400	-3.2087611073
C	-1.6346425247	-0.1237876242	-2.8850195817
C	-0.0202011171	-2.3408484912	-3.3062132032
C	-2.1972309701	-1.3954582404	-2.9794105794
C	-1.3973492765	-2.5135819176	-3.1984060695
H	1.6346621760	-0.9504732864	-3.2891640175
H	-2.2736339709	0.7450676052	-2.7152107886
H	-1.8389117637	-3.5075043750	-3.2685844197
C	-3.6824234395	-1.5359987695	-2.7791328618
F	-4.1279781563	-2.7606878253	-3.0728544361
F	-4.0274472137	-1.2879010941	-1.5045775108
F	-4.3727901928	-0.6719550962	-3.5331776283
C	0.8903624742	-3.5275777165	-3.4764404681
F	1.7726664247	-3.3411230106	-4.4656963342
F	1.6074401634	-3.7574095389	-2.3648849576
F	0.2223247965	-4.6523772862	-3.7492109862

HA2

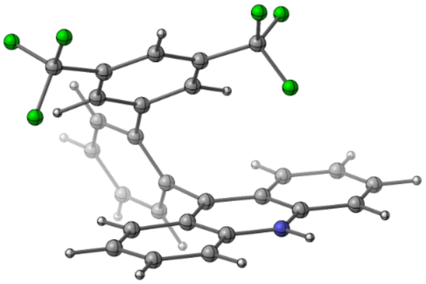

E(UωB97XD) = -1690.64306855

Charge = 0 Multiplicity = 2

C	-3.0365411672	0.2975467320	1.9546510030
C	-1.8421037371	0.2004637838	1.2292554474
C	-1.5964154192	1.0503092083	0.1133116423
C	-2.6168479831	1.9646850864	-0.2428802605
C	-3.7965113766	2.0577599237	0.4813522606
C	-4.0061925161	1.2255840000	1.5881394718
H	-3.1903961140	-0.3621891242	2.8122426868
H	-2.4637289023	2.6136344920	-1.1071881384
H	-4.5583352723	2.7826263445	0.1866298473
H	-4.9311584706	1.2966585356	2.1647395337
C	-0.3373900760	0.9645472724	-0.5709049338
C	0.3292353034	-0.8581607457	0.9509084907
C	0.6282927519	-0.0126192994	-0.1565348606
C	1.2575926649	-1.8052992201	1.4011940323
C	2.4954227800	-1.9261358482	0.7779208036
H	3.2124918858	-2.6667913683	1.1388440533
C	2.8201312529	-1.0928277900	-0.2999541394
H	3.7954724048	-1.1772274134	-0.7842764590
C	1.9049047469	-0.1532827268	-0.7532978012
H	2.1712841402	0.4975327175	-1.5879063317
N	-0.8809341954	-0.7154450786	1.5891540234
C	-0.0028942827	1.9493723102	-1.6358014863
C	0.2019099411	3.3109011169	-1.3164459537
C	0.0881413206	1.5518078123	-2.9761552829
C	0.4656978109	4.2314471341	-2.3366717772
C	0.3681501603	2.4731888136	-3.9836923098
H	-0.0750048771	0.5011499021	-3.2289120631
C	0.5543320520	3.8176523878	-3.6642057122
H	0.6161579040	5.2833679732	-2.0808089826
H	0.4326618921	2.1402829670	-5.0221815602
H	0.7697250506	4.5463894672	-4.4488753087
H	0.9965238170	-2.4383387334	2.2531620431
C	0.0827603036	3.7605955236	0.0974823813
C	-0.8580987470	4.7288629840	0.4525313574
C	0.8382852255	3.1549371871	1.1060526126
C	-1.0635503239	5.0567233569	1.7934116774
C	0.6202921050	3.4799056257	2.4426144335
C	-0.3367567689	4.4278908185	2.7996341488
H	-1.4622905198	5.2019795956	-0.3251107157
H	1.5907631490	2.4089151663	0.8432994463
H	-0.5140590400	4.6698578336	3.8473942539
C	1.3983738716	2.7371895690	3.4956790810
F	2.7125288816	2.7371487245	3.2387233871
F	1.2331298062	3.2539942679	4.7158507402
F	1.0227816685	1.4493674830	3.5618157543
C	-2.1384249285	6.0558008536	2.1291156504
F	-3.3542723757	5.5948422959	1.7973061684
F	-2.1744749457	6.3544546019	3.4300331428
F	-1.9728183427	7.2063332959	1.4628470450
H	-1.0653034935	-1.3069021666	2.3931095931

S19

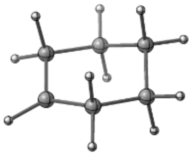

E(UωB97XD) = -234.984380043

Charge = 0 Multiplicity = 2

C	-4.6085953471	-1.7110008679	-2.5583415240
C	-5.9715754852	-1.1003663789	-2.6027313278
C	-3.5994681065	-1.2984371413	-3.5800755313
H	-4.2719545635	-2.2052390830	-1.6410826297
C	-6.5273171453	-1.0220146800	-4.0319577152
H	-5.9201308340	-0.0657162151	-2.1962946340
H	-6.6673349314	-1.6443088583	-1.9437517411
C	-4.2005925620	-1.2168207930	-4.9905640130
H	-3.2042147431	-0.2923861427	-3.3155527364
H	-2.7282409773	-1.9729637806	-3.5665224584
C	-5.5055606500	-0.4198459288	-4.9971155723
H	-7.4599812670	-0.4356149061	-4.0408005838
H	-6.7877603550	-2.0389776626	-4.3746306235
H	-3.4710394458	-0.7699624345	-5.6847497385
H	-4.4031776747	-2.2387681120	-5.3566947978
H	-5.9223121573	-0.3821784317	-6.0165526802
H	-5.2966917548	0.6250934167	-4.7027496927

S20

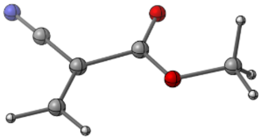

E(RωB97XD) = -398.284469893

Charge = 0 Multiplicity = 1

C	-3.9334447265	-0.6279618112	0.1303343025
H	-4.7589569152	-0.9544137170	0.7664933582
C	-2.7694864266	-1.2925598878	0.1053699826
C	-1.5956212680	-0.9024814568	-0.7424308087
C	-0.7881530044	0.6352946494	-2.3156255121
H	-0.5175086745	-0.1362952110	-3.0501028539
H	-1.1784486558	1.5213163284	-2.8276802734
H	0.0977860550	0.9024668696	-1.7224926041
C	-2.5660835225	-2.4631028200	0.9188651397
N	-2.4149551812	-3.4022829203	1.5769202546
O	-0.5570211587	-1.5152861823	-0.7461918887
O	-1.8391343082	0.1746833749	-1.4684918555
H	-4.0737482134	0.2579687841	-0.4917242411

TS3

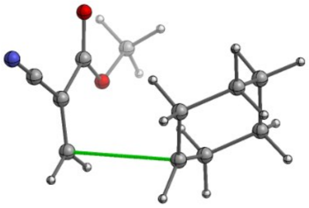

E(UωB97XD) = -633.277217371

Charge = 0 Multiplicity = 2

C	-3.7758652400	-0.6125789829	0.2061174672
H	-4.5793918959	-1.0719334138	0.7849824218
C	-2.6508416610	-1.2879110818	-0.0829457849
C	-1.5363918086	-0.7315905135	-0.9108046759
C	-0.7314555015	1.1790717785	-2.0079459718
H	-0.7428593689	0.7076040007	-3.0010672371
H	-1.0297920503	2.2299362733	-2.0878697918
H	0.2779824463	1.1053207534	-1.5796215312
C	-2.4831810580	-2.6551858813	0.3285356644
N	-2.3615267639	-3.7554379033	0.6662785654
O	-0.6186343607	-1.4000649621	-1.3191286463
O	-1.6882212597	0.5619792079	-1.1503141238
H	-3.8905998665	0.4242584694	-0.1131002673
C	-5.1829218599	-1.0688363162	-2.3978639665
C	-4.7295248949	0.1985316323	-3.0457598381
C	-4.5229203731	-2.3393198398	-2.8173908280
H	-6.0407122938	-1.0781955986	-1.7202961937
C	-4.6493104682	0.0525588201	-4.5765914470
H	-3.7122582481	0.4492410448	-2.6822398582
H	-5.3774747846	1.0436130017	-2.7673797926
C	-4.4472067697	-2.4567509781	-4.3510136844
H	-3.4796077049	-2.3554109306	-2.4370444398
H	-5.0252811994	-3.2160740067	-2.3811386172
C	-3.8531881715	-1.1917898724	-4.9714814658
H	-4.1961890558	0.9565018403	-5.0146849605
H	-5.6707235979	-0.0254322475	-4.9886251392
H	-3.8504328350	-3.3404087132	-4.6286919524
H	-5.4624509082	-2.6162611354	-4.7545702466
H	-3.8178825306	-1.2875771522	-6.0686990463
H	-2.8077072637	-1.0774592290	-4.6303201393

S21

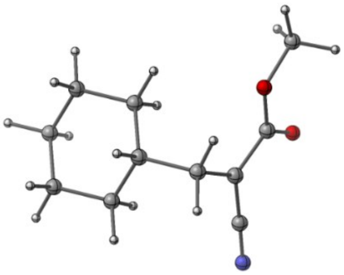

E(UωB97XD) = -633.336430923

Go to table of contents

Charge = 0 Multiplicity = 2

C	-0.8499718554	0.1591782551	1.0637706656
H	-1.4482109902	-0.4644387355	1.7458465594
C	0.5827197950	-0.2499421777	1.1699713567
C	1.7211885583	0.6839552959	1.0224561039
C	2.3386998894	2.9293456303	0.7470406739
H	2.8910271094	2.7441196909	-0.1852628653
H	1.8208957999	3.8928804665	0.6911395256
H	3.0406673479	2.9271577728	1.5930160798
C	0.9322444820	-1.5987661670	1.3748602827
N	1.1889043067	-2.7206963348	1.5533364128
O	2.8778888680	0.3268219743	0.9900243127
O	1.3247316361	1.9464569954	0.9320126074
H	-0.9284082402	1.2039079433	1.3944523675
C	-1.4529721210	0.0410229182	-0.3588725081
C	-0.7244163364	0.9036751665	-1.3948783179
C	-1.5572325844	-1.4084957185	-0.8445310299
H	-2.4818390818	0.4322986447	-0.2640310604
C	-1.4011047509	0.8233541159	-2.7638402394
H	0.3201223537	0.5530000440	-1.4904385386
H	-0.6751619102	1.9482982075	-1.0495750355
C	-2.2394849122	-1.4921305033	-2.2107193217
H	-0.5444494297	-1.8423723674	-0.9265659976
H	-2.1033109544	-2.0152619470	-0.1035395577
C	-1.5248682310	-0.6223661728	-3.2452277476
H	-0.8399942123	1.4254038176	-3.4964683152
H	-2.4090445323	1.2702750343	-2.6946032094
H	-2.2763494576	-2.5398765208	-2.5494689933
H	-3.2868689287	-1.1546460029	-2.1134059239
H	-2.0560964457	-0.6604816899	-4.2098789075
H	-0.5142151720	-1.0314426358	-3.4244623789

S21-

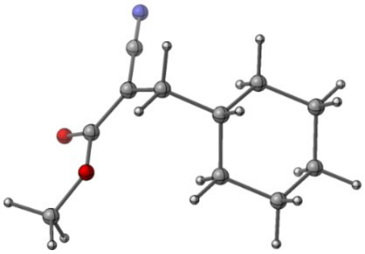

E(RωB97XD) = -633.490266019

Charge = -1 Multiplicity = 1

C	-0.8282197814	0.1574552972	1.0482499460
H	-1.4467190623	-0.4418955715	1.7409069565
C	0.6239026099	-0.2602261697	1.1800772134
C	1.7244859066	0.6363442189	1.0643086811
C	2.3599464910	2.8846271901	0.7225946862
H	2.9943394580	2.6648412744	-0.1514215766
H	1.8707327947	3.8572073502	0.5762390988
H	3.0099236451	2.9369949437	1.6109593947
C	0.9377164591	-1.6062066448	1.4037040265
N	1.1616605764	-2.7416709849	1.5996012208
O	2.9226805908	0.3649824569	1.1253925081
O	1.3317109423	1.9368556644	0.8762486693
H	-0.9194650765	1.2022632205	1.3845849761
C	-1.4667036141	0.0480185308	-0.3520100012
C	-0.7819477647	0.9367063893	-1.3960699922
C	-1.5414808781	-1.3985735733	-0.8553016015
H	-2.5096046166	0.4090569117	-0.2515383857
C	-1.4512781764	0.8380393654	-2.7678394513
H	0.2759629572	0.6303750767	-1.4833439330
H	-0.7701062631	1.9818899529	-1.0475005912
C	-2.2178571805	-1.5021055054	-2.2230035984
H	-0.5173564905	-1.8059351085	-0.9305121655
H	-2.0720473043	-2.0245802615	-0.1174429960
C	-1.5267191522	-0.6094306620	-3.2538619903
H	-0.9105805146	1.4595895144	-3.5006135767
H	-2.4751056554	1.2496278503	-2.7032378169
H	-2.2248864676	-2.5495762260	-2.5666434229
H	-3.2754833704	-1.1948257562	-2.1304579345
H	-2.0501787952	-0.6636204746	-4.2225526793
H	-0.5022332672	-0.9859932693	-3.4273566643

HA1

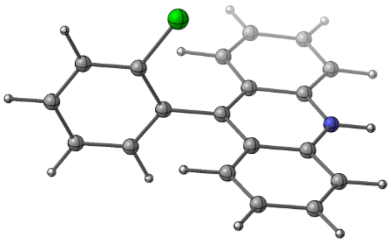

E(UωB97XD) = -1245.86972669

Charge = 0 Multiplicity = 2

C	-3.3702557190	0.2056326213	1.5505549593
C	-2.1370711647	0.1734482877	0.8881136159
C	-1.7652244542	1.2202424358	-0.0047340868
C	-2.6812524278	2.2846452744	-0.1839367044
C	-3.9006108124	2.3091127128	0.4781812909
C	-4.2481996200	1.2666593063	1.3468912909
H	-3.6302327971	-0.6116861337	2.2280091669
H	-2.4138134781	3.0998713593	-0.8597535592
H	-4.5878611041	3.1432517707	0.3203055770
H	-5.2071140793	1.2814415021	1.8697285172
C	-0.4965842248	1.1541991233	-0.6650554639
C	-0.0259835425	-0.9703830509	0.4877611274
C	0.3852913808	0.0549279864	-0.4124158812
C	0.8102722670	-2.0604380717	0.7573408897

Go to table of contents

C	2.0595913809	-2.1527148292	0.1500868247
H	2.7039346447	-3.0072328569	0.3685580655
C	2.4861193548	-1.1536578113	-0.7344390253
H	3.4669599932	-1.2246234689	-1.2097860062
C	1.6626647400	-0.0709030739	-1.0092660942
H	1.9989223472	0.7056746536	-1.6997624664
N	-1.2614997187	-0.8703826307	1.0870274328
C	-0.0995203263	2.2221763376	-1.6196782902
C	0.6220576540	3.3541036295	-1.2149458249
C	-0.4421664768	2.1275184884	-2.9757865535
C	0.9922752753	4.3541215465	-2.1129176853
C	-0.0811991455	3.1173286810	-3.8872919770
H	-1.0050317394	1.2530429124	-3.3113564510
C	0.6370887809	4.2315981480	-3.4543957512
H	1.5539040313	5.2207821910	-1.7599801154
H	-0.3614149825	3.0179075267	-4.9381230512
H	0.9250277077	5.0132808423	-4.1607288005
H	0.4674155351	-2.8330934916	1.4502556217
Cl	1.0769876031	3.5287858235	0.4655576732
H	-1.5407018829	-1.6095317421	1.7242887344

HA2⁺

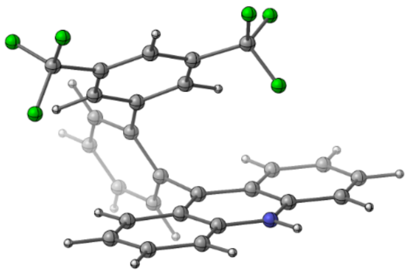

E(RωB97XD) = -1690.50651885

Charge = 1 Multiplicity = 1

C	-3.0731469784	0.2891011018	1.9183620954
C	-1.8547340724	0.2015970591	1.2012145543
C	-1.5972659913	1.0565466576	0.0906752130
C	-2.6177127006	1.9814300151	-0.2917125379
C	-3.7882098235	2.0580912545	0.4114067548
C	-4.0120753662	1.2083997887	1.5284702587
H	-3.2395590894	-0.3738363665	2.7693720885
H	-2.4496394032	2.6271301598	-1.1542960988
H	-4.5582671993	2.7723899403	0.1163046340
H	-4.9507707739	1.2850093196	2.0808761336
C	-0.3437440919	0.9792333464	-0.5568118453
C	0.2867993179	-0.8298729985	0.9604071132
C	0.6035808137	0.0183810344	-0.1411075605
C	1.2179943391	-1.7861477683	1.4333887882
C	2.4425410042	-1.8820811182	0.8255806738
H	3.1666922968	-2.6146567282	1.1876757246
C	2.7903362079	-1.0348824855	-0.2614837116
H	3.7776752334	-1.1247211618	-0.7172953206
C	1.9001349503	-0.1082327971	-0.7304850171
H	2.1723879520	0.5480189075	-1.5573199457
N	-0.9094122130	-0.6897251981	1.5720916612
C	-0.0062428683	1.9542161865	-1.6329109269
C	0.2151077136	3.3092190150	-1.3101349825
C	0.0561783168	1.5337963861	-2.9649630021
C	0.4792571450	4.2165717278	-2.3404051065
C	0.3267820637	2.4496810954	-3.9790560029
H	-0.1217027365	0.4831233806	-3.2049383764
C	0.5378660494	3.7915738018	-3.6660561604
H	0.6486529011	5.2671862617	-2.0932383730
H	0.3687319057	2.1123580002	-5.0166920760
H	0.7501727118	4.5130778576	-4.4578480691
H	0.9449427555	-2.4224616324	2.2773596362
C	0.1133221459	3.7618104340	0.1053635770
C	-0.8533292657	4.7008999700	0.4714261990
C	0.9032934806	3.1823843487	1.1020404603
C	-1.0536302046	5.0176504768	1.8145617563
C	0.6881074415	3.4953627205	2.4421602034
C	-0.2956123160	4.4094595064	2.8115177205
H	-1.4802495677	5.1558366801	-0.2989689378
H	1.6801277442	2.4651916983	0.8290924456
H	-0.4703797659	4.6420640783	3.8618431383
C	1.4947973366	2.7659918957	3.4839101004
F	2.8049689813	2.7988186992	3.2142033389
F	1.3269976687	3.2691302775	4.7080888632
F	1.1479154575	1.4690979536	3.5405260619
C	-2.1555843005	5.9839005219	2.1636855947
F	-3.3560617245	5.4975929317	1.8140004071
F	-2.2060678090	6.2540575889	3.4695450122
F	-2.0136929678	7.1499278703	1.5211599323
H	-1.1057336894	-1.2882040466	2.3760160466

A1

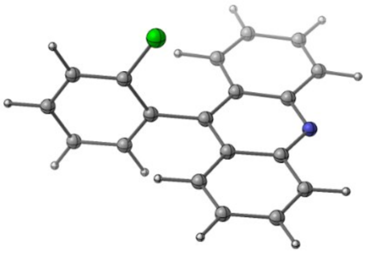

E(RωB97XD) = -1245.26769197

Charge = 0 Multiplicity = 1

C	-3.3489129904	0.1813455809	1.5447017514
C	-2.0826041084	0.1382662502	0.8731097394
C	-1.7396891299	1.2116770816	-0.0193324258
C	-2.6701376613	2.2871290273	-0.1985204726
C	-3.8653303634	2.2914053381	0.4635234990
C	-4.2095739753	1.2229663224	1.3456326950

H	-3.5921746328	-0.6447075601	2.2162424109
H	-2.4099435496	3.1061935509	-0.8721487151
H	-4.5669424241	3.1161595962	0.3210134137
H	-5.1709842961	1.2458350202	1.8640156376
C	-0.4956029362	1.1588024962	-0.6714145778
C	-0.0880701127	-0.9462974529	0.4895272315
C	0.3585464444	0.0704878761	-0.4229596949
C	0.7749126017	-2.0611770454	0.7513428697
C	1.9982714179	-2.1531434562	0.1510615977
H	2.6477840250	-3.0065406620	0.3594184568
C	2.4428532546	-1.1398246620	-0.7510581892
H	3.4256186991	-1.2317439991	-1.2185187024
C	1.6497296984	-0.0628925686	-1.0305612802
H	1.9929950848	0.7101562901	-1.7211440418
N	-1.2728714447	-0.8991414786	1.1062425263
C	-0.0952050911	2.2324896437	-1.6251504351
C	0.6114345780	3.3665157332	-1.2076257496
C	-0.4247353644	2.1251516523	-2.9815324758
C	0.9826178659	4.3667539185	-2.1044184190
C	-0.0606301646	3.1168759887	-3.8890748315
H	-0.9762608914	1.2450322040	-3.3201744830
C	0.6432713596	4.2375455364	-3.4492228641
H	1.5331345029	5.2386685767	-1.7472556419
H	-0.3277266804	3.0134182451	-4.9427084254
H	0.9327390516	5.0200035709	-4.1538713365
H	0.4167352027	-2.8244588497	1.4453410608
Cl	1.0437720306	3.5427502346	0.4772518722

A2

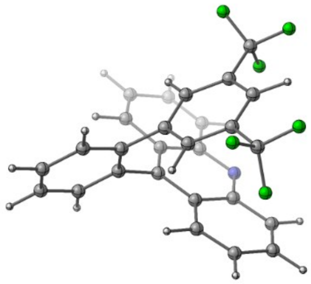

E(RωB97XD) = -1690.04029710

Charge = 0 Multiplicity = 1

C	-3.0839278582	0.2353615943	1.8883854321
C	-1.8377901952	0.1457546945	1.1841946810
C	-1.6002074481	1.0383172271	0.0839198832
C	-2.6284472838	1.9681043397	-0.2803901610
C	-3.8000393792	2.0270372067	0.4199980785
C	-4.0289307968	1.1510780170	1.5231488944
H	-3.2402913636	-0.4497893165	2.7242031196
H	-2.4642288570	2.6356990225	-1.1280999704
H	-4.5706360622	2.7469821306	0.1364961783
H	-4.9715816809	1.2148056752	2.0716504818
C	-0.3521005676	0.9789144920	-0.5655085180
C	0.2306460189	-0.8372372479	0.9583608830
C	0.5862599446	0.0207534447	-0.1389951480
C	1.1860997613	-1.8038436918	1.4158711835
C	2.4180360195	-1.8985245241	0.8347302156
H	3.1394573996	-2.6355137756	1.1951342432
C	2.7800054217	-1.0312542521	-0.2395152211
H	3.7746479055	-1.1130084991	-0.6831537511
C	1.8956722582	-0.1018722818	-0.7102731177
H	2.1826019522	0.5618613364	-1.5276106378
N	-0.9426800626	-0.7586670553	1.5923278099
C	-0.0117840094	1.9612628175	-1.6376800361
C	0.2163063281	3.3152352344	-1.3142200953
C	0.0510902837	1.5545015808	-2.9744515994
C	0.4851883619	4.2290004036	-2.3387576457
C	0.3309801482	2.4721953248	-3.9851710134
H	-0.1316603079	0.5060439069	-3.2216080331
C	0.5471843307	3.8121570060	-3.6668316477
H	0.6563250236	5.2781717613	-2.0856072502
H	0.3747984217	2.1389165365	-5.0243485592
H	0.7648367237	4.5368478182	-4.4544757393
H	0.8916473813	-2.4484284297	2.2466873061
C	0.1132146590	3.7676982314	0.1011861474
C	-0.8474909049	4.7128535064	0.4663269462
C	0.9035690756	3.1903591636	1.0990412726
C	-1.0389296081	5.0421319300	1.8082736991
C	0.7003501892	3.5182591721	2.4372686518
C	-0.2764767241	4.4407819595	2.8054098722
H	-1.4763912170	5.1654342934	-0.3038927619
H	1.6712517859	2.4629593143	0.8277131270
H	-0.4424128148	4.6842419908	3.8546771236
C	1.5165399548	2.8012587394	3.4797615454
F	2.8258134308	2.8419241111	3.2023528564
F	1.3536879734	3.3144496375	4.7013812549
F	1.1807626227	1.5034366636	3.5504678197
C	-2.1339386443	6.0159175474	2.1554949060
F	-3.3390087050	5.5360680010	1.8126900476
F	-2.1788556093	6.2947000394	3.4601327866
F	-1.9878612734	7.1785207284	1.5061335679

S22

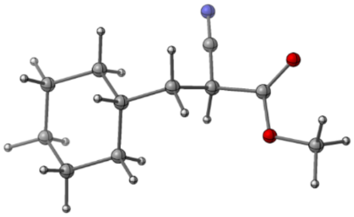

E(RωB97XD) = -633.981308954

Charge = 0 Multiplicity = 1

C	-0.0976321748	1.6531640508	0.3424128898
H	-1.0018595686	1.6031439704	0.9663474482
C	1.0522948071	1.0219767776	1.1721410861
C	0.9226738163	1.3804585207	2.6503489710
C	-0.4136064351	1.0447510873	4.5493894315
H	-0.6162701034	2.1149483711	4.6969151773
H	-1.3041091473	0.4569417918	4.7969335032
H	0.4278413435	0.7406232107	5.1876636952
C	2.3775788525	1.4248945939	0.6975292740
N	3.4149197699	1.7474114764	0.3040871746
O	1.6592579916	2.1178091235	3.2517913109
O	-0.1281969990	0.7720341107	3.1786911399
H	0.1291664307	2.7202677948	0.1924122240
C	-0.4083963081	0.9819667425	-1.0001890858
C	0.7523505224	0.9951243093	-2.0017661084
C	-0.9576206621	-0.4415095284	-0.8415061719
H	-1.2196507740	1.5911086467	-1.4398832107
C	0.3320076519	0.4133103352	-3.3522346450
H	1.5876010736	0.3907262548	-1.6056882835
H	1.1353822899	2.0217059170	-2.1245733107
C	-1.3736289587	-1.0387707620	-2.1868274123
H	-0.1834278512	-1.0876506971	-0.3891957476
H	-1.8119282469	-0.4371713257	-0.1444343610
C	-0.2269936567	-1.0013283250	-3.1975121681
H	1.1878964509	0.4132999664	-4.0461190087
H	-0.4398299547	1.0628449875	-3.8026533224
H	-1.7282458752	-2.0727077913	-2.0476401429
H	-2.2278830015	-0.4639185881	-2.5870598441
H	-0.5639708758	-1.3892885891	-4.1723384782
H	0.5807306375	-1.6720715635	-2.8529749337
H	0.9946399555	-0.0765628690	1.1018029094

A7

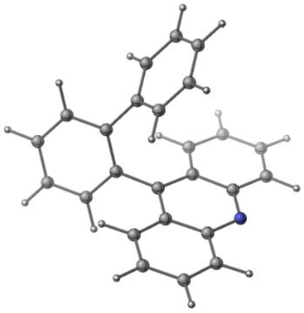

E(UwB97XD) = -1016.56476916

Charge = 0 Multiplicity = 3

C	-3.6922918645	-0.3161667280	0.1916419067
C	-2.3136950691	-0.0768634485	0.1759848457
C	-1.7732309006	0.8710657563	-0.7614230418
C	-2.6560521462	1.5422344583	-1.6048522095
C	-4.0462179641	1.2828819248	-1.5735430425
C	-4.5620297993	0.3588302778	-0.6866757880
H	-4.0759436822	-1.0403419057	0.9135703916
H	-2.2737364089	2.2871119470	-2.3051383885
H	-4.7050401933	1.8242491832	-2.2559193045
H	-5.6339061521	0.1531700552	-0.6554747141
C	-0.3463519452	1.1143918850	-0.7461113037
C	-0.1929321059	-0.5427461822	1.0909749006
C	0.4651787022	0.3633562140	0.1893889426
C	0.5693073605	-1.2587557714	2.0217804451
C	1.9688162217	-1.1177178931	2.0831227773
H	2.5409321138	-1.6848945821	2.8201514679
C	2.6033524442	-0.2655343328	1.2009335215
H	3.6888877395	-0.1486460072	1.2263111528
C	1.8520470620	0.4675866071	0.2530988544
H	2.3760909404	1.1287338457	-0.4397102783
N	-1.5429221634	-0.7552174819	1.0794060819
C	0.2706072972	2.1087107438	-1.6559701946
C	0.8585610105	3.2957529094	-1.1630645854
C	0.3069931335	1.8507168222	-3.0347272108
C	1.4833716148	4.1696312141	-2.0628038360
C	0.9229826903	2.7341740896	-3.9173027891
H	-0.1446269047	0.9302751525	-3.4130087530
C	1.5172601422	3.8969691402	-3.4282365921
H	1.9351969489	5.0890064877	-1.6819799189
H	0.9437891200	2.5103081641	-4.9862773500
H	2.0035494452	4.5972756089	-4.1111985838
H	0.0434879652	-1.9353524897	2.6989549766
C	0.8331232281	3.6237559290	0.2897933740
C	-0.3687441385	3.6139487063	1.0120484363
C	2.0219498478	3.9319965997	0.9647847644
C	-0.3779066342	3.8892297514	2.3785740402
C	2.0131082926	4.2064087592	2.3321598293
C	0.8135604453	4.1812910557	3.0441430193
H	-1.3075016953	3.3967768308	0.4975030007
H	2.9671993927	3.9348358775	0.4159077506
H	-1.3232960235	3.8779250647	2.9262090199
H	2.9506581494	4.4337569133	2.8452292236
H	0.8064904827	4.3915868481	4.1162721615

TS3-*anti*

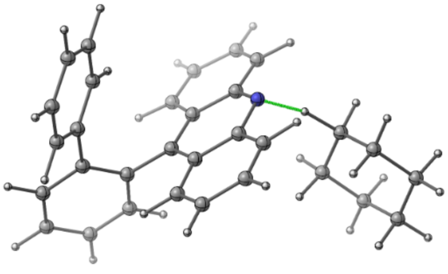

E(UwB97XD) = -1252.19800807

Charge = 0 Multiplicity = 3

C	-3.2605438867	0.0659387584	1.6326711441

Go to table of contents

C	-1.9385254301	0.0227980646	1.1835061765
C	-1.5207118421	0.8711654418	0.1155202197
C	-2.4804896400	1.7233881991	-0.4641143502
C	-3.8017470051	1.7427729090	-0.0178529783
C	-4.1942078330	0.9163986958	1.0327439695
H	-3.5488467570	-0.5761129887	2.4673392759
H	-2.1823912017	2.3808306913	-1.2828305244
H	-4.5223348949	2.4132601965	-0.4913415525
H	-5.2236495008	0.9341473374	1.3973455609
C	-0.1500604427	0.8094806758	-0.3270442342
C	0.2582572943	-0.9566533256	1.3513328537
C	0.7499074146	-0.1215894348	0.3043612206
C	1.1023721632	-1.8958268702	1.9488476155
C	2.4354759044	-2.0189867996	1.5460507893
H	3.0796794992	-2.7595102718	2.0253446401
C	2.9398085060	-1.1804176391	0.5540313480
H	3.9864292495	-1.2528187349	0.2501000270
C	2.1087252091	-0.2369264907	-0.0495049858
H	2.5160016796	0.4265216572	-0.8148908315
N	-1.0475669661	-0.8397065946	1.8089073050
C	0.3147966144	1.6652929281	-1.4491521845
C	0.4526099119	3.0638070557	-1.3073626648
C	0.5858063790	1.0770710979	-2.6932239084
C	0.8346499375	3.8267712989	-2.4196654322
C	0.9754703498	1.8463003641	-3.7867357086
H	0.4745316436	-0.0049666597	-2.8007803599
C	1.0967887376	3.2287556203	-3.6497851825
H	0.9432310949	4.9086999767	-2.3087748351
H	1.1779225822	1.3659144012	-4.7468053381
H	1.4003333048	3.8433133044	-4.5004629136
H	0.7019069159	-2.5281870399	2.7442521348
C	-1.9635348245	-3.2339768345	1.3608422953
C	-1.6298442720	-3.3124279816	-0.1123354045
C	-2.1344044370	-4.6380681870	-0.7039151506
C	-3.6229617783	-4.8437533550	-0.4206634518
C	-3.9300600694	-4.7462546004	1.0737972280
C	-3.4356190916	-3.4111047885	1.6568962978
H	-1.5944157455	-2.1054327470	1.7718375096
H	-1.3227477043	-3.8872774356	1.9775222152
H	-0.5453857732	-3.2099436352	-0.2732877923
H	-2.1196287288	-2.4736714525	-0.6395813044
H	-1.9393341567	-4.6597022409	-1.7879933956
H	-1.5604691663	-5.4705957392	-0.2602761130
H	-3.9515254896	-5.8199731240	-0.8122841147
H	-4.2042126074	-4.0745475975	-0.9600402060
H	-5.0118636502	-4.8502485652	1.2547840654
H	-3.4357518284	-5.5785621190	1.6049021800
H	-3.6285093876	-3.3663036491	2.7398841989
H	-4.0062337014	-2.5919160605	1.1862497703
C	0.1836877991	3.7409080970	-0.0074590290
C	-0.7156376330	4.8141211387	0.0560435294
C	0.8140881599	3.3206255023	1.1725514380
C	-0.9890244799	5.4435092322	1.2703213386
C	0.5413140926	3.9496567280	2.3862003060
C	-0.3642158817	5.0104476652	2.4399425647
H	-1.2206047493	5.1474550081	-0.8543365199
H	1.5334451756	2.4993589066	1.1383495500
H	-1.6996197464	6.2729632735	1.3026503953
H	1.0430760848	3.6103352709	3.2955800818
H	-0.5803924005	5.5005654654	3.3922632214

TS3-*syn*

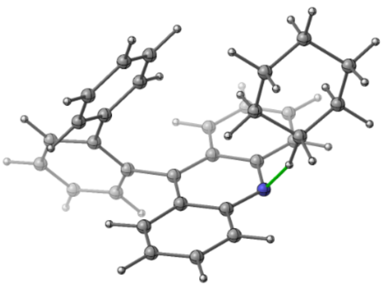

E(UwB97XD) = -1252.20072495

Charge = 0 Multiplicity = 3

C	-3.1946510208	0.0966752010	1.8059381736
C	-1.9049634150	0.0318531539	1.2734459229
C	-1.5303824178	0.9054676460	0.2080661871
C	-2.4962988188	1.8090190008	-0.2771111978
C	-3.7865443459	1.8483852211	0.2517089538
C	-4.1380801913	0.9931557149	1.2938182222
H	-3.4508606457	-0.5652294775	2.6354597187
H	-2.2255610394	2.4893422076	-1.0869577726
H	-4.5147573605	2.5561024138	-0.1506374464
H	-5.1424849385	1.0250053819	1.7217938986
C	-0.2054673707	0.8005497086	-0.3442214091
C	0.2656753316	-1.0175630359	1.2602850352
C	0.7076858851	-0.1722563927	0.1996772931
C	1.1171496173	-2.0016593802	1.7687970089
C	2.4065354071	-2.1676359808	1.2550436384
H	3.0561357000	-2.9449506559	1.6635988029
C	2.8621322681	-1.3273760423	0.2409275851
H	3.8751296559	-1.4366250571	-0.1526800290
C	2.0258448957	-0.3354303058	-0.2695893936
H	2.3962254742	0.3299008377	-1.0519744773
N	-0.9982372800	-0.8690564385	1.8145821759
C	0.2285239683	1.6847982156	-1.4577079745
C	0.4068984923	1.1949511478	-2.7688198834
C	0.5233336692	3.0288098144	-1.1867768358
C	0.9011287458	2.0559809948	-3.7580407677
C	0.9992706951	3.8782507696	-2.1828713113

H	0.3873127340	3.4050918760	-0.1696075634
C	1.1951983394	3.3874098001	-3.4732229838
H	1.0406959579	1.6733607285	-4.7723666547
H	1.2248637137	4.9210810856	-1.9481823062
H	1.5726330536	4.0426731197	-4.2616908514
H	0.7555991471	-2.6398799315	2.5779862240
C	-2.0069631959	-3.2418222192	1.4288627439
C	-1.6983139255	-3.3436155214	-0.0480487213
C	-2.2673063613	-4.6455974083	-0.6301226870
C	-3.7600031177	-4.7818510973	-0.3297192401
C	-4.0433887454	-4.6662147035	1.1682462443
C	-3.4822993610	-3.3533238974	1.7417810352
H	-1.5859199154	-2.1253642091	1.8156846994
H	-1.3875563572	-3.9164432155	2.0444177410
H	-0.6141829509	-3.2849143143	-0.2307387533
H	-2.1597791253	-2.4861647444	-0.5683404141
H	-2.0843315486	-4.6758836342	-1.7161779212
H	-1.7262258884	-5.5030106239	-0.1925962280
H	-4.1388676352	-5.7425211779	-0.7143167944
H	-4.3106946454	-3.9872490109	-0.8646557379
H	-5.1263735478	-4.7205700285	1.3633909079
H	-3.5805640885	-5.5188557871	1.6954611654
H	-3.6617827379	-3.2971304476	2.8265623140
H	-4.0212216816	-2.5109649074	1.2747131656
C	0.0820783689	-0.2166136543	-3.1209168810
C	1.0656395959	-1.0579718214	-3.6585082354
C	-1.2130914013	-0.7214997088	-2.9407488086
C	0.7645798348	-2.3769074273	-3.9977570693
C	-1.5170973343	-2.0359243109	-3.2922629200
C	-0.5282541790	-2.8695639095	-3.8162188077
H	2.0805908654	-0.6771634963	-3.7989223724
H	-1.9922180793	-0.0739539497	-2.5311340349
H	1.5452595682	-3.0232001862	-4.4061349113
H	-2.5334143832	-2.4121831560	-3.1530773190
H	-0.7660342277	-3.9014427700	-4.0855871461

NMR spectroscopic data

9-(3',5'-Bis(trifluoromethyl)-[1,1'-biphenyl]-2-yl)acridine (A2)

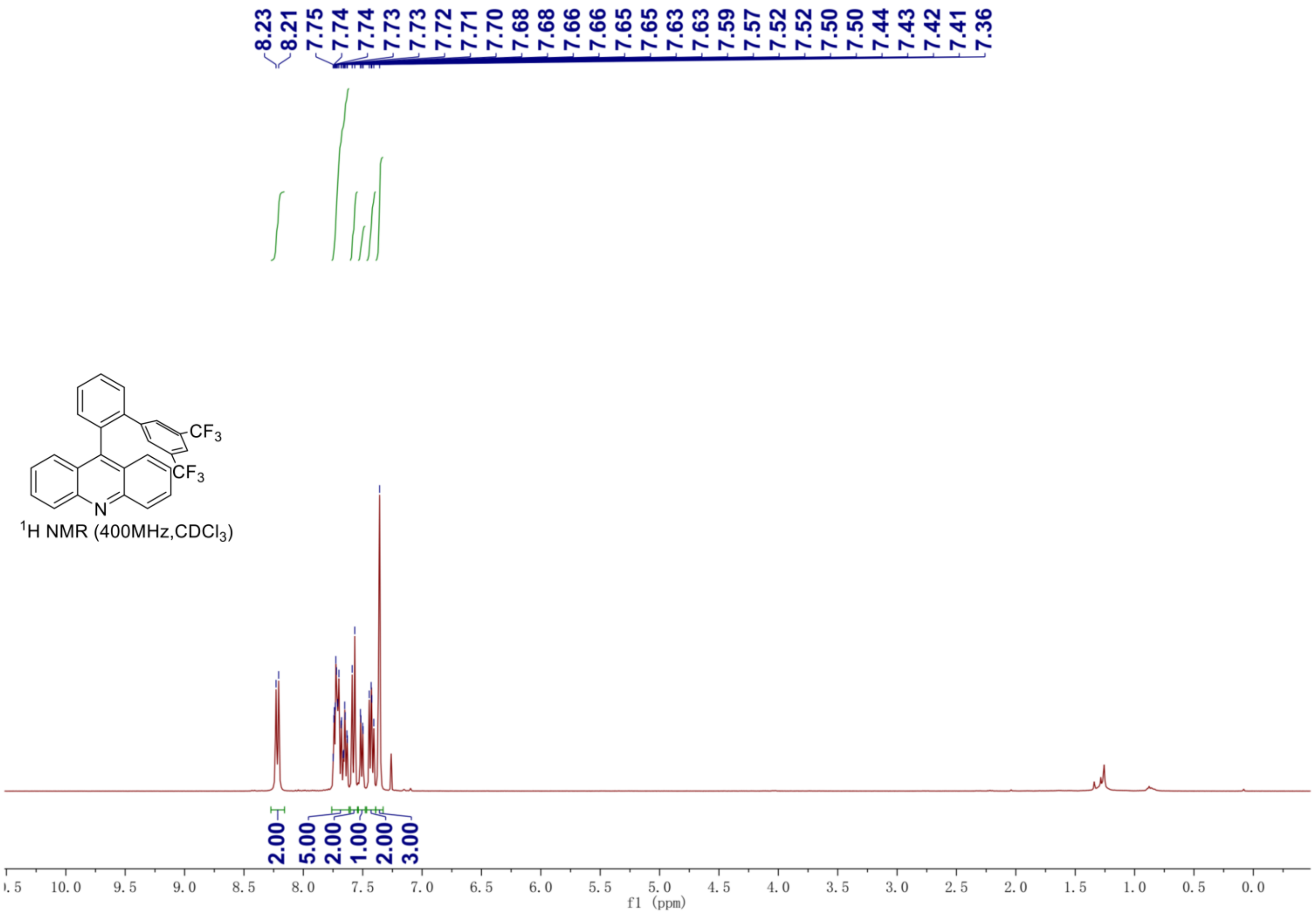

Go to table of contents

9-(3',5'-Bis(trifluoromethyl)-[1,1'-biphenyl]-2-yl)acridine(A2)

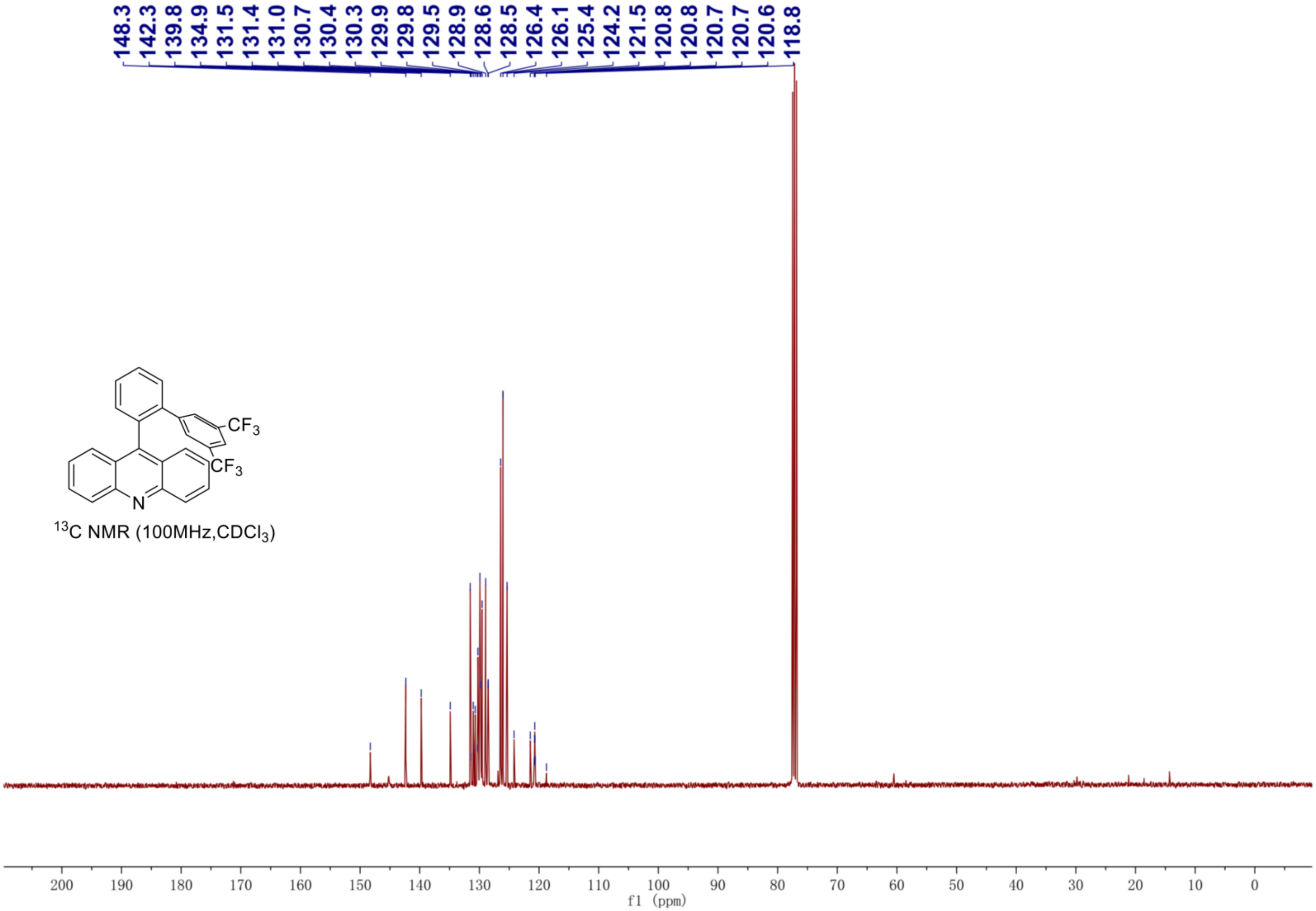

Go to table of contents

9-(3',5'-Bis(trifluoromethyl)-[1,1'-biphenyl]-2-yl)acridine(A2)

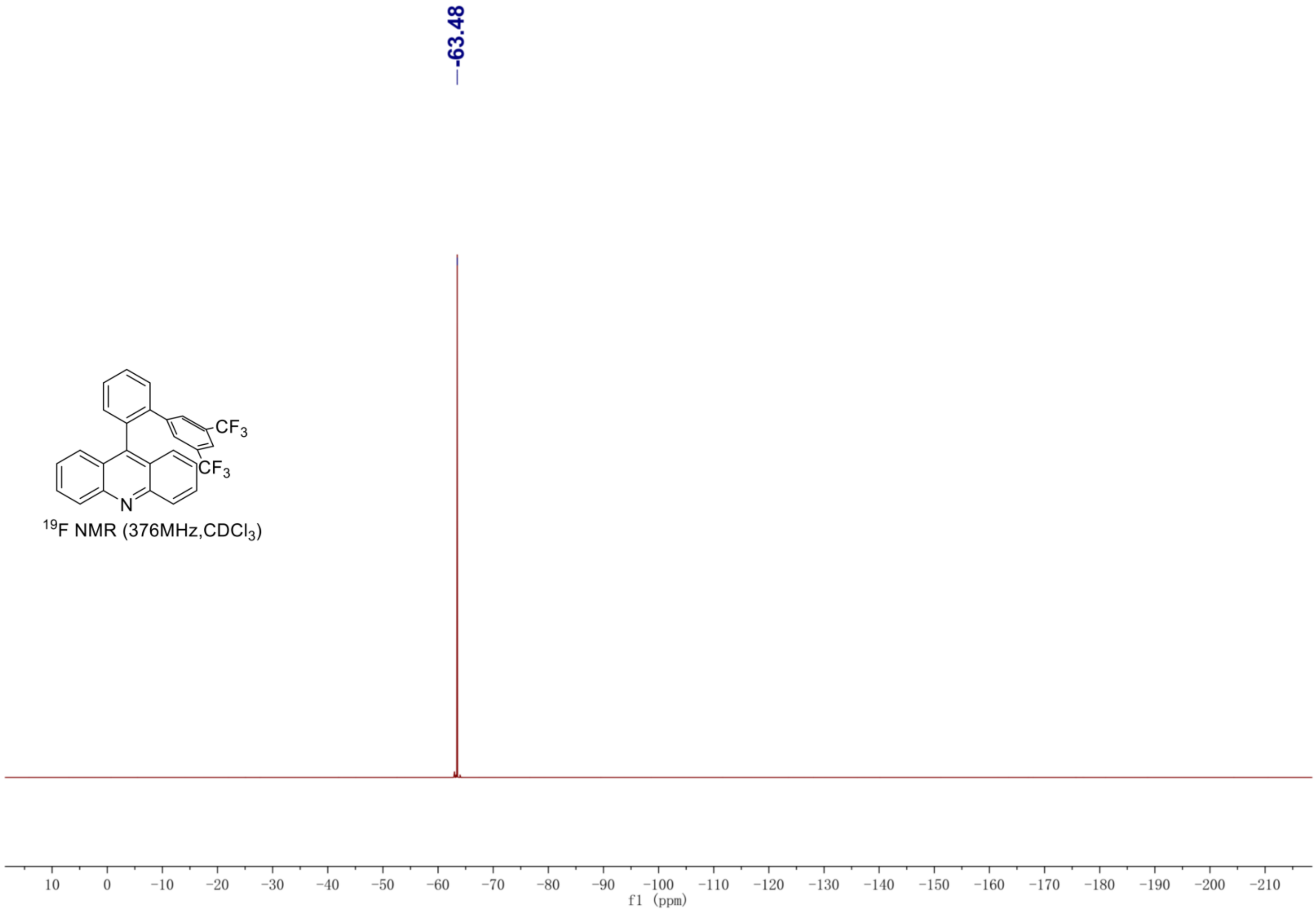

Go to table of contents

9-(2-Fluorophenyl)acridine (A3)

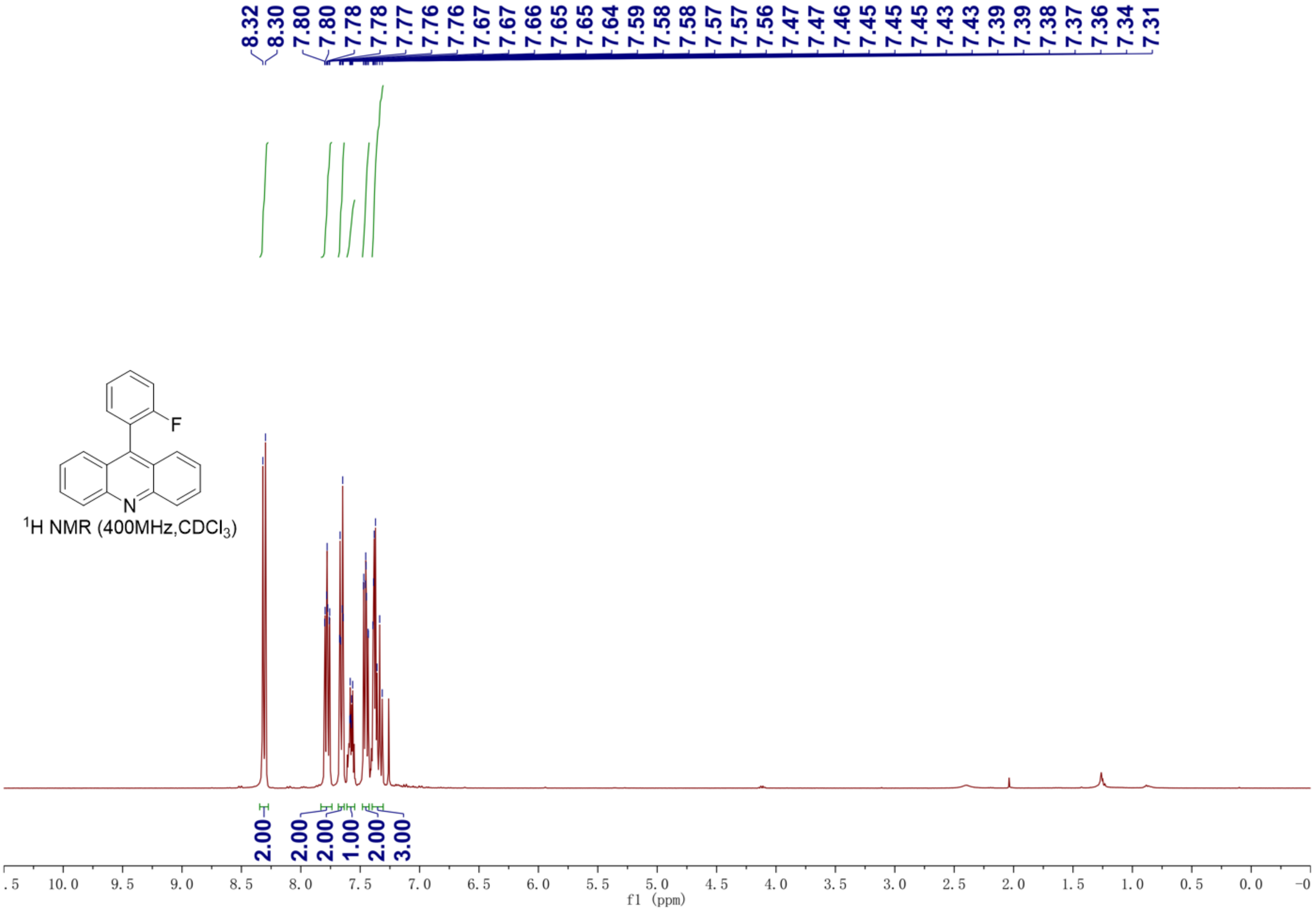

Go to table of contents

9-(2-Fluorophenyl)acridine (A3)

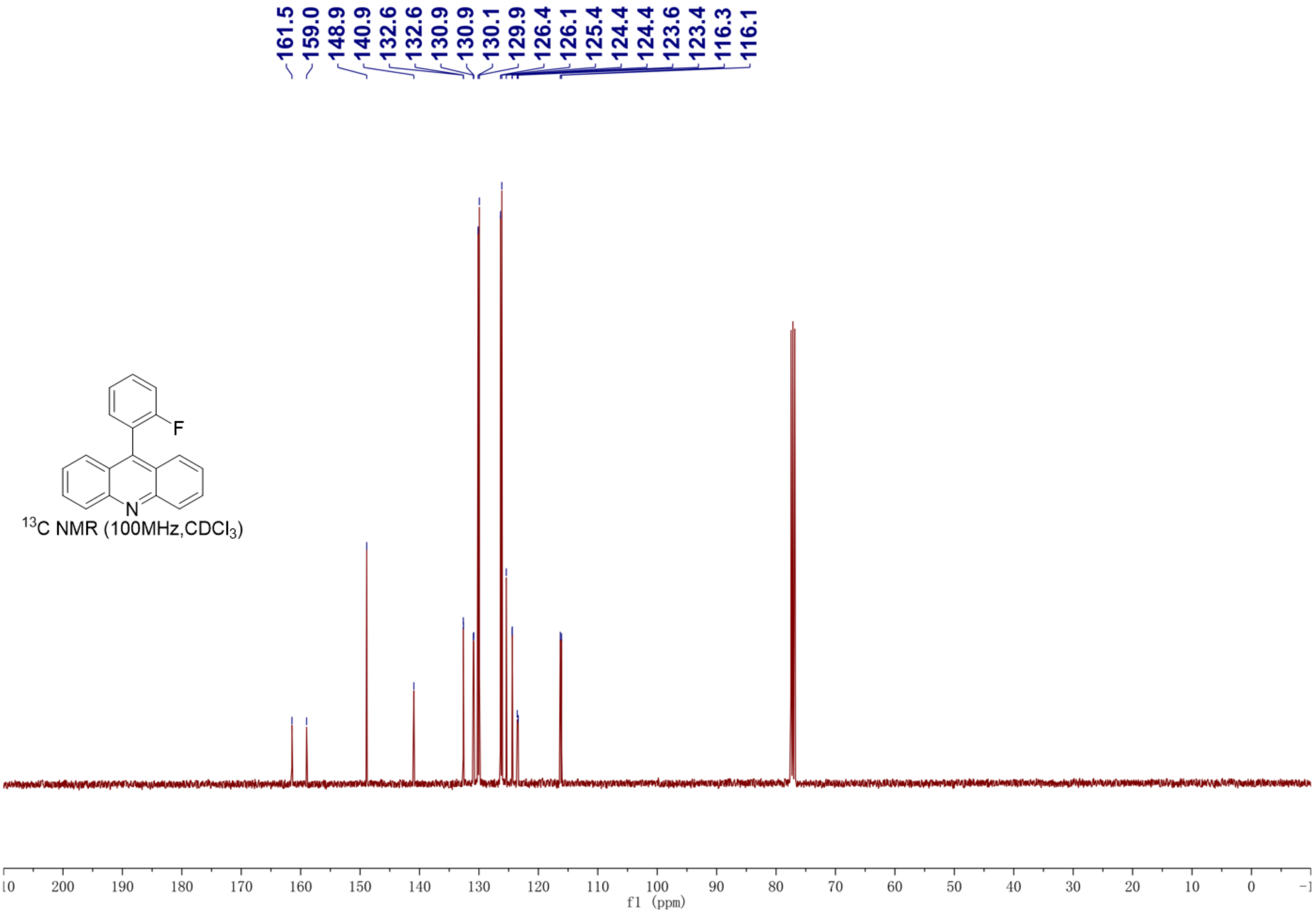

Go to table of contents

9-(2-Fluorophenyl)acridine (A3)

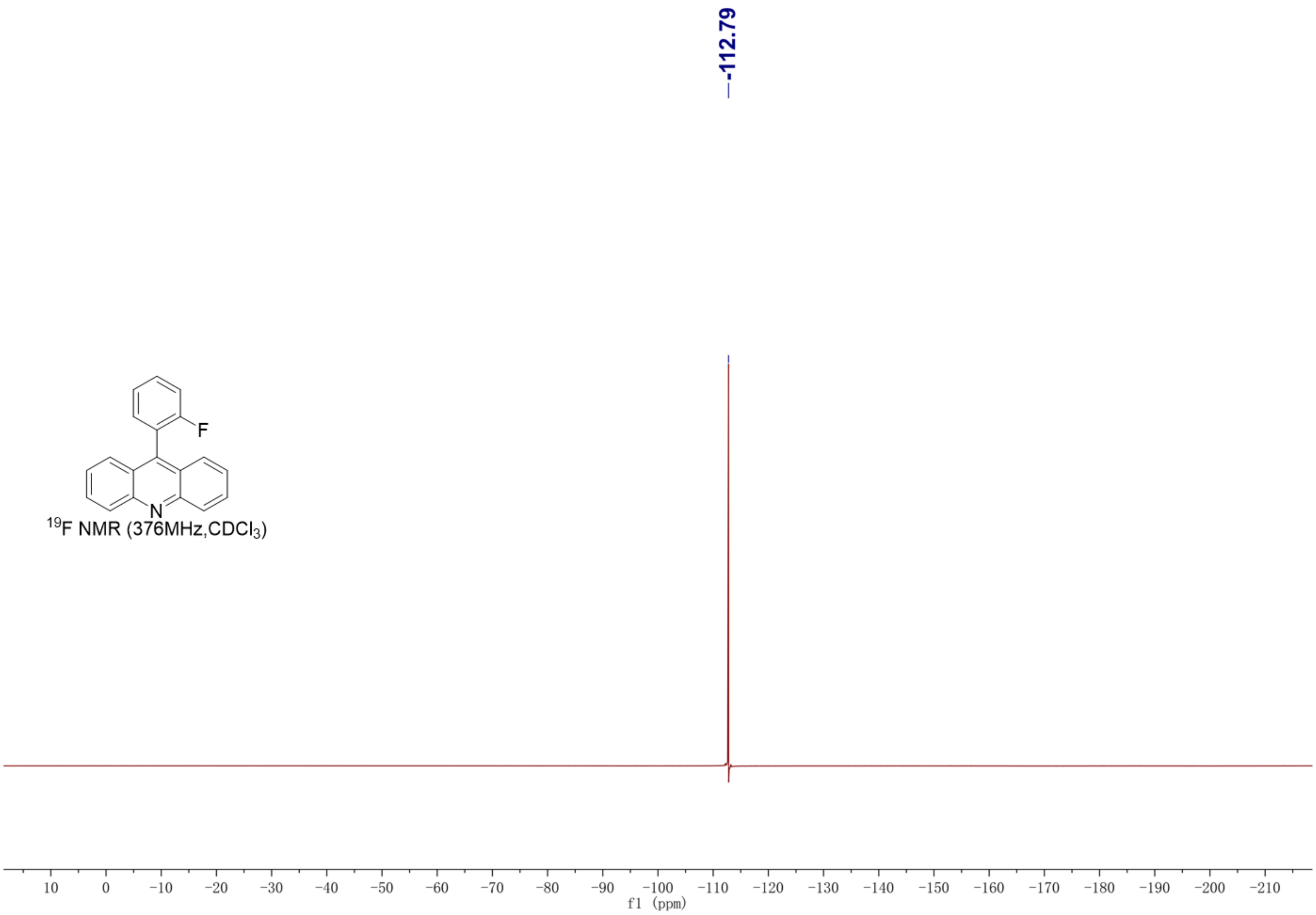

Go to table of contents

9-(2-(Trifluoromethyl)phenyl)acridine (A6)

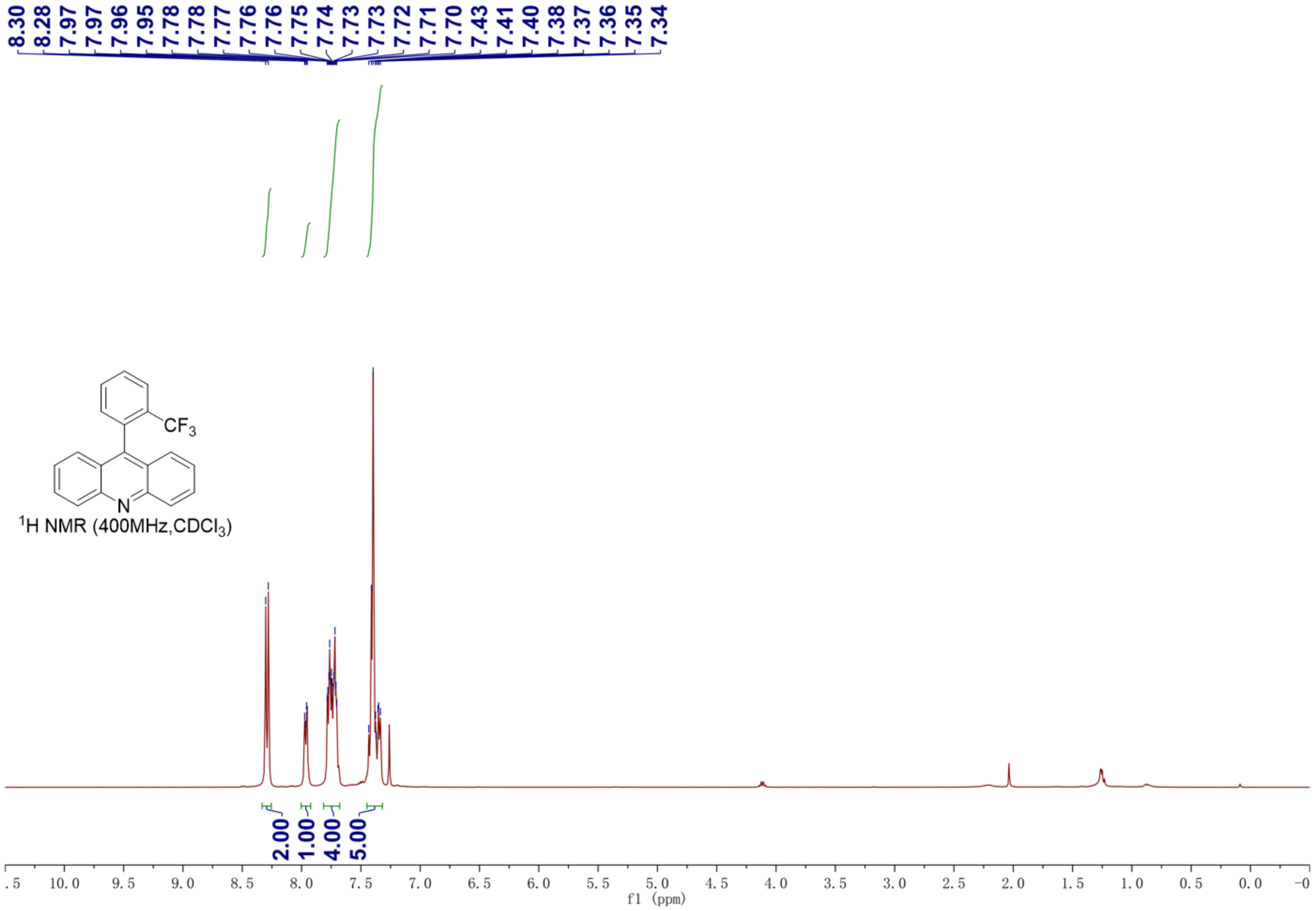

Go to table of contents

9-(2-(Trifluoromethyl)phenyl)acridine (A6)

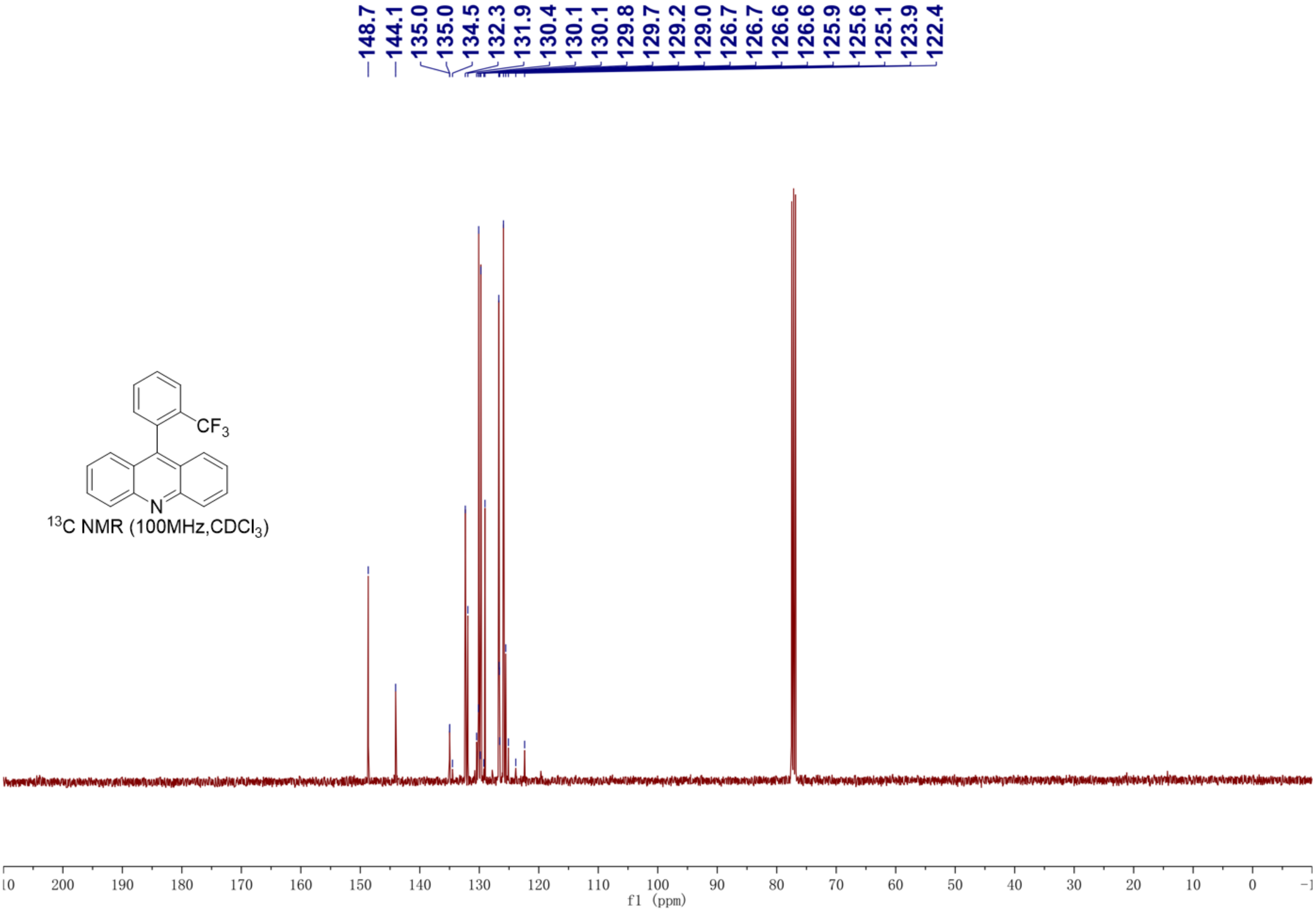

Go to table of contents

9-(2-(Trifluoromethyl)phenyl)acridine (A6)

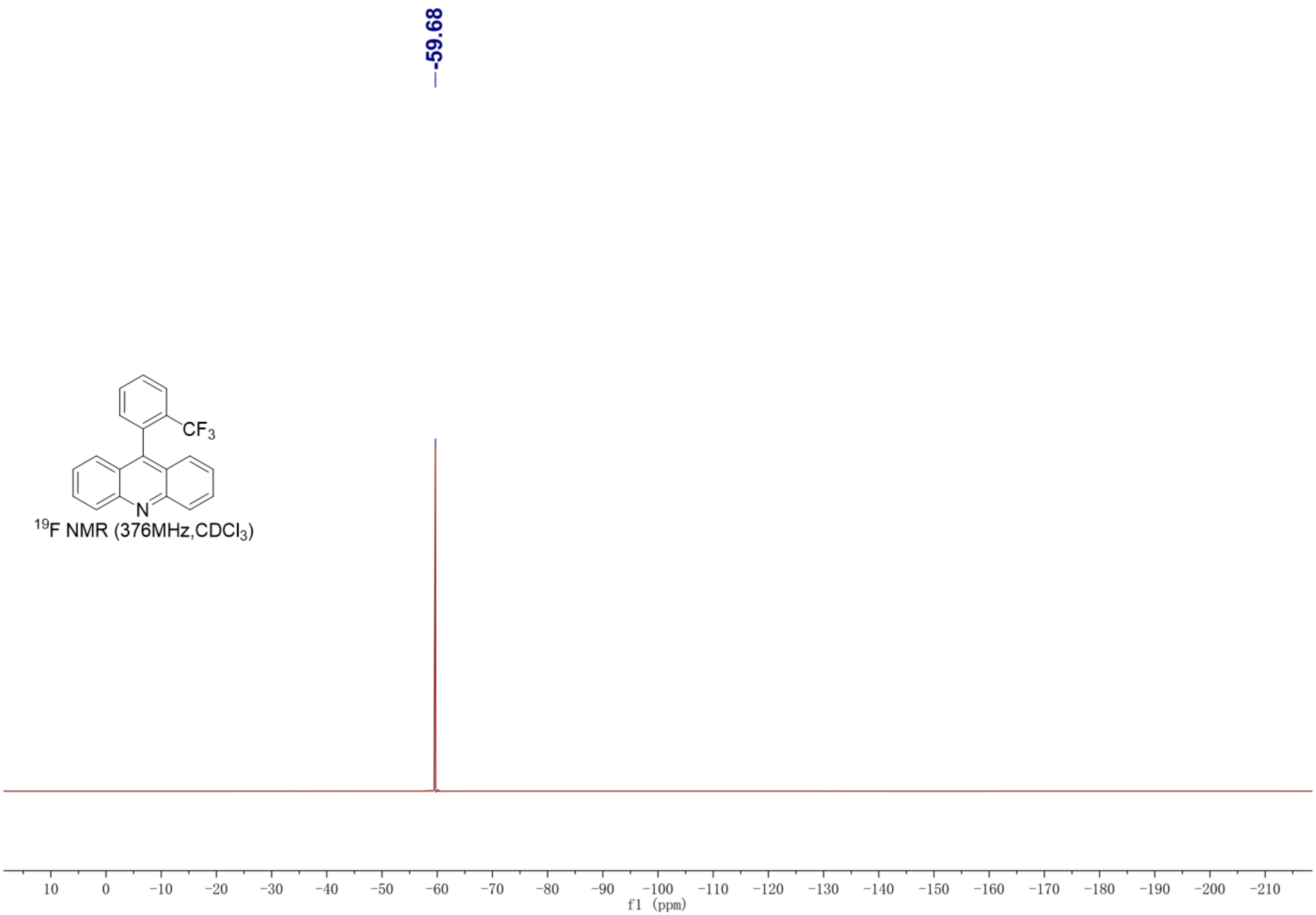

Go to table of contents

9-([1,1'-Biphenyl]-2-yl)acridine (A7)

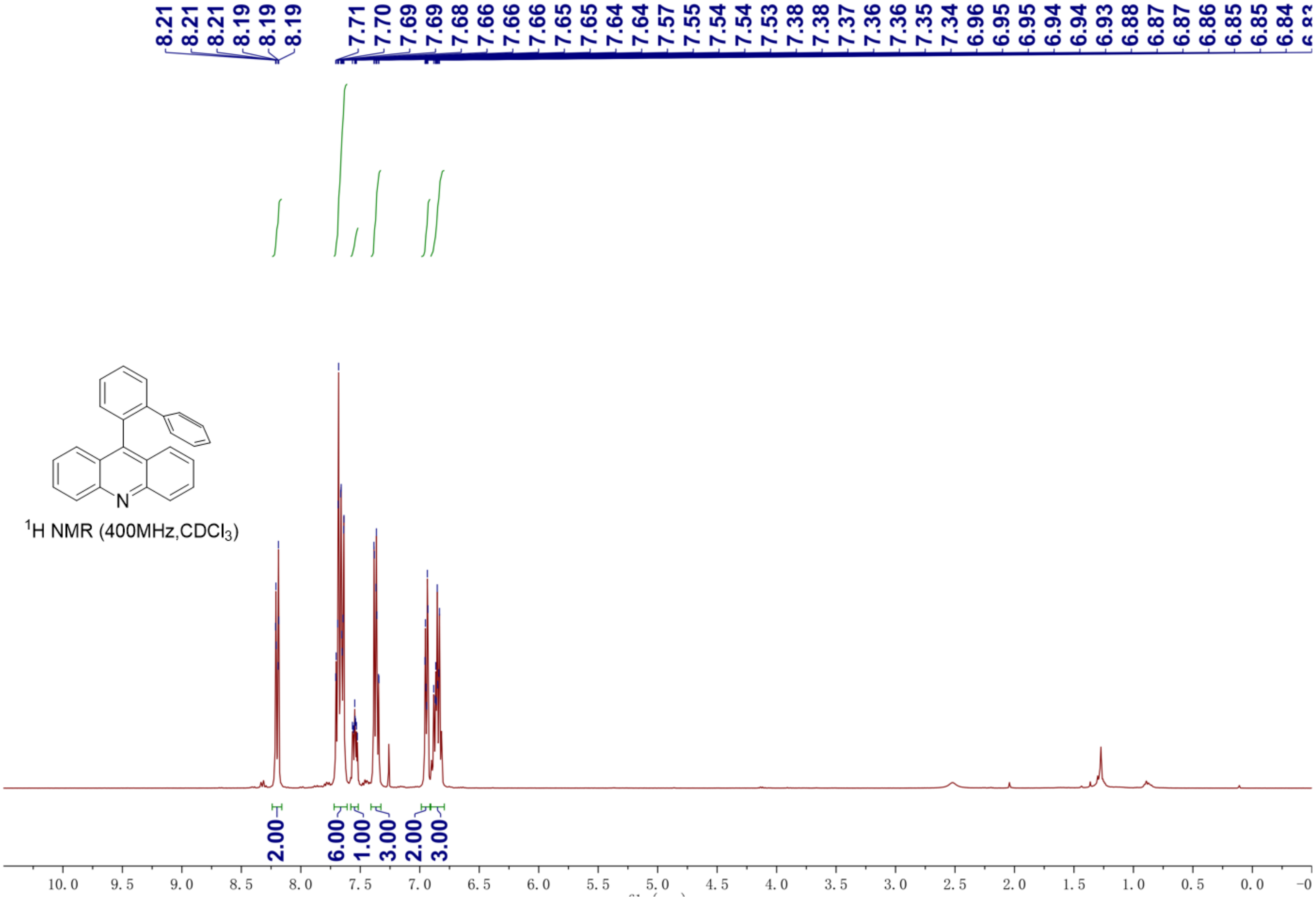

Go to table of contents

9-([1,1'-Biphenyl]-2-yl)acridine (A7)

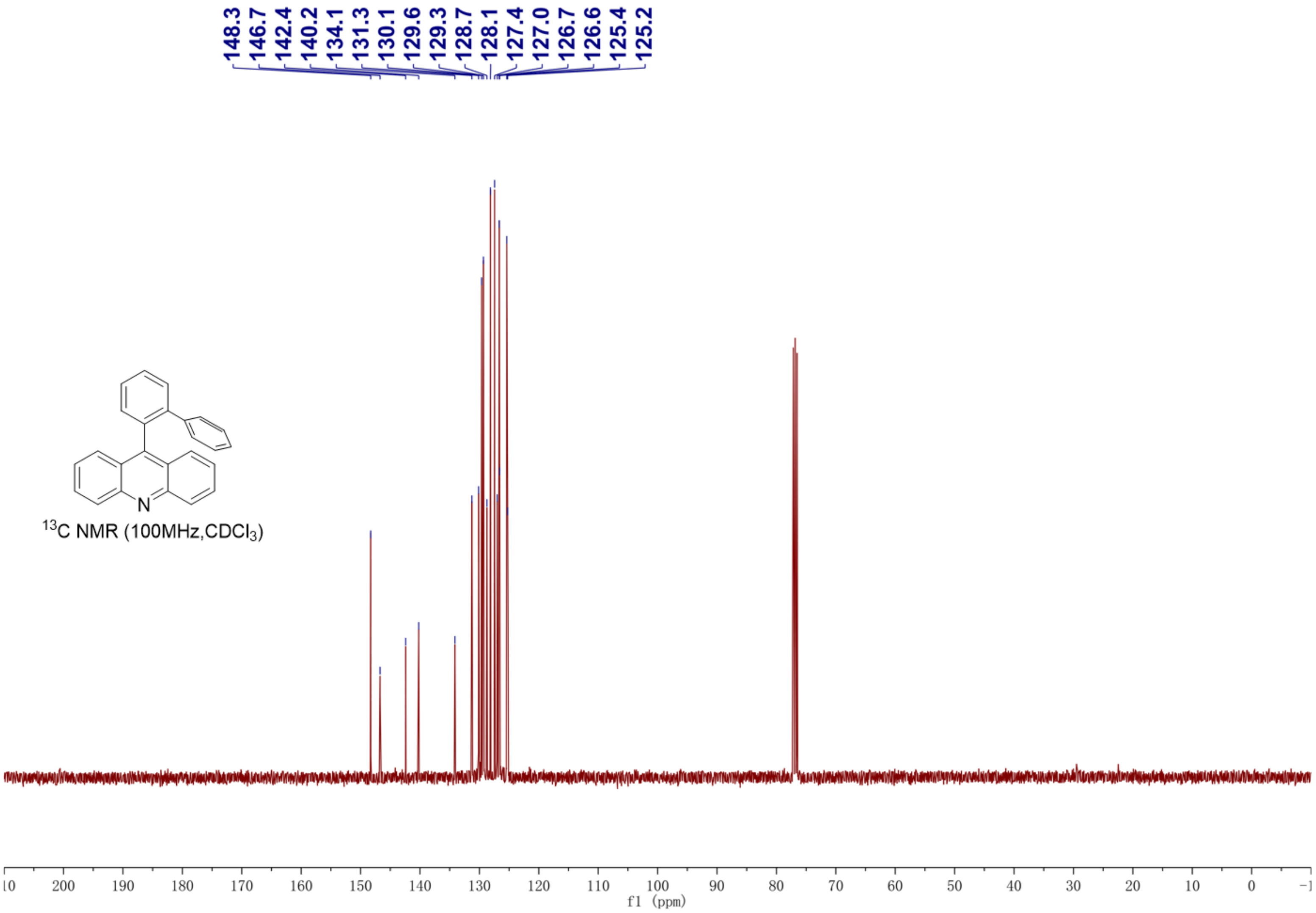

Go to table of contents

9-(4'-(Trifluoromethyl)-[1,1'-biphenyl]-2-yl)acridine (A8)

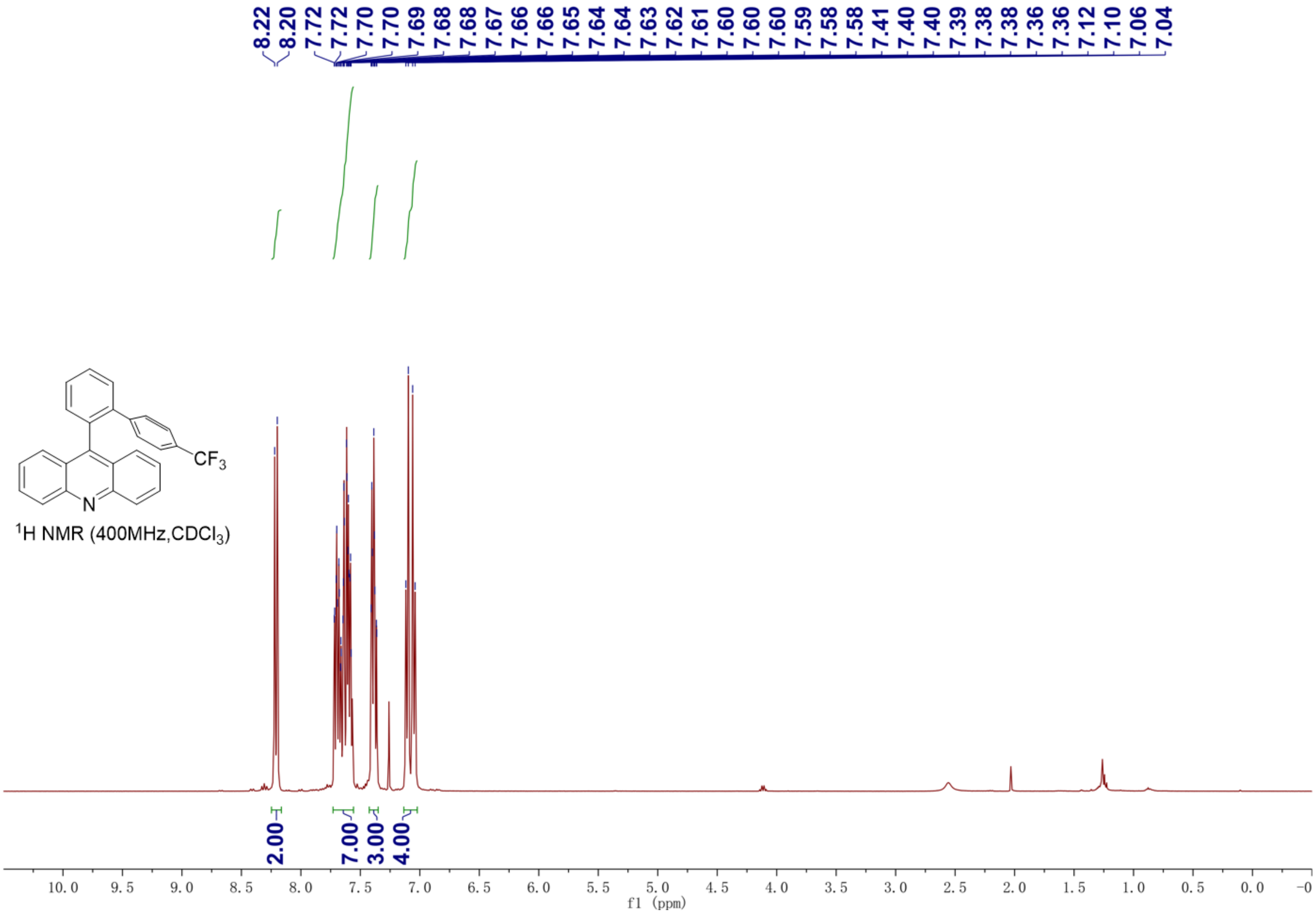

Go to table of contents

9-(4'-(Trifluoromethyl)-[1,1'-biphenyl]-2-yl)acridine (A8)

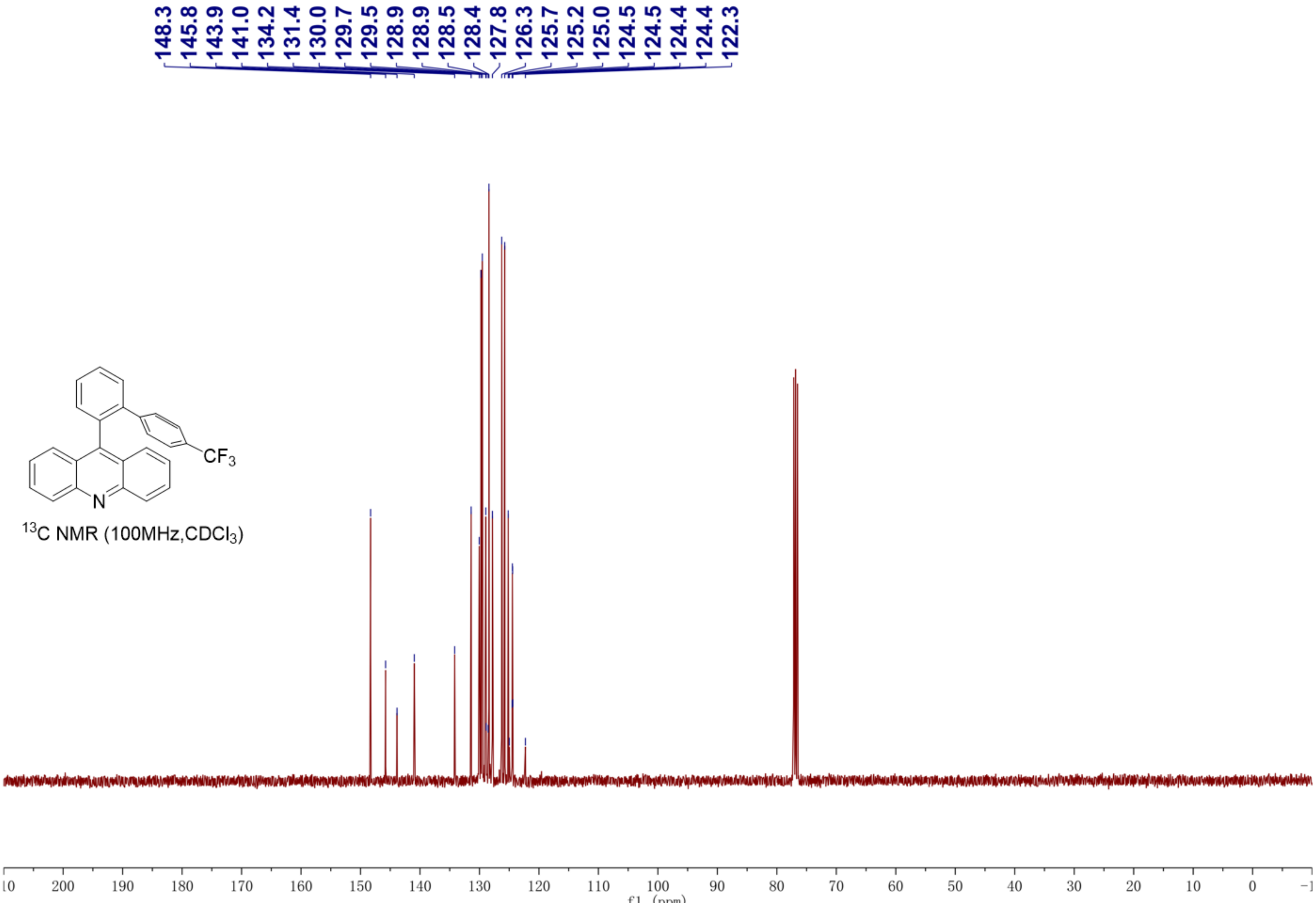

Go to table of contents

9-(4'-(Trifluoromethyl)-[1,1'-biphenyl]-2-yl)acridine (A8)

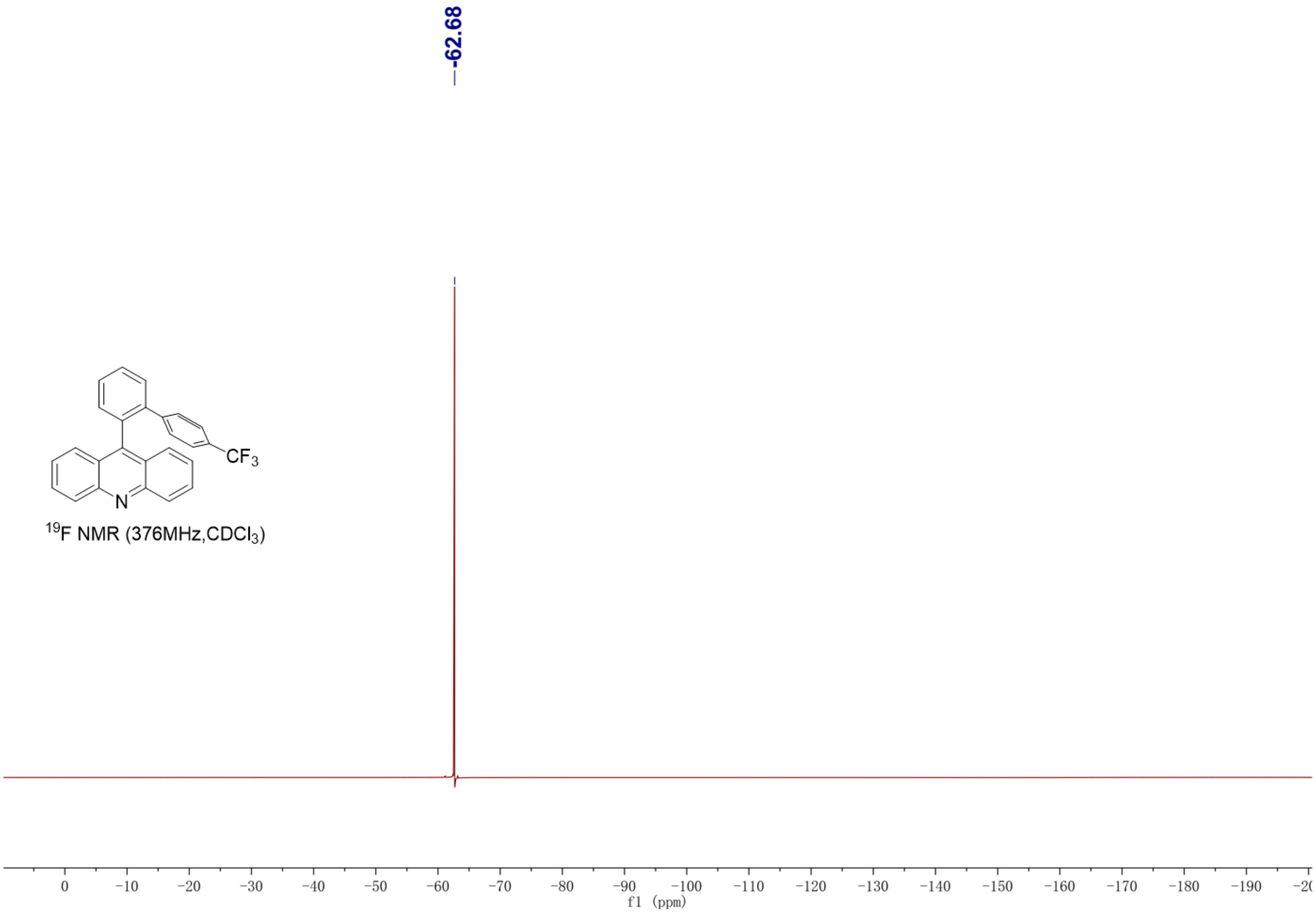

Go to table of contents

Methyl 2'-(acridin-9-yl)-3-fluoro-[1,1'-biphenyl]-4-carboxylate (A9)

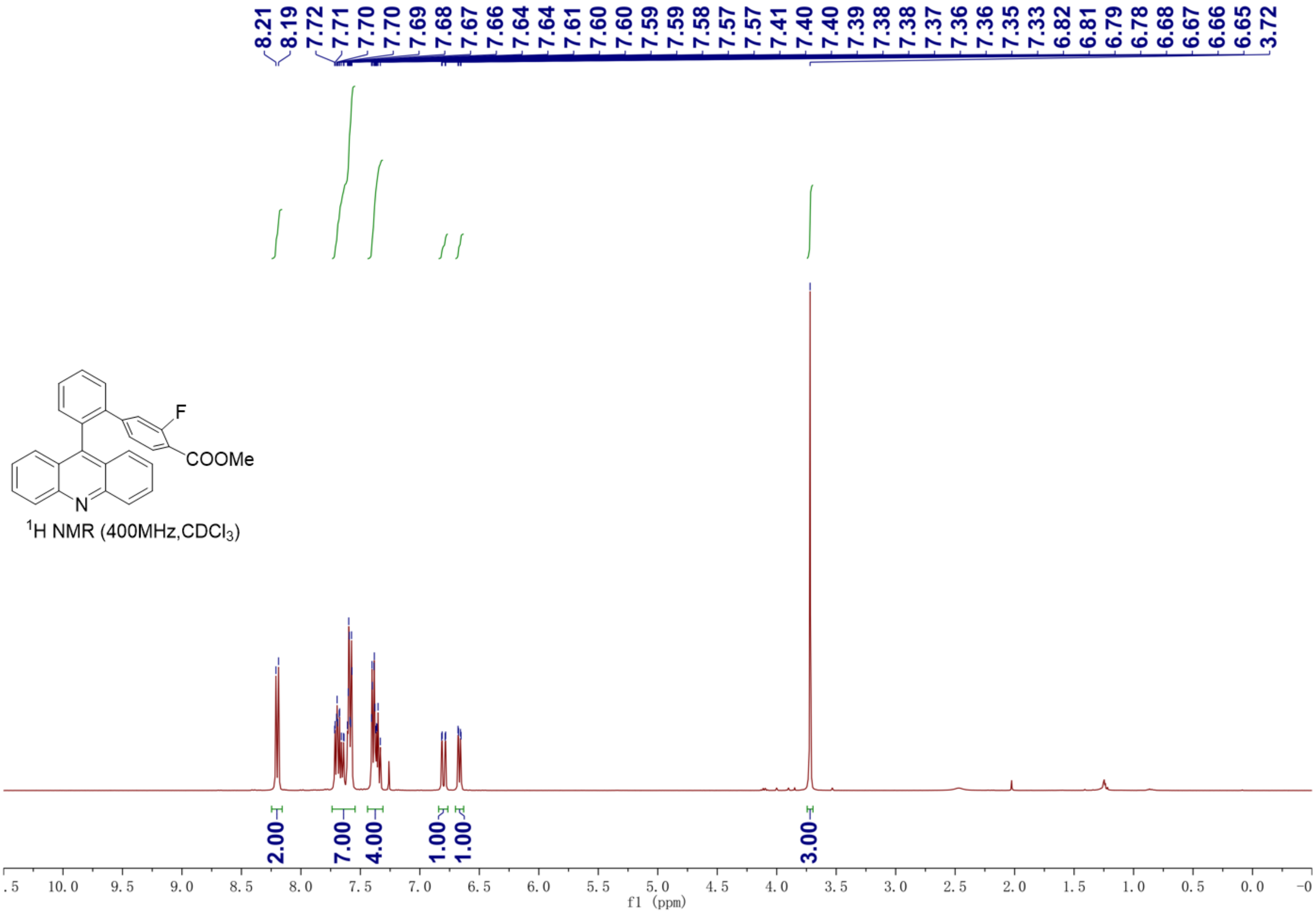

Go to table of contents

Methyl 2'-(acridin-9-yl)-3-fluoro-[1,1'-biphenyl]-4-carboxylate (A9)

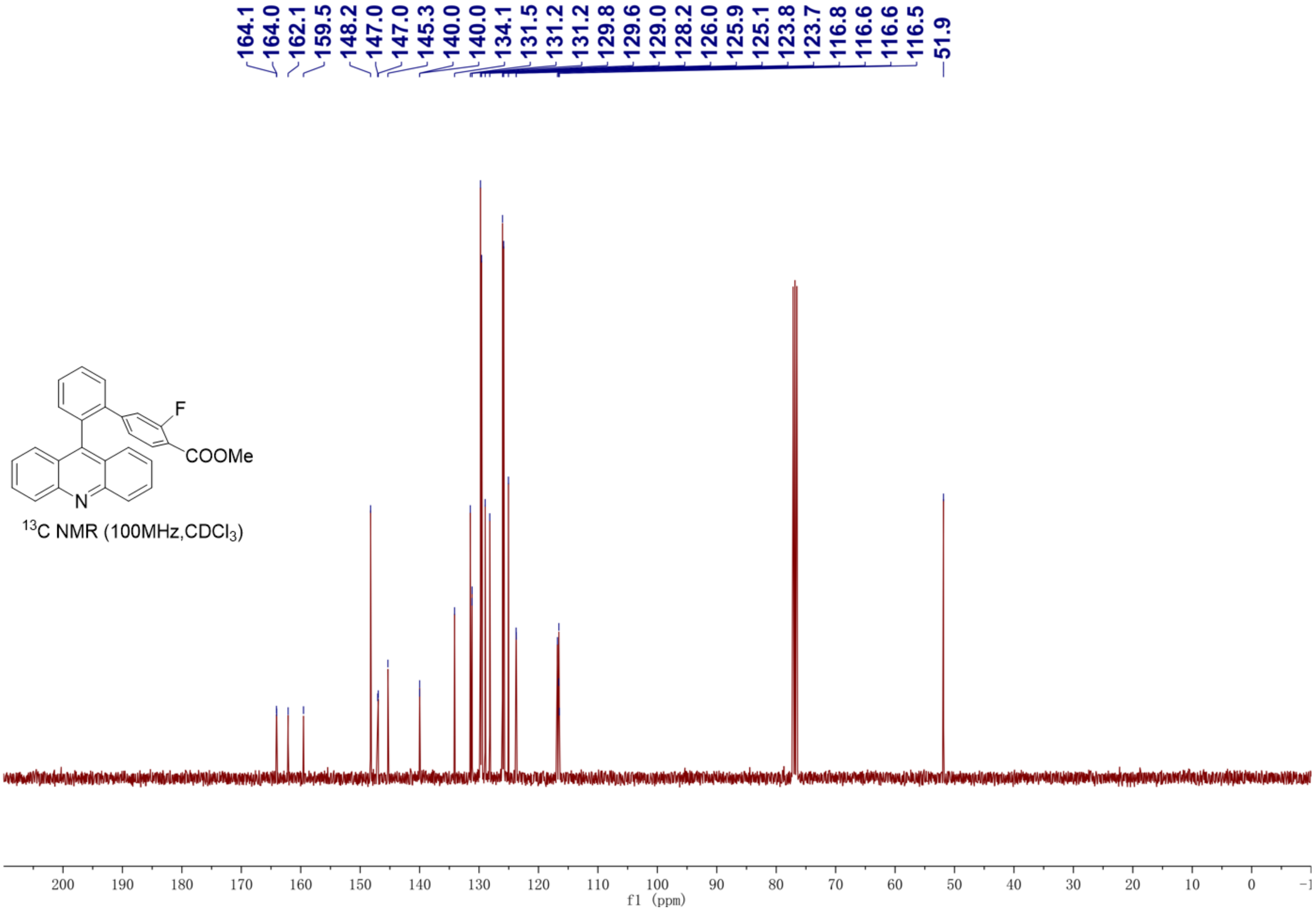

Go to table of contents

Methyl 2'-(acridin-9-yl)-3-fluoro-[1,1'-biphenyl]-4-carboxylate (A9)

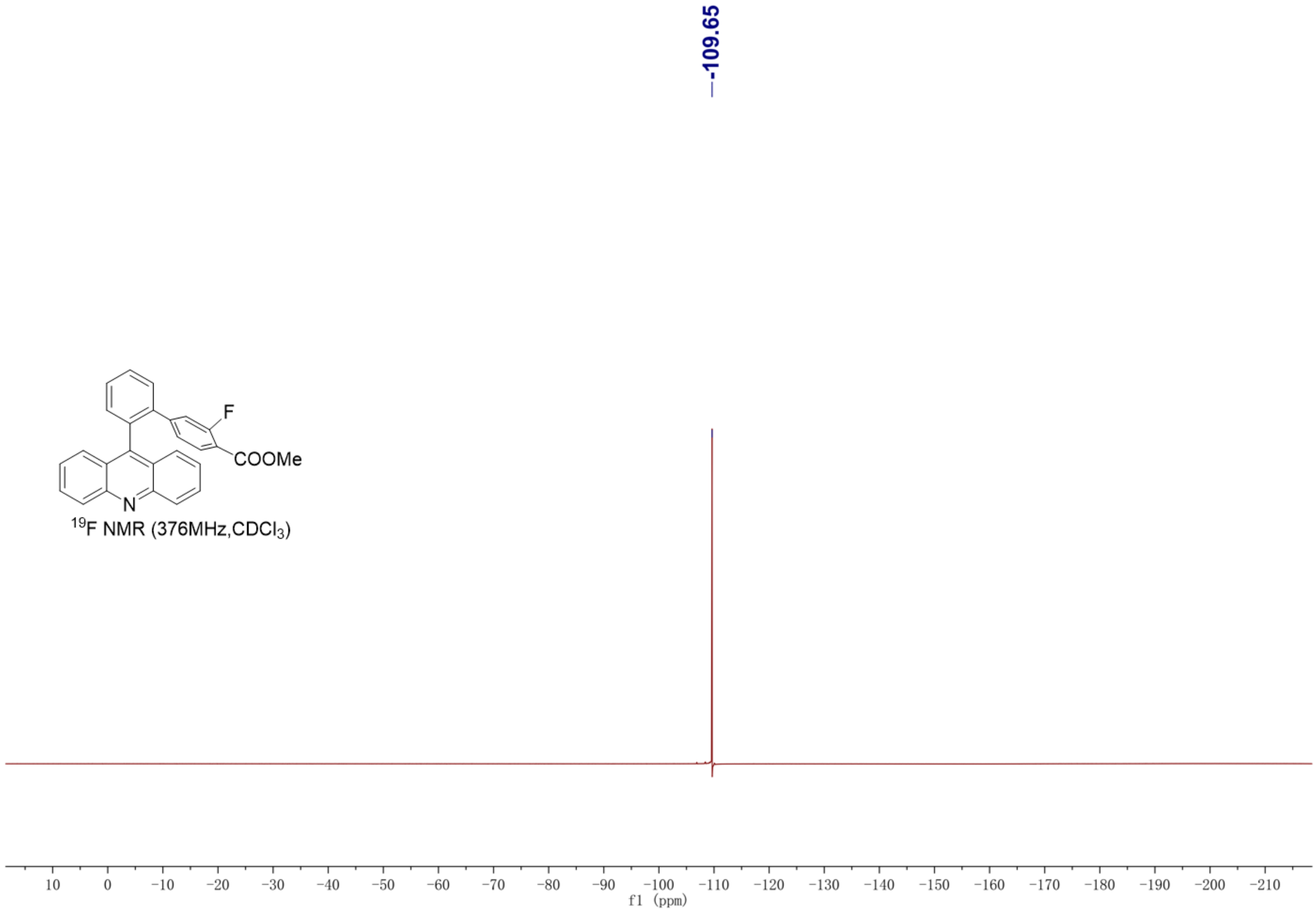

Go to table of contents

2'-(Acridin-9-yl)-[1,1'-biphenyl]-3-carbonitrile (A10)

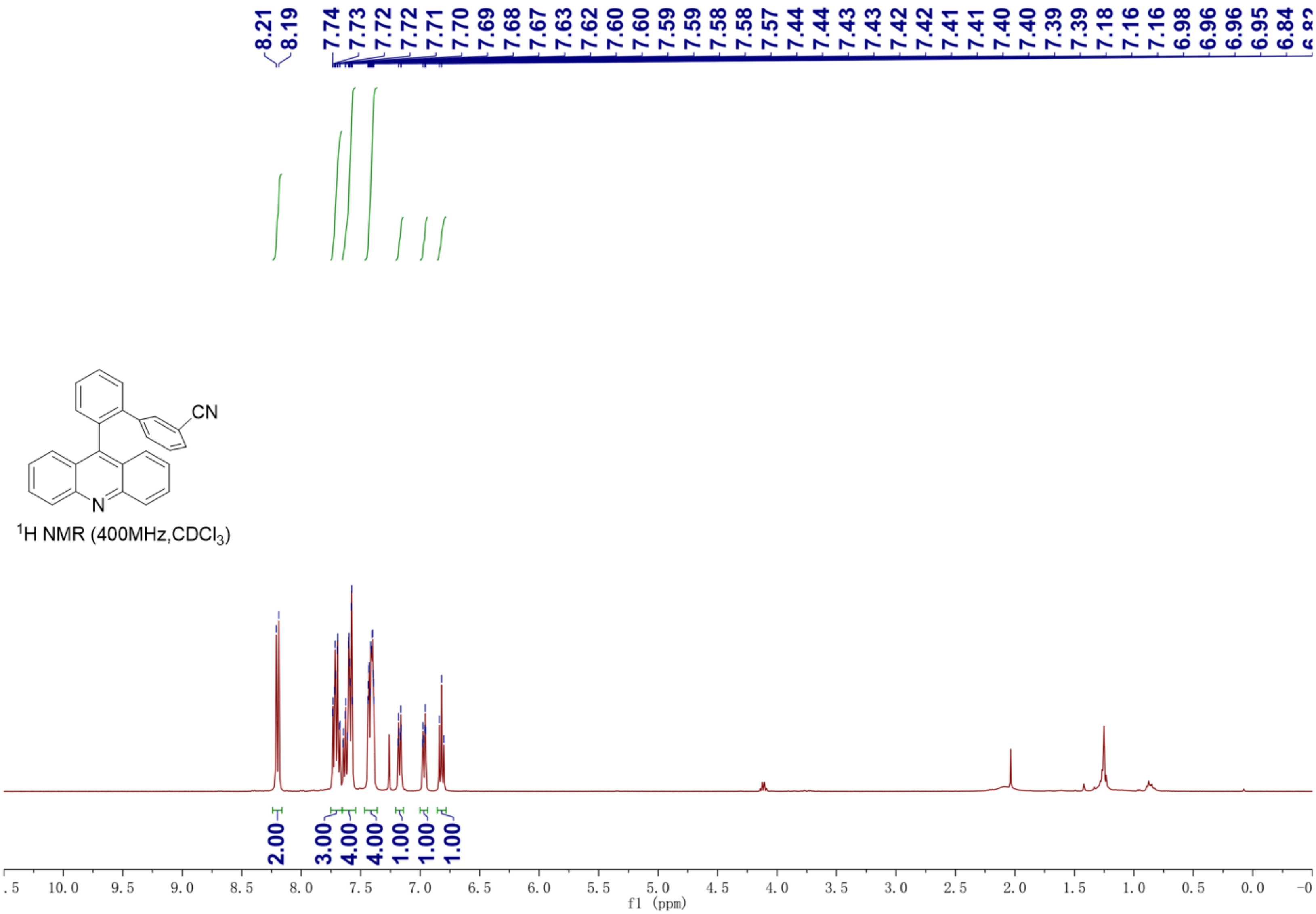

Go to table of contents

2'-(Acridin-9-yl)-[1,1'-biphenyl]-3-carbonitrile (A10)

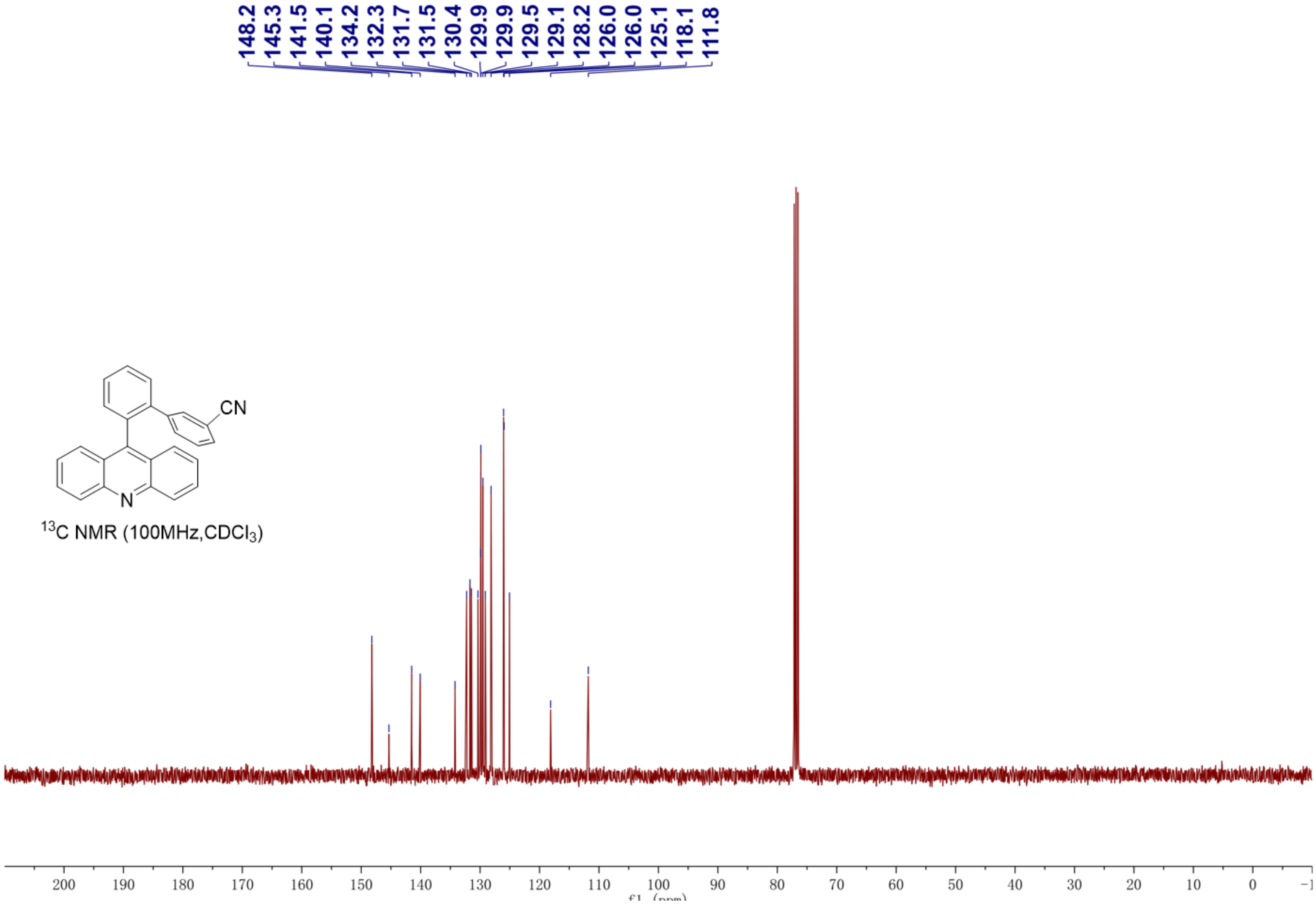

Go to table of contents

9-(3',5'-Di-tert-butyl-[1,1'-biphenyl]-2-yl)acridine (A11)

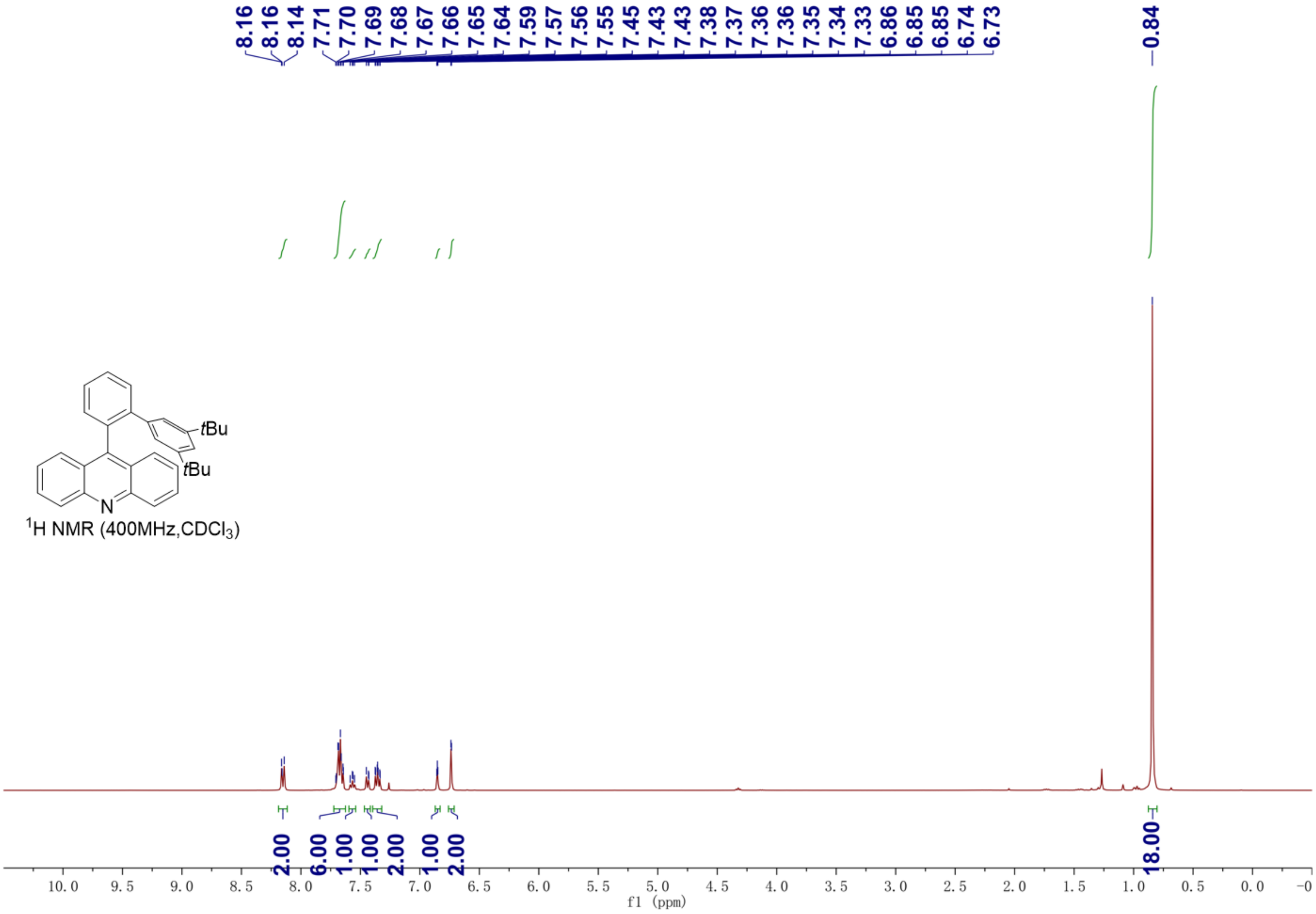

Go to table of contents

9-(3',5'-Di-*tert*-butyl-[1,1'-biphenyl]-2-yl)acridine (A11)

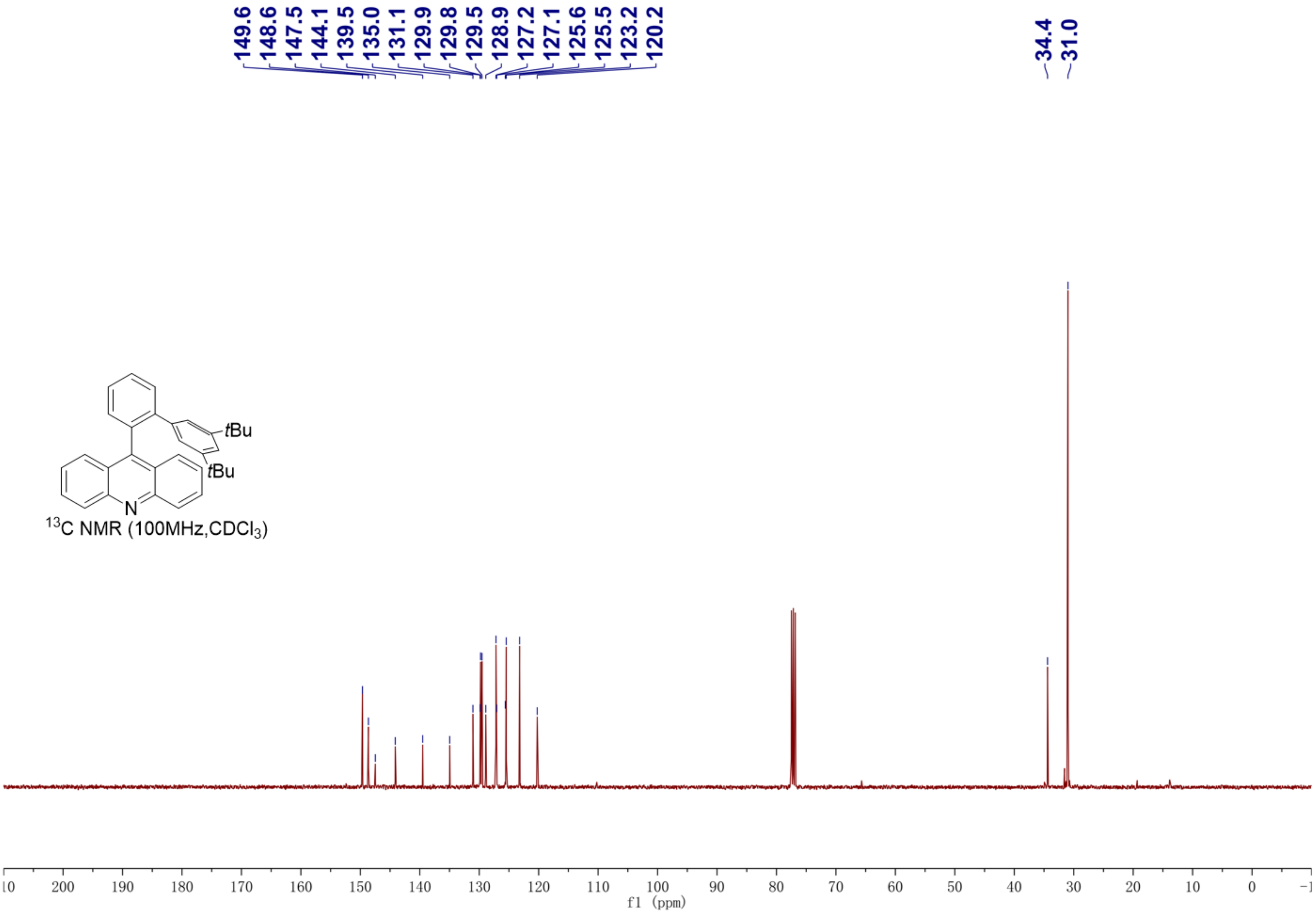

9-(5'-Phenyl-[1,1':3',1''-terphenyl]-2-yl)acridine (A12)

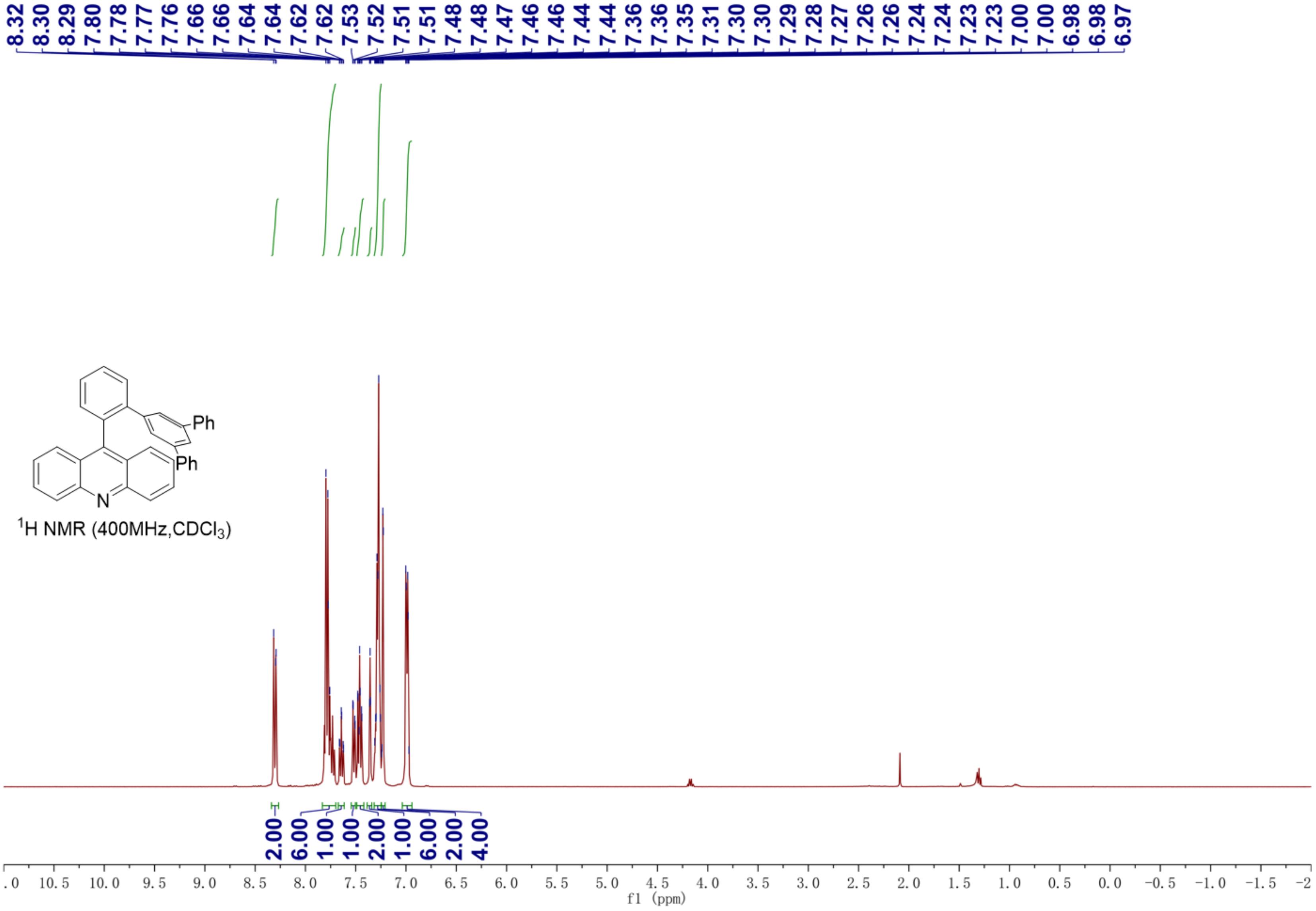

Go to table of contents

9-(5'-Phenyl-[1,1':3',1''-terphenyl]-2-yl)acridine (A12)

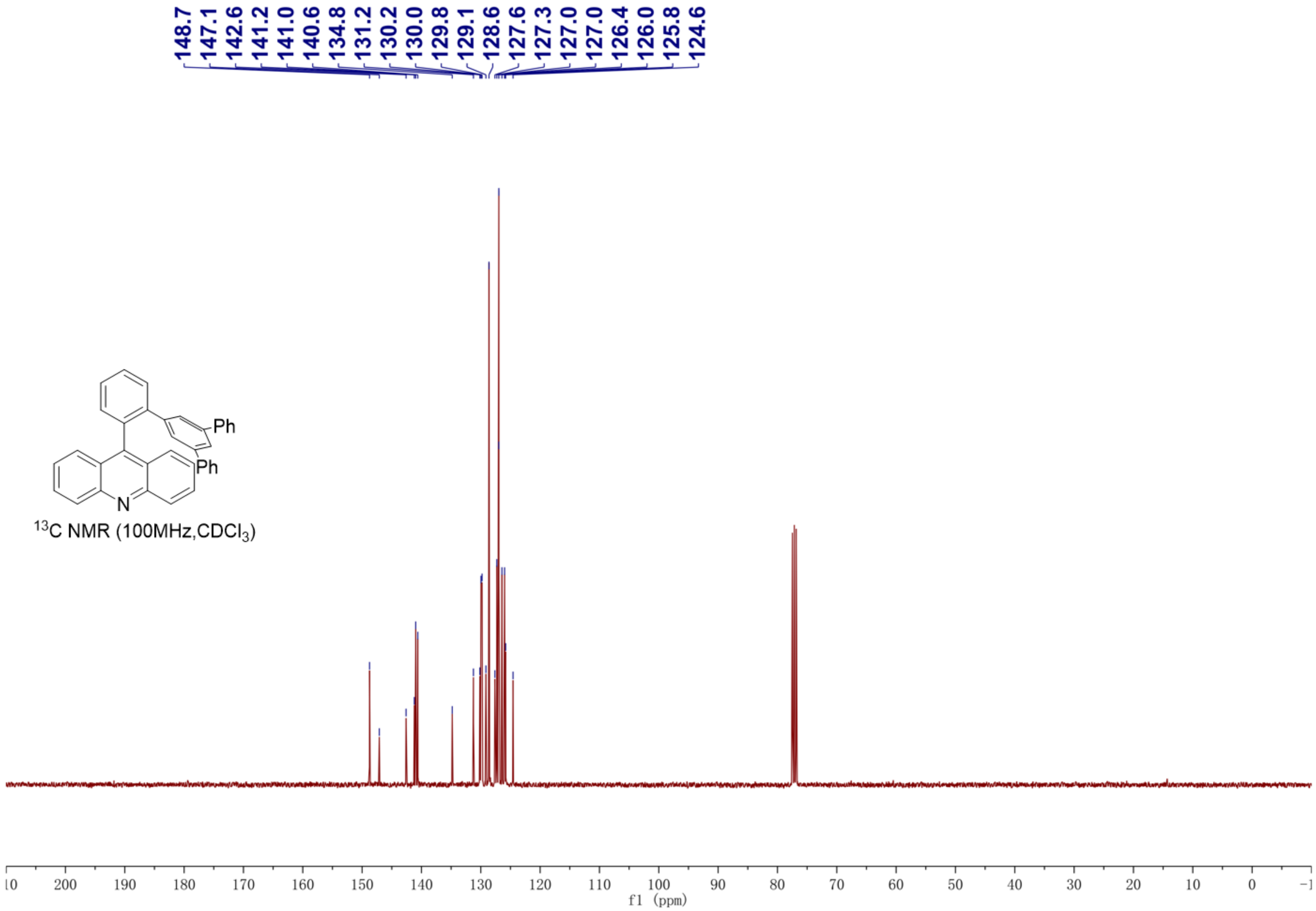

Go to table of contents

4-(2,2-Dicyanovinyl)phenyl 5-(2,5-dimethylphenoxy)-2,2-dimethylpentanoate

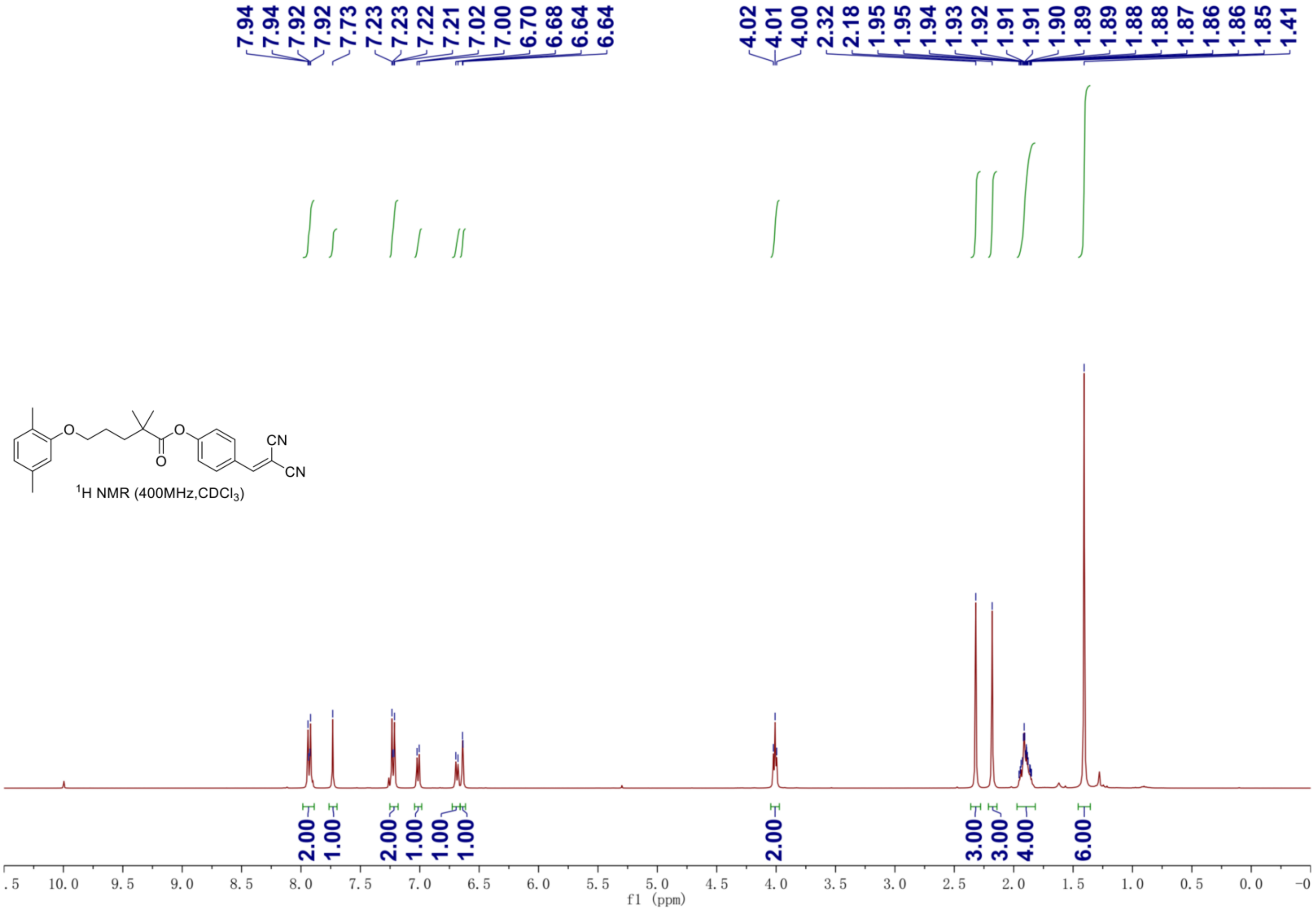

Go to table of contents

4-(2,2-Dicyanovinyl)phenyl 5-(2,5-dimethylphenoxy)-2,2-dimethylpentanoate

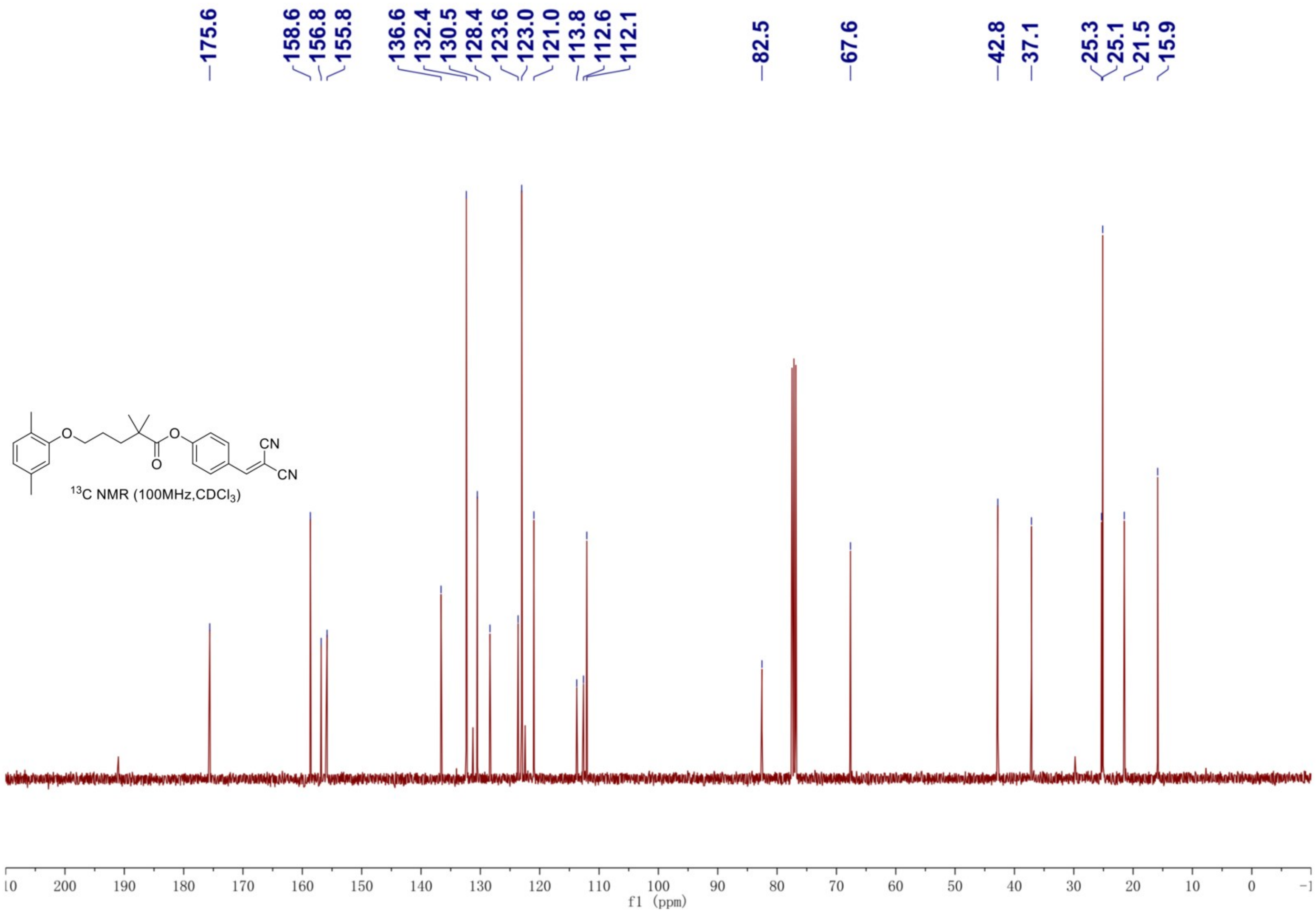

Go to table of contents

Ethyl 2-(4-(2,2-dicyanovinyl)phenoxy)-2-methylpropanoate

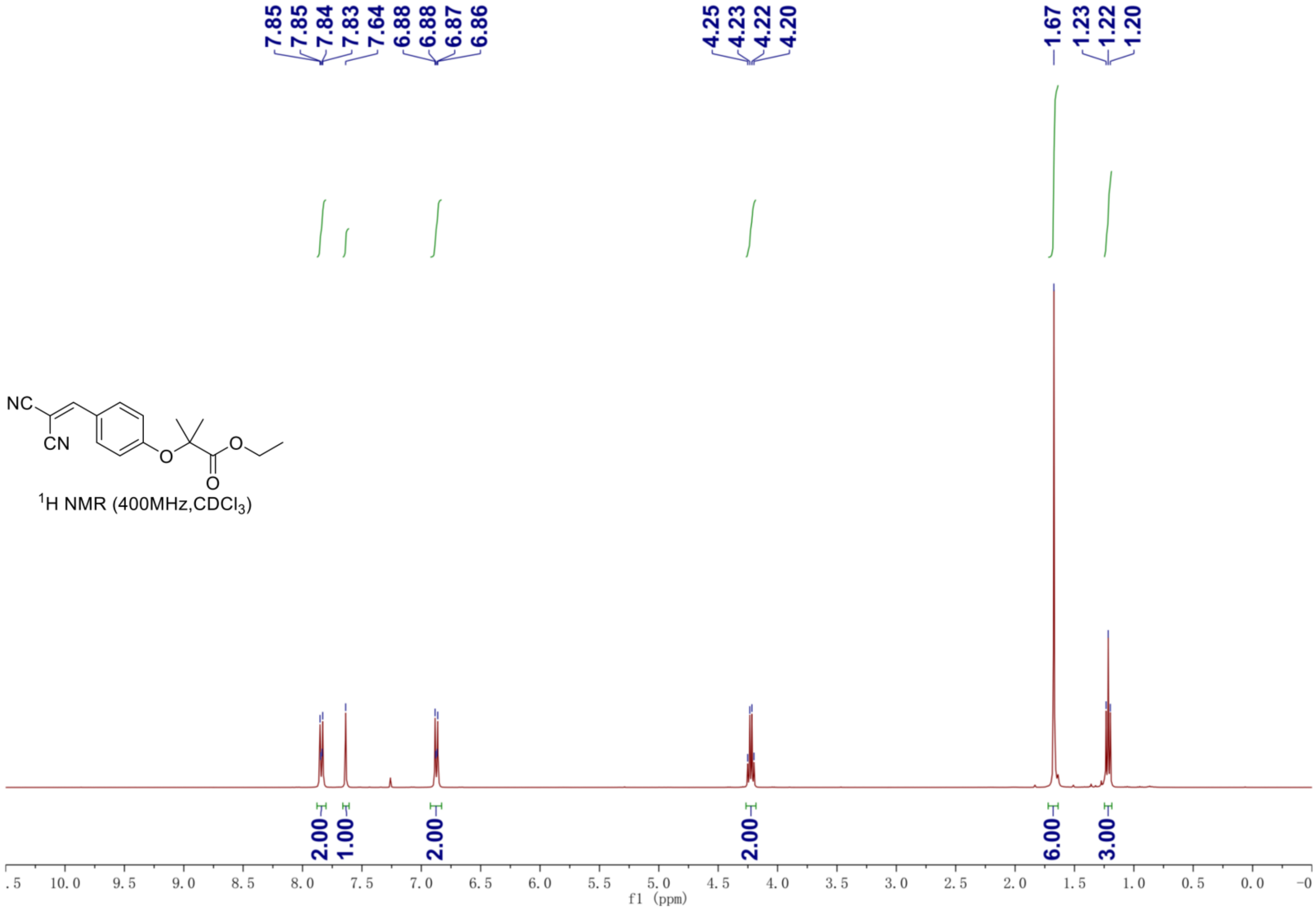

Go to table of contents

Ethyl 2-(4-(2,2-dicyanovinyl)phenoxy)-2-methylpropanoate

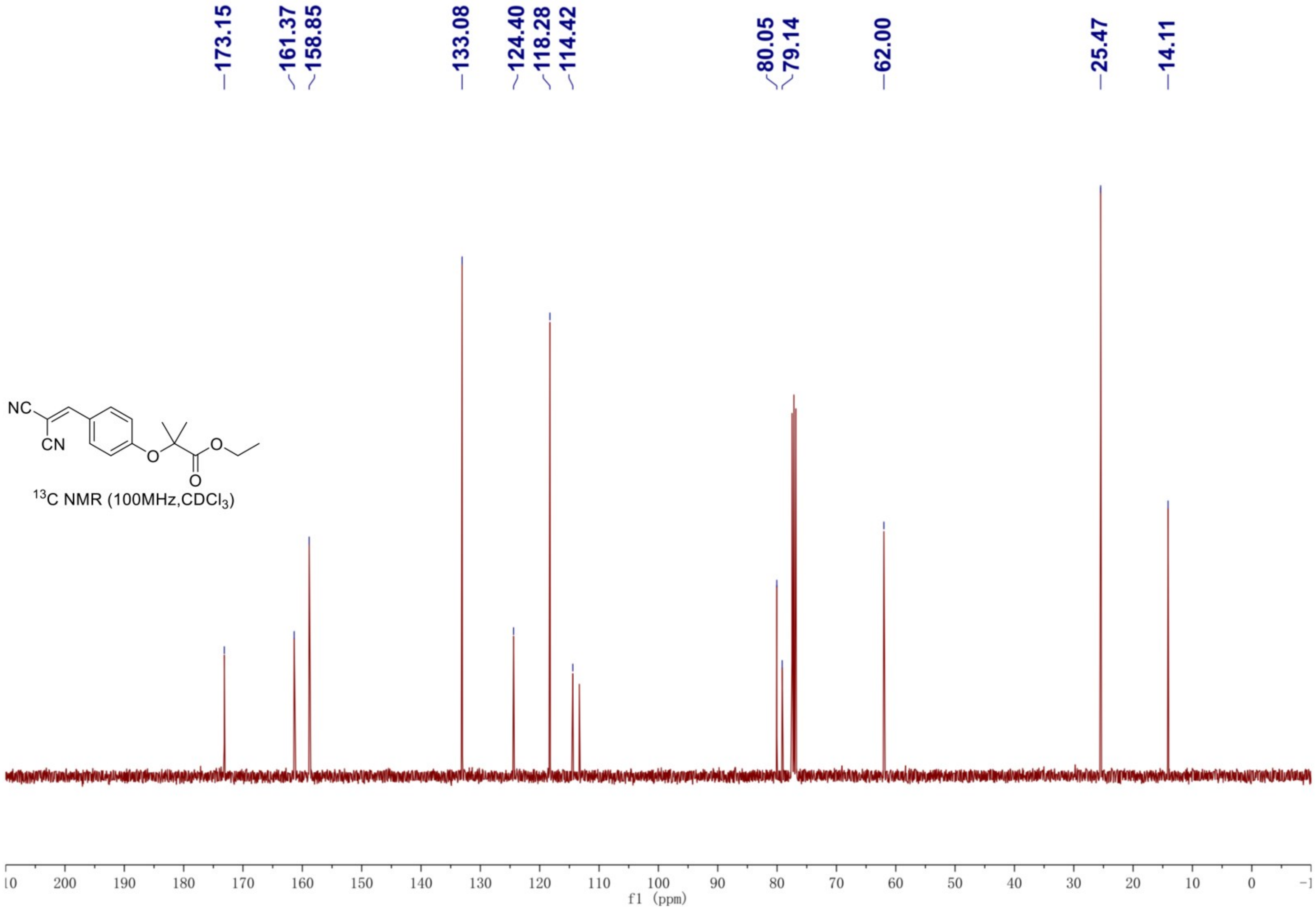

Go to table of contents

4-(2,2-Dicyanovinyl)phenyl 3-(4,5-diphenyl-4,5-dihydrooxazol-2-yl)propanoate

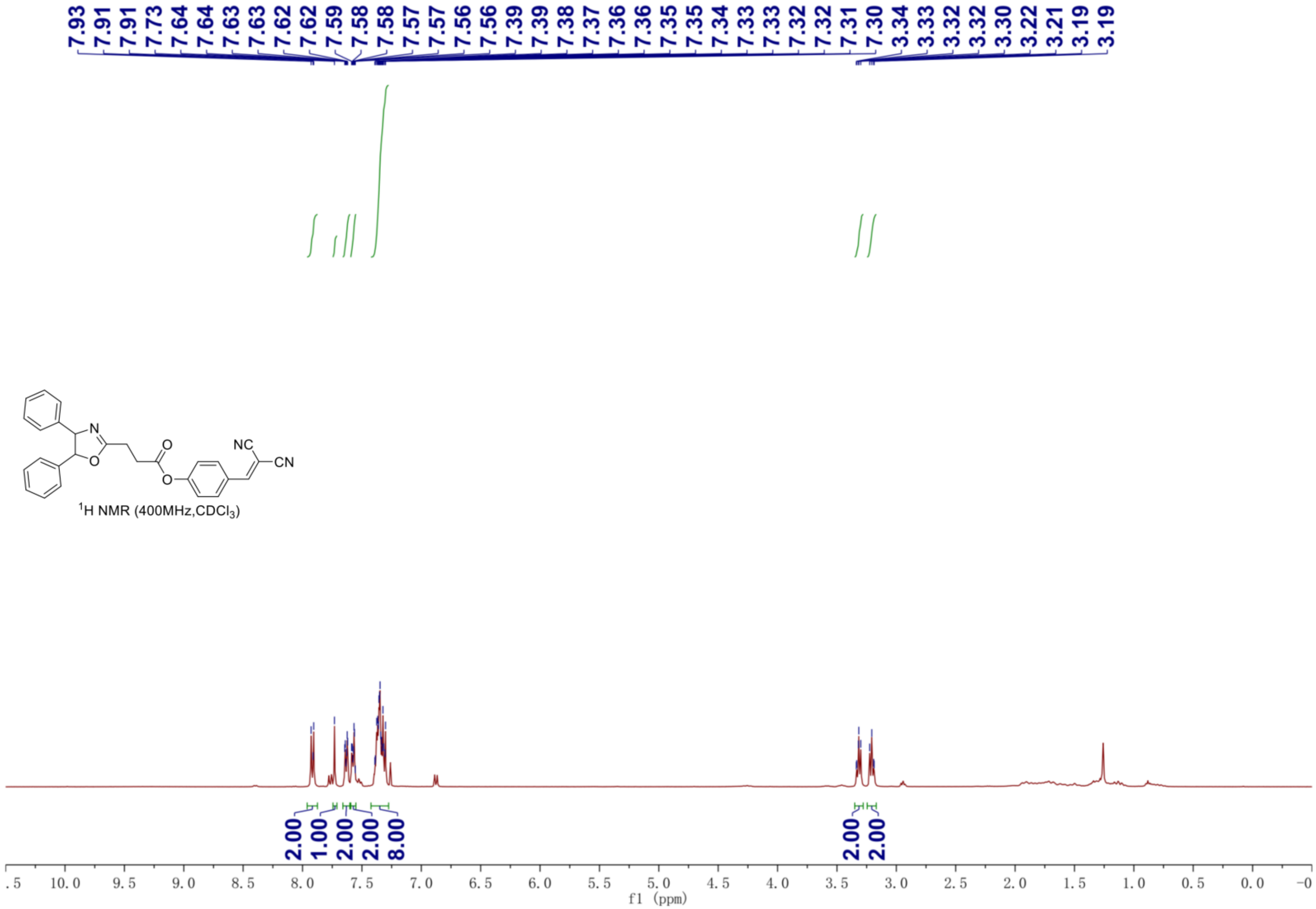

Go to table of contents

4-(2,2-Dicyanovinyl)phenyl 3-(4,5-diphenyl-4,5-dihydrooxazol-2-yl)propanoate

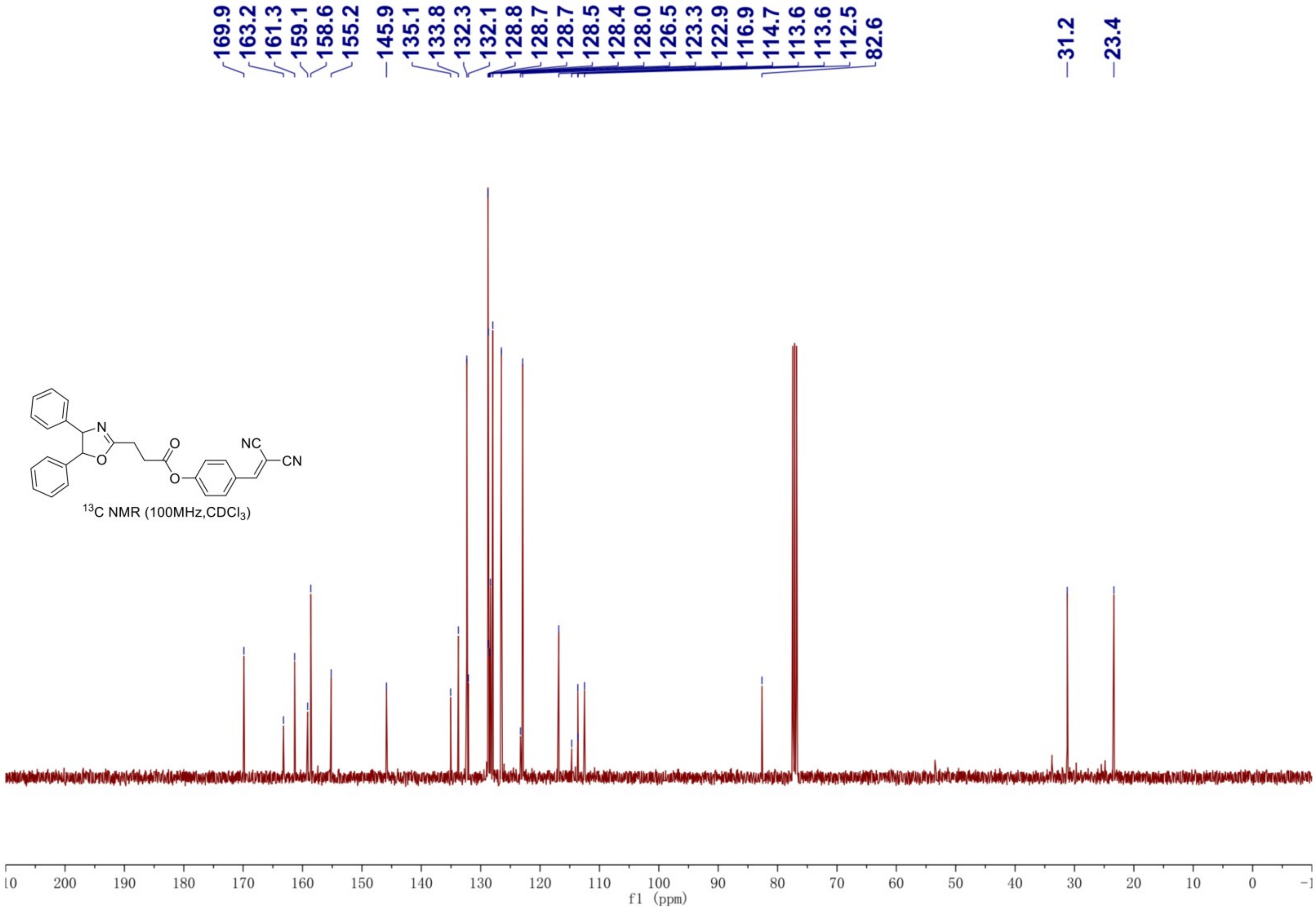

Go to table of contents

Methyl 2-(11-oxo-6,11-dihydrodibenzo[*b*,*e*]oxepin-2-yl)acrylate

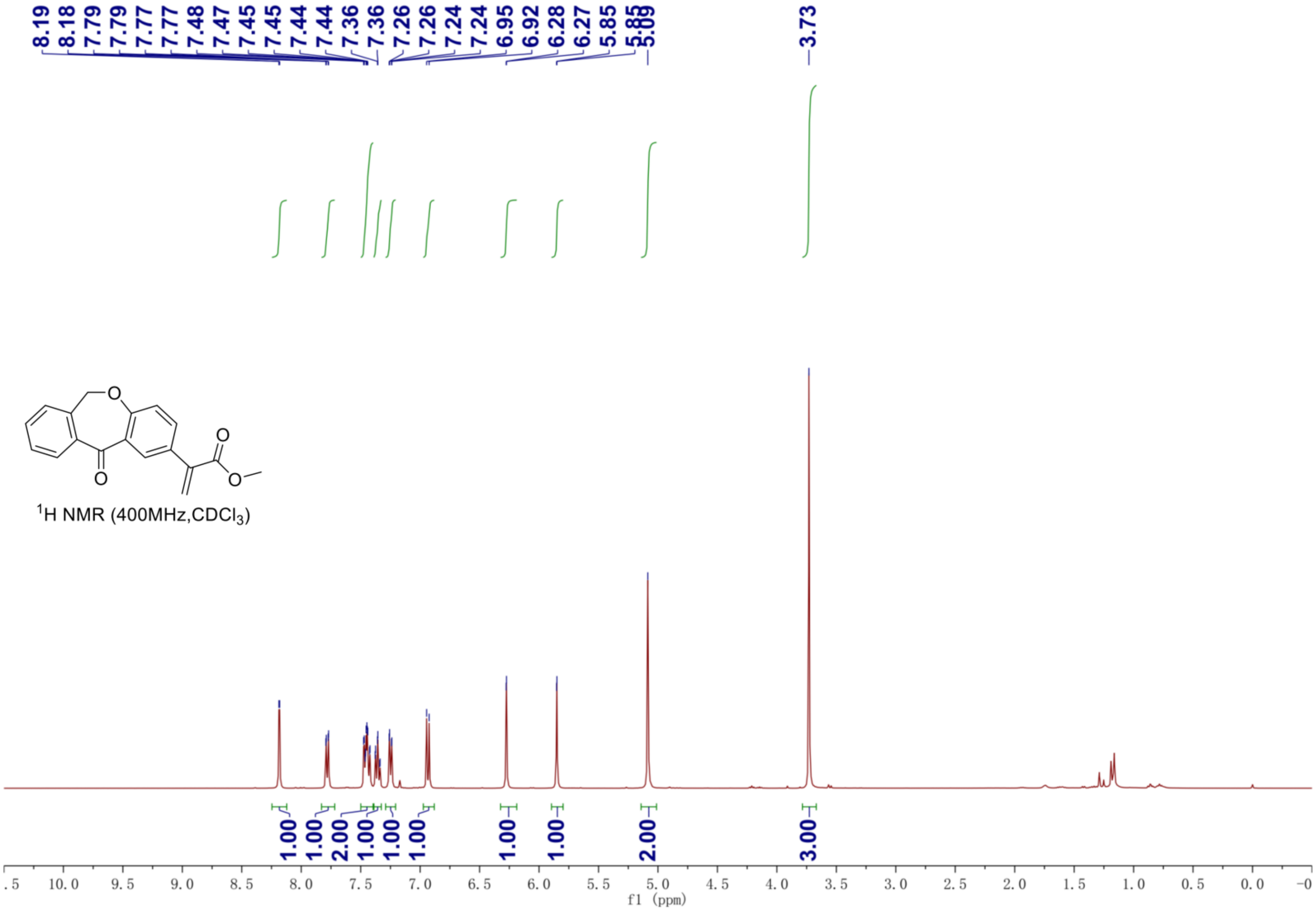

Go to table of contents

Methyl 2-(11-oxo-6,11-dihydrodibenzo[*b*,*e*]oxepin-2-yl)acrylate

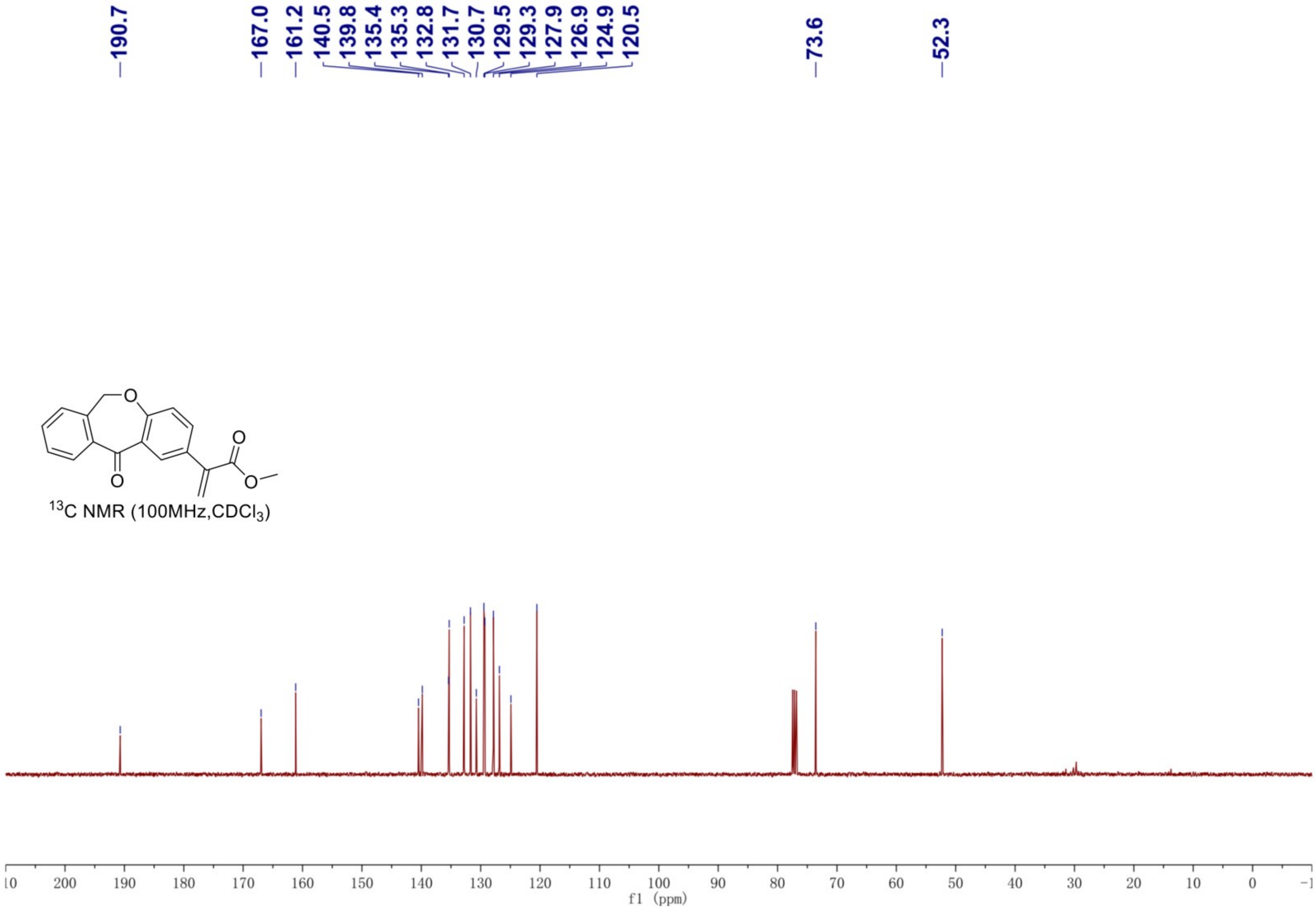

Go to table of contents

Ethyl 2-(4-isobutylphenyl)acrylate

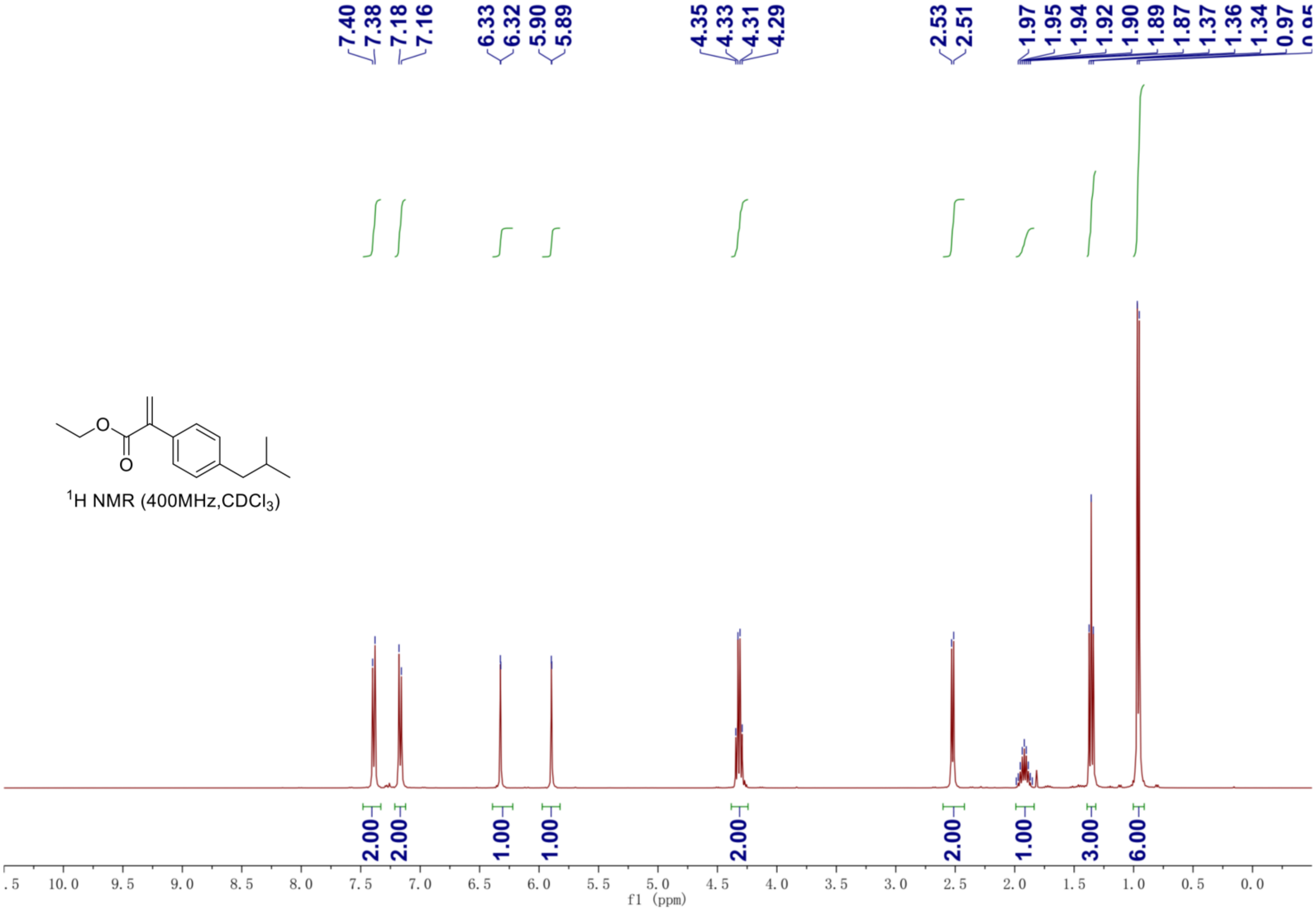

^{1}H NMR (400MHz,CDCl$_3$)

Go to table of contents

Ethyl 2-(4-isobutylphenyl)acrylate

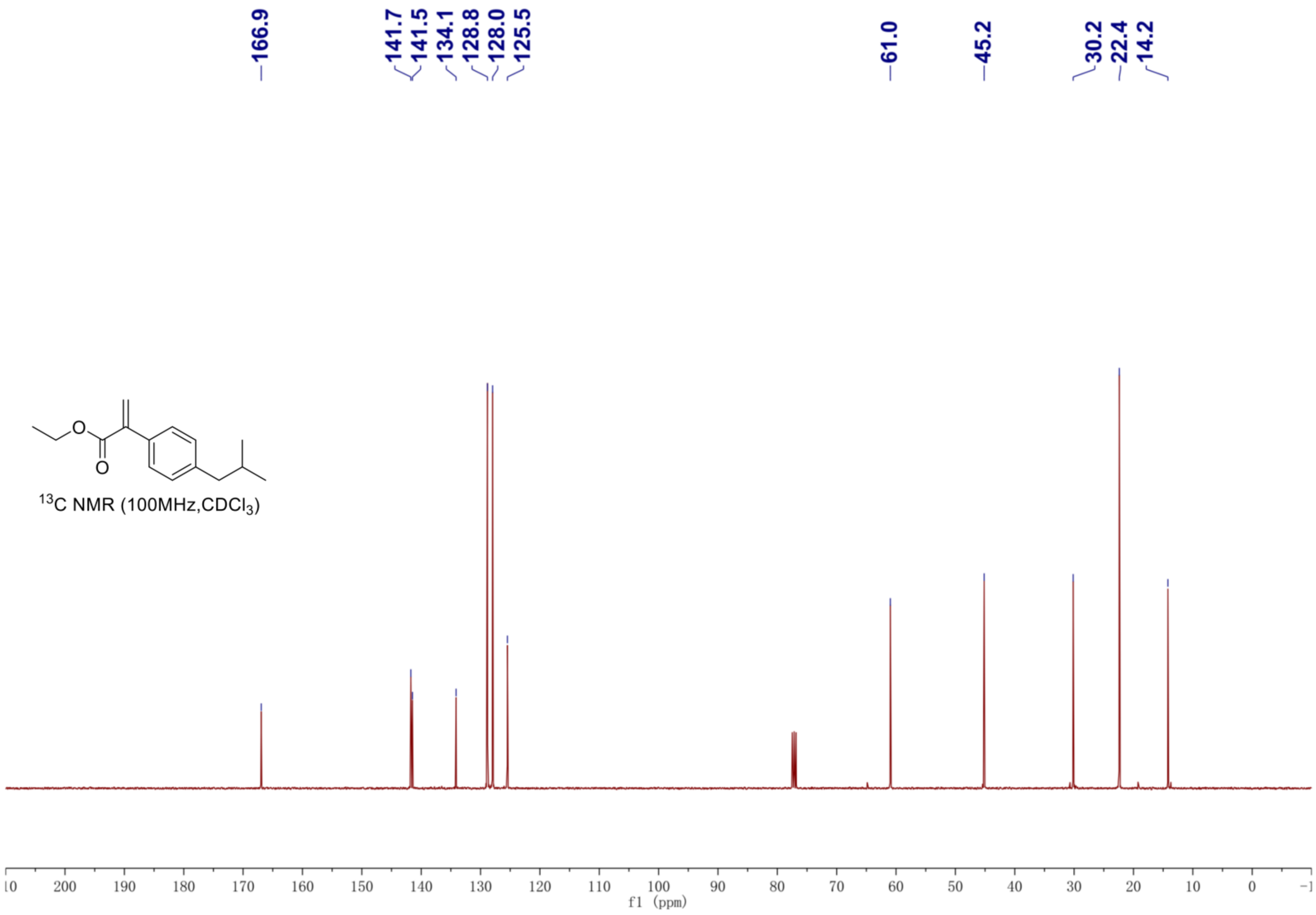

Go to table of contents

N-(4-(1-Butyl-3-ethyl-2,6-dioxopiperidin-3-yl)phenyl)-2-phenylacrylamide

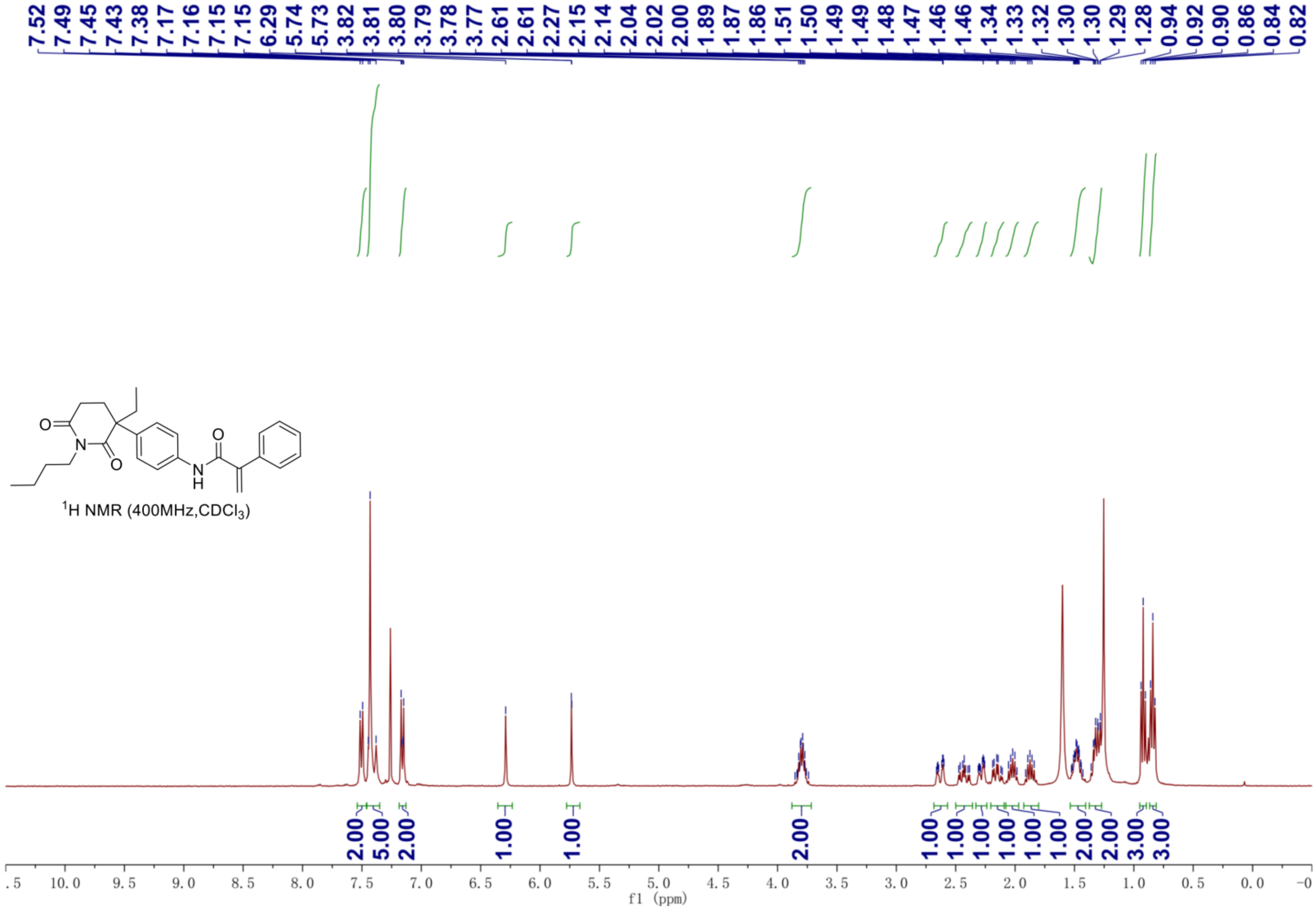

Go to table of contents

N-(4-(1-Butyl-3-ethyl-2,6-dioxopiperidin-3-yl)phenyl)-2-phenylacrylamide

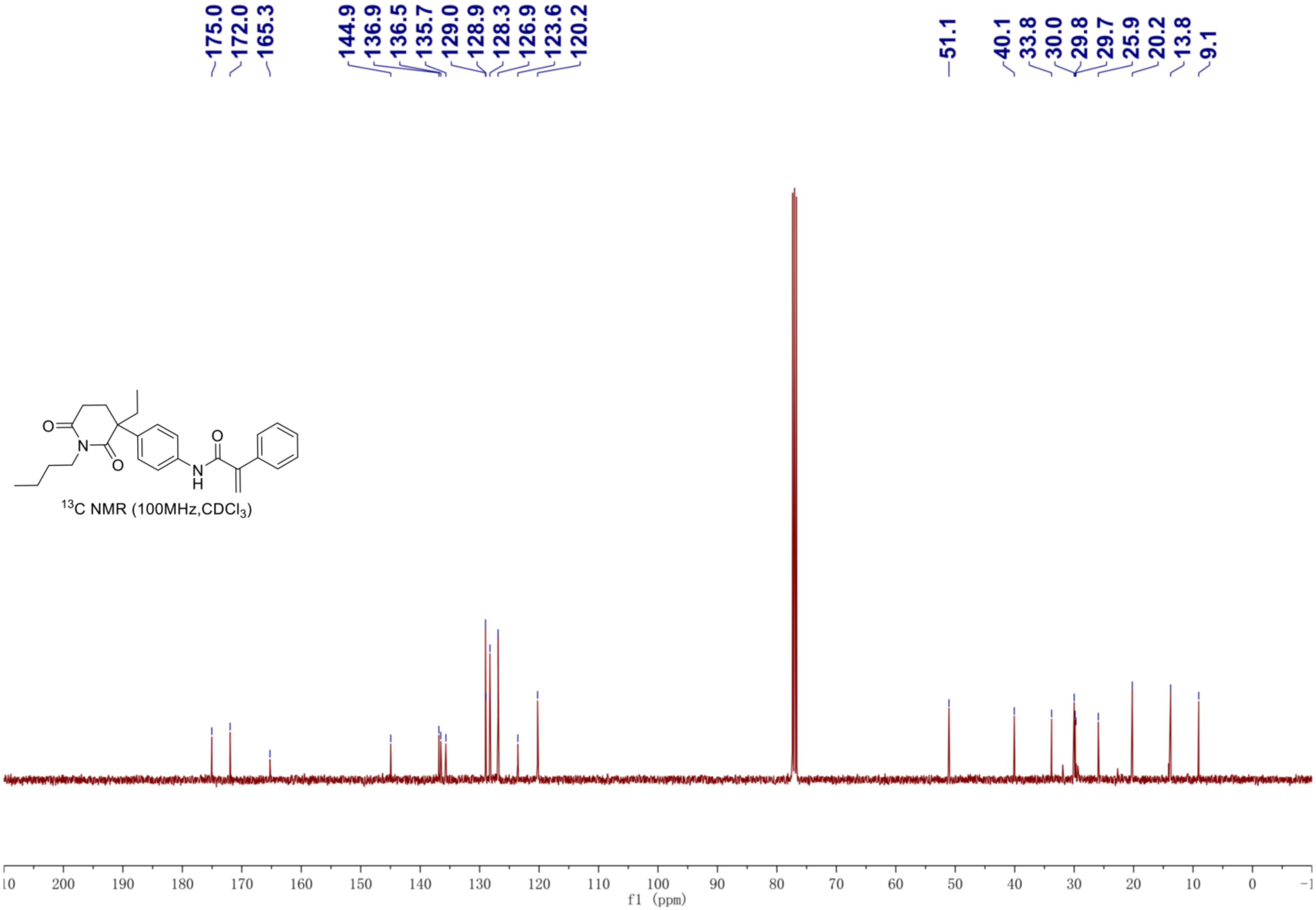

Go to table of contents

2-(Cyclohexyl(4-fluorophenyl)methyl)malononitrile (4i)

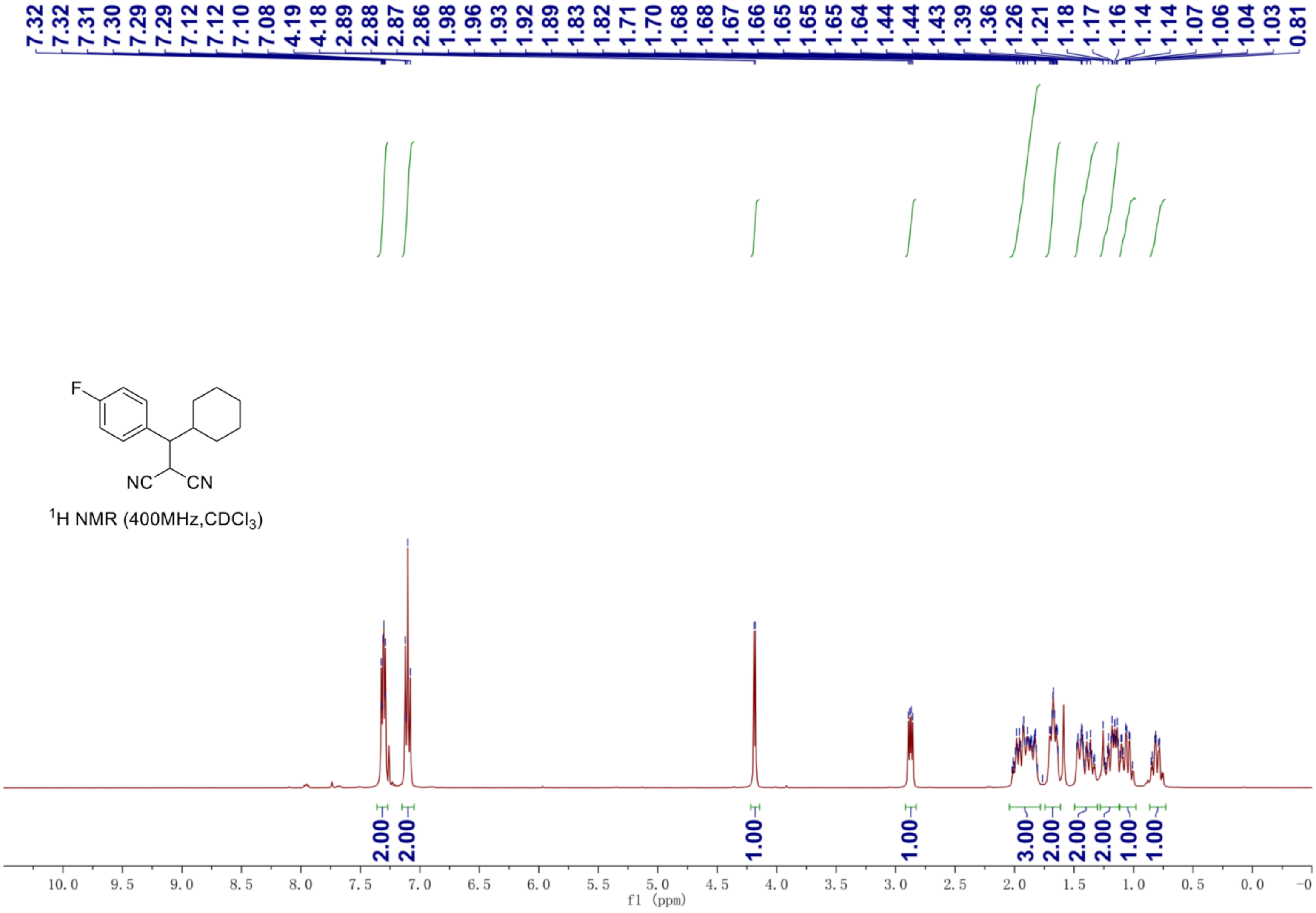

2-(Cyclohexyl(4-fluorophenyl)methyl)malononitrile (4i)

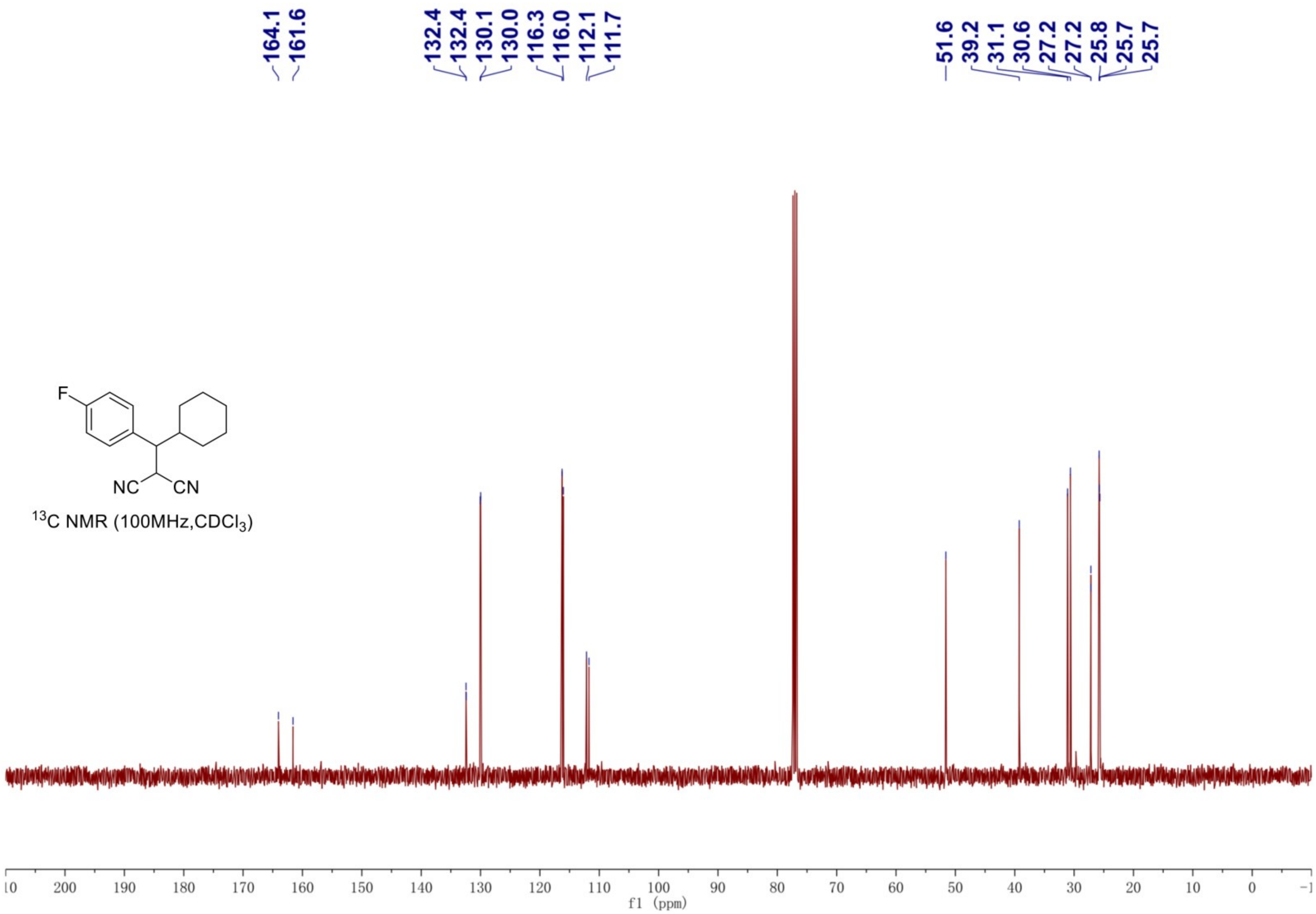

Go to table of contents

2-(Cyclohexyl(4-fluorophenyl)methyl)malononitrile (4i)

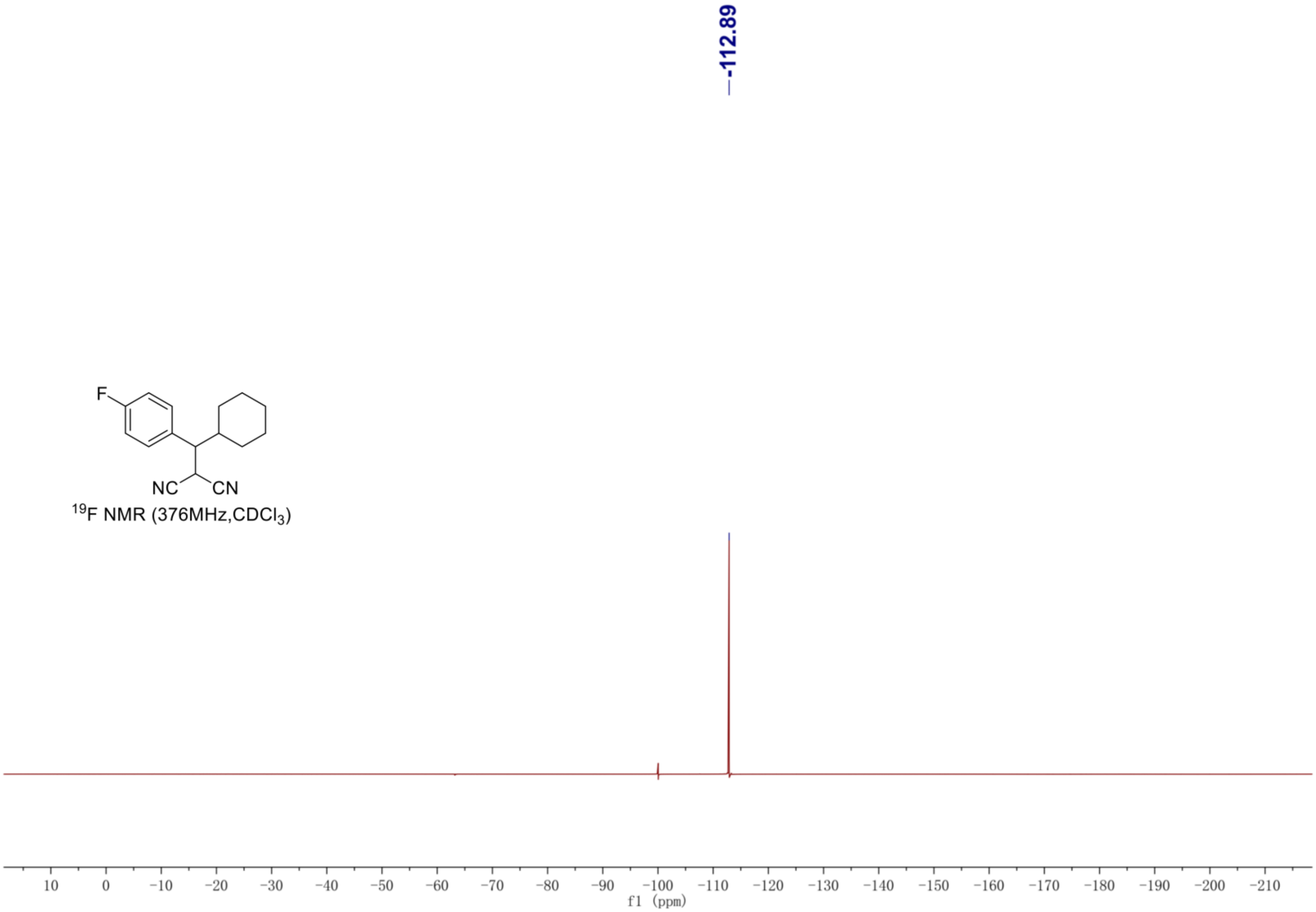

Go to table of contents

1-(Cyclohexyloxy)-2,2,6,6-tetramethylpiperidine (3a)

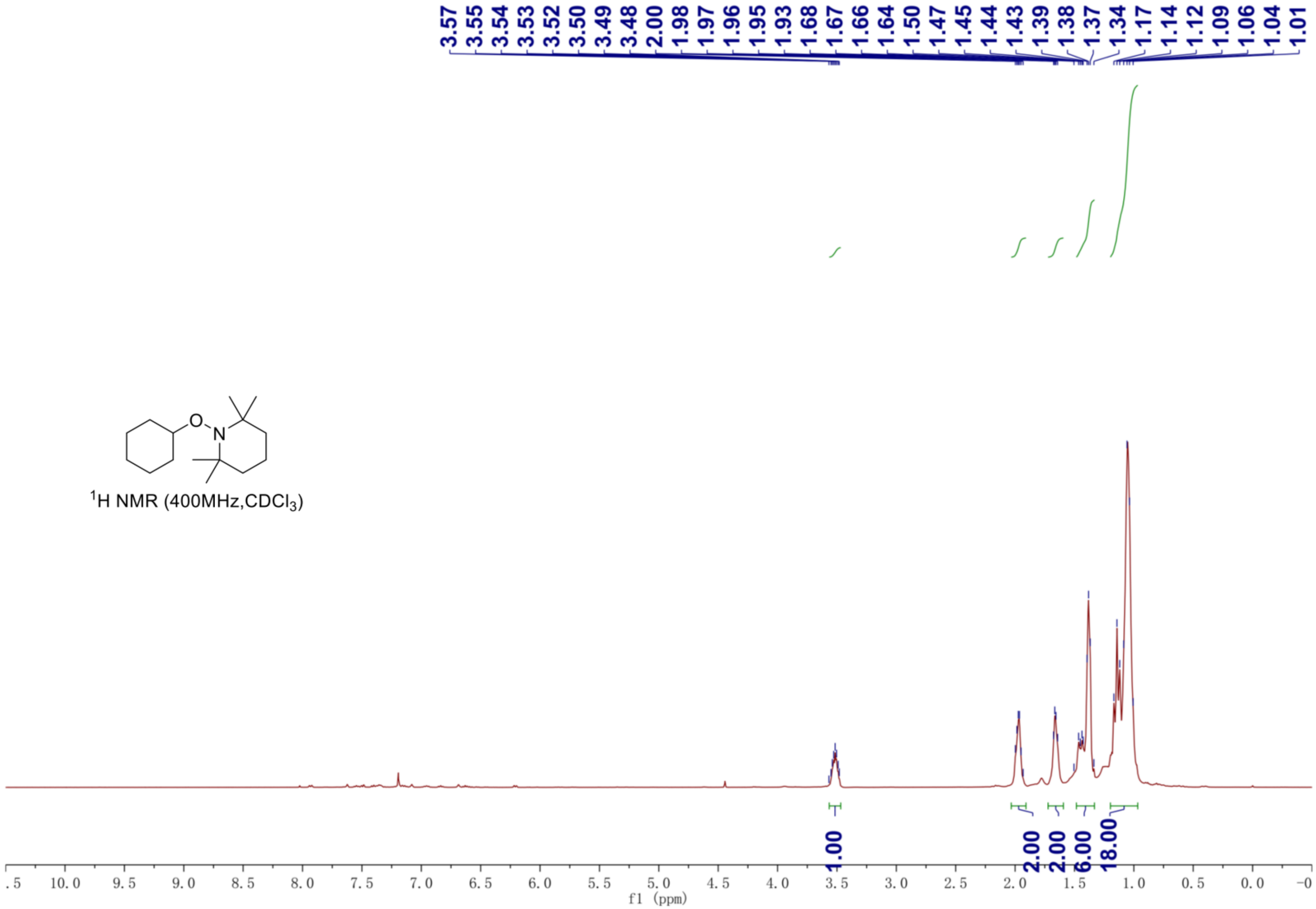

Go to table of contents

1-(Cyclohexyloxy)-2,2,6,6-tetramethylpiperidine (3a)

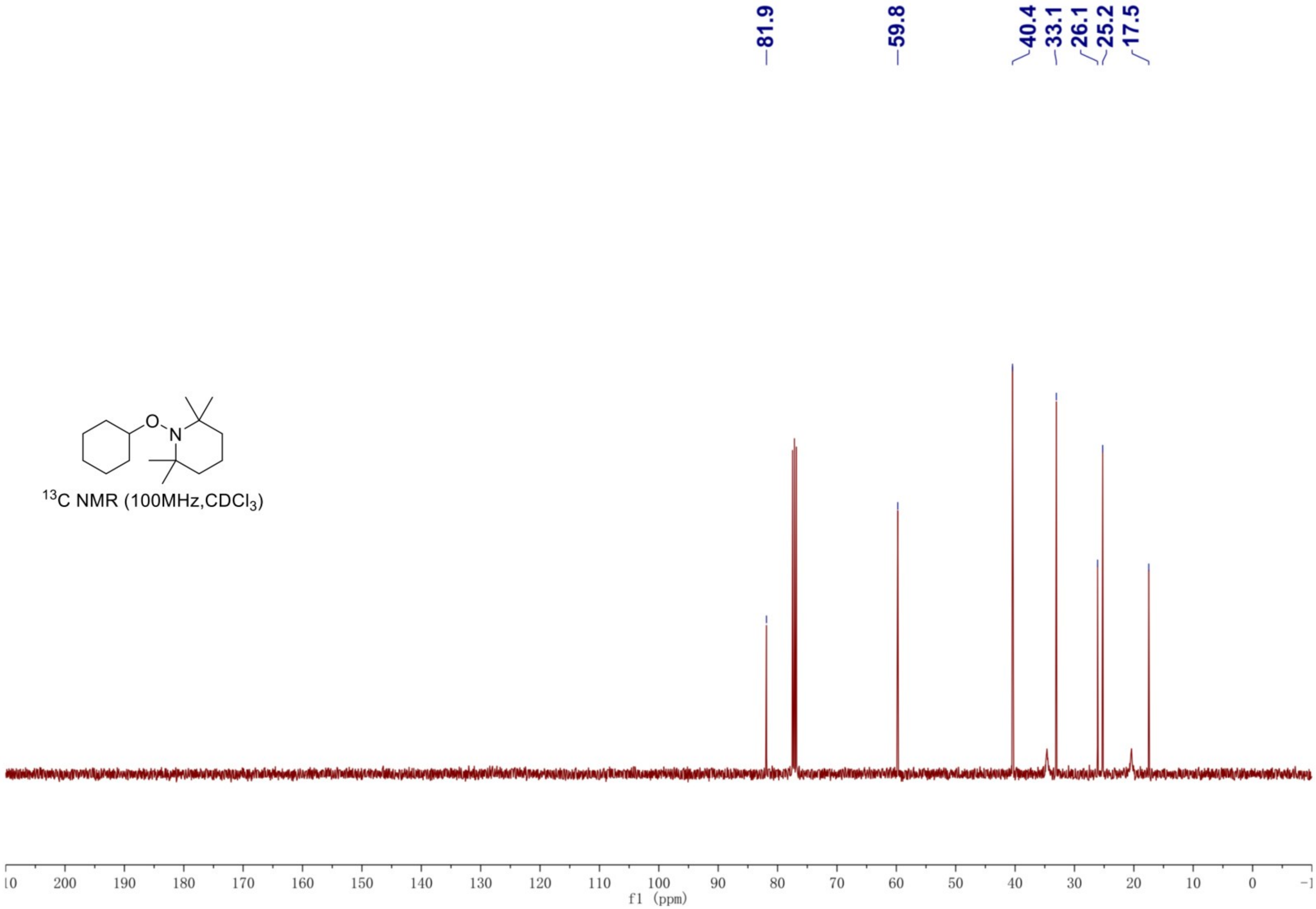

Go to table of contents

1-(Cyclopentyloxy)-2,2,6,6-tetramethylpiperidin-4-ol (3b)

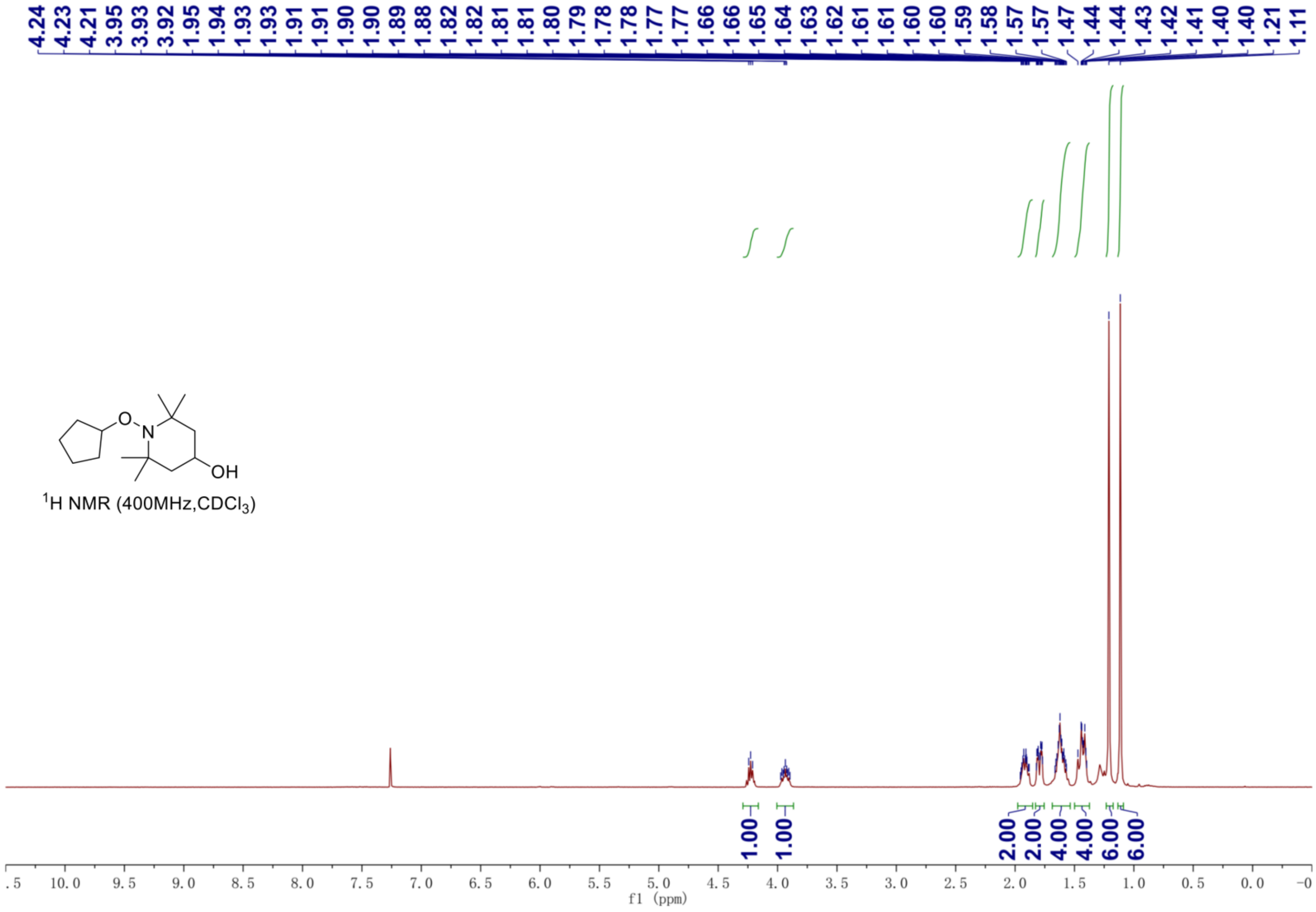

^{1}H NMR (400MHz,$CDCl_3$)

Go to table of contents

1-(Cyclopentyloxy)-2,2,6,6-tetramethylpiperidin-4-ol (3b)

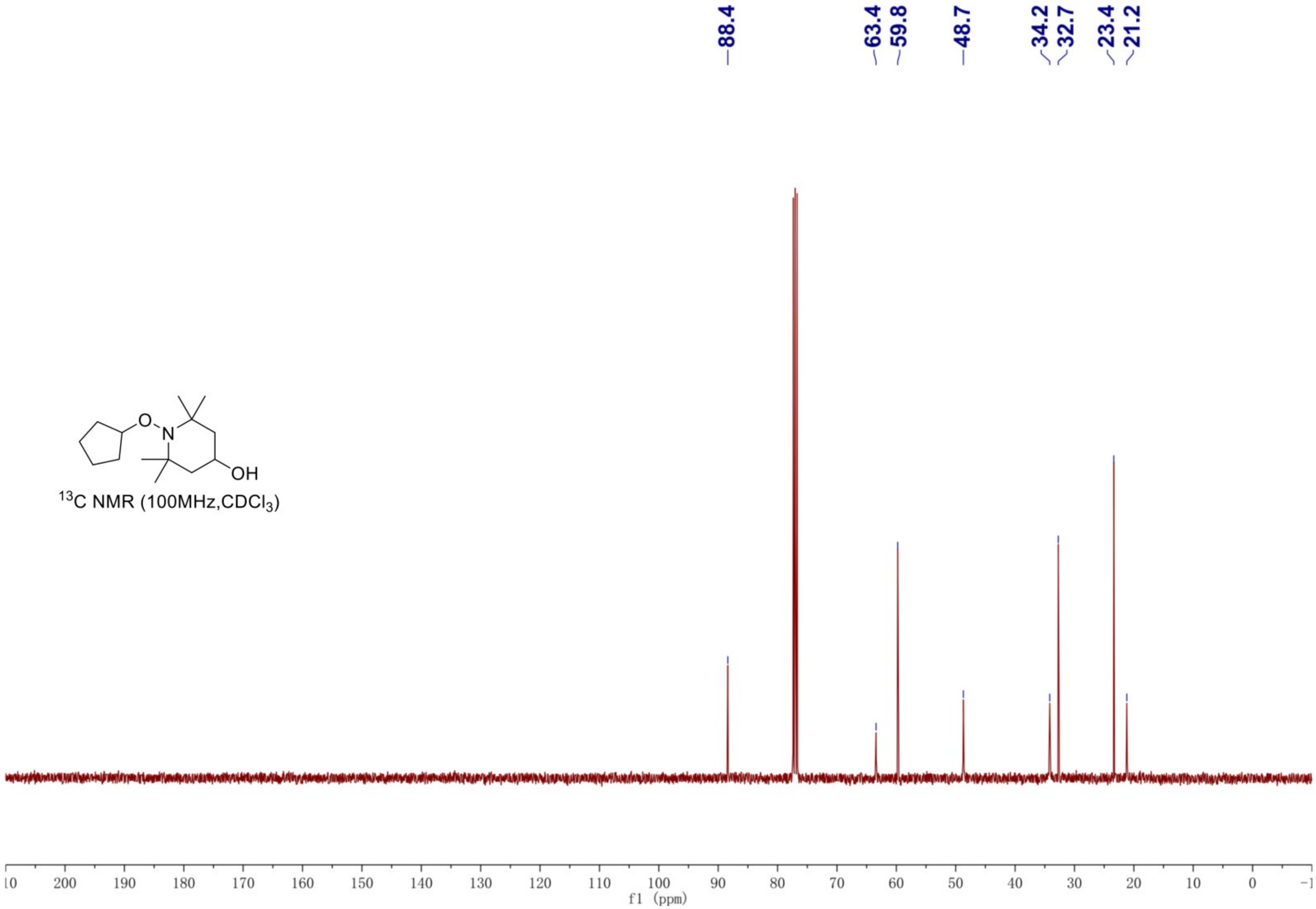

^{13}C NMR (100MHz,$CDCl_3$)

Go to table of contents

1-(Cycloheptyloxy)-2,2,6,6-tetramethylpiperidin-4-ol (3c)

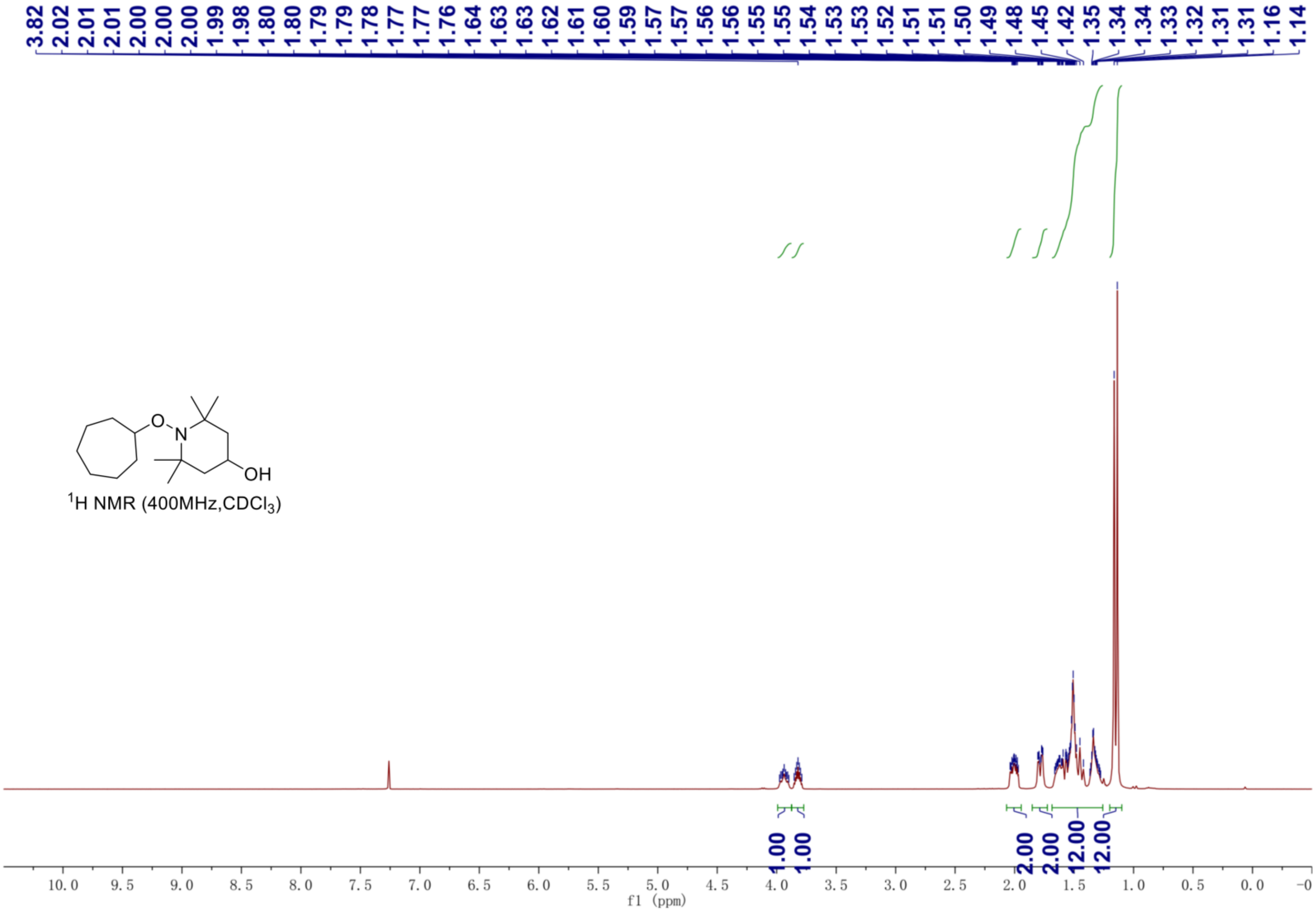

1-(Cycloheptyloxy)-2,2,6,6-tetramethylpiperidin-4-ol (3c)

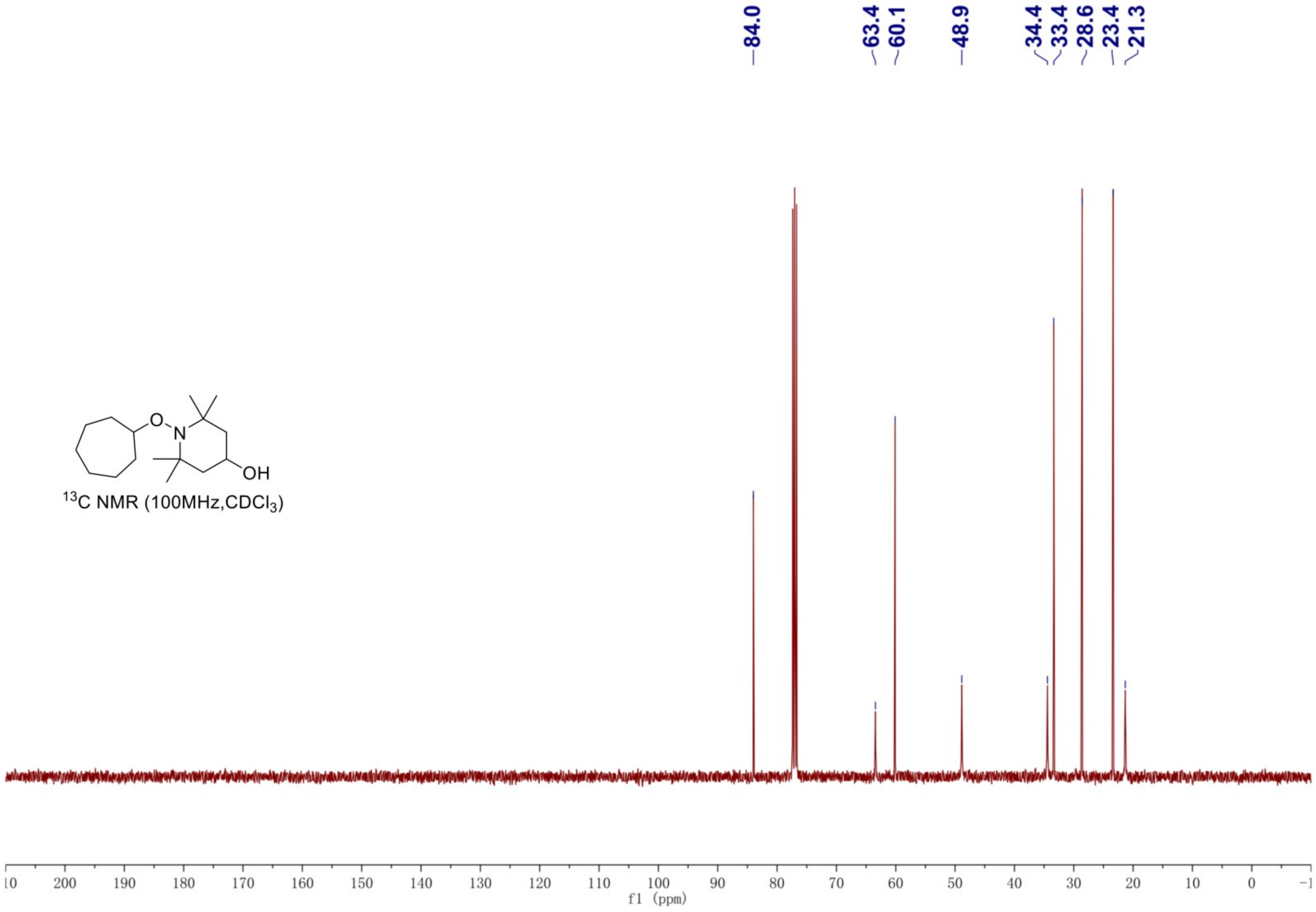

Go to table of contents

1-(Cyclooctyloxy)-2,2,6,6-tetramethylpiperidin-4-ol (3d)

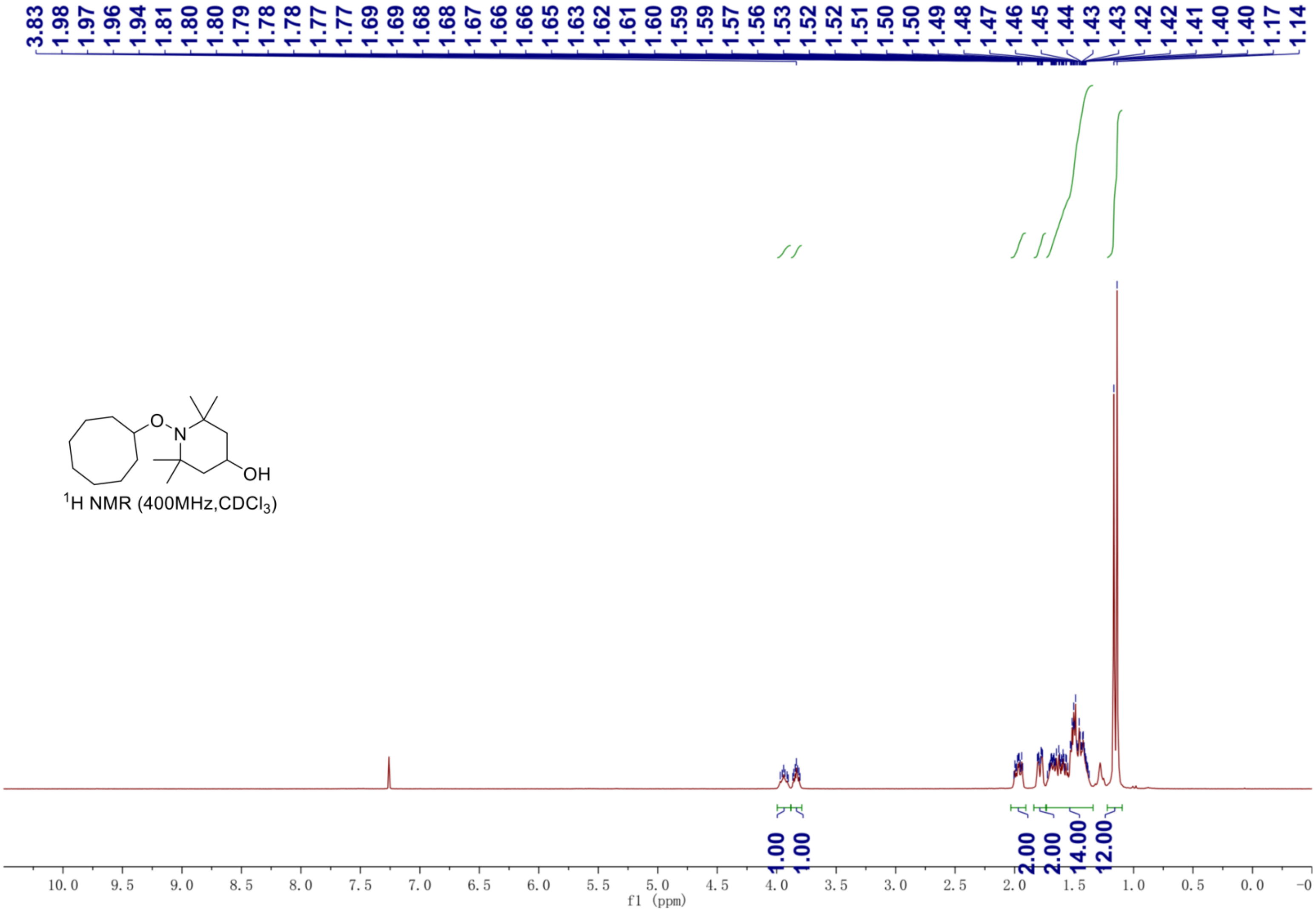

Go to table of contents

1-(Cyclooctyloxy)-2,2,6,6-tetramethylpiperidin-4-ol (3d)

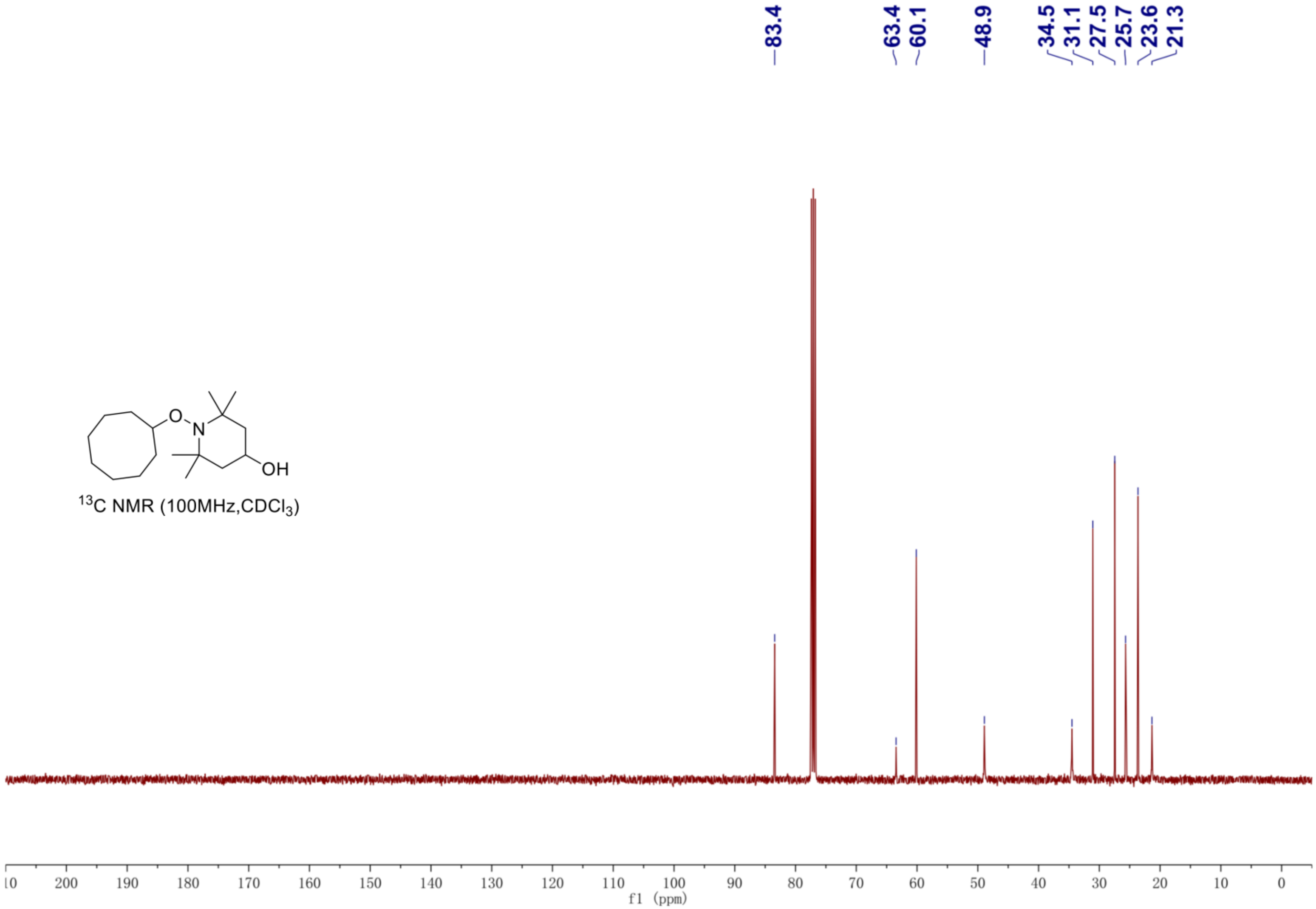

Go to table of contents

1-(2,3-Dimethylbutoxy)-2,2,6,6-tetramethylpiperidin-4-ol (3e-α)

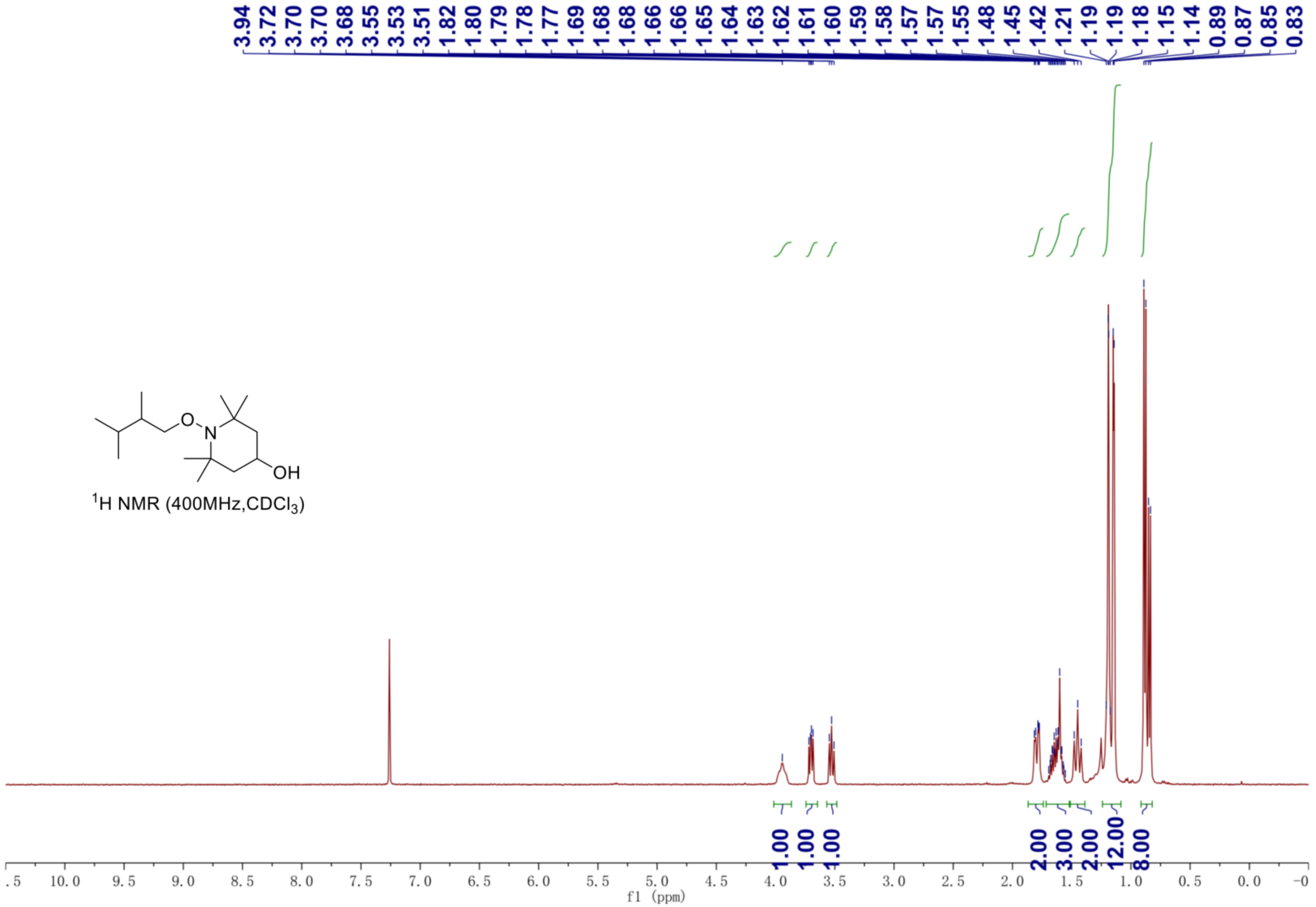

Go to table of contents

1-(2,3-Dimethylbutoxy)-2,2,6,6-tetramethylpiperidin-4-ol (3e-α)

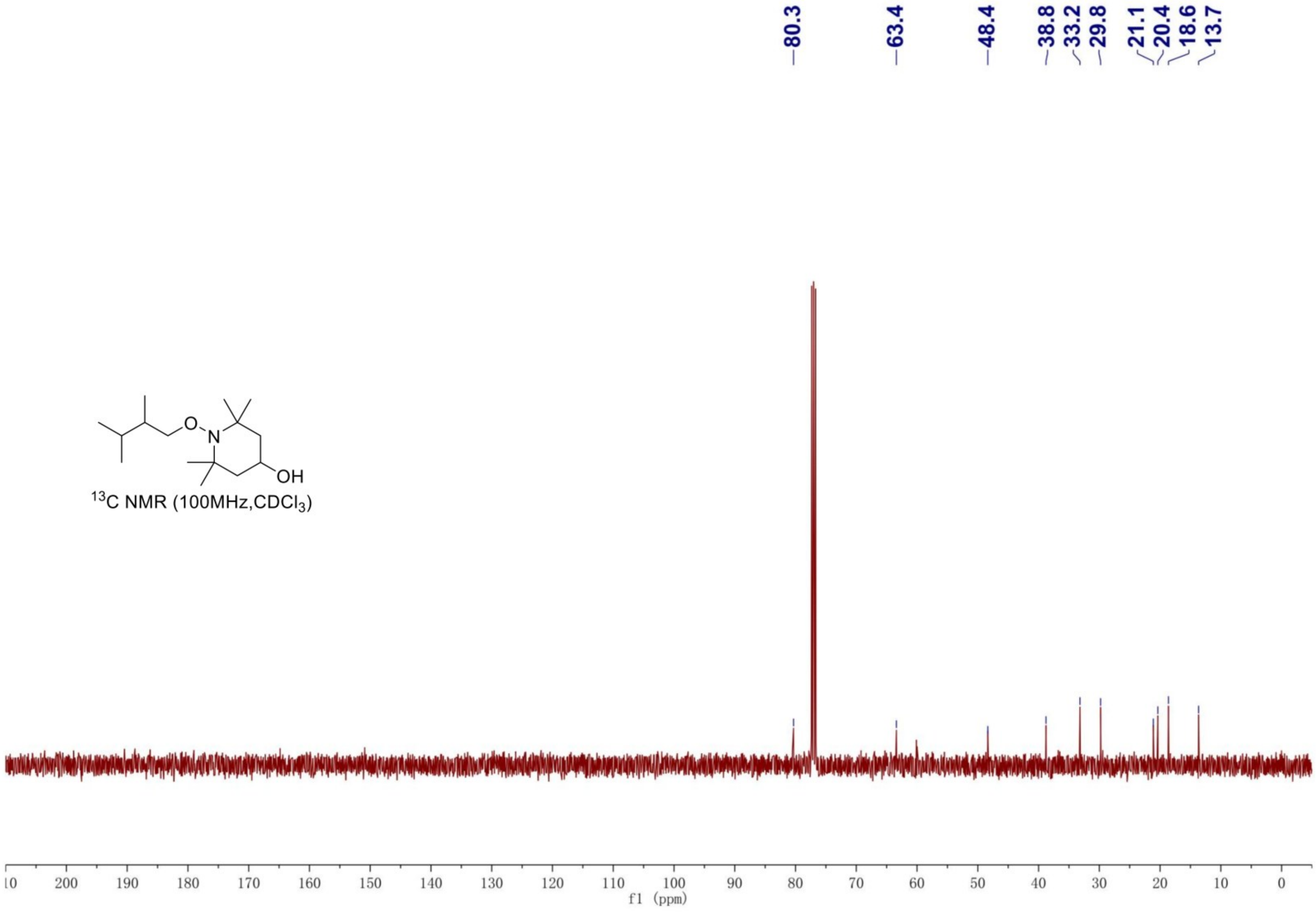

^{13}C NMR (100MHz,$CDCl_3$)

Go to table of contents

1-((2,3-Dimethylbutan-2-yl)oxy)-2,2,6,6-tetramethylpiperidin-4-ol (3e-β)

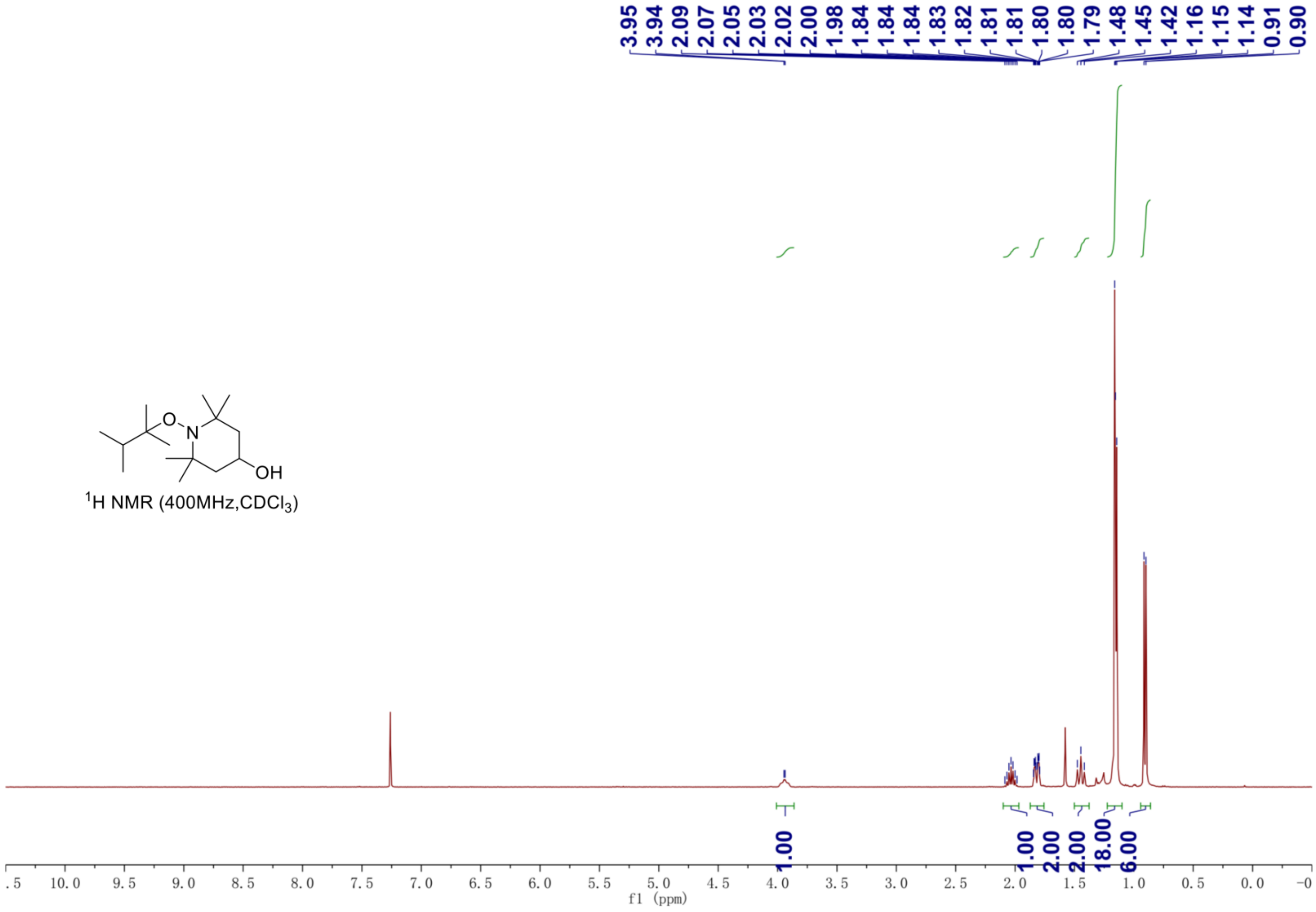

Go to table of contents

1-((2,3-Dimethylbutan-2-yl)oxy)-2,2,6,6-tetramethylpiperidin-4-ol (3e-β)

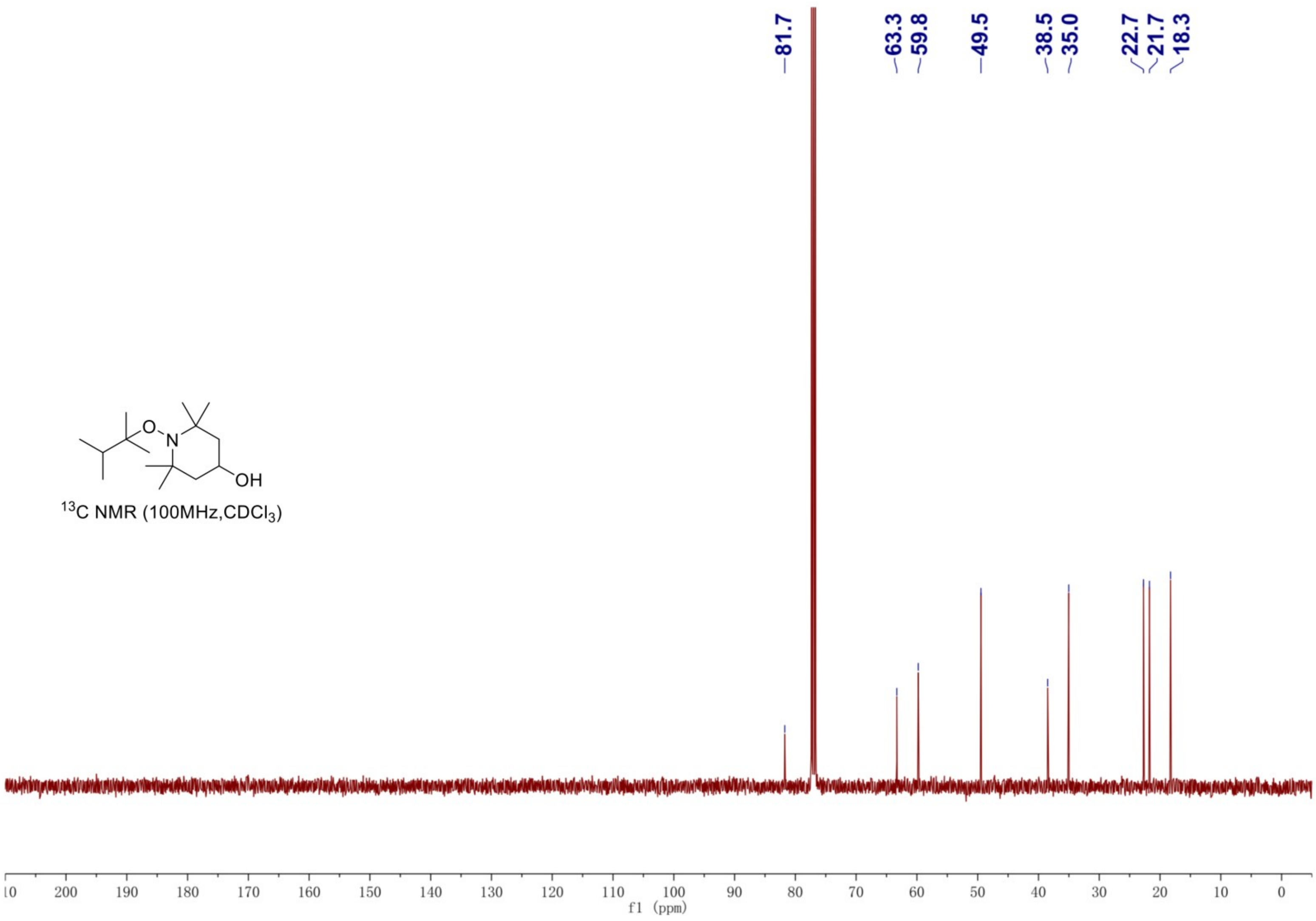

1-(((3*S*,5*S*,7*S*)-Adamantan-1-yl)oxy)-2,2,6,6-tetramethylpiperidin-4-ol (3f-*α*)

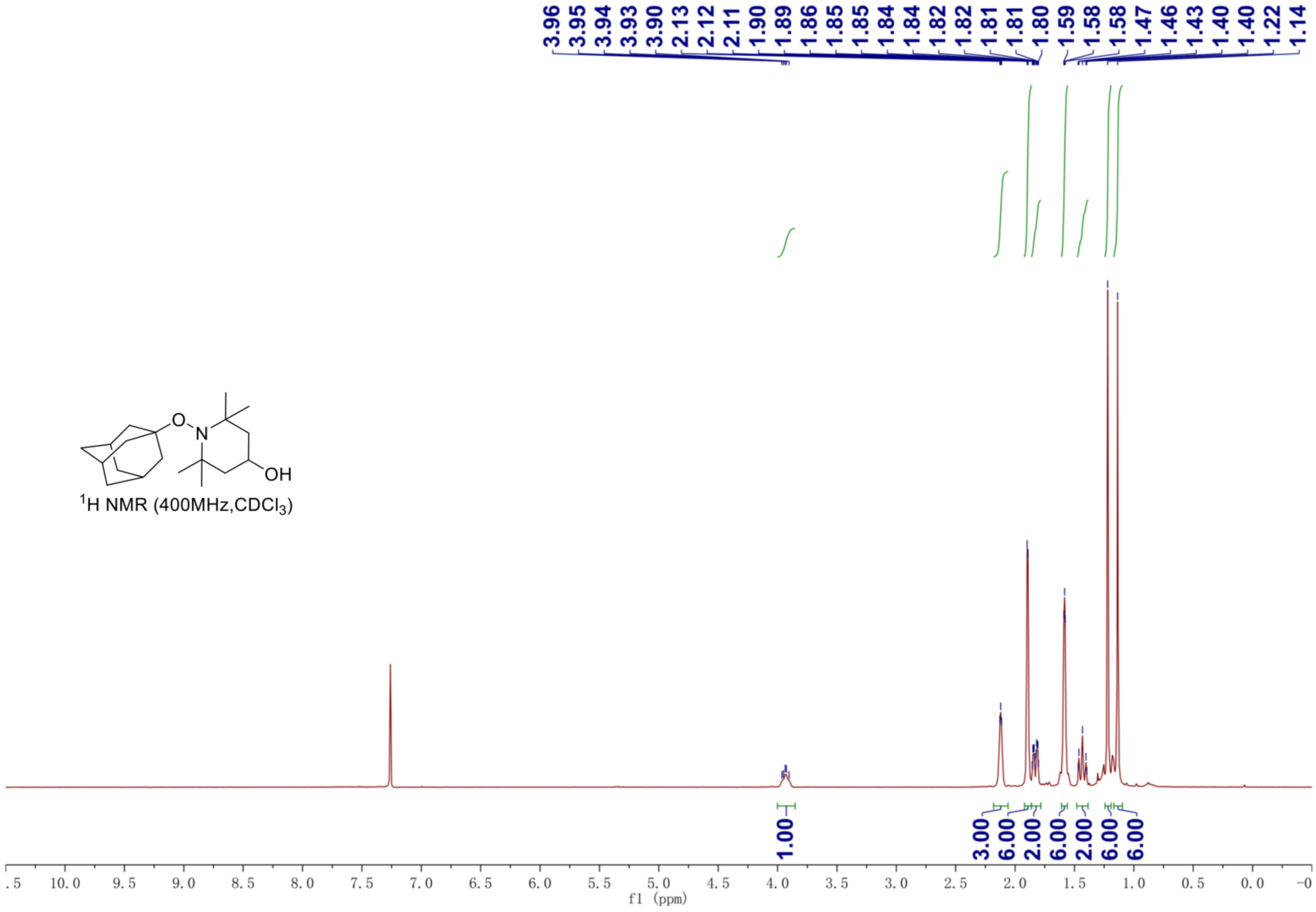

Go to table of contents

1-(((3*S*,5*S*,7*S*)-Adamantan-1-yl)oxy)-2,2,6,6-tetramethylpiperidin-4-ol (3f-*α*)

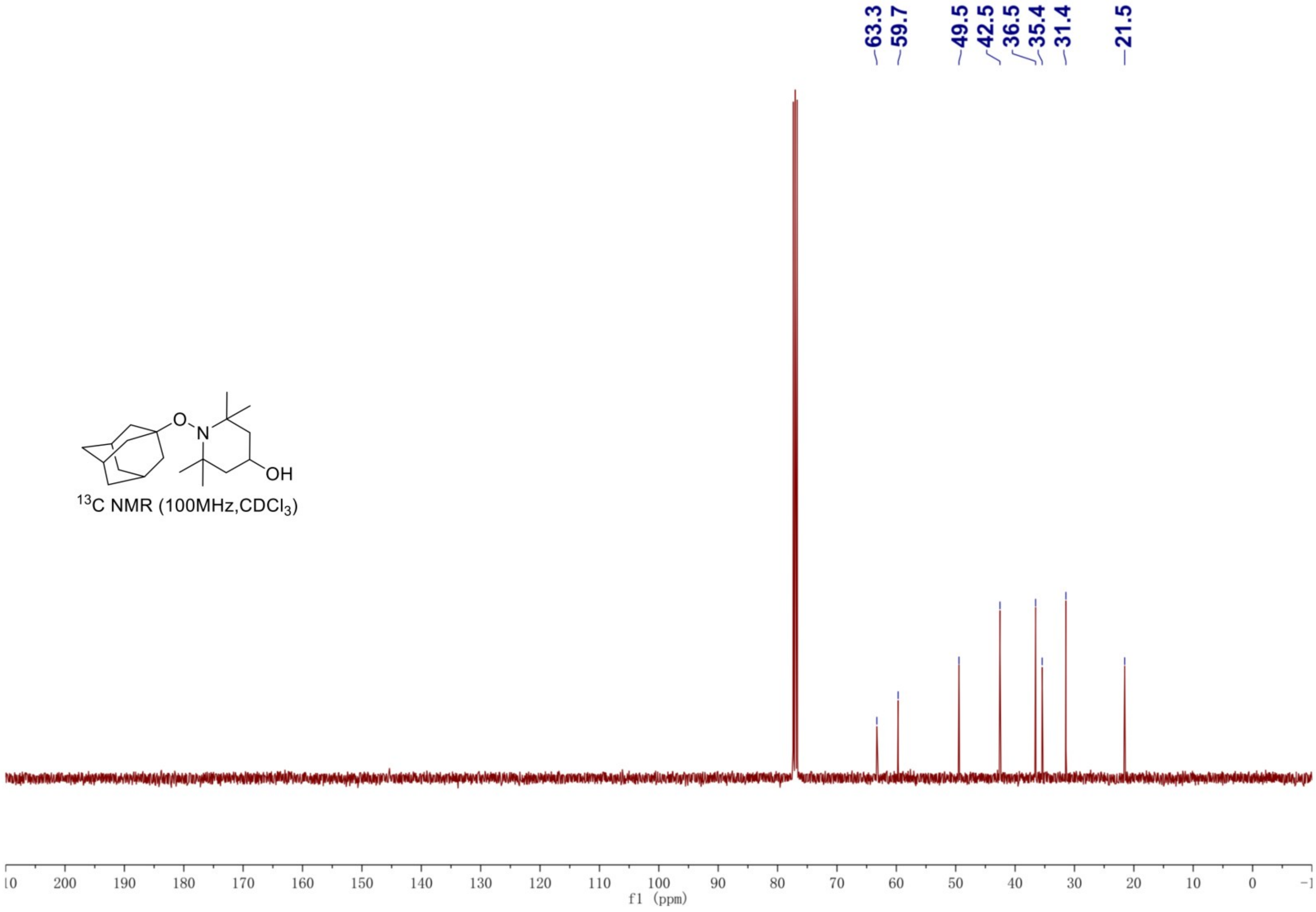

Go to table of contents

1-(((1*R*,3*R*,5*R*,7*R*)-Adamantan-2-yl)oxy)-2,2,6,6-tetramethylpiperidin-4-ol (3f-β)

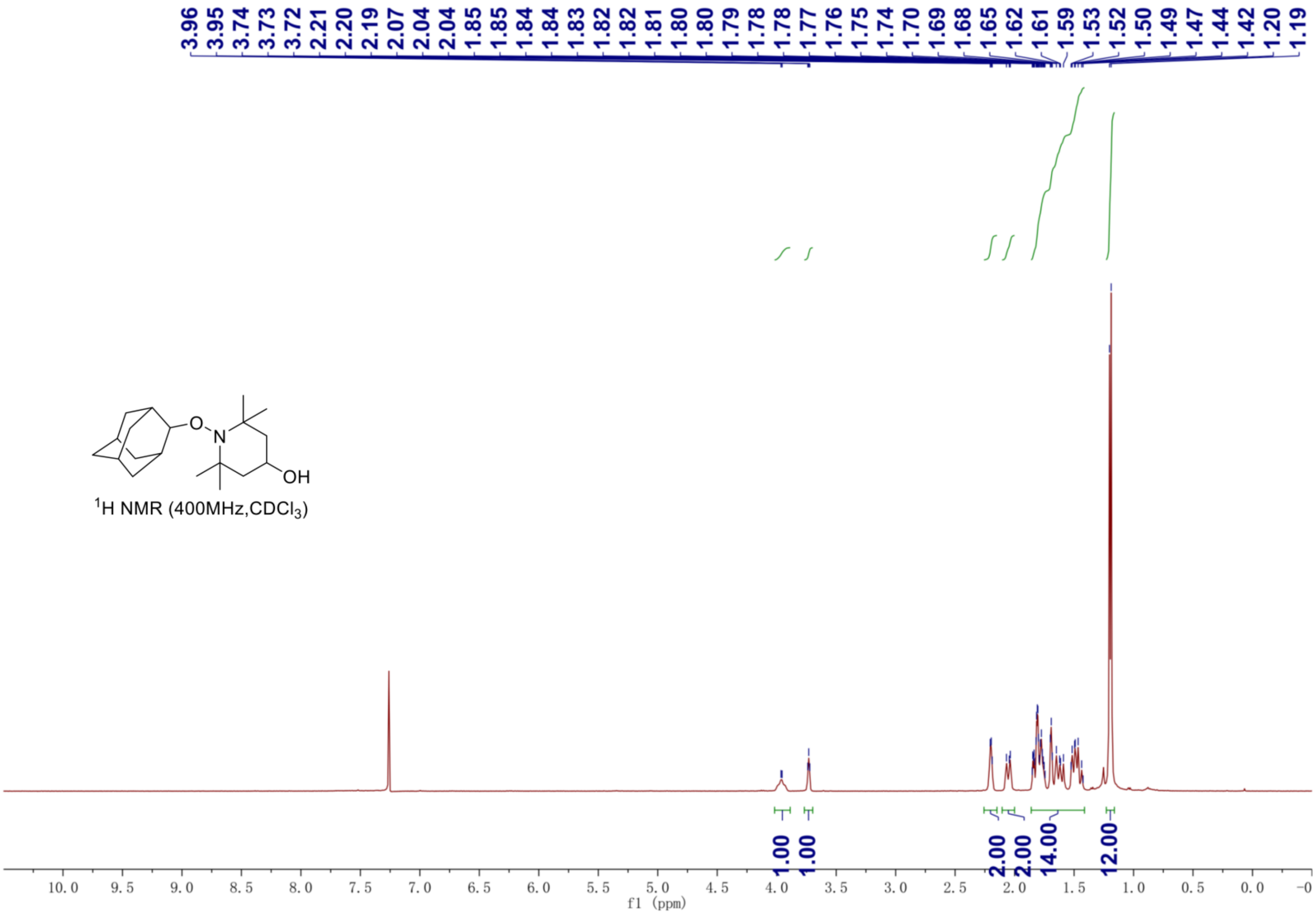

Go to table of contents

1-(((1*R*,3*R*,5*R*,7*R*)-Adamantan-2-yl)oxy)-2,2,6,6-tetramethylpiperidin-4-ol (3f-β)

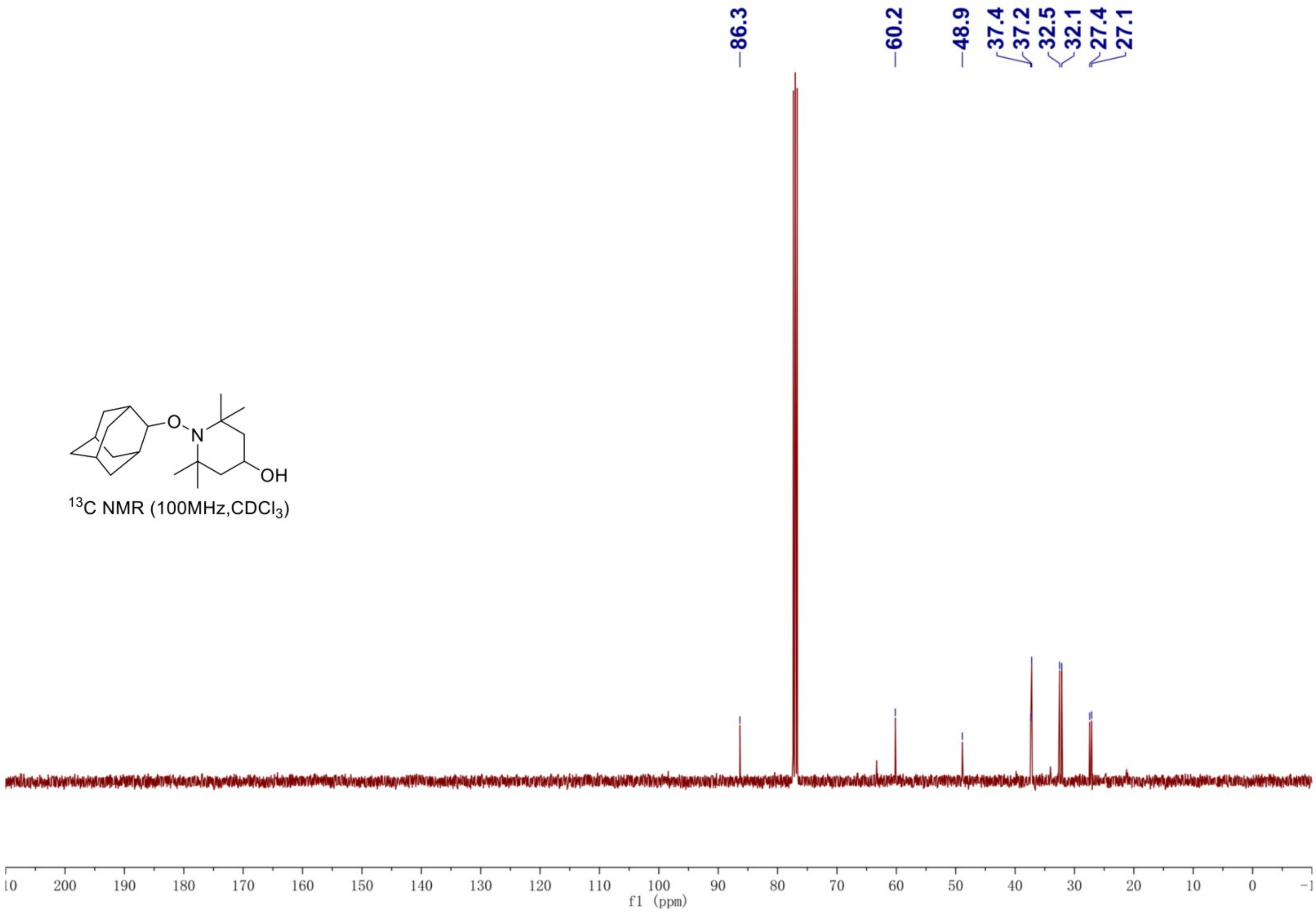

Go to table of contents

1-((2,3-Dimethylbut-2-en-1-yl)oxy)-2,2,6,6-tetramethylpiperidin-4-ol (3g-α)

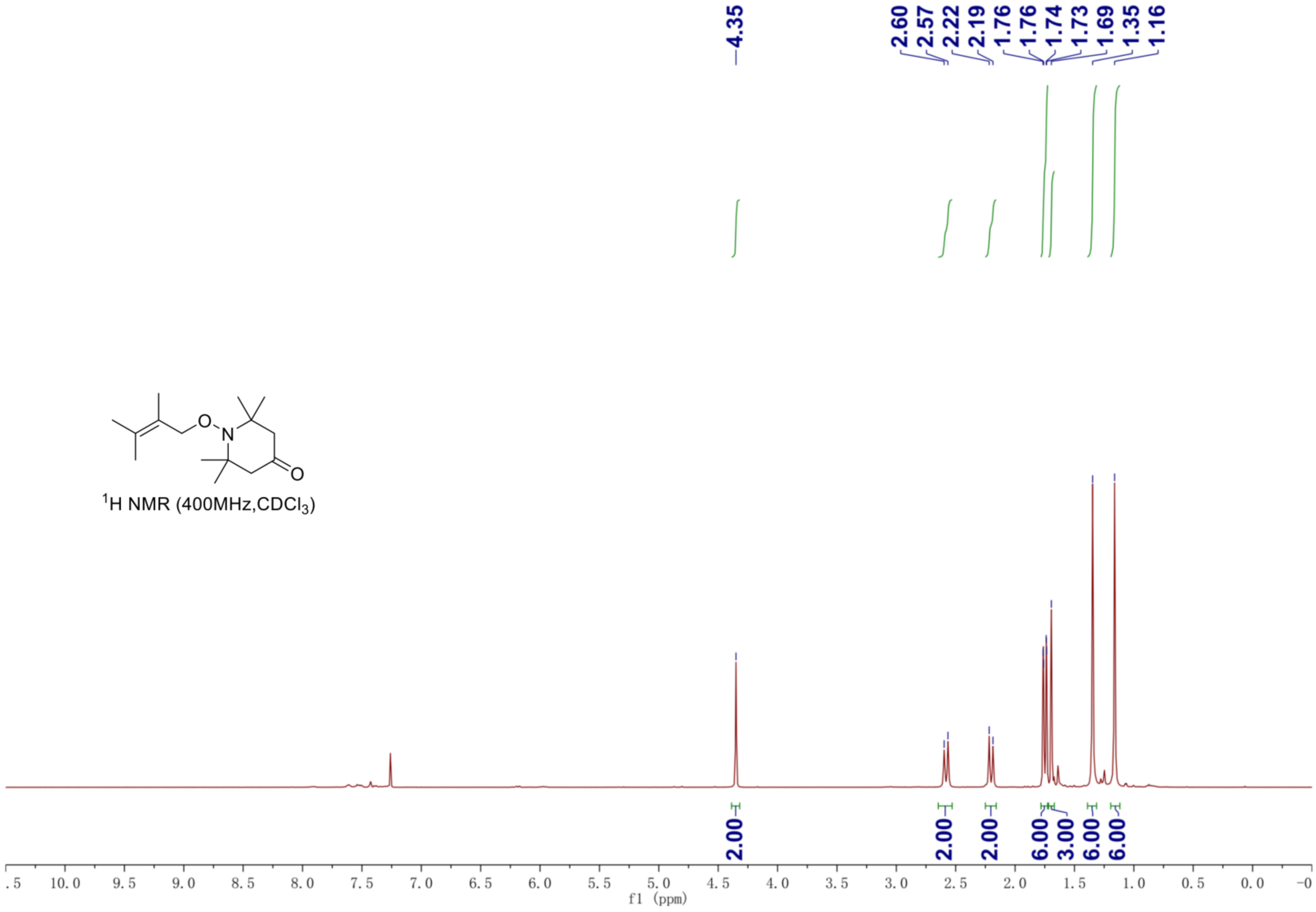

Go to table of contents

1-((2,3-Dimethylbut-2-en-1-yl)oxy)-2,2,6,6-tetramethylpiperidin-4-ol (3g-α)

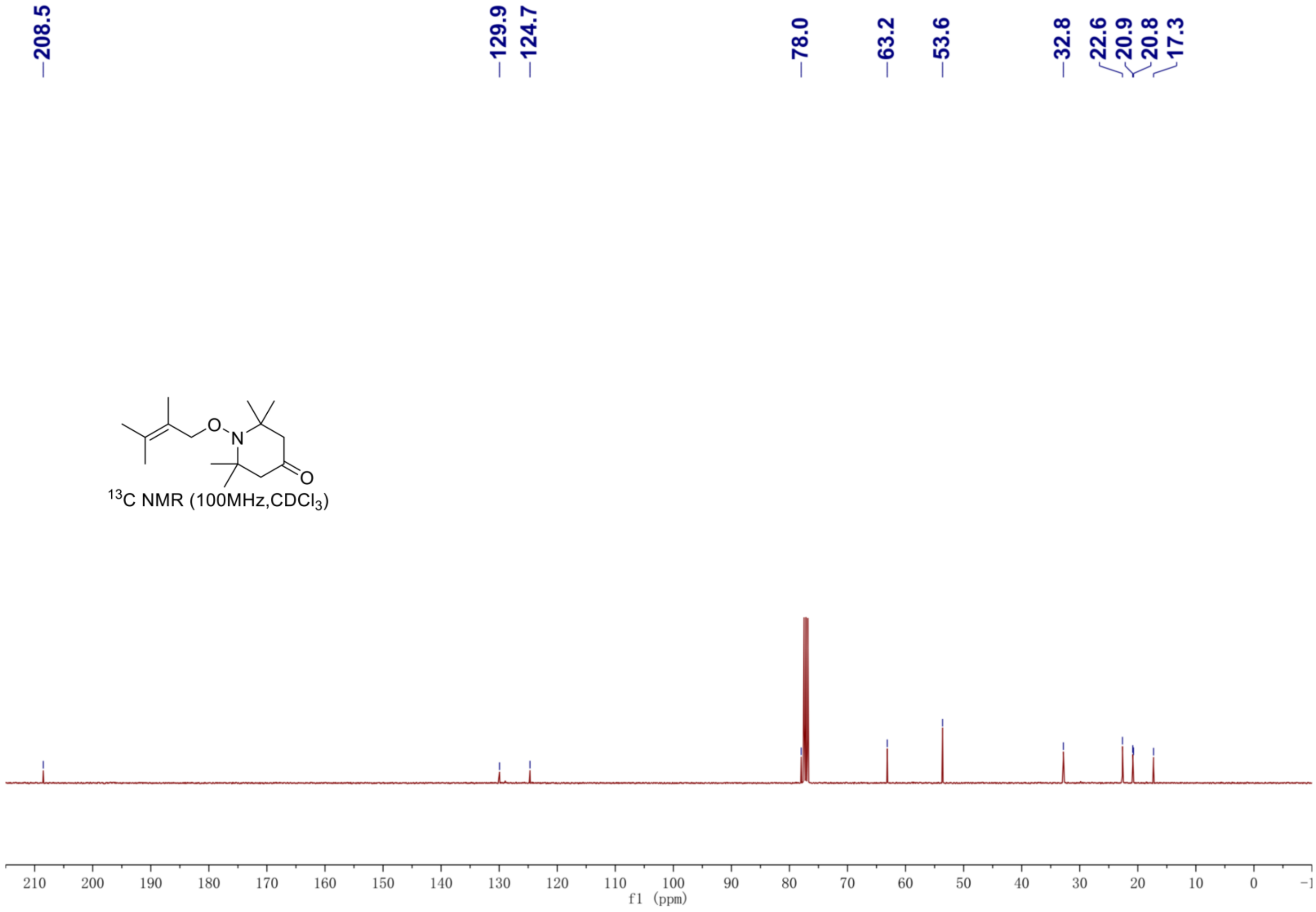

Go to table of contents

1-((2,3-dimethylbut-3-en-2-yl)oxy)-2,2,6,6-tetramethylpiperidin-4-ol (3g-β)

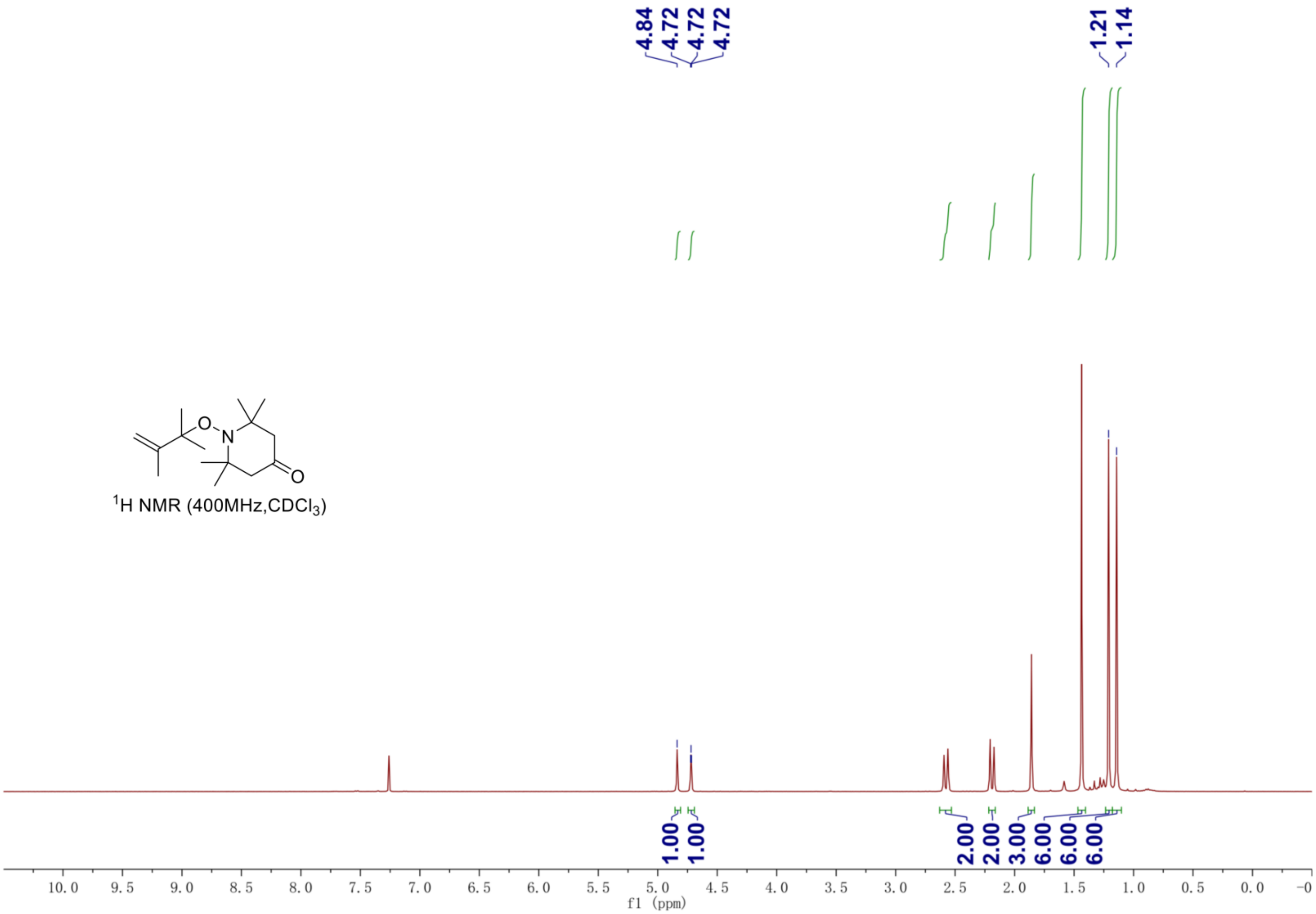

1-((2,3-dimethylbut-3-en-2-yl)oxy)-2,2,6,6-tetramethylpiperidin-4-ol (3g-β)

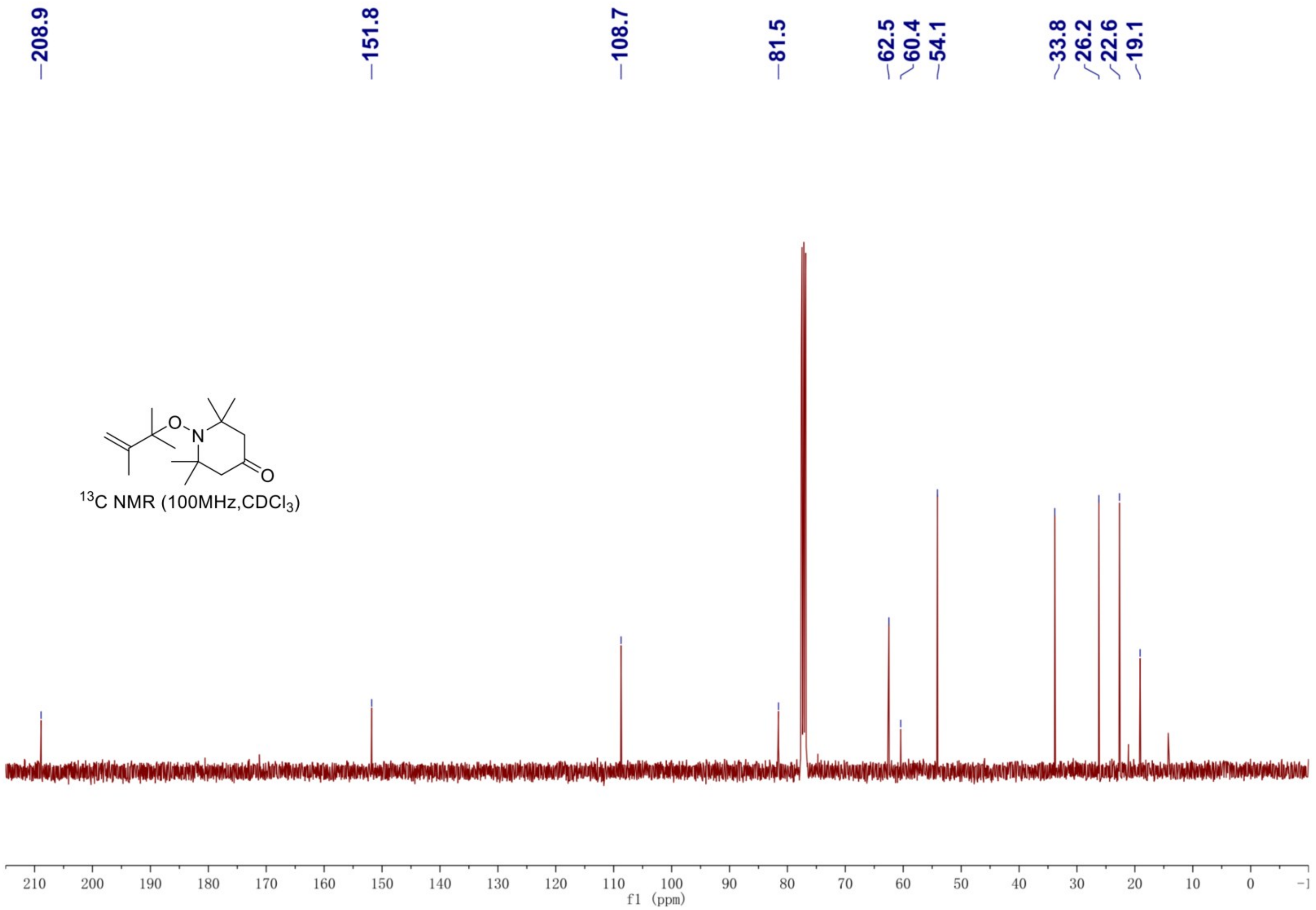

1-(Cyclohex-2-en-1-yloxy)-2,2,6,6-tetramethylpiperidin-4-ol (3h)

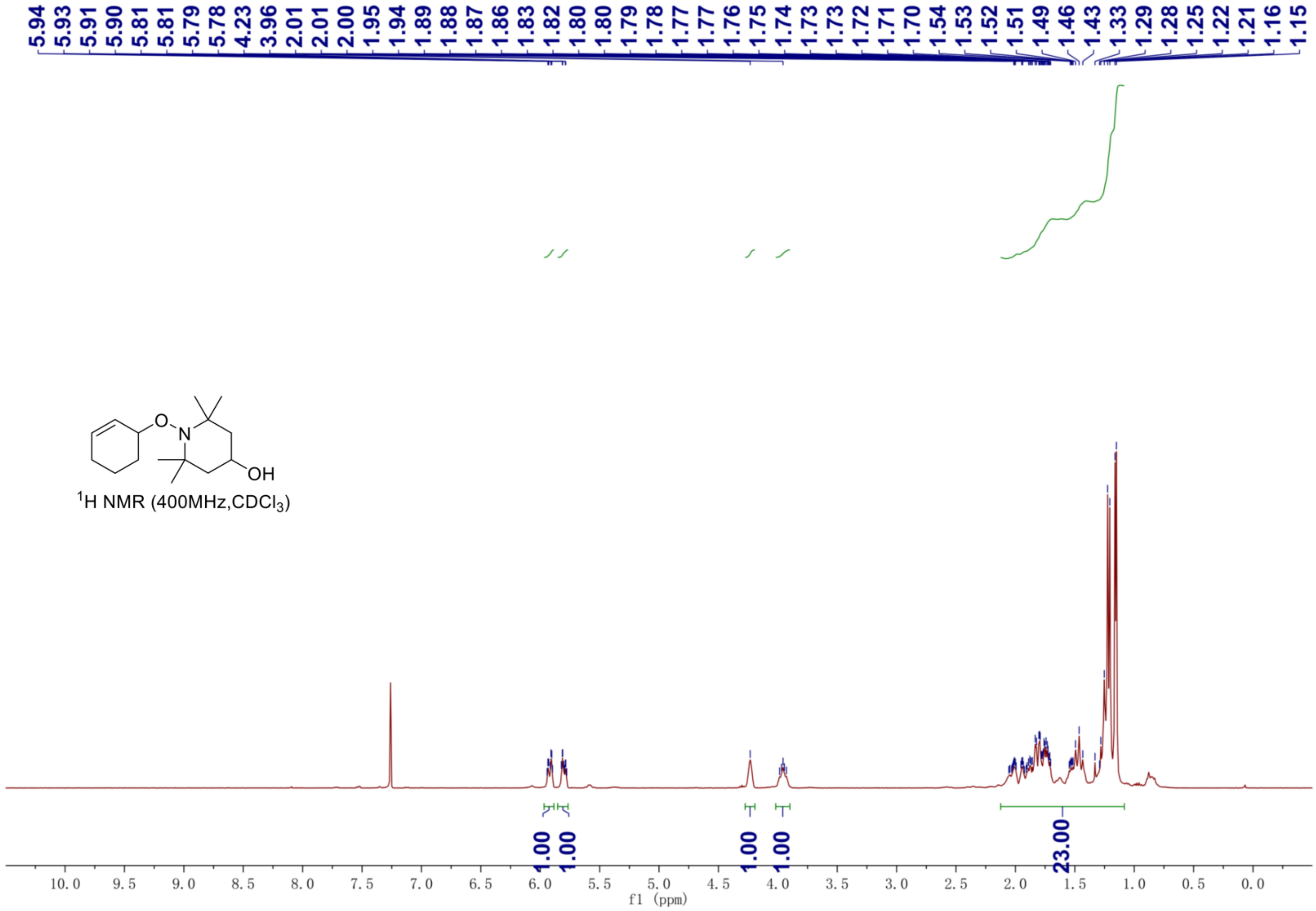

Go to table of contents

1-(Cyclohex-2-en-1-yloxy)-2,2,6,6-tetramethylpiperidin-4-ol (3h)

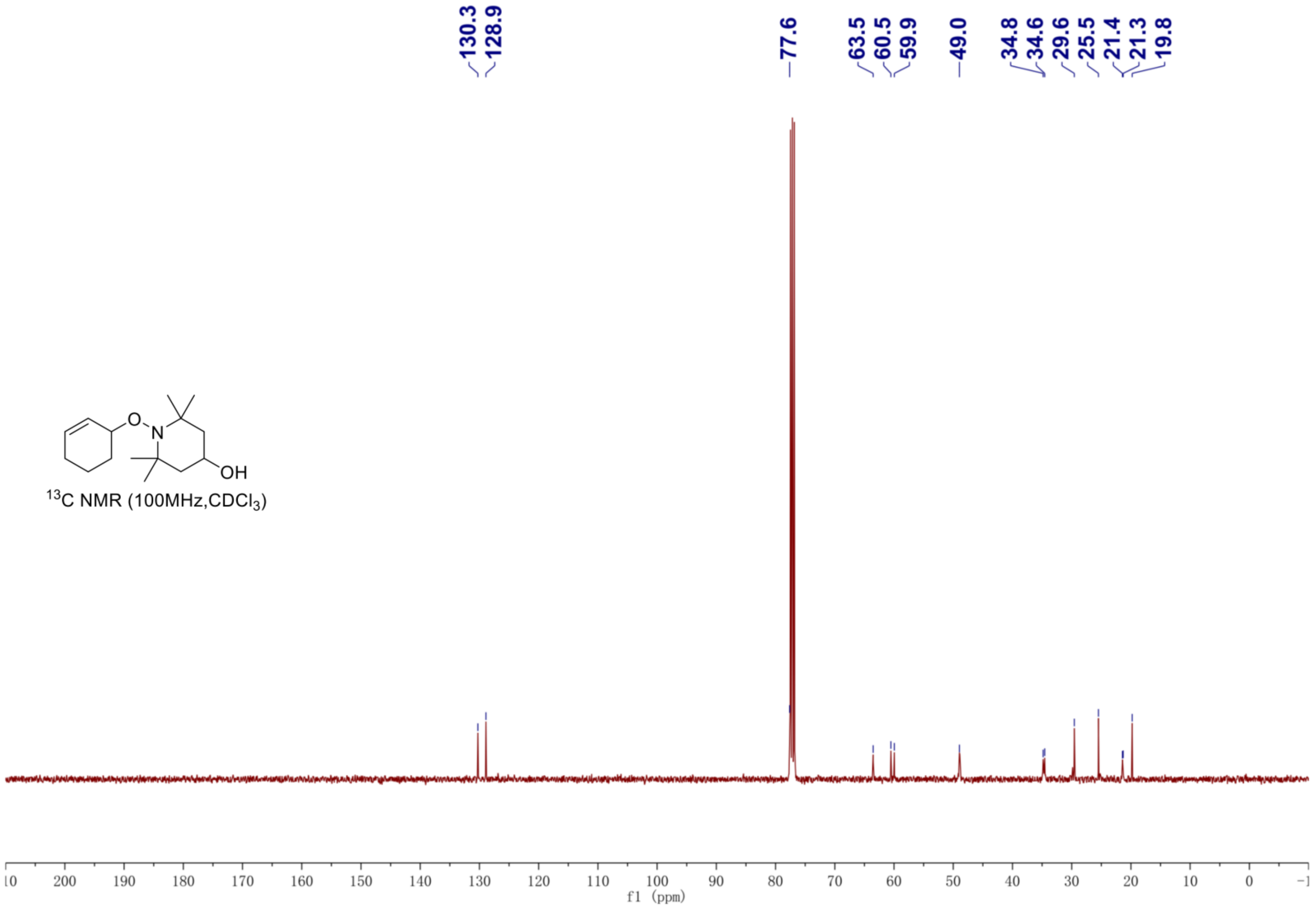

^{13}C NMR (100MHz, $CDCl_3$)

Go to table of contents

1-(Benzyloxy)-2,2,6,6-tetramethylpiperidin-4-ol (3i)

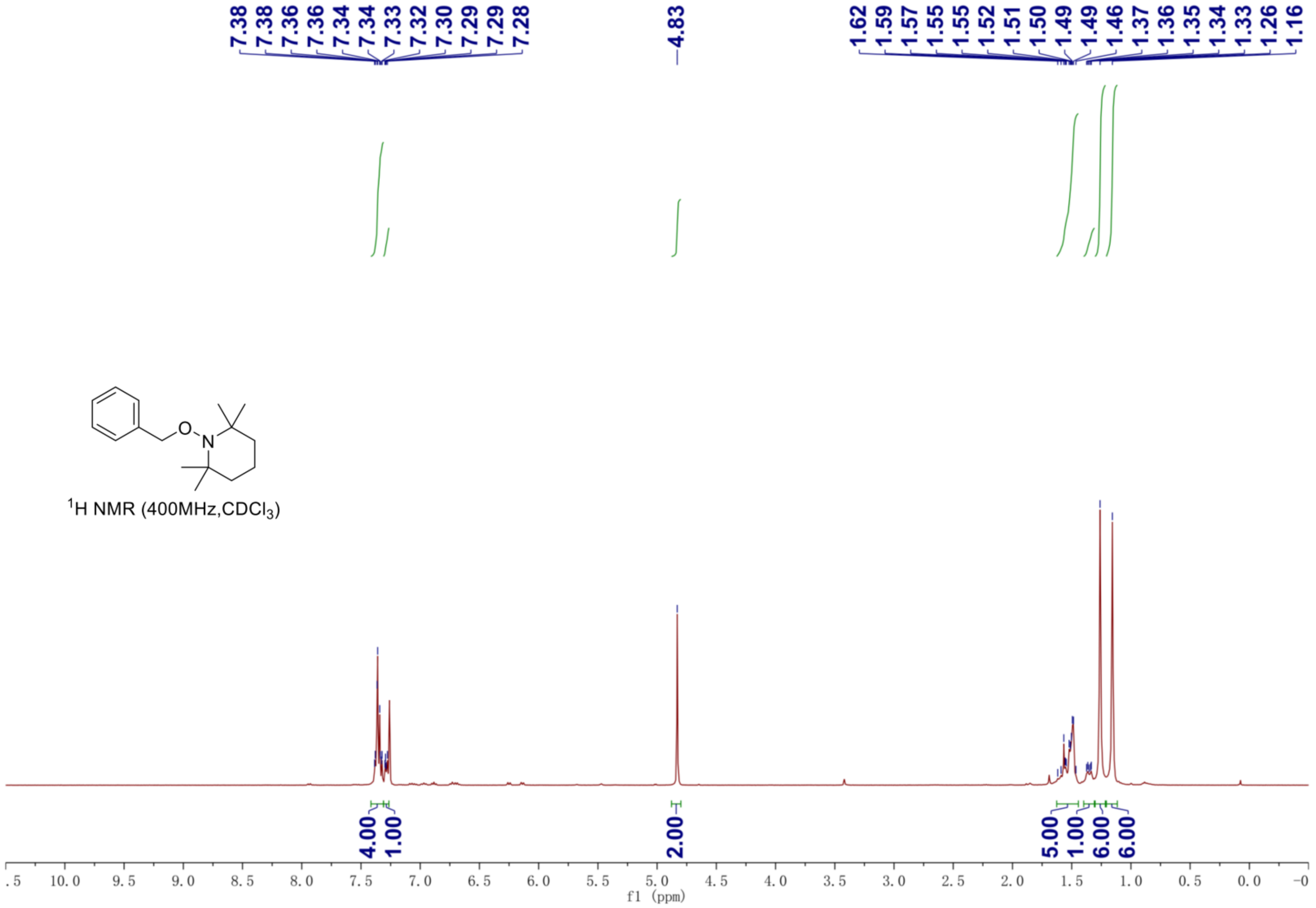

Go to table of contents

1-(Benzyloxy)-2,2,6,6-tetramethylpiperidin-4-ol (3i)

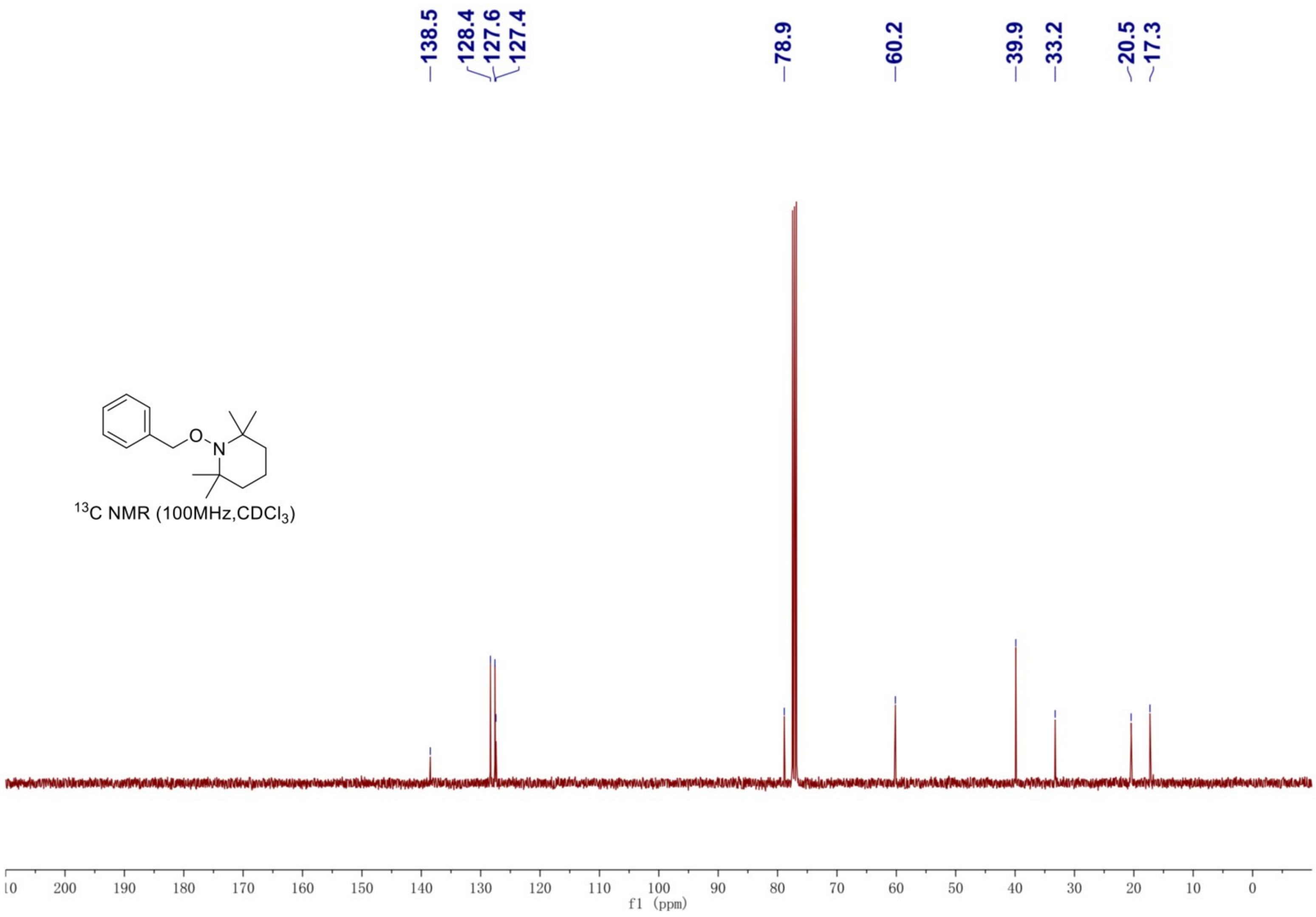

Go to table of contents

2,2,6,6-Tetramethyl-1-(1-phenylethoxy)piperidine (3j-α) and 2,2,6,6-Tetramethyl-1-phenethoxypiperidine (3j-β)

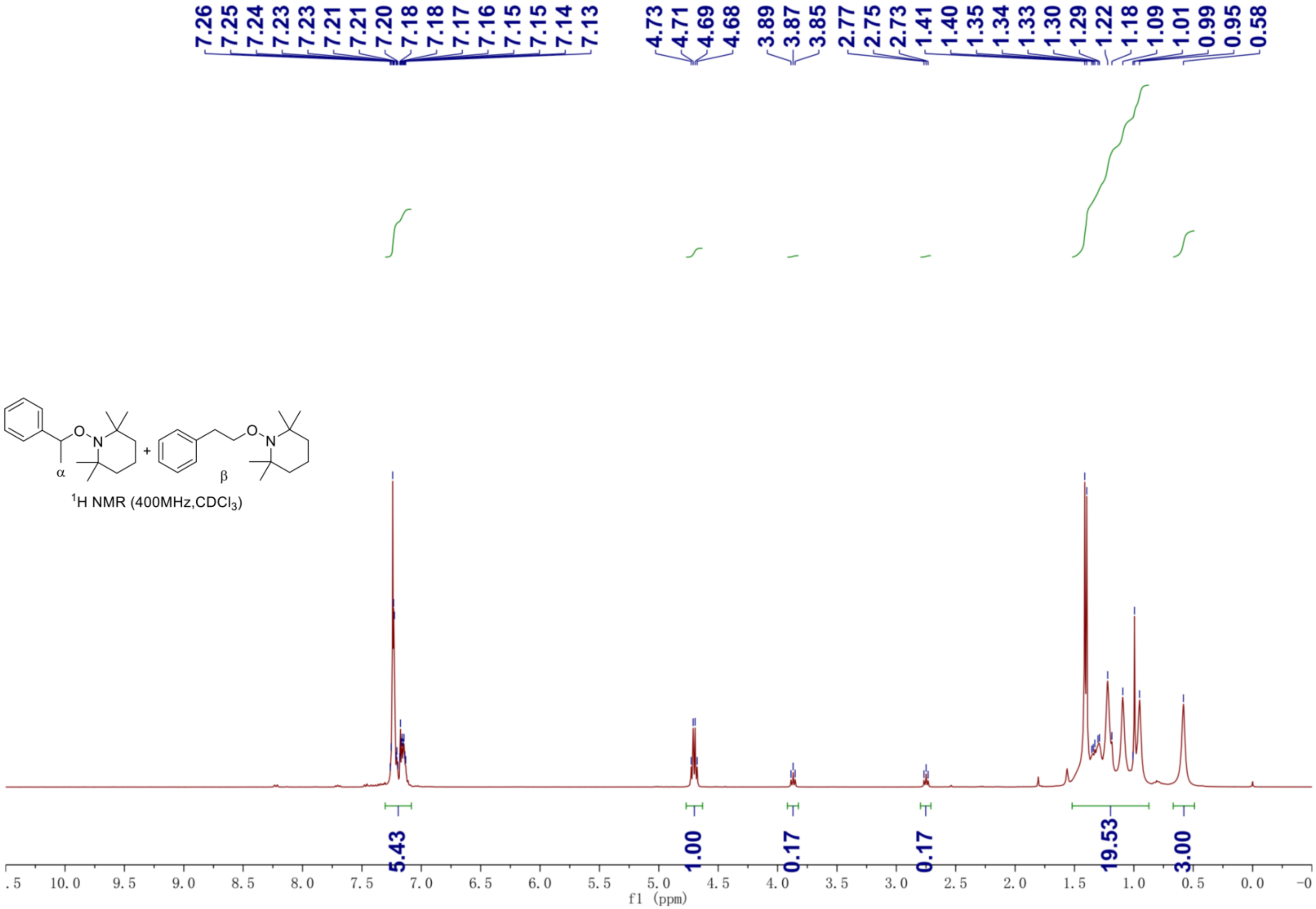

Go to table of contents

2,2,6,6-Tetramethyl-1-(1-phenylethoxy)piperidine (3j-α) and 2,2,6,6-Tetramethyl-1-phenethoxypiperidine (3j-β)

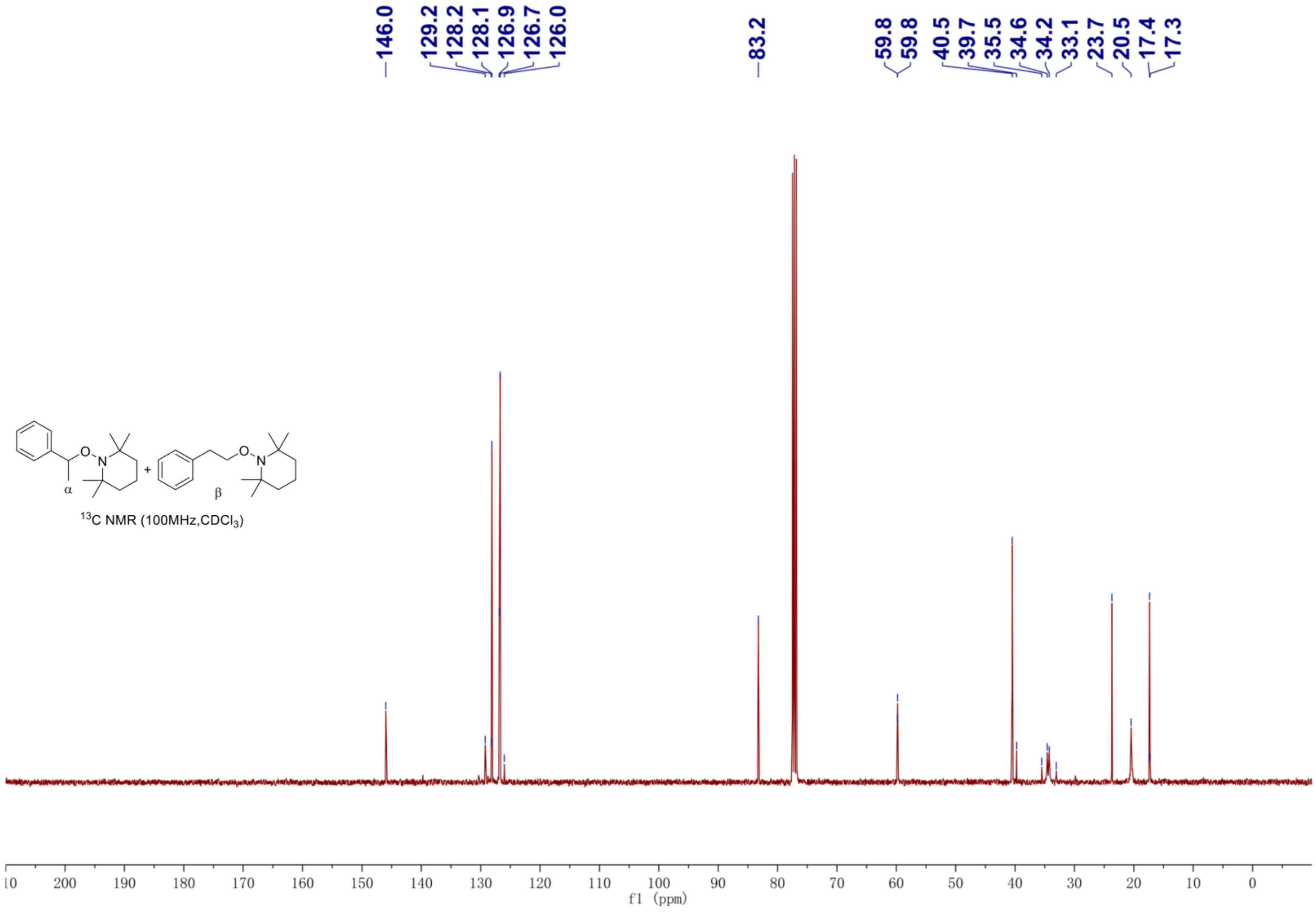

Go to table of contents

2,2,6,6-Tetramethyl-1-((1,2,3,4-tetrahydronaphthalen-1-yl)oxy)piperidin-4-ol (3k-α) and 2,2,6,6-Tetramethyl-1-((1,2,3,4-tetrahydronaphthalen-2-yl)oxy)piperidin-4-ol (3k-β)

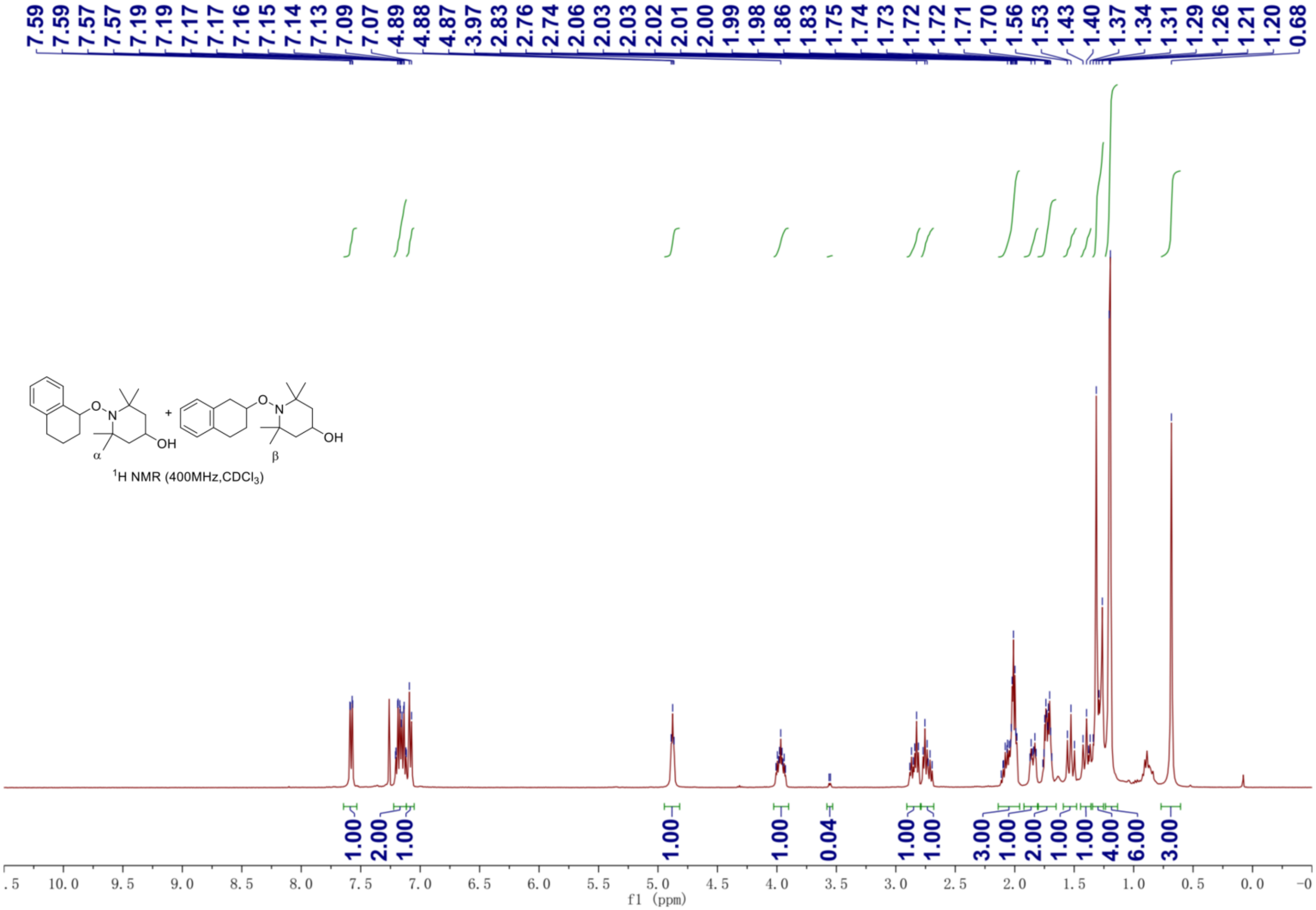

Go to table of contents

2,2,6,6-Tetramethyl-1-((1,2,3,4-tetrahydronaphthalen-1-yl)oxy)piperidin-4-ol (3k-α) and 2,2,6,6-Tetramethyl-1-((1,2,3,4-tetrahydronaphthalen-2-yl)oxy)piperidin-4-ol (3k-β)

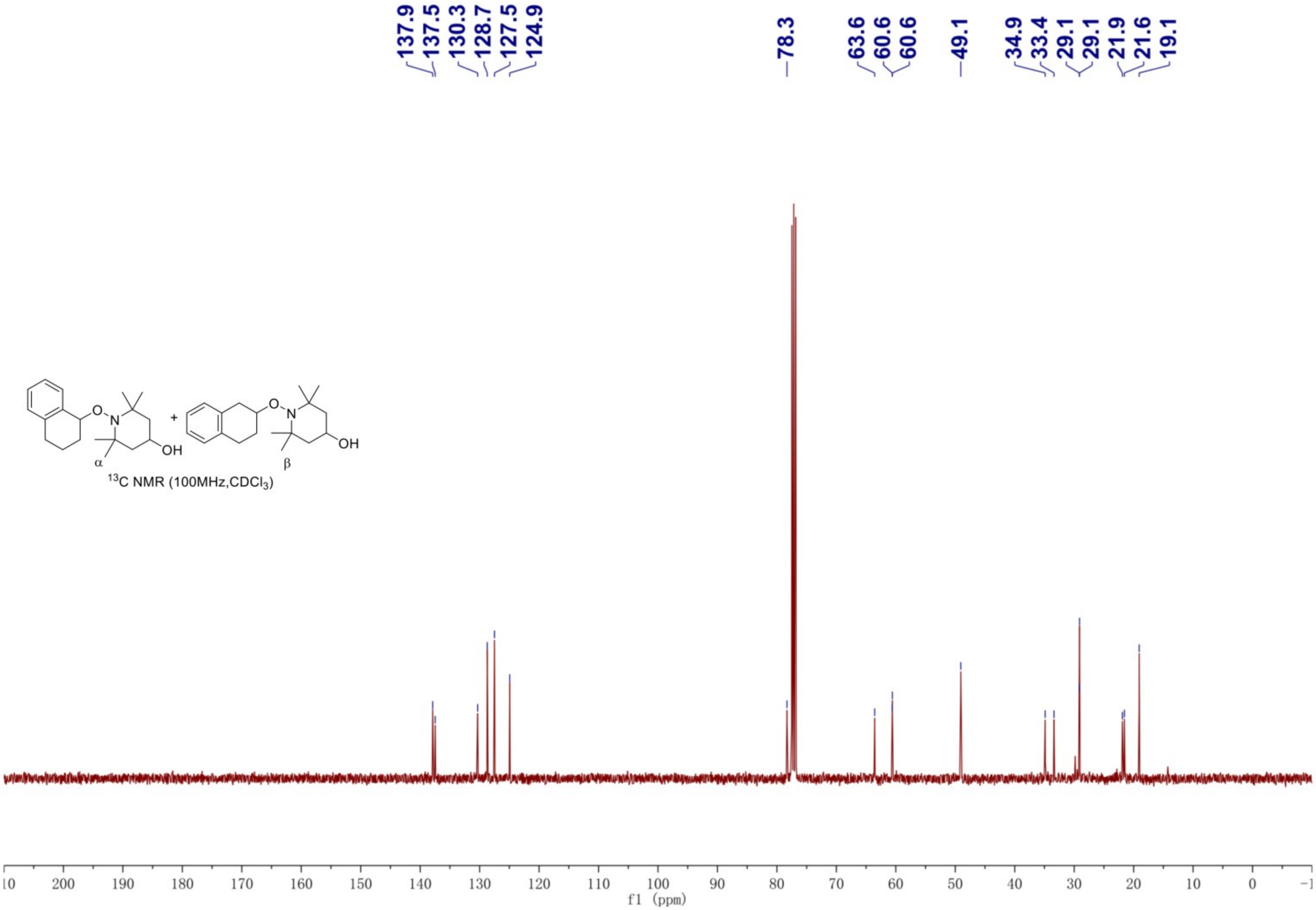

Go to table of contents

2,2,6,6-Tetramethyl-1-((6-methylpyridin-2-yl)methoxy)piperidin-4-ol (3l)

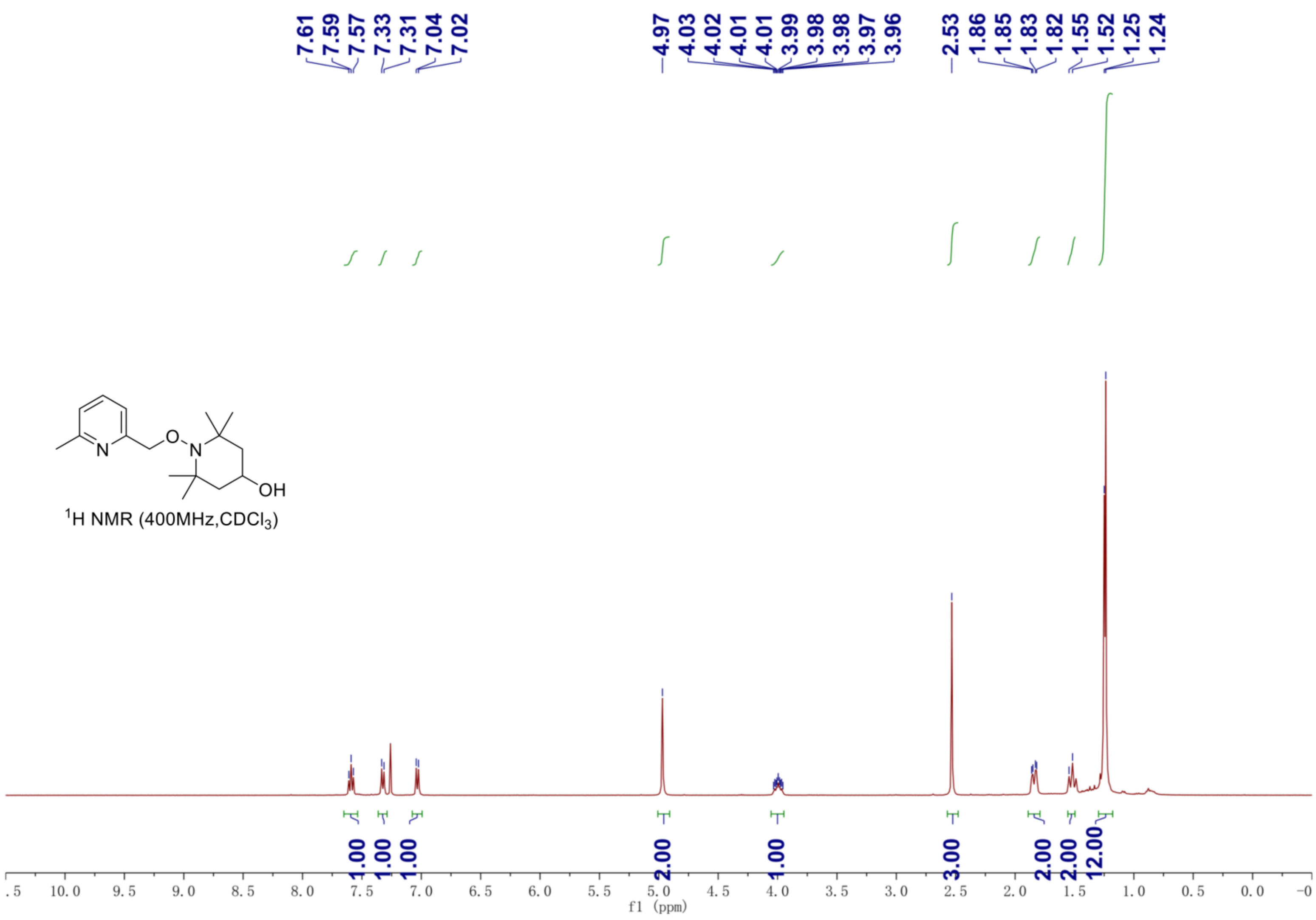

Go to table of contents

2,2,6,6-Tetramethyl-1-((6-methylpyridin-2-yl)methoxy)piperidin-4-ol (3l)

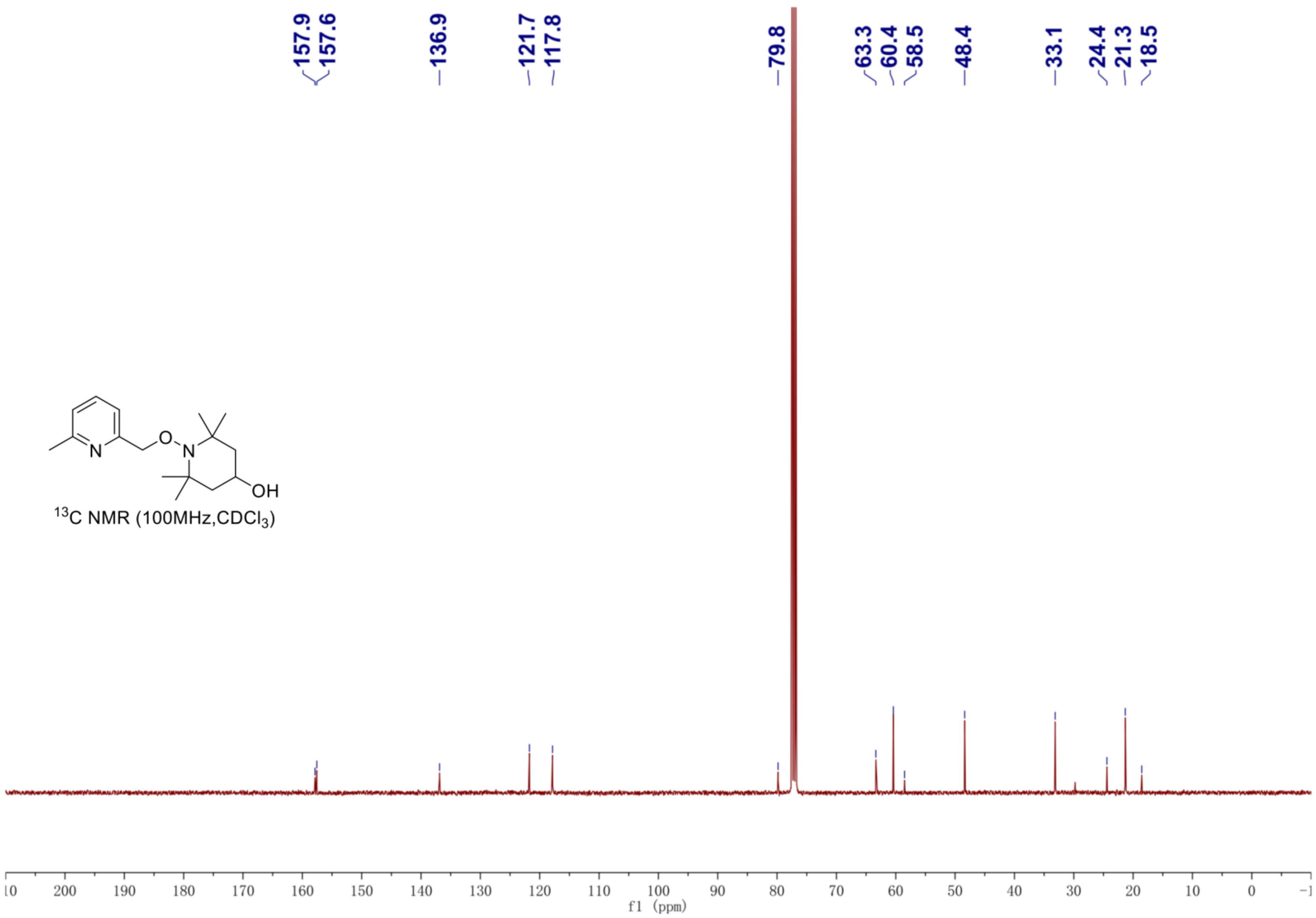

Go to table of contents

2,2,6,6-Tetramethyl-1-(thiophen-2-ylmethoxy)piperidine (3m)

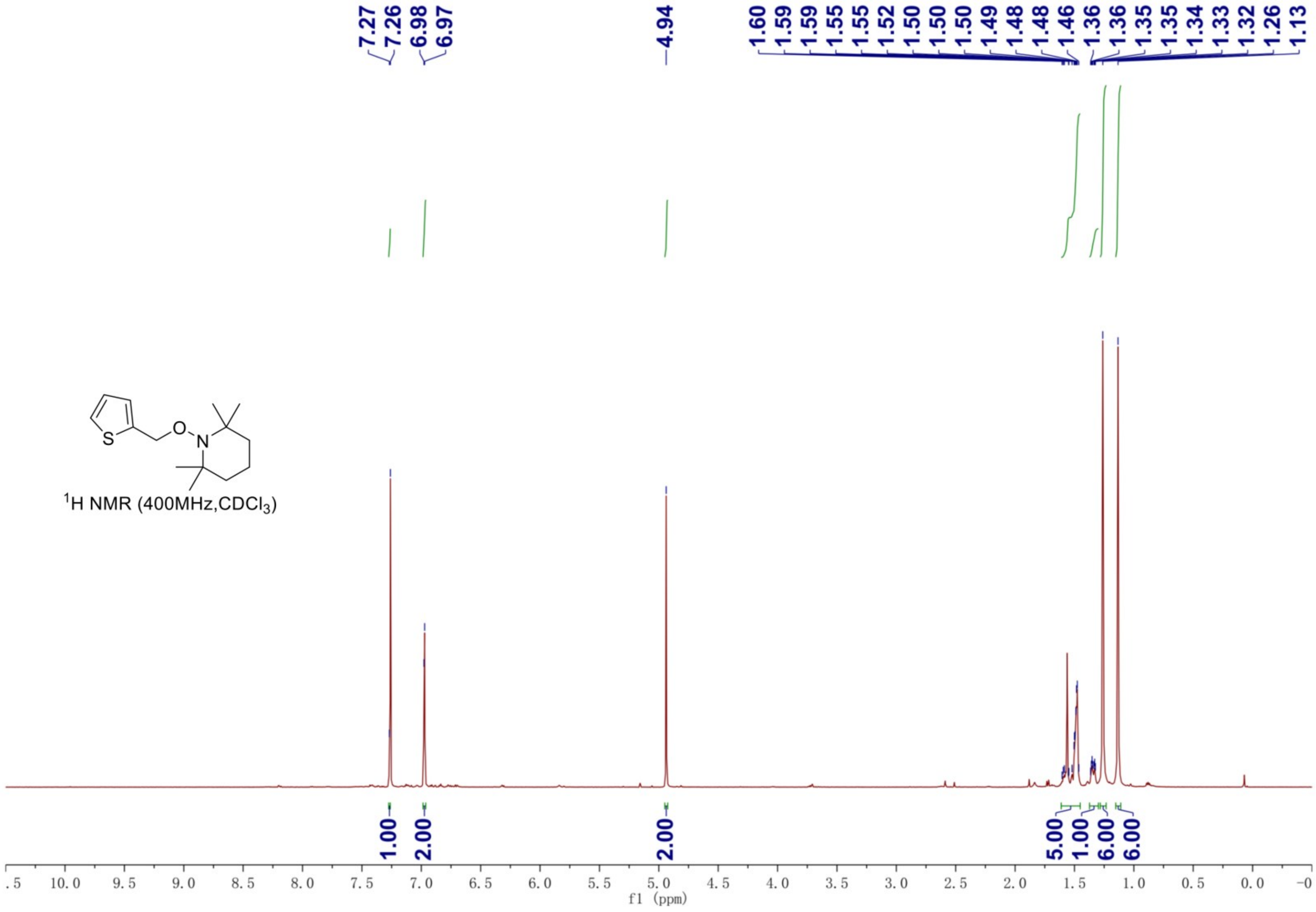

2,2,6,6-Tetramethyl-1-(thiophen-2-ylmethoxy)piperidine (3m)

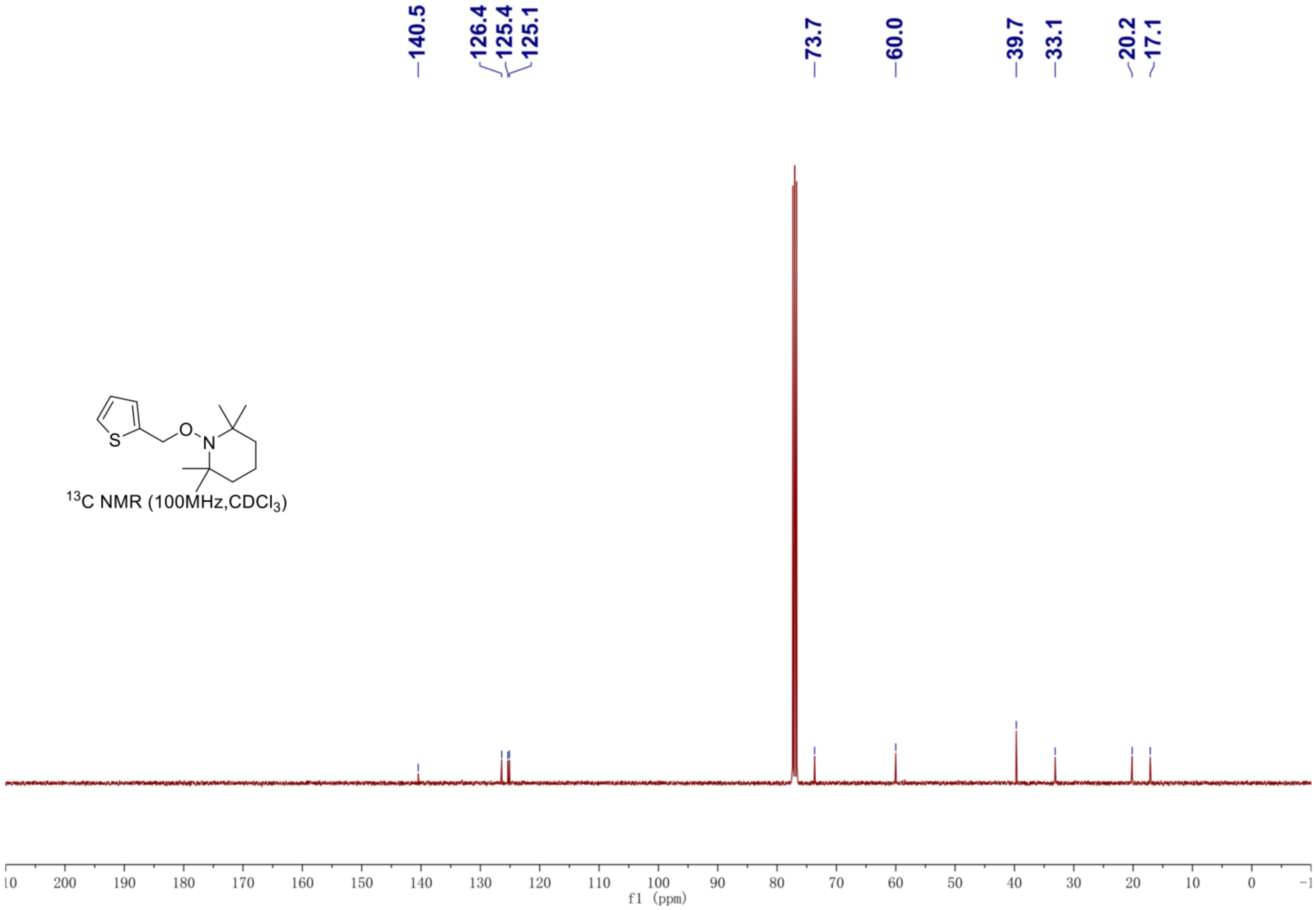

Go to table of contents

1-((1,3-Dihydroisobenzofuran-1-yl)oxy)-2,2,6,6-tetramethylpiperidin-4-ol (3n-α) and 1-((2,3-dihydrobenzofuran-2-yl)oxy)-2,2,6,6-tetramethylpiperidin-4-ol (3n-β)

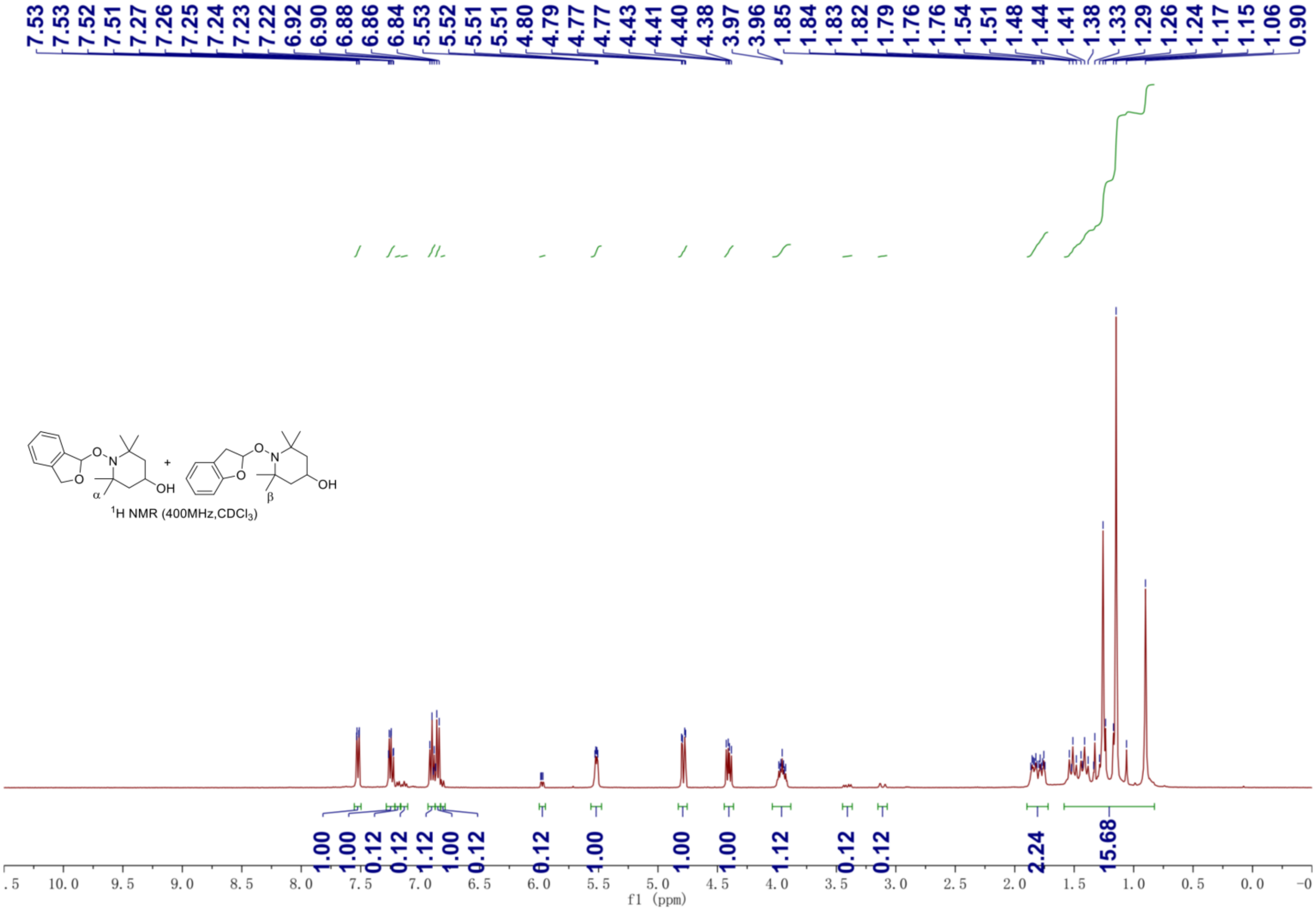

Go to table of contents

1-((1,3-Dihydroisobenzofuran-1-yl)oxy)-2,2,6,6-tetramethylpiperidin-4-ol (3n-α) and 1-((2,3-dihydrobenzofuran-2-yl)oxy)-2,2,6,6-tetramethylpiperidin-4-ol (3n-β)

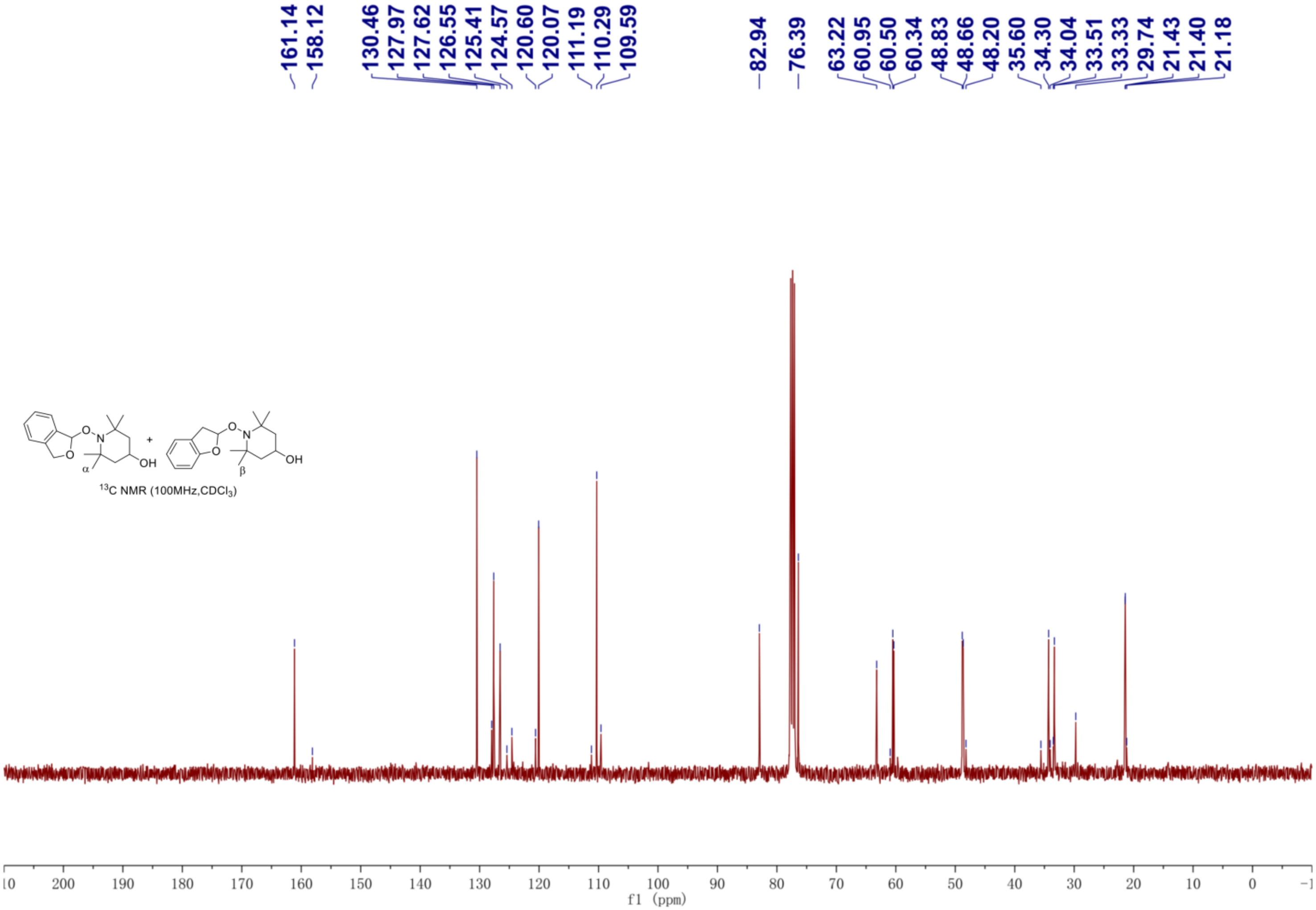

Go to table of contents

1-(*Tert*-Butoxymethoxy)-2,2,6,6-tetramethylpiperidin-4-ol (3o)

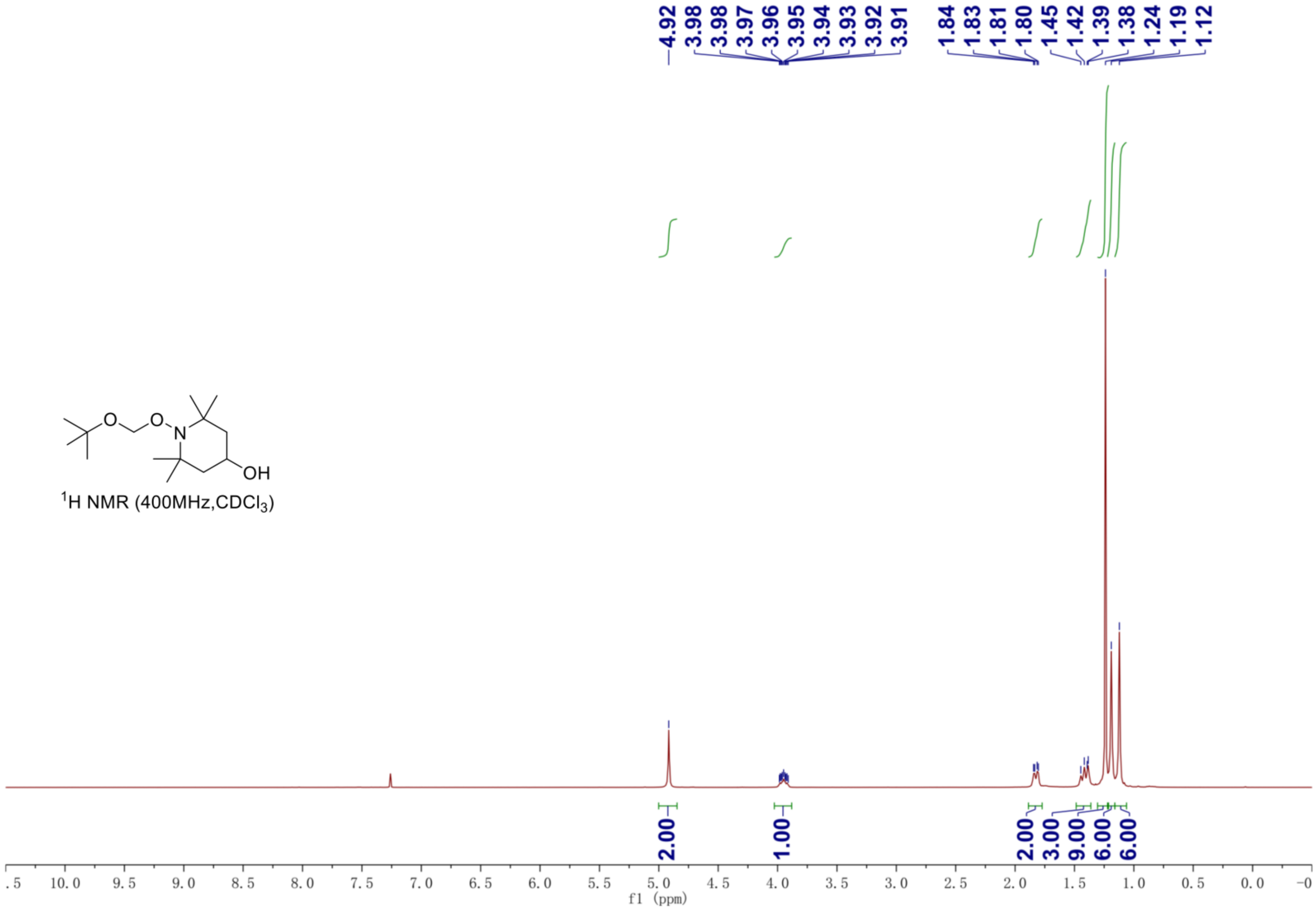

Go to table of contents

1-(*Tert*-Butoxymethoxy)-2,2,6,6-tetramethylpiperidin-4-ol (3o)

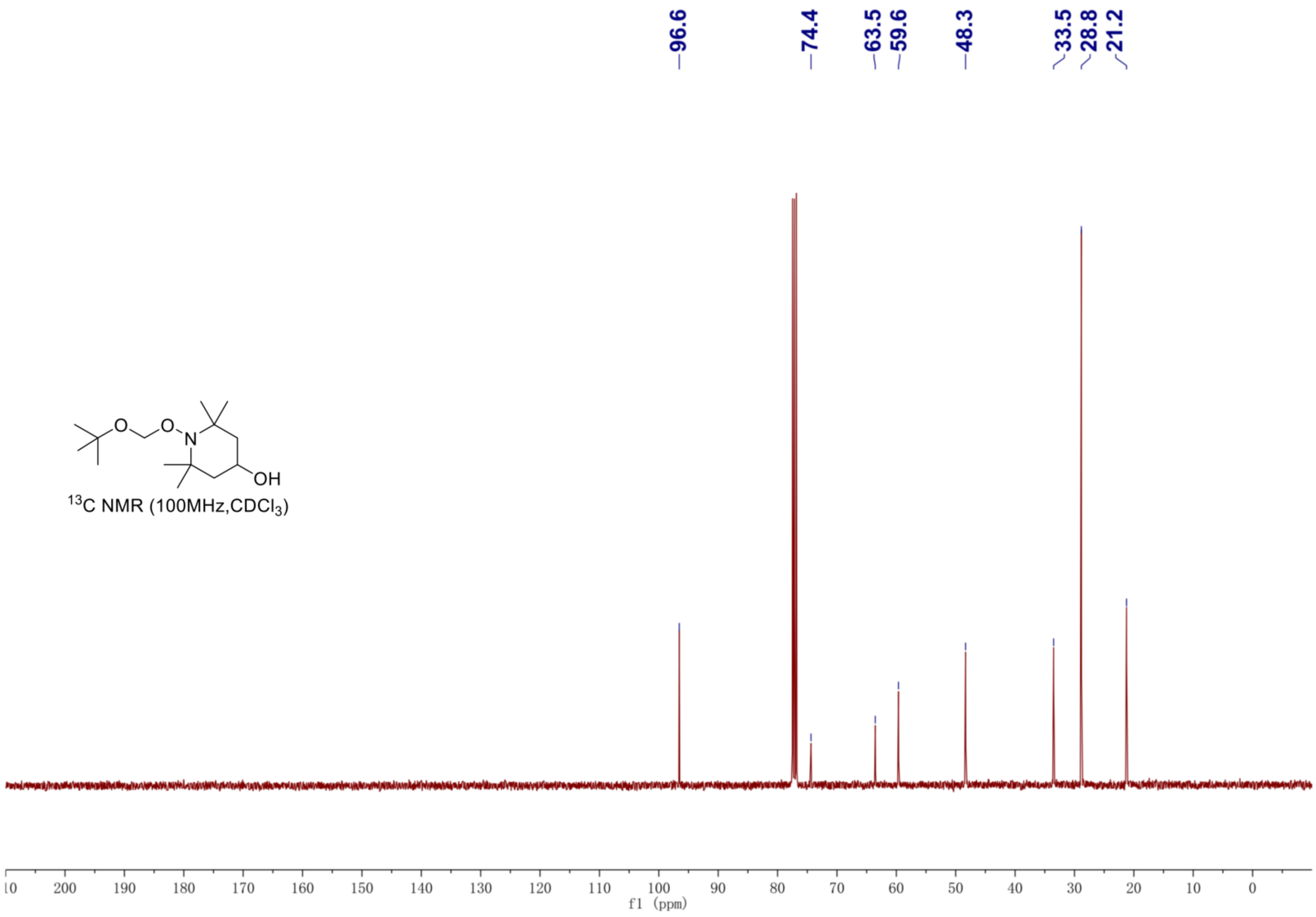

Go to table of contents

1-((1,4-Dioxan-2-yl)oxy)-2,2,6,6-tetramethylpiperidin-4-ol (3p)

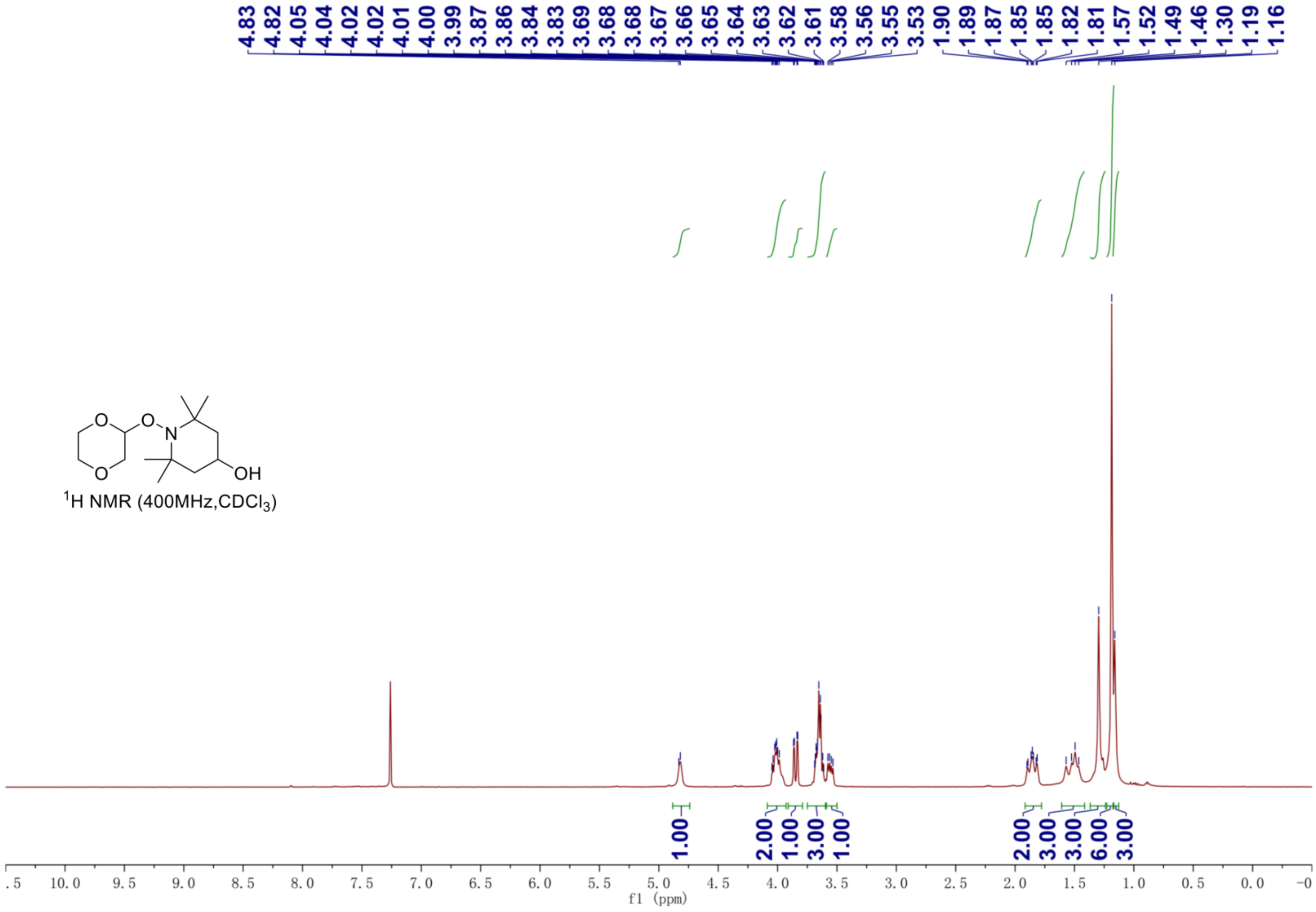

Go to table of contents

1-((1,4-Dioxan-2-yl)oxy)-2,2,6,6-tetramethylpiperidin-4-ol (3p)

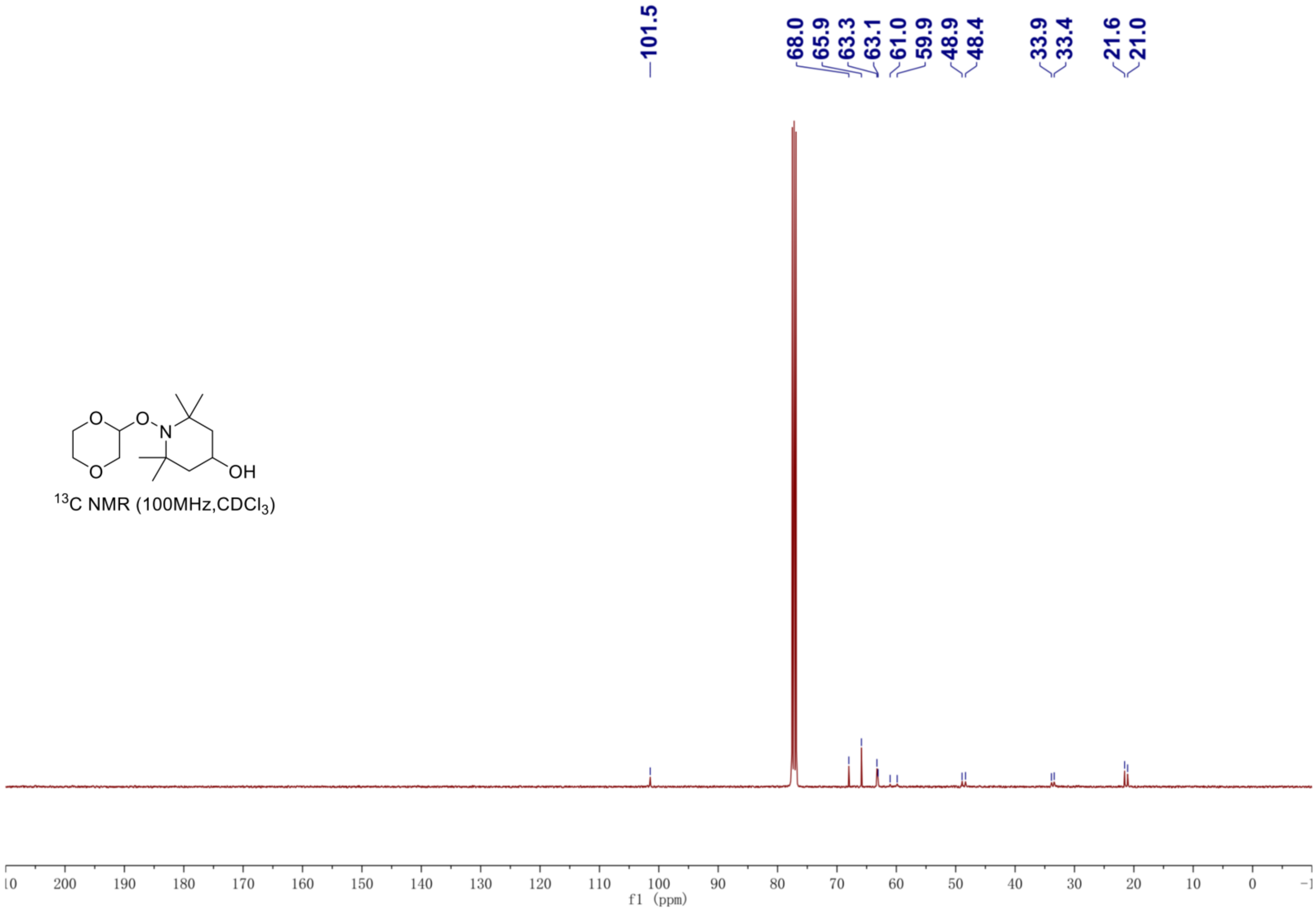

^{13}C NMR (100MHz, $CDCl_3$)

Go to table of contents

2,2,6,6-Tetramethyl-1-((tetrahydrofuran-2-yl)oxy)piperidin-4-one (3q)

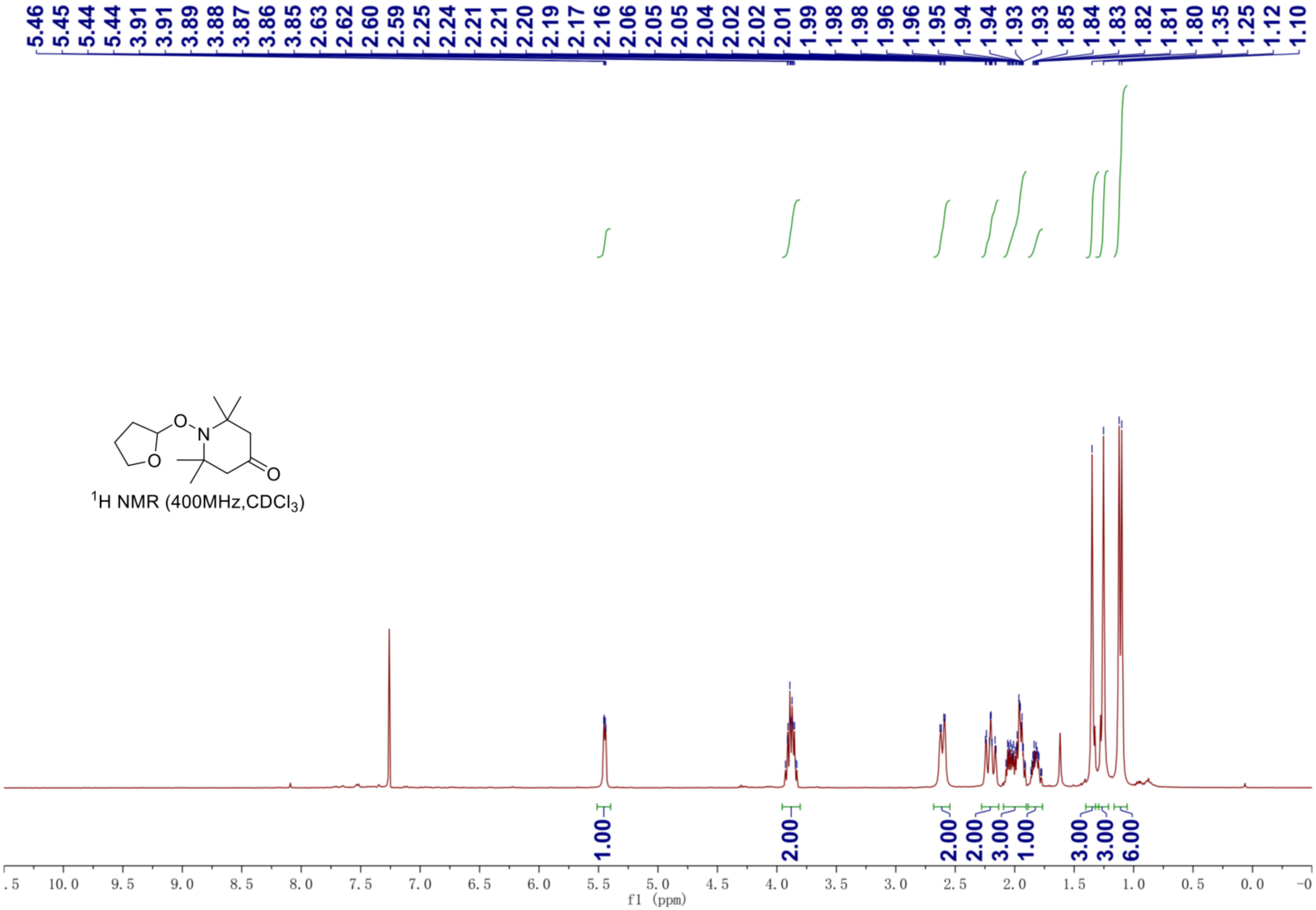

Go to table of contents

2,2,6,6-Tetramethyl-1-((tetrahydrofuran-2-yl)oxy)piperidin-4-one (3q)

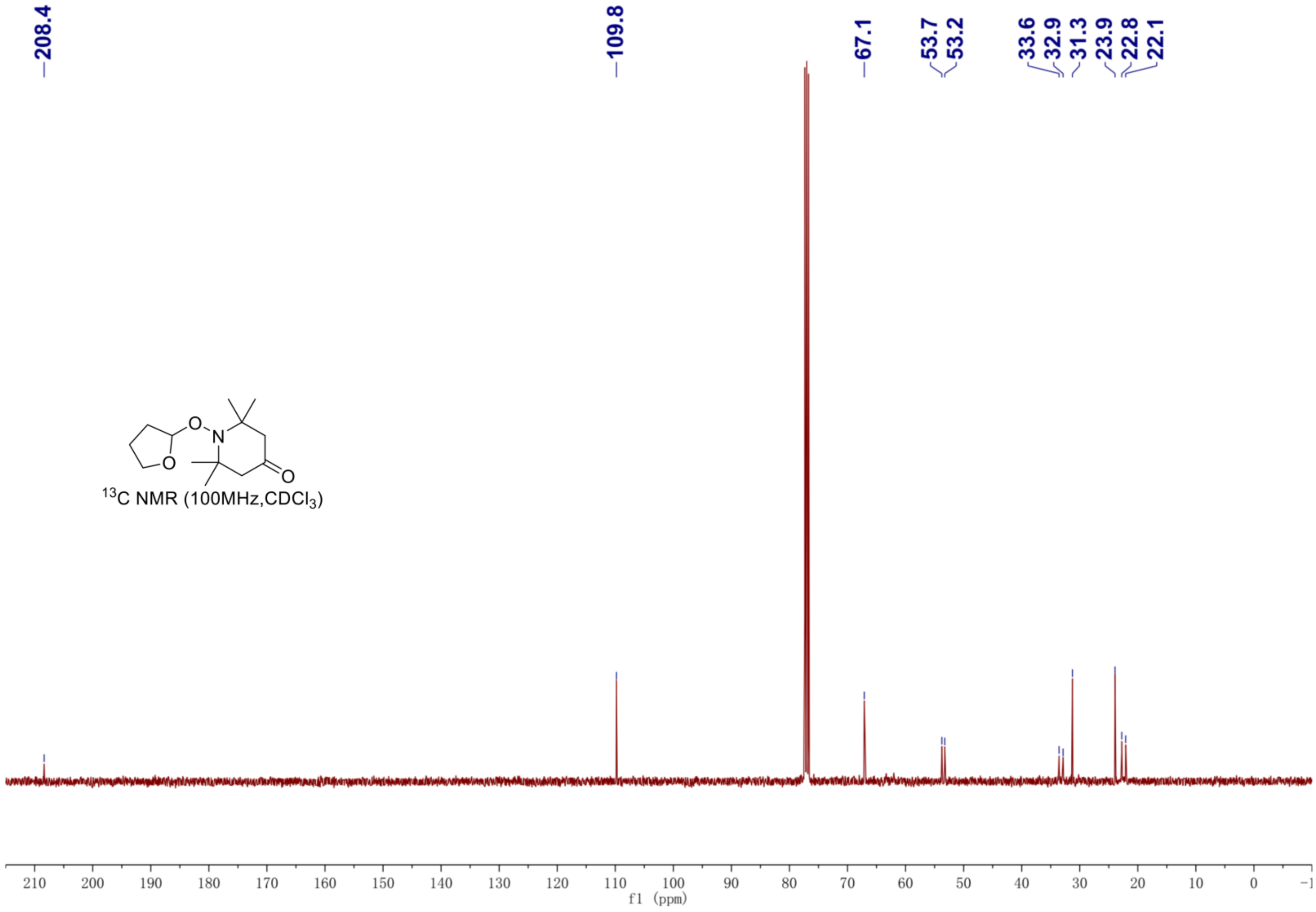

Go to table of contents

2,2,6,6-Tetramethyl-1-((tetrahydrothiophen-2-yl)oxy)piperidin-4-one (3r)

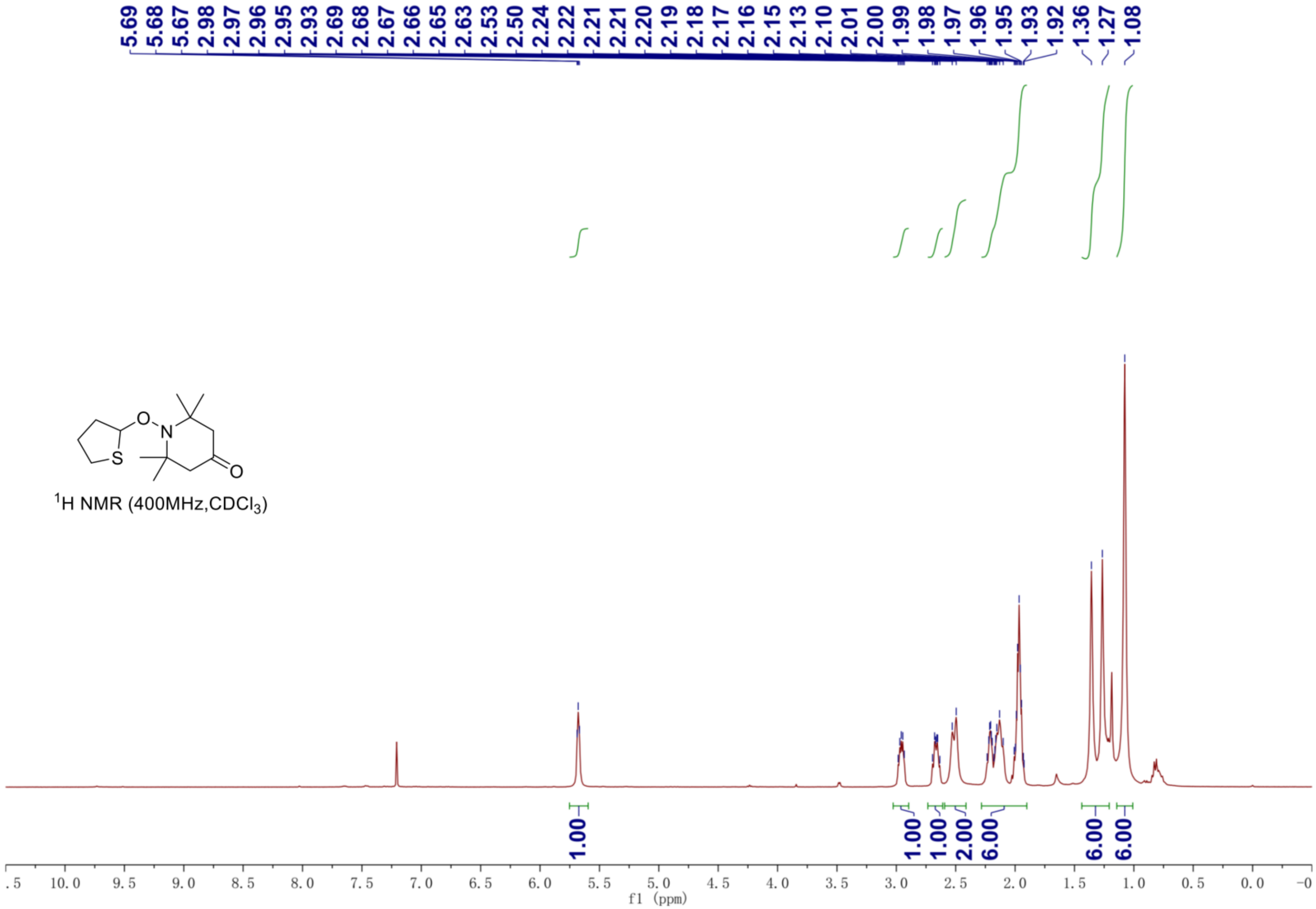

Go to table of contents

2,2,6,6-Tetramethyl-1-((tetrahydrothiophen-2-yl)oxy)piperidin-4-one (3r)

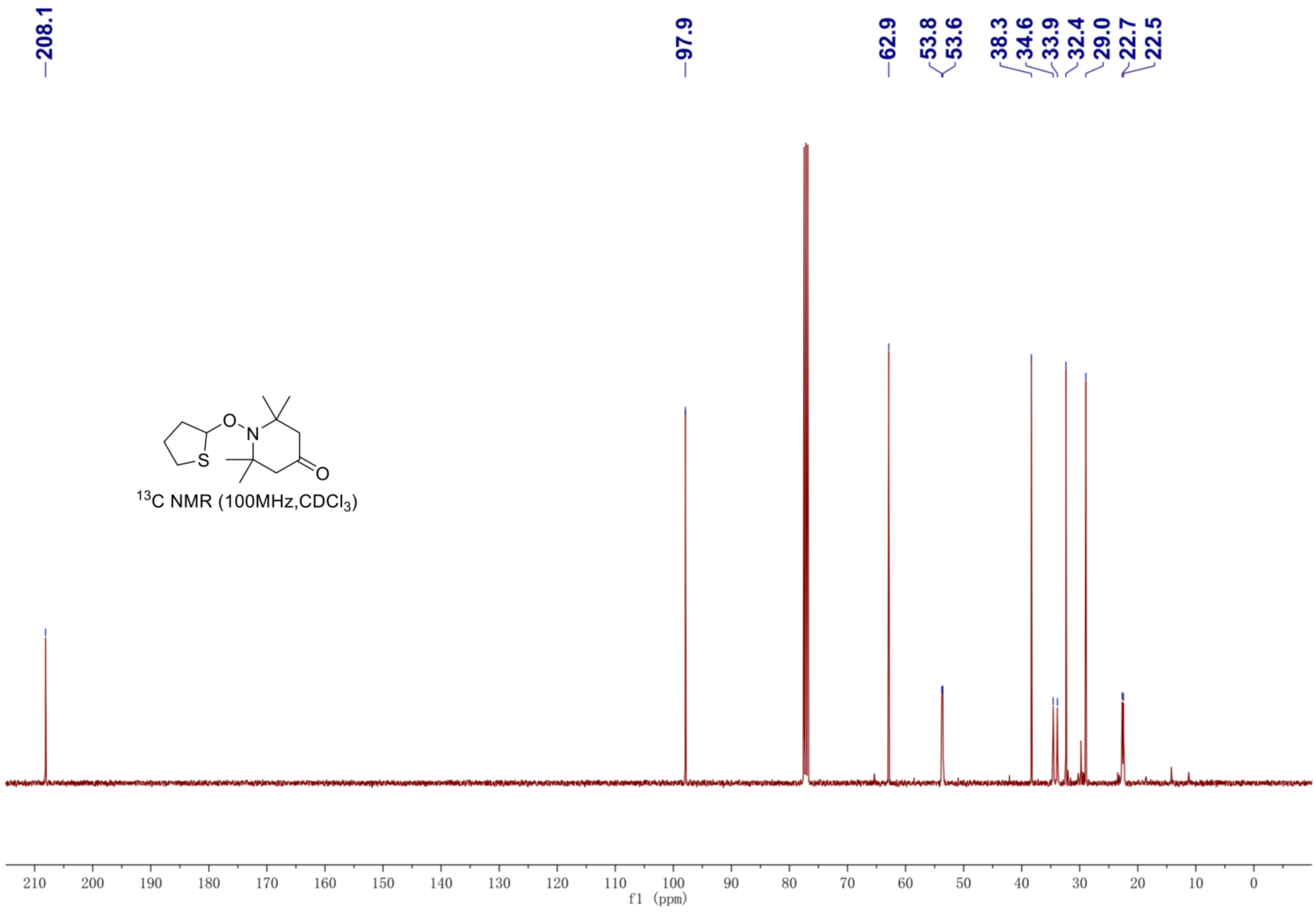

Go to table of contents

1-((2,2,6,6-Tetramethyl-4-oxopiperidin-1-yl)oxy)ethyl acetate (3s-α) and 2-((2,2,6,6-Tetramethyl-4-oxopiperidin-1-yl)oxy)ethyl acetate (3s-β)

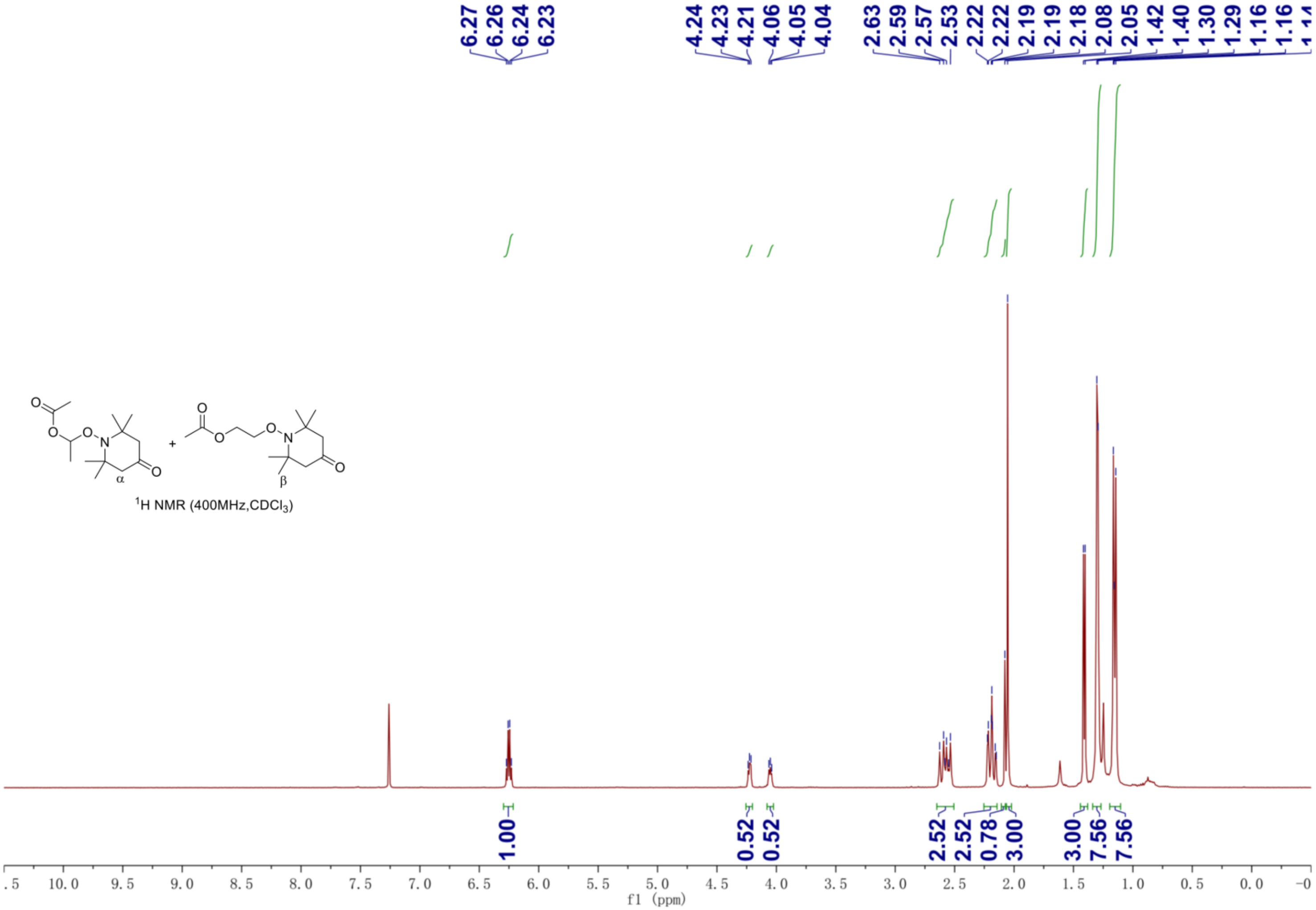

Go to table of contents

1-((2,2,6,6-Tetramethyl-4-oxopiperidin-1-yl)oxy)ethyl acetate (3s-α) and 2-((2,2,6,6-Tetramethyl-4-oxopiperidin-1-yl)oxy)ethyl acetate (3s-β)

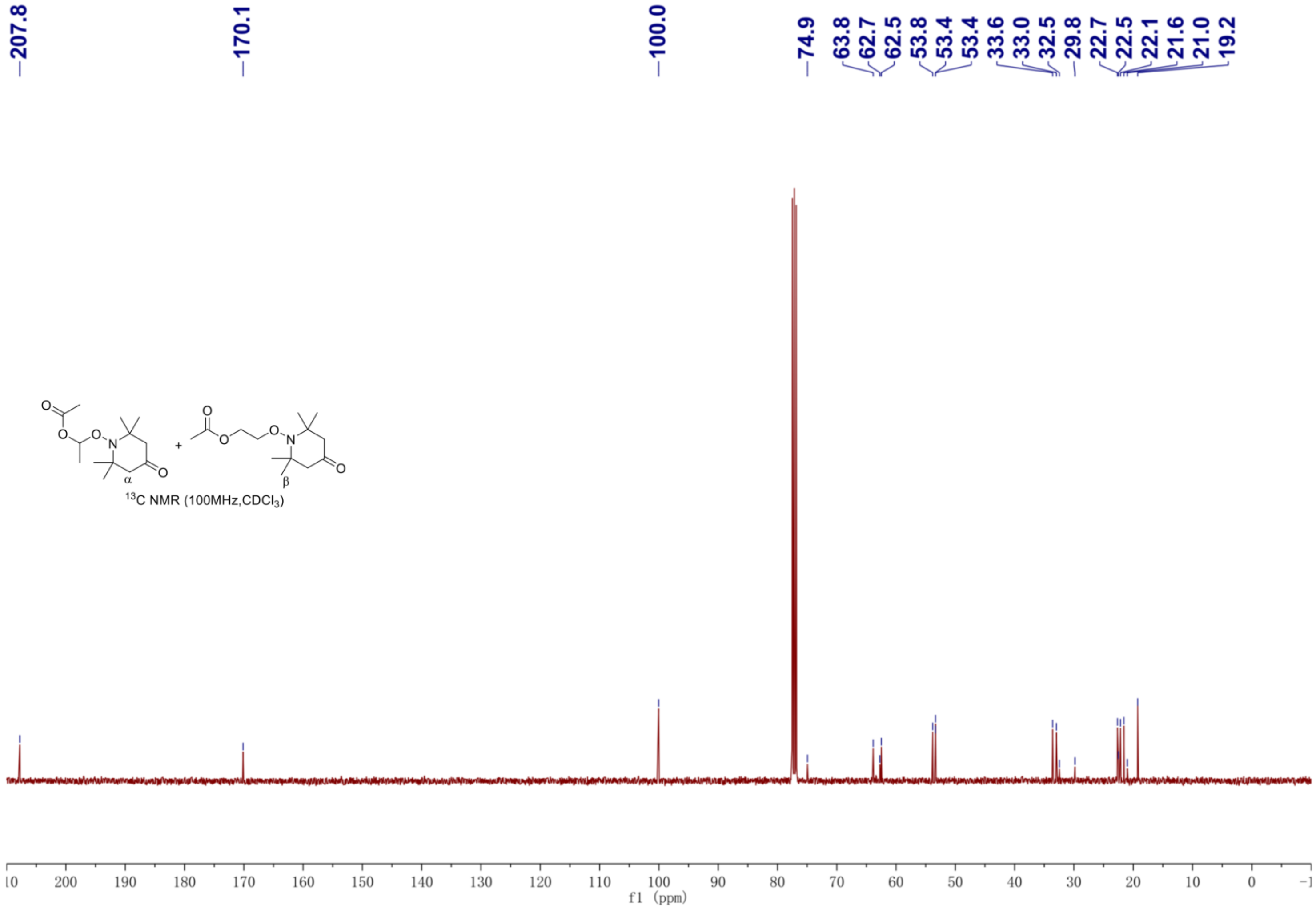

Go to table of contents

1-(1-Ethoxyethoxy)-2,2,6,6-tetramethylpiperidin-4-ol (3t)

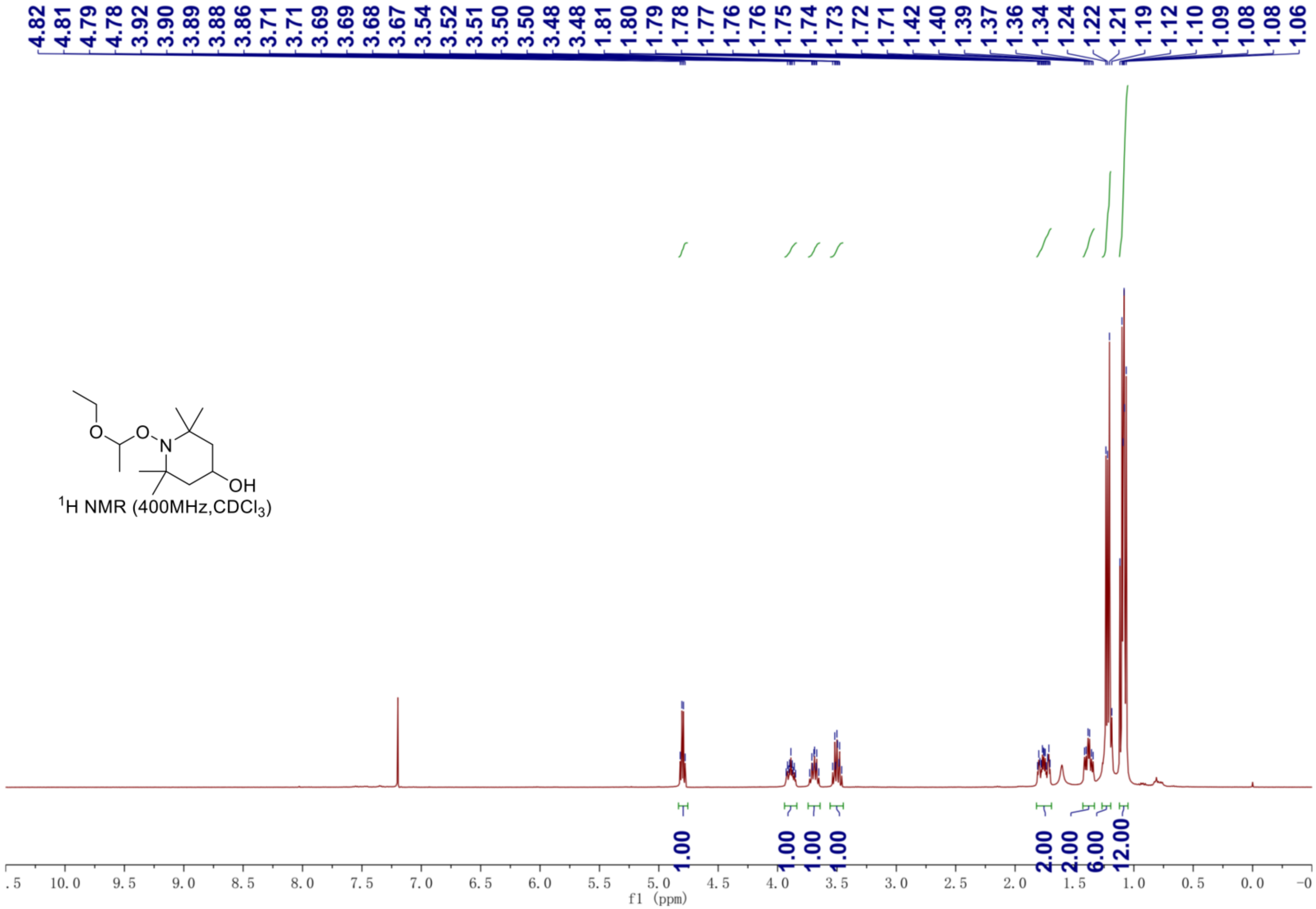

Go to table of contents

1-(1-Ethoxyethoxy)-2,2,6,6-tetramethylpiperidin-4-ol (3t)

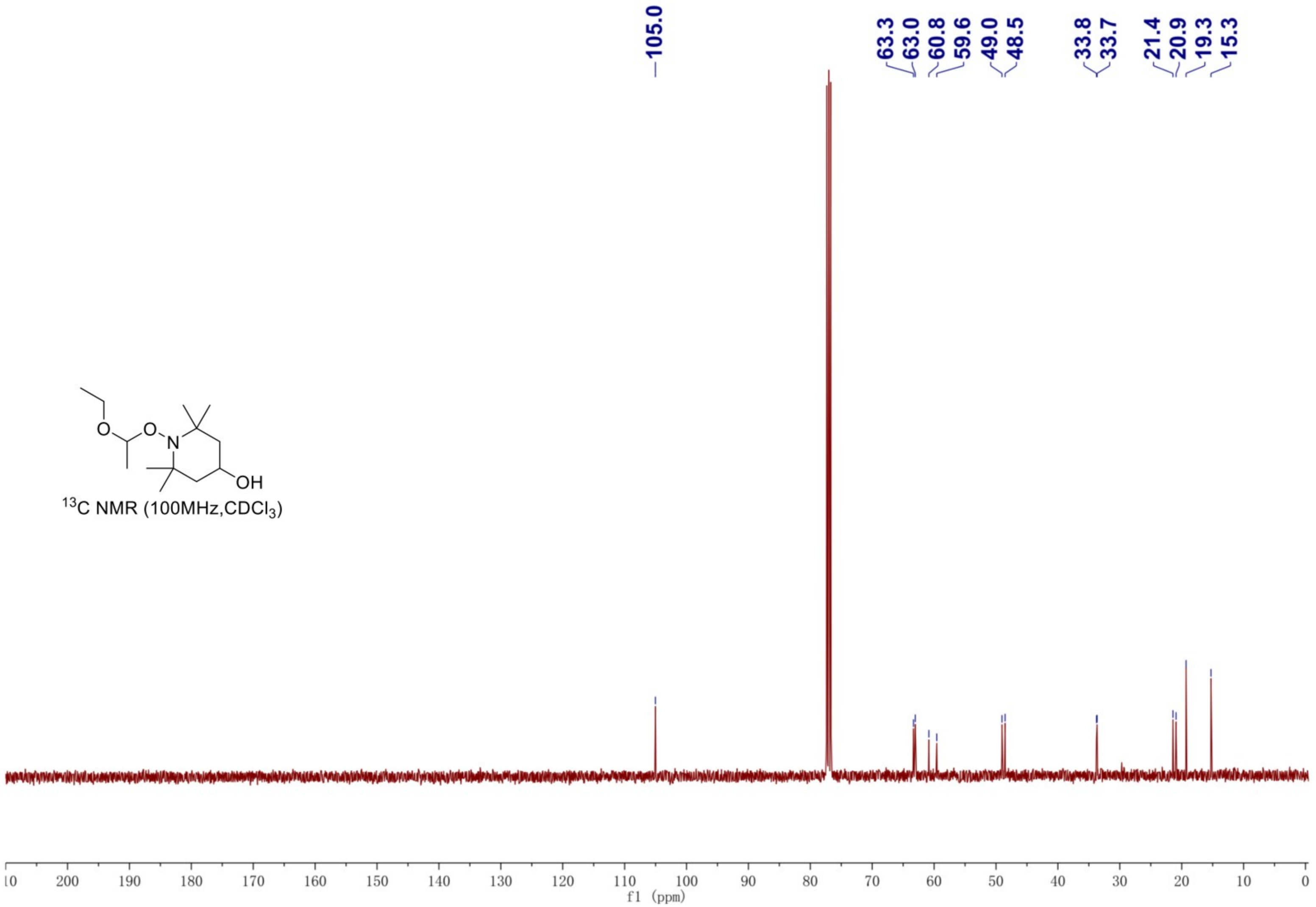

Go to table of contents

2,2,6,6-Tetramethyl-1-((phenylthio)methoxy)piperidin-4-one (3u)

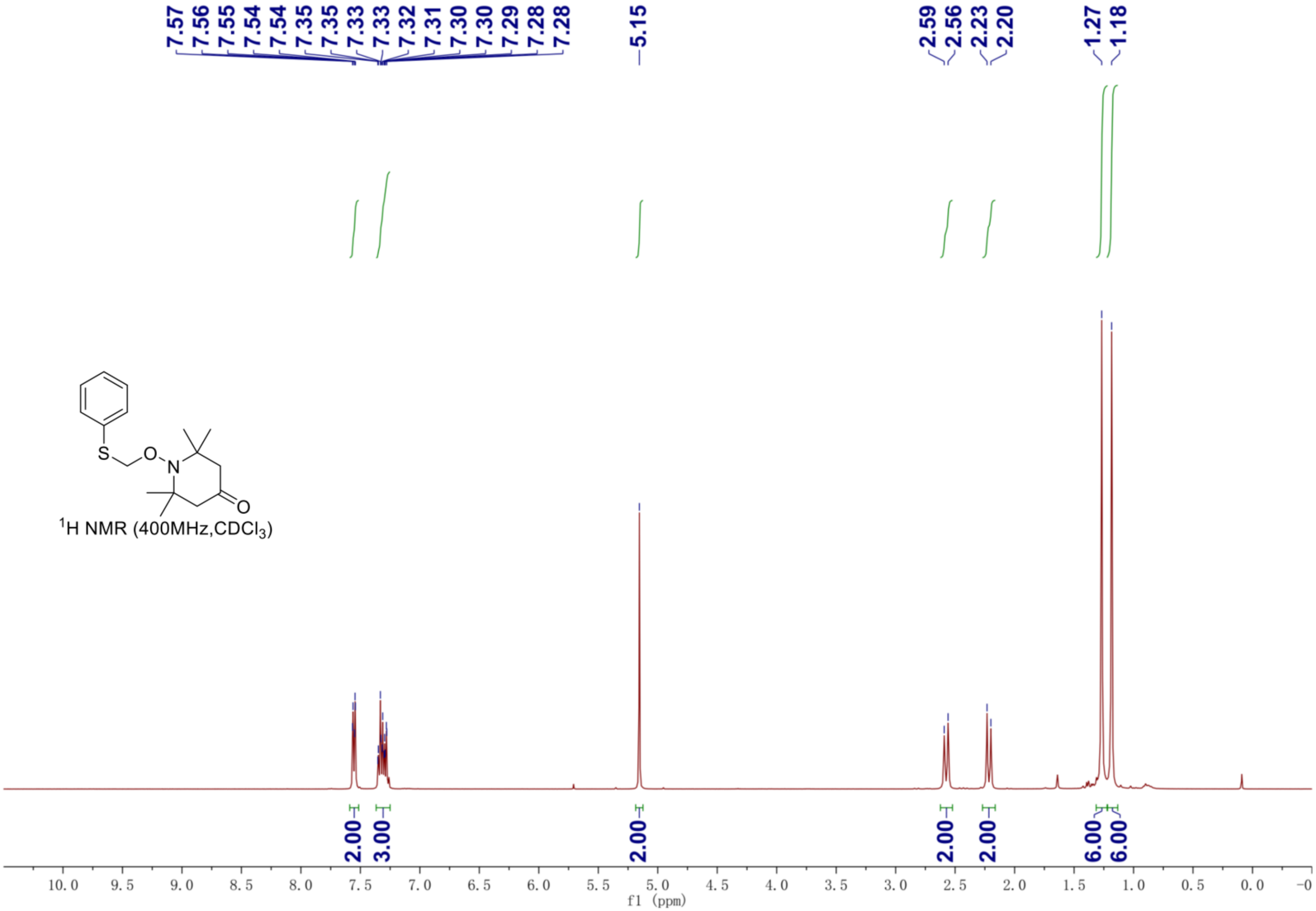

Go to table of contents

2,2,6,6-Tetramethyl-1-((phenylthio)methoxy)piperidin-4-one (3u)

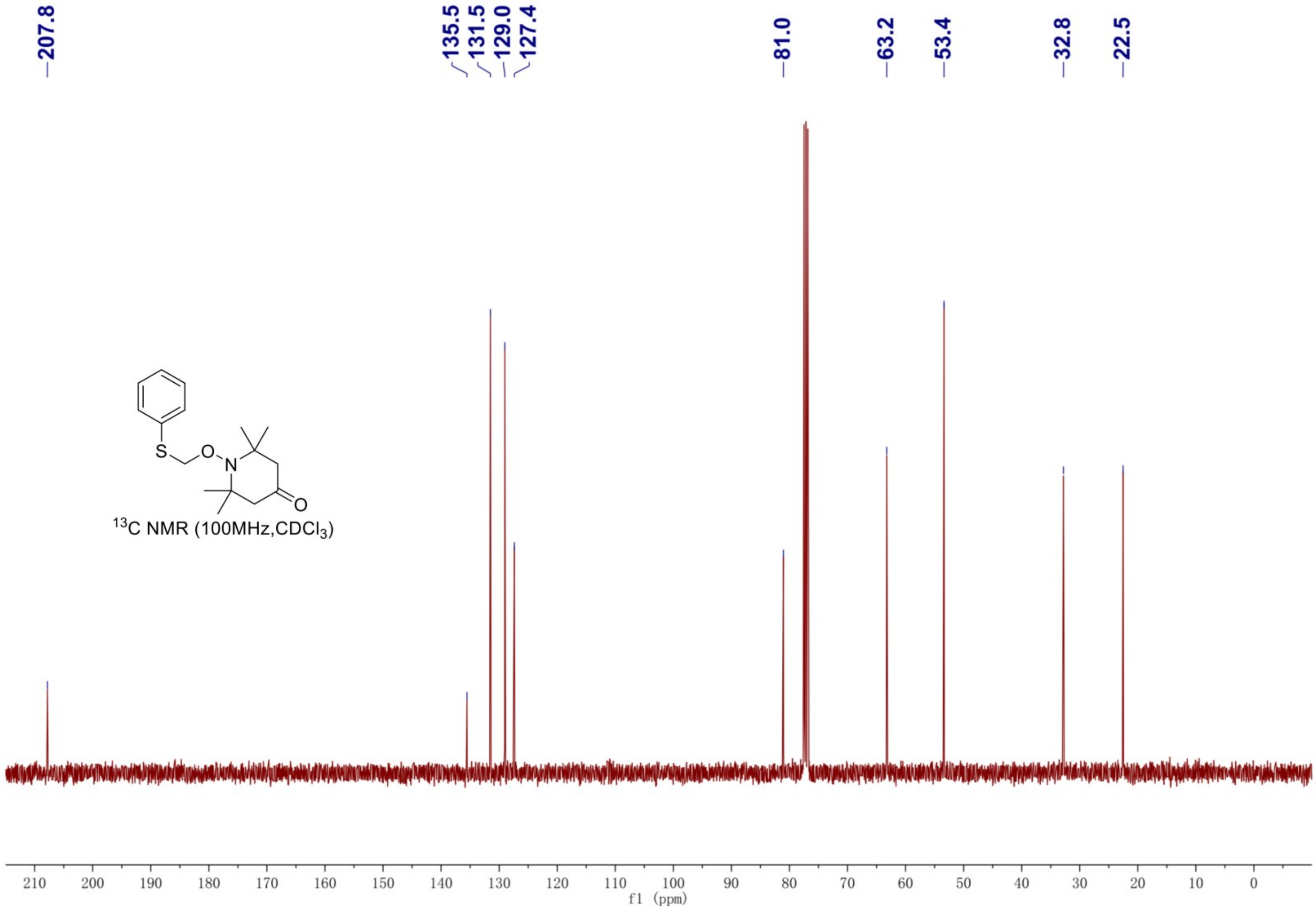

Go to table of contents

1-(((4-Fluorophenyl)thio)methoxy)-2,2,6,6-tetramethylpiperidin-4-one (3v)

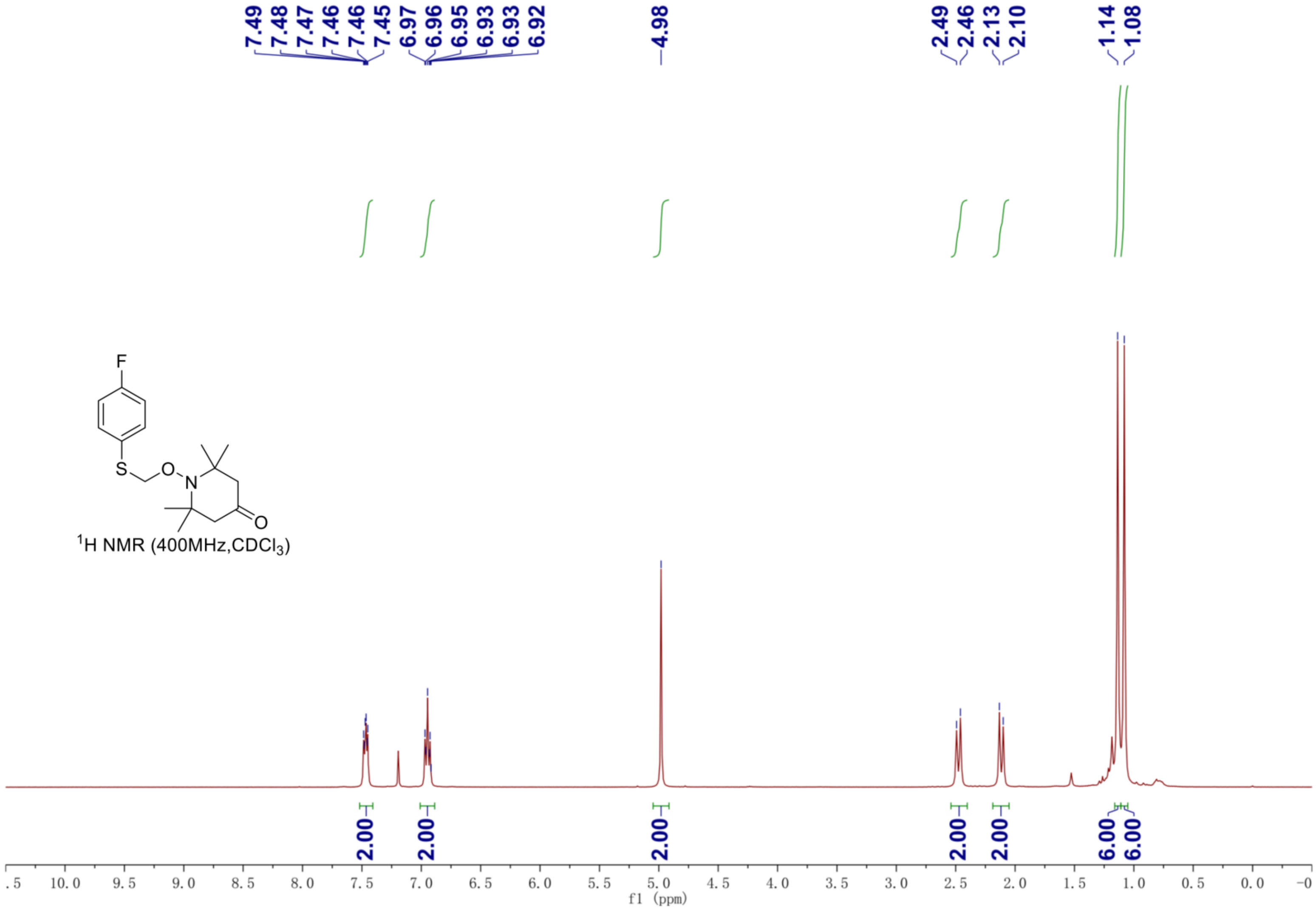

Go to table of contents

1-(((4-Fluorophenyl)thio)methoxy)-2,2,6,6-tetramethylpiperidin-4-one (3v)

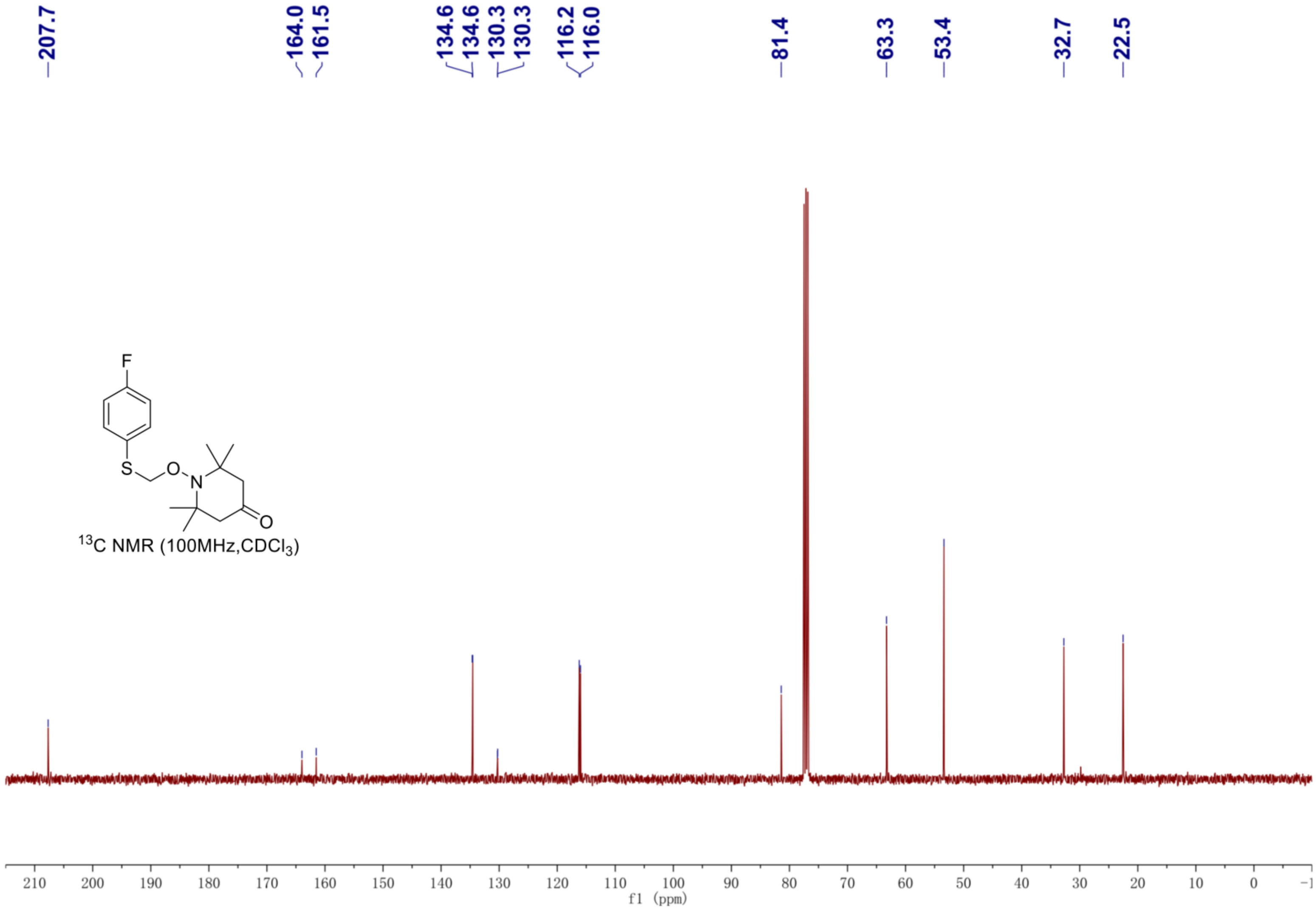

Go to table of contents

1-(((4-Fluorophenyl)thio)methoxy)-2,2,6,6-tetramethylpiperidin-4-one (3v)

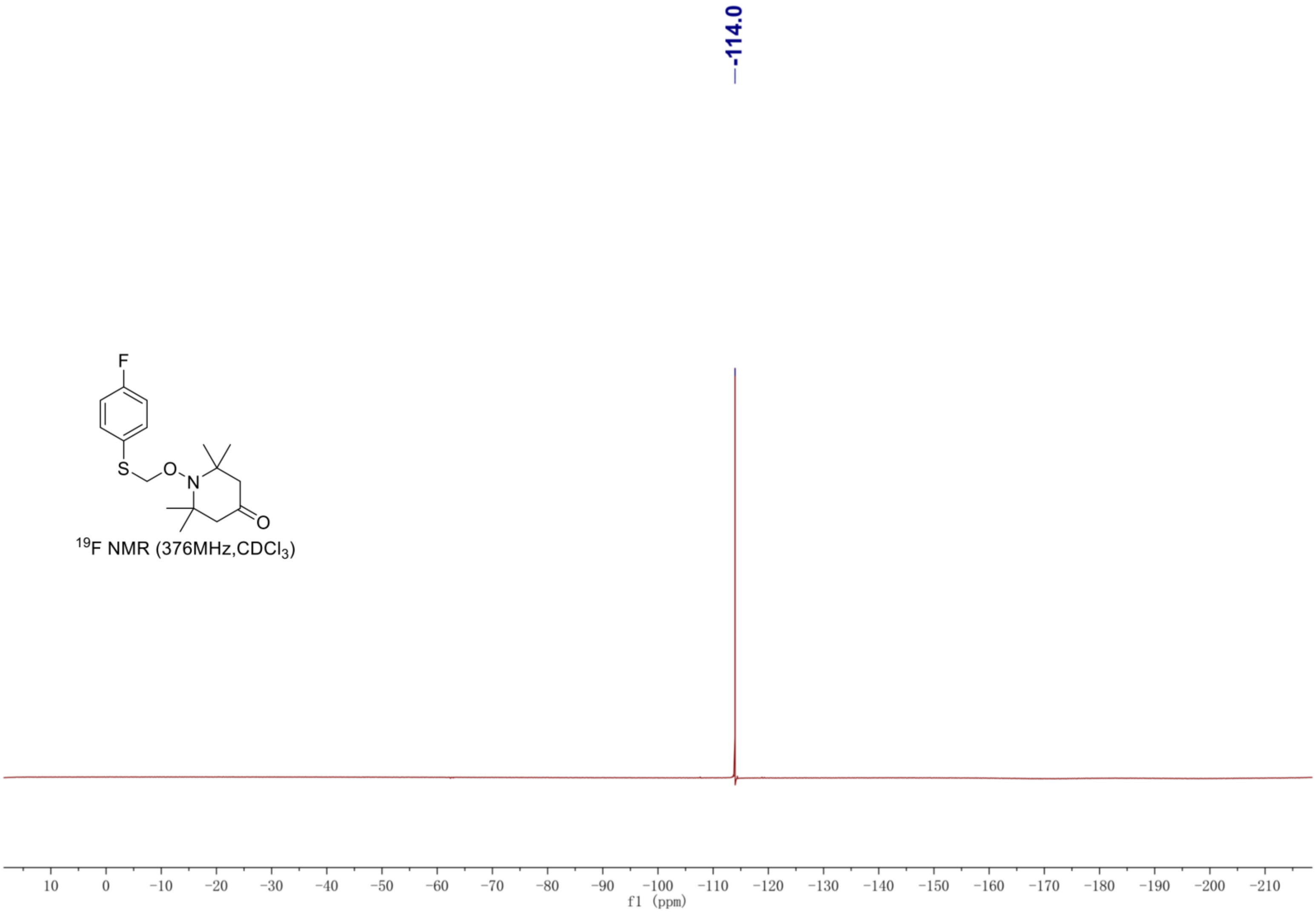

Go to table of contents

1-(((4-Chlorophenyl)thio)methoxy)-2,2,6,6-tetramethylpiperidin-4-one (3w)

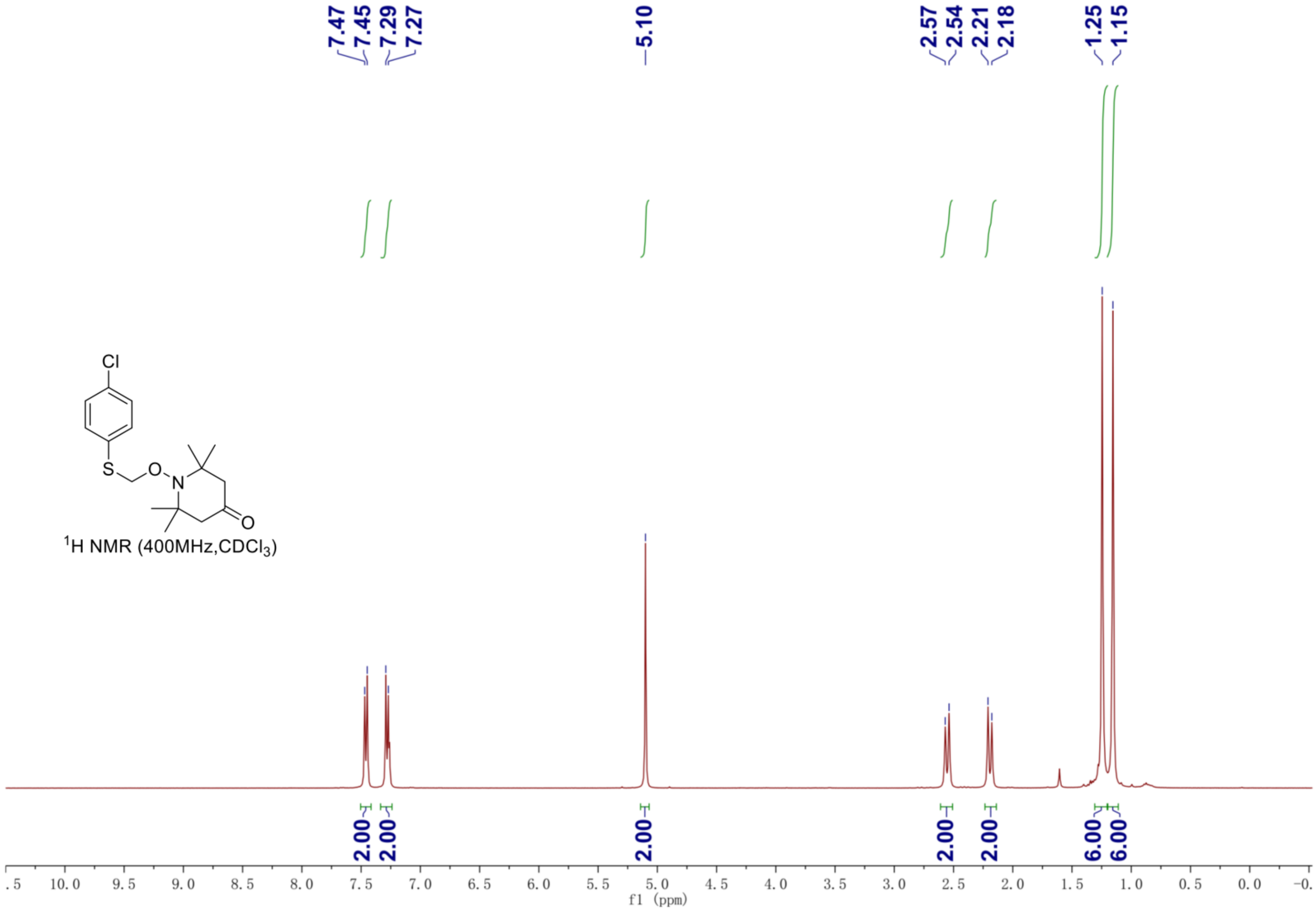

Go to table of contents

1-(((4-Chlorophenyl)thio)methoxy)-2,2,6,6-tetramethylpiperidin-4-one (3w)

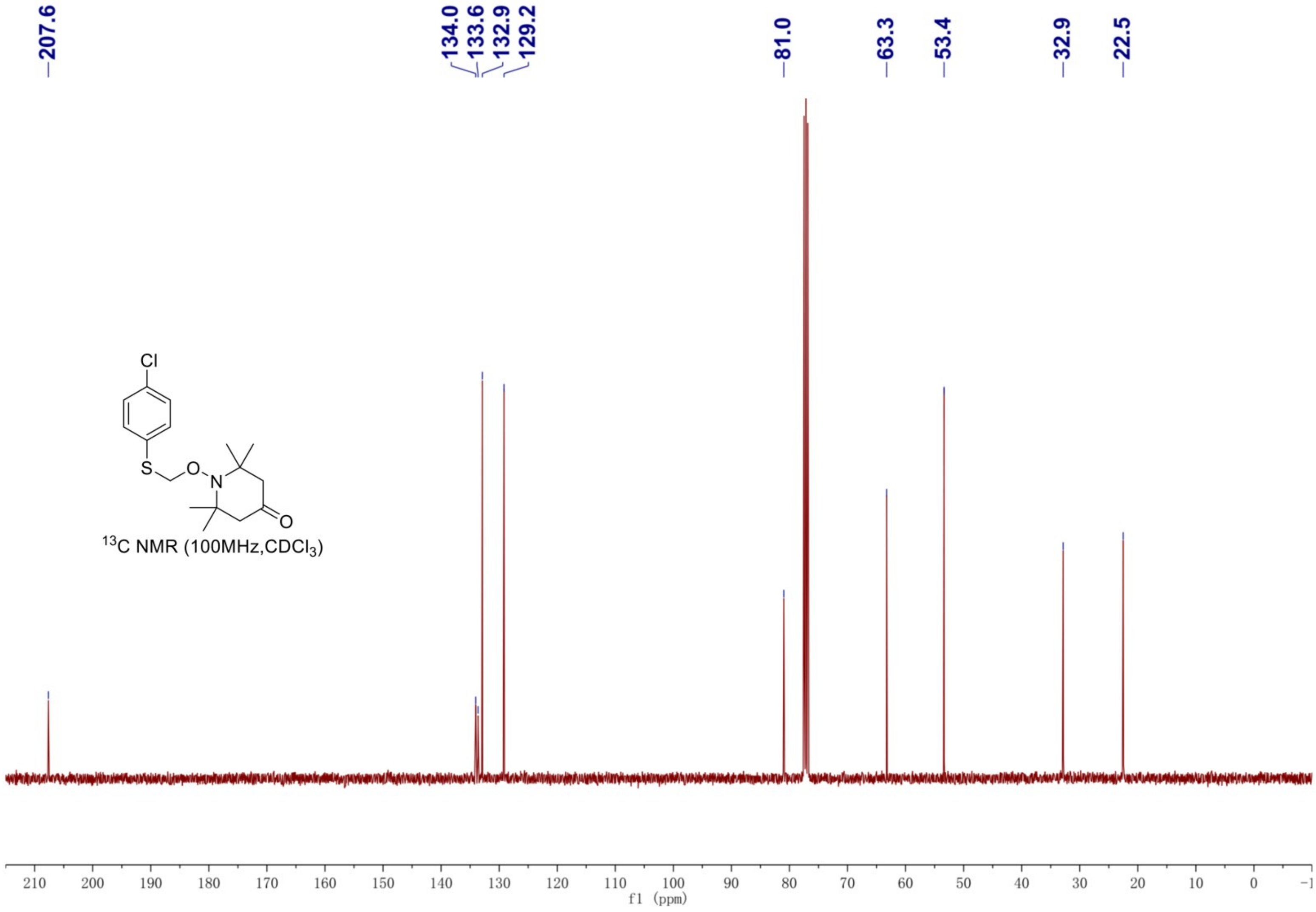

Go to table of contents

1-(((4-Bromophenyl)thio)methoxy)-2,2,6,6-tetramethylpiperidin-4-one (3x)

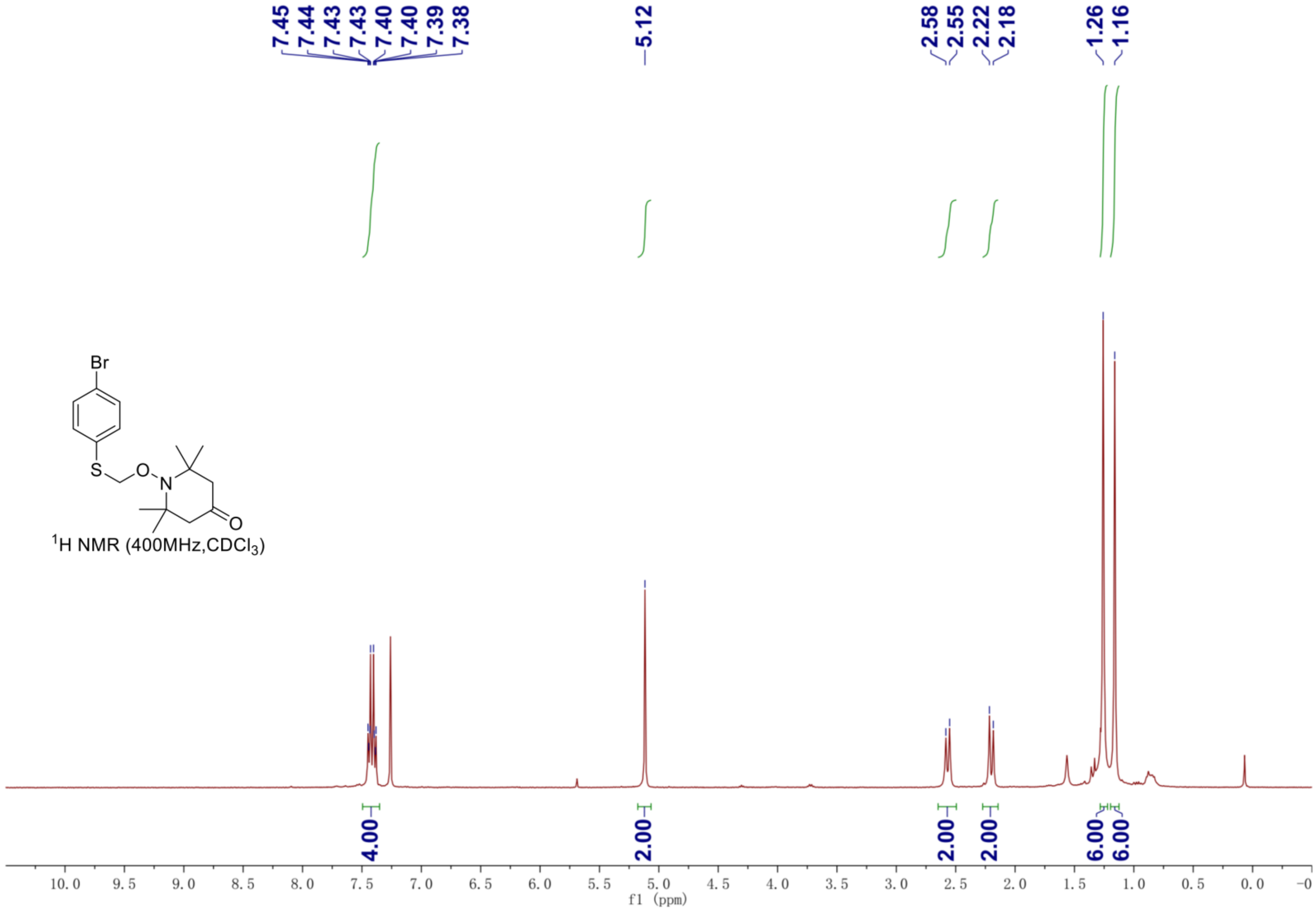

1-(((4-Bromophenyl)thio)methoxy)-2,2,6,6-tetramethylpiperidin-4-one (3x)

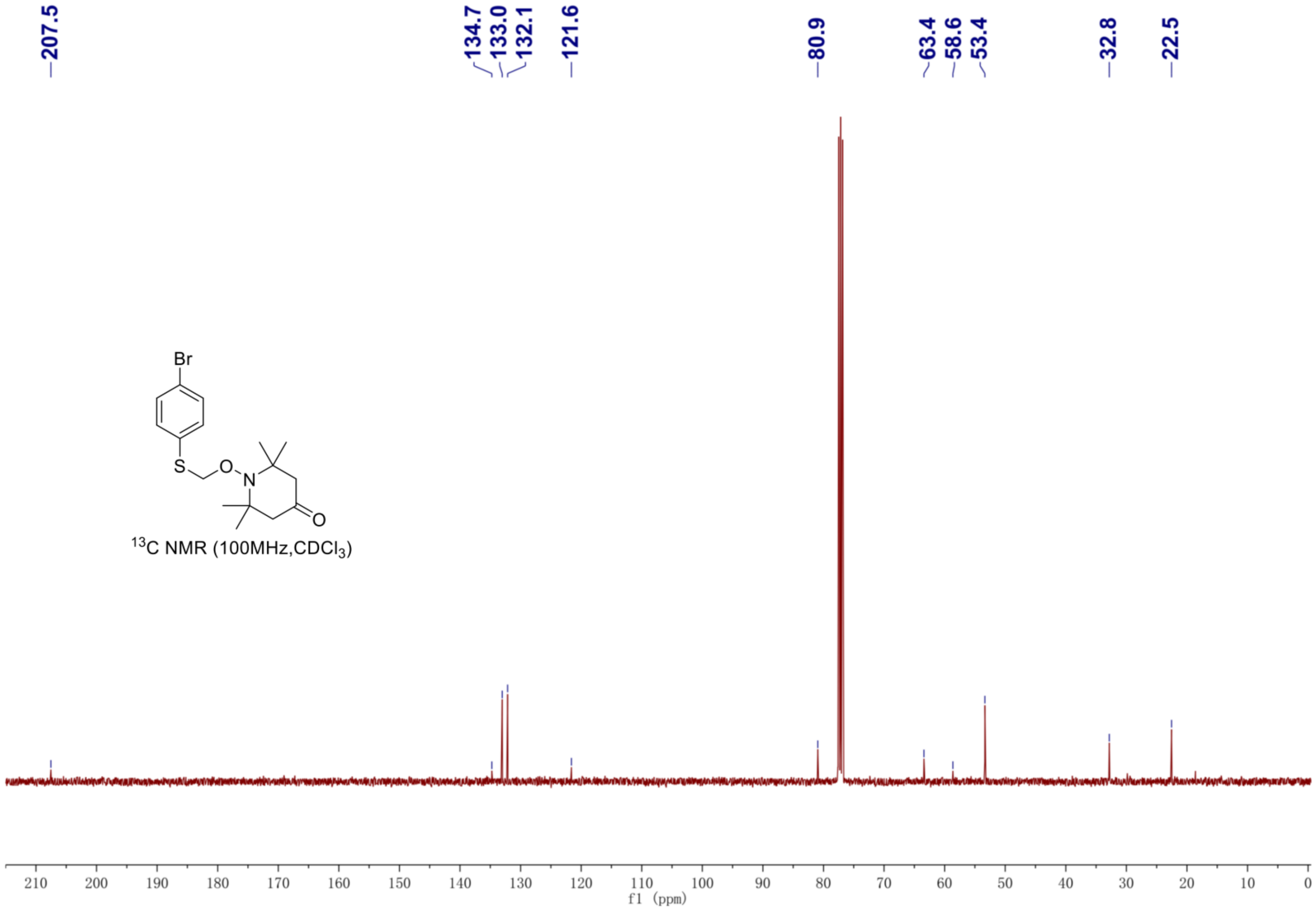

Go to table of contents

2,2,6,6-Tetramethyl-1-(((4-(4,4,5,5-tetramethyl-1,3,2-dioxaborolan-2-yl)phenyl)thio)methoxy)piperidin-4-one (3y)

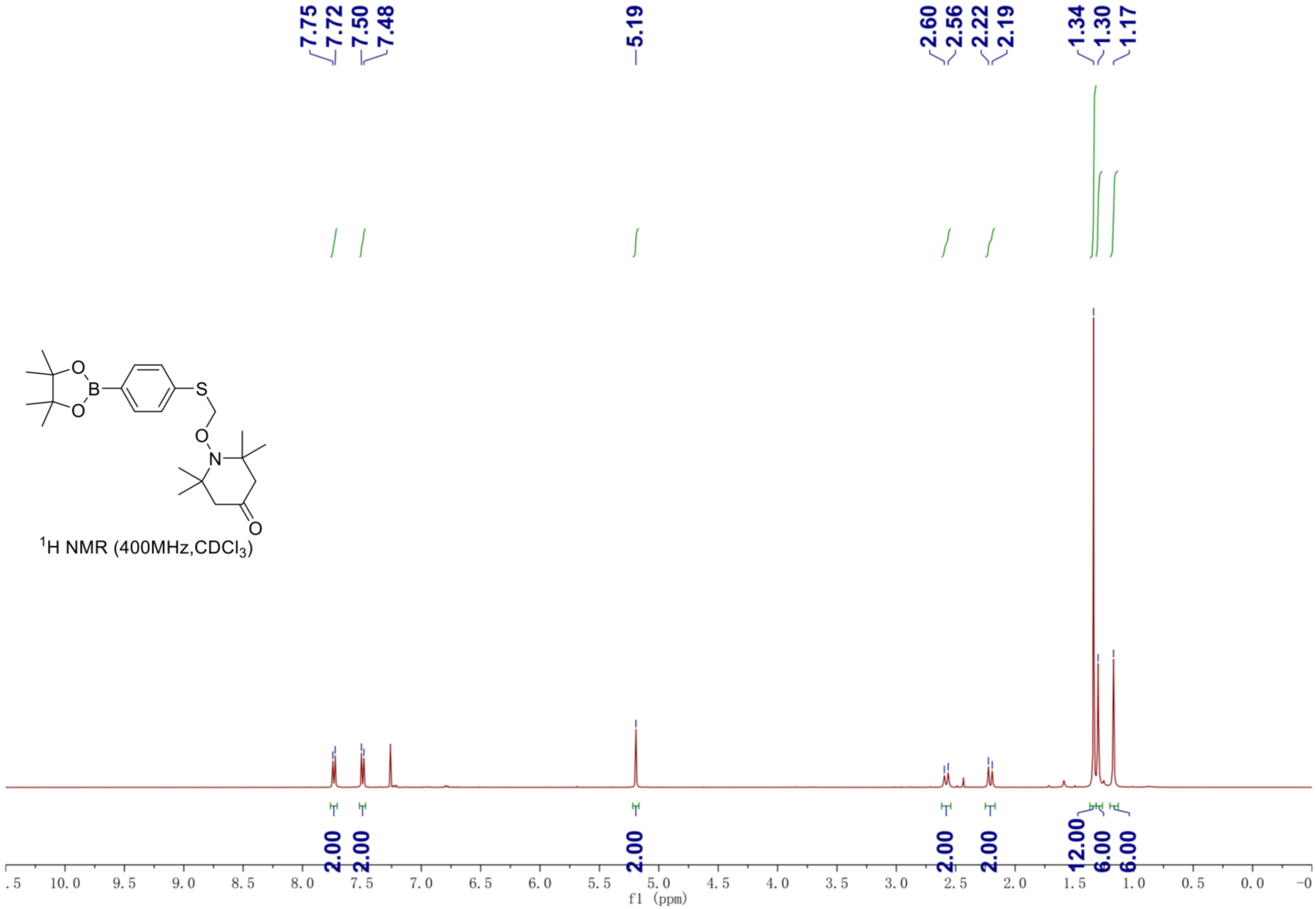

Go to table of contents

2,2,6,6-Tetramethyl-1-(((4-(4,4,5,5-tetramethyl-1,3,2-dioxaborolan-2-yl)phenyl)thio)methoxy)piperidin-4-one (3y)

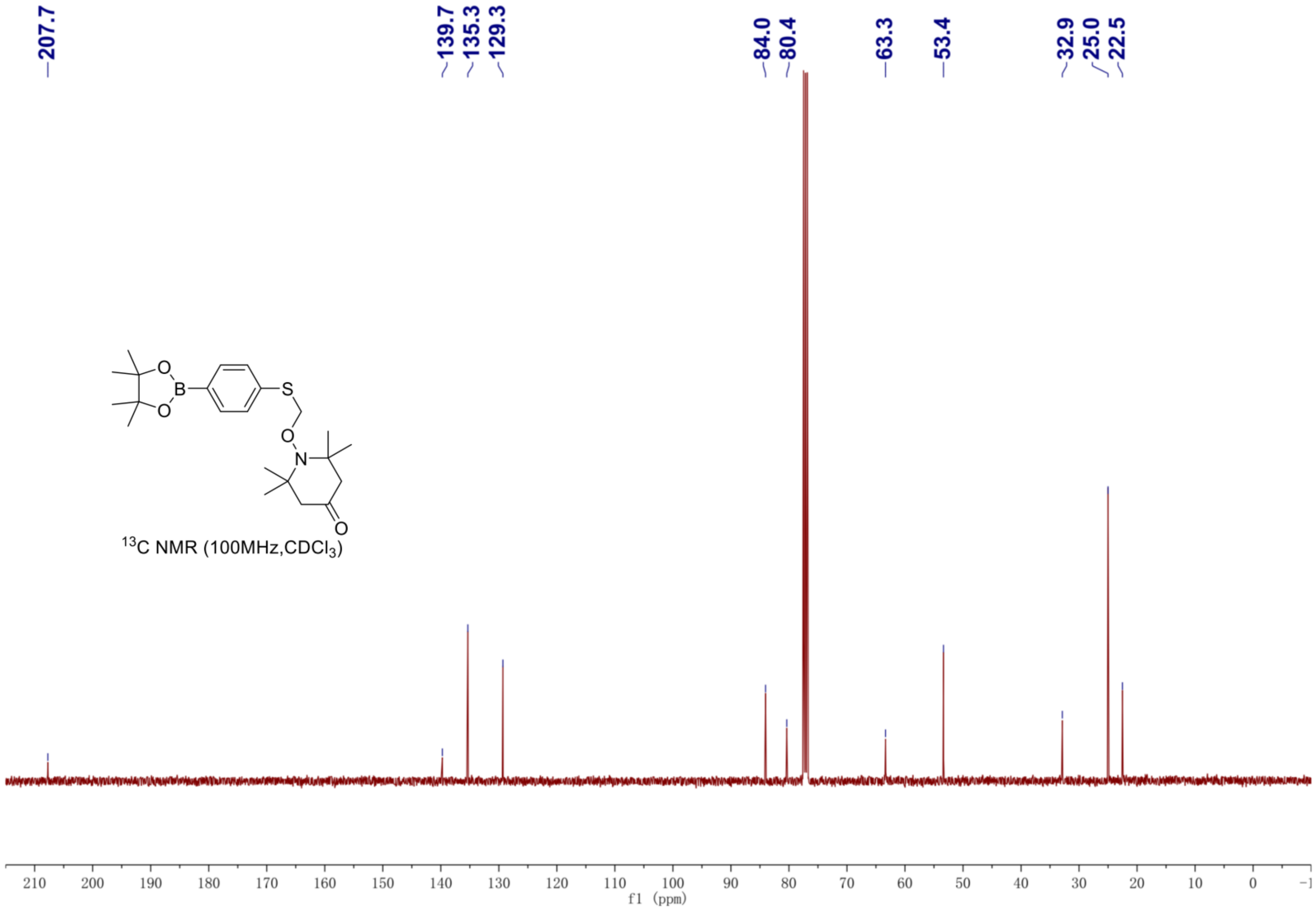

^{13}C NMR (100MHz, $CDCl_3$)

Go to table of contents

2,2,6,6-Tetramethyl-1-((p-tolylthio)methoxy)piperidin-4-one (3z)

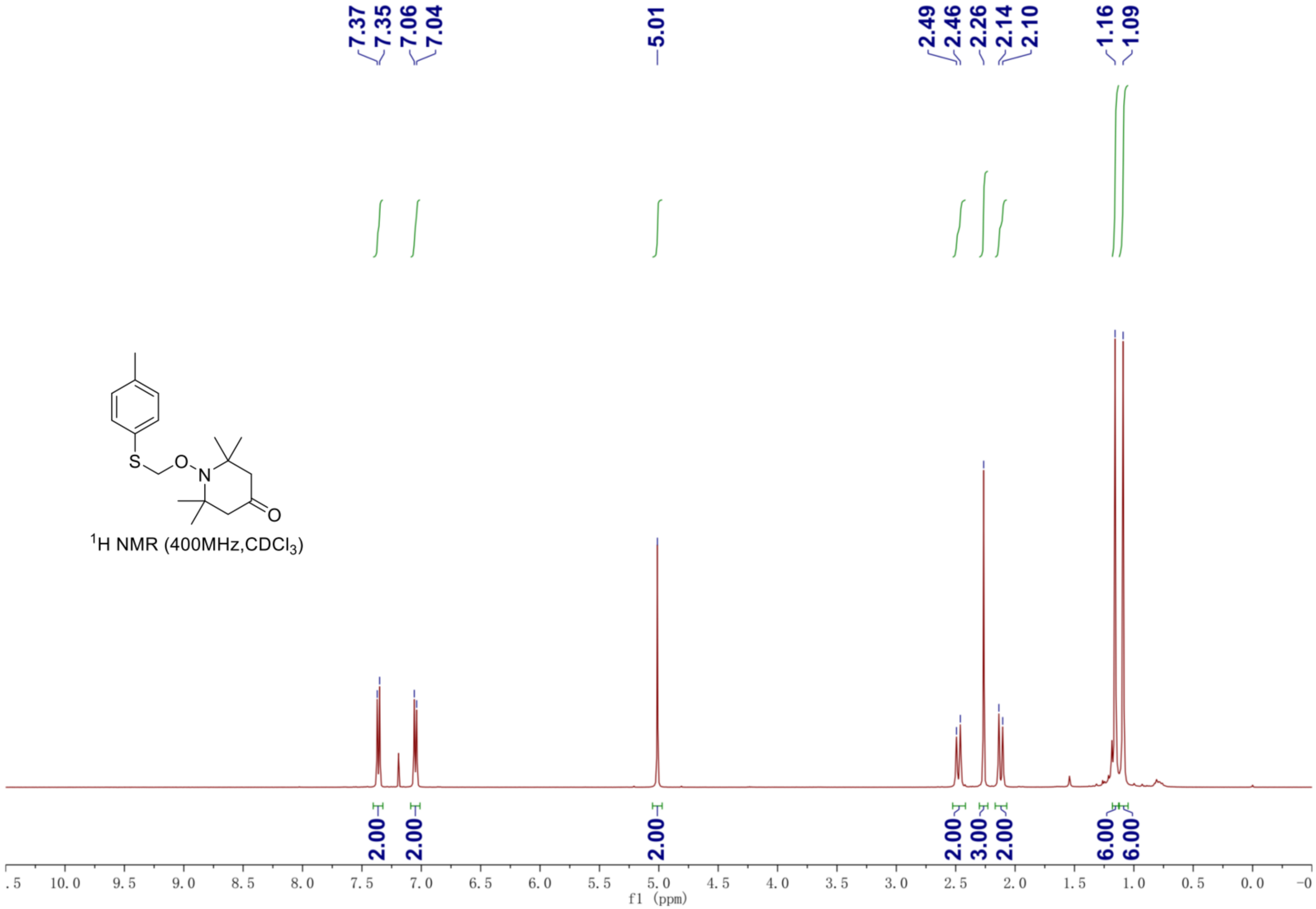

Go to table of contents

2,2,6,6-Tetramethyl-1-((p-tolylthio)methoxy)piperidin-4-one (3z)

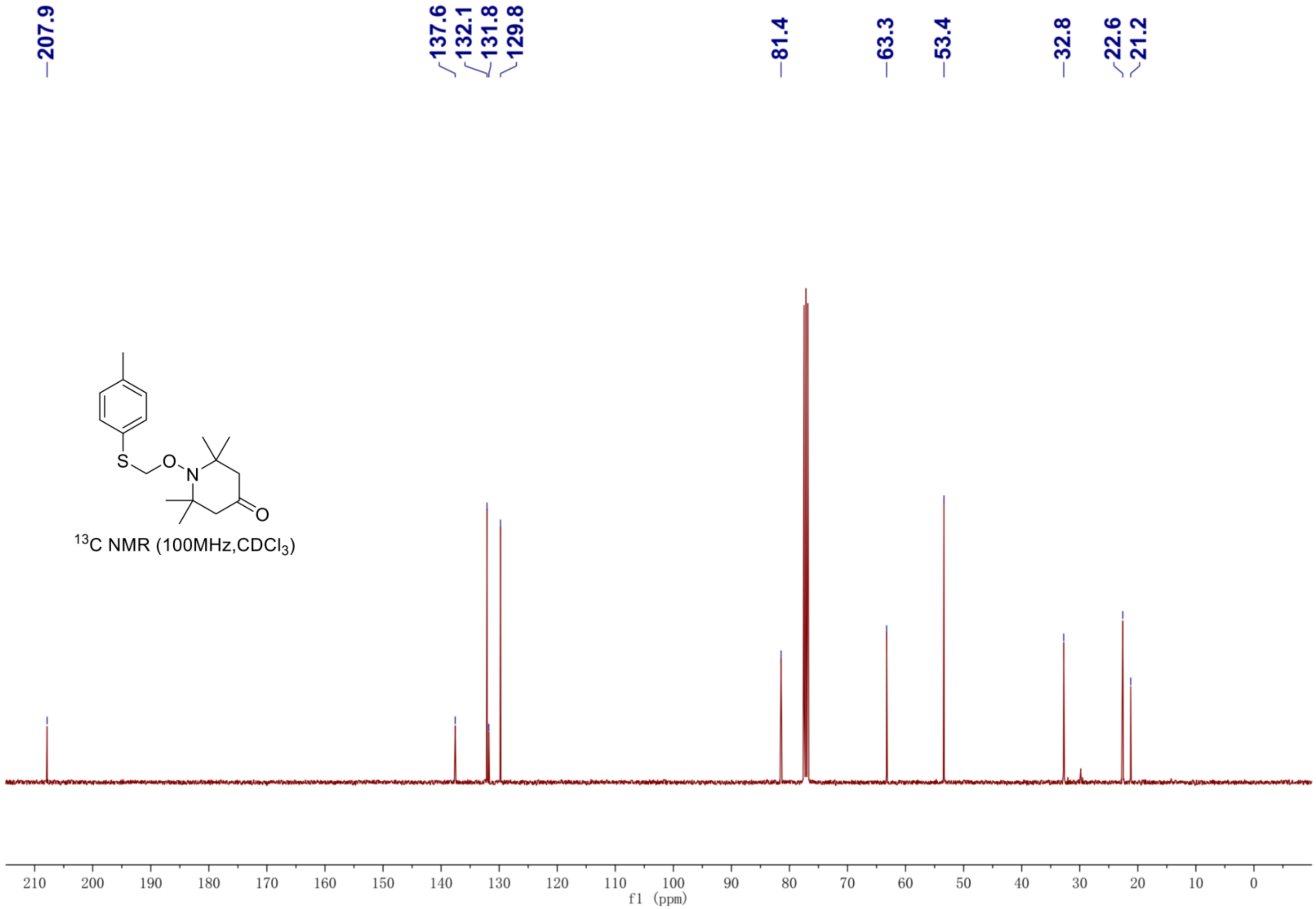

Go to table of contents

1-(((4-Hydroxyphenyl)thio)methoxy)-2,2,6,6-tetramethylpiperidin-4-one (3aa)

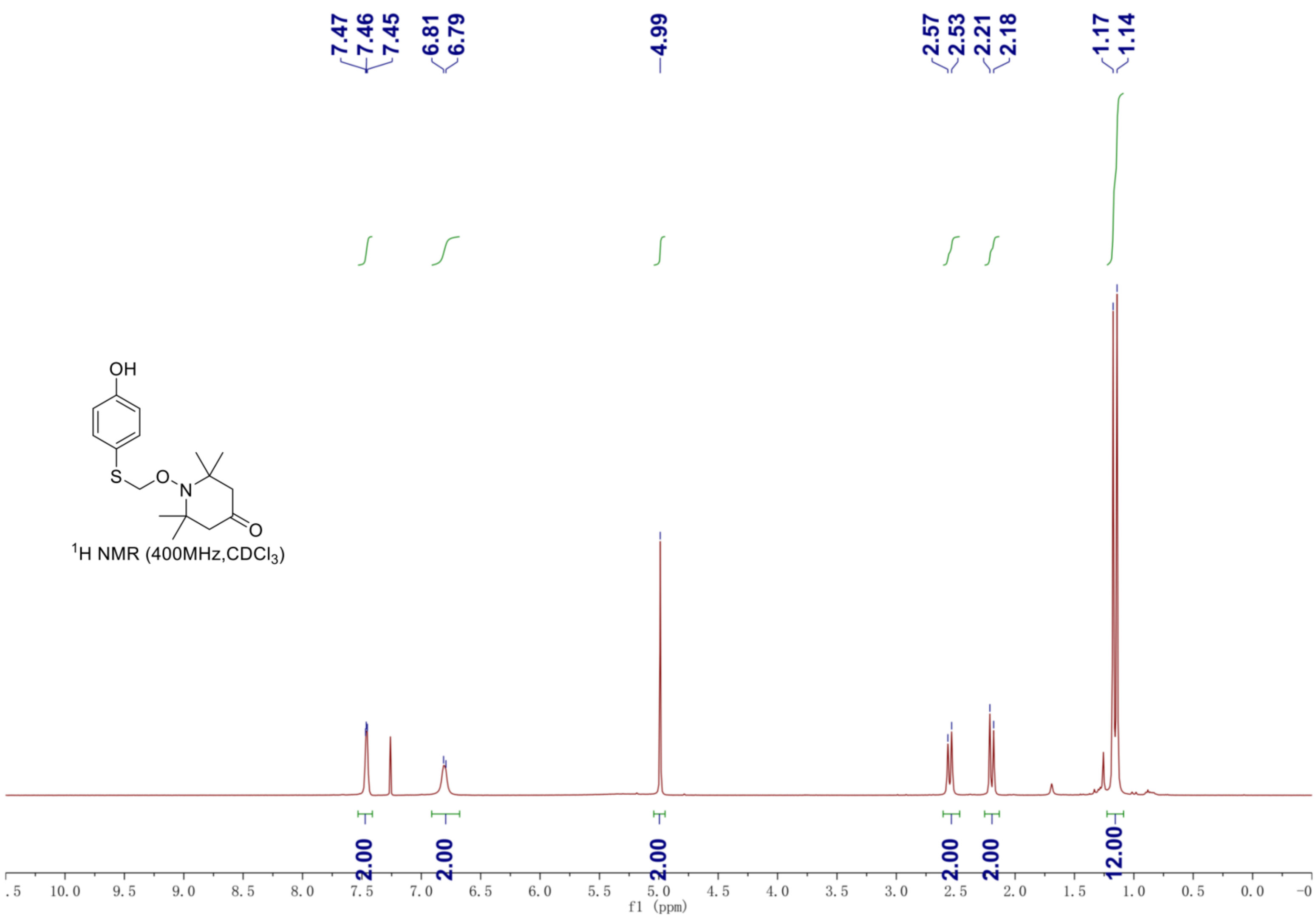

Go to table of contents

1-(((4-Hydroxyphenyl)thio)methoxy)-2,2,6,6-tetramethylpiperidin-4-one (3aa)

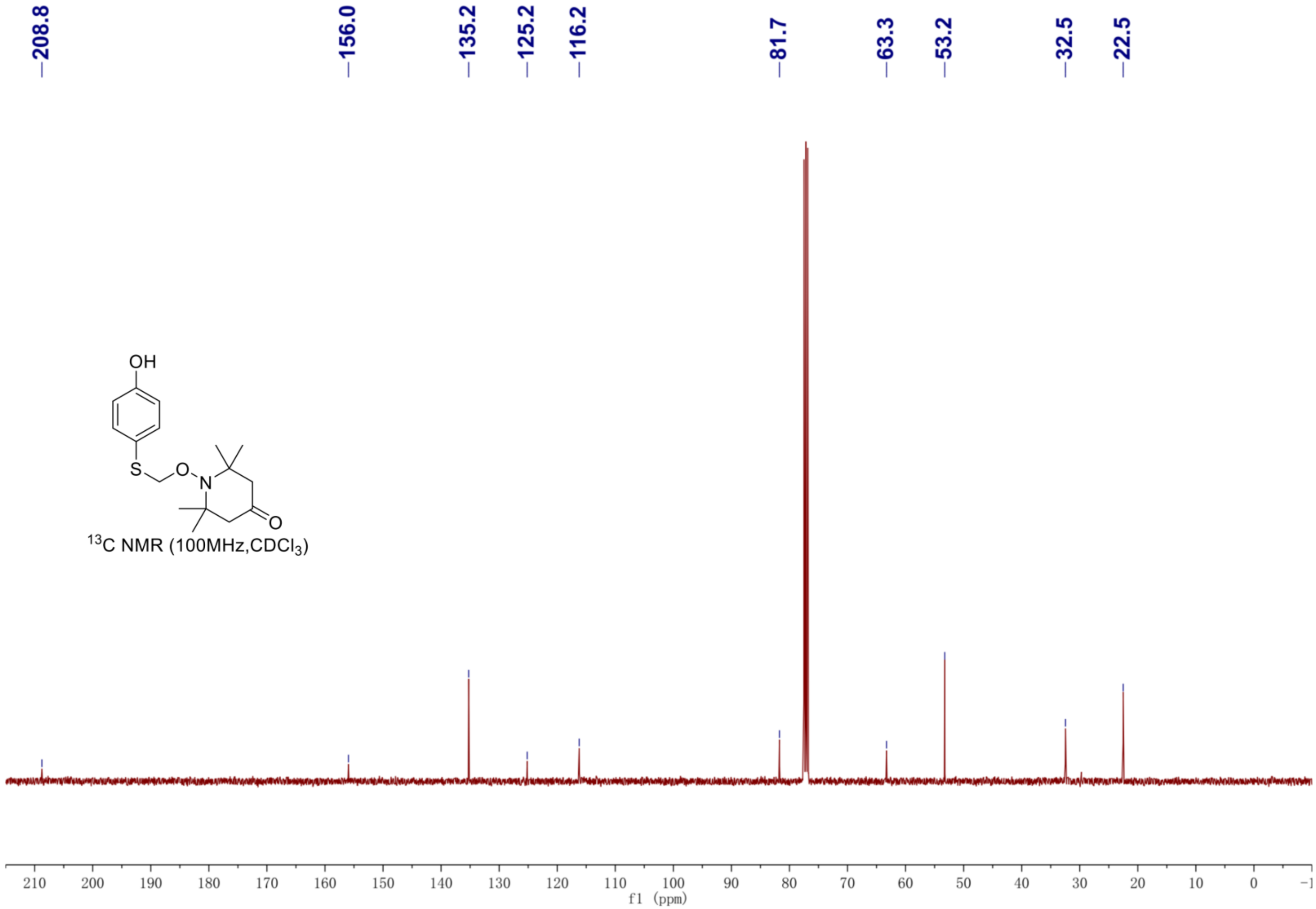

Go to table of contents

1-(((4-Methoxyphenyl)thio)methoxy)-2,2,6,6-tetramethylpiperidin-4-one (3ab-α) and 2,2,6,6-tetramethyl-1-((4-(methylthio)phenoxy)methoxy)piperidin-4-one (3ab-β)

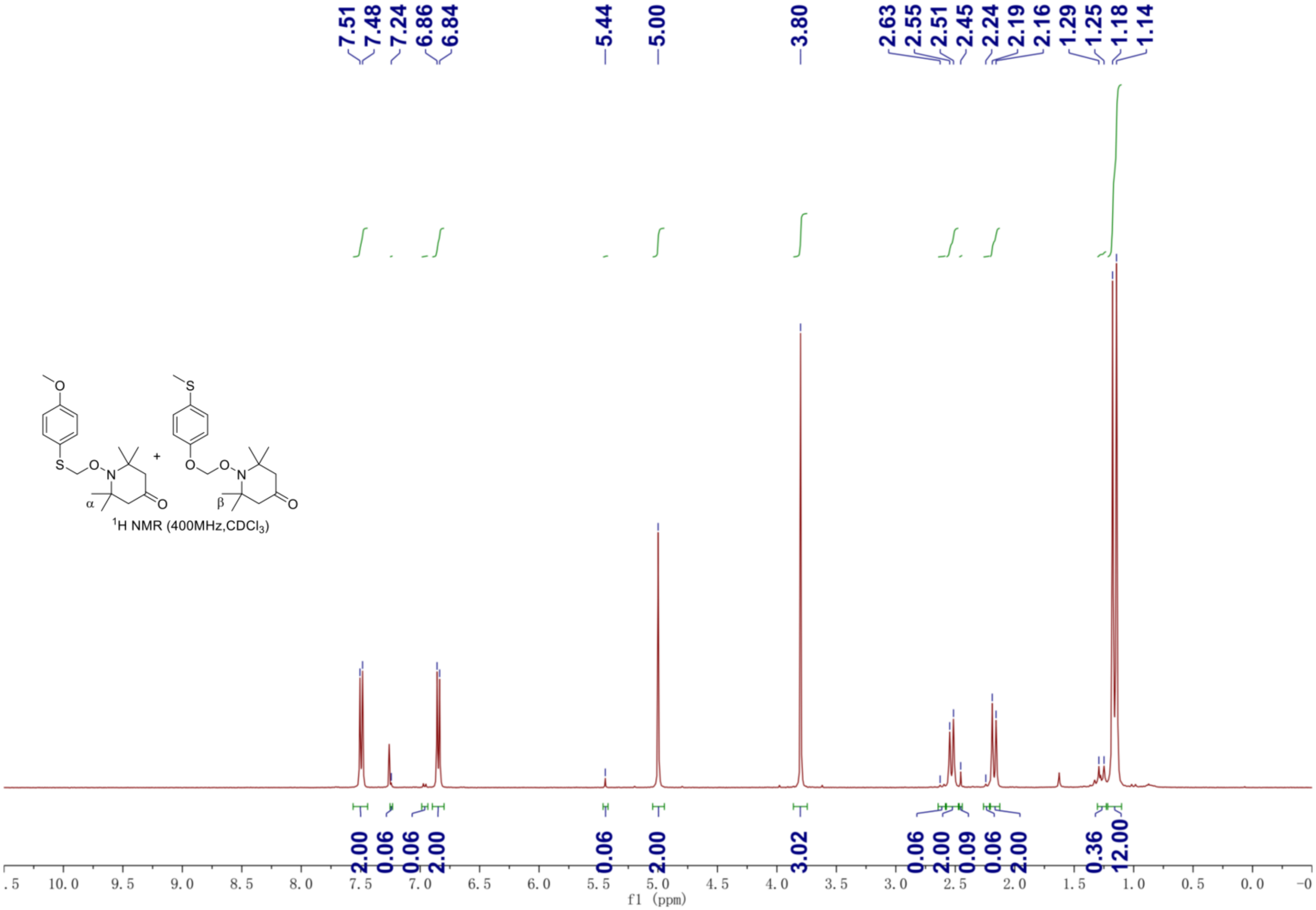

Go to table of contents

1-(((4-Methoxyphenyl)thio)methoxy)-2,2,6,6-tetramethylpiperidin-4-one (3ab-α) and 2,2,6,6-tetramethyl-1-((4-(methylthio)phenoxy)methoxy)piperidin-4-one (3ab-β)

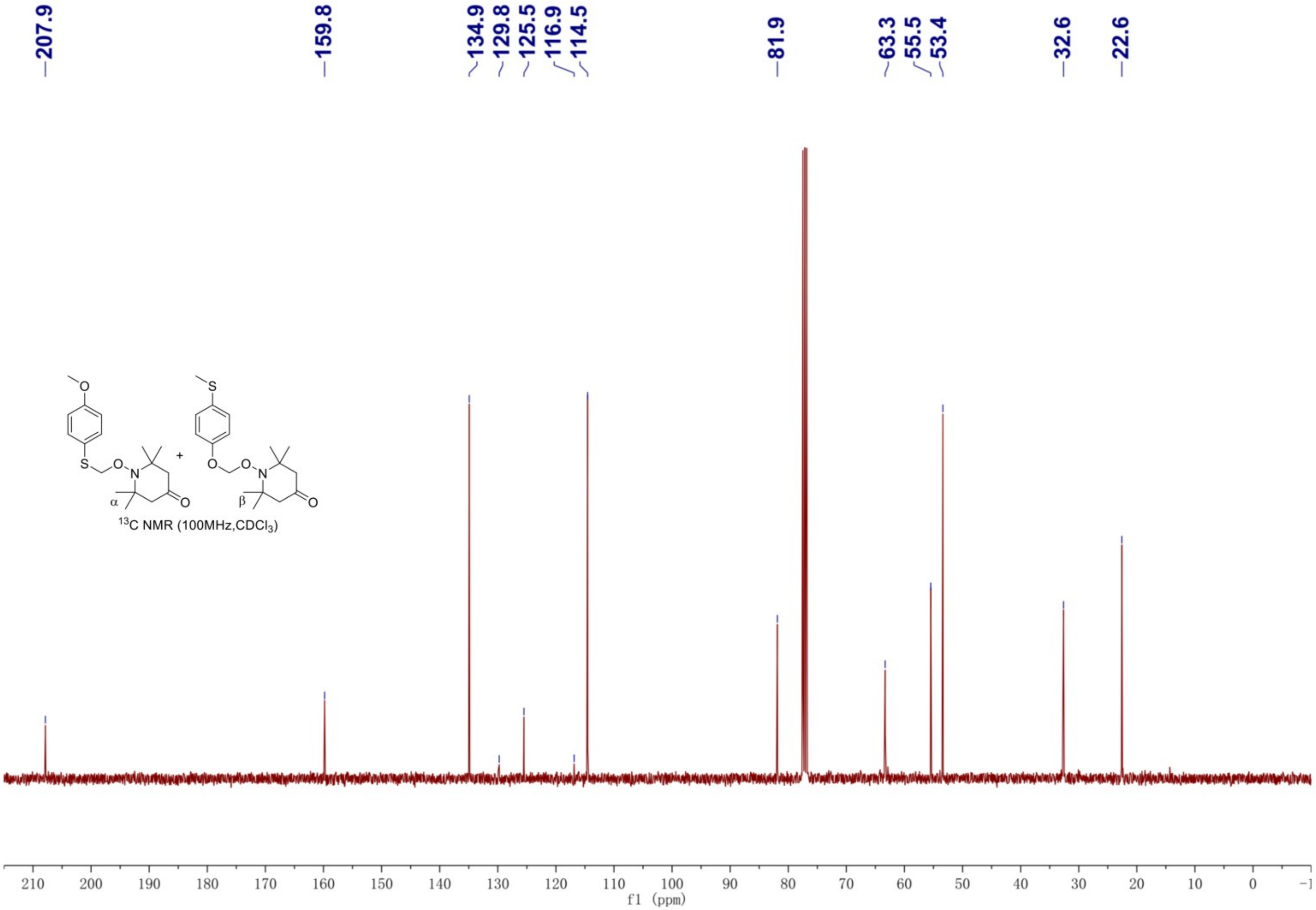

Go to table of contents

Methyl 2-((((2,2,6,6-tetramethyl-4-oxopiperidin-1-yl)oxy)methyl)thio)benzoate (3ac)

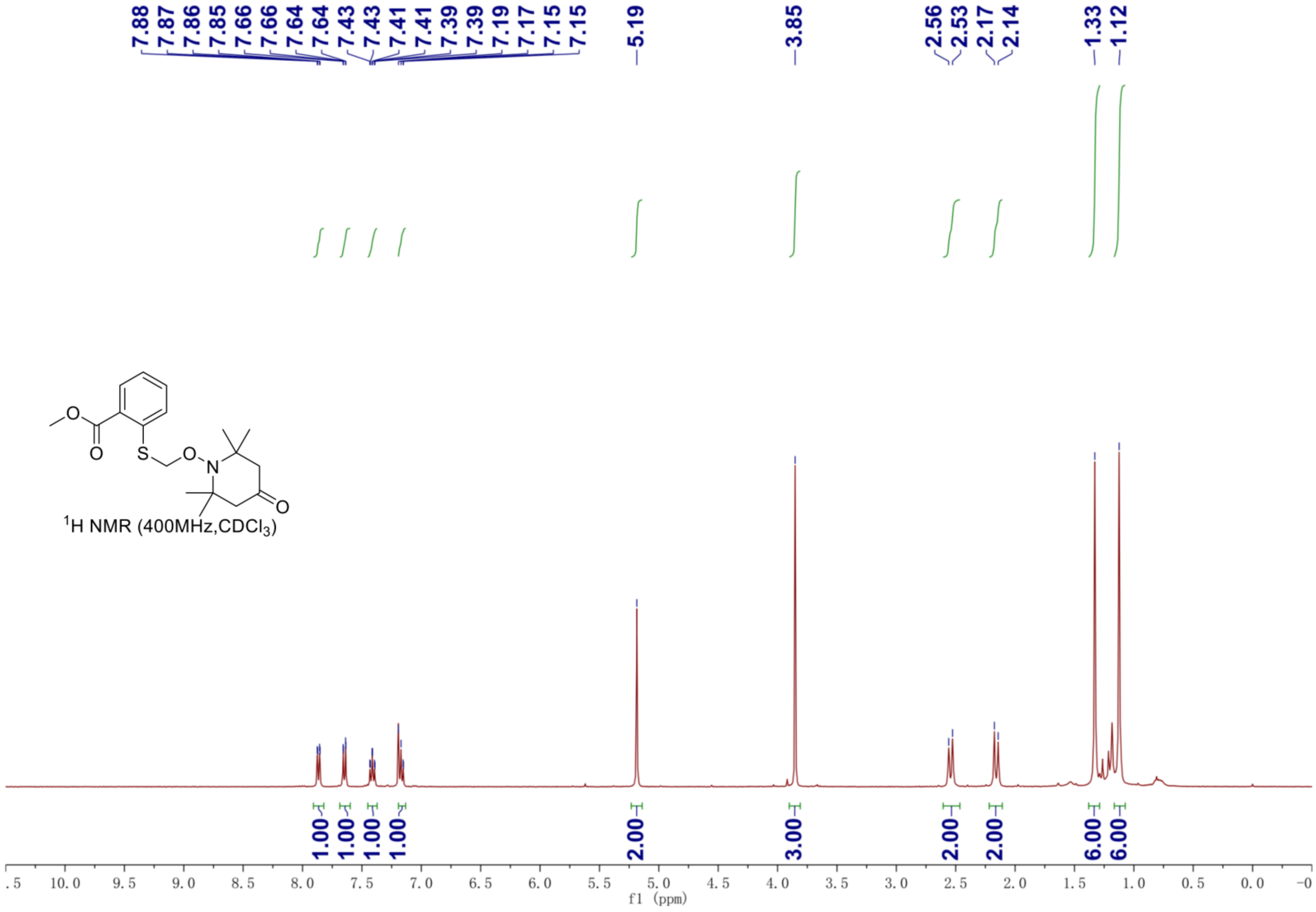

Go to table of contents

Methyl 2-((((2,2,6,6-tetramethyl-4-oxopiperidin-1-yl)oxy)methyl)thio)benzoate (3ac)

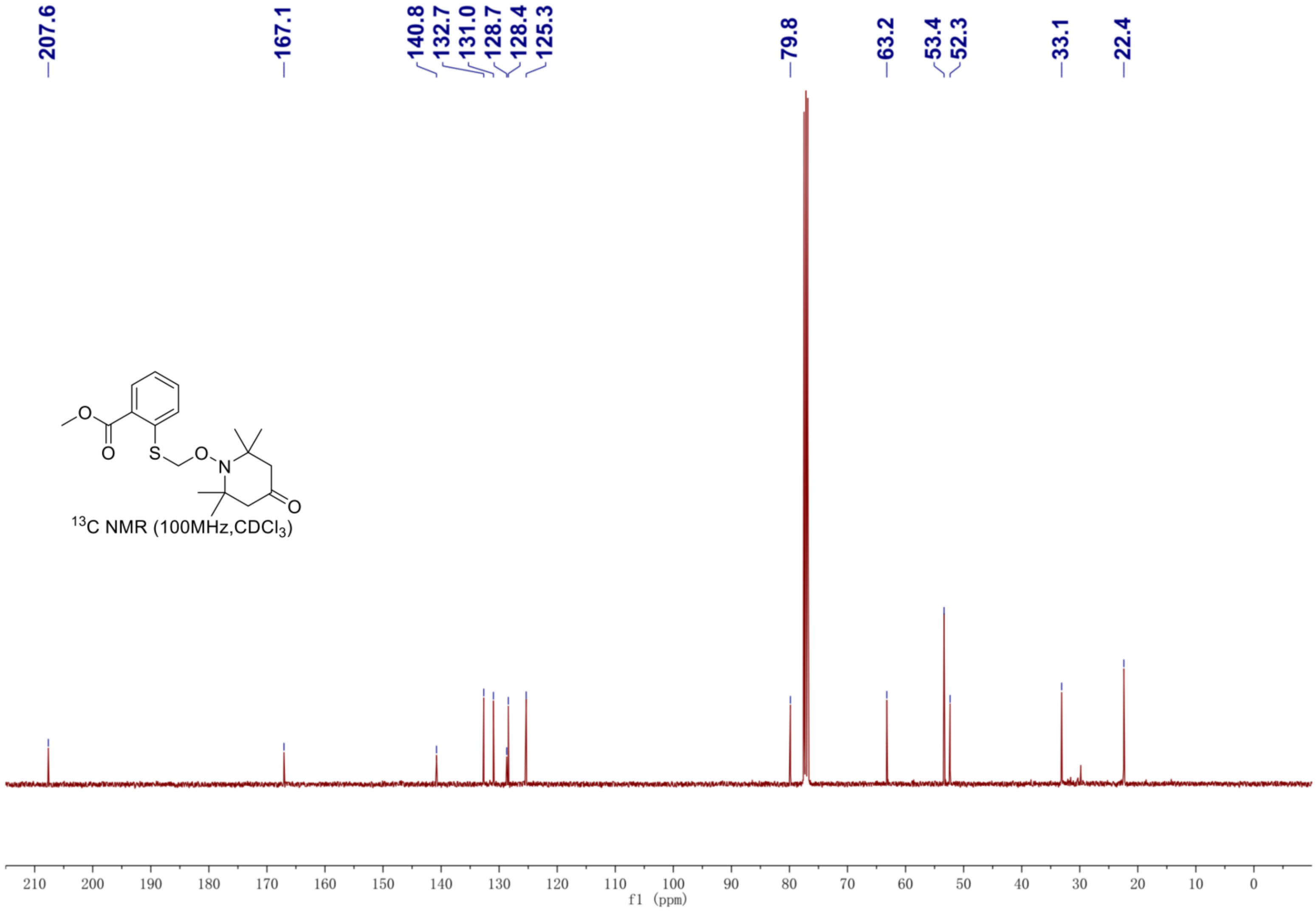

Go to table of contents

2,2,6,6-Tetramethyl-1-(((3-(4,4,5,5-tetramethyl-1,3,2-dioxaborolan-2-yl)phenyl)thio)methoxy)piperidin-4-one (3ad)

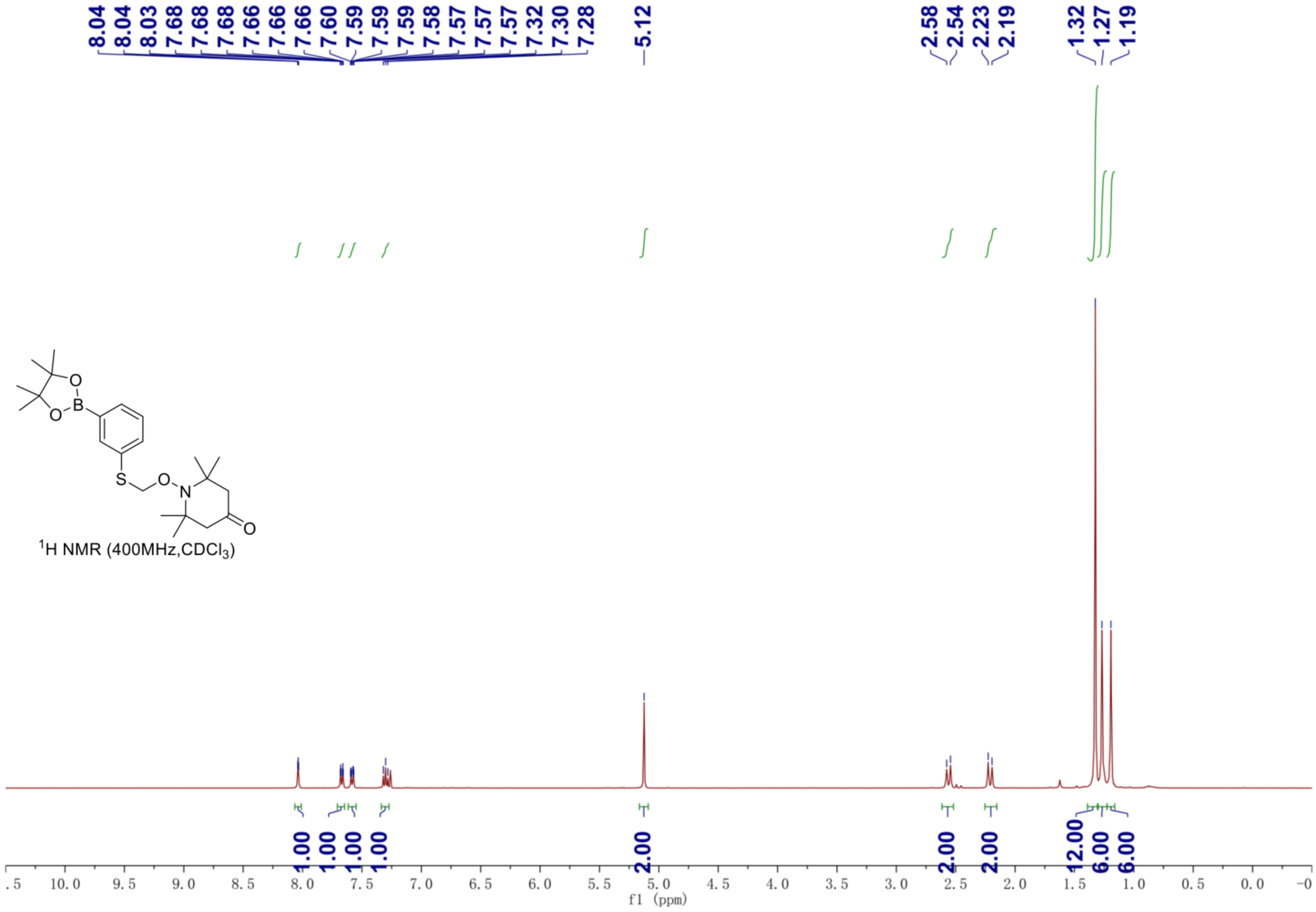

Go to table of contents

2,2,6,6-Tetramethyl-1-(((3-(4,4,5,5-tetramethyl-1,3,2-dioxaborolan-2-yl)phenyl)thio)methoxy)piperidin-4-one (3ad)

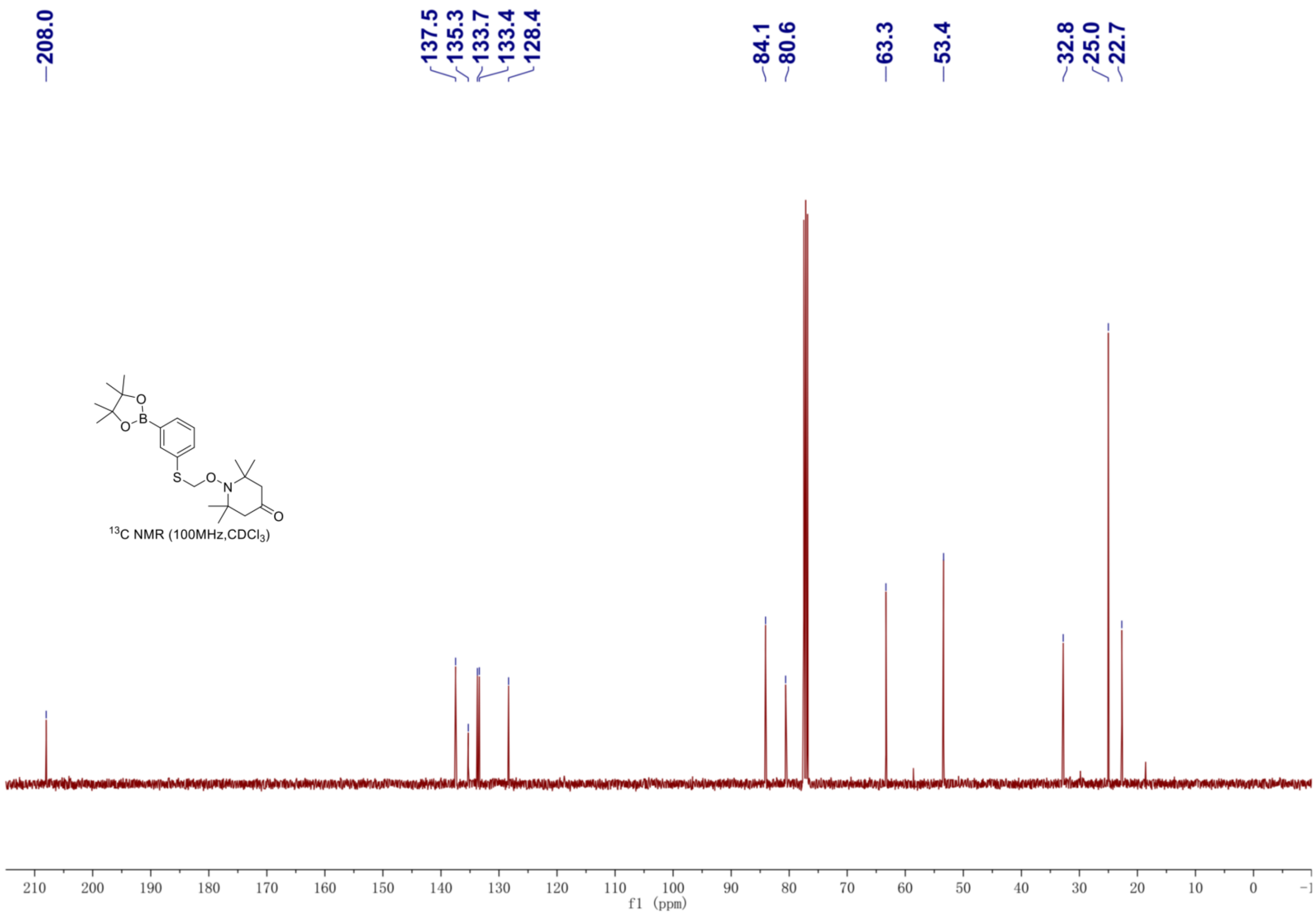

2,2,6,6-Tetramethyl-1-((1,1,2,2,2-pentamethyldisilaneyl)methoxy)piperidine (3ae)

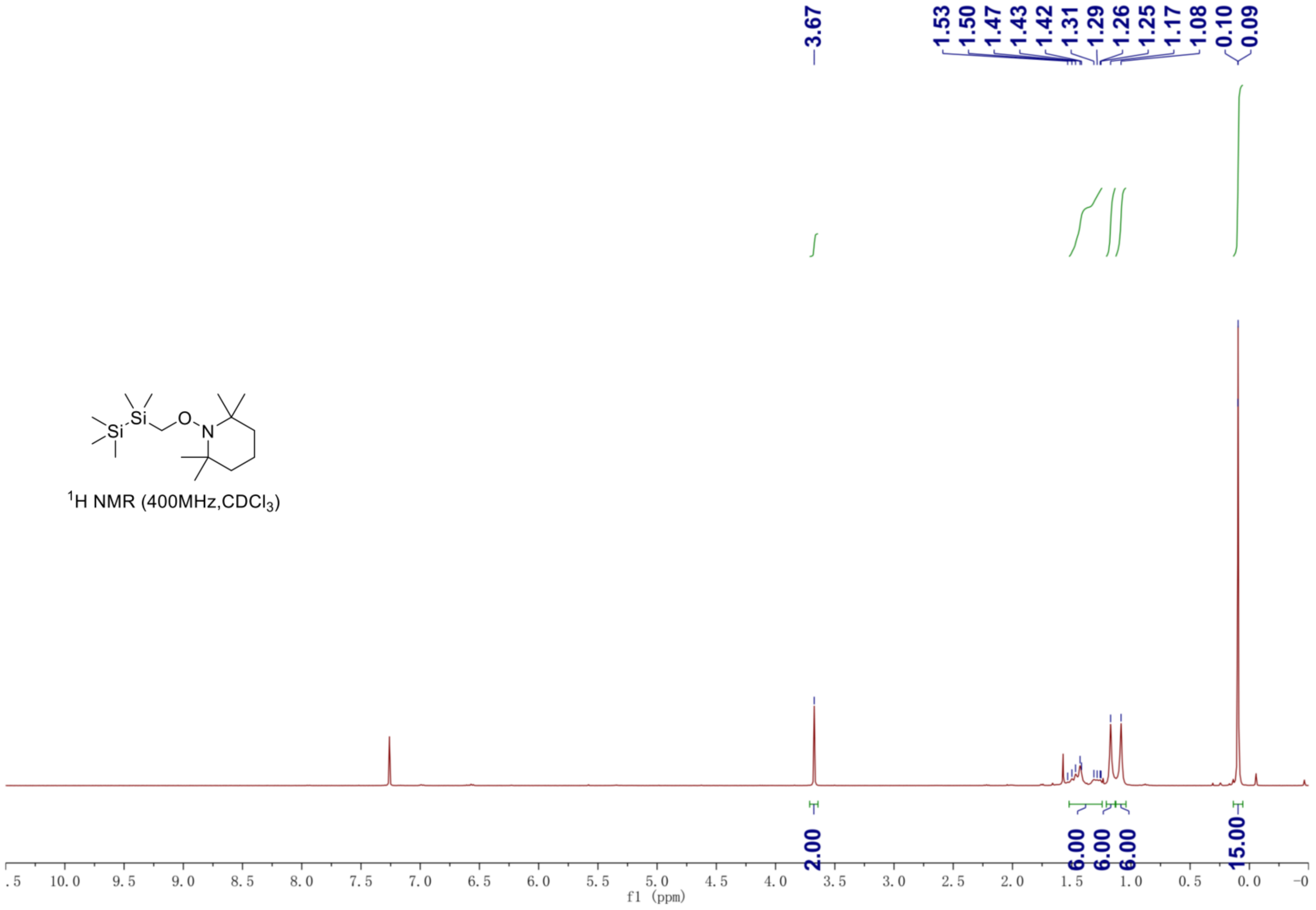

^{1}H NMR (400MHz,$CDCl_3$)

Go to table of contents

2,2,6,6-Tetramethyl-1-((1,1,2,2,2-pentamethyldisilaneyl)methoxy)piperidine (3ae)

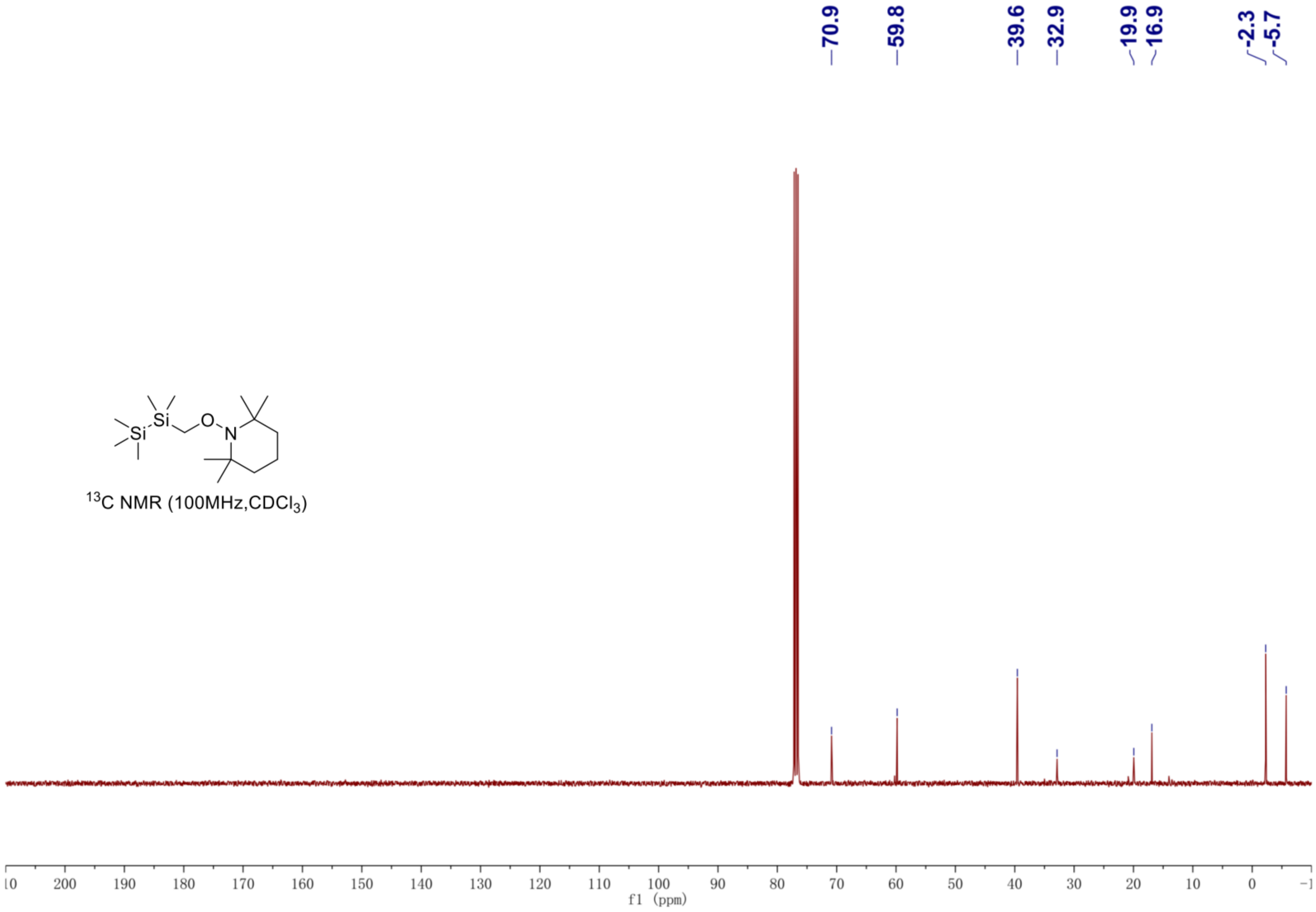

2,2,6,6-Tetramethylpiperidin-1-yl benzoate (3af)

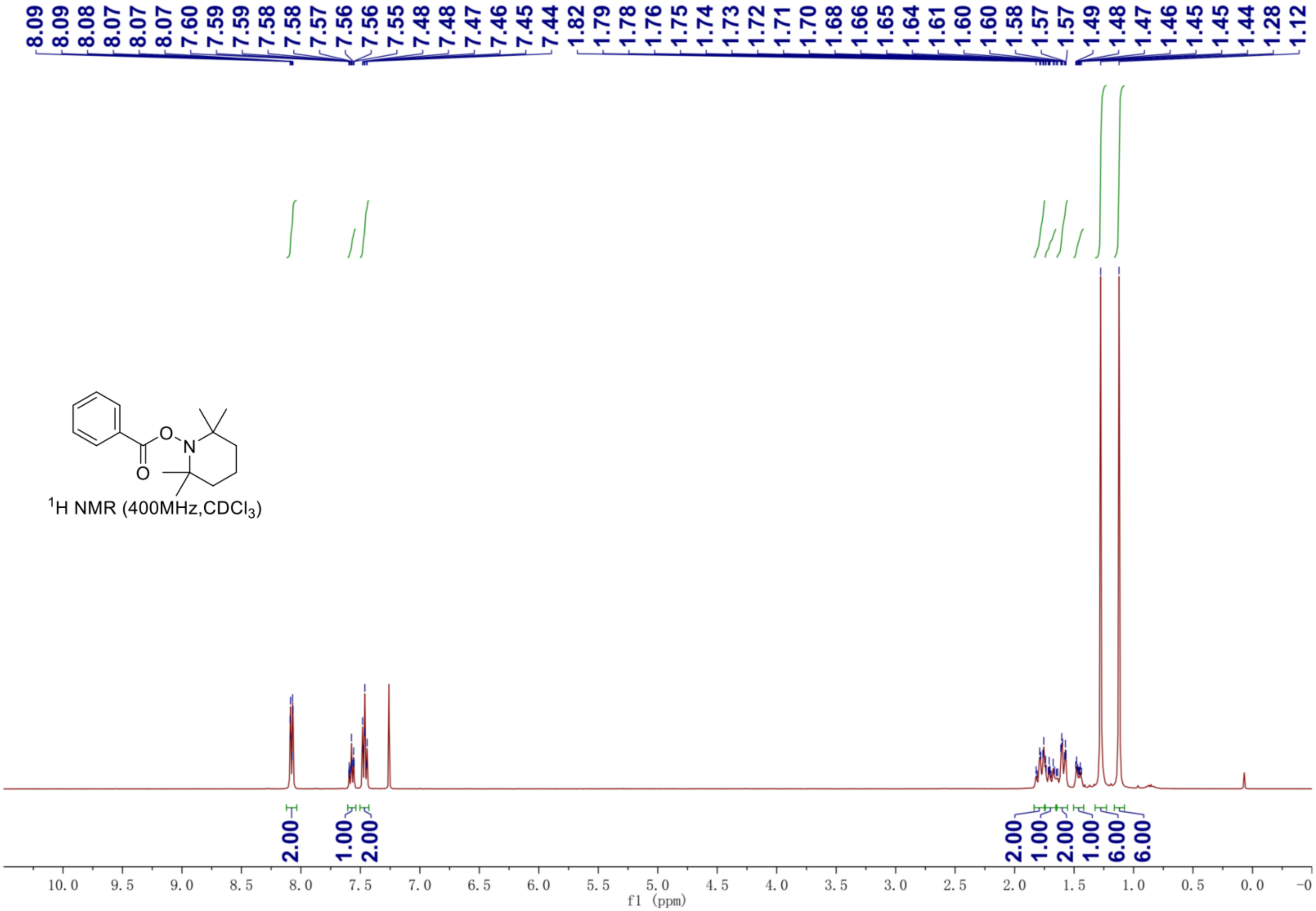

Go to table of contents

2,2,6,6-Tetramethylpiperidin-1-yl benzoate (3af)

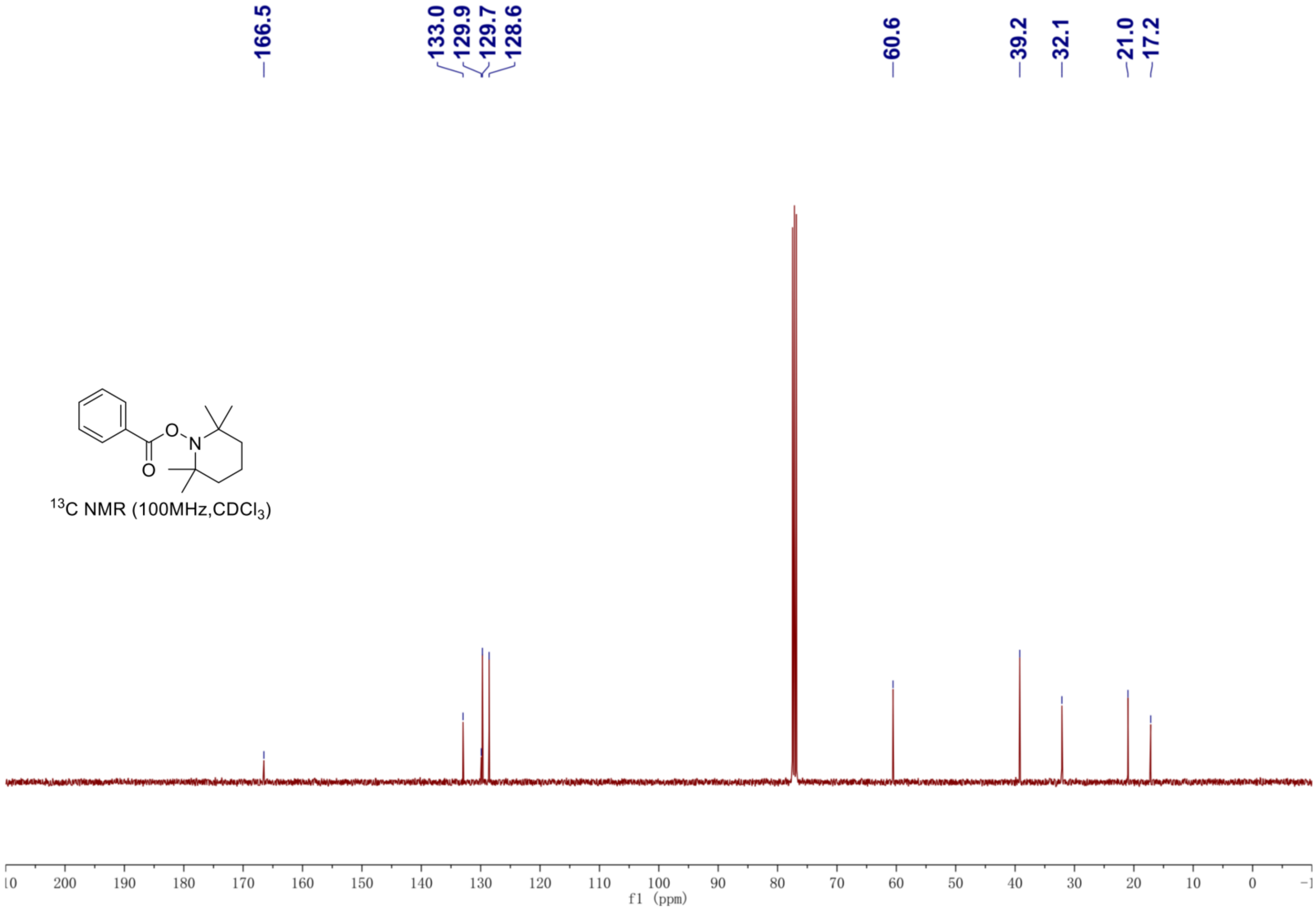

Go to table of contents

1-((1-(4-(*tert*-Butyl)phenyl)propan-2-yl)oxy)-2,2,6,6-tetramethylpiperidin-4-ol (3ag)

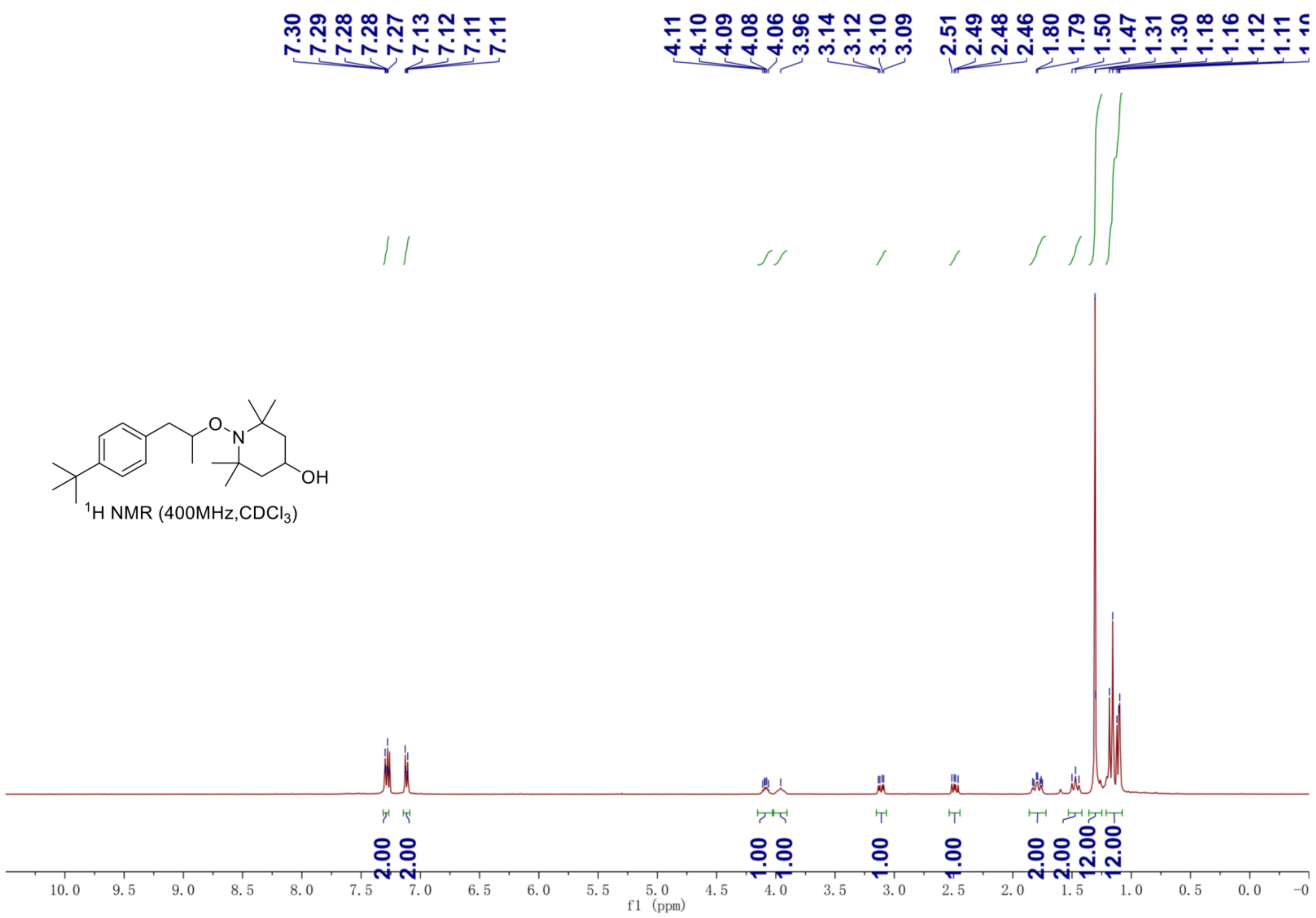

Go to table of contents

1-((1-(4-(*tert*-Butyl)phenyl)propan-2-yl)oxy)-2,2,6,6-tetramethylpiperidin-4-ol (3ag)

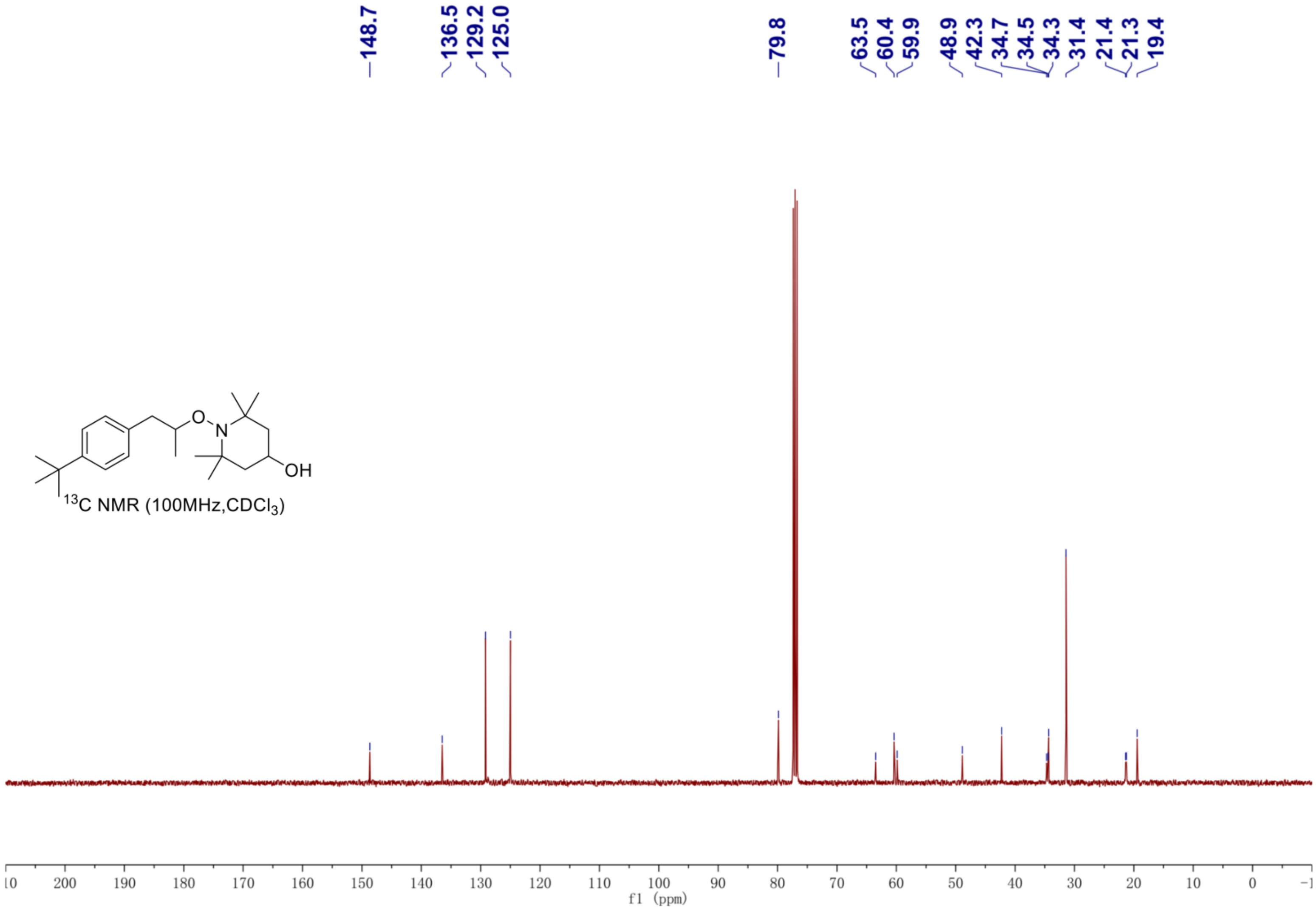

Go to table of contents

Methyl-3-cyclohexyl-2-(4-(trifluoromethyl)phenyl)propanoate (4a)

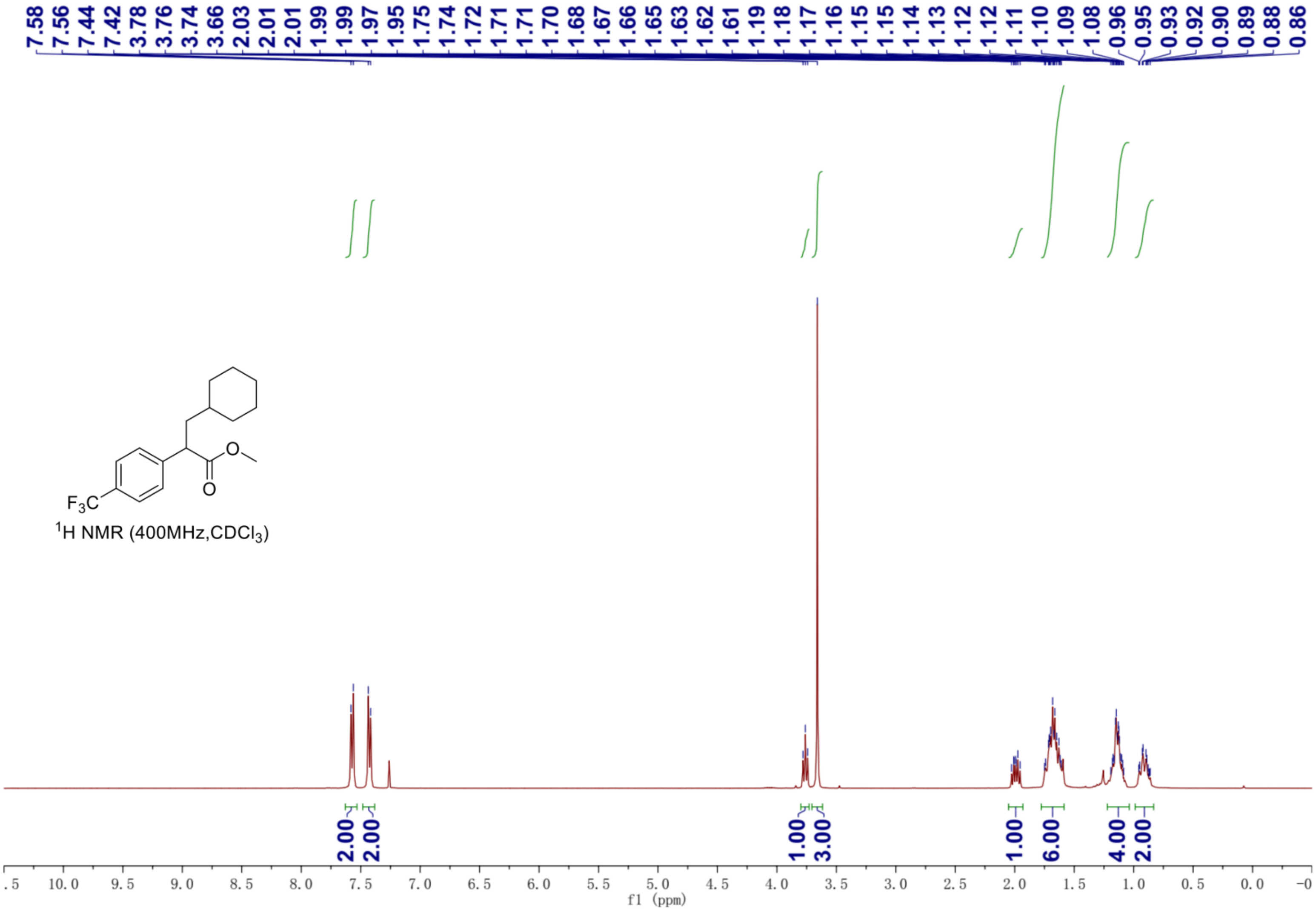

Go to table of contents

Methyl-3-cyclohexyl-2-(4-(trifluoromethyl)phenyl)propanoate (4a)

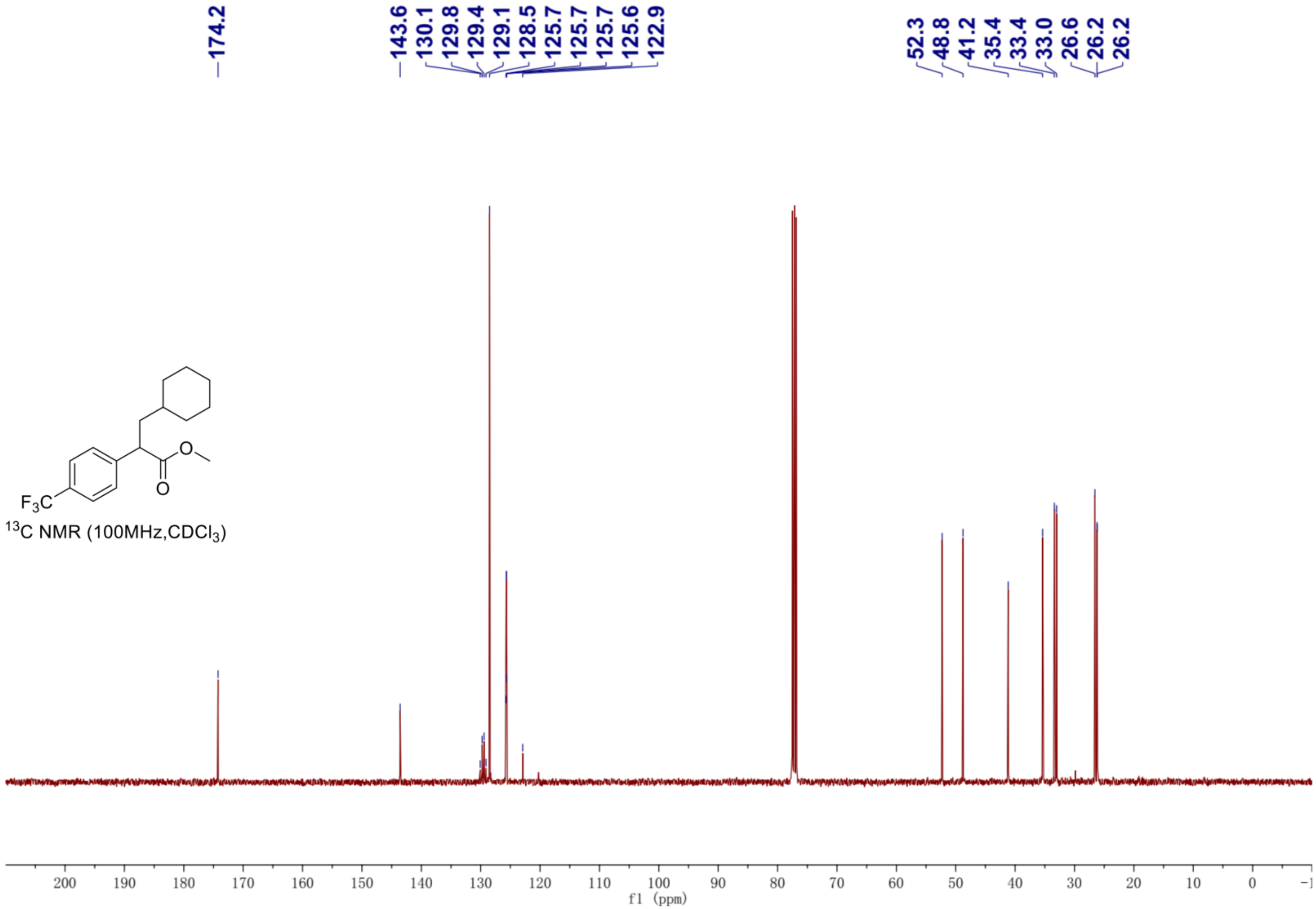

Go to table of contents

Methyl-3-cyclohexyl-2-(4-(trifluoromethyl)phenyl)propanoate (4b)

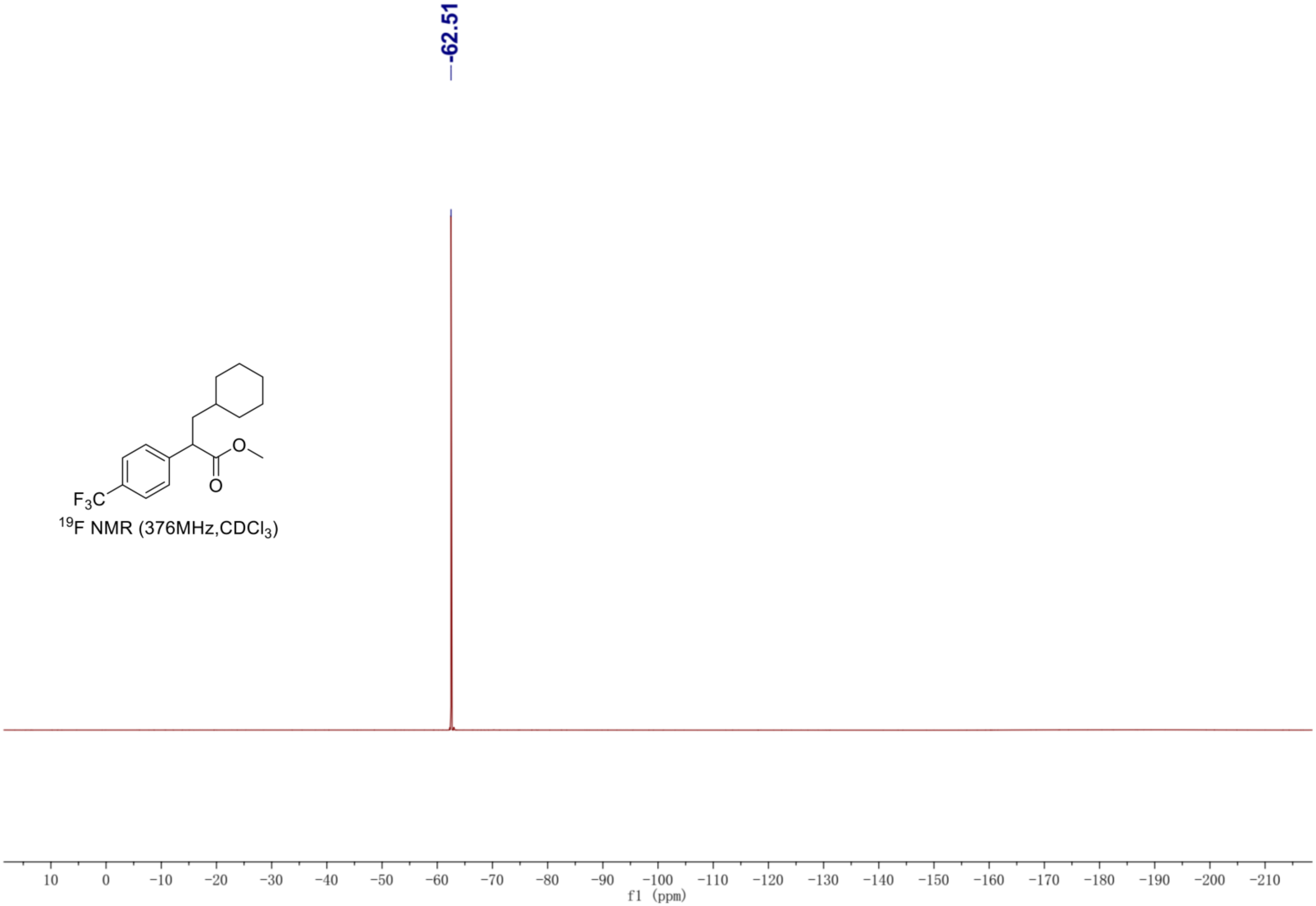

Go to table of contents

Methyl-3-cyclohexyl-2-(4-fluorophenyl)propanoate (4b)

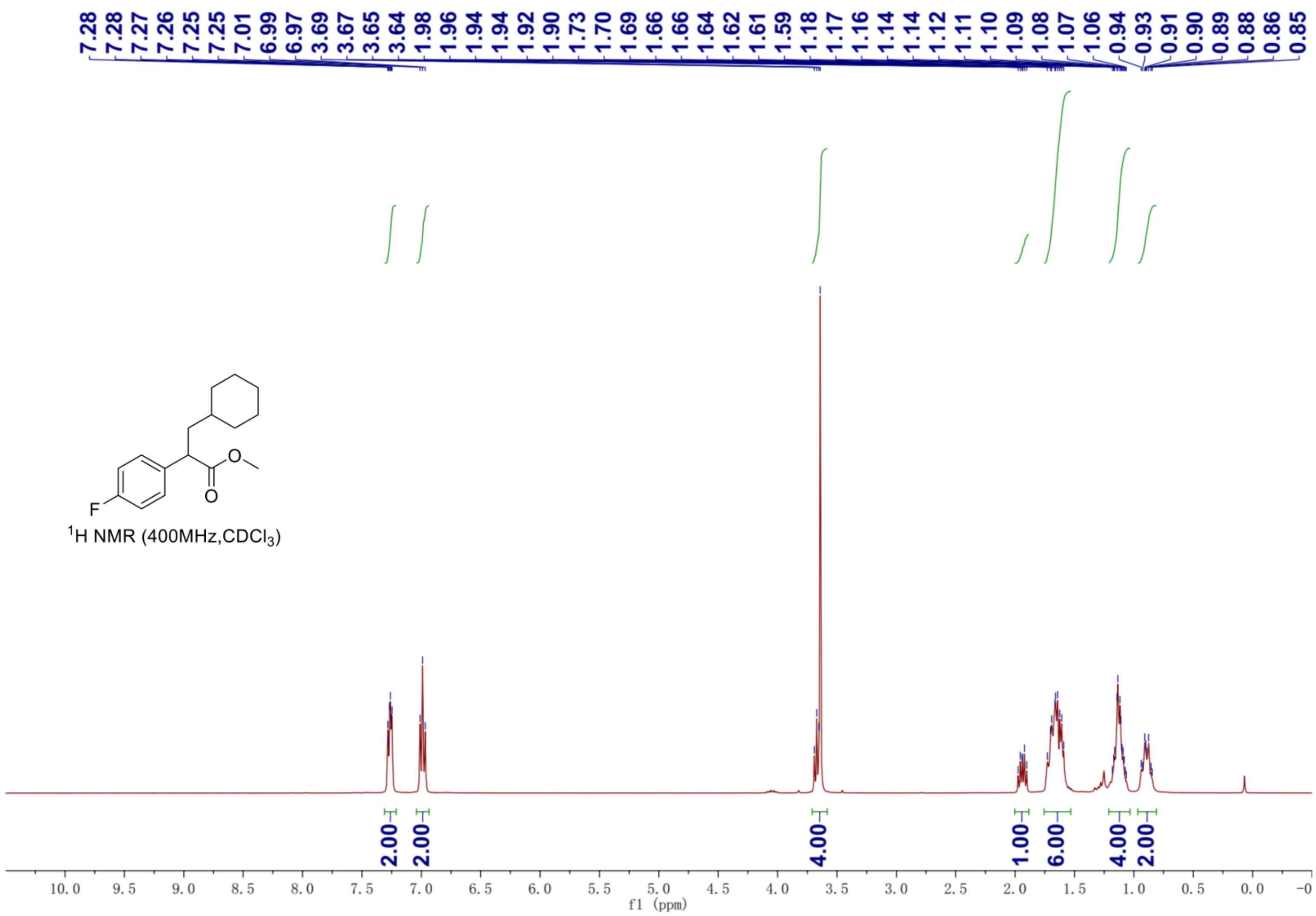

Go to table of contents

Methyl-3-cyclohexyl-2-(4-fluorophenyl)propanoate (4b)

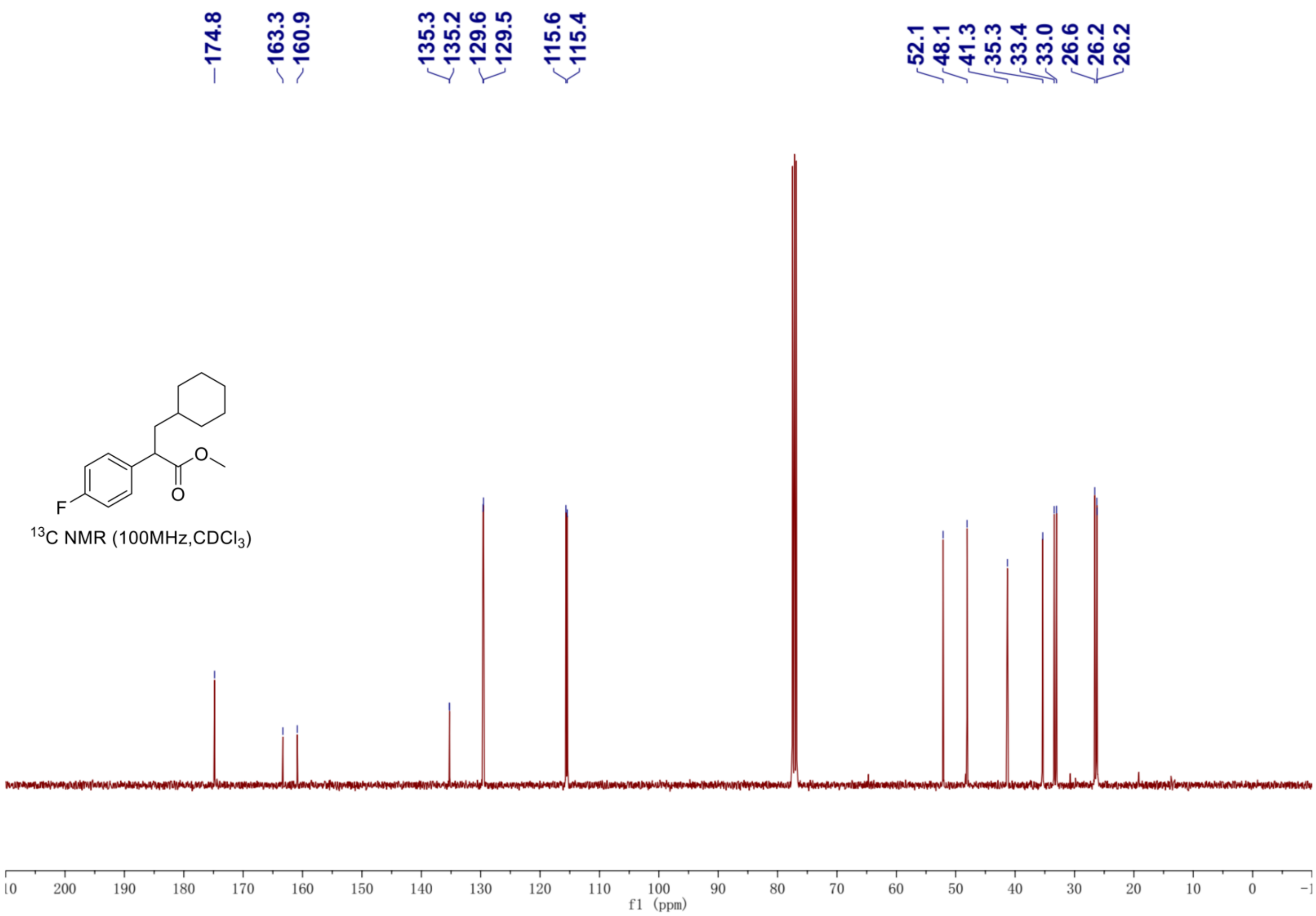

Go to table of contents

Methyl-3-cyclohexyl-2-(4-fluorophenyl)propanoate (4b)

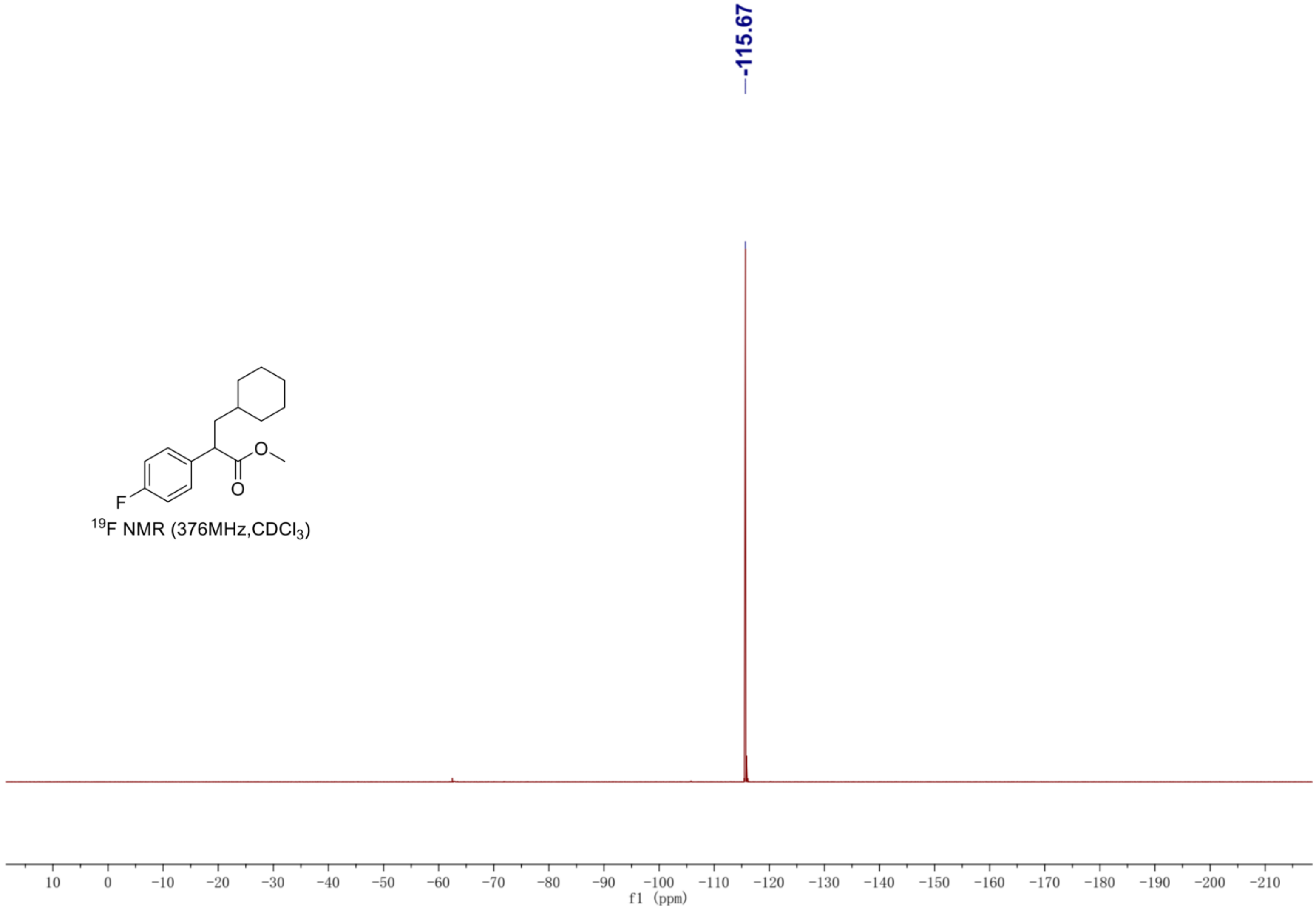

Go to table of contents

Triethyl-2-cyclohexylethane-1,1,2-tricarboxylate (4c)

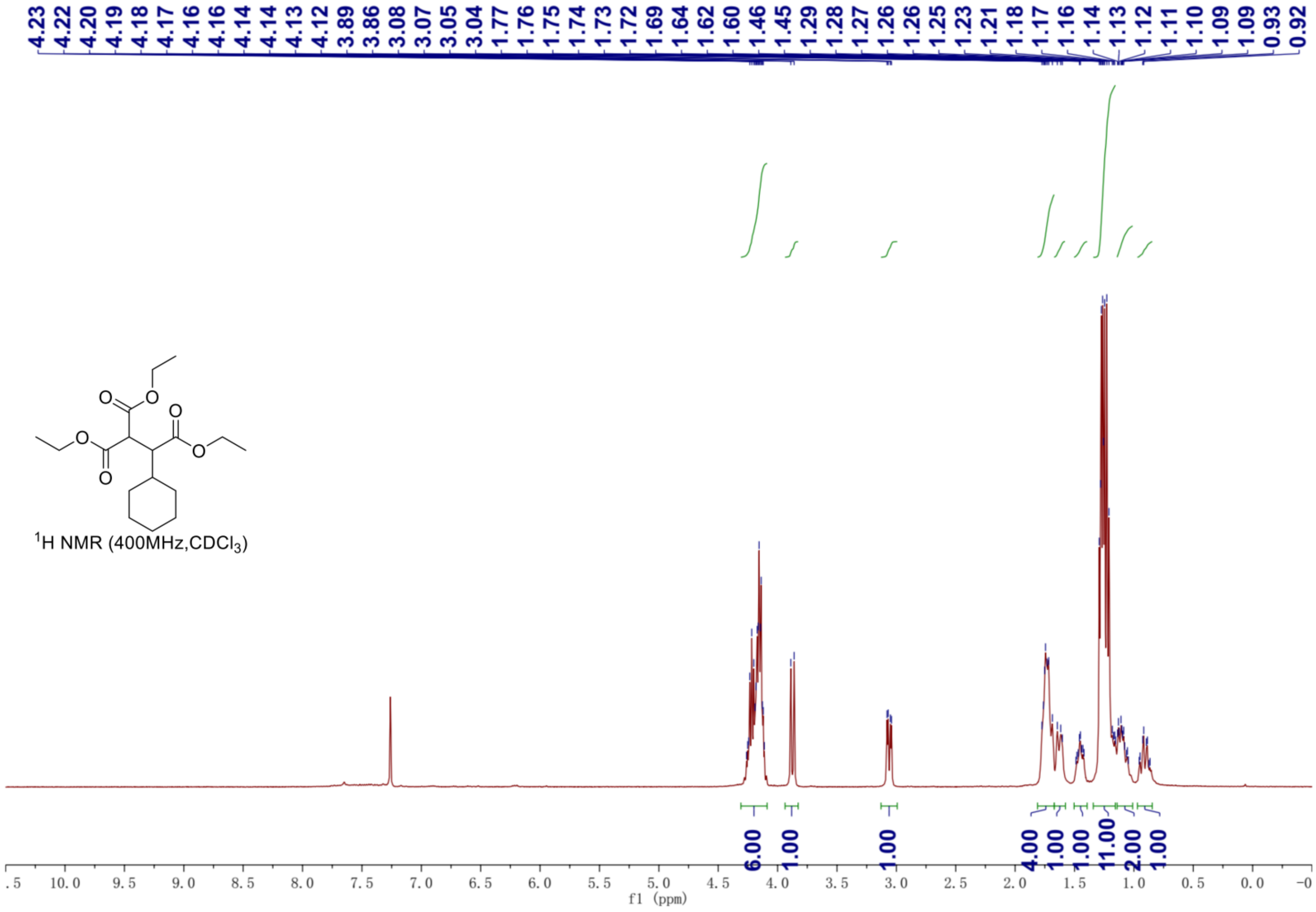

Go to table of contents

Triethyl-2-cyclohexylethane-1,1,2-tricarboxylate (4c)

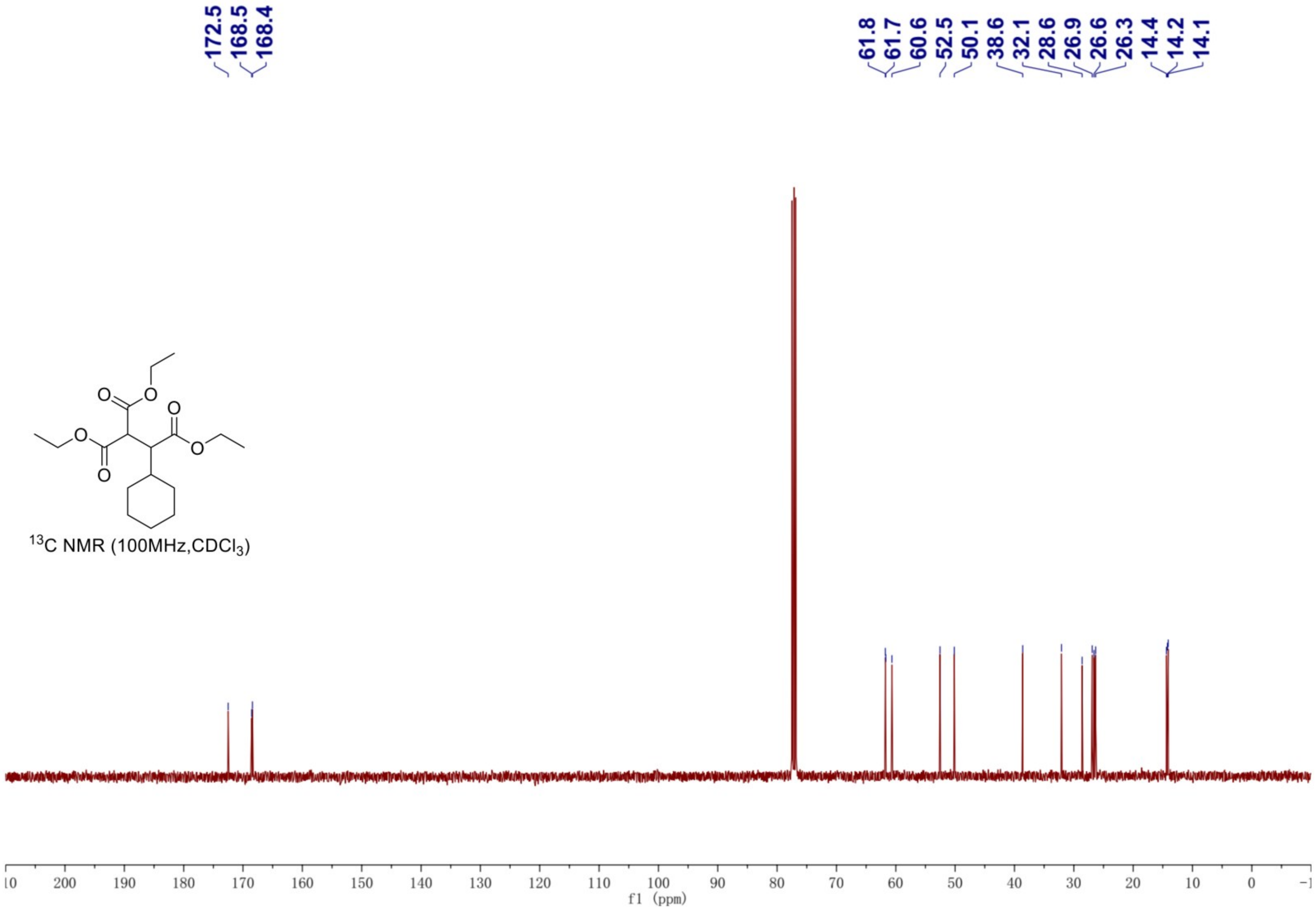

Go to table of contents

2,2-Dimethyl-5-(phenyl(tetrahydrothiophen-2-yl)methyl)-1,3-dioxane-4,6-dione (4d)

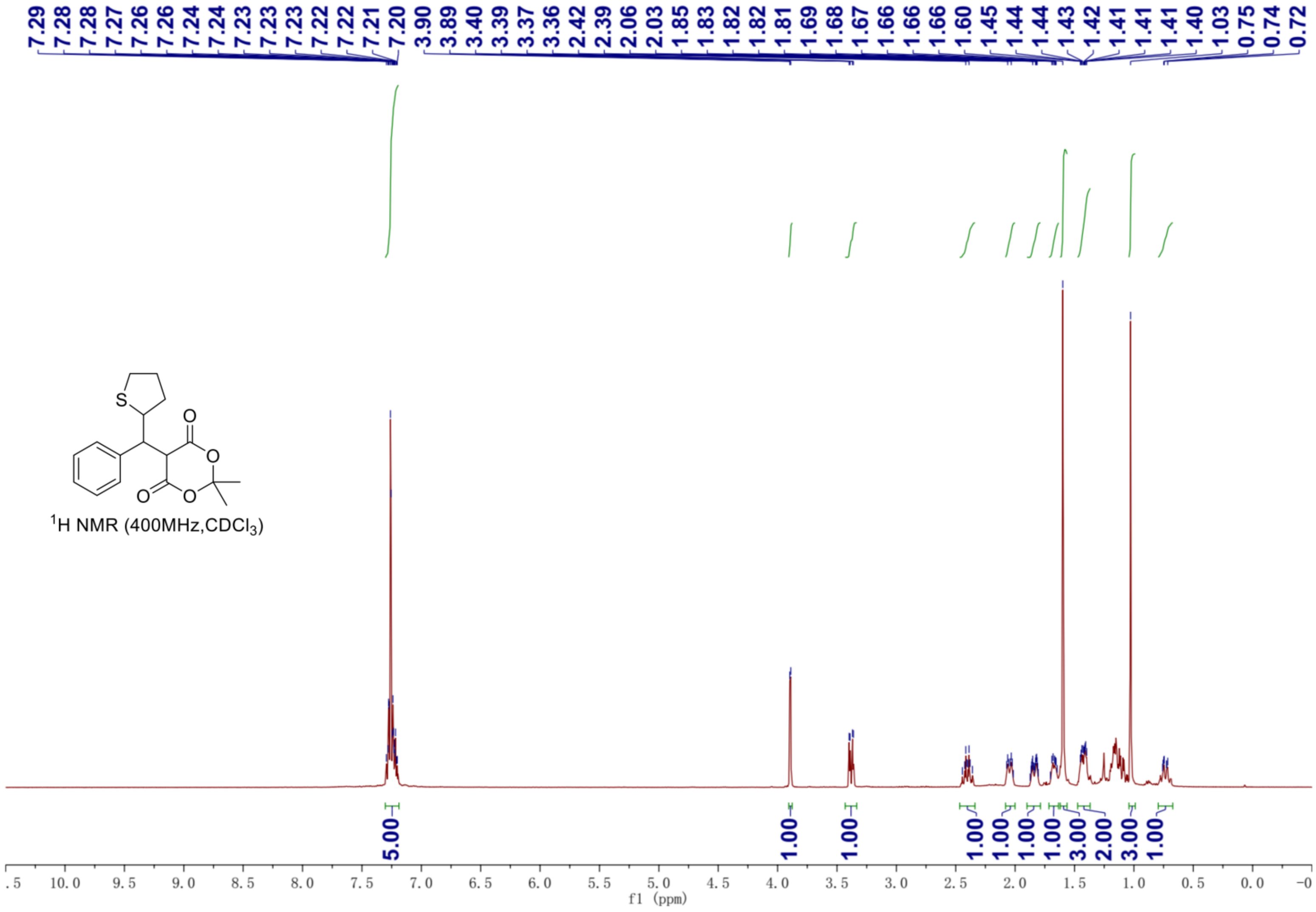

Go to table of contents

2,2-Dimethyl-5-(phenyl(tetrahydrothiophen-2-yl)methyl)-1,3-dioxane-4,6-dione (4d)

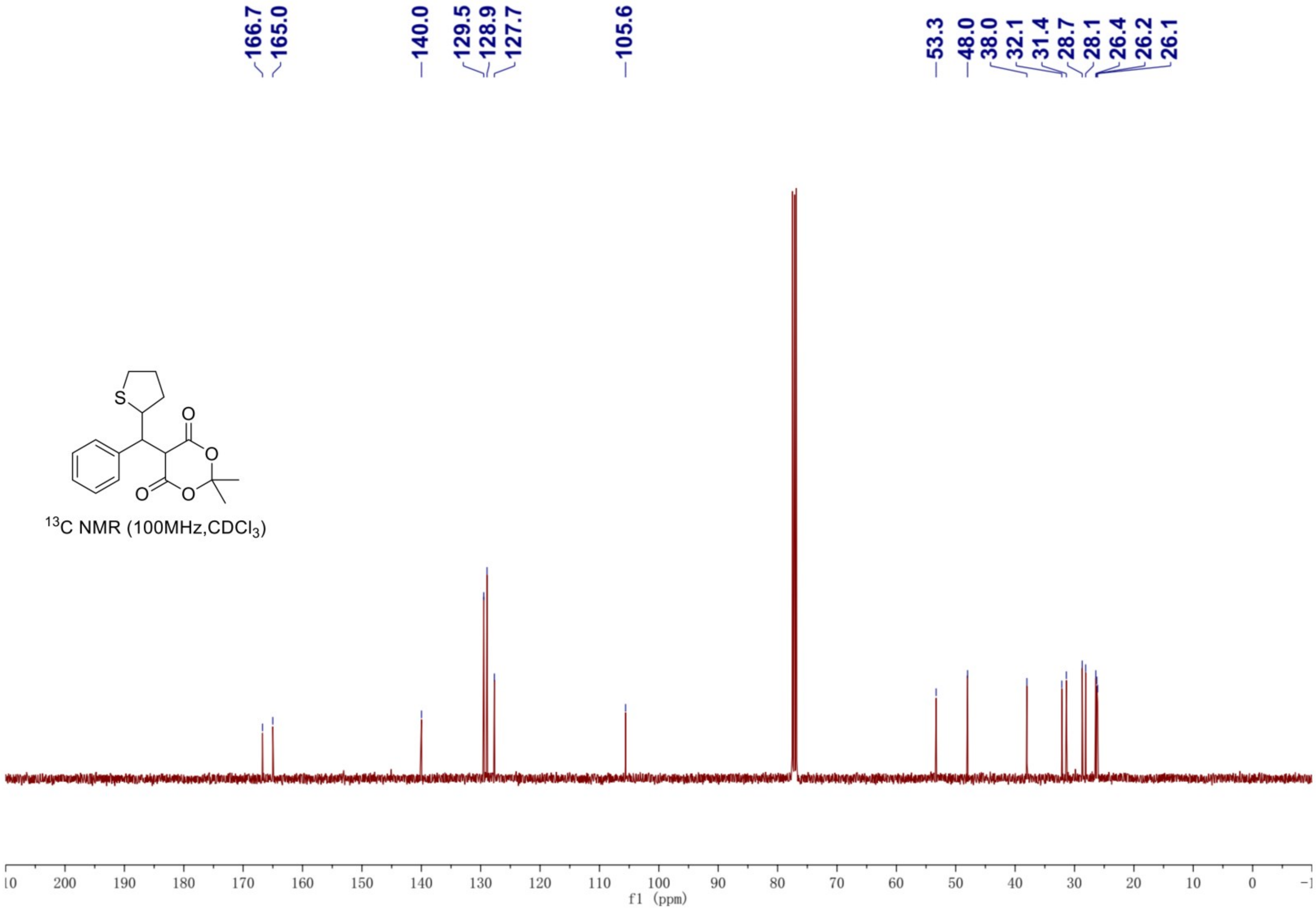

^{13}C NMR (100MHz,CDCl$_3$)

Go to table of contents

3-Cyclohexyl-*N*,2-diphenylpropanamide (4e)

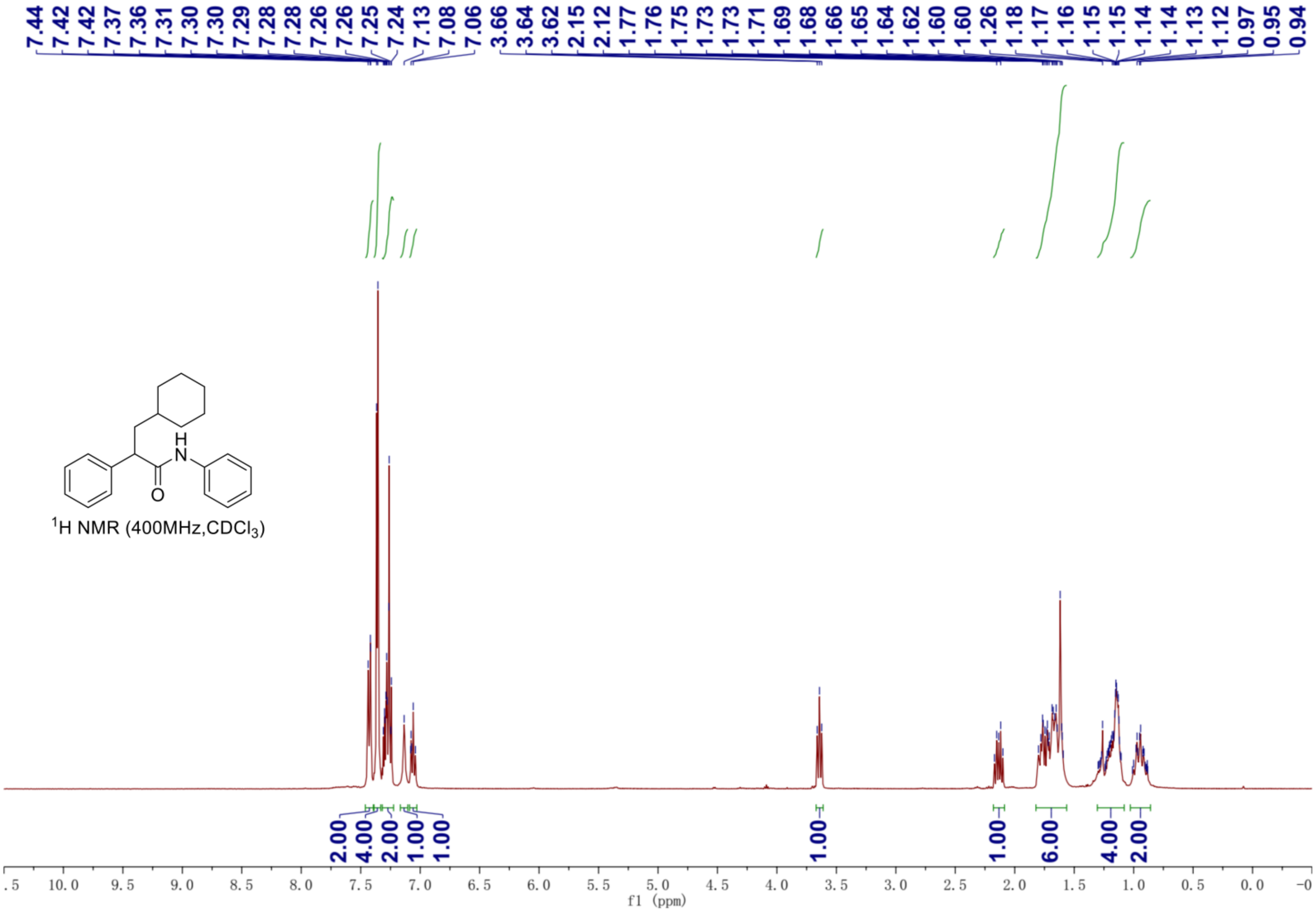

Go to table of contents

3-Cyclohexyl-*N*,2-diphenylpropanamide (4e)

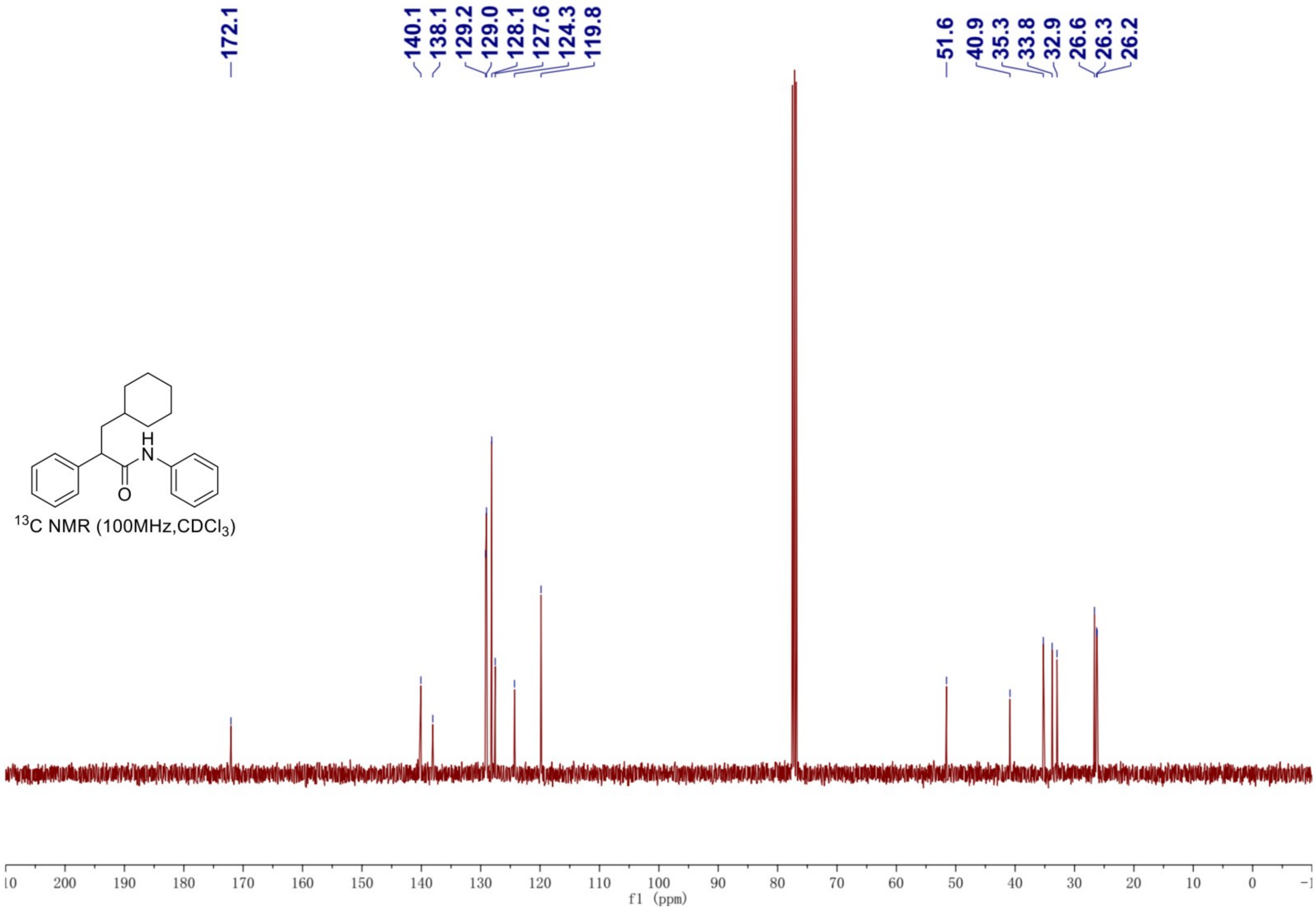

Go to table of contents

2-((4-Phenoxyphenyl)(tetrahydrothiophen-2-yl)methyl)malononitrile (4f)

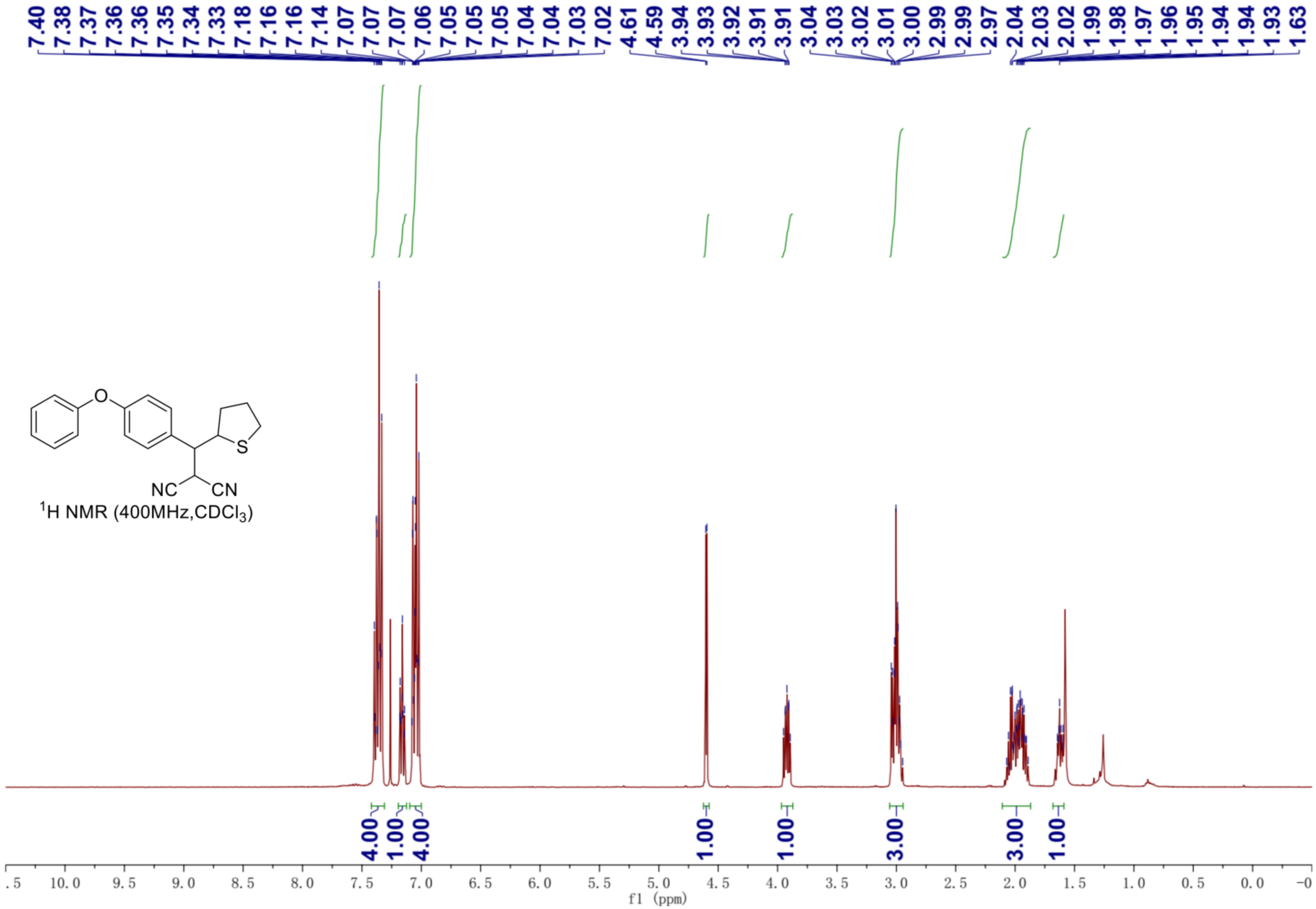

Go to table of contents

2-((4-Phenoxyphenyl)(tetrahydrothiophen-2-yl)methyl)malononitrile (4f)

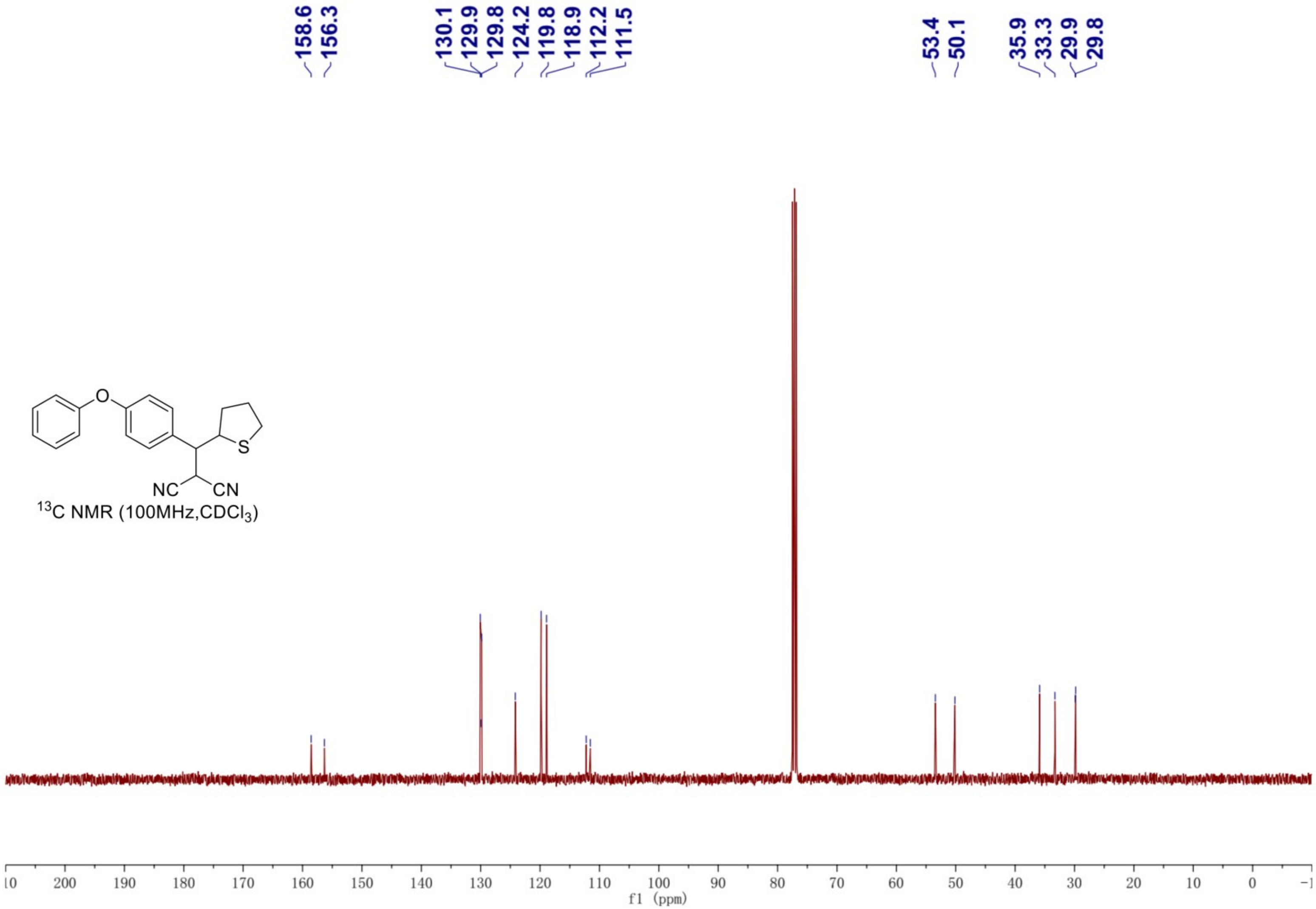

Go to table of contents

2-(4-Methyl-2-oxo-1-phenylpentyl)malononitrile (4g)

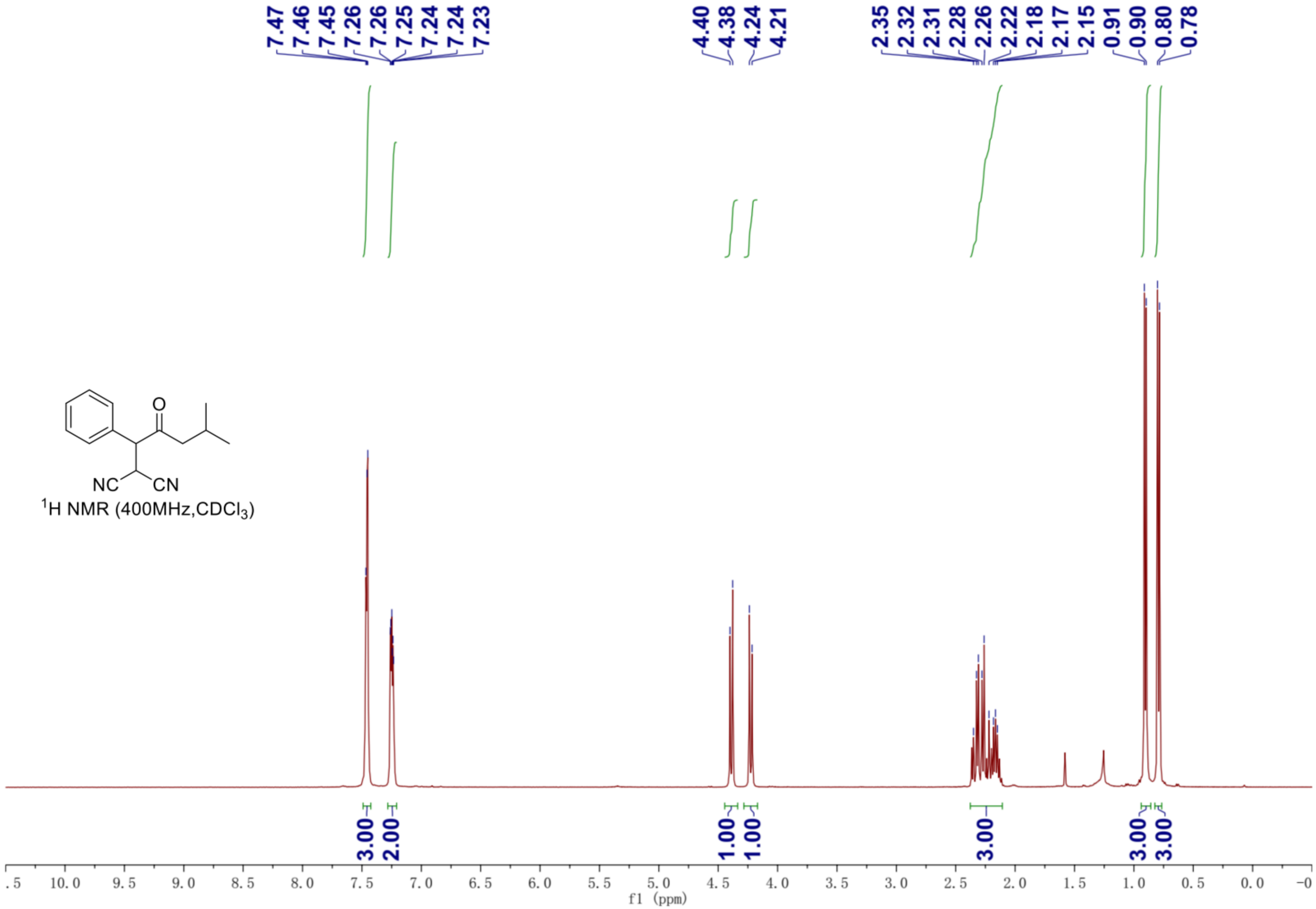

Go to table of contents

2-(4-Methyl-2-oxo-1-phenylpentyl)malononitrile (4g)

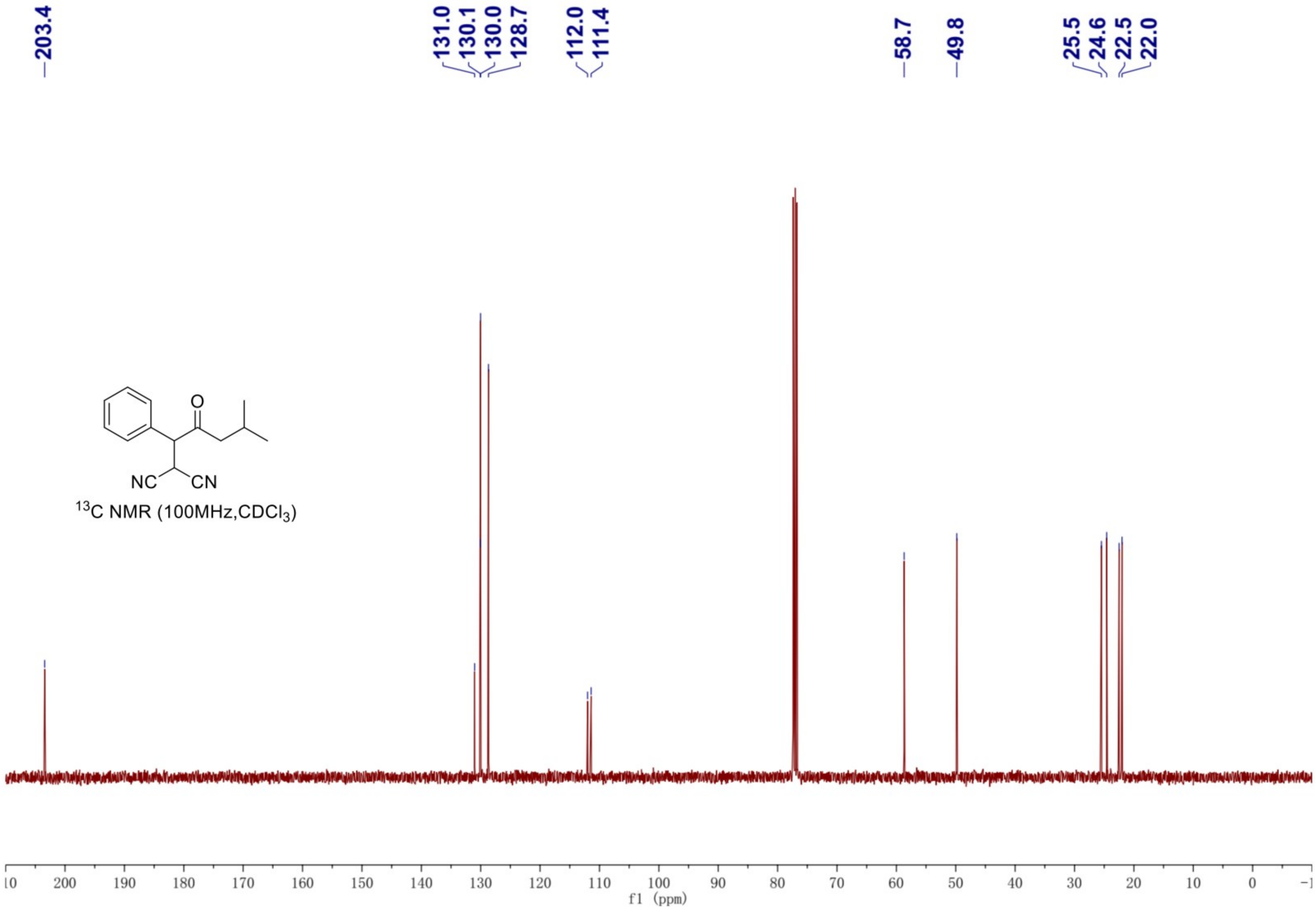

Go to table of contents

N-(3,3-Dicyano-2-(4-fluorophenyl)propyl)-N-methylacetamide (4h)

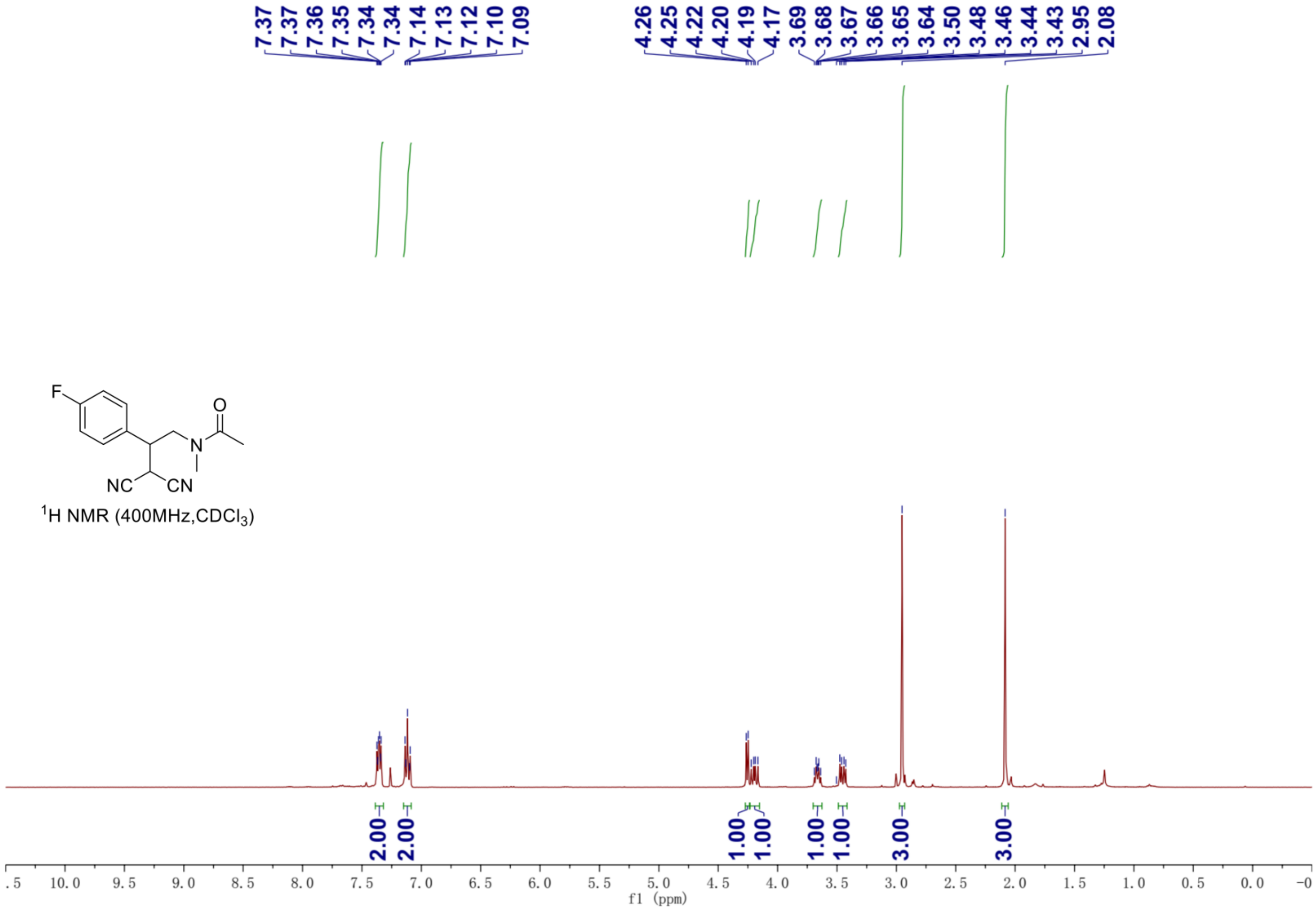

Go to table of contents

N-(3,3-Dicyano-2-(4-fluorophenyl)propyl)-N-methylacetamide (4h)

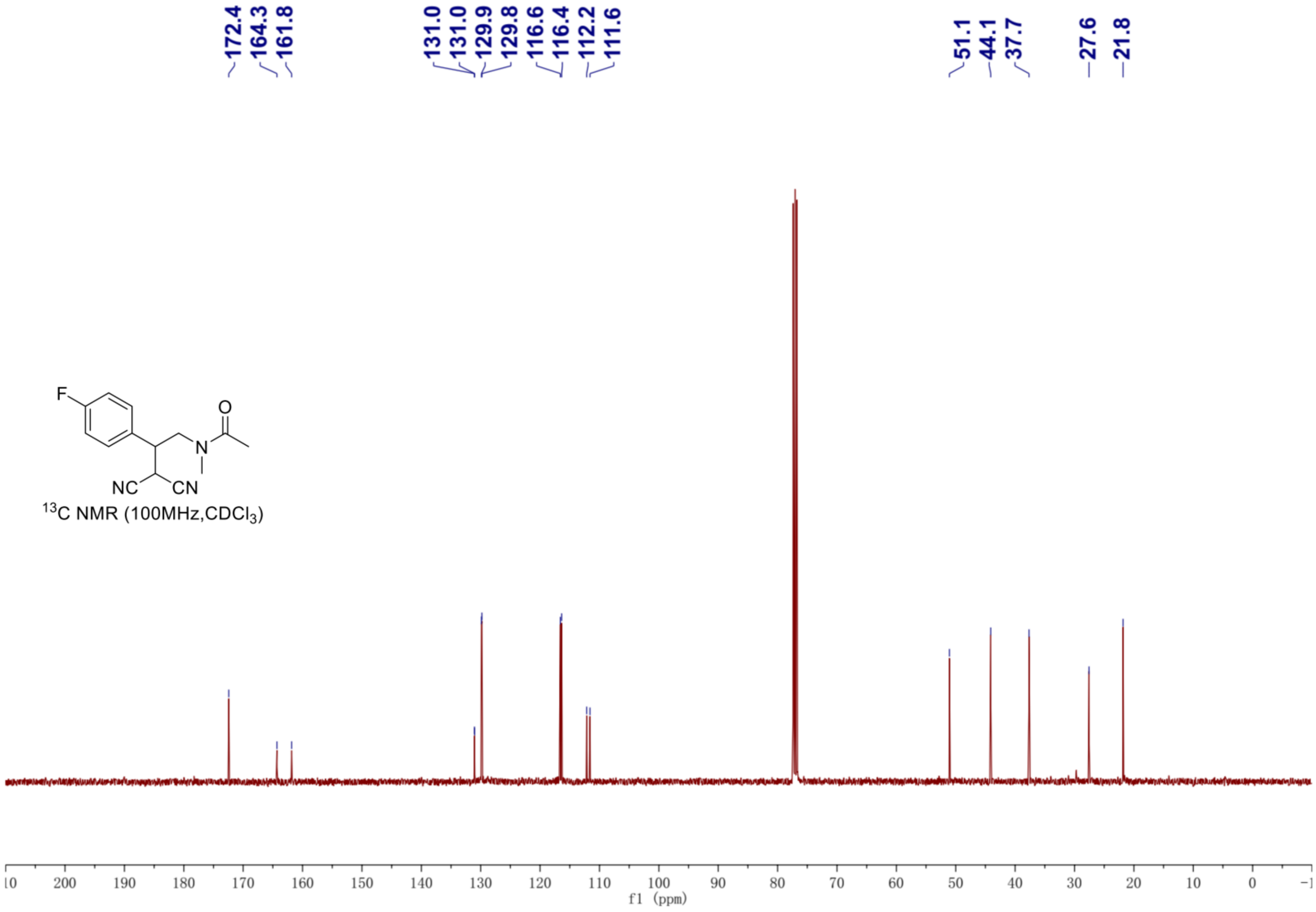

Go to table of contents

N-(3,3-Dicyano-2-(4-fluorophenyl)propyl)-N-methylacetamide (4h)

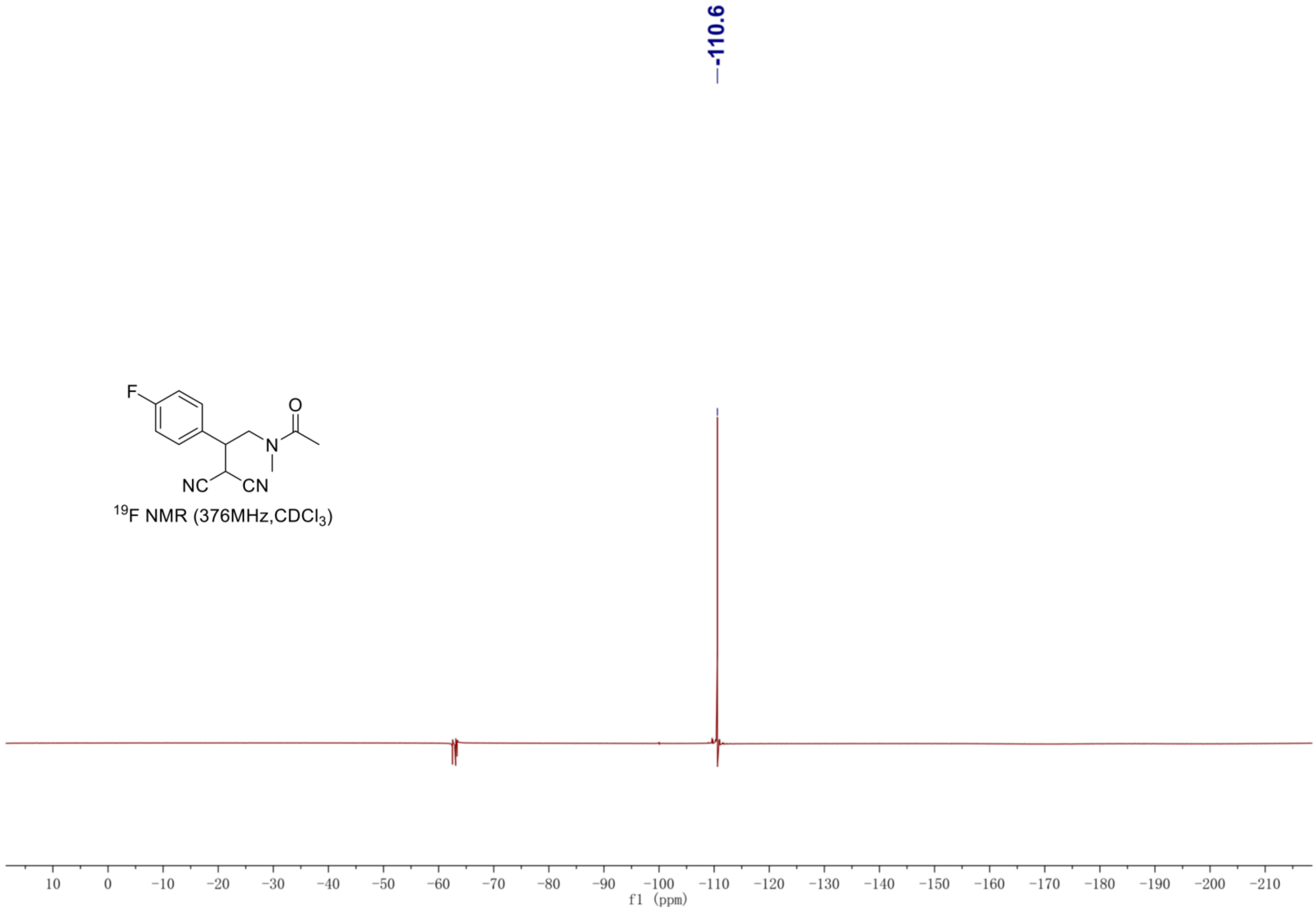

Go to table of contents

2-Amino-4-(4-fluorophenyl)-5,5-dimethyl-4,5-dihydrofuran-3-carbonitrile (4j)

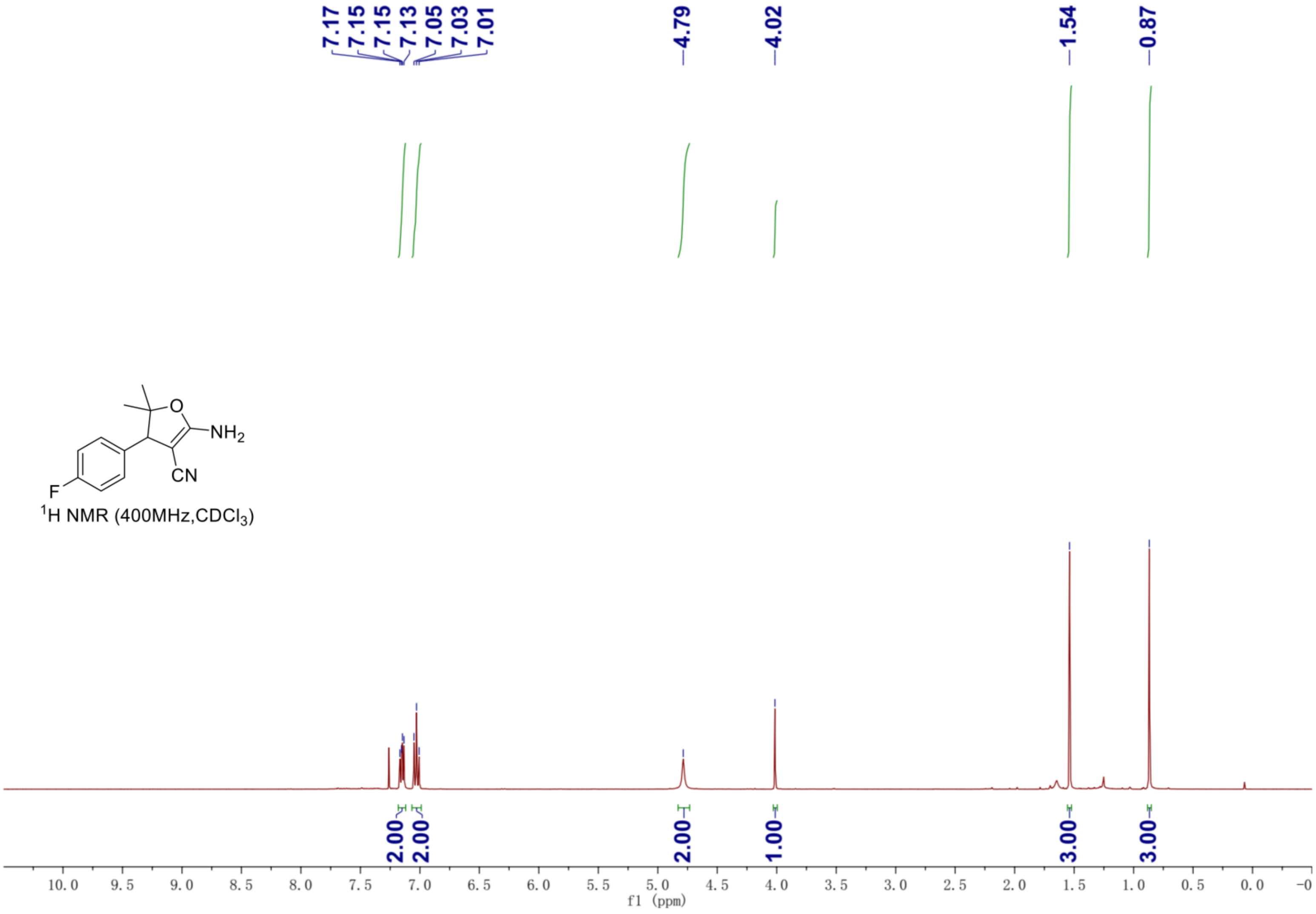

Go to table of contents

2-Amino-4-(4-fluorophenyl)-5,5-dimethyl-4,5-dihydrofuran-3-carbonitrile (4j)

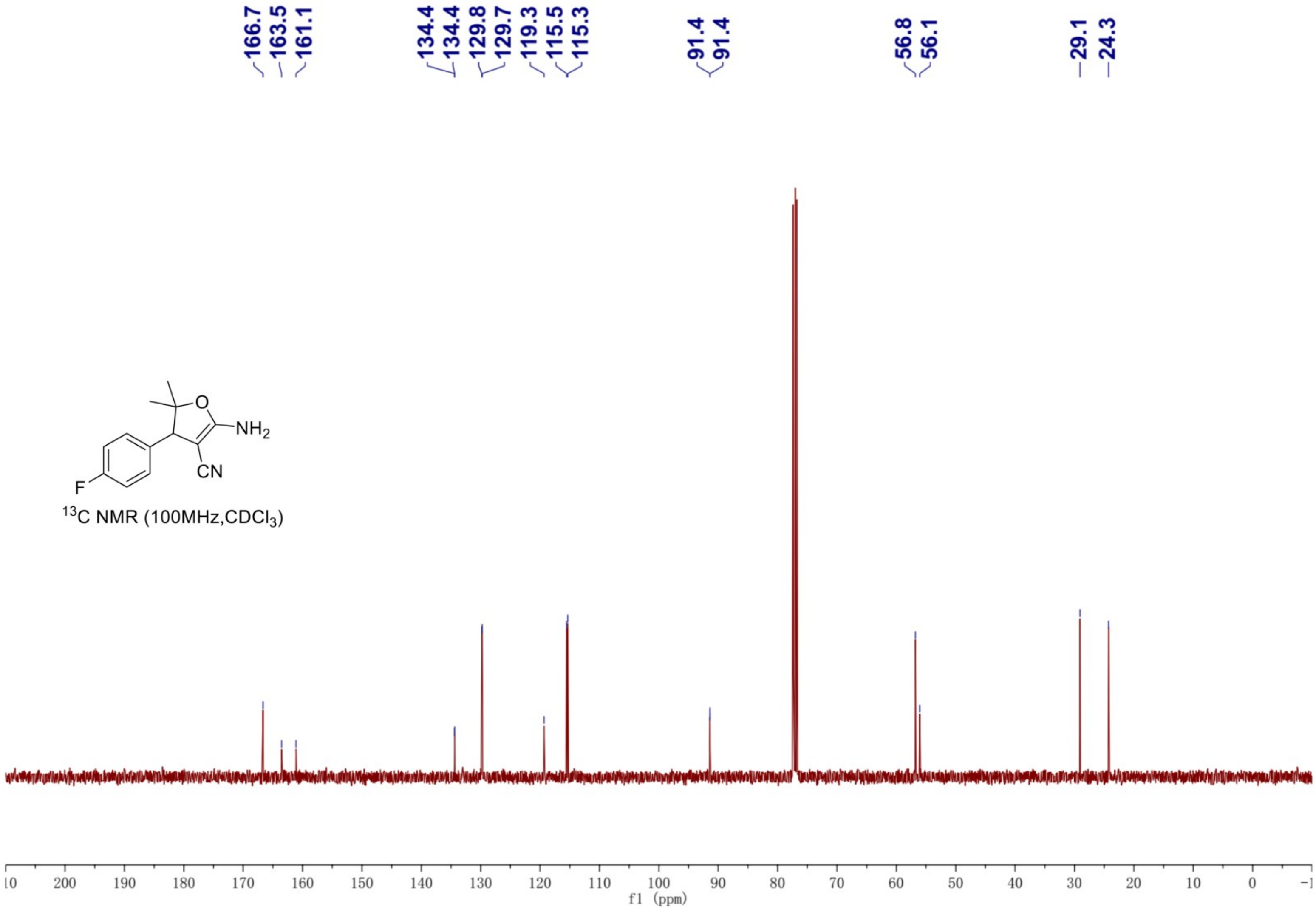

Go to table of contents

2-Amino-4-(4-fluorophenyl)-5,5-dimethyl-4,5-dihydrofuran-3-carbonitrile (4j)

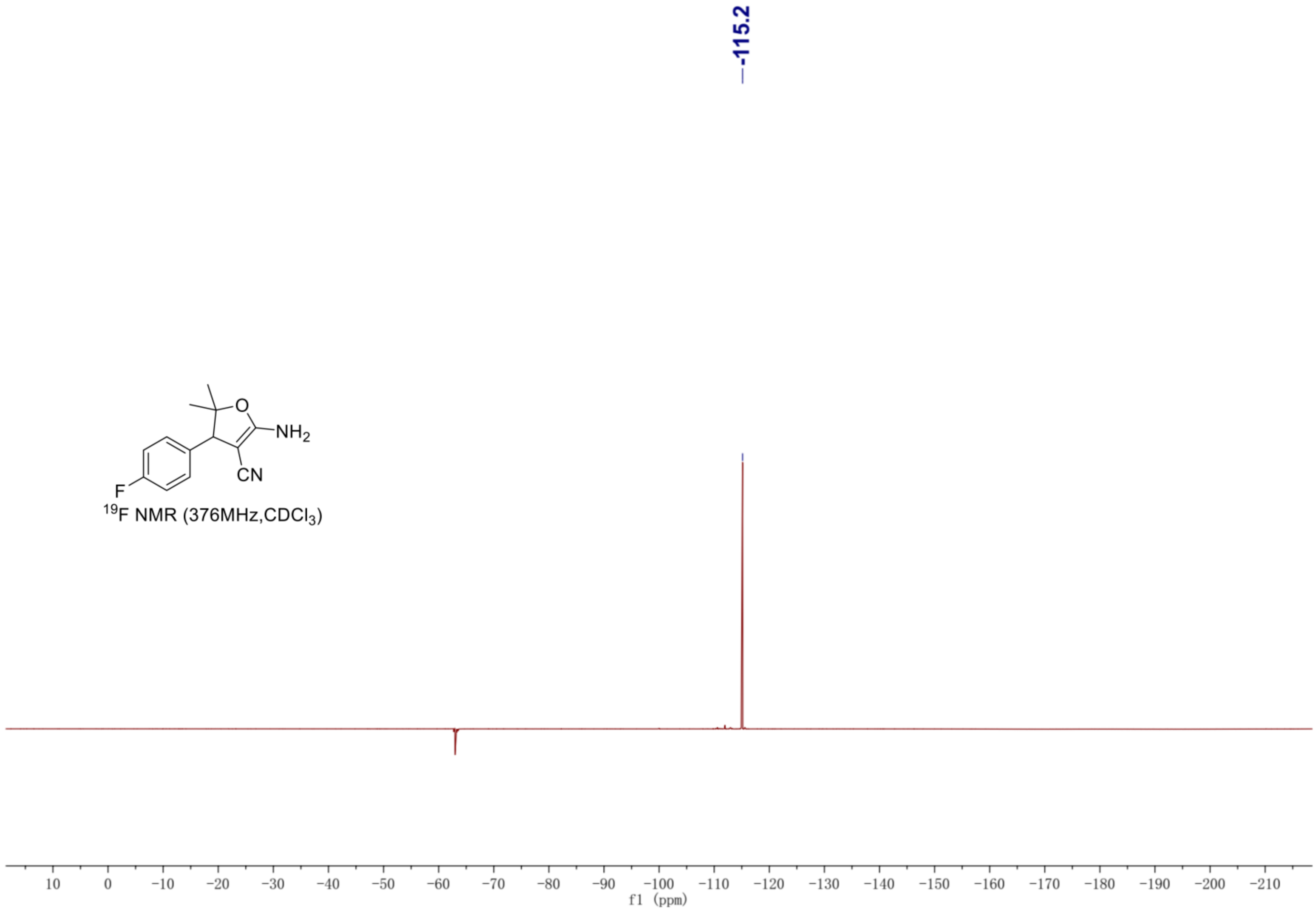

Go to table of contents

Di-*tert*-butyl 1-(tetrahydrofuran-2-yl)hydrazine-1,2-dicarboxylate (4k)

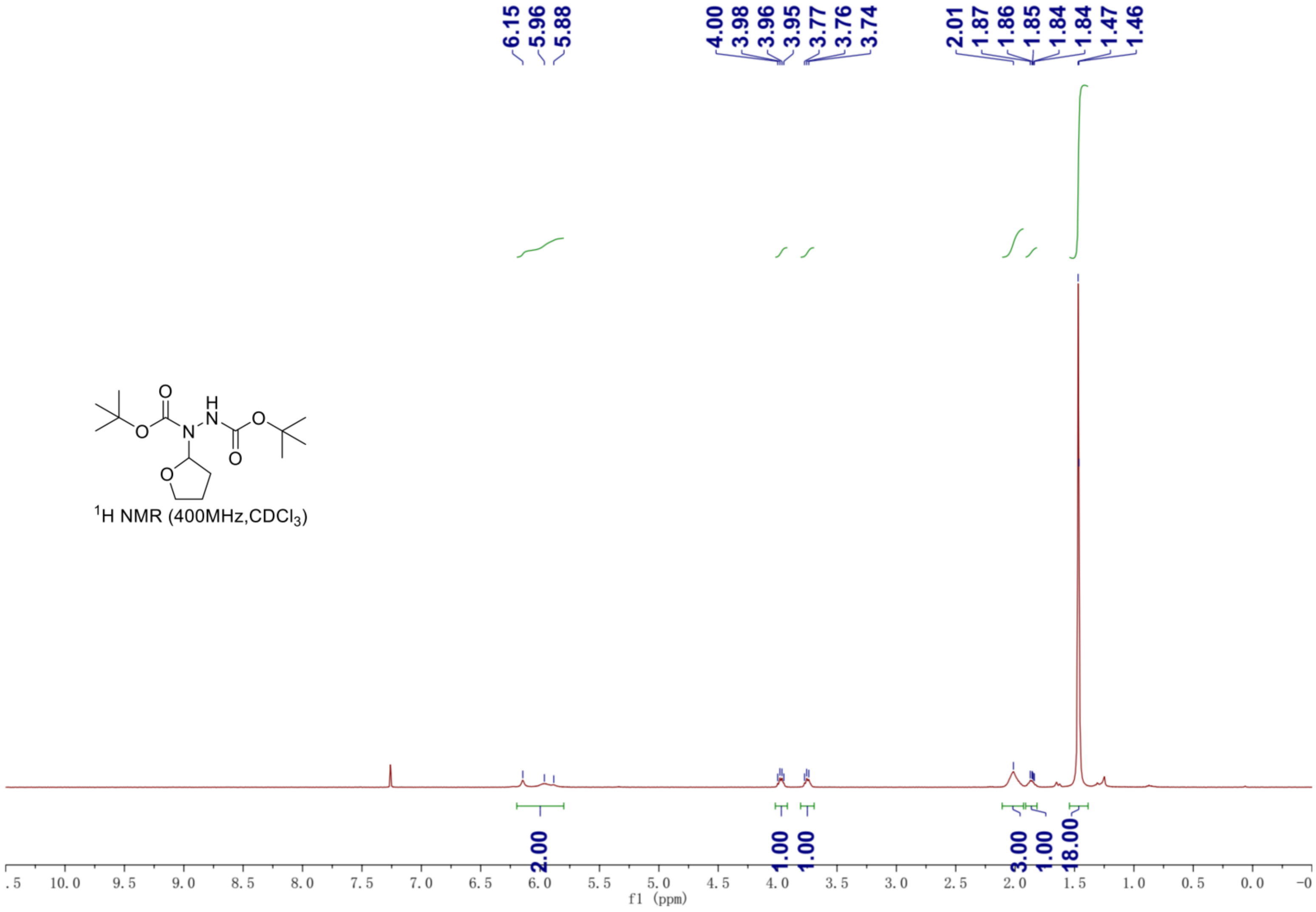

Go to table of contents

Di-*tert*-butyl 1-(tetrahydrofuran-2-yl)hydrazine-1,2-dicarboxylate (4k)

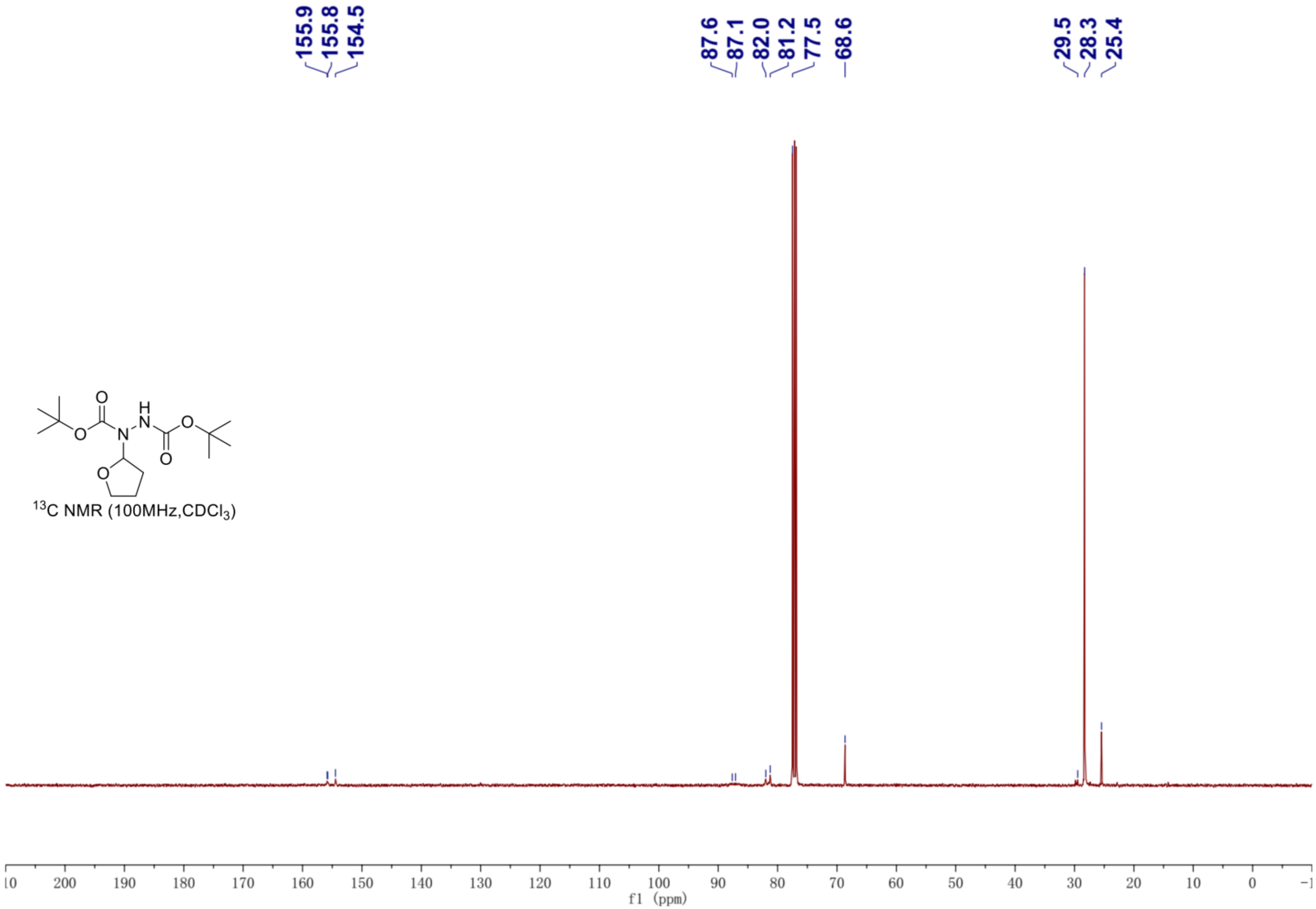

1,2-Diphenyl-1-(tetrahydrothiophen-2-yl)hydrazine (4l)

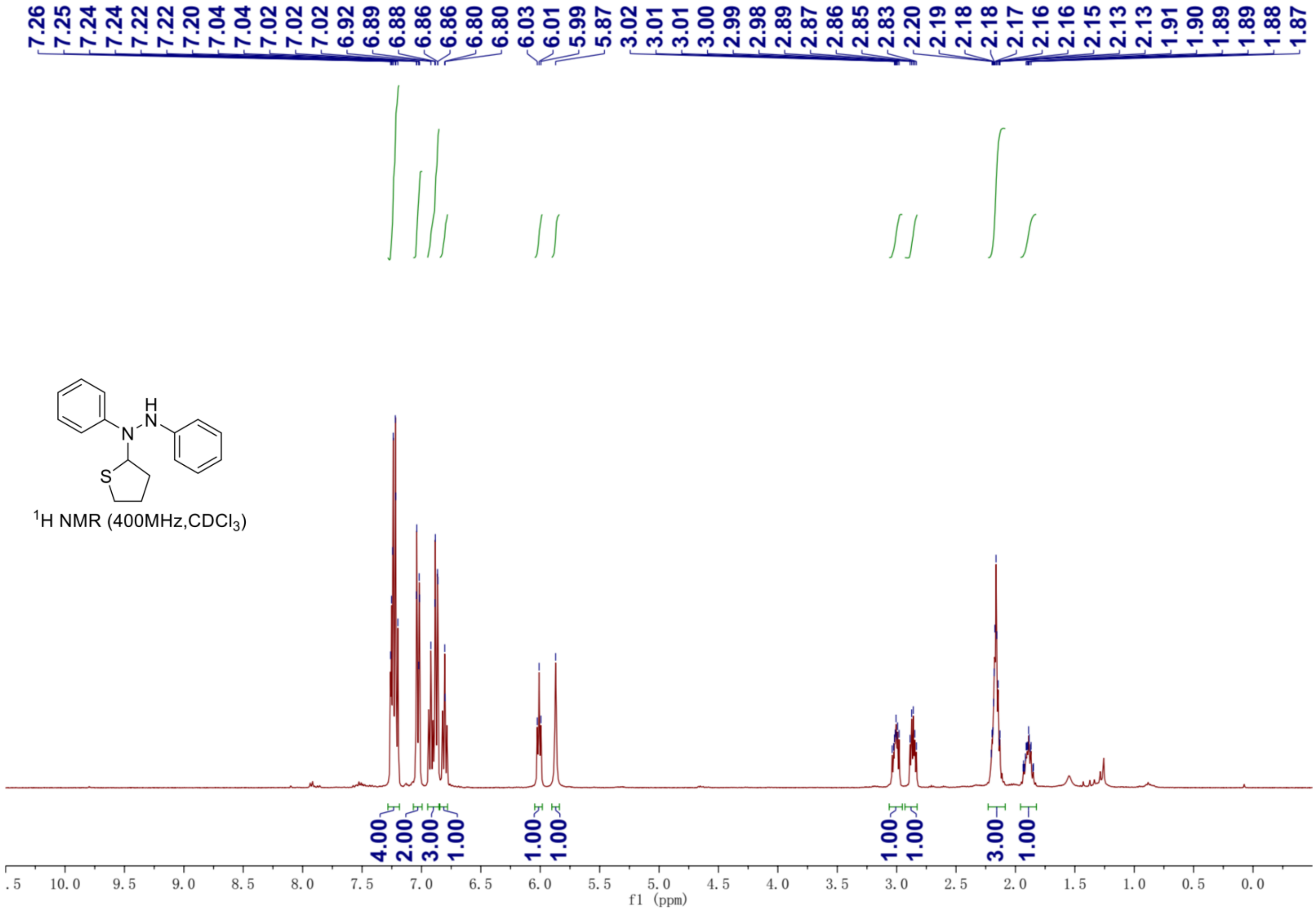

Go to table of contents

1,2-Diphenyl-1-(tetrahydrothiophen-2-yl)hydrazine (4l)

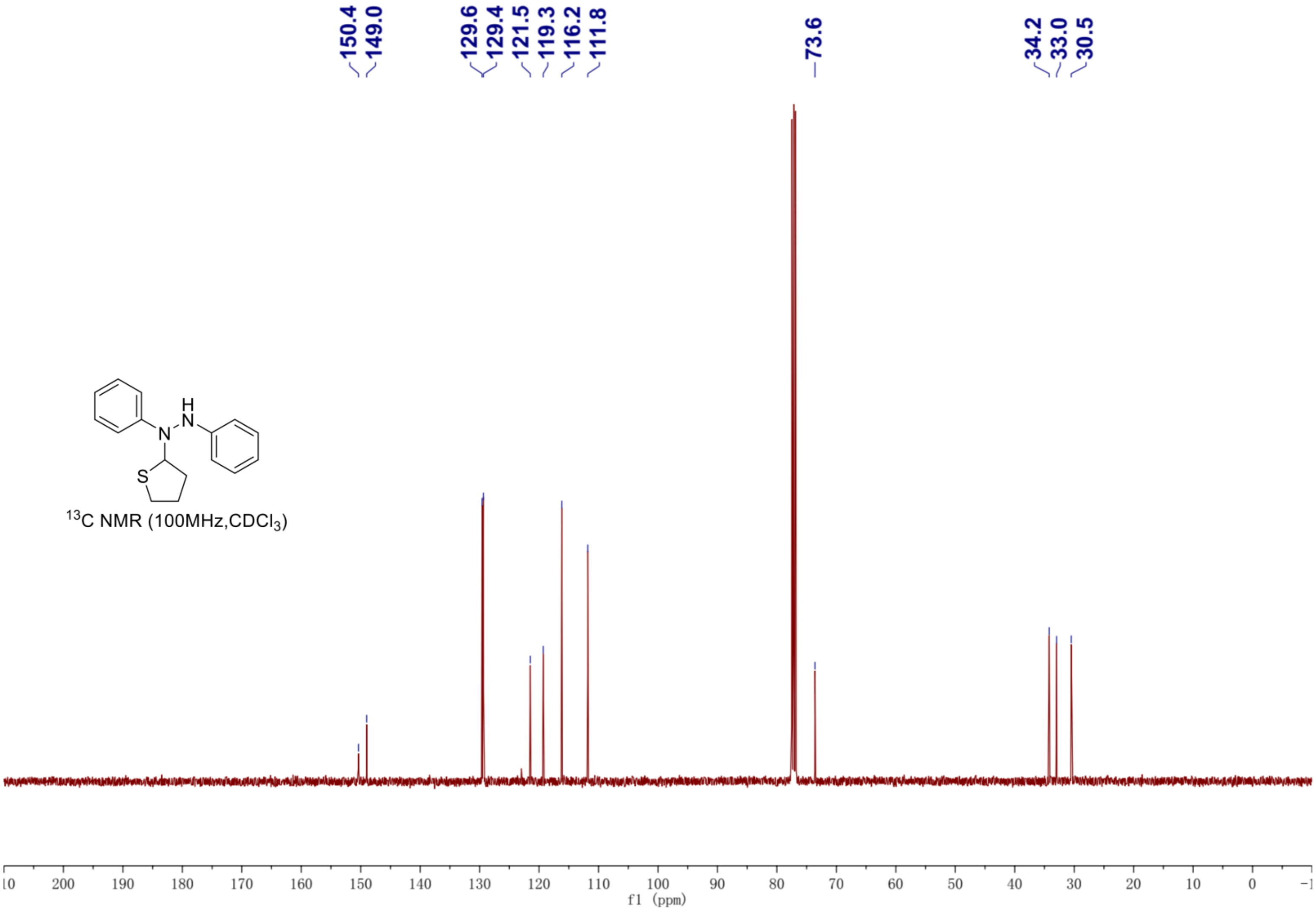

Go to table of contents

1-(Tetrahydrothiophen-2-yl)-1,2-bis(4-(trifluoromethyl)phenyl)hydrazine (4m)

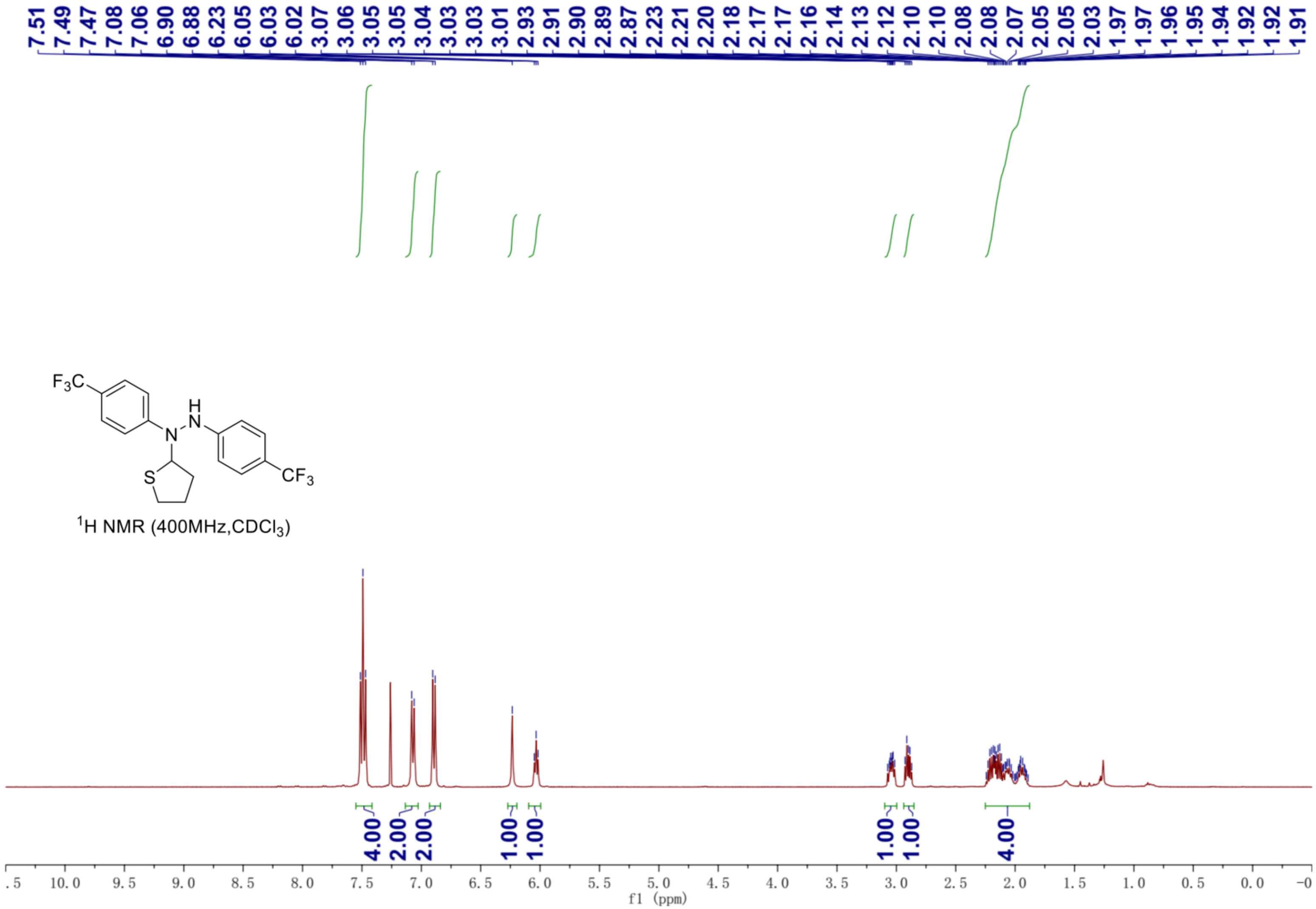

Go to table of contents

1-(Tetrahydrothiophen-2-yl)-1,2-bis(4-(trifluoromethyl)phenyl)hydrazine (4m)

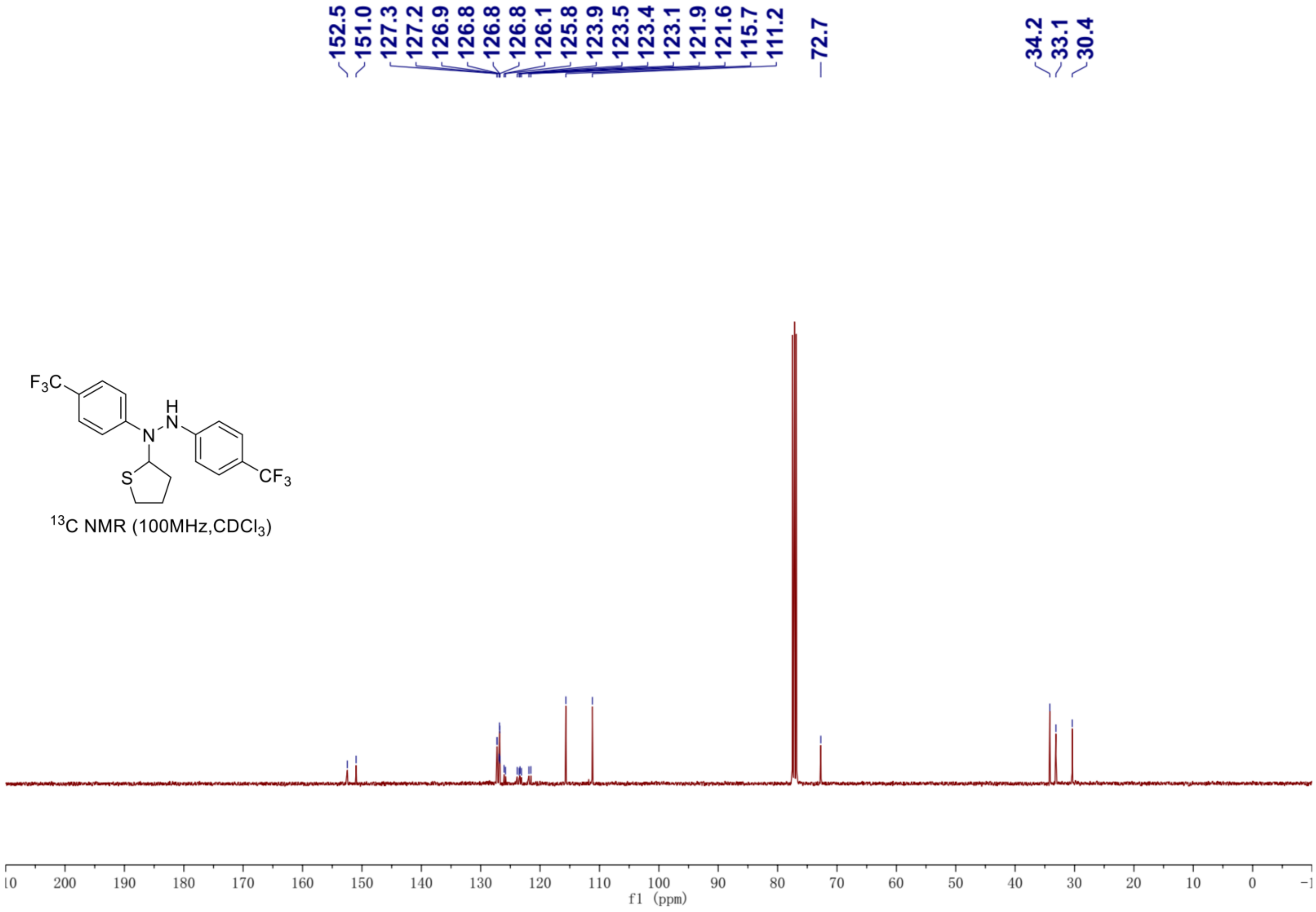

Go to table of contents

1-(Tetrahydrothiophen-2-yl)-1,2-bis(4-(trifluoromethyl)phenyl)hydrazine (4m)

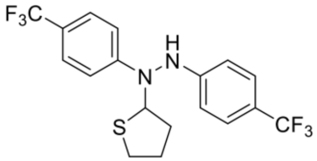

^{19}F NMR (376MHz, $CDCl_3$)

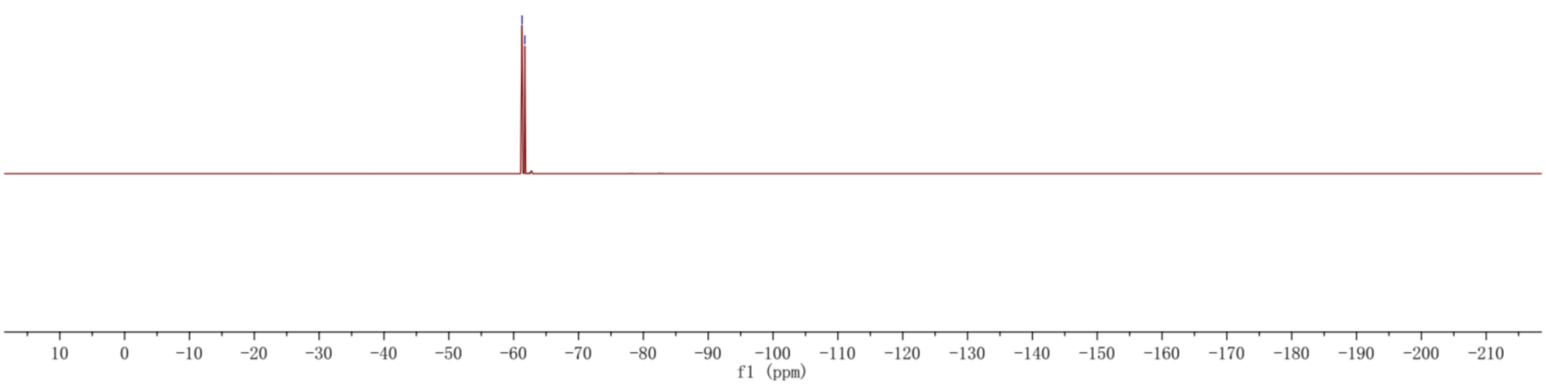

Go to table of contents

Diethyl 1-(tetrahydrothiophen-2-yl)hydrazine-1,2-dicarboxylate (4n)

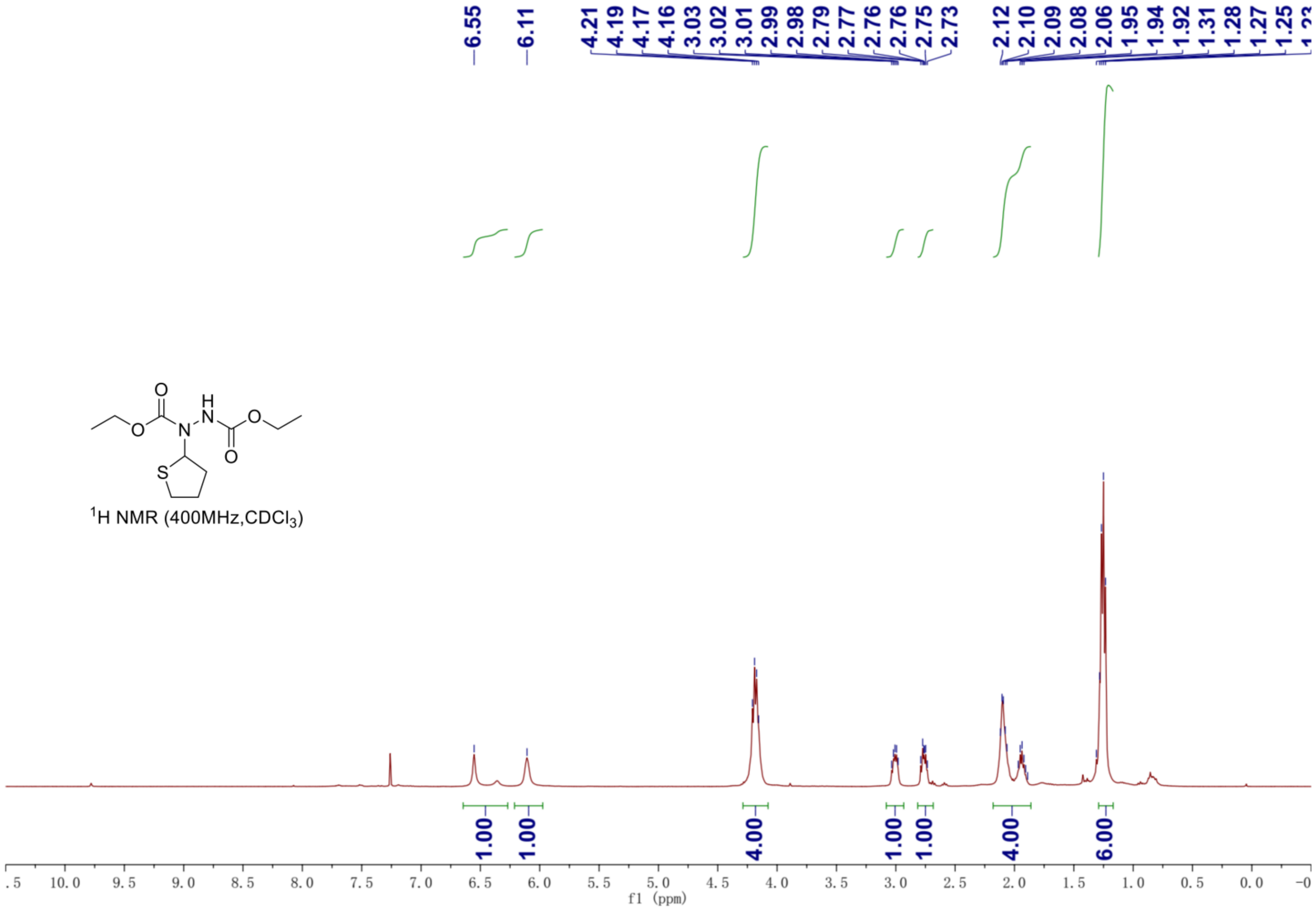

Go to table of contents

Diethyl 1-(tetrahydrothiophen-2-yl)hydrazine-1,2-dicarboxylate (4n)

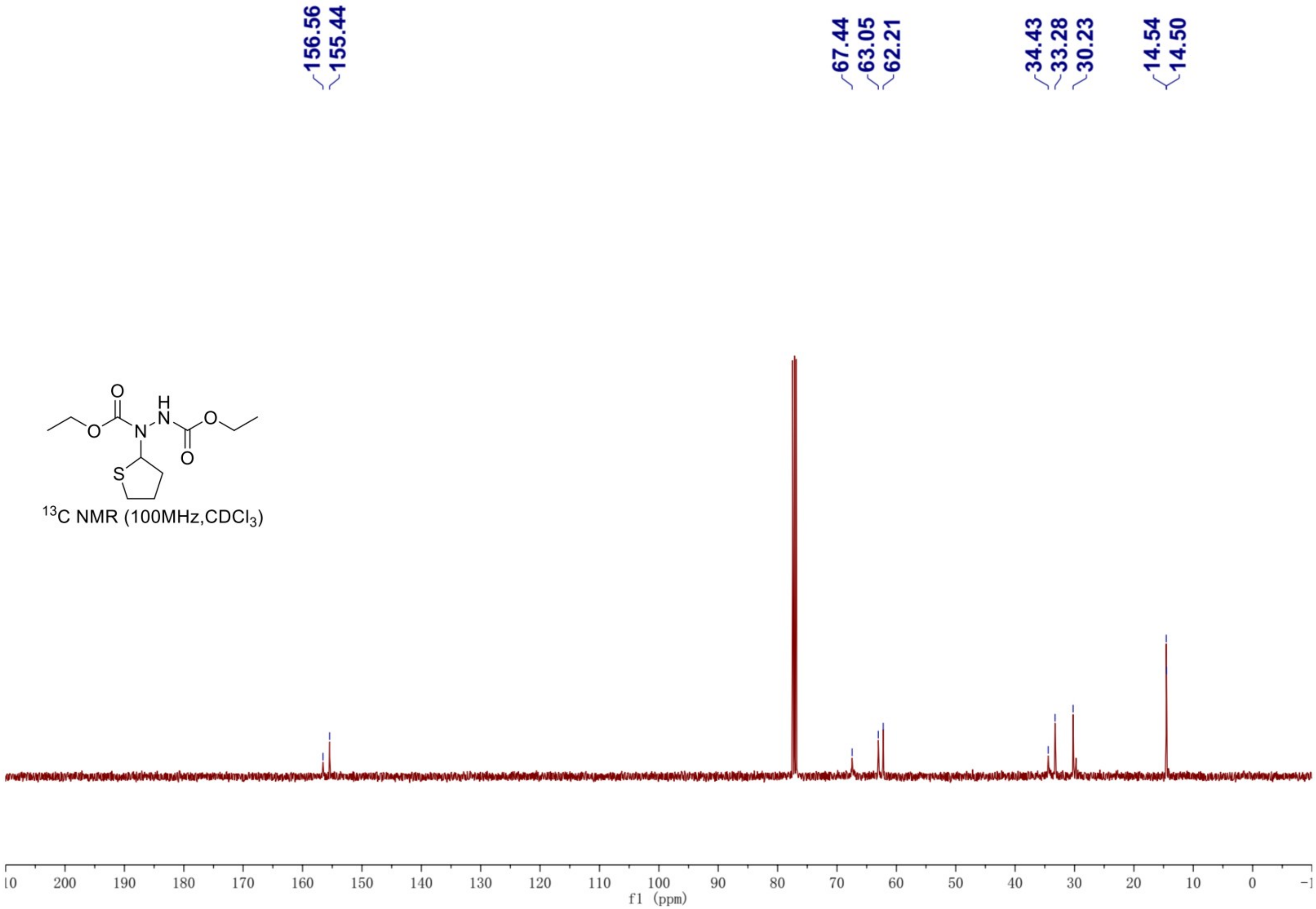

Go to table of contents

Butyl 2-cyclohexyl-2-phenylhydrazine-1-carboxylate (4o)

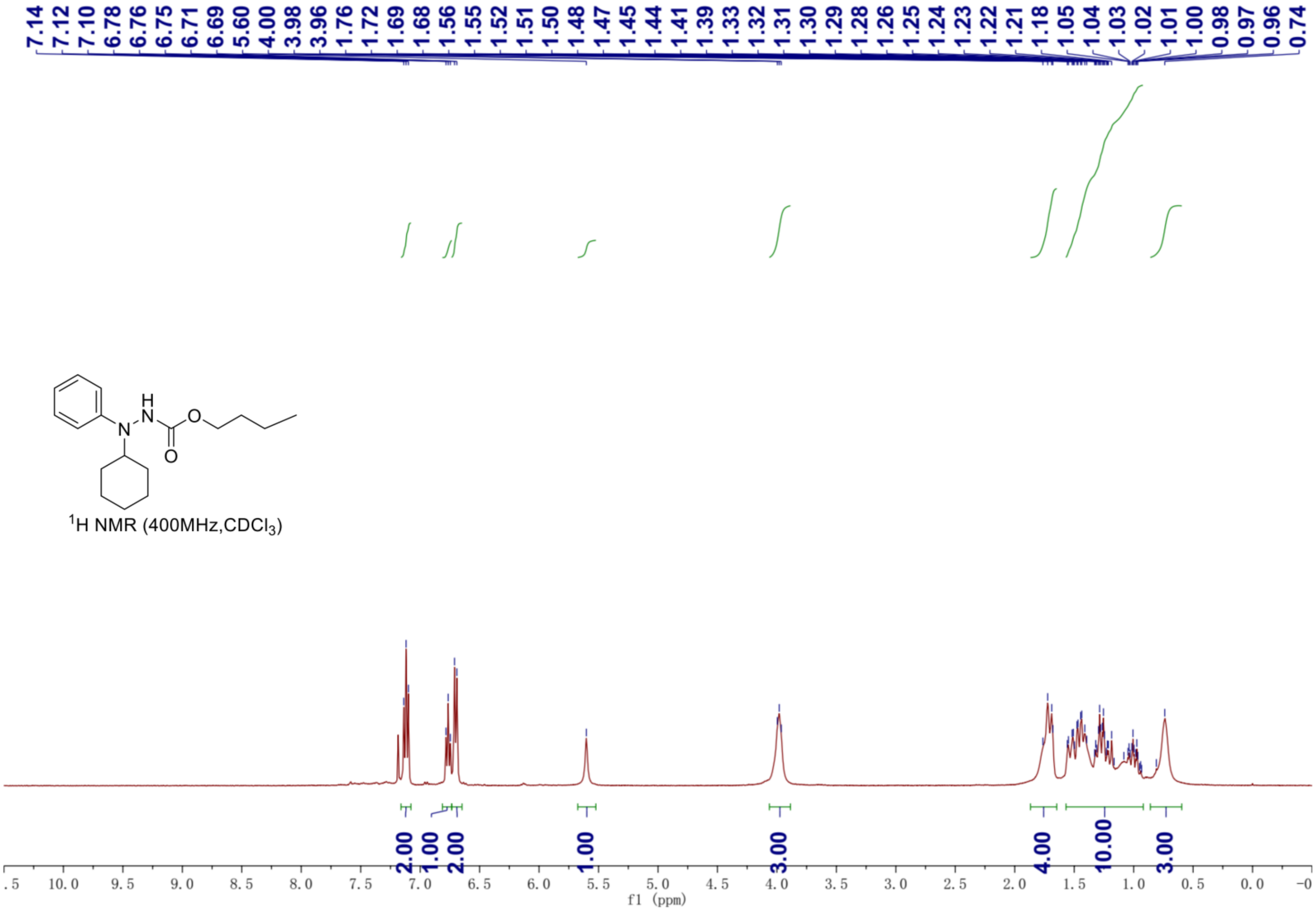

^{1}H NMR (400MHz,CDCl$_3$)

Go to table of contents

Butyl 2-cyclohexyl-2-phenylhydrazine-1-carboxylate (4o)

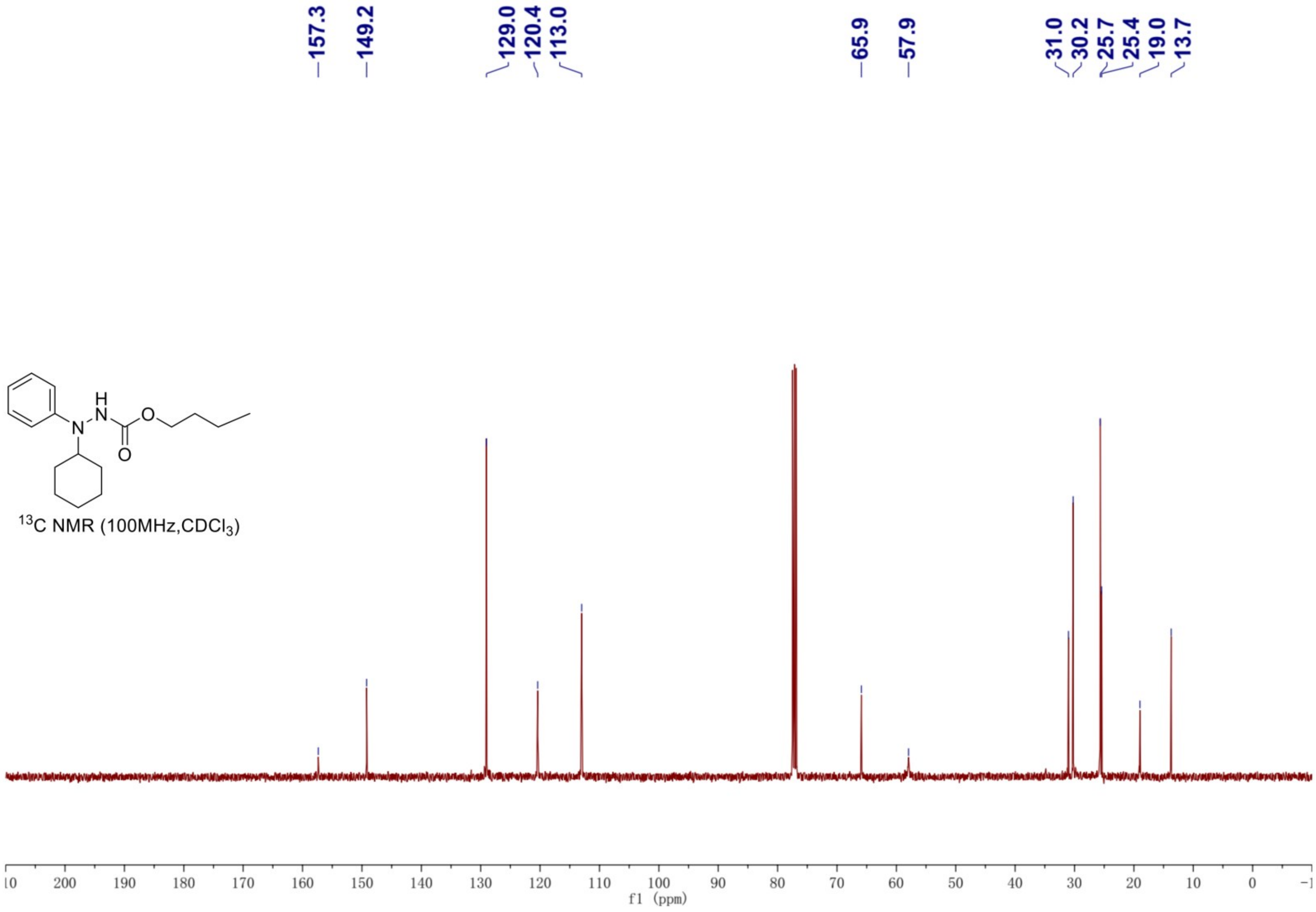

Go to table of contents

4-Phenyl-1-(tetrahydrothiophen-2-yl)-1,2,4-triazolidine-3,5-dione (4p)

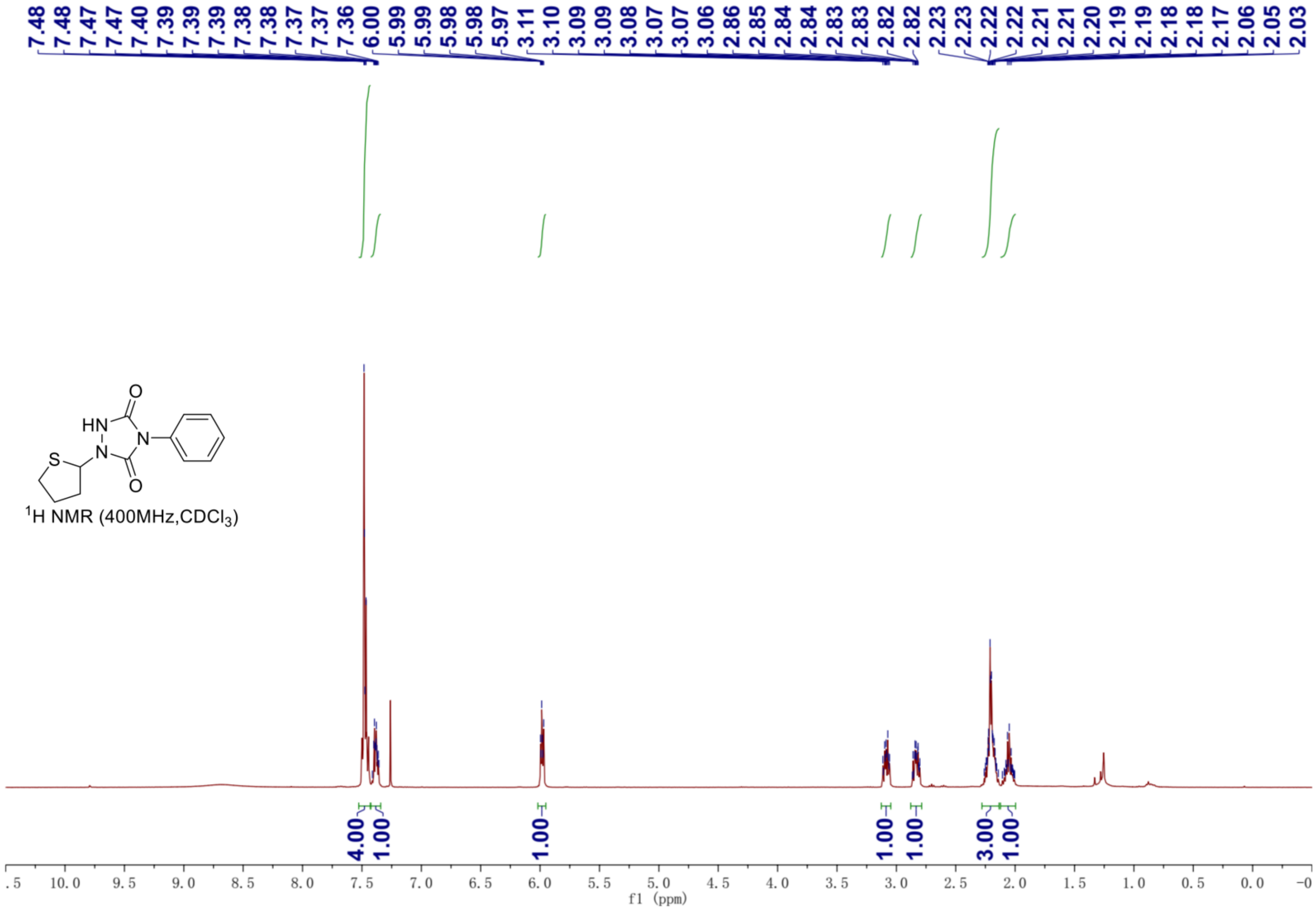

Go to table of contents

4-Phenyl-1-(tetrahydrothiophen-2-yl)-1,2,4-triazolidine-3,5-dione (4p)

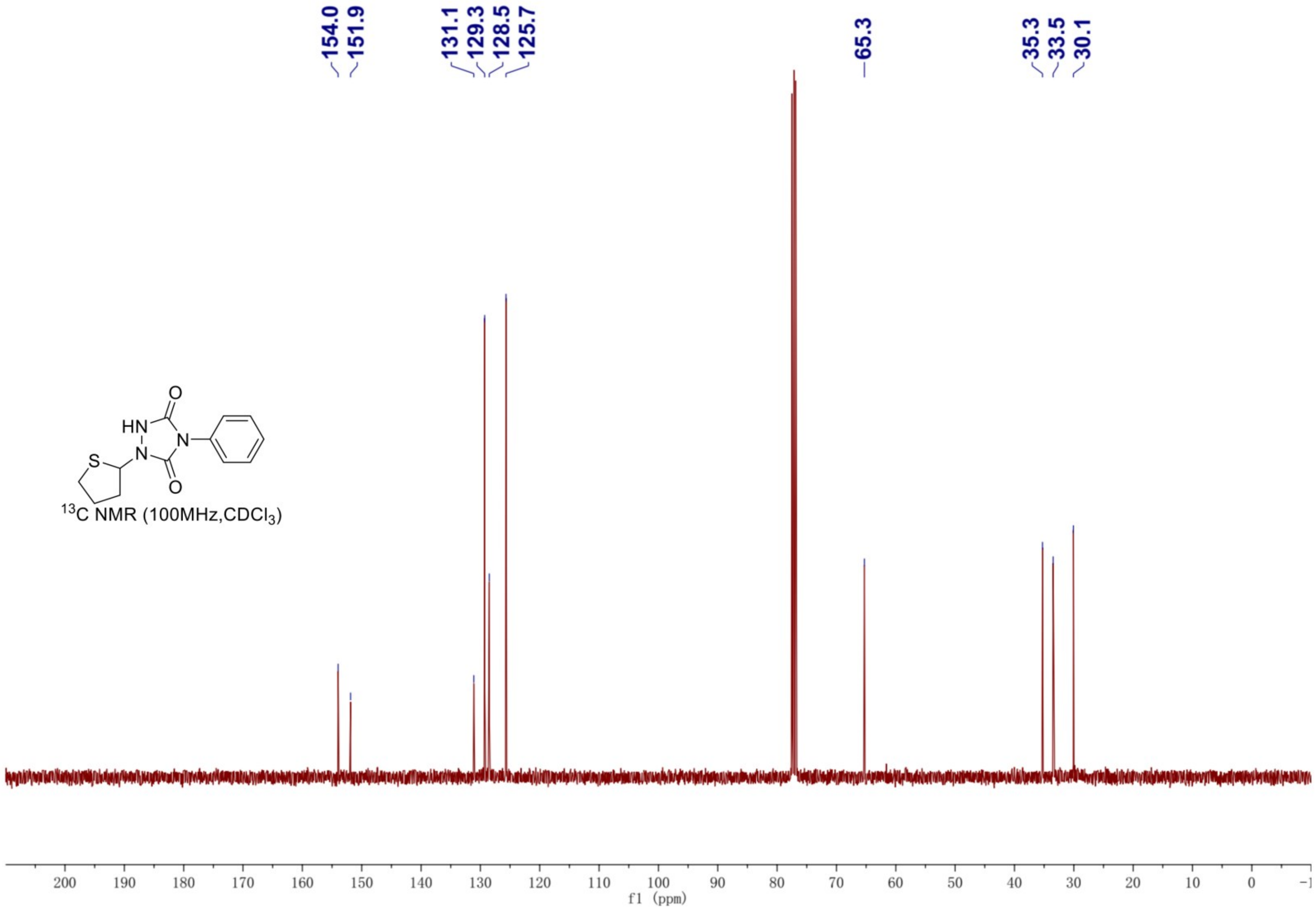

Go to table of contents

2-(2-(Phenylsulfonyl)allyl)-1,4-dioxane (4q)

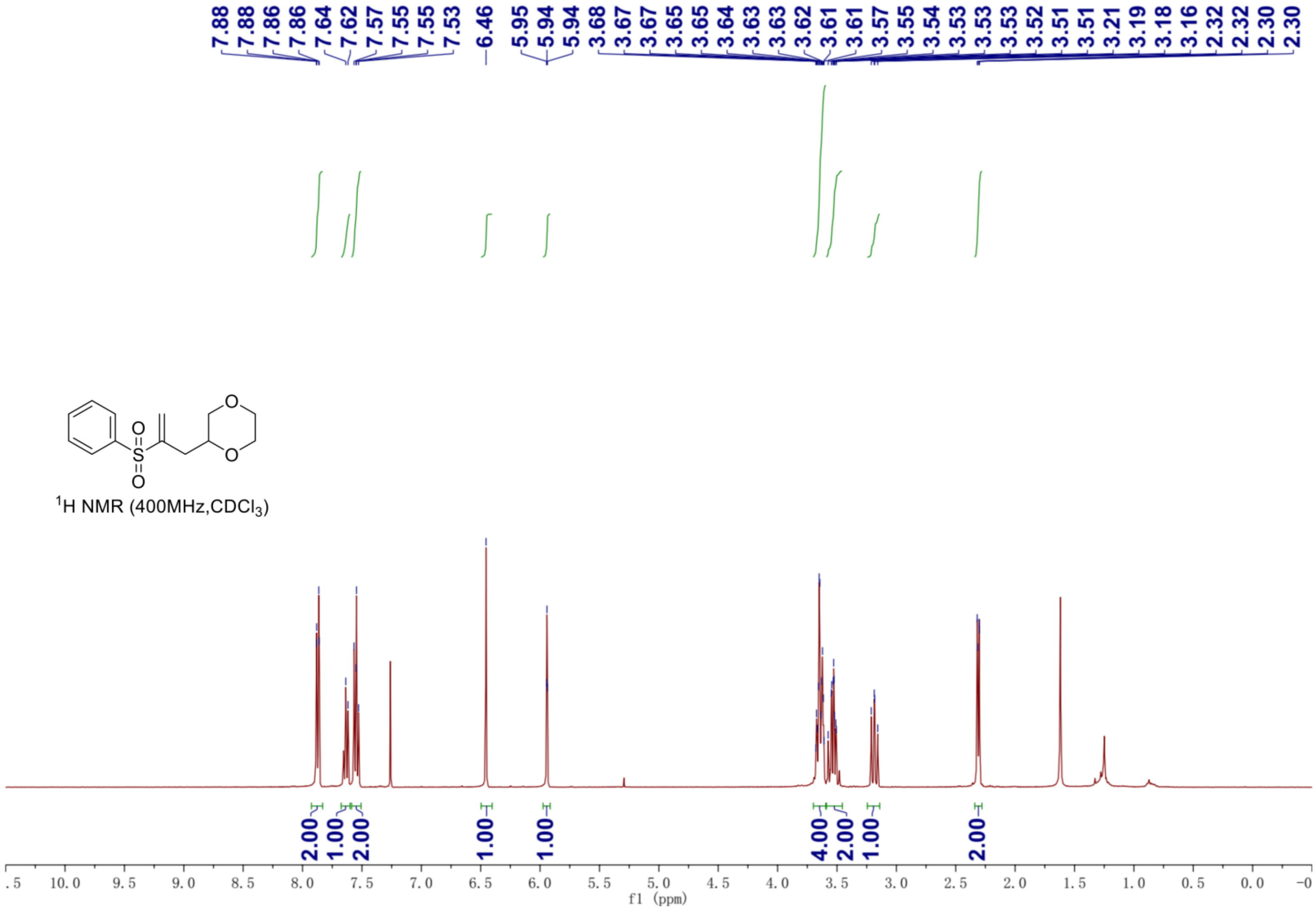

Go to table of contents

2-(2-(Phenylsulfonyl)allyl)-1,4-dioxane (4q)

^{13}C NMR (100MHz,CDCl$_3$)

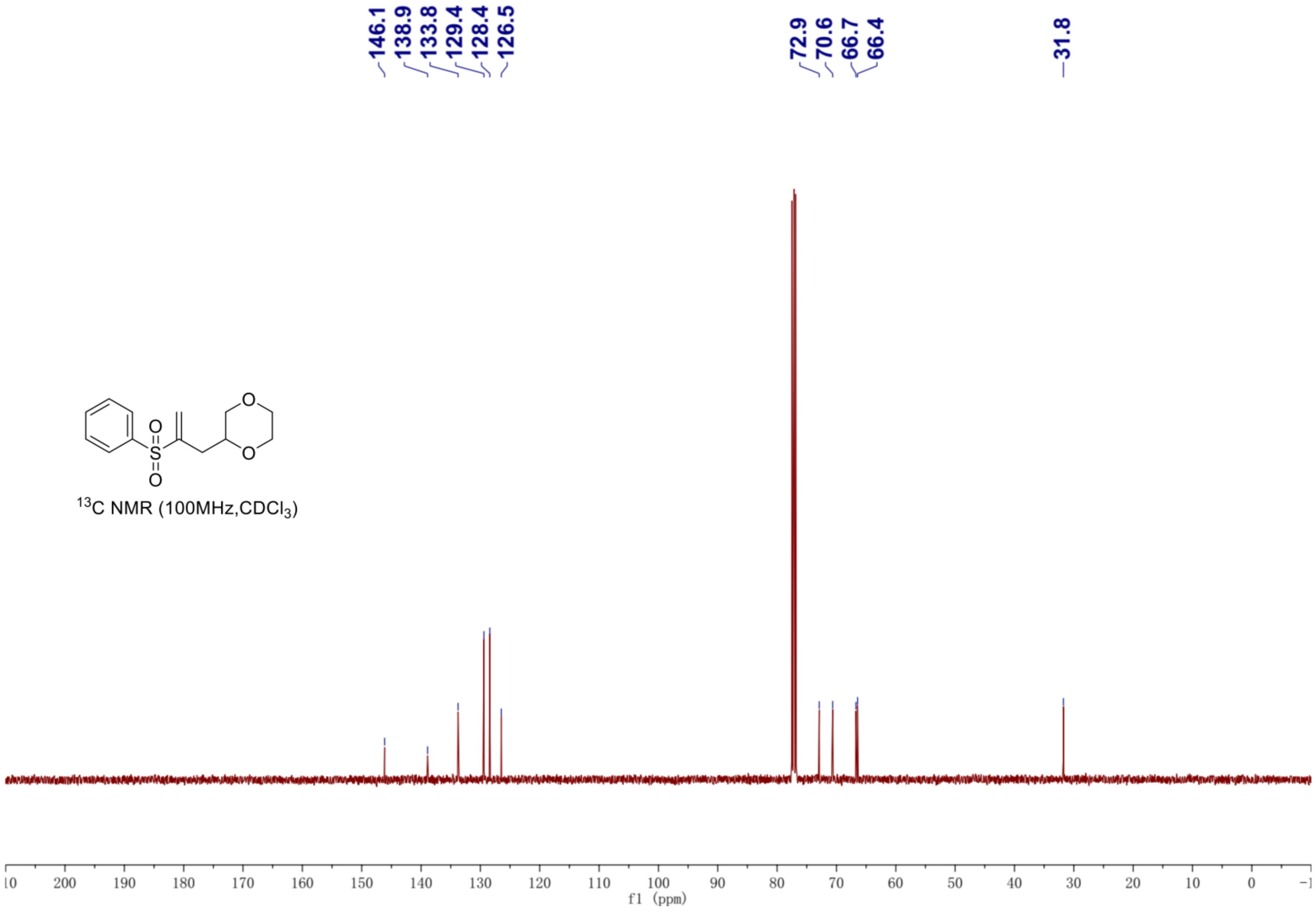

Go to table of contents

2-(2-(Phenylsulfonyl)allyl)tetrahydrothiophene (4r)

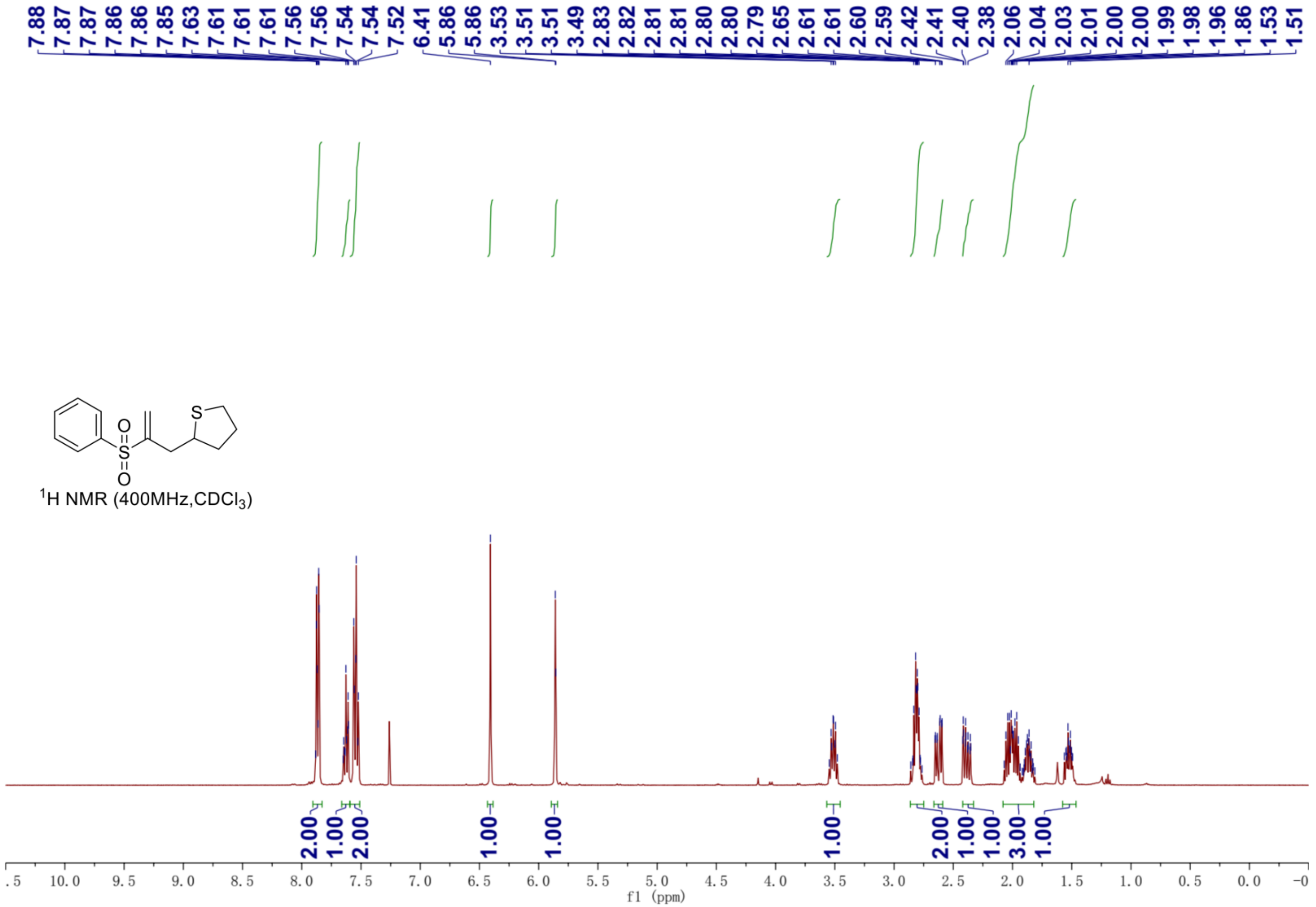

Go to table of contents

2-(2-(Phenylsulfonyl)allyl)tetrahydrothiophene (4r)

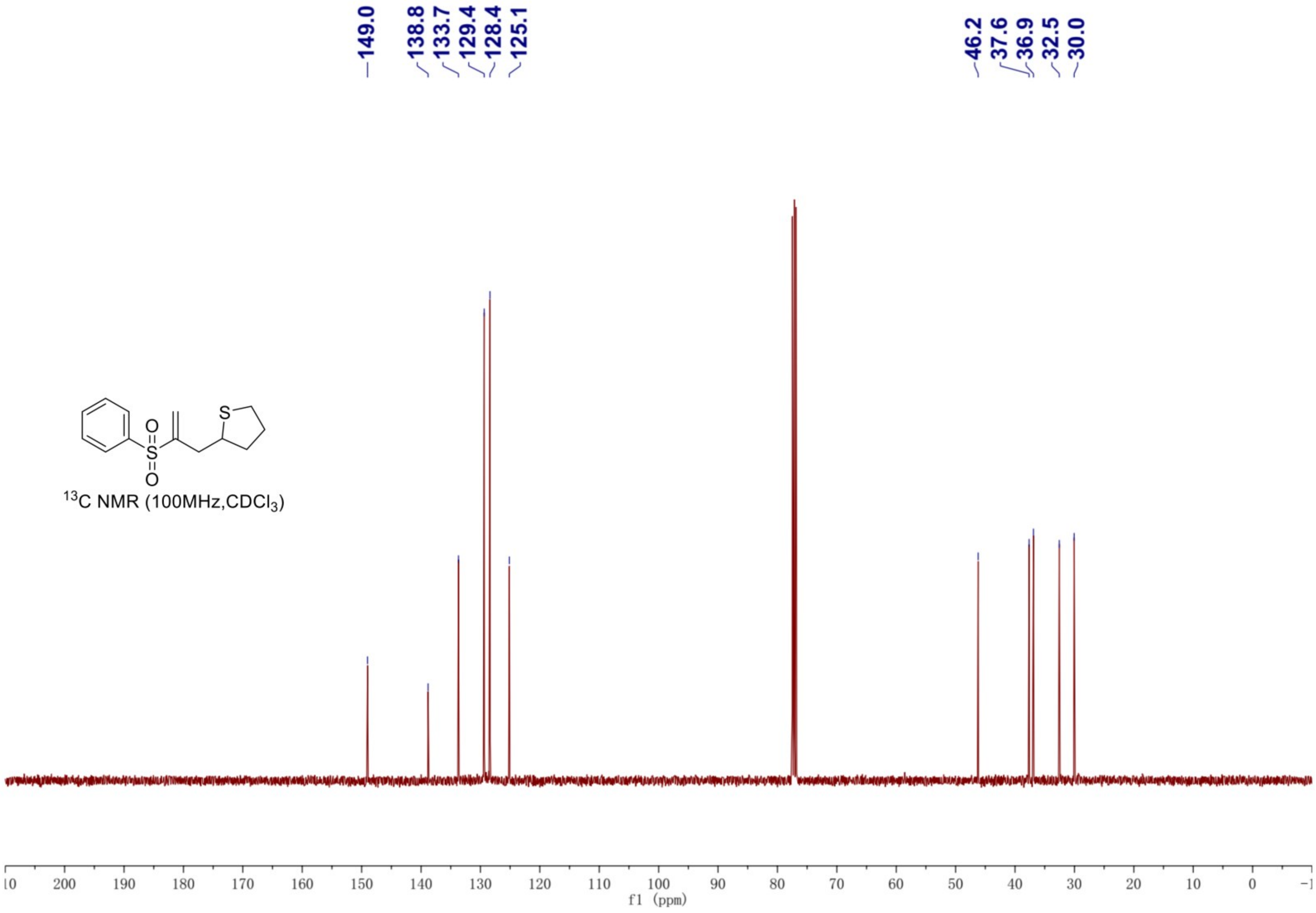

Go to table of contents

S-Cyclohexyl O-phenyl carbonothioate (4s)

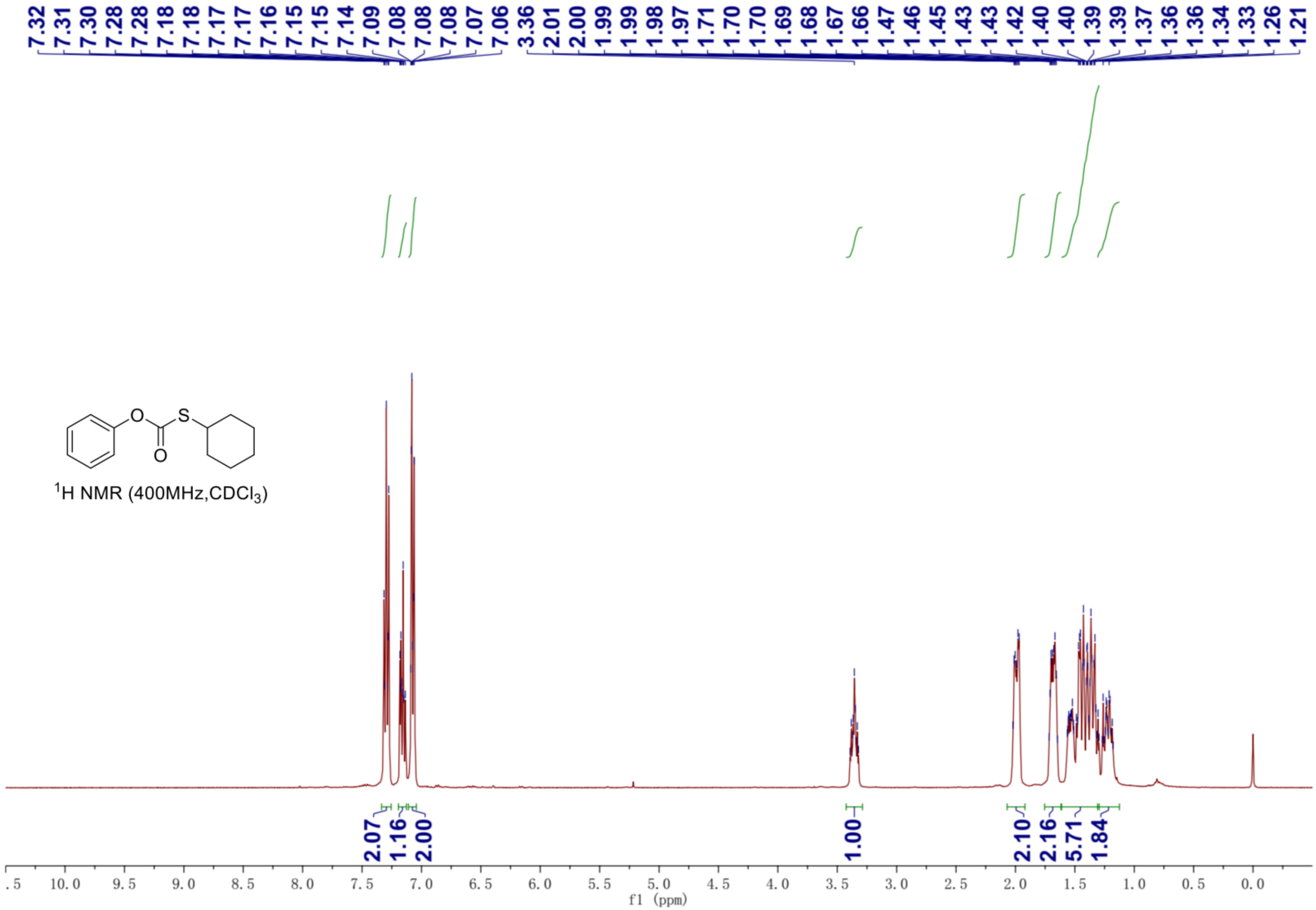

Go to table of contents

S-Cyclohexyl O-phenyl carbonothioate (4s)

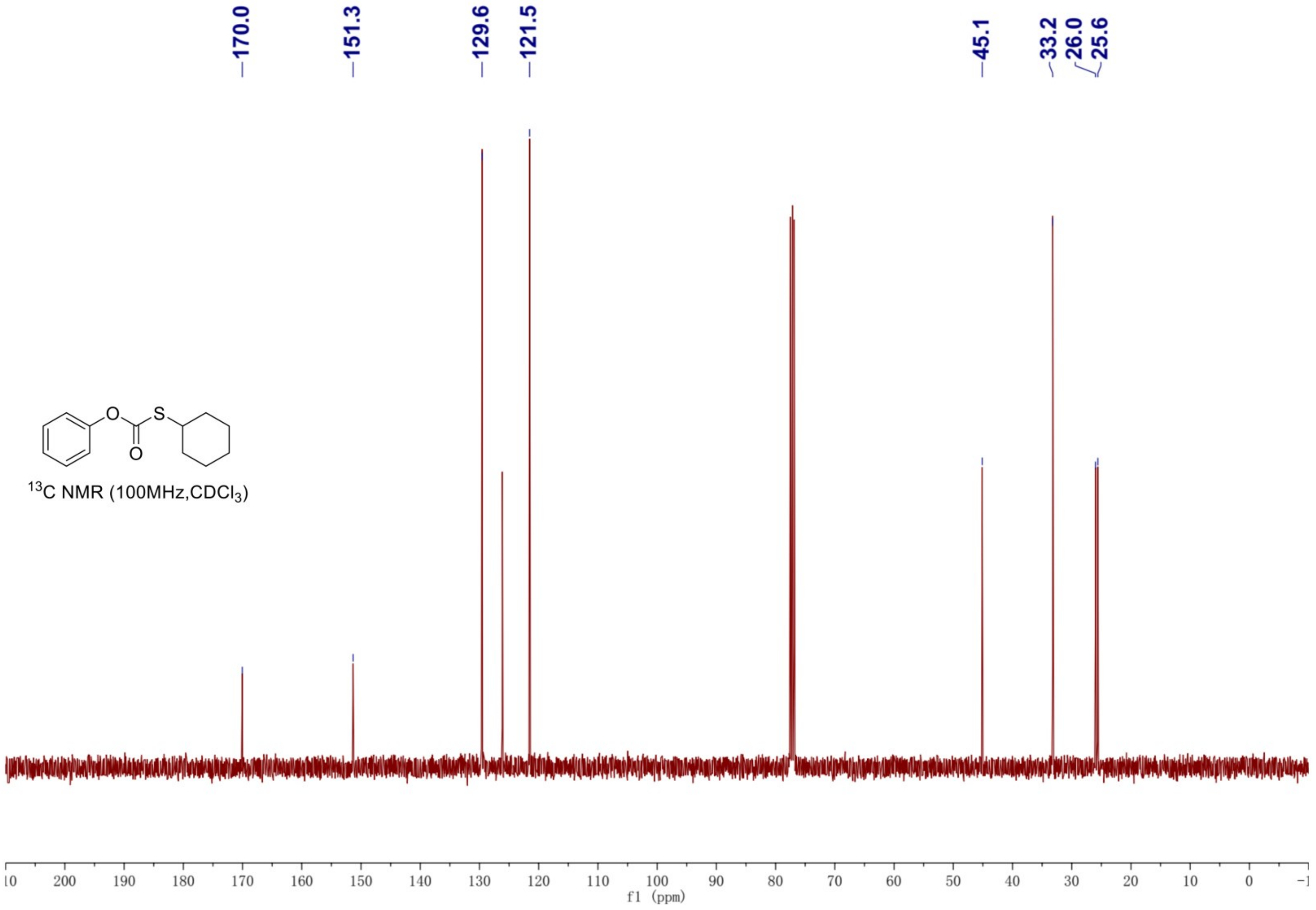

Go to table of contents

1-(Pyridin-4-yl)pyrrolidin-2-one (5a)

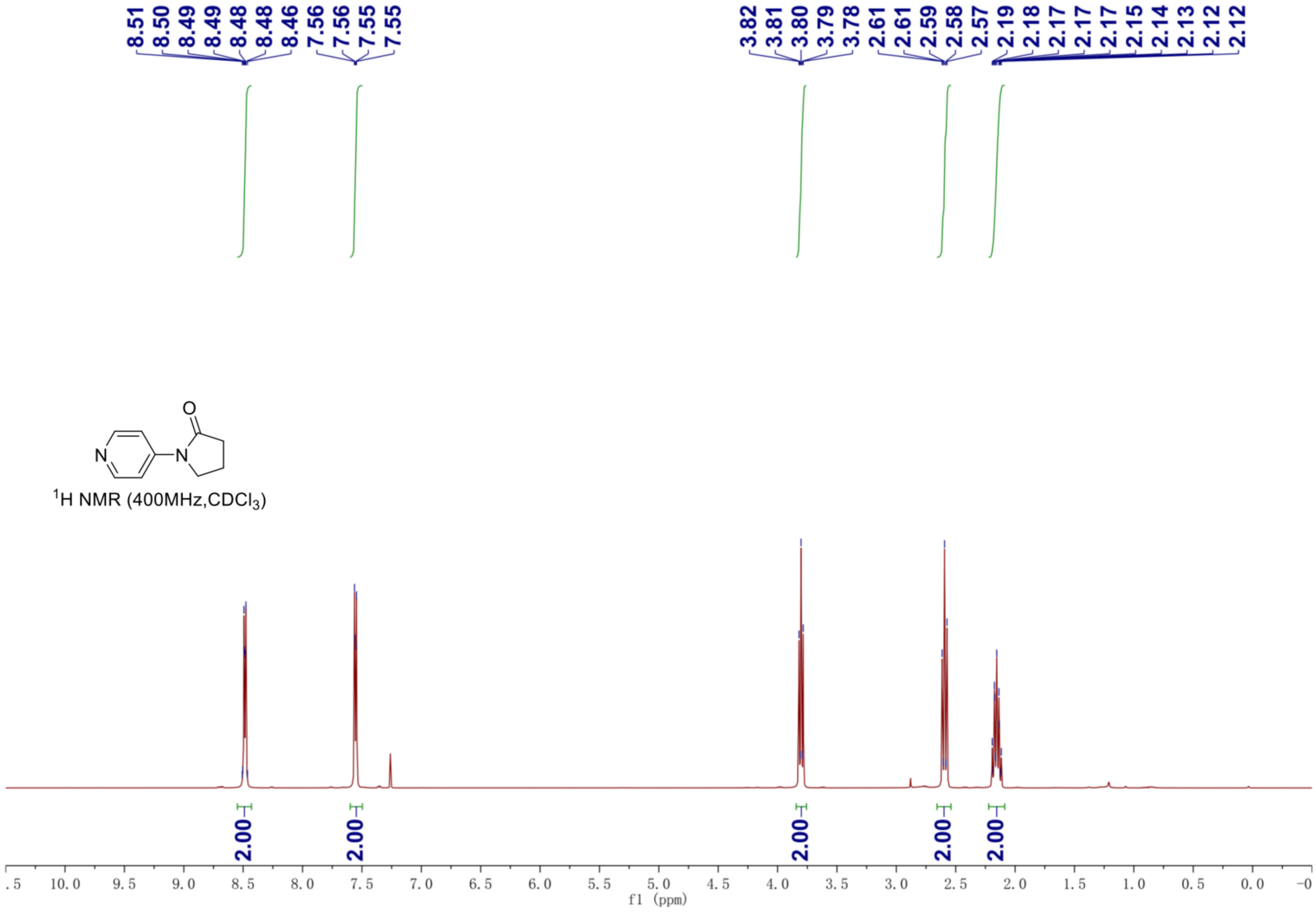

1-(Pyridin-4-yl)pyrrolidin-2-one (5a)

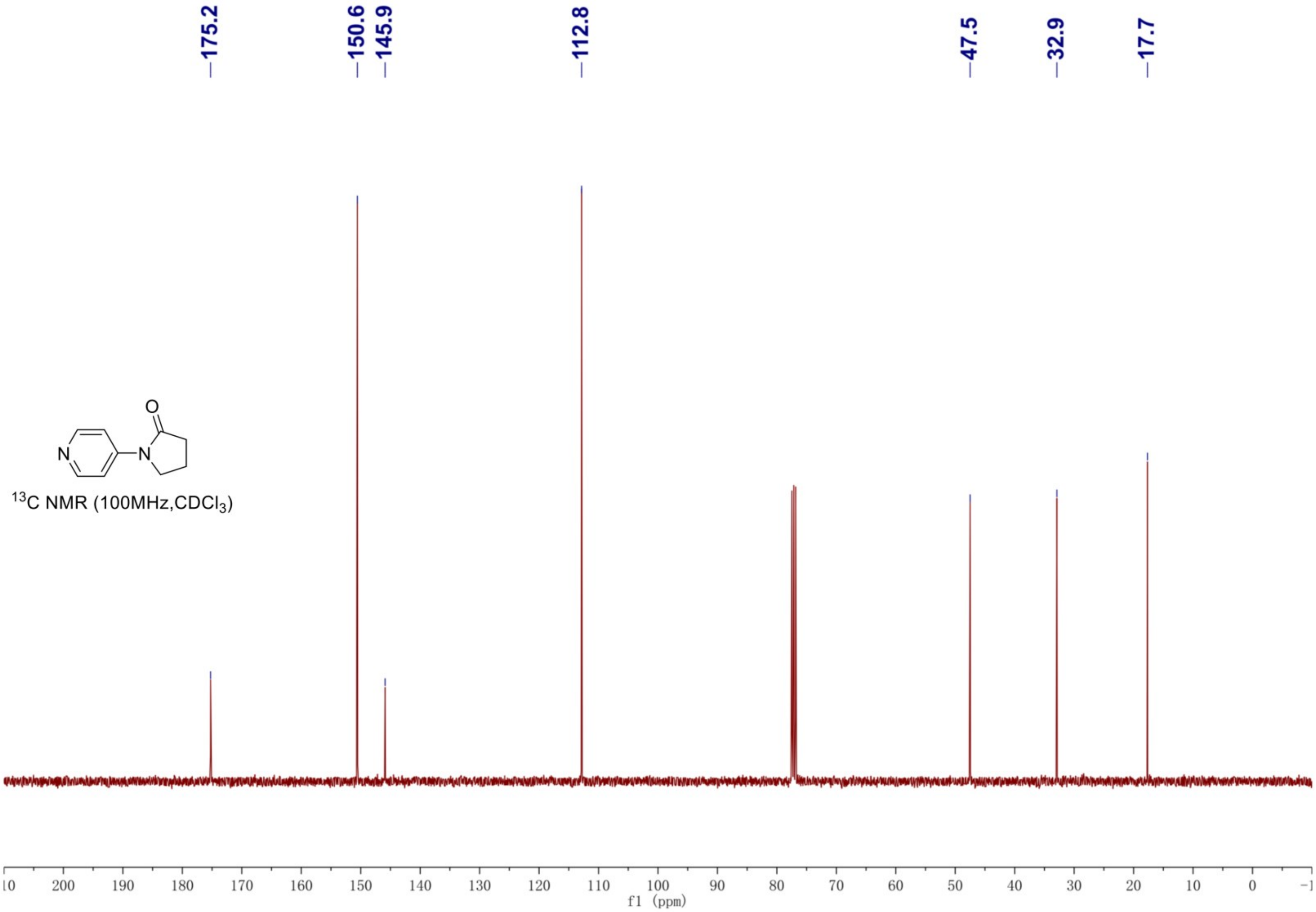

Go to table of contents

1-(3-Bromophenyl)ethan-1-one (5b)

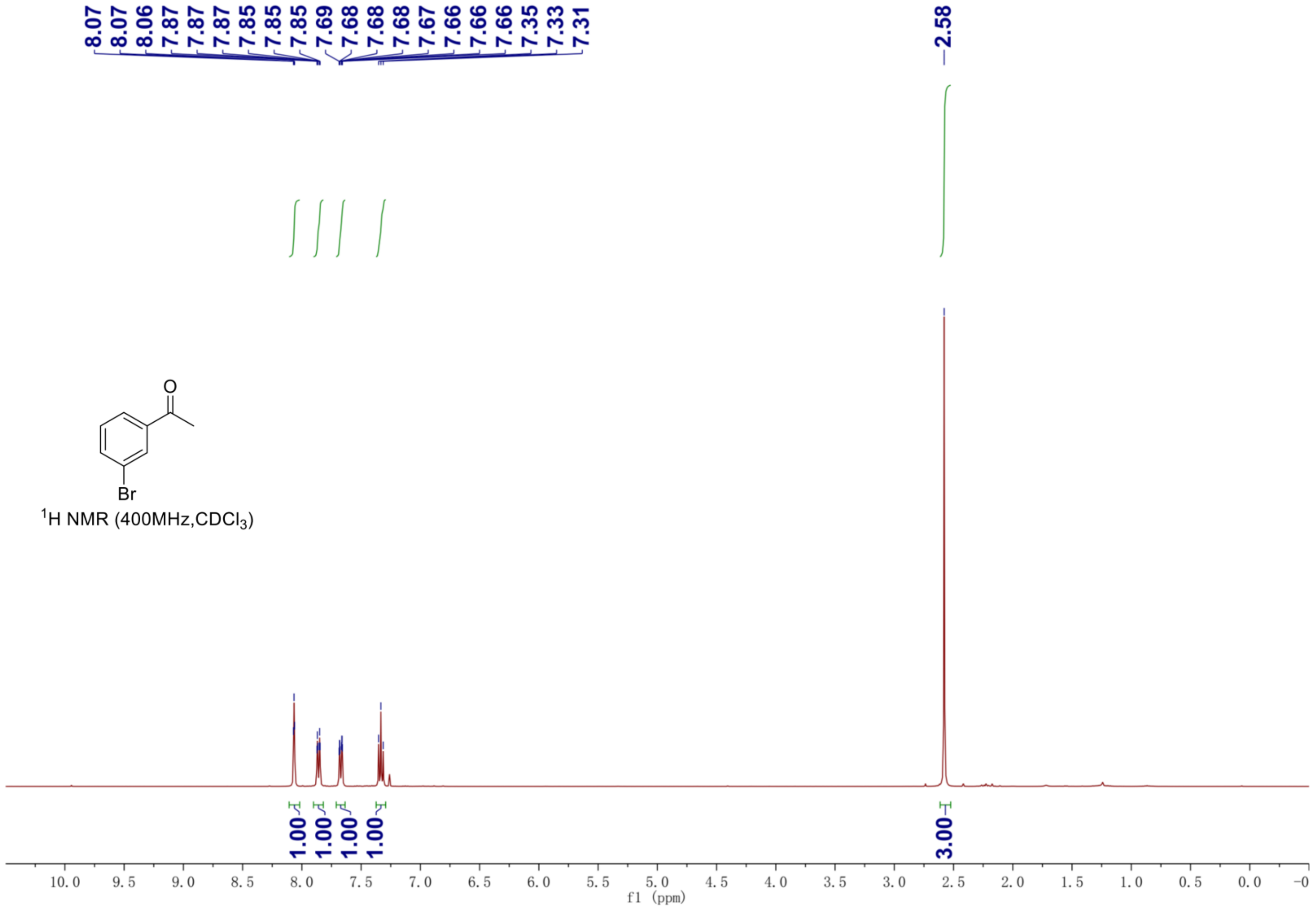

Go to table of contents

1-(3-Bromophenyl)ethan-1-one (5b)

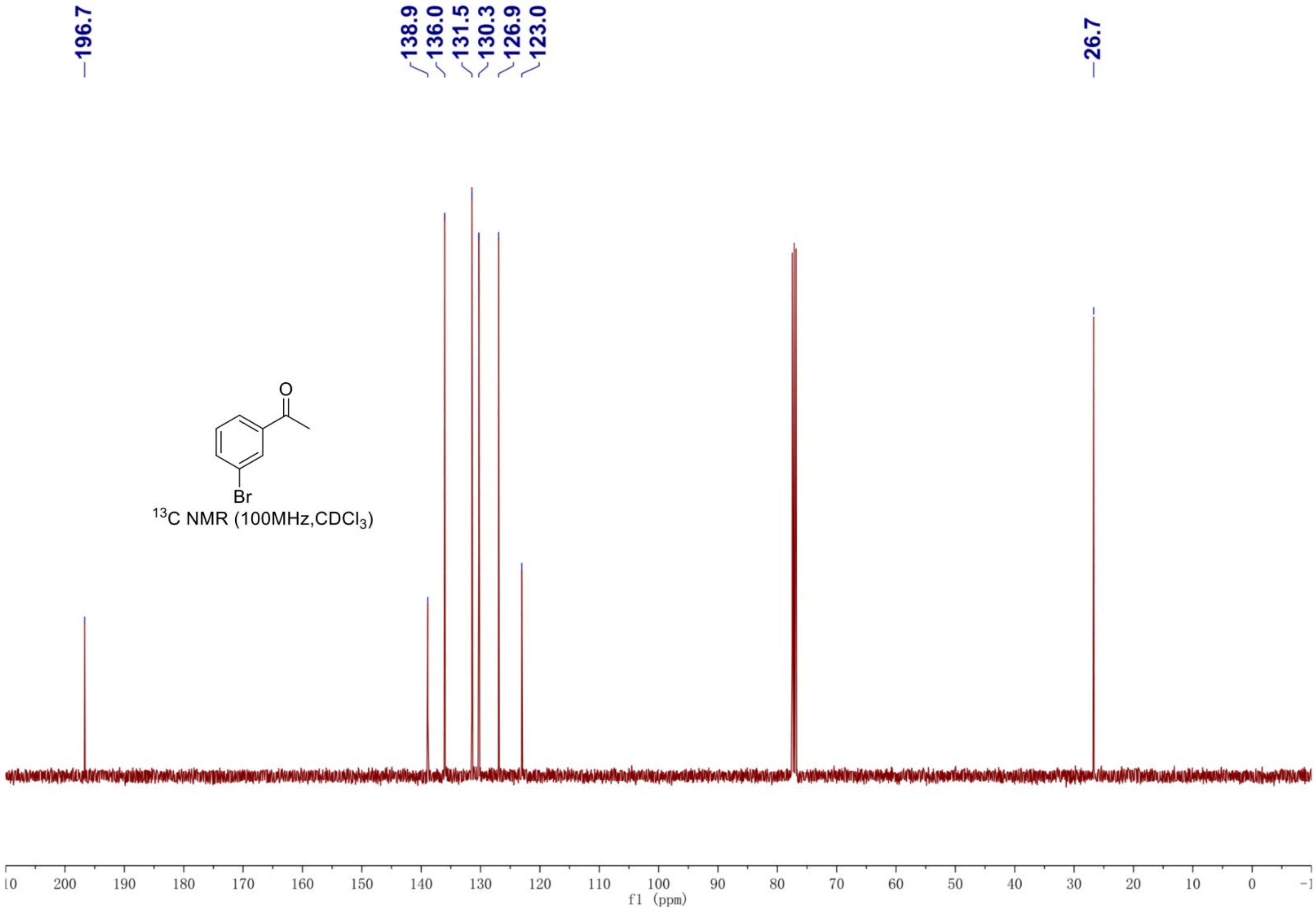

Go to table of contents

3,4-Dihydronaphthalen-1(2*H*)-one (5c)

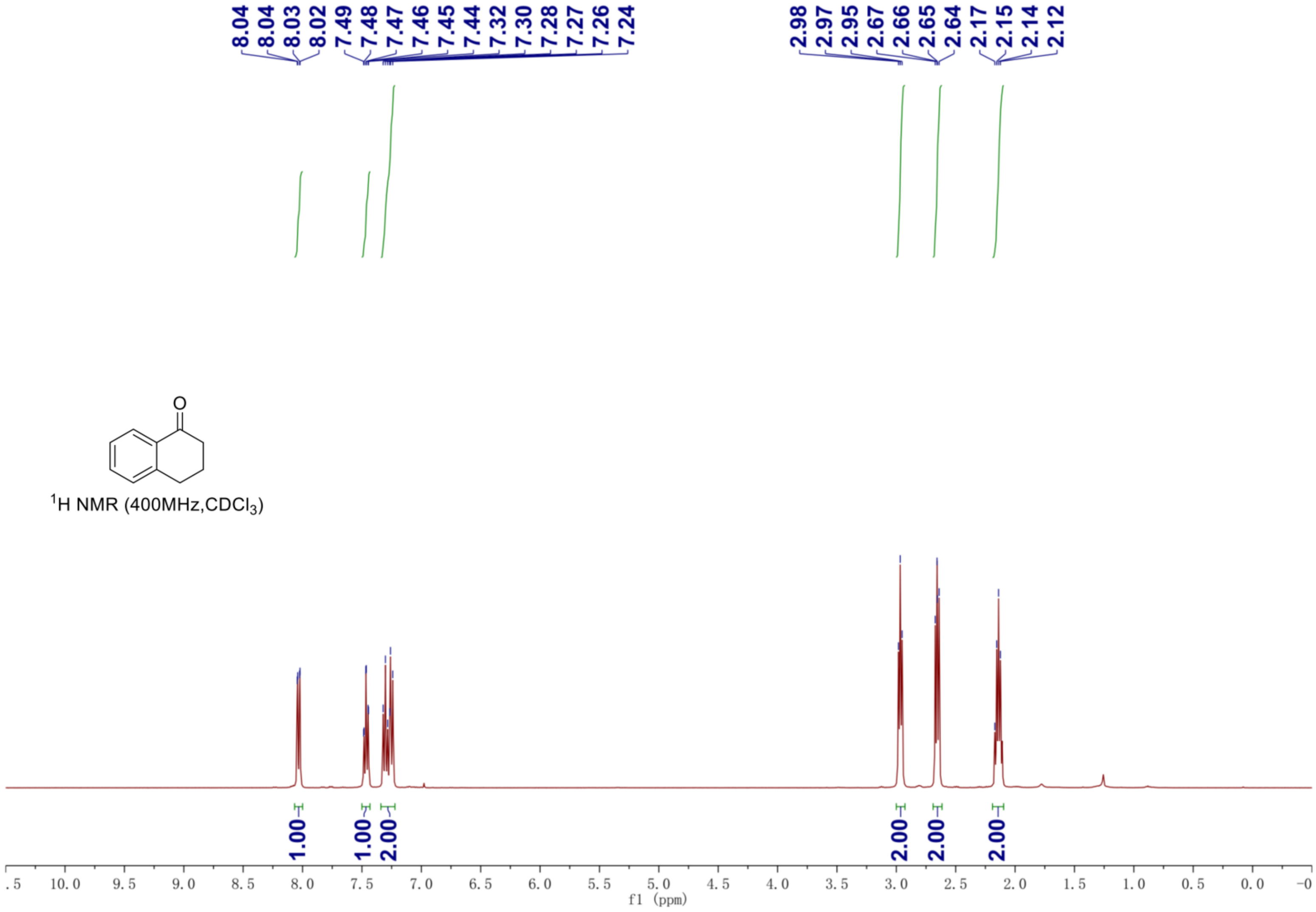

Go to table of contents

3,4-Dihydronaphthalen-1(2*H*)-one (5c)

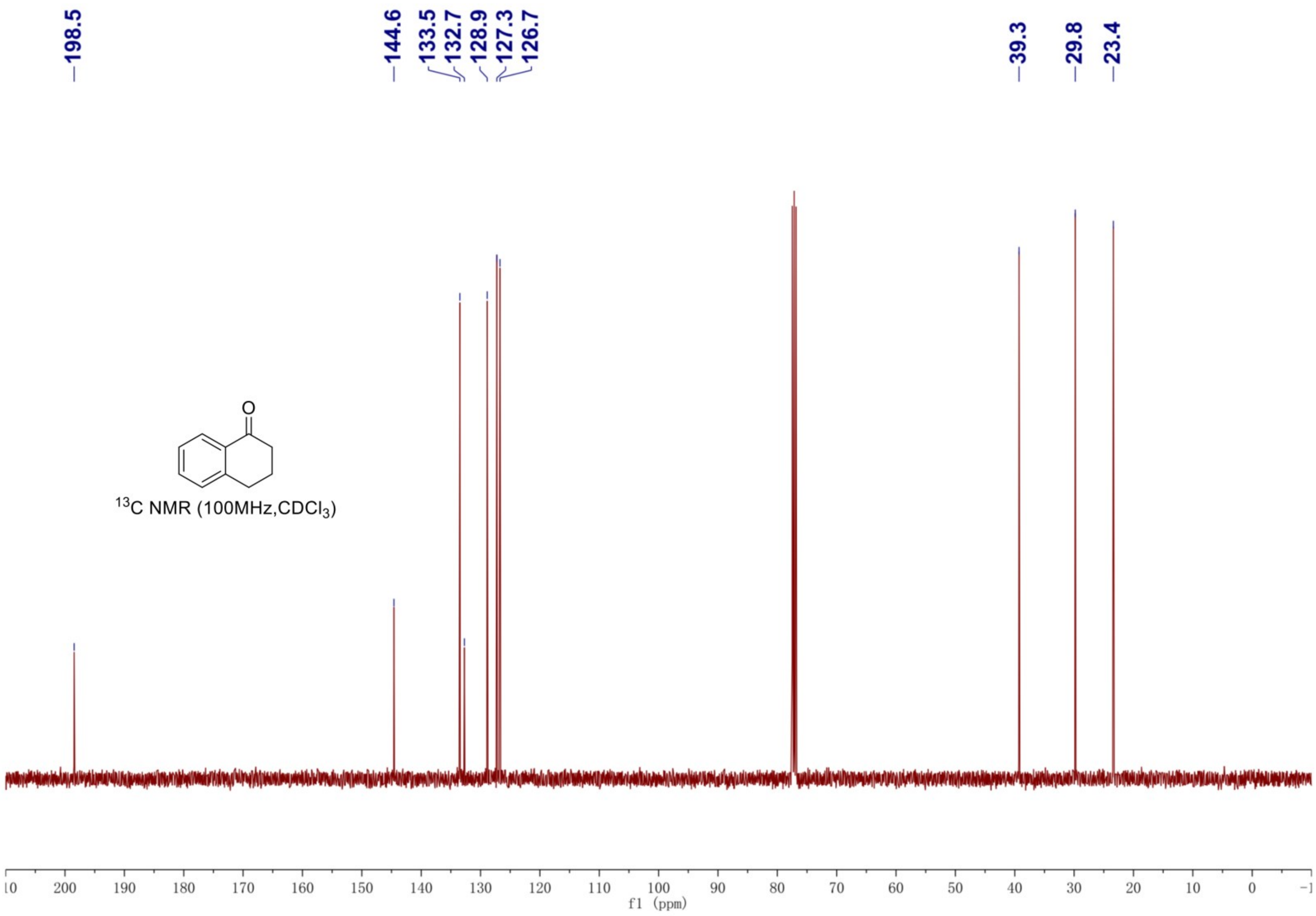

Go to table of contents

9*H*-Fluoren-9-one (5d)

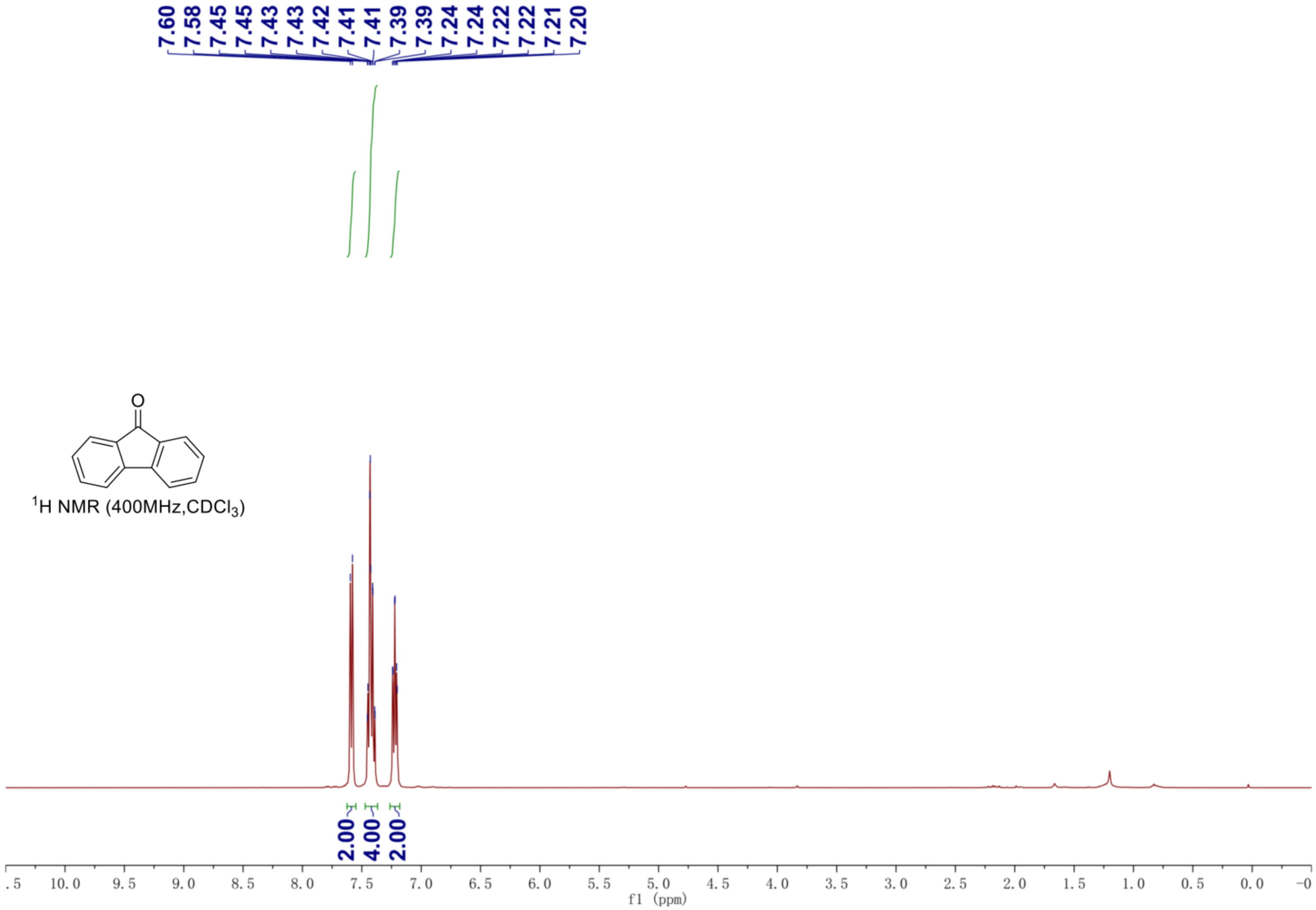

Go to table of contents

9*H*-Fluoren-9-one (5d)

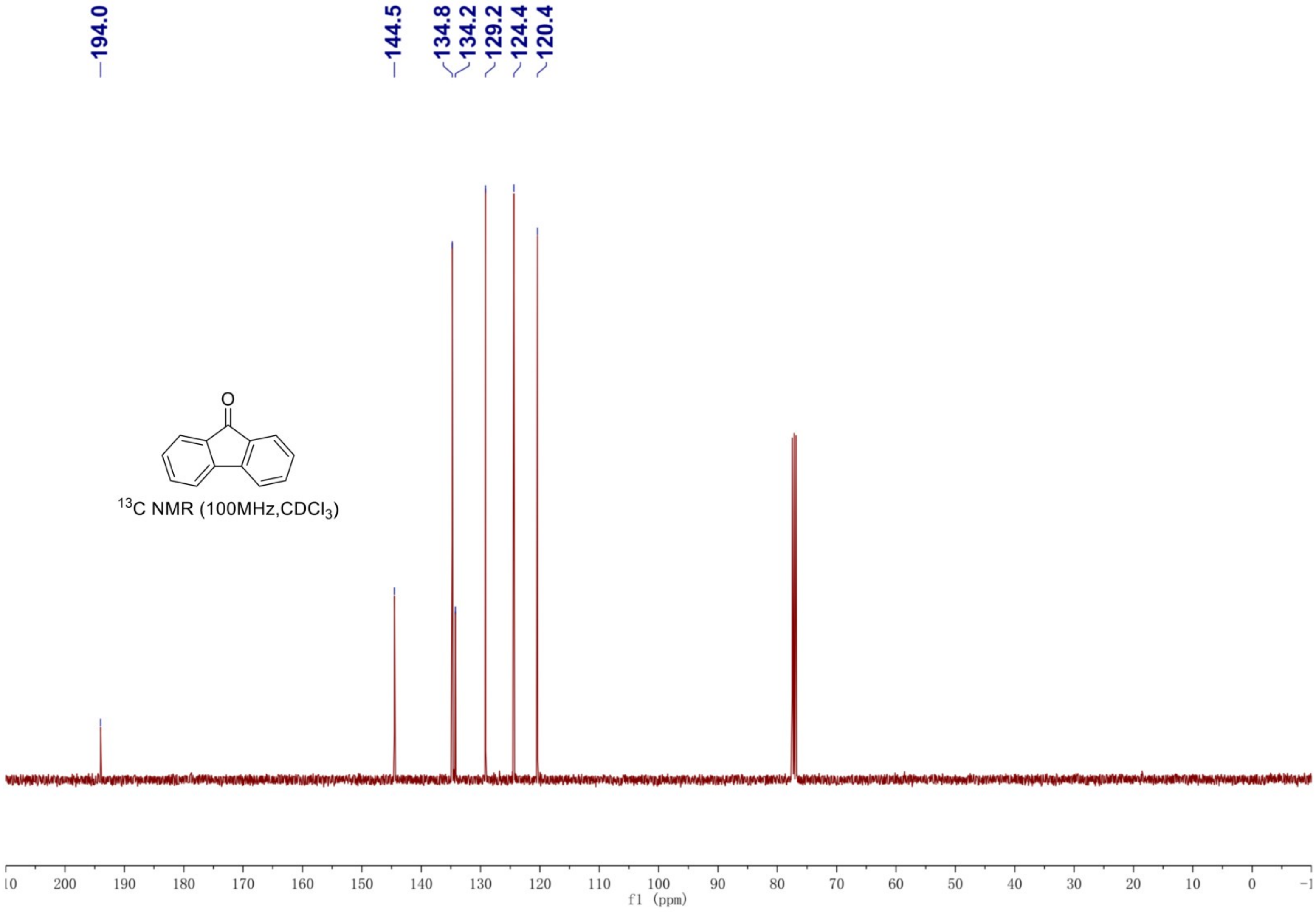

2-Bromo-9*H*-fluoren-9-one (5e)

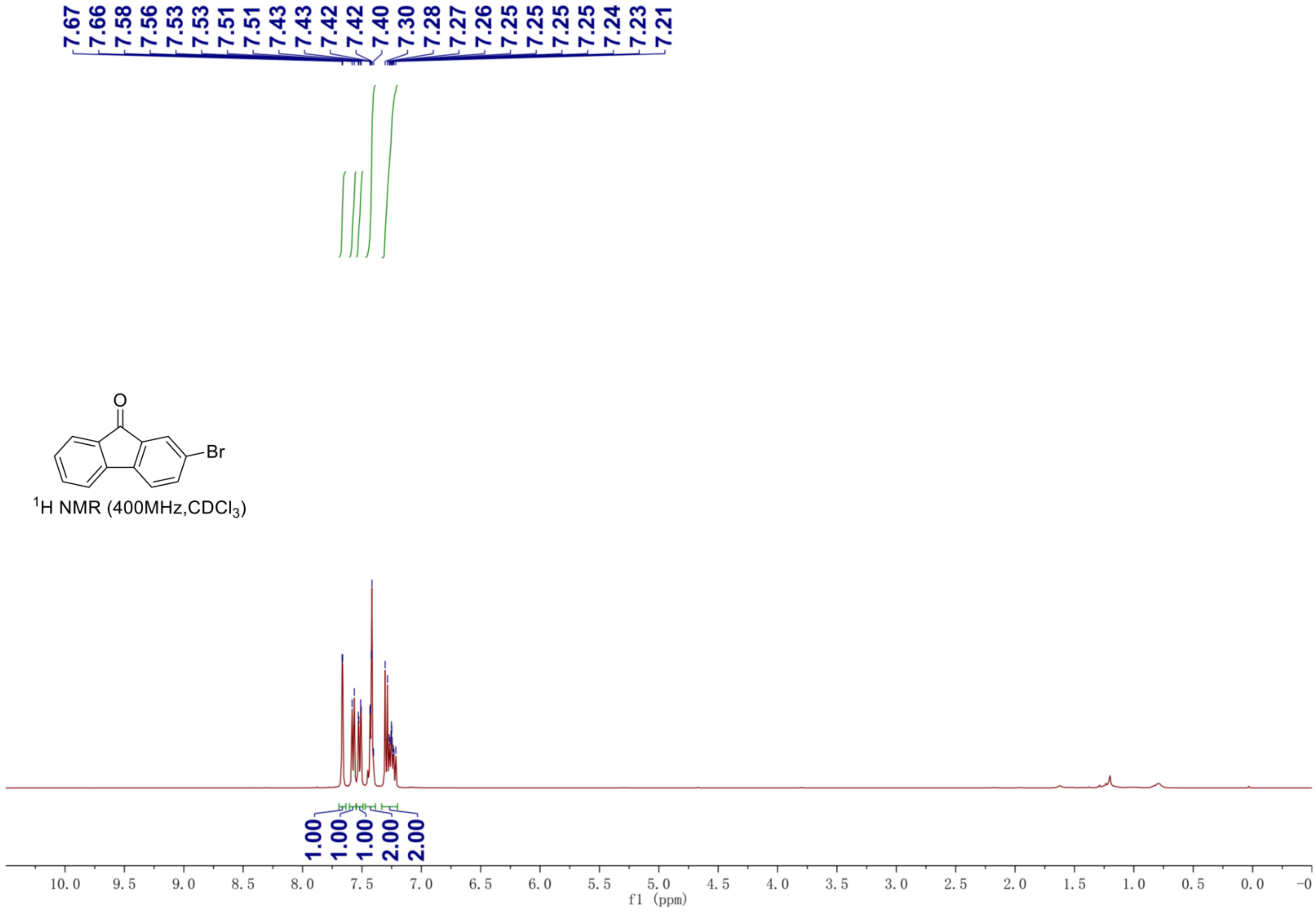

Go to table of contents

2-Bromo-9*H*-fluoren-9-one (5e)

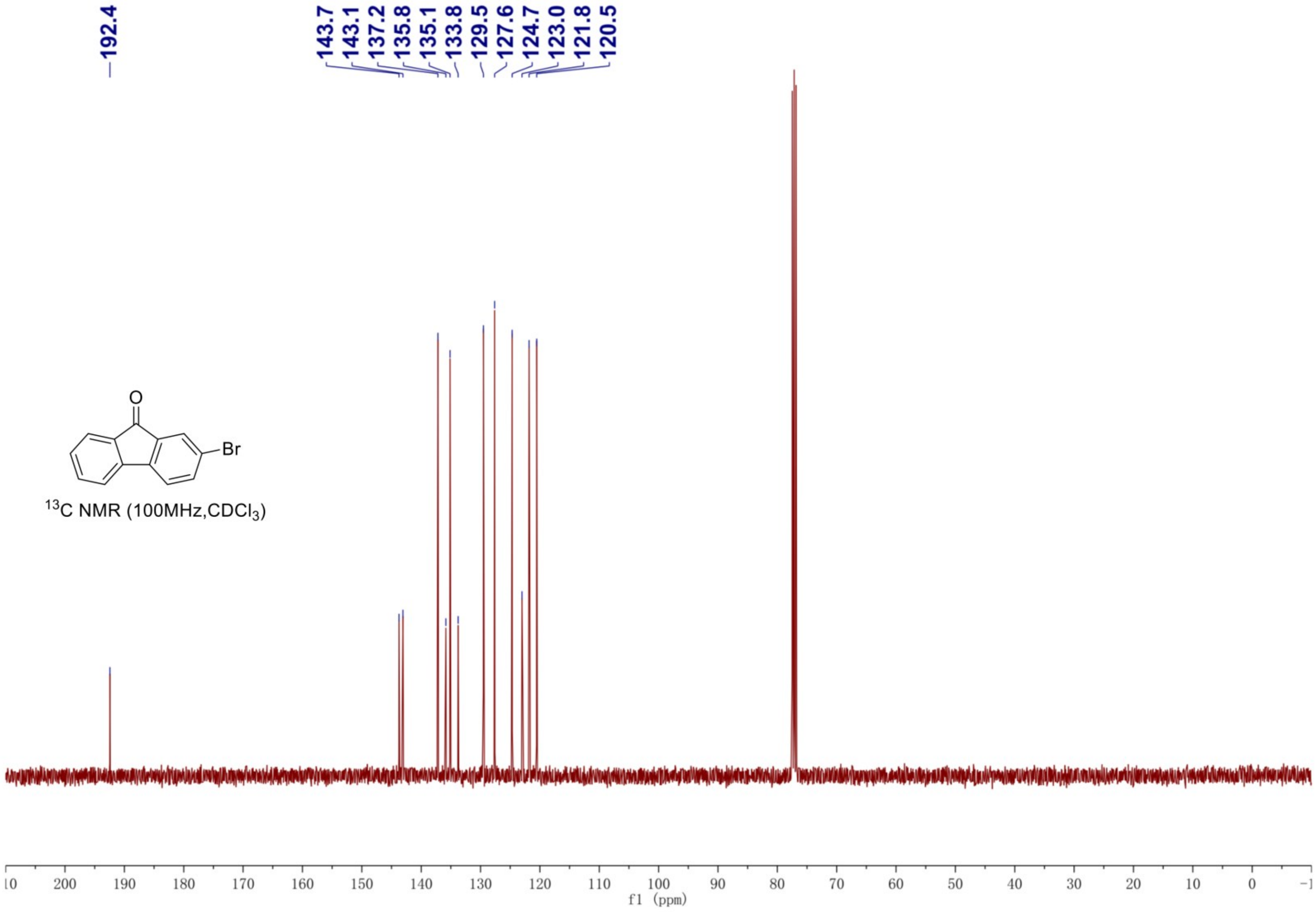

Go to table of contents

Benzophenone (5f)

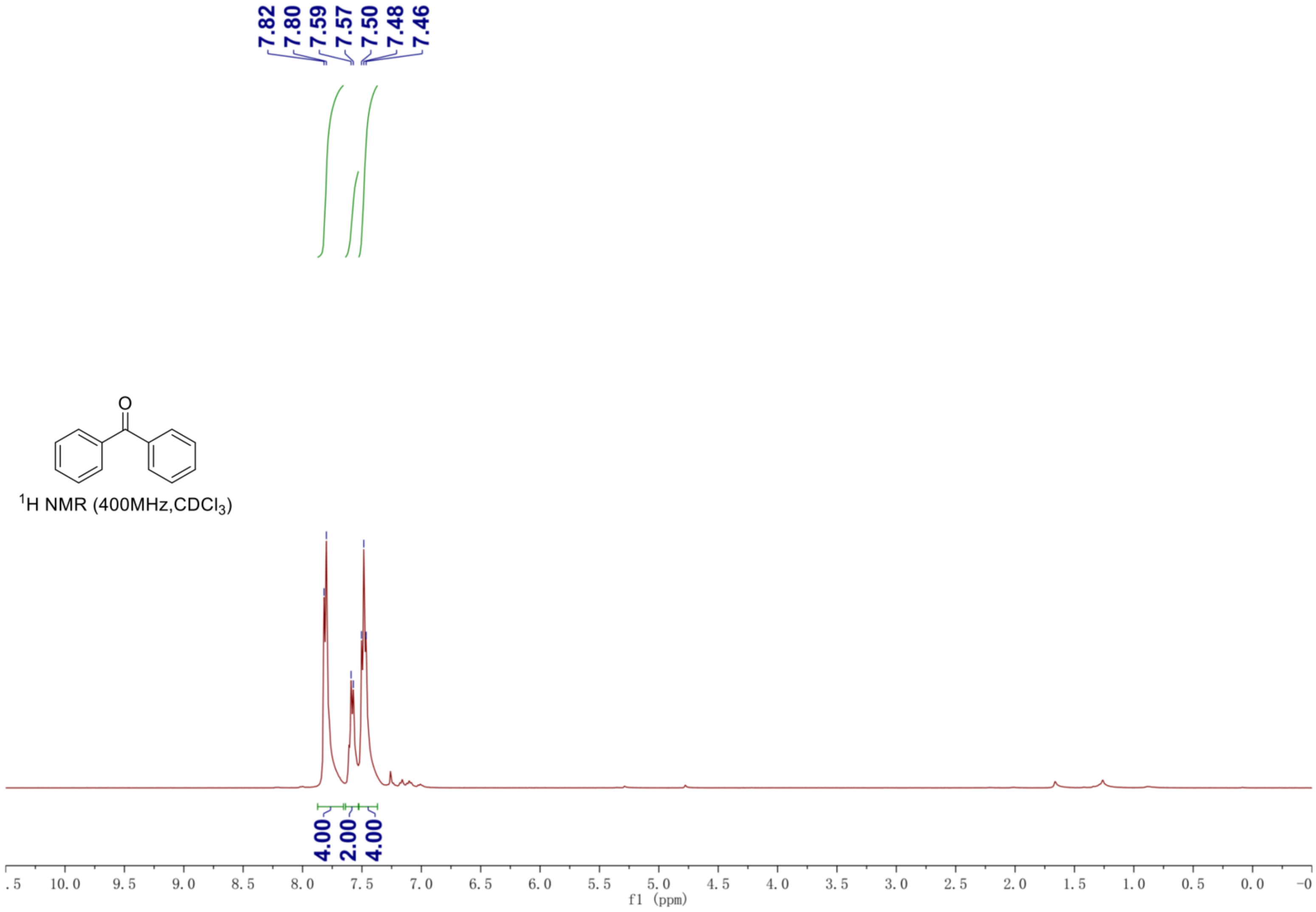

Go to table of contents

Benzophenone (5f)

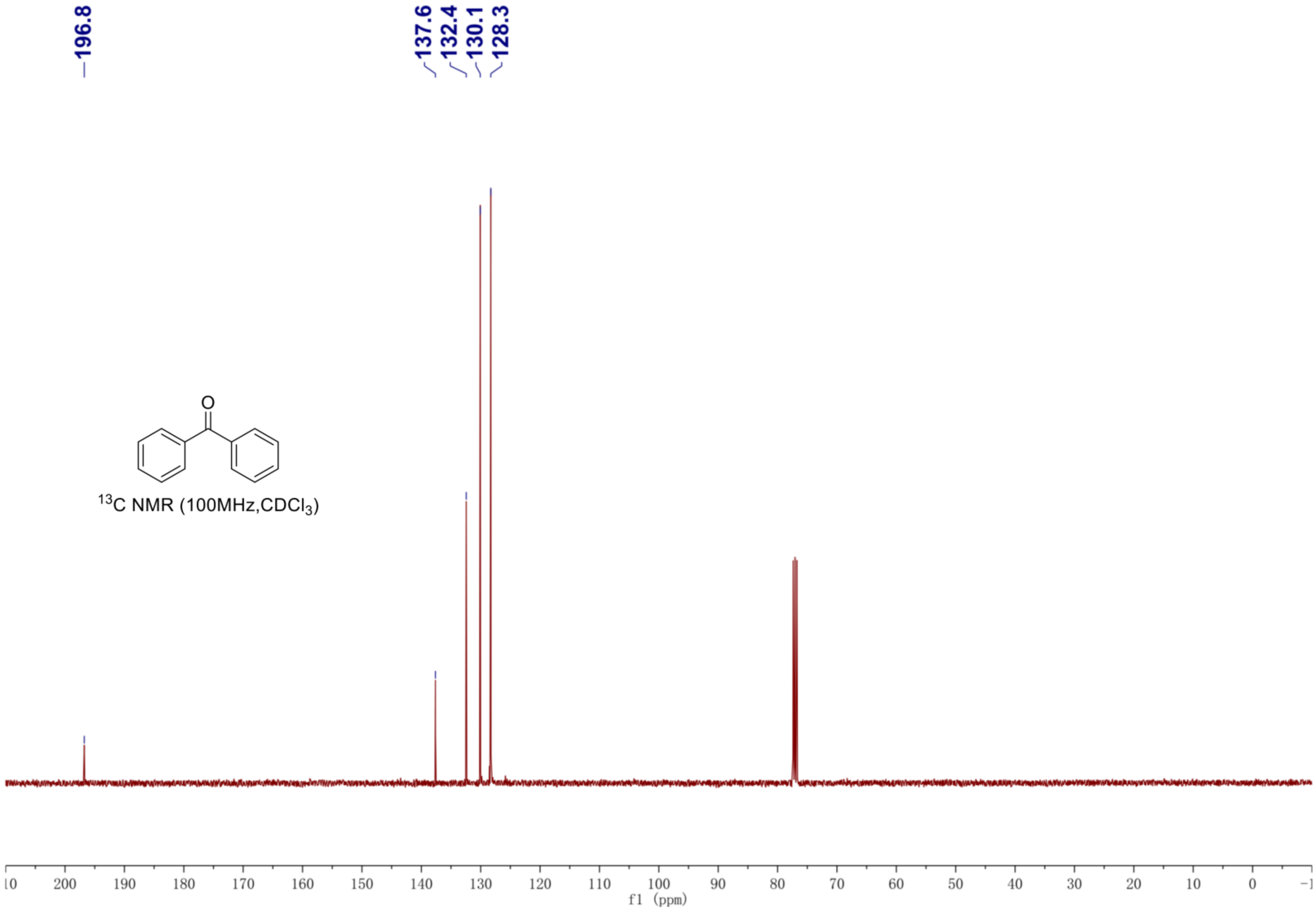

Go to table of contents

1-(((2*S*,4*R*,5*S*)-5-Isopropyl-2-methyl-4-((triisopropylsilyl)oxy)cyclohexyl)oxy)-2,2,6,6-tetramethylpiperidin-4-ol (6a)

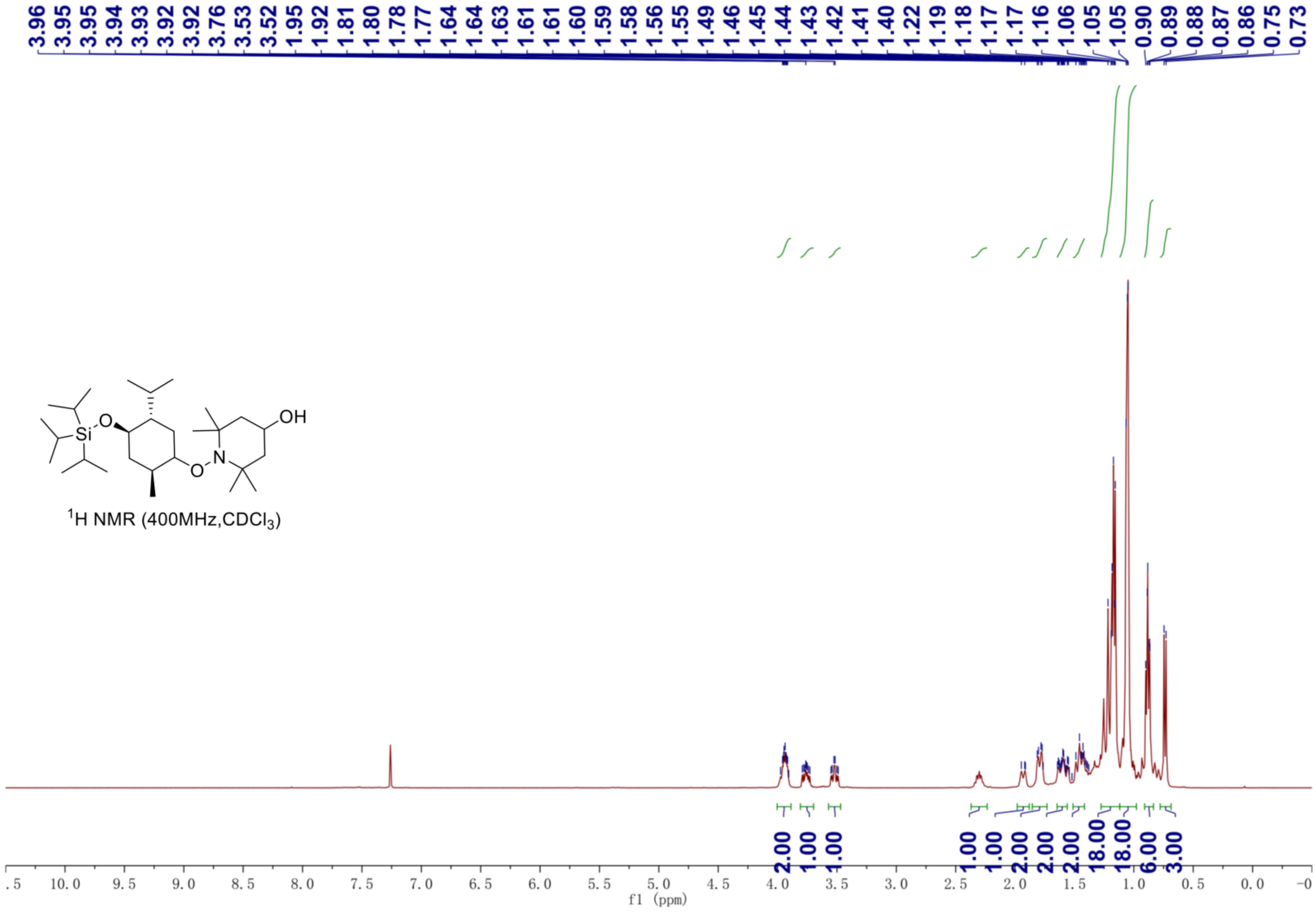

Go to table of contents

1-(((2*S*,4*R*,5*S*)-5-Isopropyl-2-methyl-4-((triisopropylsilyl)oxy)cyclohexyl)oxy)-2,2,6,6-tetramethylpiperidin-4-ol (6a)

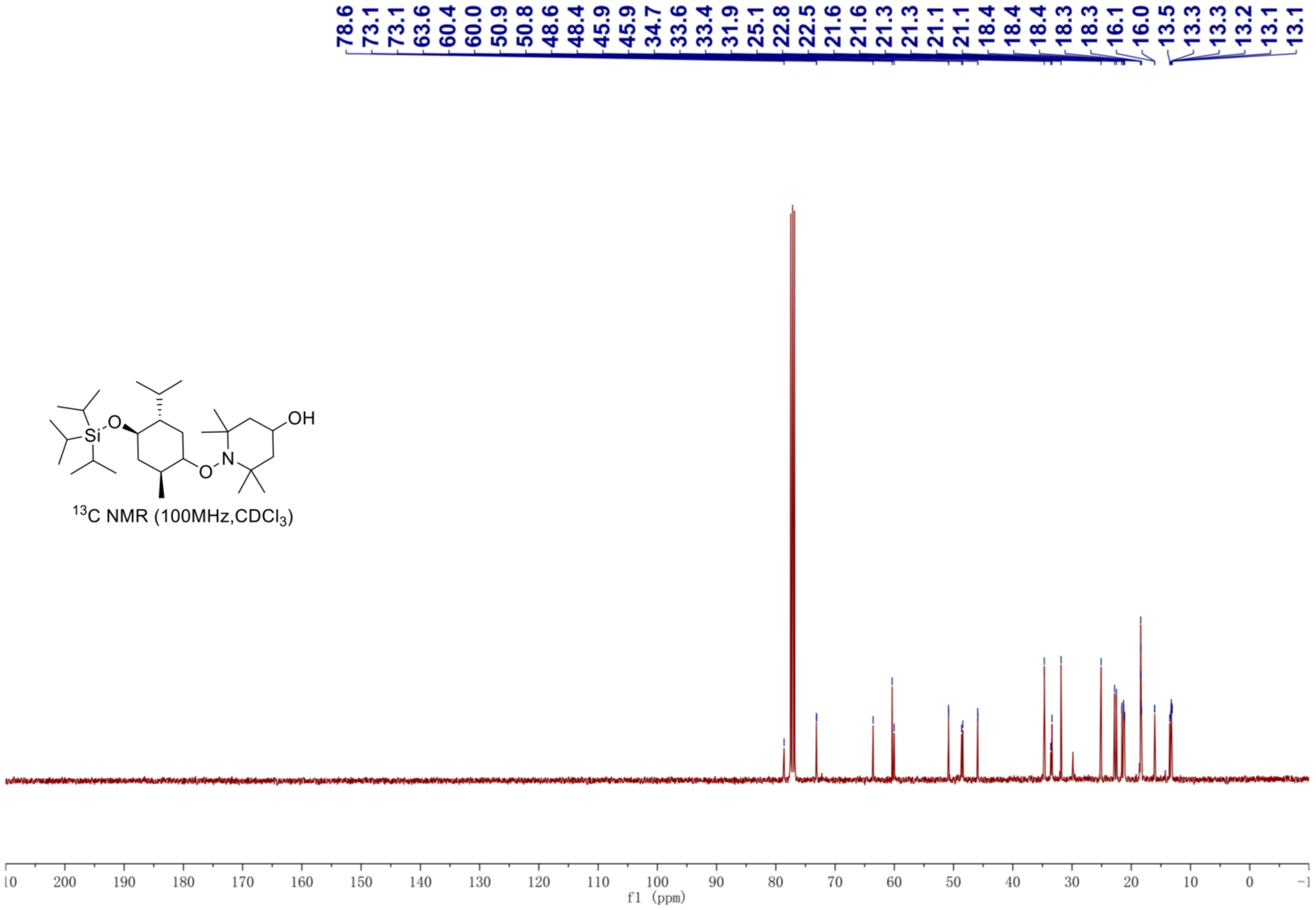

Go to table of contents

6-(((4-Hydroxy-2,2,6,6-tetramethylpiperidin-1-yl)oxy)methyl)-2*H*-chromen-2-one (6b)

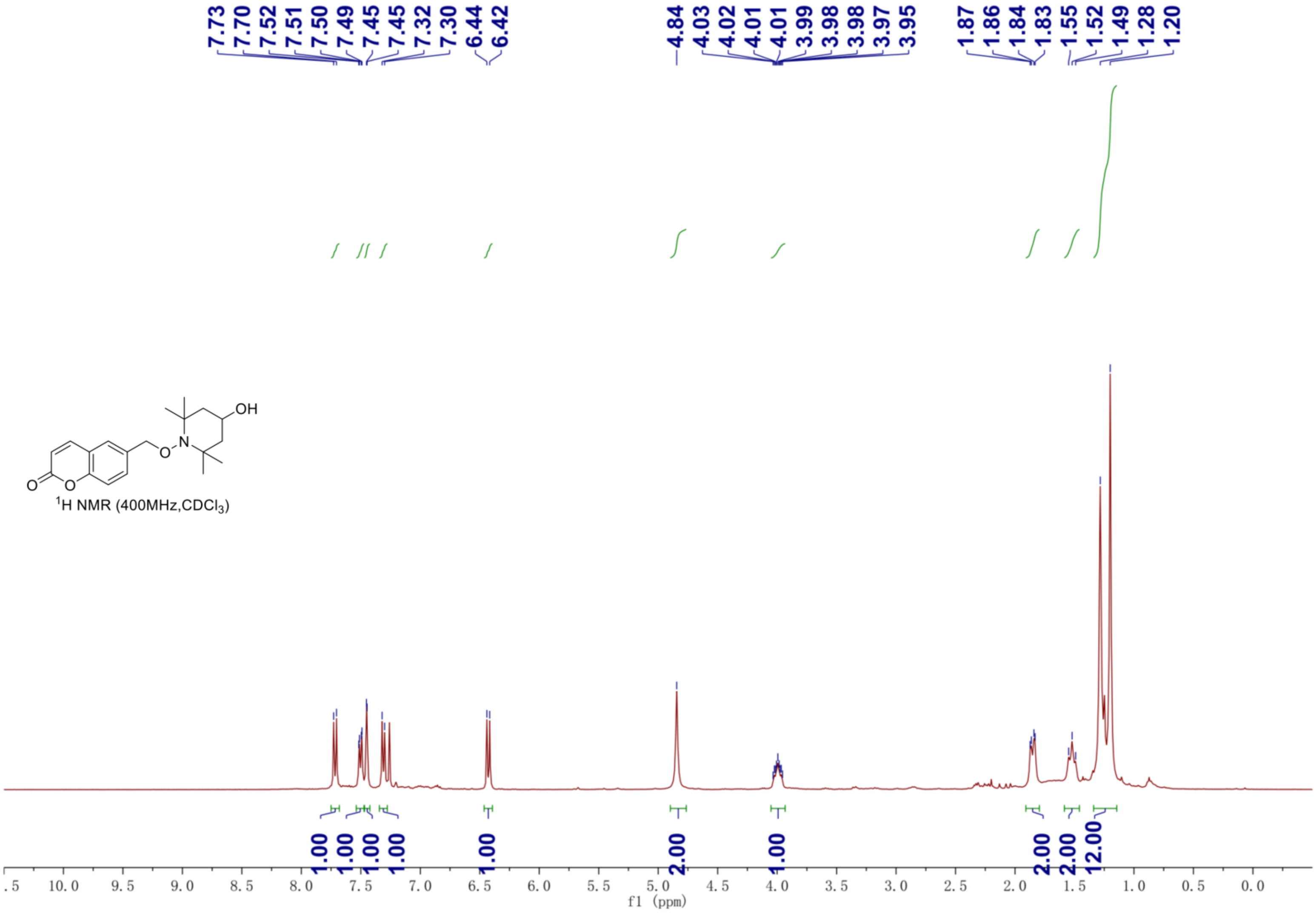

Go to table of contents

6-(((4-Hydroxy-2,2,6,6-tetramethylpiperidin-1-yl)oxy)methyl)-2*H*-chromen-2-one (6b)

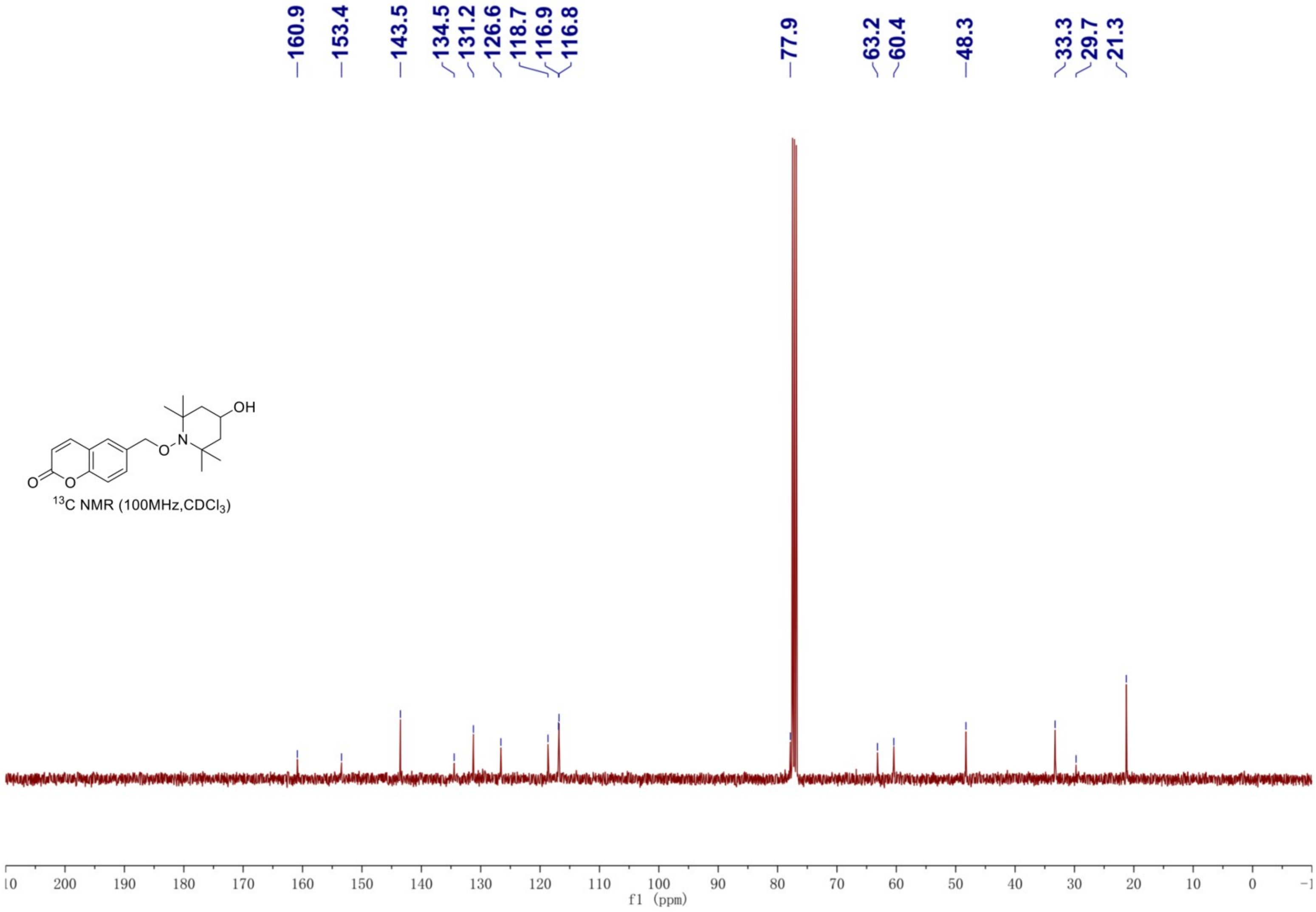

4-(3-(*tert*-Butoxy)-1,1-dicyanopropan-2-yl)phenyl 5-(2,5-dimethylphenoxy)-2,2-dimethylpentanoate (6c)

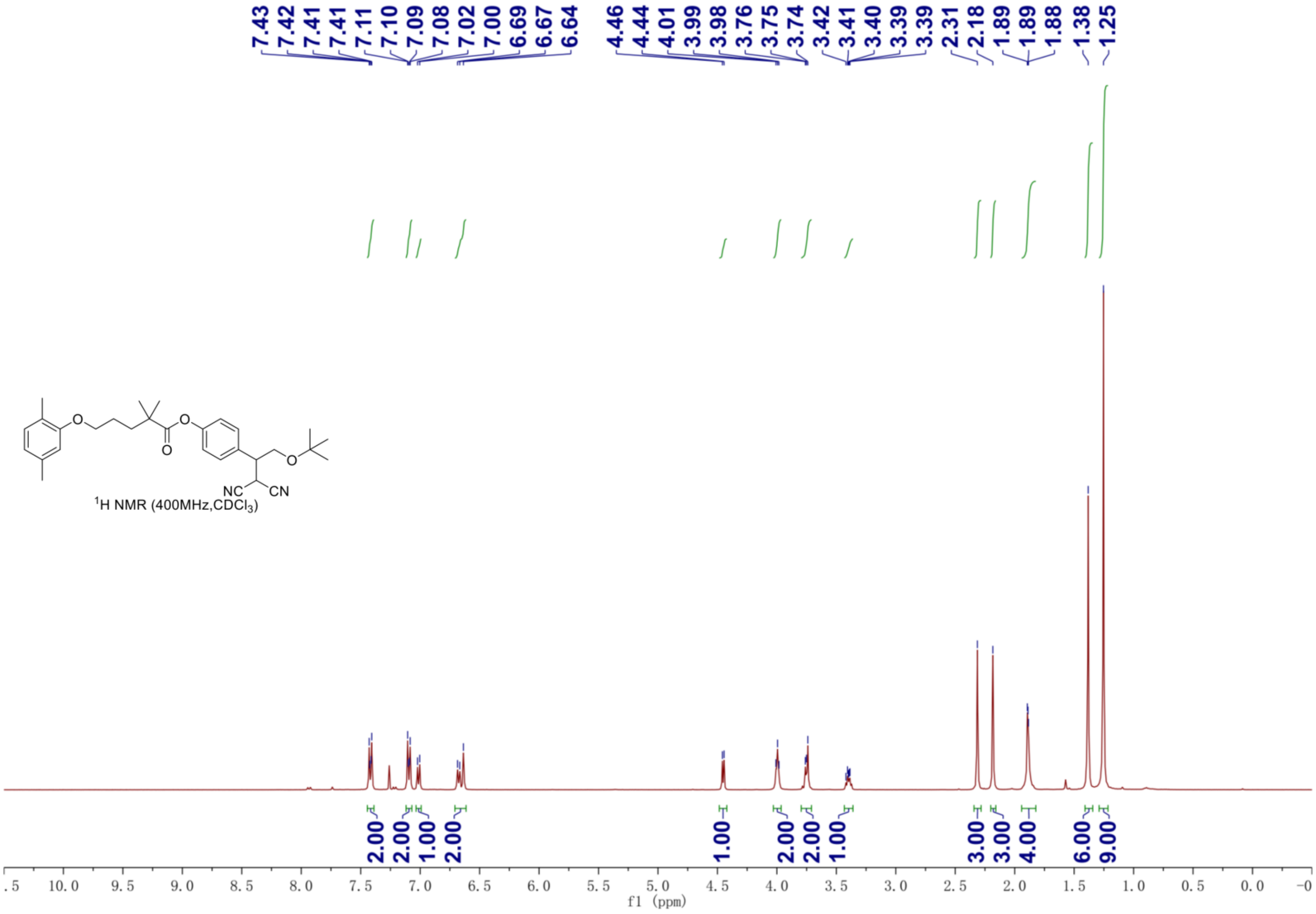

Go to table of contents

4-(3-(*tert*-Butoxy)-1,1-dicyanopropan-2-yl)phenyl 5-(2,5-dimethylphenoxy)-2,2-dimethylpentanoate (6c)

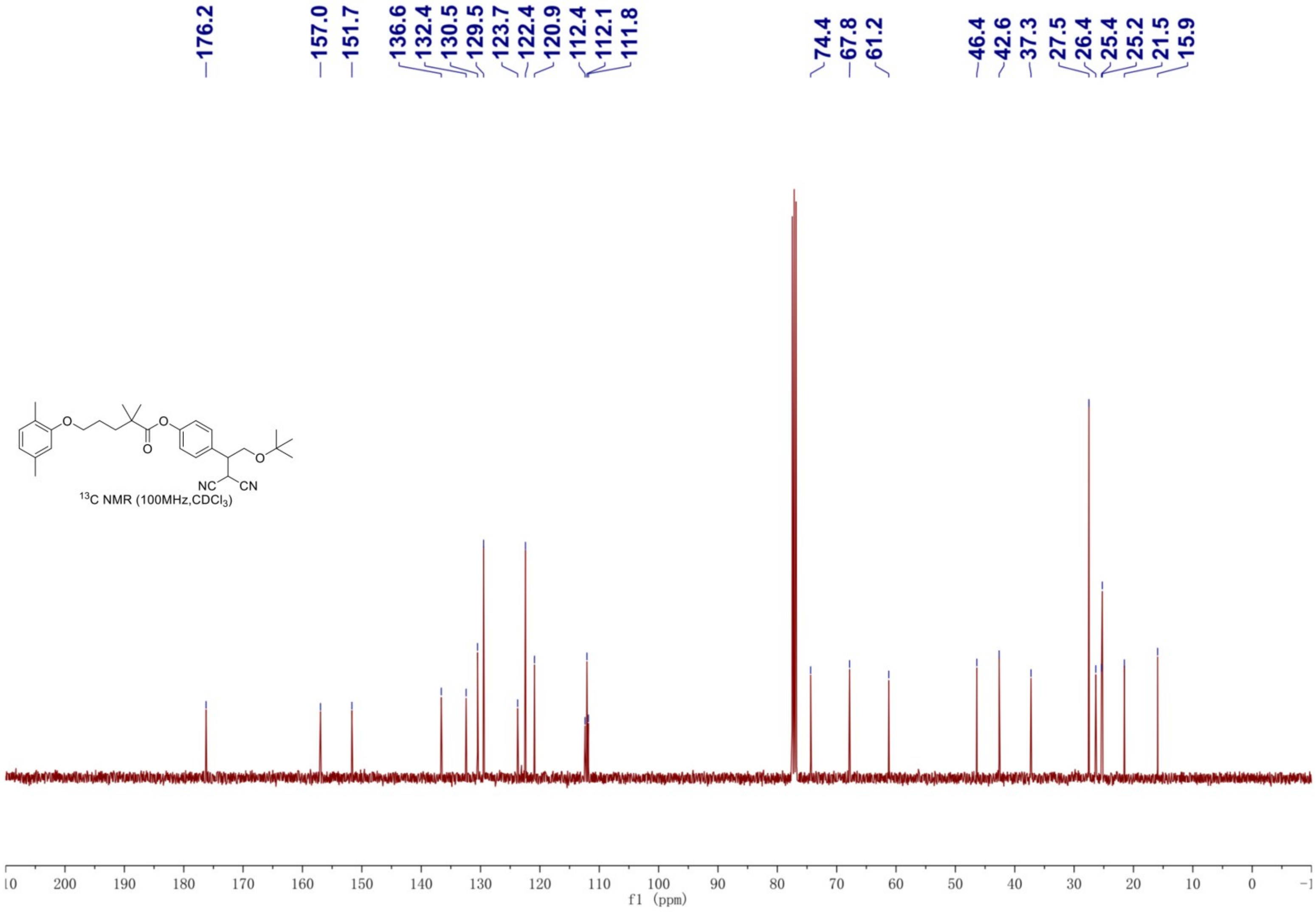

Go to table of contents

Ethyl 2-(4-(2,2-dicyano-1-cyclohexylethyl)phenoxy)-2-methylpropanoate (6d)

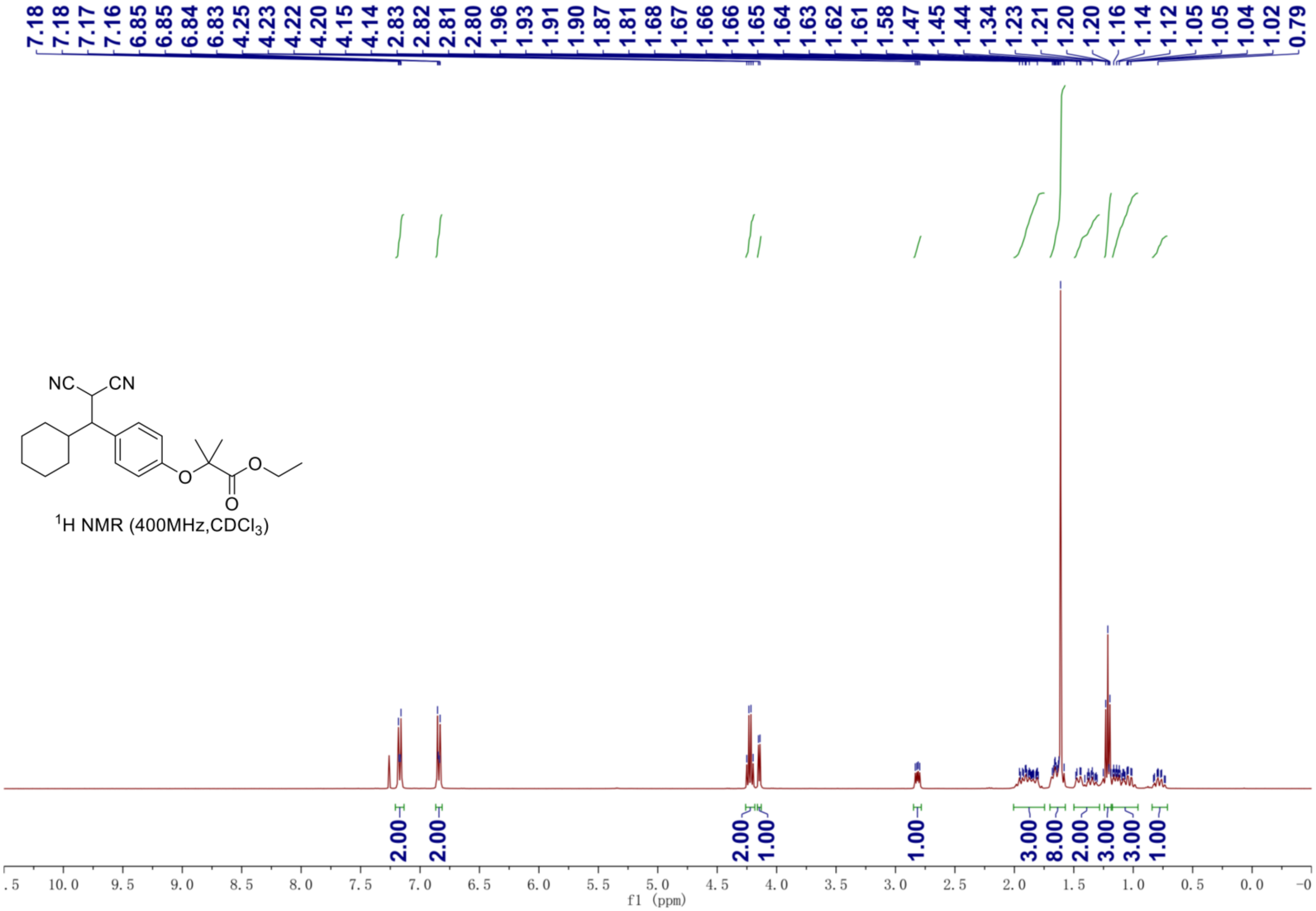

Go to table of contents

Ethyl 2-(4-(2,2-dicyano-1-cyclohexylethyl)phenoxy)-2-methylpropanoate (6d)

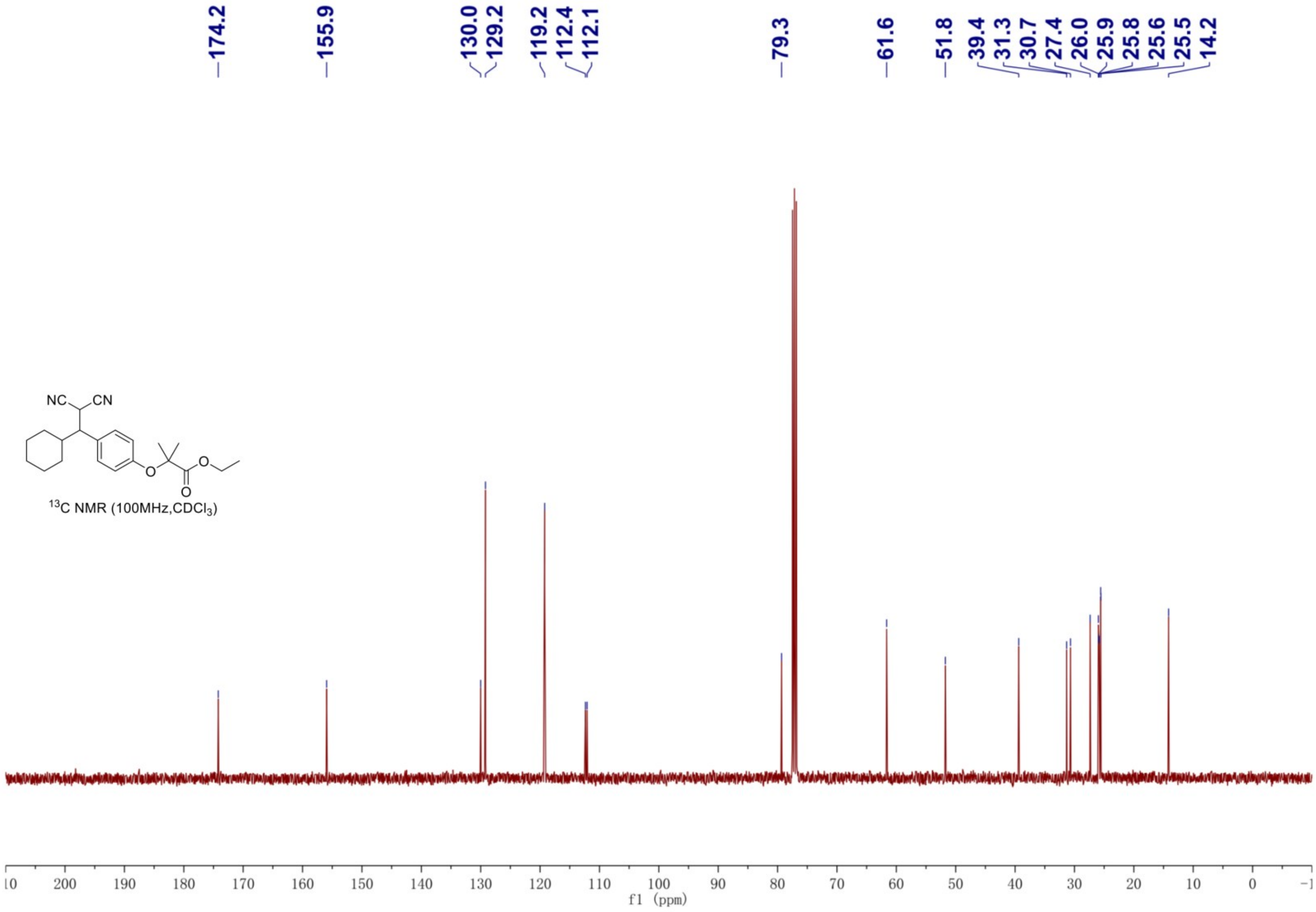

Go to table of contents

Methyl 3-cyclohexyl-2-(11-oxo-6,11-dihydrodibenzo[*b*,*e*]oxepin-2-yl)propanoate (6e)

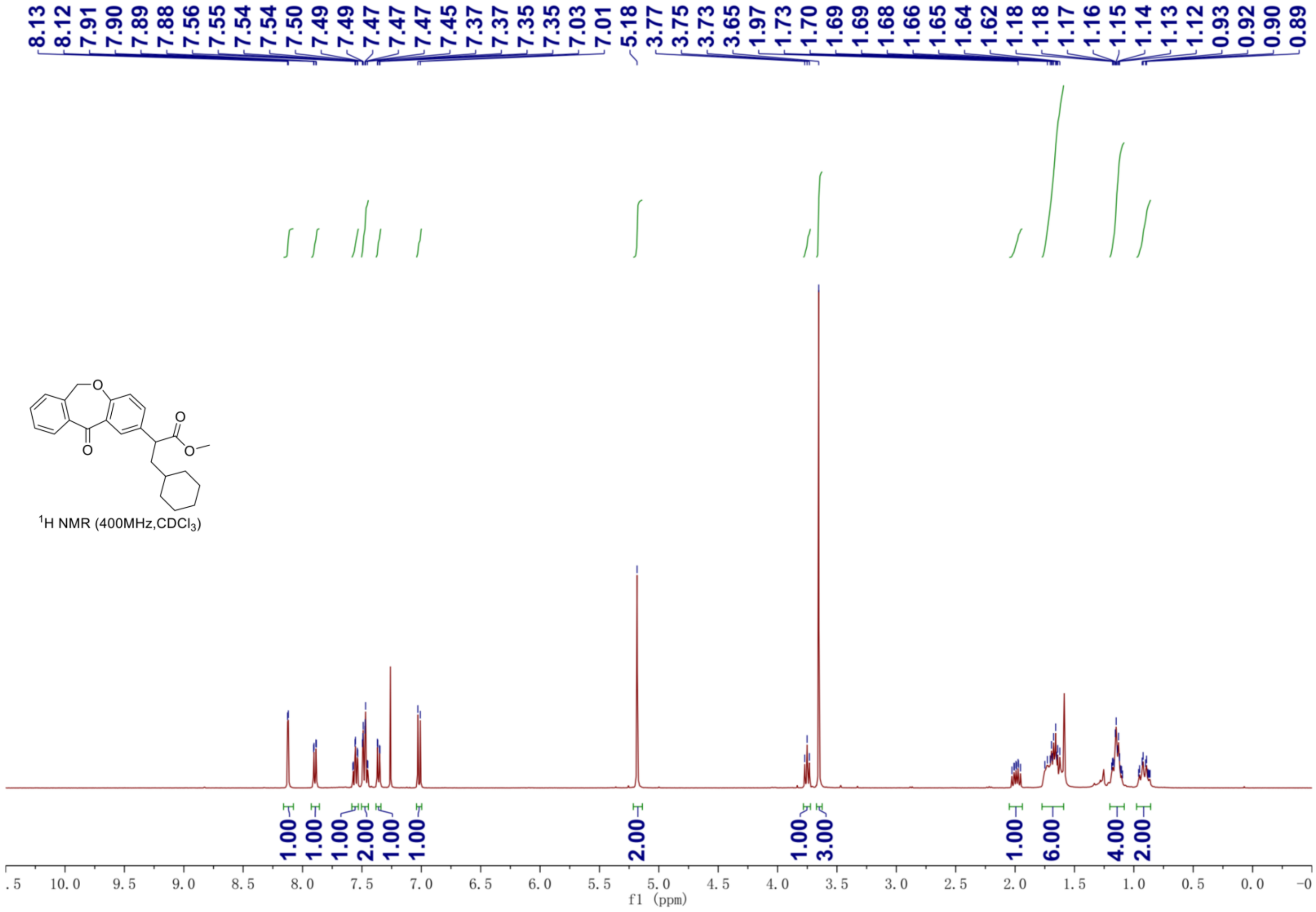

Go to table of contents

Methyl 3-cyclohexyl-2-(11-oxo-6,11-dihydrodibenzo[*b*,*e*]oxepin-2-yl)propanoate (6e)

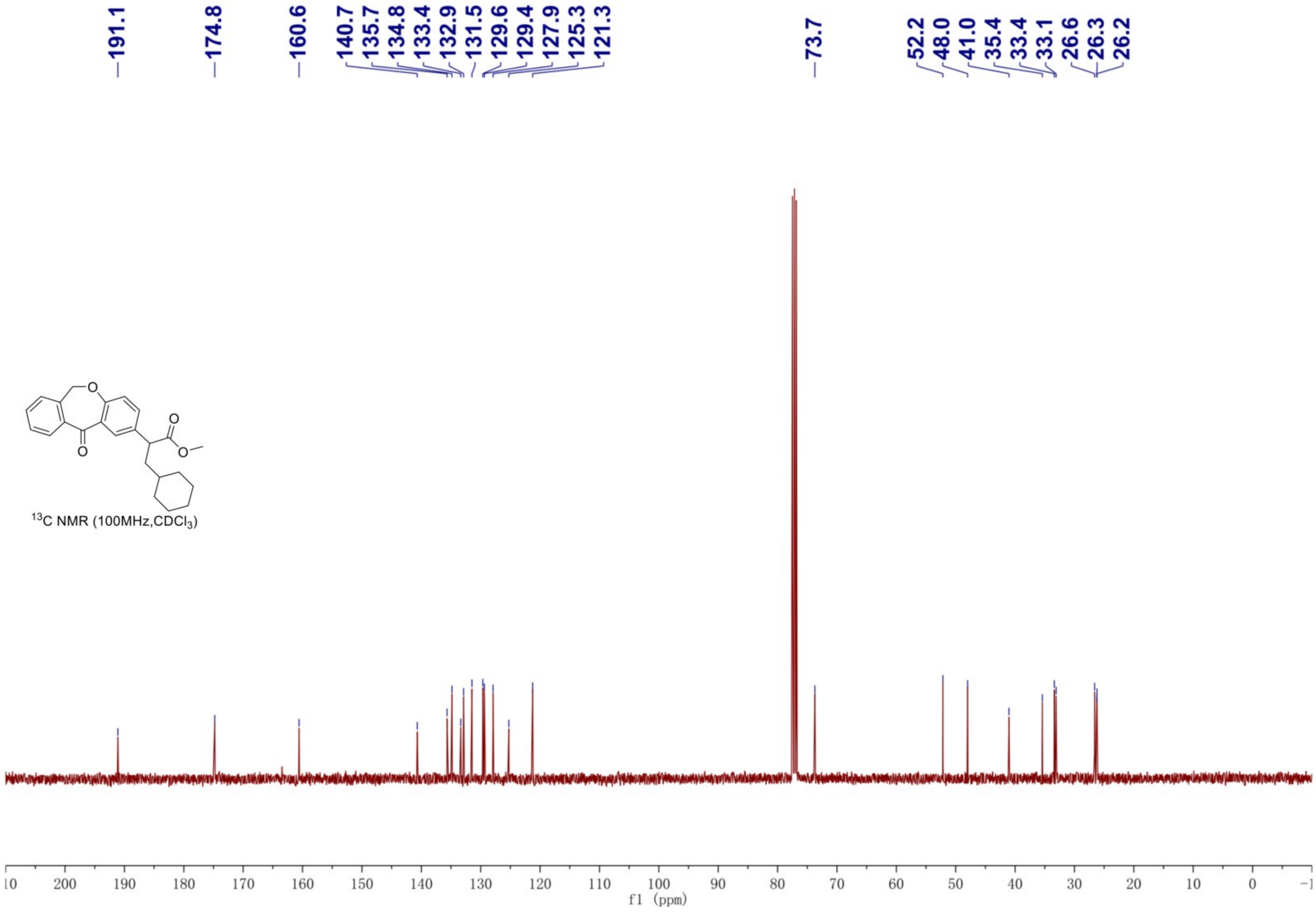

Go to table of contents

4-(2,2-Dicyano-1-cyclohexylethyl)phenyl 3-(4,5-diphenyloxazol-2-yl)propanoate (6f)

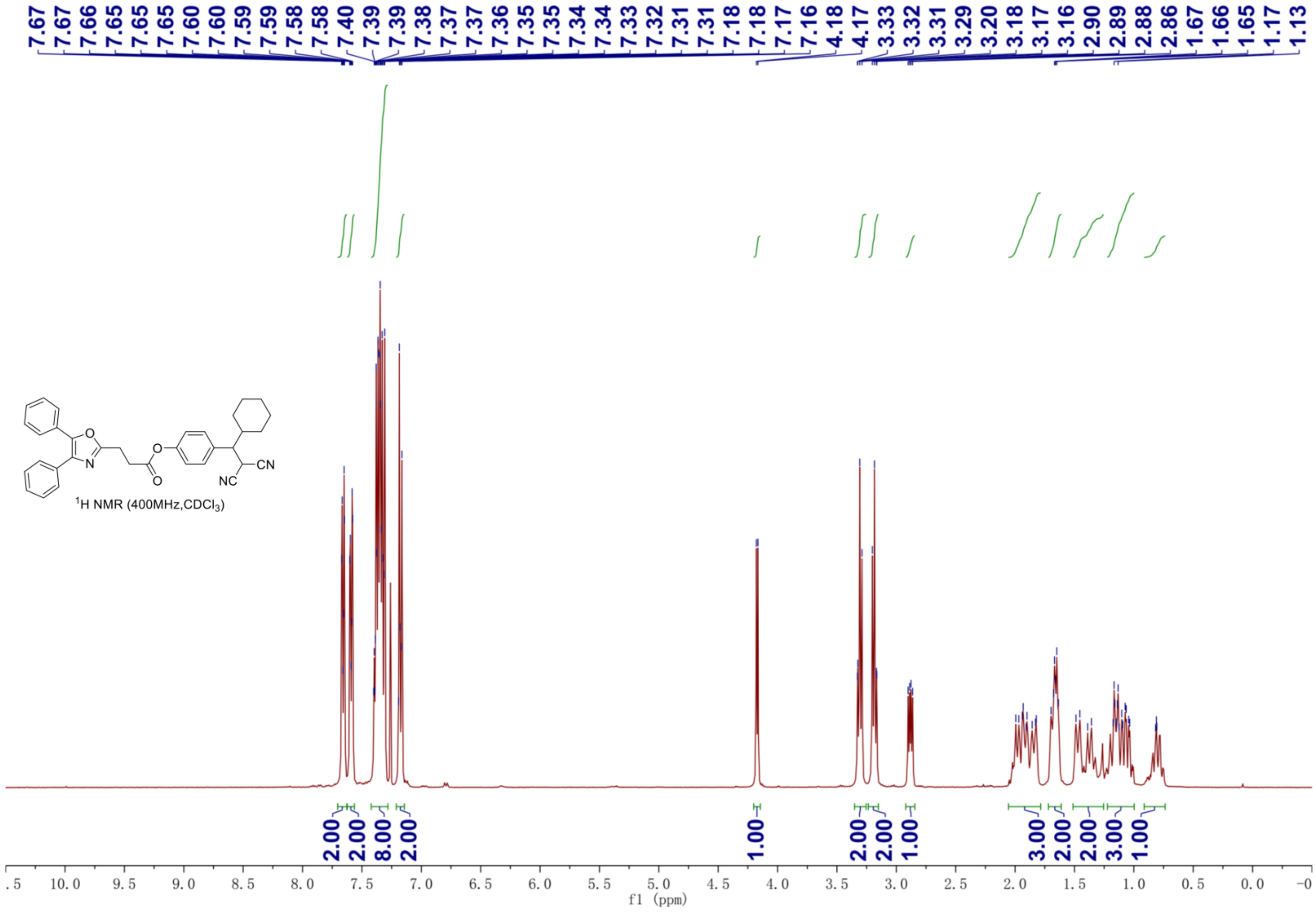

Go to table of contents

4-(2,2-Dicyano-1-cyclohexylethyl)phenyl 3-(4,5-diphenyloxazol-2-yl)propanoate (6f)

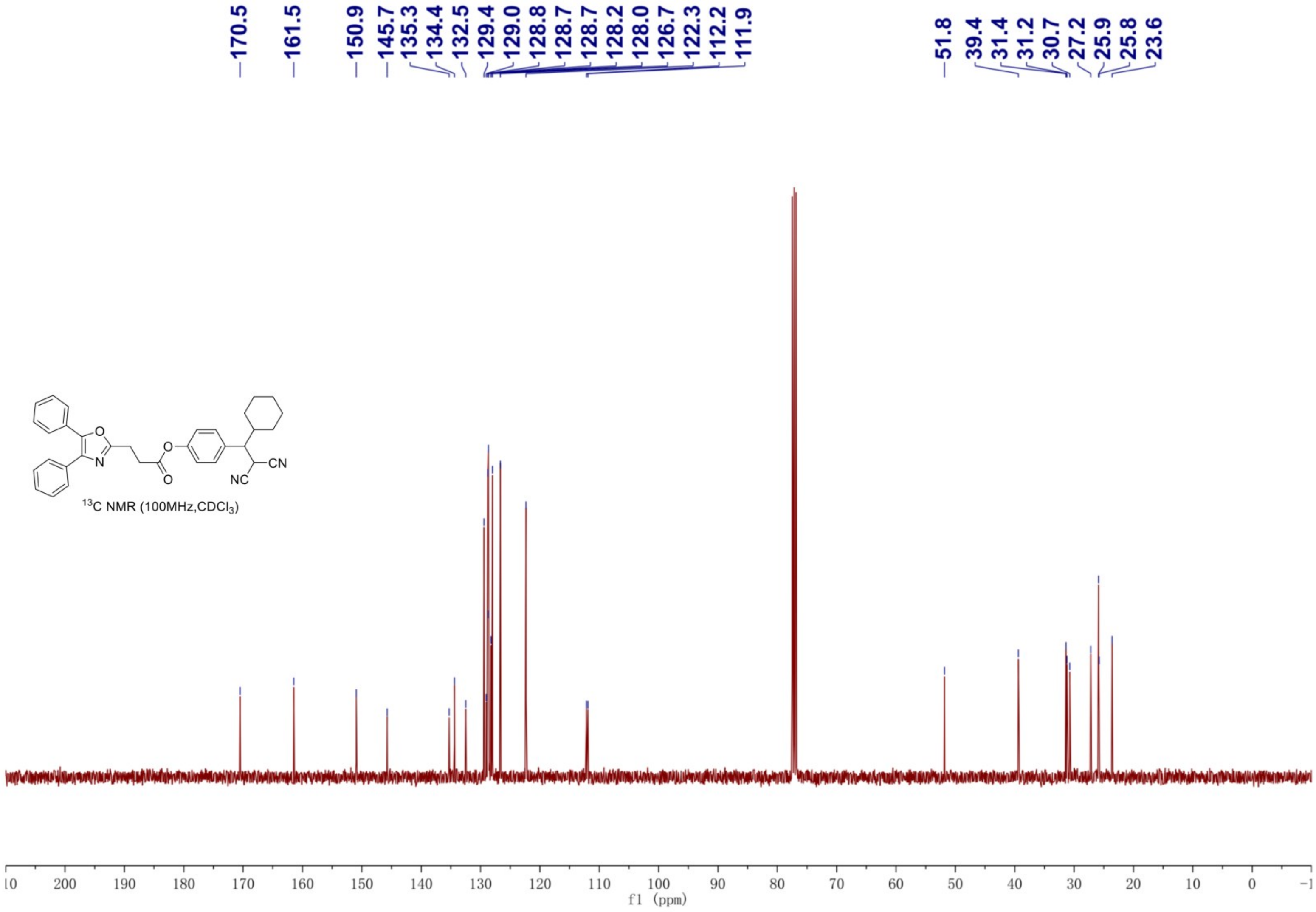

Go to table of contents

Ethyl 3-cyclohexyl-2-(4-isobutylphenyl)propanoate (6g)

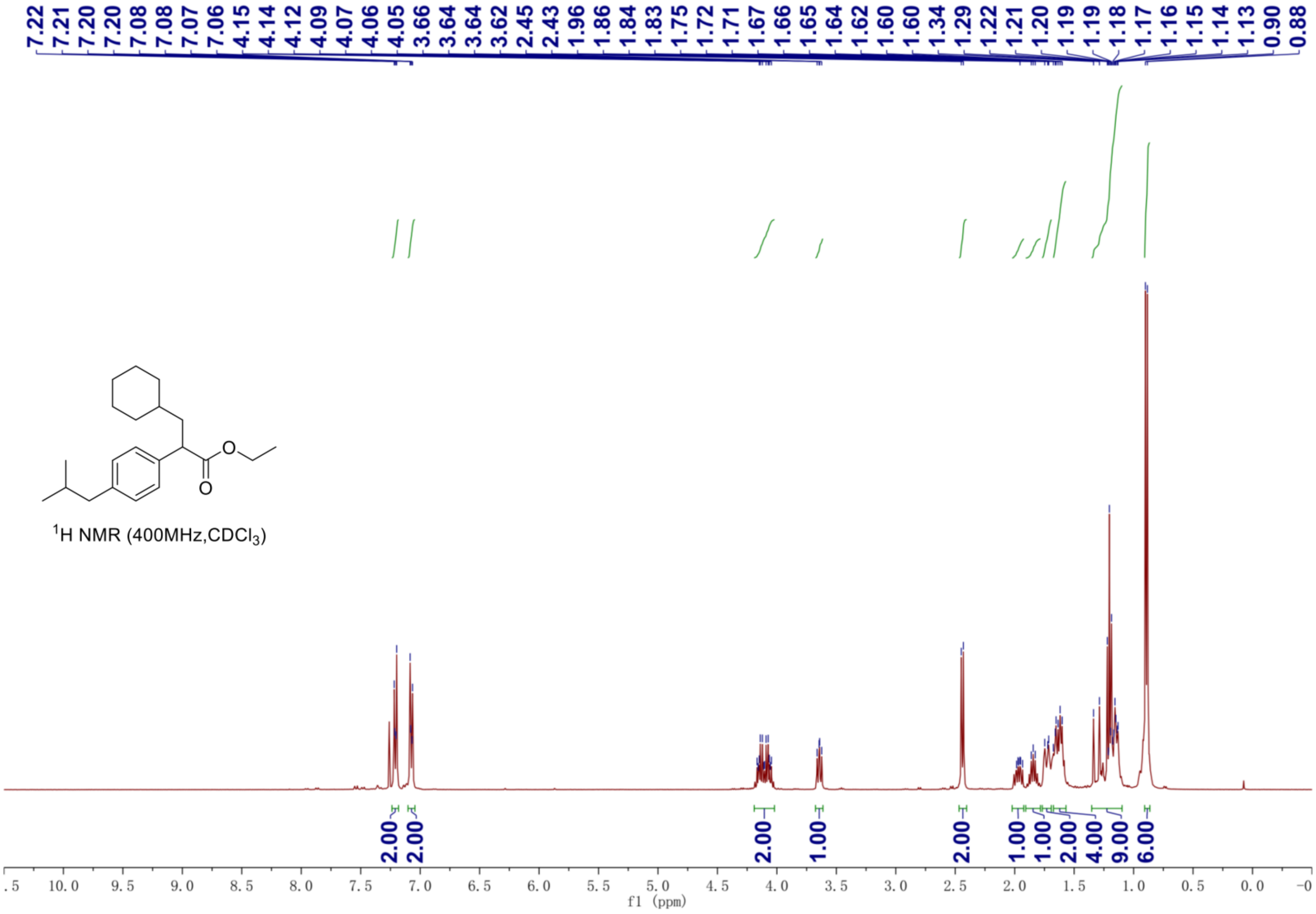

Go to table of contents

Ethyl 3-cyclohexyl-2-(4-isobutylphenyl)propanoate (6g)

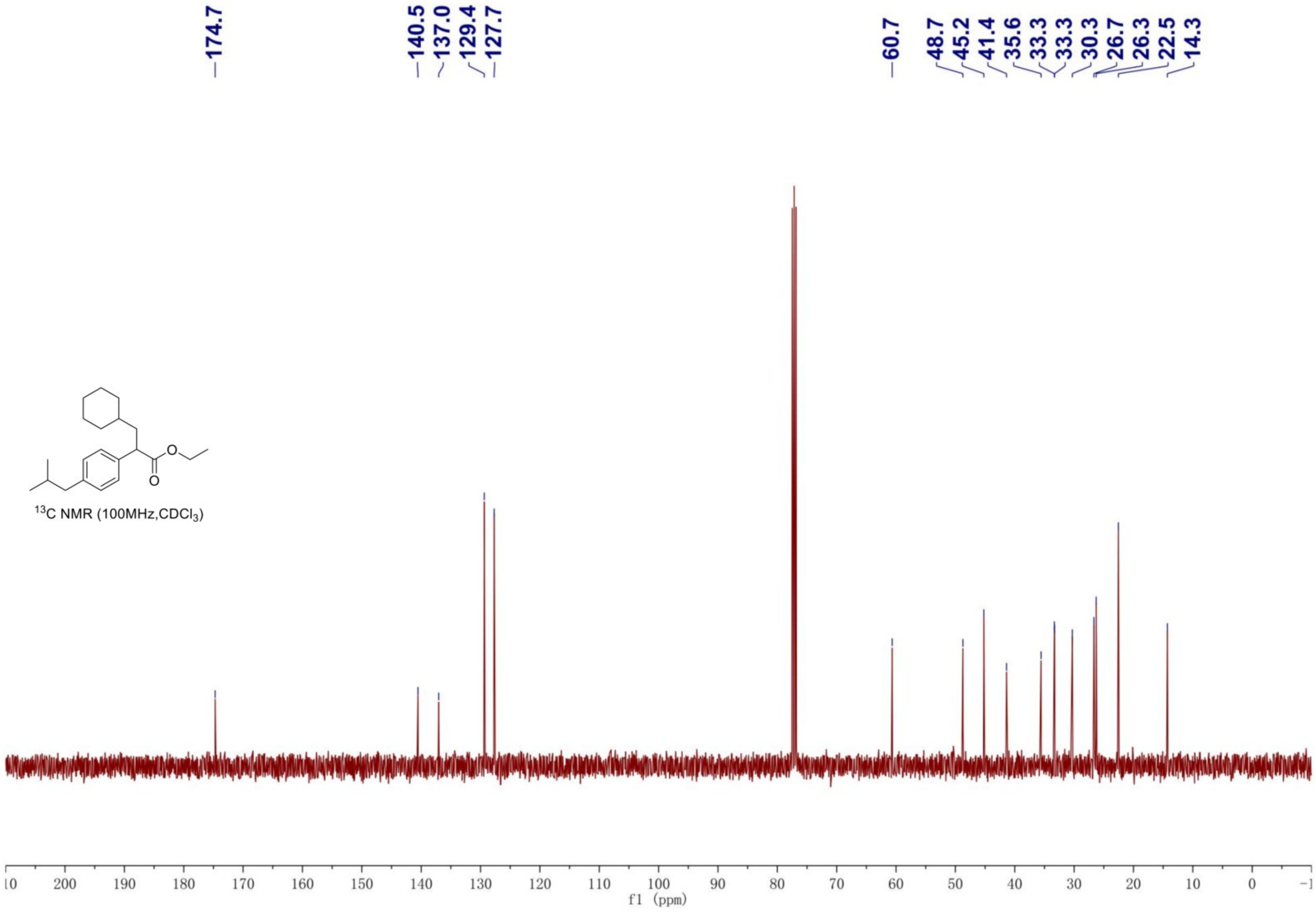

Go to table of contents

N-(4-(1-Butyl-3-ethyl-2,6-dioxopiperidin-3-yl)phenyl)-3-cyclohexyl-2-phenylpropanamide (6h)

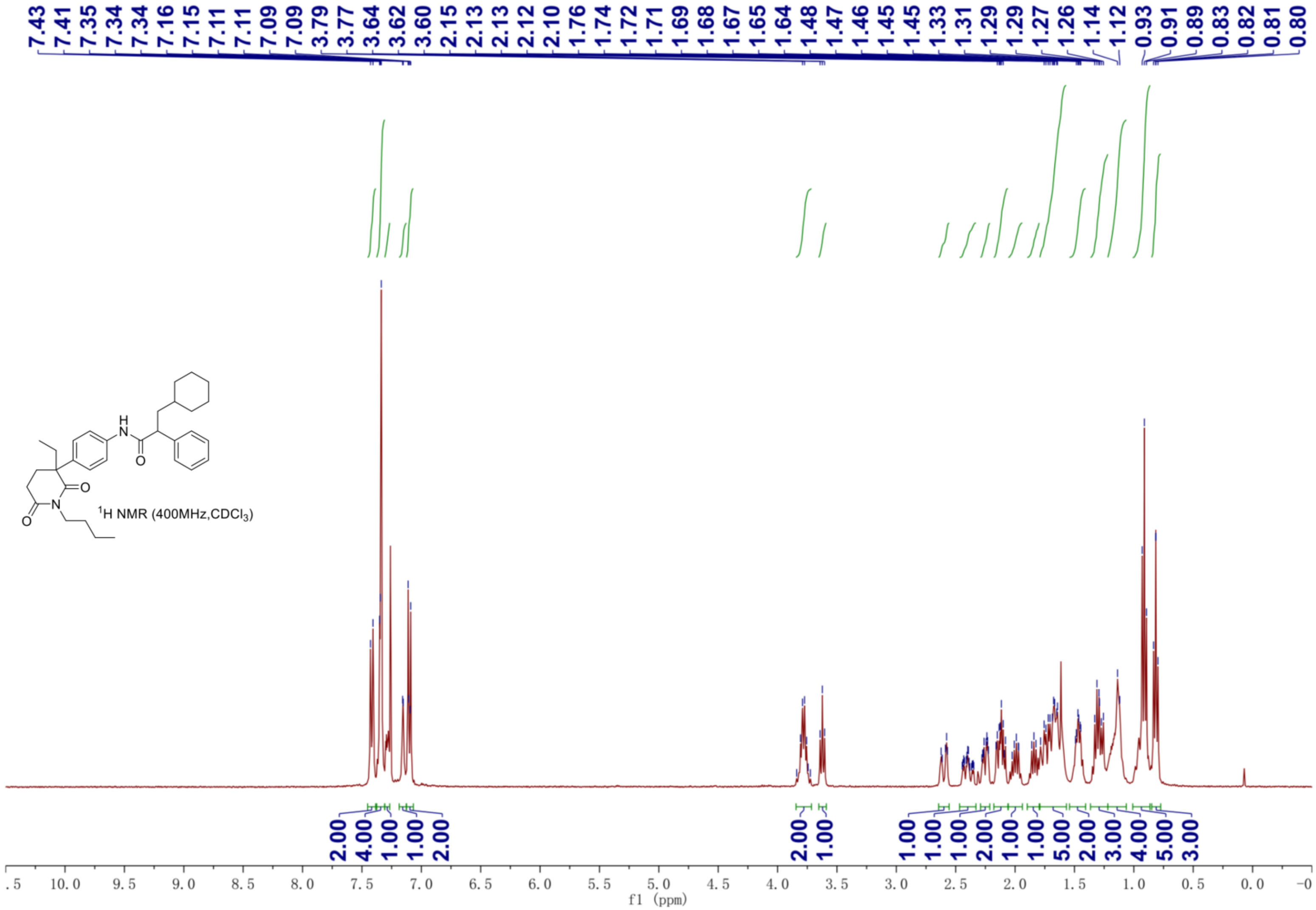

Go to table of contents

N-(4-(1-Butyl-3-ethyl-2,6-dioxopiperidin-3-yl)phenyl)-3-cyclohexyl-2-phenylpropanamide (6h)

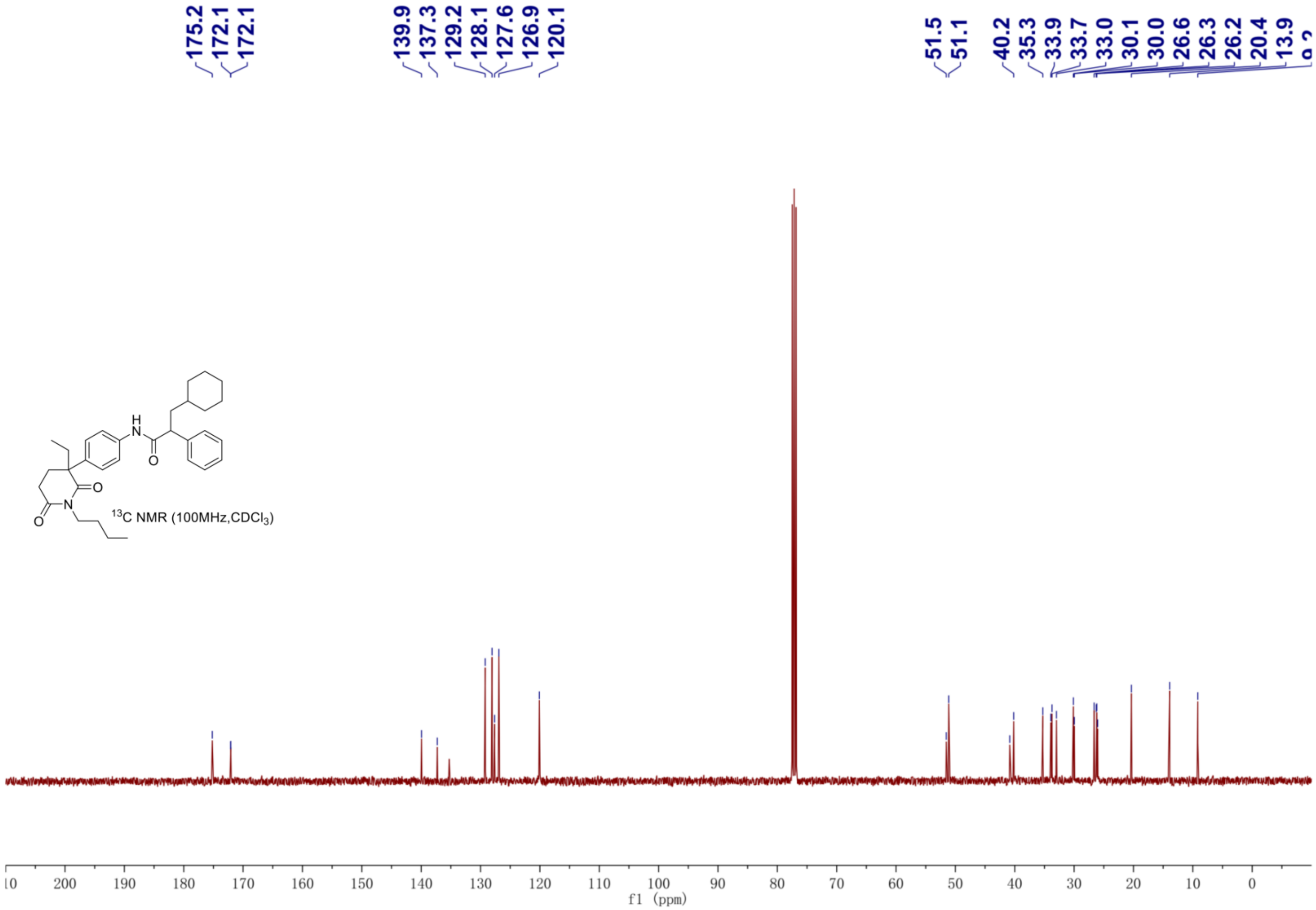

Go to table of contents

References

1. An, Q.; Wang, Z.; Chen, Y.; Wang, X.; Zhang, K.; Pan, H.; Liu, W.; Zuo, Z. Cerium-Catalyzed C–H Functionalizations of Alkanes Utilizing Alcohols as Hydrogen Atom Transfer Agents. *J. Am. Chem. Soc.* **2020**, *142*, 6216–6226.

2. Frost, C. G.; Hartley, B. C. Lewis Base-Promoted Hydrosilylation of Cyclic Malonates: Synthesis of β-Substituted Aldehydes and γ-Substituted Amines. *J. Org. Chem.* **2009**, *74*, 3599–3602.

3. Duan, Z.; Liu, M.; Zheng, B.; Tang, Y.; Du, J.; Wang, L.; Yu, S.; Wu, Y.; Guo, H. Phosphine-Catalyzed Tandem Annulation of Allenylic Alcohols with 1,1-Dicyanoalkenes. *Org. Lett.* **2023**, *25*, 3298–3302.

4. Li, Q.; Chen, J.; Luo, Y.; Xia, Y. Photoredox-Catalyzed Hydroacylation of Azobenzenes with Carboxylic Acids. *Org. Lett.* **2024**, *26*, 1517–1521.

5. Mao, J.-H.; Wang, Y.-B.; Yang, L.; Xiang, S.-H.; Wu, Q.-H.; Cui, Y.; Lu, Q.; Lv, J.; Li, S.; Tan, B. Organocatalyst-controlled site-selective arene C–H functionalization. *Nat. Chem.* **2021**, *13*, 982–991.

6. H. D. Pickford, J. Nugent, B. Owen, J. J. Mousseau, R. C. Smith, E.A. Anderson, *J. Am. Chem. Soc.* **2021**, *143*, 9729–9736.

7. Lipilin, D. L.; Zubkov, M. O.; Kosobokov, M. D.; Dilman, A. D. Direct conversion of carboxylic acids to free thiols via radical relay acridine photocatalysis enabled by N–O bond cleavage. *Chem. Sci.* **2023**, *15*, 644–650.

8. M. O. Zubkov, M. D. Kosobokov, V. V. Levin, A. D. Dilman, *Org. Lett.* **2022**, *24*, 2354–2358.

9. Gaussian 16, Revision C.01, Frisch, M. J.; Trucks, G. W.; Schlegel, H. B.; Scuseria, G. E.; Robb, M. A.; Cheeseman, J. R.; Scalmani, G.; Barone, V.; Petersson, G. A.; Nakatsuji, H.; Li, X.; Caricato, M.; Marenich, A. V.; Bloino, J.; Janesko, B. G.; Gomperts, R.; Mennucci, B.; Hratchian, H. P.; Ortiz, J. V.; Izmaylov, A. F.; Sonnenberg, J. L.; Williams-Young, D.; Ding, F.; Lipparini, F.; Egidi, F.; Goings, J.; Peng, B.; Petrone, A.; Henderson, T.; Ranasinghe, D.; Zakrzewski, V. G.; Gao, J.; Rega, N.; Zheng, G.; Liang, W.; Hada, M.; Ehara, M.; Toyota, K.; Fukuda, R.; Hasegawa, J.; Ishida, M.; Nakajima, T.; Honda, Y.; Kitao, O.; Nakai, H.; Vreven, T.; Throssell, K.; Montgomery, J. A., Jr.; Peralta, J. E.; Ogliaro, F.; Bearpark, M. J.; Heyd, J. J.; Brothers, E. N.; Kudin, K. N.; Staroverov, V. N.; Keith, T. A.; Kobayashi, R.; Normand, J.; Raghavachari, K.; Rendell, A. P.; Burant, J. C.; Iyengar, S. S.; Tomasi, J.; Cossi, M.; Millam, J. M.; Klene, M.; Adamo, C.; Cammi, R.; Ochterski, J. W.; Martin, R. L.; Morokuma, K.; Farkas, O.; Foresman, J. B.; Fox, D. J. Gaussian, Inc., Wallingford CT, 2019.

10. Chemcraft – graphical software for visualization of quantum chemistry computations. Version 1.8, build 682, https://www.chemcraftprog.com.

11. C Y. Legault. CYLview 2.0, http://www.cylview.org, 2009.

12. Humphrey, W.; Dalke, A.; Schulten, K. VMD – Visual Molecular Dynamics. *J. Molec. Graphics*. **1996**, *14*, 33–38.

13. G. Luchini, J. V. Alegre-Requena, Y. Guan, I. Funes-Ardoiz, R. S. Paton, GoodVibes v3.0.1, 2019.

14. Garrett, B. C.; Truhlar, D. G. Accuracy of tunneling corrections to transition state theory for thermal rate constants of atom transfer reactions. *J. Phys. Chem.* **1979**, *83*, 200–203.

15. Wigner, E. The transition state method. *Trans. Faraday Soc*. **1938**, *34*, 29–41.

Go to table of contents

16. Matxain, J. M.; Huertos, M. A. Hydrogen Tunneling in Stoichiometric and Catalytic Reactions involving Transition Metals. *ChemCatChem* **2023**, *15*, e202300962.

17. For the details of electron–hole analysis, see Supplemental Information for: Jin, S.; Sui, X.; Haug, G. C.; Nguyen, V. D.; Dang, H. T.; Armon, H. D.; Larionov, O. V. N-Heterocyclic Carbene-Photocatalyzed Tricomponent Regioselective 1,2-Diacylation of Alkenes Illuminates the Mechanistic Details of the Electron Donor–Acceptor Complex-Mediated Radical Relay Processes. *ACS Catal*. **2022**, *12*, 285–294.

18. Isse, A. A.; Gennaro, A. Absolute Potential of the Standard Hydrogen Electrode and the Problem of Interconversion of Potentials in Different Solvents. *J. Phys. Chem. B.* **2010**, *114*, 7894–7899.

19. (a) Khaliullin, R. Z.; Cobar, E. A.; Lochan, R. C.; Bell, A. T.; Head-Gordon, M. Unravelling the Origins of Intermolecular Interactions Using Absolutely Localized Molecular Orbitals. *J. Phys. Chem. A* **2007**, *111*, 8753–8765. (b) Horn, P. R.; Mao, Y.; Head-Gordon, M. Probing Non-Covalent Interactions with a Second-Generation Energy Decomposition Analysis Using Absolutely Localized Molecular Orbitals. *Phys. Chem. Chem. Phys.* **2016**, *18*, 23067−23079.

20. For the details of the calculations, see Supplemental Information for: Jin, S.; Haug, G. C.; Trevino, R.; Nguyen, V. D.; Arman, H. D.; Larionov, O. V.; Photoinduced C(sp^3)–H sulfination empowers the direct and chemoselective introduction of the sulfonyl group. *Chem. Sci*. **2021**, 12, 13914–13921.

21. Shao, Y.; Gan, E; Epifanovsky, A.; Gilbert, A . T. B.; Wormit, M.; Kussmann, J.; Lange, A. W.; Behn, A.; Deng, J.; Feng, X.; Ghosh, D.; Goldey, M.; Horn, P. R.; Jacobson, L. D.; Kaliman, I.; Khaliullin, R. Z.; Kús, T.; Landau, A.; Liu, J.; Proynov, E. I.; Rhee, Y. M.; Richard, R. M.; Rohrdanz, M. A.; Steele, R. P.; Sundstrom, E. J.; Woodcock III, H. L.; Zimmerman, P. M.; Zuev, D.; Albrecht, B.; Alguire, E.; Austin, B.; Beran, G. J. O.; Bernard, Y. A.; Berquist, E.; Brandhorst, K.; Bravaya, K. B.; Brown, S. T.; Casanova, D.; Chang, C.-M.; Chen, Y.; Chien, S. H.; Closser, K. D.; Crittenden, D. L.; Diedenhofen, M.; DiStasio Jr., R. A.; Dop, H.; Dutoi, A. D.; Edgar, R. G.; Fatehi, S.; Frusti-Molnar, L.; Ghysels, A.; Golubeva-Zadorozhnaya, A.; Gomes, J.; Hanson- Heine, M. W. D.; Harbach, P. H. P.; Hauser, A. W.; Hohenstein, E. G.; Holden, Z. C.; Jagau, T.-C.; Ji, H.; Kaduk, B.; Khistyaev, K.; Kim, J.; Kim, J.; King, R. A.; Klunzinger, P.; Kosenkov, D.; Kowalczyk, T.; Krauter, C. M.; Lao, K. U.; Laurent, A.; Lawler, K. V.; Levchenko, S. V.; Lin, C. Y.; Liu, F.; Livshits, E.; Lochan, R. C.; Luenser, A.; Manohar, P.; Manzer, S. F.; Mao, S.-P.; Mardirossian, N.; Marenich, A. V.; Maurer, . A.; Mayhall, N. J. Oana, C. M.; Olivares-Amaya, R.; O'Neill, D. P.; Parkhill, J. A.; Perrine, T. M.; Peverati, R.; Pieniazek, P. A.; Prociuk, A.; Rehn, D. R.; Rosta, E.; Russ, N. J.; Sergueev, N.; Sharada, S. M.; Sharmaa, S.; Small, D. W.; Sodt, A.; Stein, T.; Stück, D.; Su, Y.-C.; Thom, A. J. W.; Tsuchimochi, T.; Vogt, L.; Vydrov, O.; Wang, T.; Watson, M. A.; Wenzel, J.; White, A.; Williams, C. F.; Vanovschi, V.; Yeganeh, S.; Yost, S. R.; You, Z.-Q.; Zhang, Y.; Zhang, X.; Zhou, Y.; Brooks, B. R.; Chan, G. K. L.; Chipman, D. M.; Cramer, C. J.; Goddard III, W. A.; Gordon, M. S.; Hehre, W. J.; Klamt, A.; Schaefer III, H. F.; Schmidt, M. W.; Sherrill, C. D.; Truhlar, D. G.; Warshel, A.; Xua, X.; Aspuru-Guzik, A.; Baer, R.; Bell, A. T.; Besley, N. A.; Chai, J.-D.; Dreuw, A.; Dunietz, B. D.; Furlani, T. R.; Gwaltney, S. R.; Hsu, C.-P.; Jung, Y.; Kong, J.; Lambrecht, D. S.; Liang, W.; Ochsenfeld, C.; Rassolov, V. A.; Slipchenko, L. V.; Subotnik, J. E.; Van Voorhis, T.; Herbert, J. M.; Krylov, A. I.; Gill, P. M. W.; Head- Gordon, M. Advances in Molecular Quantum Chemistry Contained in the Q-Chem 4 Program Package. Mol. Phys. 2015, 113, 184–215

22. Lu, F.; Chen, F. Multiwfn: A multifunctional wavefunction analyzer. *J. Comp. Chem.,* **2012**, *33*, 580–592.

23. For the details of the Marcus theory calculations, see Supplemental Information for: Sui, X.; Dang, H. T.; Porey, A.; Trevino, R.; Das, A.; Fremin, S. O.; Hughes, W. B.; Thompson, W. T.; Dhakal, S. K.; Arman, H. D.; Larionov, O. V. Acridine photocatalysis enables tricomponent direct decarboxylative amine construction. *Chem. Sci***.**, **2024** ,*15*, 9582–9590.

24. a) Fifen, J.J.; Nsangou, M.; Dhaouadi, Z.; Motapon, O.; Jaidane, N. Solvent effects on the antioxidant activity of 3,4-dihydroxyphenypyruvic acid: DFT and TD-DFT studies. *Computational and Theoretical Chemistry* **2011**, *966*, 232-243; b) Rohatgi-Mukherjee, K.K. Fundamentals of Photochemistry, Wiley, New York, 1978.
